# Population matched (pm) germline allelic variants of immunoglobulin (*IG*) loci: Relevance in infectious diseases and vaccination studies in human populations

**DOI:** 10.1038/s41435-021-00143-7

**Published:** 2021-06-12

**Authors:** Indu Khatri, Magdalena A. Berkowska, Erik B. van den Akker, Cristina Teodosio, Marcel J. T. Reinders, Jacques J. M. van Dongen

**Affiliations:** 1 Department Immunology, Leiden University Medical Center, Leiden, The Netherlands; 2 Leiden Computational Biology Center, Leiden University Medical Center, Leiden, The Netherlands; 3 Department Molecular Epidemiology, Leiden University Medical Center, Leiden, The Netherlands; 4 Delft Bioinformatics Lab, Delft University of Technology, Delft, The Netherlands

**Keywords:** Immunogenetics, Haplotypes, Population genetics, Adaptive immunity

## Abstract

Immunoglobulin (*IG*) loci harbor inter-individual allelic variants in many different germline *IG* variable, diversity and joining genes of the *IG* heavy (*IGH*), kappa (*IGK*) and lambda (*IGL*) loci, which together form the genetic basis of the highly diverse antigen-specific B-cell receptors. These allelic variants can be shared between or be specific to human populations. The current immunogenetics resources gather the germline alleles, however, lack the population specificity of the alleles which poses limitations for disease-association studies related to immune responses in different human populations. Therefore, we systematically identified germline alleles from 26 different human populations around the world, profiled by “1000 Genomes” data. We identified 409 *IGHV*, 179 *IGKV*, and 199 *IGLV* germline alleles supported by at least seven haplotypes. The diversity of germline alleles is the highest in Africans. Remarkably, the variants in the identified novel alleles show strikingly conserved patterns, the same as found in other *IG* databases, suggesting over-time evolutionary selection processes. We could relate the genetic variants to population-specific immune responses, e.g. *IGHV1-69* for flu in Africans. The population matched *IG* (pmIG) resource will enhance our understanding of the SHM-related B-cell receptor selection processes in (infectious) diseases and vaccination within and between different human populations.

## Introduction

**Fig. 1:**
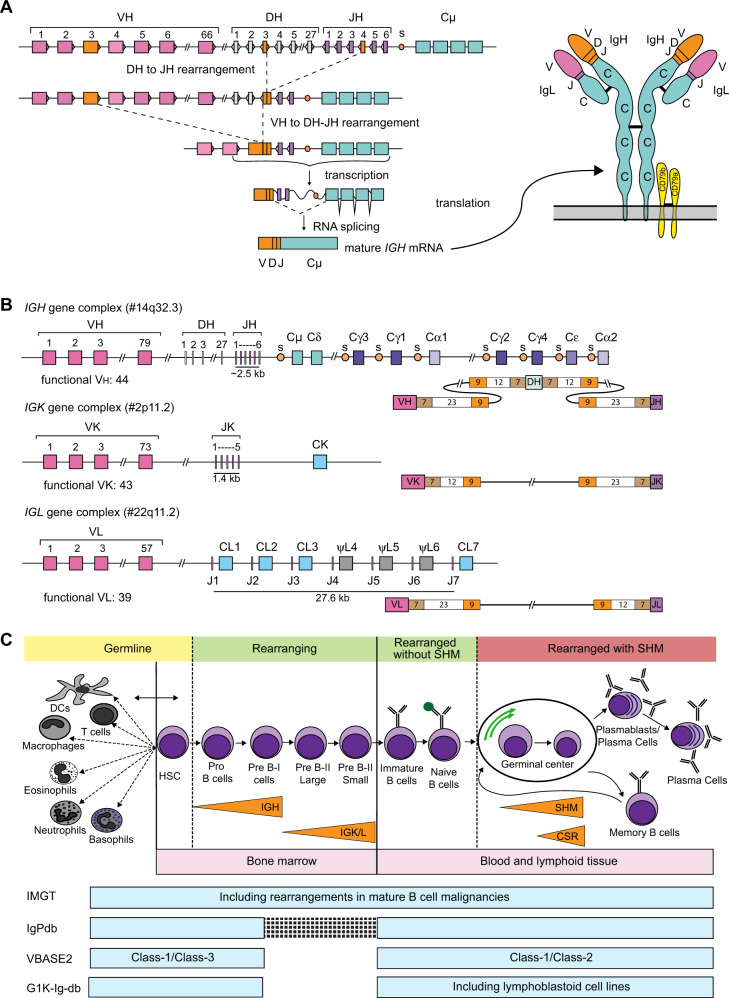
Generation and assessment of diversity in *IG* loci. **A** In the first step of *V(D)J* recombination in the *IGH* locus a *D* gene is coupled to a *J* gene. Subsequently, a *V* gene is coupled to the *DJ* joint. The *VDJ* exon is transcribed and spliced to the *IGHM* exons. An analogous process takes place in the Ig light chain genes. When a functional IgM protein is transported to the plasma membrane with anchoring molecules CD79a and CD79b and assembled with a functional Ig light chain, it forms a complete antibody molecule. **B** Schematic overview of the three *IG* loci: *IGH*, *IGK*, *IGL* and the structure of their corresponding Recombination Signal Sequences (RSS). Genomic position of the loci is indicated in brackets. In *IG* loci each rectangle depicts one of the variable (*V*), diversity (*D*), joining (*J*) and constant (*C*) genes, and circles (assigned with “s”) depict switch regions. The number of known functional genes, as listed in the IMGT, is indicated underneath each scheme. RSS structure schemes depict the position of heptamers (7), nonamers (9) and spacers (12/23) relative to *V*, *D* and *J* genes. **C** Hematopoietic stem cells in bone marrow, give rise to cells of both myeloid and lymphoid origin. While most of the cell types retain *IG* genes in their germline configuration, precursor B cells rearrange first Ig heavy chain and then Ig light chain genes to form a functional antibody. B cells with the functional B-cell receptor migrate to the periphery where they can recognize antigen. Upon antigen recognition and receiving help from T cells, B cells enter germinal center reaction during which they undergo intensive proliferation, improve affinity for antigen by the introduction of somatic hypermutations (SHM) in rearranged *IG* genes, and may change their effector functions in the process of class-switch recombination (CSR). This results in the formation of memory B cells and antibody-secreting plasma cells. *IG* genes can be sequenced from any B-cell type. However, in (virtually) all cells other than B cells, they will be in their germline configuration. Precursor B cells and naive mature B cells carry rearranged *IG* genes, which can be further modified by the presence of SHM in post-germinal center cells. Light blue block underneath B-cell maturation scheme depicts the sources of *IG* sequencing in the three existing *IG* databases: IMGT (ImMunoGeneTics, http://www.imgt.org/), IgPdb (http://cgi.cse.unsw.edu.au/~ihmmune/IgPdb), VBASE2 (http://www.vbase2.org), and the *IG* gene sequence data from the 1000 genome project (G1K-Ig-db, http://www.internationalgenome.org). VBASE2 has classified the alleles in different classes based on their genomic and rearrangement evidence. Class-1 alleles in VBASE2 have evidence from both; Class-2 and Class-3 alleles of VBASE2 are supported either by germline occurrence or rearranged repertoires.

Population genomics has revolutionized the field of personalized medicine and plays a significant role to improve clinical patient care [1]. These genomic studies have provided a better understanding of the population demography of human evolution, migration and diseases [2–4]. In the same view, the genetic variants in the immunoglobulin (*IG*) loci likely also play a role in vaccination efficacy and disease association [5–8]. The complex mechanism of antibody production from *IG* genes is a key to the development of the broad repertoire of the antigen-specific B-cell receptors of the adaptive immune system [9–13]. These Ig proteins (antibodies) are assembled in B cells from two pairs of polypeptide chains, the Ig heavy (IgH) and Ig light (Igκ or Igλ) chains that are encoded by different combinations of genes present in the *IG* loci, termed variable (*V*), diversity (*D*), joining (*J*) and constant (*C*) (Fig. 1A). The *IG* heavy chain locus (*IGH*) on chromosome 14q32.3 consists of multiple different functional genes: ~44 *V*, ~27 *D*, ~6 *J* and ~9 *C* genes (Fig. 1B). During recombination, one of each *V*, *D* and *J* genes recombine to a *V*-*D*-*J* exon to code for the antigen-binding domain of the IgH chain (Fig. 1A). The *V(D)J* recombination process is guided via short highly-conserved DNA stretches, called “recombination signal sequences” (RSS), present at each recombination site of the *IG* gene segments, i.e. downstream to *V*, upstream to *J*, and at both sites of *D* (RSS panel in Fig. 1B) and that govern the usage and hence the selection of the accounted genes [14–17]. The rearrangements in both *IG* light chain loci (kappa: *IGK*, on chromosome 2p11.2; lambda: *IGL*, on chromosome 22q11.2) take place in an analogous way with direct rearrangement between *V* and *J* genes, as *D* genes are absent. This process itself can produce up to three million different antibodies [15]. Additionally, most of the *IG* genes harbor inter-individual germline allelic variants, which can be shared between or be specific to human populations [18–20]. Consequently, different individuals can produce different antibodies (derived from different allelic variants), implying that at the population level the diversity of antibodies is even more extensive than at the individual level [5, 7, 21, 22].

The B-cell receptor (BCR) remains unchanged during the developmental stages of B cells from bone marrow (BM) to naive mature B cells (Fig. 1C). However, upon antigen recognition, generally taking place in germinal centers (GC) during interaction with T cells, B cells proliferate and modify the antigen-binding domain of their BCR via somatic hypermutation (SHM), randomly occurring in the *V(D)J* exon region. B cells with SHM that induce better antigen-binding of their antibodies will be positively selected and contribute to improved B-cell responses such as in vaccination. Therefore, the variants in the genes and RSS regions in the *IG* loci can govern the diversity, selection and expansion of B cells and their antibodies in individuals which subsequently are related to the population-specific immune responses in vaccination and diseases.

Several *IG* databases, such as IMGT [23–28], IgPdb (http://cgi.cse.unsw.edu.au/∼ihmmune/*IgPdb*/), and VBASE2 [29], report germline variations, present in different individuals. The sources of these three databases differ and each of them may comprise specific biases depending on the origin of the included *IG* sequences [30]. In Fig. 1C, the sources of the three different databases are aligned with the B-cell differentiation and maturation pathway. IMGT is the most widely used database, because of its early availability, longstanding experience and the most complete structure, but is at least in part derived from mature post-GC B cells [26, 27]. IgPdb does not comprise complete *IG* gene sequences. VBASE2 has incorporated strategies for identification of true germline alleles, by drawing them from genome databases, namely the EMBL nucleotide sequence database, Ensembl, and supported by evidence from the rearranged repertoires, but all the alleles are partial in sequence. So far, the *IG* databases lack documentation on the B-cell source, the ethnic origin and information on the frequency of the most reported genes/alleles [19, 30, 31].

Completeness and accuracy of germline sequences will influence downstream analyses in repertoire sequencing (Rep-Seq), as unreported *IG* allelic variants can appear as recurrent SHMs and skew estimated segment distributions and/or estimated mutation frequencies in clinically relevant settings, e.g. antibody responses in infection and in vaccination studies [30, 32–34]. Accordingly, the open germline receptor database (OGRDB) aims to stringently assess and classify germline alleles [35, 36]. OGRDB’s endeavor does not include *IG* alleles called from high-throughput whole genome mapping studies, as OGRDB believes that the inferred sequences may contain false positives [36]. Nevertheless, whole genome sequencing provides a unique opportunity to profile all allelic variants in single individuals with the possibility to investigate the specificity of such allelic variants to different human populations.

Likewise, the “1000 Genomes (G1K)” dataset, derived from cell samples of 2504 individuals, has been used to call alleles [20]. Yu et al. developed a method named AlleleMiner to determine alleles for the *IG* and *TR* loci from the G1K data creating the Lym1k database. Unfortunately, they did not provide information on the reliability of the newly identified alleles. Moreover, they did not retain the accompanying population information. Yu et al. also did not include all relevant components of each *IG* locus namely *D*, *J*, *C* genes and RSS, which is a strong feature of the IMGT database. Consequently, we set out to obtain an accurate set of alleles with enriched information. We have profiled all alleles for the *V*, *D*, *J*, and *C* regions as well as the RSS regions for all three *IG* loci from the G1K whole-genome sequencing (WGS) data. Moreover, we assessed the reliability and population information of each allele meticulously.

Using the G1K resource or other short read data to profile alleles for the *IG* loci raises potential pitfalls [6, 37, 38]. The repetitive and complex nature of *IG* loci makes it difficult to identify germline alleles from short read sequencing data. To deal with alleles resulting from these events, we have grouped these duplicated genes into so-called operationally indistinguishable (OI) genes and filtered them manually. Another complexity with analyzing the G1K data lies in the sample origin from the Epstein–Barr virus (EBV) transfected B-cell lines (Fig. 1C). As ~75% of the genomes (1941 genomes) in G1K are derived from EBV-transformed B-cell cultures, we defined a set of rules to obtain high-fidelity alleles (detailed in Methods) preventing them from being the result of sequencing errors or SHM. Consequently, the compiled resource comprises of *IG* germline allelic variants that cover a wide range of ethnicities in five superpopulations, namely, Africans (AFR), Americans (AMR), East Asians (EAS), Europeans (EUR) and South Asians (SAS). We made the combined data set of germline *IG* genes available through a database called “population matched *IG*” (pmIG) database. pmIG database, in conjunction with the classical databases, can support the understanding of the antigen-specific B-cell repertoire and the affinity-maturation related selection processes in health and disease.

## Results

The 1000 Genomes (G1K) database from 2504 individuals is a resource covering 26 populations representing five continents (Table S1). The identified population-specific alleles in all three *IG* loci (*IGH*, *IGK* and *IGL*), are grouped into three allele sets (AS1, AS2, or AS3) based on different confidence levels (see Methods) (Fig. S1). The pmIG database further contains meta information about the alleles such as the support of haplotypes for each (sub)population (Tables S2–S4).

### The alleles from G1K samples are not affected by SHM from EBV transfected cell-lines

The G1K samples originate from different sources, including EBV-transfected B-cell cultures and blood as well as an unreported source in a few cases. SHMs might be present in mature B cells immortalized by EBV transfection. It should be noted that such EBV-transfected B cells are mainly polyclonal, unless cultured for (very) long time (many months or more) or single cell subcloned; this has not been the case for the G1K samples. Polyclonal B-cell cultures will not likely have dominant SHM-based nucleotide variants detectable [39]. Nevertheless, we tested whether more allelic variants are found in samples stemming from an EBV origin. The metadata of the G1K samples report EBV coverage which was obtained by mapping the sequencing reads to the EBV genome. Using these annotations, we divided the samples into two groups: (1) Set-Blood, the non-EBV samples (563 samples), comprising of samples derived from blood (i.e. samples with EBV coverage <20X) and samples where the source was not reported; and (2) Set-EBV, the EBV samples, containing the remaining samples (1941 samples). Of 410 different *IGHV* alleles, 186 (45%) alleles are supported by samples in Set-Blood (Fig. S2A). From the 410 *IGHV* alleles, 145 (35%) are known to existing databases (AS1 category, Methods), and 103 (71%) of those are supported by Set-Blood samples. There are 196 (47%) frequent novel alleles (AS2 category, Methods) from which 77 (39%) are also supported by Set-Blood and 119 (61%) by Set-EBV. Furthermore, Set-Blood covered majority of the known alleles that are frequently present in the human populations (Fig. S2B) which suggests that rare alleles are not supported by Set-Blood samples. These observations on the novel alleles convince us that the alleles that we detected in the G1K samples are not influenced by SHMs related to EBV-transfection of mature (post-GC) B cells, likely because we used the strict requirement of at least seven haplotypes for calling an allele. Especially because relative frequencies of alleles in Set-Blood and Set-EBV comply with the distribution of the number of samples between the two sets, as Set-Blood has about 3 times fewer samples than Set-EBV.

### Conserved mutation pattern of pmIG alleles differs from SHM-affected *IGH* sequences

GC reactions result in affinity maturation of the Ig molecules, based on SHM and subsequent selection processes [40]. To further substantiate that the alleles identified from the G1K resource with our stringent filtering criteria are correct, we aligned the alleles from all the resources with the *IG* sequences obtained after Hepatitis B (HepB) vaccination in naive individuals (Methods section). Figure S2C shows the mutating positions (mutated positions in red) for three genes *IGHV1-69, IGHV3-15* and *IGHV3-30* each being a representative example of duplicated genes, self-evident (SE) genes and operationally indistinguishable (OI) genes, respectively. Interestingly, the mutations in the pmIG alleles for these genes are not random and thus follow the above described strictly conserved pattern. From this, we conclude that the novel alleles that we identify in our database, pmIG, are free from SHMs and mostly represent combinations of already known mutating positions with only a few new mutations, if at all (see also Fig. S3).

### Most known alleles are frequent and present in all ethnicities

The alleles that map to the IMGT database as well as to the other databases were instrumental in identifying two groups of alleles, i.e. known and novel alleles (see Methods section). We found that 35% of the *IGHV* alleles mapped to the known alleles (Table 1) with 60% of them present in all the superpopulations with support of at least 100 haplotypes (Fig. S4A). Most of these alleles are shared with the IMGT database, indicating that the IMGT database contains universal alleles. This was similar for the *IGKV* (34%) and *IGLV* (31%) alleles.

**Table 1:** Number of alleles in different functional gene segments in *IG* loci.

	AS1				AS2				AS3							
	Sum	SE	GA	OI	Sum	SE	GA	OI	Sum	SE	GA	OI	TOTAL	IMGT	IgPdb	VBASE2
***IGHV***	145	95	31	19	196	91	47	58	68	25	18	25	409	236	196	272
***IGHD***	29	29	0	0	8	8	0	0	5	5	0	0	42	33	2	0
***IGHJ***	5	5	0	0	4	4	0	0	0	0	0	0	9	12	1	0
***IGHC***	16^a^	5	11	0	71	24	47	0	44	16	28	0	125	49	0	0
***IGKV***	83	10	73	0	70	5	65	0	26	3	23	0	179	80	23	168
***IGKJ***	7	7	0	0	2	2	0	0	0	0	0	0	9	9	0	0
***IGKC***	2	2	0	0	0	0	0	0	1	1	0	0	3	5	0	0
***IGLV***	84	56	0	28	81	59	0	23	34	26	0	8	200	74	9	136
***IGLJ***	10	10	0	0	11	11	0	0	6	6	0	0	27	10	0	0
***IGLC***	5	5	0	0	16	16	0	0	8	8	0	0	29	14	0	0

^a^5 AS1 alleles were identified as false positives, based on mapping to the paralogs in *IGHG* alleles.

AS1 (Known), AS2 (Frequent) and AS3 (Rare) are major confidence levels. AS2 and AS3 alleles are further subdivided into SE (self-evident genes alleles), GA (group gene alleles) and OI (operationally indistinguishable gene alleles) categories.

Out of 29 African *IGHV* alleles that mapped to known alleles (Figs. S4B, 4), 17% (5) map to IMGT and the majority of them (60%;16) map to IgPdb with a minimum haplotype support of 5 (Fig. S4B). The lower haplotype support of the alleles mapped to IgPdb databases suggests that the IgPdb includes rare alleles, whereas IMGT is comprised of frequent alleles. Similarly, known African alleles in *IGK* and *IGL* loci had a larger overlap with IgPdb and VBASE2 than with IMGT, suggesting that alleles private to specific populations are not present in IMGT. IgPdb and VBASE2, however, do not capture the complete diversity of African ethnicity as they contain ~20% of the total African frequent *IGHV* alleles found from ~600 African individuals in G1K. *IGHV* alleles private to Asian populations are mostly absent in the three existing databases. *IGKV* and *IGLV* alleles private to any population including Africans are not profiled in any of the current resources (Figs. S5 and 3). These findings suggest a biased sampling by the current databases.

### Conserved mutation patterns in the filtered alleles as compared to the existing databases

Even after stringent filtering, “novel alleles” might suffer from SHM or sequencing errors [38]. As SHMs are introduced randomly and do not have a fixed pattern, we have already eliminated possible false positives by putting a threshold of seven haplotypes (= at least four individuals). Furthermore, 118 potential false-positive alleles were eliminated that appeared due to the lack of support in other databases or the same mutating patterns between the members of group and OI genes (Table S5). Moreover, when we performed the alignments of the novel alleles with the known alleles present in the pmIG, IMGT, IgPdb and VBASE2 databases, it appeared that the novel alleles revealed highly conserved mutation patterns at fixed nucleotide positions, the same positions as found in the other databases (Fig. S3). The most spectacular examples from the *IGH* locus are that the novel alleles of the *IGHV2-5*, *3-15*, *3-20*, *3-49*, *3-64*, *3-72*, *3-74*, *4-39*, *6-1* genes did not gain any new polymorphisms, suggesting that evolutionary pressure and selection play an important role for the remaining locations in these genes (Fig. S3). A comparable observation was made for the *IGHV1-69* gene, where only one new mutation was found in the CDR1 region, which was specific to Asian populations (1 allele supported by 10 haplotypes) (Fig. S3, Table S2). The identification of these specific mutation patterns provides extra evidence that the identified variant nucleotides are not sequencing errors, but genuine allelic variants.

On the contrary, we found that several alleles for the *IGKV* genes (*IGKV1-5, 1-33, 1-39, 3-11, 3-15, 3D-20*) in the IgPdb database suffer from possible SHMs, sequencing and/or sequencing mishandling errors, as several new mutations across the reported alleles are concentrated towards the 3' end of the *V* gene (near the CDR3 region) (Fig. S3). Also, Wang et al. [30] concluded that many IMGT alleles are in fact not genuine germline sequences (Fig. S3, alleles with yellow background). We did not find these alleles back in our pmIG database.

Based on the comparisons of different resources we realized that each *IG* germline database has certain unique features as well as disadvantages (Table 2). Our database does not contain alleles from the genes that are duplicated, as they are not present in human chromosome GRCh37 assembly. Elegantly, the IMGT database has profiled all such genes to completeness.

**Table 2:** Comparison of key features in different databases that profile *IG* germline alleles.

	IMGT	VBASE2	IgPdb	pmIG
Ethnic origin	None	None	None	Available
Allelic frequency per ethnic group/population	None	None	None	Available
Cell-source for alleles	Unclear	Unclear	Unclear	Available
All germline genes from single individuals	No	No	No	Yes (from all 2504 individuals)
Completeness of *IGHV* alleles	138 Complete and 98 Partial	*V* region only	V region only	All complete (including leader)
Completeness of *IGKV* alleles	71 Complete and 9 Partial	*V* region only	V region only	All complete (including leader)
Completeness of *IGLV* alleles	39 Complete and 35 Partial	*V* region only	V region only	All complete (including leader)
Presence of *IGHD* and *IGHJ* genes	Available	None	A few	Available
Presence of *IGKJ* genes	Available	None	A few	Available
Presence of *IGLJ* genes	Available	None	A few	Available
Presence of *IGHC* genes	Available	None	None	Available
Presence of *IGKC* gene	Available	None	None	Available
Presence of *IGLC* genes	Available	None	None	Available
RSS	Available	None	None	Available
Completeness in terms of alternate duplicated genes	Complete	No (Not all genes present)	No (Not all genes present)	Complete as per availability from GRCh37
Strategy for exclusion of SHM	Not reported	Not reported	Not reported	Explicitly reported with avoidance of SHM by strict rules
Confidence levels	NONE	Class-1, 2, 3	None	Double layer of confidence levels (Confidence levels with information on group genes and OI genes)

### Novel alleles are “maximally two mutations away” from the known alleles

Frequently, new alleles were either a combination of known polymorphisms or gained polymorphisms. Many of the newly detected *IGHV* alleles (AS2/3 category) have no new mutations or only one new previously unreported mutation (Table 3), or in other words, the 134 novel *IGHV* alleles each contain a new unique combination of already observed (individual) mutations. Seven *IGHV* alleles gained more than three new mutations which belonged to the lower confidence category AS3. These observations were corroborated in the IgPdb and VBASE2 database, where novel alleles indeed also had one to ten new mutations as compared to the IMGT alleles (note that frequency of alleles is not reported in both databases) (Fig. S3).

**Table 3:** Number of alleles with count of new mutating positions as compared to the existing databases.

	*IGHV*		*IGKV*		*IGLV*	
Mutation counts	Complete sequence	*V* region only	Complete sequence	*V* region only	Complete sequence	*V* region only
Zero	279	312	85	95	68	91
One	102	72	85	78	102	93
Two	21	19	7	4	26	14
Three	5	5	1	1	3	2
Four	1	1	1	1	1	0
Five	1	1	0	0	0	0

Complete sequence includes leader sequence and the *V* region for all the *V* genes. This is important to realize that 98 *IGHV*, 9 *IGKV* and 35 *IGLV* alleles are partial and do not contain the leader sequence.

The *IGKV* and *IGLV* alleles did show a slightly different pattern as in *IGHV* alleles, i.e. the 85 novel *IGKV* and 102 novel *IGLV* alleles all have at least one new mutation as compared to the ones in known alleles (Table 3). However, the position of these new mutations is important, i.e. they can occur in the *V* region or in the leader region (16% *IGHV*, 18% *IGKV* and 25% *IGLV* of total mutating positions). Most of the light chain alleles (9 *IGKV* and 35 *IGLV* alleles) in the current databases are only partial i.e. they do not comprise of leader region. We believe this to be a major reason why for the *IGKV* and *IGLV* novel alleles we observe new mutations instead of a novel combination of existing mutations.

### Long read sequencing of an individual from “1000 Genomes” suggests completeness of the pmIG resource

Recently, the NA12878 sample from 1000 Genomes was sequenced using long read sequencing technology [41]. The study identified four novel alleles and 66 IMGT alleles from the individual. Although that study mapped the long sequencing reads to the GRCh38 human reference genome as compared to the shorter sequencing reads in 1000 Genomes data, we have obtained comparable result. We recovered all the novel alleles identified in that study and 61 of 66 known IMGT alleles were also identified in the pmIG resource. Of 61 matched alleles, the *V* region of four IMGT alleles: IGHV3-35*01, IGHV3-23*01, IGHV1-18*01 and IGHV3-11*01 matched completely with the alleles in the pmIG resource i.e. the changes were recorded in the Leader sequence. 95% IMGT alleles were recovered in the pmIG resource and 100% novel alleles were recovered for this individual. Three of four novel alleles reported in the study were already present in the IgPdb database. The 5% false negative rate for this sample (unidentified IGHV4-4*02, IGHV3-66*01, IGHV1-69*04, IGHV2-70*01 and IGHV2-70*15) can be explained by the differences in the human reference genome assembly used for mapping and calling alleles.

### Population Distribution of the *IGH* alleles

**Fig. 2:**
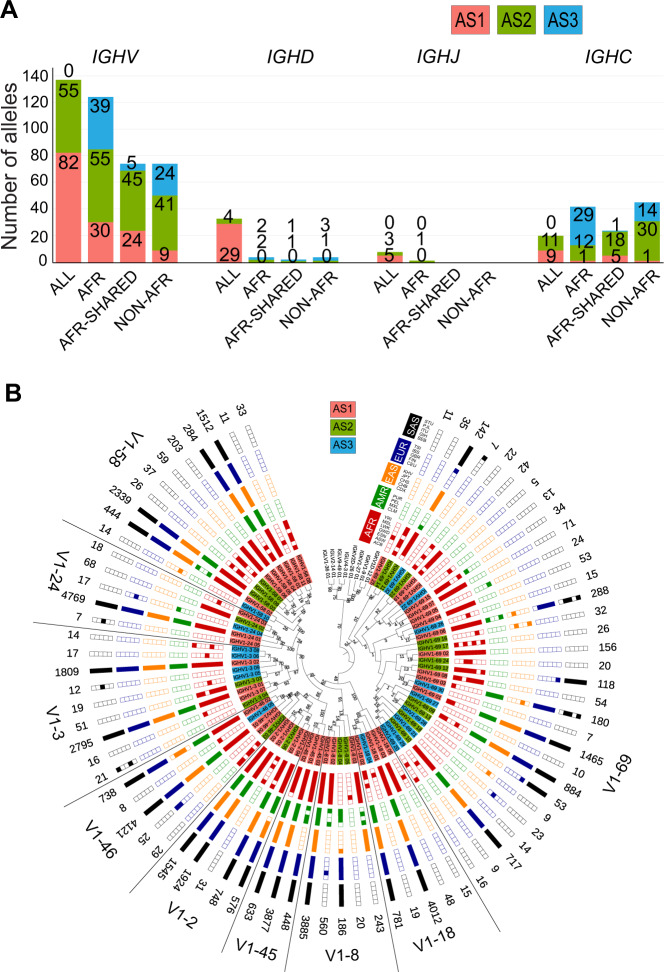
Population distribution of alleles in *IGH* heavy chain locus. **A** The superpopulation distribution of *VDJ* genes and *C* genes for *IGH* locus. The alleles are categorized based on the populations i.e. “ALL” represents alleles present in all the superpopulations, “AFR” category comprises of alleles present only in African populations, “AFR Shared” comprises of the alleles present in African populations and shared with one of the other superpopulations and “Non-AFR” where alleles are present in at least one of the populations other than Africans. **B** Maximum Likelihood tree of the population distribution of *IGHV1* family alleles. The *IGHV1* family genes are indicated in the legends. Red label background indicates AS1 alleles, green AS2 and blue AS3 alleles. The population distribution is plotted in a binary format where each block is a population. Filled block represents the presence of that allele in at least four haplotypes in that population, otherwise the block is unfilled. For the population distribution of other *IGHV* families refer to Figs. S3 and S4. A few *IGKV* and *IGLV* alleles were used as outgroups.

The first allele of each *IGHV* gene (_01) in pmIG database, sorted such that it is supported by the maximum number of haplotypes, is known and present across all superpopulations (denoted as “ALL”). Approximately 100 new *IGHV* alleles (frequent or rare) are unique to the African populations (“AFR”) while only 29 African *IGHV* alleles are known (Fig. 2A). Alleles not observed in Africa (“Non-AFR”) or observed in Africa and other superpopulations but not all (“AFR Shared”) occur less (~40% less). Together, this suggests that Africans have a considerable diversity that has not been captured so far.

*IGHV1-69* (Fig. 2B), *IGHV2-70*, *IGHV3-53* and *IGHV3-23* (Fig. S6) genes have the highest allelic diversity in the African superpopulation. *IGHV3-48* alleles are highly diverse in South Asians (Fig. S6), whereas new alleles in the *IGHV4* family are highly diverse in African and East Asian populations (Fig. S7). Similarly, *IGHV7-81* alleles mostly belong to the African and American superpopulations (Fig. S7). These differential diversities suggest an environmental adaptability or population drift of these *IGHV* genes.

Most of the *IGHD* alleles are present in all populations and only a few are private to (super-)populations. Even rare variants are shared among different ethnicities, which hints that the *IGHD* genes are evolutionary conserved across populations. Also, all *IGHJ* alleles are shared between all the ethnicities (Fig. 2A).

The constant genes of the *IG* loci are responsible for the effector functions of the antibodies and have been considered to be more conserved as compared to the *V*, *D*, *J* genes. In contrast, we found 196 alleles for the nine *IGHC* genes. As *IGHA1,2* and *IGHG1,2,3,4* genes are highly conserved, also visible from the alignment of the alleles within these groups based on their CH1-3 domains (Fig. S8), we grouped the alleles within these two groups (group genes, Methods). This resulted in only 125 alleles being retained, where the majority of the alleles that were filtered out are from the *IGHA1*, *IGHG1*, *IGHG3* and *IGHG4* genes (Tables S2, S5).

To further understand the contrast between the supposedly conserved constant genes and the many alleles found, we converted the nucleotide sequences of the *IGHC* alleles into protein sequences and mapped them to the known allotype sequences. This shows that multiple mutations in the allelic sequences for *IGHC* genes are synonymous and, therefore, the diversity at the protein level is quite low as compared to the nucleotide level (Table S2, Fig. S9). This suggests a high evolutionary pressure on the *IGH* constant genes to conserve the structural and functional properties of the Ig proteins.

Three new allotypes were identified for the IgA1, IgE and IgD proteins, respectively, all specific to African populations (Fig. S9). The amino acid change in the IgG1 allotypes did not result in a change of either structural properties (aliphatic ↔ aromatic) or the charge (neutral ↔ negative ↔ positive).

### Population distribution of the *IGK* alleles

Seventy-six *IGKV* alleles are present in all superpopulations, of which 62 alleles are already known (Fig. S5A). Only 10 of the 41 African alleles map to known alleles. A large number of alleles is observed outside Africa (“Non-AFR”), suggesting that the diversity in the *IGK* locus does not only prevail in the African superpopulation (Figs. S5B and S10). All *IGKJ* alleles are shared between ethnicities except one allele that was unique to Africans. Of the two major *IGKC* alleles, one is present in all superpopulations and one is unique to Africans.

### Population distribution of the *IGL* alleles

**Fig. 3:**
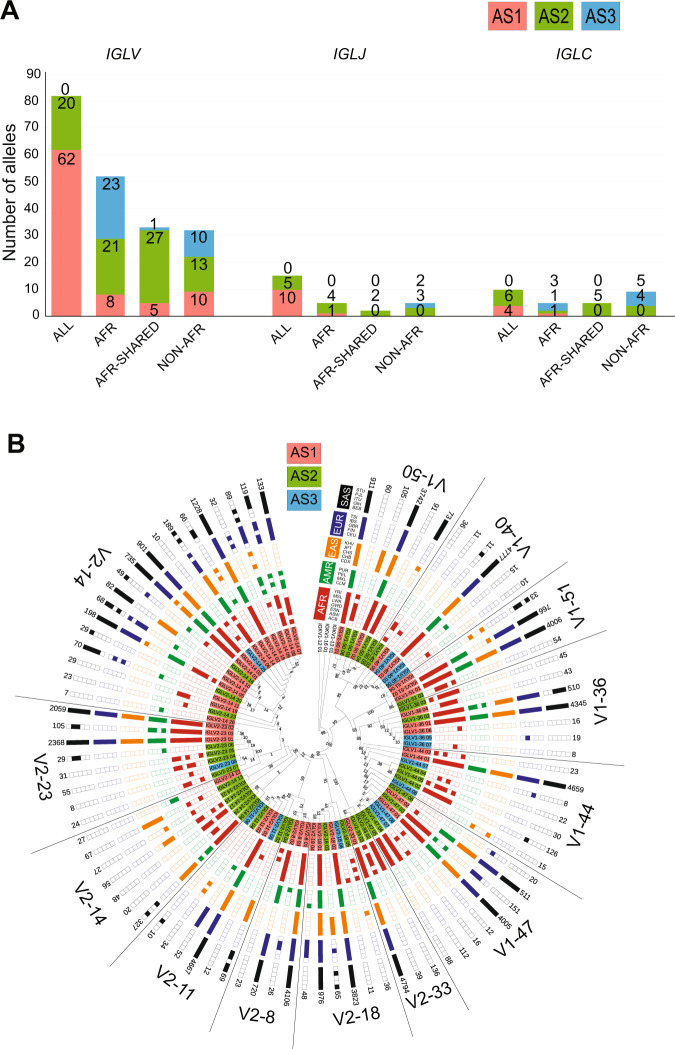
Population distribution of alleles in *IGL* light chain locus. **A** The superpopulation distribution of *VJ* genes and *C* genes for *IGL* locus. The alleles are categorized based on the populations i.e. “ALL” represents alleles present in all the superpopulations, “AFR” category comprises of alleles present only in African populations, “AFR Shared” comprises of the alleles present in African populations and are shared with one of the other superpopulations and Non-AFR where alleles are present in at least one of the populations other than Africans. **B** Maximum Likelihood tree of the population distribution of *IGLV1* and *V2* family alleles. For the population distribution of other *IGLV* families refer to Fig. S8. A few *IGKV* alleles were used as outgroups.

The population distribution of the *IGL* alleles is similar to that of the *IGH* alleles. Most of the known *IGLV* alleles are present in all superpopulations. The majority of the new alleles in both the frequent and rare alleles is unique to African populations (Fig. 3A). Similarly, known *IGLJ* alleles are present in all the superpopulations, whereas rare *IGLJ* alleles are either unique to African populations or are not observed in the African population (“Non-AFR”). The first *IGLC* alleles (_01) are present in all superpopulations, except *IGLC2_01* that is unique to Africans. New rare *IGLC* alleles belong to either the African populations or populations outside Africa (Non-AFR). Of the new frequent *IGLC* alleles, only a few are unique to European and South Asian populations, and the majority exists in African populations (Figs. 3B and S11).

### Recombinant signal sequence (RSS) variants in *IG* (pseudo)genes influence their recombination frequencies

RSS regulates the recombination process in which the conservation of RSS heptamers and nonamers plays a significant role [42–44]. We did not find variations in conserved heptamers and nonamers of allelic RSSs that may explain population-specific recombination frequency of the respective genes. Also the conservation of RSSs in *IGHV* genes was reported to be related to differences in recombination frequencies [15, 32]. We found that the heptamers and nonamers in all *IGHV* RSSs are conserved, except the ones related to *IGHV3-16* and *IGHV7-81*. Interestingly, a relatively lower recombination frequency of *IGHV7-81* has been reported before [32].

Several *IGHD* genes have mutated heptamer sequences at 3′D-RS and 5′D-RS (Fig. S12, Table S6), which might explain their reported reduced recombination frequencies in healthy individuals [32]. All *V* genes in *IGK* and *IGL* loci have conserved heptamers, except the *IGKV1D-13* and *IGKV2D-30* genes in the *IGK* locus (Table S7), and the *IGLV5-48*, *IGLV2-33*, *IGLV3-22*, *IGLV3-19* and *IGLV2-14* genes in the *IGL* locus (Table S8). These genes have mutations in the first three bases of their heptamers, which consequently should result in reduced recombination frequencies [42–44].

The RSS spacer length also plays a role in the recombination frequency [16, 45, 46]. We found that the spacer length in RSSs of most *IGHJ* genes is 22 bp, except for the *IGHJ3* and *IGHJ4* genes that have a spacer of 23 bp. In addition, *IGHJ4* has the most conserved heptamers followed by *IGHJ6* (Table S6). These observations could explain why the *IGHJ4* and *IGHJ6* genes have the maximum recombination frequency among *IGHJ* genes [47].

We also found conserved RSS heptamers adjacent to fifteen and eight pseudogenes in the heavy and light *IG* loci, respectively (Table S9). The location of the RSS in few of these pseudogenes is 10–30 bases more distant from the *V* pseudogene boundaries than for regular *IGHV* genes in which the RSS is generally 0-3 bases adjacent to the *V* gene boundary. Also, five *IGLV* functional genes had RSS sequence 10–25 bases downstream to the gene (Table S8). We found that only *IGLV3-7* (pseudogene*)* has a stop codon in between RSS heptamer and *V* gene boundary which could impact the recombination frequency of this gene. The impact of distance between RSS and gene boundaries is not yet known.

### Relation of *IG* alleles to variable immune responses in populations

The efficiency of the antibody response in different populations can be driven by the germline allelic variants. Therefore, we set out to understand the diversity in immune responses to infections or diseases by investigating the allele distribution in the *IG* loci. To do so, we annotated alleles with their impact on human health based on their polymorphisms and whether they contain at least one known disease-associated variant (based on a literature search with keyword “*IG* gene name + disease/vaccine”). Here, we report four examples of the disease alleles that show different frequencies across the different (super-)populations.

#### EXAMPLE-1

**Fig. 4:**
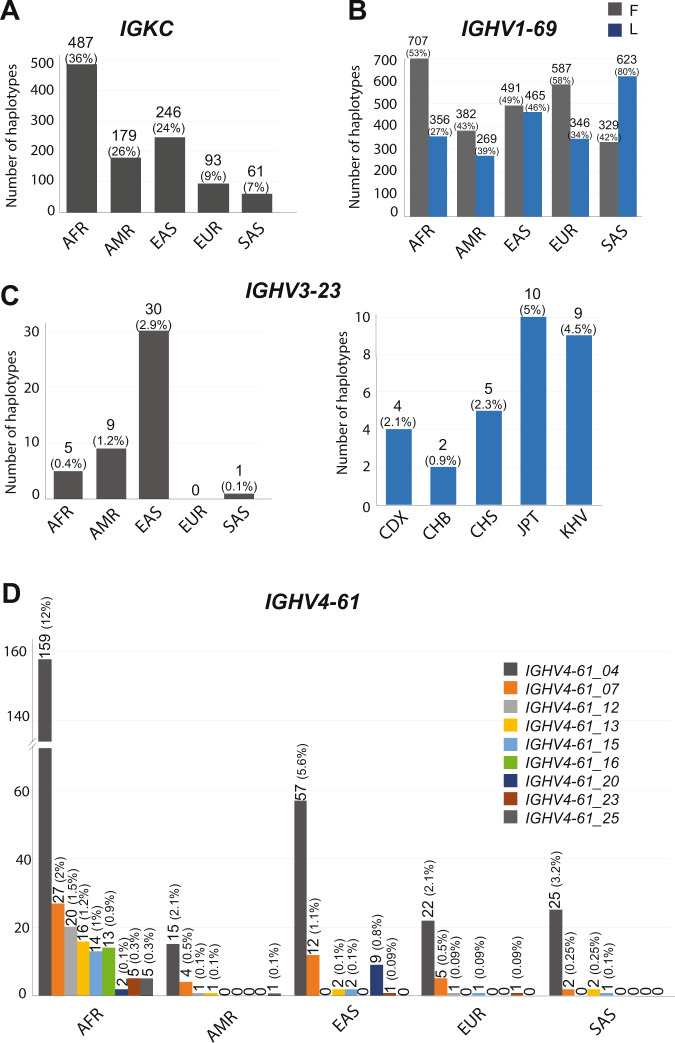
Frequency of *IG* alleles associated with disease immune responses in populations. **A** *IGKC:* One allele supported by 1066 haplotypes is present in all the superpopulations. **B** *IGHV1-69*: All the alleles were divided into two groups i.e. one with F “Phe” in CDR2 region and other with “L”. The combined distribution of the two groups is represented in the figure. **C** *IGHV3-23***:** One allele is supported by 45 haplotypes. The plot on the left represents the superpopulation distribution of the allele. The right plot represents the population distribution of the allele in the East Asian population. CDX: Chinese Dai in Xishuangbanna, China; CHB: Han Chinese in Beijing, China; CHS: Han Chinese South; JPT: Japanese in Tokyo, Japan; KHV: Kinh in Ho Chi Minh City, Vietnam. **D** *IGHV4-61***:** Nine alleles i.e. *IGHV4-61_04, IGHV4-61_07, IGHV4-61_12, IGHV4-61_13, IGHV4-61_15, IGHV4-61_16, IGHV4-61_20, IGHV4-61_23* and *IGHV4-61_25* were found to have the mutations in combination with other mutations. Separate bar plots are drawn for each allele.

The *IGKC* gene mutation rs232230 (C- > G) (IGKC*04 in the IMGT database) results in a nonsynonymous variant (V- > L) that is a risk factor in *Helicobacter pylori* infection in gastric cancer and age in breast cancer (odds ratio 1.64 and 1.94, respectively) [48]. We found this allele to be present in 1066 Haplotypes of the G1K samples (Fig. 4A). The distribution of the alleles in different populations was not known before, but we found the allele to be evenly distributed across all populations with a median of 38 (Min: 7 – Max: 90) haplotypes (Table S3).

#### EXAMPLE-2

The F- > L polymorphism at amino-acid position 62 in the CDR2 region of *IGHV1-69* gene is known to have a potential role in modulating the anti-influenza antibody repertoire [5]. We not only detected a high diversity of the *IGHV1-69* gene in the African (super-)populations that bear the amino-acid “Phe: F” polymorphism (10 AFR alleles with F variant, whereas only 5 with L variant), but also that the F variant is overrepresented in African (super-)populations (Fig. 4B, gray bars). For Non-African populations, we found that F and L amino-acid mutations occur in equal ratios, except for South Asians where most alleles have the “Leu: L” polymorphism (Fig. 4B, blue bars). These findings are concordant with those of Avnir et al. [5]. The understanding of the population distribution of this polymorphism in *IGHV1-69* gene and its role in flu, can have an immediate implication in the implementation of “influenza” vaccines in different superpopulations. Because relative frequencies of these alleles are very different in different populations, therefore, we might conclude that the antigenic pressure in different continents is different and thereby has selected for different repertoires.

#### EXAMPLE-3

The IGHV3-23*03 IMGT allele is known to be fourfold more effective than IGHV3-23*01 against *Haemophilus influenza* type b (Hib) [7]. Recent studies suggest that meningitis caused by Hib is a common and serious disease in children in China [22, 49]. We observed that the IGHV3-23*03 allele is very rare and is present frequently only in the East Asian superpopulations. Only 30 haplotypes support this allele in the East Asian (super-)populations (CDX:4, CHB:2, CHS:5, JPT:10, KHV:9) (30 haplotypes) (Fig. 4C).

#### EXAMPLE-4

The IGHV4-61*02 IMGT allele is related to higher risk of rheumatic heart disease (RHD) in Oceanic populations where four polymorphisms (rs201076896, rs201691548, rs200931578, and rs202166511) increase the susceptibility [50]. In that study, the relationship was drawn only within the Oceanic populations. Therefore, we profiled the alleles carrying these four mutating positions in our pmIG resource. We found nine alleles comprising of these four mutating positions and their frequency was highest in African populations followed by Asian populations (Fig. 4D). This might suggest that RHD is more frequent in African populations as compared to Asian populations.

### Evolutionary dynamics of variation patterns in different populations

**Fig. 5:**
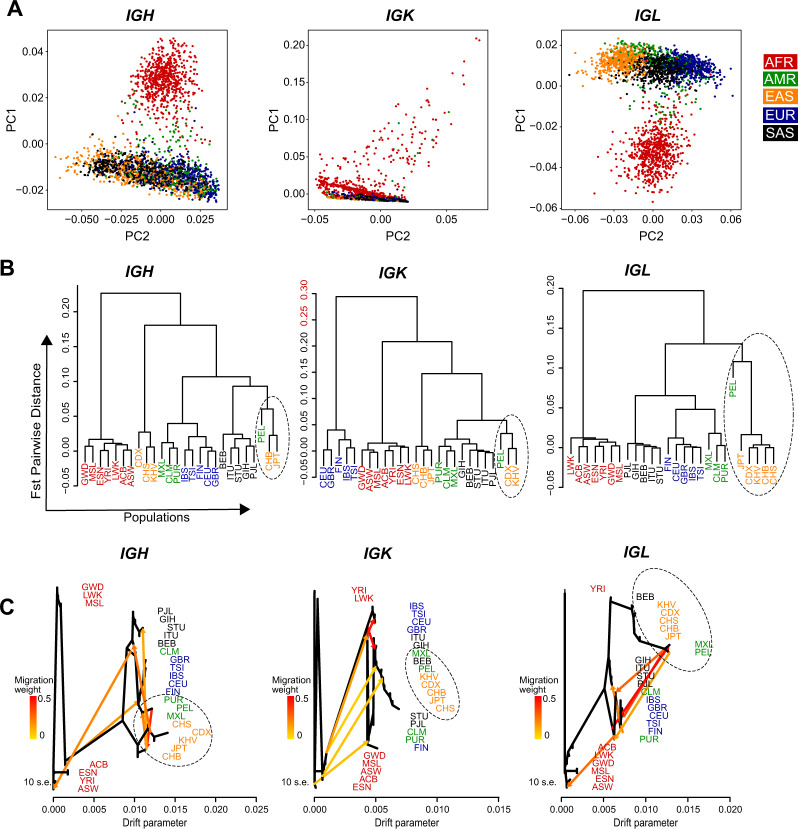
Genetic diversity, population structure and migration events in five superpopulations for *IG* loci. **A** Separate PCA plot of the Ig heavy and light chain genes based on the single-nucleotide polymorphisms in the complete locus. Each dot represents a sample and each sample is colored based on the superpopulation it belongs to. In *IGH* and *IGL* locus Africans have higher diversity as compared to Non-African superpopulations, while *IGK* has more diversity in part of the European population. **B** Pairwise population distribution calculated by Fst Matrix is represented as a cladogram for each locus namely *IGH*, *IGK* and *IGL*. 26 populations are colored as per the superpopulations i.e., Africans in red; Americans in green; East Asians in orange; Europeans in blue and South Asians in black. **C** Migration events in *IGH*, *IGK*, and *IGL* locus. Six migration events are marked in the ML tree where edge color represents the migration weight; red suggest higher migration weight and yellow the lowest.

The genetic diversity of individual genes does not reflect on the diversity of the complete *IG* loci including coding and non-coding regions. Therefore, we used the SNPs in the complete locus to identify the existing variations between (super-)populations. In Fig. 5A, we found African populations to be unique and highly diverse for *IGH* and *IGL* loci, whereas the *IGK* locus is much more condensed due to some large outlier samples in African and American populations.

Gleaning into the pairwise population differentiation (F_ST_) in the *IG* loci, we again observed a higher diversity in African populations in *IGH* and *IGL* loci (F_ST_ ~0.20; Fig. 5B). Interestingly, we found a different pattern in the *IGK* locus that has highest variability within Europeans (Fst ~0.30) rather than Africans (Fig. 5B). The lower diversity of the *IGK* locus in Africans is consistently visible in the population distribution of the alleles (Figs. 4 and S9). This might suggest that the variability in this locus is more recent in evolution, as compared to the *IGH* and *IGL*. Furthermore, the cladogram (Fig. 5B, marked by dotted circles) reveals a closer relationship between the Peruvian (PEL) population and East Asian populations, especially the Han Chinese in Beijing in China (CHB), and Japanese in Tokyo (JPT) in each *IG* locus, suggestive of a mixture between the Peruvian and East Asian populations.

To further substantiate the relationship between Peruvian population and East Asian populations, we performed the migration analysis on these 26 populations using Yorubian (YRI) African population as an outgroup. African populations formed the parental clades in the maximum likelihood tree (Fig. 5C). A migratory event, depicted by orange/red arrows, was observed in all the loci between African populations and other populations. In the *IGH* loci, we observed a cyclic connection (high migration weight depicted by red arrow connection) between Peruvian population and East Asian populations. Furthermore, we also observed strong migratory connections between East Asian and South Asian populations in the *IGH* loci. In the *IGK* loci, we observed migratory events between European and South Asian populations. We did not observe a migratory event between Peruvian and East Asian populations in the *IGK* loci, however these populations shared a clade which depicts a close relationship among Peruvian, East Asian and also South Asian populations (Fig. 5C, marked by dotted circle). Interestingly, in *IGL* locus, several migratory connections were observed between Mexican/Peruvian populations and South Asian and European populations. Similar to *IGK* locus, Mexican/Peruvian populations shared clade with East Asian population and BEB (Bengali in Bangladesh) South Asian population (Fig. 5C, marked by dotted circle). Similar to pairwise population differentiation, we observed a closer relationship between Peruvian and Asian populations, however, the migratory connections between different populations in each locus varied among all the populations.

## Discussion

We performed an extensive analysis of *IG* germline alleles from 2504 individuals, representing 26 populations, and created a population matched *IG* germline database (pmIG), that comprises a comprehensive overview of haplotypes across five main different ethnicities. We enriched the pmIG database by including information on frequent and rare germline alleles per population, facilitating identification of genuine germline alleles and excluding SHM. This will be important when studying differences in immune responses between ethnic populations as a consequence of germline differences [18, 51–56].

Similar to the Lym1K resource by Yu et al., we have used the “1000 Genomes” dataset to derive all *IG* alleles. But there are important differences. To avoid SHM-mutated alleles from EBV clones, we report information on haplotype support, including a minimal support of seven haplotypes (four haplotypes for known alleles), categorized them into confidence levels, scrutinized each allele manually for their use in repertoire studies, and also profiled population information for each allele. As a result, we report ten times less alleles than reported by Lym1K. It is fair to say that the “AlleleMiner tool” (used to create Lym1k) has a cutoff option for the minimal haplotype support. However, to identify potential sources of non-germline errors, the processes of B-cell development and SHM-based affinity maturation of antibodies should be understood carefully. For example, the existence of duplicated genes reduces the confidence in associated alleles. Therefore, we adopted a manual curation of alleles and assigned confidence levels to each allele.

The AS1 confidence level refers to alleles that are previously reported in other databases. Aligning such known alleles with tools like Muscle [57] is, however, not trivial, therefore a manual check was also performed to ensure accuracy. We found that the known alleles that we detect (and thus present in existing databases) are mainly present in the European-Caucasian populations, suggesting a population bias in the current IMGT, VBASE2, and IgPdb databases.

For the novel alleles we have also assessed the false-positive rate in our resource which is quite low, if not absent, albeit that we can never reach 100% accuracy. Still, we wish to emphasize that the accuracy of our novel alleles is supported by the highly conserved mutation patterns, restricted to a limited number of nucleotide positions, remarkably the same nucleotide positions as found in other *IG* databases. Also, a very small false-negative rate of 5% for the known alleles and 0% for the novel alleles was observed. We also found that the novel pmIG *IGHD3-10* and *IGHD3-16* alleles were profiled in a recent study [58]. Apart from profiling the alleles for genes, we could further identify conserved heptamer sequences adjacent to the genes and also pseudogenes. The pseudogenes are reported to be rearranged in several antibody repertoire studies [32, 59], which also relates to our unique above-mentioned finding. These findings suggest accuracy and completeness of the pmIG database.

We have used high stringency and manual curation to generate this resource, however, the inherent limitations of the short-read mapping and missing genes in the reference genome may persist. Such issues are majorly limited to the duplicated genes which are absent in the reference genome and also cannot be assembled or mapped accurately into individual genes i.e. *IGHV1-69*, *IGHV2-70* and *IGHV4-4* and henceforth into the allele calls. If indeed the sequence of the duplicated gene is different, we were able to identify such alleles and considered these alleles for future assessment. Just for reason of reliability, these alleles are not included in the database, but are listed with the lowest confidence in Table S5. The confidence level of such alleles in the pmIG database can be further increased by an additional support from Sanger sequencing of the alleles from independent sources.

Different germline *IG* alleles have shown to result in different responses and effectiveness against infections in individuals [5, 7, 60]. The population information of these *IG* alleles can provide better understanding of these differences in B-cell response at the population level e.g. as represented by IGHV3-23*03, *IGHV1-69* alleles. Together, these examples underpin the significant role of germline alleles in different populations and their protective nature against infection with the consequent potential impact of population-specific therapeutic antibodies. Detailed studies on differences in clinical disease course and final outcome in different regions of the world during pandemic outbreaks, such as the currently ongoing SARS-CoV2 pandemic, might at least in part show a role of the here-presented diversity of *IG* gene alleles within and between human populations.

The largest numbers of novel frequent and rare alleles were identified in African populations of which 70-90% of diversity is not captured by the existing databases. The high variability in the *IGH* and *IGL* loci in African populations, as compared to Non-African superpopulations, could indicate the migration of human populations out of Africa. On the other hand, the *IGK* locus shows higher variability in European populations, as compared to Non-African populations, which may be impacted by the duplicated *IGK* locus. Although we cannot follow the trend of mutations over time, the migration analysis and the allele statistics support the variability and environmental adaptation of this locus over time.

Several studies analyzing genome-wide patterns, genetic variations, demographic history and immune responses have also reported higher immune diversity in Africans [2–4, 53, 61–64]. The sampling of more individuals from multiple African populations can further unravel the genetic diversity in Africa and can thereby substantiate our understanding of allelic diversity in *IG* loci. As a future endeavor to obtain a more complete set of African germline *IG* alleles, we will evaluate the recently available 910 African genomes [4] and further increase the confidence in our novel African alleles.

We fully realize that the creation of an *IG* database is a complex endeavor. Till today, the IMGT database is the most used database in research because of its completeness. Also, the database hosts several tools to support researchers in analyzing and understanding next generation sequencing data of *IG* loci. The IMGT database includes 250 *V* genes, 35 *D* genes and 12 *J* genes for *IGH* locus wherein 50 *V*, 25 *D* and 6 *J* genes are present in each individual. Most of the *V* germline alleles are partial at 3′end that may contribute to the additional diversity and hence can impact the exploratory studies based on studying the immune diversity. The pmIG database addresses many problems faced by other databases and brings added value by enriching population-specific information as well as allelic frequencies to the meticulously identified germline *IG* alleles.

## Concluding remarks

With our pmIG database, we report identified and curated alleles across 5 superpopulations, containing 26 ethnic subpopulations, resulting in 170% more allelic variation. Having a richer source of *IG* alleles improves the interpretation of repertoire sequences. For example, determining whether an observed sequence belongs to a germline allele or is the result of SHM in response to an antigen. Or, whether measured sequences of naive B cells are considered to be the result of a sequencing error, which now is determined based on the presence of the observed allele in databases [65]. Alternatively, pmIG, in addition to the existing *IG* databases, can be exploited for applications in immune response dynamics analysis and clonal *IG* gene lineage analysis. Perhaps the most clinically relevant application of pmIG is understanding differences between populations and to support implementation of population-specific vaccination studies with analysis of the antigen-specific B-cell repertoire, i.e. from naive repertoire (without SHM) to primary response and booster response B-cell repertoires.

## Methods

### Data source

The “1000 Genomes” data (G1K) (May, 2013 release; http://www.1000genomes.org; GRCh37 assembly) in the form of phased variant call format (VCF) was used for this study. Phased variants for GRCh38 (a recent release for the “1000 Genomes”) are available, however, we used the GRCh37 version as the SNP IDs are not yet available for the GRCh38 mapping. These SNP IDs are relevant to perform mapping to other databases and assess false negatives. Both genome assemblies do not comprise of all the *V* genes mentioned in the IMGT database as multiple duplicated genes are not present in all individuals. The full release of the data set was collected from 2504 cell samples from diverse ethnic groups that have a uniform distribution of individuals across populations. The samples are classified in five superpopulations i.e. African, American, East Asian, European and South Asian, that are further subdivided into 26 populations (7 African, 4 American, 5 East Asian, 5 European and 5 South Asian populations) with a minimum of 61 and a maximum of 113 samples per population (Table S1). The VCF format of the data comprises information of both parental and maternal chromosomes for each sample.

### Identification of alleles from G1K data

The genes (*V*, *D*, *J* and *C*) from *IG* loci were retrieved from the VCF files for Chromosome 14 (*IGH*), Chromosome 2 (*IGK*), and Chromosome 22 (*IGL*). Only SNPs, deletions or insertions were processed from the VCF files. Copy number variations were not considered. Software, such as Plink (http://zzz.bwh.harvard.edu/plink/), to retrieve haplotypes from VCF files cannot process multi-allelic SNPs. Therefore, special Python scripts were written, while R scripting was used to obtain the 5,008 independent haplotypes from the 2504 cell samples (all available via GitHub). Identical haplotypes are merged, and the number of times a particular haplotype appears is counted and marked as an allele. The IMGT nomenclature is used to name genes, and alleles extend this name with a numbering, for example the 01 and 02 alleles of the *IGHV1-8* gene are referred to as *IGHV1-8_01*, *IGHV1-8_02*. IMGT alleles are denoted with an asterix, such as IGHV1-8*01, IGHV1-8*02. The alleles are sorted in descending order such that the first allele is supported by the maximum number of haplotypes.

### Terminology

With the term *“haplotype*” we refer to an operationally distinguishable gene (segment) present on one strand (inherited from a single parent) in one individual. There are two haplotypes, one on each positive and negative strand, with exactly the same or different polymorphisms. With *“allele”* we refer to the profile of variants across one haplotype. For example, *IGHV2-70_01* is an allele of gene *IGHV2-70*, which has been identified from 5008 haplotypes from 2504 individuals as a gene variant with maximum haplotype support.

*“Mutations”* are genetic mutations that occurred to form different alleles of the gene and “*Somatic Hypermutation Mutation (SHM)*” is the mutation that arises in *IGH/IGK/IGL* sequences in post-GC cells.

### Mapping the alleles to existing databases

The alleles obtained from the G1K samples are mapped to three different databases namely IMGT, IgPdb and VBASE2 [29] using Muscle [57] and manually checked to ensure accurate mapping. IMGT (www.imgt.org) is the global reference in immunogenetics and immunoinformatics studies and is maintained since 1989. IgPdb (http://cgi.cse.unsw.edu.au/~ihmmune/IgPdb) is a repository of suspected allelic variants of human *IG* germline genes. VBASE2 (http://www.vbase2.org) presents *V* gene sequences extracted from the EMBL nucleotide sequence database and Ensembl together with links to the respective source sequences. VBASE2 classifies the *V* genes into three different classes: Class-1, genomic and rearranged evidence; Class-2, genomic evidence only; and Class-3, rearranged evidence only [29]. This evidence classification of alleles was only performed by VBASE2 database; none of the other databases had such feature, implying that the capability for rearrangement is formally not included in the IMGT and IgPdb databases. At this point, we would like to emphasize that the mapping between databases is done at the full *V* gene length including the leader sequence. Any mutation in the leader region is also considered as a different allele. We on purpose included the leader sequence in the evaluation process, because the leader sequence guides the usage of the *V* exon and thereby directly influences the composition of the repertoire [66].

### Classifying alleles into confidence levels

The alleles in our study were classified into three major confidence levels (allele set (AS) 1–3) as described below:

#### AS1 (known)

G1K alleles with a minimum support of four haplotypes and identified in either the IMGT, IgPdb and/or VBASE2 (Class-1) databases. This AS1 allele set obviously has the highest level of confidence as the alleles are observed in the G1K resource as well as in at least one of the three existing databases. This set of alleles also validates G1K as a solid resource since there is substantial overlap with the existing databases.

The alleles that did not classify as AS1 were divided into two categories.

##### AS2 (frequent new alleles)

G1K alleles with a minimum support of 19 haplotype (minimum of ten individuals). These alleles would represent a set of newly identified alleles that are frequent.

##### AS3 (rare new alleles)

G1K alleles that have a haplotype support between 7 and 18 (minimum four individuals). This group of new alleles with less confidence in terms of haplotype support is called rare alleles. Despite the rarity of these alleles, we believe that they are genuine allelic variants, because the chance that 7 identical haplotypes within 5008 independent haplotypes are caused by sequencing errors is highly unlikely.

As alleles can be duplicated or diverged from each other, we further subdivided all alleles into three other categories (Fig. S1).

*Alleles for Group genes*: alleles for which the genes are marked as duplicated in the *IG* loci. These are *IGHV1-69*, *IGHV1-69D*; *IGHV3-43*, *IGHV 3-43D*; *IGHV3-23*, *IGHV3-23D*; *IGHV3-64*, *IGHV3-64D* and *IGHV2-70*, *IGHV2-70D* pairs.

*Alleles for operationally indistinguishable (OI) genes:* As multiple *V* genes are paralogous [63, 67], the mapping of short reads to such genes can be erroneous, influencing the subsequently derived alleles. Mutations on the alleles of such genes can thus easily be false positives, even after using stringent parameters. We denote these genes as operationally indistinguishable (OI) genes. As these genes can be recognized based on their similarity [37], we generated a neighbor-joining (NJ) tree for all *V* genes on the *IGH*, *IGK* and *IGL* loci, separately. The genes sharing a clade with a short branch length i.e., 0.02, are called OI genes (Fig. S1); and the corresponding alleles as OI alleles.

*Alleles for self-evident (SE) genes*: Alleles that are *not* annotated as group or OI alleles.

The alleles that fall into AS1 category i.e. known alleles are also termed as group or OI alleles as these resources also contain false positives.

### Filtering out false positive alleles

The G1K alleles were scrutinized manually.

1. Alleles with stop codons were removed from the final set.

2. Alleles with mutations or frameshift mutations absent in any of the following resources were removed: ESP (https://evs.gs.washington.edu/EVS/), TOPMed (https://www.nhlbi.nih.gov/science/trans-omics-precision-medicine-topmed-program), gnomAD (https://gnomad.broadinstitute.org/), and ProjectMine (https://www.projectmine.com/).

3. All the alleles of group genes or OI genes (e.g. *IGHV1-69D* gene from IMGT and alleles *for IGHV1-69* gene (group genes); alleles for all *IGHA1* and *IGHA2* genes (OI genes)) were aligned. We removed alleles within group genes and OI genes when a mutation of an allele is shared between alleles belonging to different genes within the group (pointing towards a mis-alignment of a read) except when this mutation is present in one of the databases across multiple alleles. For example, an allele of *IGHA1* has a position mutated i.e., A- > C exclusively, and two alleles from *IGHA2* also have similar mutation at the same aligned position, then all the three alleles were considered as false positives and filtered out.

### Identifying mutation patterns in the filtered alleles

To identify the mutation patterns, we performed alignments of all alleles per gene. The alleles were compiled from our database pmIG and the other existing databases i.e. IMGT, IgPdb and VBASE2. In the alignments per gene, the mutating positions are identified for all alleles. In the complete set of alignments per gene, the mutated positions for all alleles of that gene are compared and characterized as *new* (when the mutation is only seen in our resource) or *known* (when the mutation is seen in one of the other resources). We have done this for mutations in the *V* region as well as the leader sequences. The positions added by our resource are mentioned and the pattern of the mutations i.e. conserved (= at fixed positions) or random (= scattered, as caused by SHM) is identified.

### Mapping population information to the identified alleles

G1K alleles are annotated with superpopulation information (Tables S2–S4) into four categories: (1) ALL, present in all superpopulations; (2) AFR, only present in Africans; (3) AFR SHARED, present in African and at least one of the other superpopulations, but not all; and (4) NON-AFR, present in at least one of the superpopulations, but not in Africans.

### Variants in RSS haplotypes

We retrieved the RSS variants from the 40 bases adjacent to 3′ *IGHV* genes and the 5′ *IGHJ* genes (having 23-bp spacers), and from the 30 bases adjacent to 5′ and 3′ *IGHD* genes (having 12-bp spacers). Similarly, variants were retrieved from the 30 bases adjacent to 3′ *IGKV* and 5′ *IGLJ* genes (having 12-bp spacers) and from the 40 bases adjacent to 5′ *IGKJ* and 3′ *IGLV* genes (having 23-bp spacers). The perfect RSS sequence has a conserved heptamer “CACAGTG”, a conserved nonamer “ACAAAAACC”, and a specific length of the spacer sequence (23 bp or 12 bp) [15]. Mutations in heptamer and nonamer sequences as well as a deviating length of the spacer (less than 23 bp or 12 bp) directly affect the recombination frequency of the linked genes [15, 32, 46, 52].

### Phylogenetic trees for alleles

Maximum Likelihood (ML) trees were built for the alleles using RAxML [68]. The PROTGAMMAJTT model was used to build the trees with 100 bootstraps. The trees were visualized using the iTOL server [69]. The trees taxa were colored as per AS classification; the population level annotation is displayed in binary format and the frequency of alleles as text. A few alleles derived from loci not meant for evaluation were used as an outgroup in all the ML trees.

### Independent validation of the mutation patterns in the pmIG alleles

To determine whether the mutations in the germline allele sequences are caused by somatic hypermutations (SHM), we aligned the alleles to the rearranged *IGH* sequences derived from the transcriptomics data of antigen-experienced B cells i.e. sorted HBsAg^+^ B cells, sampled after primary Hepatitis B vaccination [70]. The raw FASTQ files were obtained from SRA (SRP068400). Paired-end reads were joined using fastq-join (ea-utils) with default settings and filtered for minimum Phred quality of 30 over at least 75% of bases. IMGT/HighV-Quest [34] was used for sequence annotation and functional *IGH* sequences were retained. We selected 20 B-cell receptor sequences from affinity-maturated B-cells after Hepatitis B vaccination for each gene randomly and aligned those sequences to the all the alleles (detected by us (pmIG), IMGT, IgPdb and VBASE2) for the respective genes. The mutating positions are marked and the mutation patterns were compared between germline alleles and antigen-experienced *IGH* sequences from HepB study.

### Genetic diversity and migration events of population based on *IG* loci

The VCF file of the complete individual locus, i.e. *IGH* (Chr14 [106032614, 107288051, complement]; Number of SNPs: 48,190), *IGK* (Chr2 [89890568, 90274235]; Number of SNPs: 24,706), [89156874, 89630436, complement]; Number of SNPs: 32,557) and *IGL* (Chr22 [22380474, 23265085]; Number os SNPs: 32,708), was subjected to a principal component analysis (PCA) using the R Bioconductor package ‘SNPRelate’ [71]. We then calculated the pairwise population differentiation, which is based on levels of differentiation in polymorphism frequencies across populations, as quantified by the fixation index (F_ST_). F_ST_ is proportional to the evolutionary branch length between each pair of populations. F_ST_ distances between populations were visualized with a Neighbor joining tree. We used TreeMix [72] that uses the composite likelihood to build the population trees. Six migration edges are tested for significance using 500 SNPs per block (-k 500). As “Out of Africa” is the most accepted theory [73] we used Yoruba population (YRI) of the African superpopulation an outgroup for building the migration trees.

## Supplementary information

Supplementary Figures

Supplementary Tables S1-S9

## Data Availability

The full set of alleles (AS1-3) and all R and Python scripts used for analysis are available from GitHub (https://github.com/InduKhatri/pmIG). The alleles are available in three different files, according to the confidence level. The alleles are also made available via online database https://pmtrig.lumc.nl/.
