# Supplementary material for: Population matched (pm) germline allelic variants of immunoglobulin (IG) loci: Relevance in infectious diseases and vaccination studies in human populations
Source: Genes Immun. 2021 Jun 12;22(3):172–86. doi: 10.1038/s41435-021-00143-7 (PMC8196923; doi:10.1038/s41435-021-00143-7)
Supplement: Supplementary file 1 — Supplementary Figures [file 41435_2021_143_MOESM1_ESM.pdf]

Population matched (pm) germline allelic variants of immunoglobulin (*IG*) loci: Relevance in infectious diseases and vaccination studies in human populations

Indu Khatri[1, 2], Magdalena A. Berkowska[1], Erik B. van den Akker[2, 3, 4], Cristina Teodosio[1], Marcel J.T. Reinders[2, 4], Jacques J.M. van Dongen[1]

Affiliations:

[1]Dept. Immunology, Leiden University Medical Center, 2333 ZA Leiden, The Netherlands.

[2] Leiden Computational Biology Center, Leiden University Medical Center, 2333 ZC Leiden, The Netherlands.

[3] Dept. Molecular Epidemiology, Leiden University Medical Center, 2333 ZC Leiden, The Netherlands.

[4] Delft Bioinformatics Lab, Delft University of Technology, 2628 CD Delft, The Netherlands.

Correspondence:

Prof. Jacques J.M. van Dongen, MD, PhD

Dept. Immunohematology & Blood Transfusion
Leiden University Medical Center
2333 ZA Leiden
The Netherlands
Email: J.J.M.van_Dongen@lumc.nl

Author's Contact Information:

Indu Khatri: i.khatri@lumc.nl
Magdalena A. Berkowska: M.A.Berkowska@lumc.nl
Erik B. van den Akker: E.B.van_den_Akker@lumc.nl
Cristina Teodosio: C.I.Teodosio@lumc.nl
Marcel J.T. Reinders: M.J.T.Reinders@tudelft.nl
Jacques J.M. van Dongen: J.J.M.van_Dongen@lumc.nl

Running Title: pmIG germline allelic variants

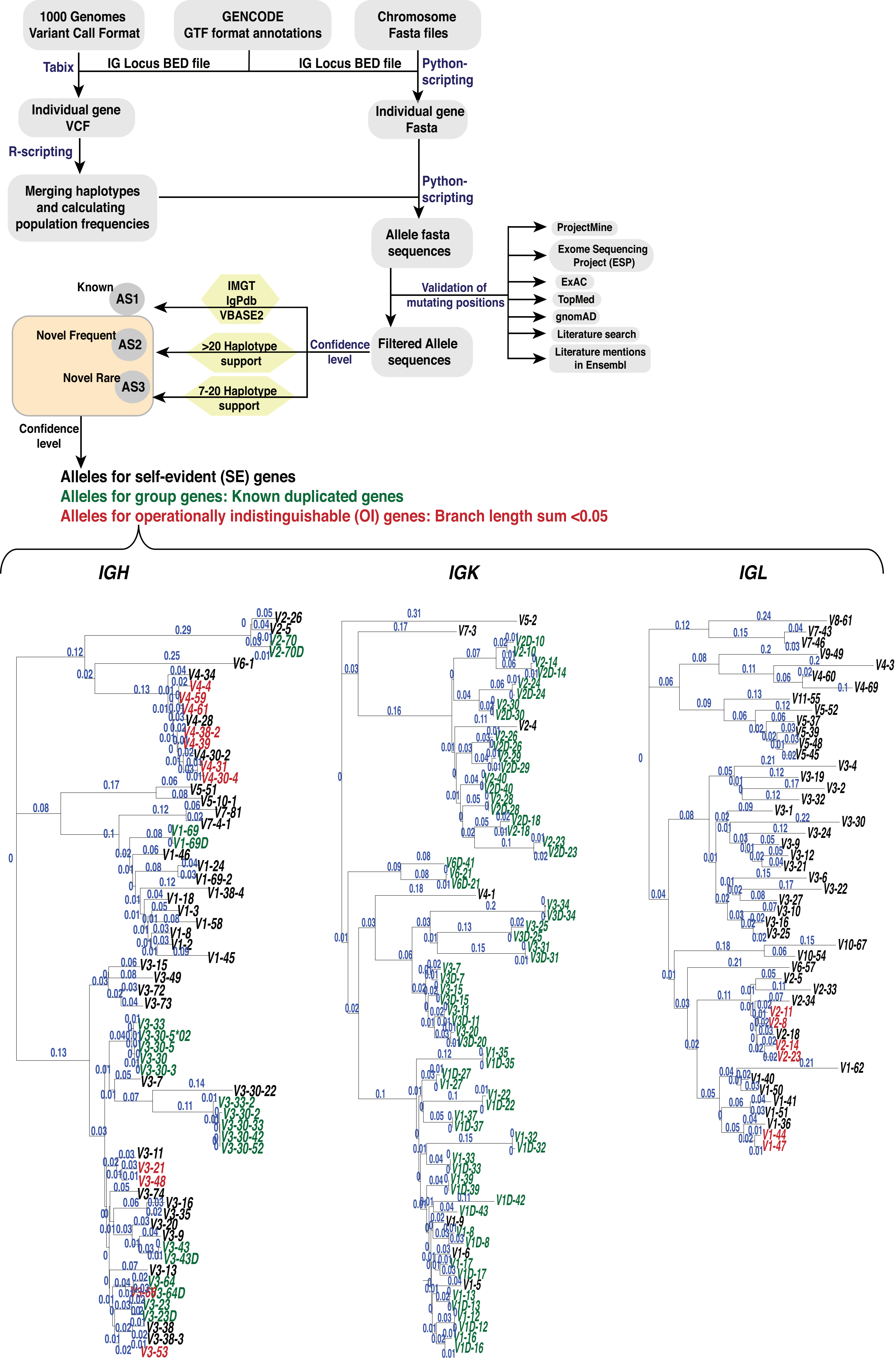

Figure S1: The pipeline developed to call alleles and assign confidence levels. The assigned confidence levels AS1-3 were identified based on known (AS1) and novel (AS2 and AS3) alleles. The novel alleles were further divided into self-evident, group and OI alleles highlighted in black, green and red colors, respectively on the NJ trees for IGHV, IGKV and IGLV. All the genes were obtained from GRCh37 version of human genomes. The genes absent in the human genome assembly were retrieved from the IMGT database.

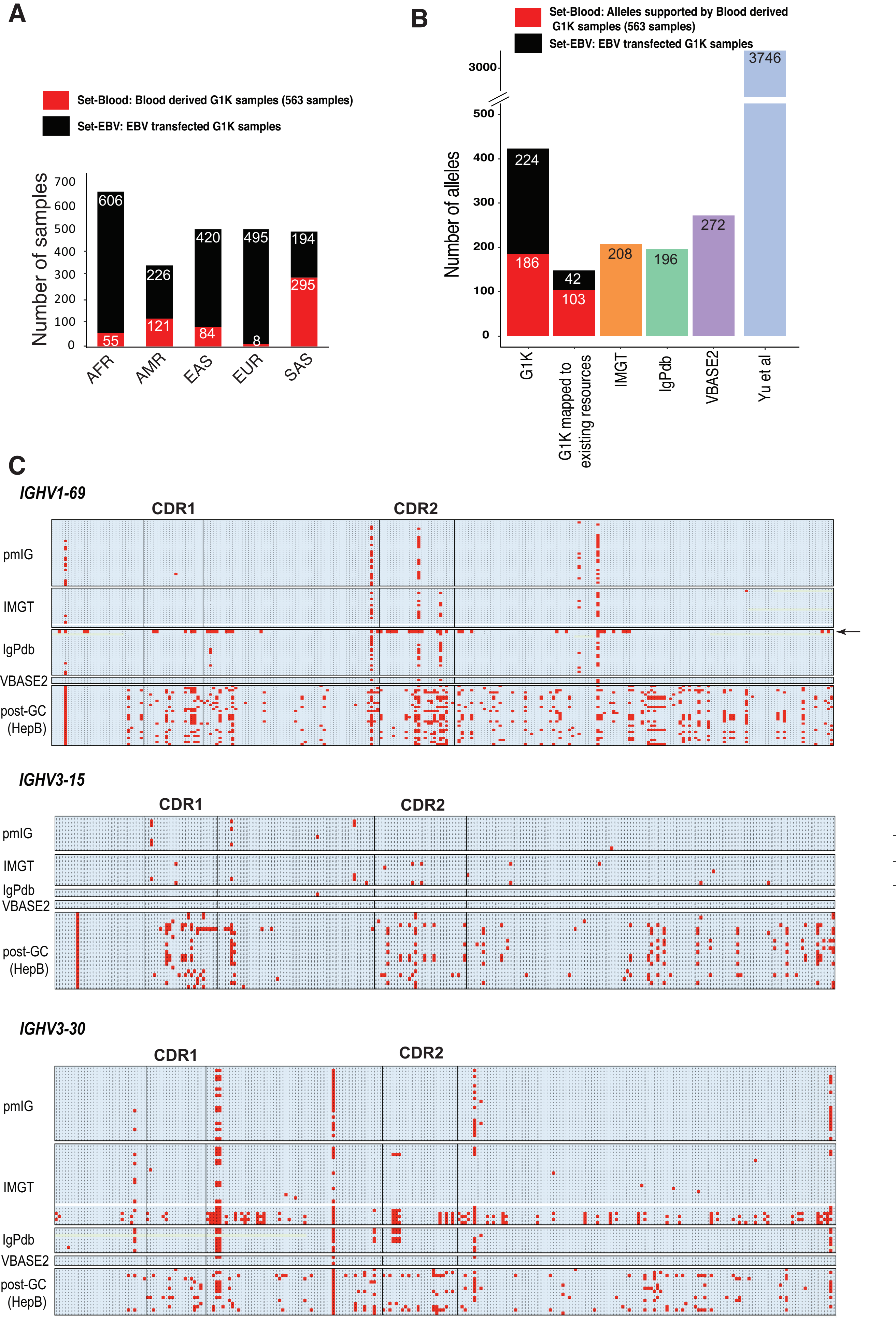

Figure S2: pmIG alleles are free of somatic-hypermutations (SHM). A) The distribution of samples in Set-1 and Set-2 from each super-population in G1K resource. B) The count of IGHV alleles in different resources. C) Mutations mapped to the alleles from pmIG, IMGT, IgPdb and VBASE2 databases with the corresponding IGHV sequences from the HepB vaccination study for IGHV1-69, IGHV3-15 and IGHV3-30 genes. The blue shade represents the conserved nucleotides and red shades indicate the mutated positions. Strikingly, the positions of the identified variant nucleotides of the IGHV1-69, IGHV3-15 and IGHV3-30 alleles in the pmIG database are not at random, but show restricted patterns, restricted to limited nucleotide positions, the same as found in other IG databases. However, IGHV1-69 sequences in the IgPdb databases show additional variant nucleotides, which are mutated in the post germinal center (GC) B-cells after Hepatitis B vaccination. IGHV1-69D and duplicated genes of IGHV3-30 were added to the IMGT alleles to represent that the mutation patterns of the pmIG alleles are consistent and the false positives are controlled.

IGHV1-2

```
V1-2_01         atggactggacctggaggatcctcttcttggtggcagcagccacaggagcccactcccaggtgcagctggtgcagtctggggctgaggtgaa
V1-2_02         atggactggacctggaggatcctcttcttggtggcagcagccacaggagcccactcccaggtgcagctggtgcagtctggggctgaggtgaa
V1-2_03         atggactggacctggaggatcctcttcttggtggcagcagccacaggagcccactcccaggtgcagctggtgcagtctggggctgaggtgaa
V1-2_04         atggactggacctggaggatcctcttcttggtggcagcagccacaggagcccactcccaggtgcagctggtgcagtctggggctgaggtgaa
V1-2_05         atggactggacctggaggatcctcttcttggtggcagcagccacaggagcccactcccaggtgcagctggtgcagtctggggctgaggtgaa
IGHV1-2*01      atggactggacctggaggatcctcttcttggtggcagcagccacaggagcccactcccaggtgcagctggtgcagtctggggctgaggtgaa
IGHV1-2*02      atggactggacctggaggatcctcttcttggtggcagcagccacaggagcccactcccaggtgcagctggtgcagtctggggctgaggtgaa
IGHV1-2*03      atggactggacctggaggatcctcttcttggtggcagcagccacaggagcccactcccaggtgcagctggtgcagtctggggctgaggtgaa
IGHV1-2*04      atggactggacctggaggatcctcttcttggtggcagcagccacaggagcccactcccaggtgcagctggtgcagtctggggctgaggtgaa
IGHV1-2*06      atggactggacctggaggatcctcttcttggtggcagcagccacaggagcccactcccaggtgcagctggtgcagtctggggctgaggtgaa
IGHV1-2*05      ---------------------------------------------------------caggtgcagctggtgcagtctggggctgaggtgaa
IP_V1-2*p05     --------------------------------------------------------------------------------------------
IP_V1-2*p06     ---------------------------------------------------------caggtgcagctggtgcagtctggggctgaggtgaa
IP_V1-2*p07     ---------------------------------------------------------caggtgcagctggtgcagtctggggctgaggtgaa
IP_V1-2*p08     ---------------------------------------------------------caggtgcagctggtgcagtctggggctgagatgaa
V_V1-2*01       ---------------------------------------------------------caggtgcagctggtgcagtctggggctgaggtgaa
V_V1-2*02       ---------------------------------------------------------caggtgcagctggtgcagtctggggctgaggtgaa
V_V1-2*03       ---------------------------------------------------------caggtgcagctggtgcagtctggggctgaggtgaa


V1-2_01         gaagcctggggcctcagtgaaggtctcctgcaaggcttctggatacaccttcaccggctactatatgcactgggtgcgacaggcccct
V1-2_02         gaagcctggggcctcagtgaaggtctcctgcaaggcttctggatacaccttcaccggctactatatgcactgggtgcgacaggcccct
V1-2_03         gaagcctggggcctcagtgaaggtctcctgcaaggcttctggatacaccttcaccggctactatatgcactgggtgcgacaggcccct
V1-2_04         gaagcctggggcctcagtgaaggtctcctgcaaggcttctggatacaccttcaccggctactatatgcactgggtgcgacaggcccct
V1-2_05         aaagcctggggcctcagtgaaggtctcctgcaaggcttctggatacaccttcaccggctactatatgcactgggtgcgacaggcccct
IGHV1-2*01      gaagcctggggcctcagtgaaggtctcctgcaaggcttctggatacaccttcaccggctactatatgcactgggtgcgacaggcccct
IGHV1-2*02      gaagcctggggcctcagtgaaggtctcctgcaaggcttctggatacaccttcaccggctactatatgcactgggtgcgacaggcccct
IGHV1-2*03      gaagcttggggcctcagtgaaggtctcctgcaaggcttctggatacaccttcaccggctactatatgcactgggtgcnacaggcccct
IGHV1-2*04      gaagcctggggcctcagtgaaggtctcctgcaaggcttctggatacaccttcaccggctactatatgcactgggtgcgacaggcccct
IGHV1-2*06      gaagcctggggcctcagtgaaggtctcctgcaaggcttctggatacaccttcaccggctactatatgcactgggtgcgacaggcccct
IGHV1-2*05      gaagcctggggcctcagtgaaggtctcctgcaaggcttctggatacaccttcaccggctactatatgcactgggtgcgacaggcccct
IP_V1-2*p05     ----------------------------------------------------------------------------------------
IP_V1-2*p06     gaagcctggggcctcagtgaaggtctcctgcaaggcttctggatacaccttcaccggctactatatgcactgggtgcgacaggcccct
IP_V1-2*p07     gaagcctggggcctcagtgaaggtctcctgcaaggcttctggatacaccttcaccggctactatatgcactgggtgcgacaggcccct
IP_V1-2*p08     gaagcctggggcctcagtgaaggtctcctgcaaggcttctggatacaccttcaccggctactatatgcactgggtgcgacaggcccct
V_V1-2*01       gaagcctggggcctcagtgaaggtctcctgcaaggcttctggatacaccttcaccggctactatatgcactgggtgcgacaggcccct
V_V1-2*02       gaagcctggggcctcagtgaaggtctcctgcaaggcttctggatacaccttcaccggctactatatgcactgggtgcgacaggcccct
V_V1-2*03       gaagcttggggcctcagtgaaggtctcctgcaaggcttctggatacaccttcaccggctactatatgcactgggtgcxacaggcccct
```

```
V1-2_01        ggacaagggcttgagtggatgggatggatcaaccctaacagtggtggcacaaactatgcacagaagtttcagggcagggtcaccatgaccagggacac
V1-2_02        ggacaagggcttgagtggatgggatggatcaaccctaacagtggtggcacaaactatgcacagaagtttcagggctgggtcaccatgaccagggacac
V1-2_03        ggacaagggcttgagtggatgggacggatcaaccctaacagtggtggcacaaactatgcacagaagtttcagggcagggtcaccatgaccagggacac
V1-2_04        ggacaagggcttgagtggatgggacggatcaaccctaacagtggtggcacaaactatgcacagaagtttcagggcagggtcaccatgaccagggacac
V1-2_05        ggacaagggcttgagtggatgggatggatcaaccctaacagtggtggcacaaactatgcacagaagtttcagggcagggtcaccatgaccagggacac
IGHV1-2*01     ggacaagggcttgagtggatgggacggatcaaccctaacagtggtggcacaaactatgcacagaagtttcagggcagggtcaccagtaccagggacac
IGHV1-2*02     ggacaagggcttgagtggatgggatggatcaaccctaacagtggtggcacaaactatgcacagaagtttcagggcagggtcaccatgaccagggacac
IGHV1-2*03     ggacaagggcttgagtggatgggatggatcaaccctaacagtggtggcacaaactatgcacagaagtttcagggcagggtcaccatgaccagggacac
IGHV1-2*04     ggacaagggcttgagtggatgggatggatcaaccctaacagtggtggcacaaactatgcacagaagtttcagggctgggtcaccatgaccagggacac
IGHV1-2*06     ggacaagggcttgagtggatgggacggatcaaccctaacagtggtggcacaaactatgcacagaagtttcagggcagggtcaccatgaccagggacac
IGHV1-2*05     ggacaagggcttgagtggatgggacggatcaaccctaacagtggtggcacaaactatgcacagaagtttcagggcagggtcaccatgaccagggacac
IP_V1-2*p05    ------gggcttgagtggatgggacggatcaaccctaacagtggtggcacaaactatgcacagaagtttcagggcagggtcaccatgaccagggacac
IP_V1-2*p06    ggacaagggcttgagtggatgggacggatcaaccctaacagtggtggcacaaactatgcacagaagtttcagggcagggtcaccatgaccagggacac
IP_V1-2*p07    ggacaagggcttgagtggatgggacggatcaaccctaacagtggtggcacaaactatgcacagaagtttcagggcagggtcaccatgaccagggacac
IP_V1-2*p08    ggacaagggcttgagtggatgggacggatcaaccctaacagtggtggcacaaactatgcacagaagtttcagggcagggtcaccatgaccagggacac
V_V1-2*01      ggacaagggcttgagtggatgggacggatcaaccctaacagtggtggcacaaactatgcacagaagtttcagggcagggtcaccagtaccagggacac
V_V1-2*02      ggacaagggcttgagtggatgggatggatcaaccctaacagtggtggcacaaactatgcacagaagtttcagggcagggtcaccatgaccagggacac
V_V1-2*03      ggacaagggcttgagtggatgggatggatcaaccctaacagtggtggcacaaactatgcacagaagtttcagggcagggtcaccatgaccagggacac
```

```
V1-2_01        gtccatcagcacagcctacatggagctgagcaggctgagatctgacgacacggccgtgtattactgtgcgaga--
V1-2_02        gtccatcagcacagcctacatggagctgagcaggctgagatctgacgacacggccgtgtattactgtgcgaga--
V1-2_03        gtccatcagcacagcctacatggagctgagcaggctgagatctgacgacacggtcgtgtattactgtgcgaga--
V1-2_04        gtccatcagcacagcctacatggagctgagcaggctgagatctgacgacacggccgtgtattactgtgcgaga--
V1-2_05        gtccatcagcacagcctacatggagctgagcaggctgagatctgacgacacggccgtgtattactgtgcgaga--
IGHV1-2*01     gtccatcagcacagcctacatggagctgagcaggctgagatctgacgacacggtcgtgtattactgtgcgagaga
IGHV1-2*02     gtccatcagcacagcctacatggagctgagcaggctgagatctgacgacacggccgtgtattactgtgcgagaga
IGHV1-2*03     gtccatcagcacagcctacatggagctgagcaggctgagatctgacgacacggccgtgtattactgtgcgagaga
IGHV1-2*04     gtccatcagcacagcctacatggagctgagcaggctgagatctgacgacacggccgtgtattactgtgcgagaga
IGHV1-2*06     gtccatcagcacagcctacatggagctgagcaggctgagatctgacgacacggccgtgtattactgtgcgagaga
IGHV1-2*05     gtccatcagcacagcctacatggagctgagcaggctgagatctgacgacacggtcgtgtattactgtgcgagaga
IP_V1-2*p05    gtccatcagcacagcctacatggagctgagcaggctgagatctgacgacacggtcgtgtattactgtgcgaga--
IP_V1-2*p06    gtccatcagcacagcctacatggagctgagcaggctgagatctgacgacacggccgtgtattactgtgcgaga--
IP_V1-2*p07    gtccatcagcacagcctacatggagctgagcaggctgagatctgacgacacggccgtgtattactgtgcgaga--
IP_V1-2*p08    gtccatcagcacagcctacatggagctgagcaggctgagatctgacgacacggccgtgtattactgtgcgagaga
V_V1-2*01      gtccatcagcacagcctacatggagctgagcaggctgagatctgacgacacggtcgtgtattactgtgcgaga--
V_V1-2*02      gtccatcagcacagcctacatggagctgagcaggctgagatctgacgacacggccgtgtattactgtgcgaga--
V_V1-2*03      gtccatcagcacagcctacatggagctgagcaggctgagatctgacgacacggccgtgtattactgtgcgaga--
```

IGHV1-3

```
V1-3_01        atggactggacctggaggatcctctttttggtggcagcagccacaggtgcccactcccaggtccagcttgtgcagtctggggctgaggtgaagaa
V1-3_02        atggactggacctggaggatcctctttttggtggcagcagccacaggtgtccactcccaggttcagctggtgcagtctggggctgaggtgaagaa
V1-3_03        atggactggacctggaggatcctctttttggtggcagcagccacaggtgtccactcccaggtccagctggtgcagtctggggctgaggtgaagaa
V1-3_04        atggactggacctggaggatcctctttttggtggcagcagccacaggtgcccactcccaggtccagcttgtgcagtctggggctgaggtgaagaa
V1-3_05        atggactggacctggaggatcctctttttggtggcagcagccacaggtgtccactcccaggtccagctggtgcagtctggggctgaggtgaagaa
V1-3_06        atggactggacctggaggatcctctttttggtggcagcagccacaggtgtccactcccaggttcagctggtgcagtctggggctgaggtgaagaa
V1-3_07        atggactggacctggaggatcctctttttggtggcagcagccacaggtgcccactcccaggtccagcttgtgcagtctggggctgaggtgaagaa
V1-3_08        atggactggacctggaggatcctctttttggtggcagcagccacaggtgtccactcccaggttcagctggtgcagtctggggctgaggtgaagaa
V1-3_09        atggactggacctggaggatcctctttttggtggcagcagccacaggtgtccactcccaggttcagctggtgcagtctggggctgaggtgaagaa
IGHV1-3*01     atggactggacctggaggatcctctttttggtggcagcagccacaggtgcccactcccaggtccagcttgtgcagtctggggctgaggtgaagaa
IGHV1-3*02     atggactggacctggaggatcctctttttggtggcagcagccacaggtgtccactcccaggttcagctggtgcagtctggggctgaggtgaagaa
IGHV1-3*03     atggactggacctggaggatcctctttttggtggcagcagccacaggtgtccactcccaggtccagctggtgcagtctggggctgaggtgaagaa
IGHV1-3*04     ---------------------------------------------------------caggtccagcttgtgcagtctggggctgaggtgaagaa
IP_V1-3*p04    ---------------------------------------------------------caggtccagcttgtgcagtctggggctgaggtgaagaa
IP_V1-3*p05    ---------------------------------------------------------caggtccagcttgtgcagtctggggctgaggtgaagaa
V_V1-3*01      ---------------------------------------------------------caggtccagcttgtgcagtctggggctgaggtgaagaa
V_V1-3*02      ---------------------------------------------------------caggttcagctggtgcagtctggggctgaggtgaagaa

V1-3_01        gcctggggcctcagtgaaggtttcctgcaaggcttctggatacaccttcactagctatgctatgcattgggtgcgccaggccccccggacaaa
V1-3_02        gcctggggcctcagtgaaggtttcctgcaaggcttctggatacaccttcactagctatgctatgcattgggtgcgccaggccccccggacaaa
V1-3_03        gcctggggcctcagtgaaggtttcctgcaaggcttctggatacaccttcactagctatgctatgcattgggtgcgccaggccccccggacaaa
V1-3_04        gcctggggcctcagtgaaggtttcctgcaaggcttctggatacaccttcactagctatgctatgcattgggtgcgccaggccccccggacaaa
V1-3_05        gcctggggcctcagtgaaggtttcctgcaaggcttctggatacaccttcactagctatgctatgcattgggtgcgccaggccccccggacaaa
V1-3_06        gcctggggcctcagtgaaggtttcctgcaaggcttctggatacaccttcactagctatgctatgcattgggtgcgccaggccccccggacaaa
V1-3_07        gcctggggcctcagtgaaggtttcctgcaaggcttctggatacaccttcactagctatgctatgcattgggtgcgccaggacccccggacaaa
V1-3_08        gcctggggcctcagtgaaggtttcctgcaaggcttctggatacaccttcactagctatgctatgcattgggtgcgccaggacccccggacaaa
V1-3_09        gcctggggcctcagtgaaggtttcctgcaaggcttctggatacaccttcactagctatgctatgcattgggtgcgccaggccccccggacaaa
IGHV1-3*01     gcctggggcctcagtgaaggtttcctgcaaggcttctggatacaccttcactagctatgctatgcattgggtgcgccaggccccccggacaaa
IGHV1-3*02     gcctggggcctcagtgaaggtttcctgcaaggcttctggatacaccttcactagctatgctatgcattgggtgcgccaggccccccggacaaa
IGHV1-3*03     gcctggggcctcagtgaaggtttcctgcaaggcttctggatacaccttcactagctatgctatgcattgggtgcgccaggccccccggacaaa
IGHV1-3*04     gcctggggcctcagtgaaggtttcctgcaaggcttctggatacaccttcactagctatgctatgcattgggtgcgccaggccccccggacaaa
IP_V1-3*p04    gcctggggcctcagtgaaggtttcctgcaaggcttctggatacaccttcactagctatgctatgcattgggtgcgccaggacccccggacaaa
IP_V1-3*p05    gcctggggcctcagtgaaggtttcctgcaaggcttctggatacaccttcactagctatgctatgcattgggtgcgccaggccccccggacaaa
V_V1-3*01      gcctggggcctcagtgaaggtttcctgcaaggcttctggatacaccttcactagctatgctatgcattgggtgcgccaggccccccggacaaa
V_V1-3*02      gcctggggcctcagtgaaggtttcctgcaaggcttctggatacaccttcactagctatgctatgcattgggtgcgccaggccccccggacaaa
```

```
V1-3_01        ggcttgagtggatgggatggatcaacgctggcaatggtaacacaaaatattcacagaagttccagggcagagtcaccattaccagggacaca
V1-3_02        ggcttgagtggatgggatggagcaacgctggcaatggtaacacaaaatattcacaggagttccagggcagagtcaccattaccagggacaca
V1-3_03        ggcttgagtggatgggatggatcaacgctggcaatggtaacacaaaatattcacaggagttccagggcagagtcaccattaccagggacaca
V1-3_04        ggcttgagtggatgggatggatcaacgctggcaatggtaacacaaaatattcacagaagttccagggcagagtcaccattaccagggacaca
V1-3_05        ggcttgagtggatgggatggatcaacgctggcaatggtaacacaaaatattcacaggagttccagggcagagtcaccattaccagggacaca
V1-3_06        ggcttgagtggatgggatggagcaacgctggcaatggtaacacaaaatattcacaggagttccagggcagagtcaccattaccagggacaca
V1-3_07        ggcttgagtggatgggatggatcaacgctggcaatggtaacacaaaatattcacagaagttccagggcagagtcaccattaccagggacaca
V1-3_08        ggcttgagtggatgggatggagcaacgctggcaatggtaacacaaaatattcacaggagttccagggcagagtcaccattaccagggacaca
V1-3_09        ggcttgagtggatgggatggagcaacgctggcaatggtaacacaaaatattcacaggagttccagggcagagtcaccattaccagggacaca
IGHV1-3*01     ggcttgagtggatgggatggatcaacgctggcaatggtaacacaaaatattcacagaagttccagggcagagtcaccattaccagggacaca
IGHV1-3*02     ggcttgagtggatgggatggagcaacgctggcaatggtaacacaaaatattcacaggagttccagggcagagtcaccattaccagggacaca
IGHV1-3*03     ggcttgagtggatgggatggatcaacgctggcaatggtaacacaaaatattcacaggagttccagggcagagtcaccattaccagggacaca
IGHV1-3*04     ggcttgagtggatgggatggatcaacactggcaatggtaacacaaaatattcacagaagttccagggcagagtcaccattaccagggacaca
IP_V1-3*p04    ggcttgagtggatgggatggatcaacgctggcaatggtaacacaaaatattcacagaagttccagggcagagtcaccattaccagggacaca
IP_V1-3*p05    ggcttgagtggatgggatggatcaacgctggcaatggtaacacaaaatattcacagaagttccagggcagagtcaccattaccaaggacaca
V_V1-3*01      ggcttgagtggatgggatggatcaacgctggcaatggtaacacaaaatattcacagaagttccagggcagagtcaccattaccagggacaca
V_V1-3*02      ggcttgagtggatgggatggagcaacgctggcaatggtaacacaaaatattcacaggagttccagggcagagtcaccattaccagggacaca
```

```
V1-3_01        tccgcgagcacagcctacatggagctgagcagcctgagatctgaagacacggctgtgtattactgtgcgaga--
V1-3_02        tccgcgagcacagcctacatggagctgagcagcctgagatctgaggacatggctgtgtattactgtgcgaga--
V1-3_03        tccgcgagcacagcctacatggagctgagcagcctgagatctgaggacatggctgtgtattactgtgcgaga--
V1-3_04        tccgcgagcacagcctacatggagctgagcagcctgagatctgaagacacggctgtgtattactatgcgaga--
V1-3_05        tccgcgagcacagcctacatggagctgagcagcctgagatctgaggacatggctgtgtattattgtgcgaga--
V1-3_06        tccgcgagcacagcctacatggagctgagcagcctgagatctgaggacatggctgtgtattattgtgcgaga--
V1-3_07        tccgcgagcacagcctacatggagctgagcagcctgagatctgaagacacggctgtgtattactgtgcgaga--
V1-3_08        tccgcgagcacagcctacatggagctgagcagcctgagatctgaggacatggctgtgtattactgtgcgaga--
V1-3_09        tccgcgagcacagcctacatggagctgagcagcctgagatctgaggacatggctgtgtattactatgcgaga--
IGHV1-3*01     tccgcgagcacagcctacatggagctgagcagcctgagatctgaagacacggctgtgtattactgtgcgagaga
IGHV1-3*02     tccgcgagcacagcctacatggagctgagcagcctgagatctgaggacatggctgtgtattactgtgcgagaga
IGHV1-3*03     tccgcgagcacagcctacatggagctgagcagcctgagatctgaggacatggctgtgtattactgtgcgagaga
IGHV1-3*04     tccgcgagcacagcctacatggagctgagcagcctgagatctgaagacacggct--------------------
IP_V1-3*p04    tccgcgagcacagcctacatggagctgagcagcctgagatctgaagacacggctgtgtattactgtgcgagaga
IP_V1-3*p05    tccgcgagcacagcctacatggagctgagcagcctgagatctgaagacacggctgtgtattactgtgcgagaga
V_V1-3*01      tccgcgagcacagcctacatggagctgagcagcctgagatctgaagacacggctgtgtattactgtgcgaga--
V_V1-3*02      tccgcgagcacagcctacatggagctgagcagcctgagatctgaggacatggctgtgtattactgtgcgaga--
```

IGHV1-8

```
V1-8_01        atggactggacctggaggatcctcttcttggtggcagcagctacaagtgcccactcccaggtgcagctggtgcagtctggggctgaggtgaaga
V1-8_02        atggactggacctggaggatcctcttcttggtggcagcagctacaagtgcccactcccaggtgcagctggtgcagtctggggctgaggtgaaga
V1-8_03        atggactggacctggaggatcctcttcttggtggcagcagctacaagtgcccactcccaggtgcagctggtgcagtctggggctgaggtgaaga
V1-8_04        atggactggacctggaggatcctcttcttggtggcagcagctacaagtgcccactcccaggtgcagcttgtgcagtctggggctgaggtgaaga
V1-8_05        atggactggacctggaggatcctcttcttggtggcagcagctacaagtgcccactcccaggtgcagctggtgcagtctggggctgaggtgaaga
IGHV1-8*01     atggactggacctggaggatcctcttcttggtggcagcagctacaagtgcccactcccaggtgcagctggtgcagtctggggctgaggtgaaga
IGHV1-8*03     -----------------gatcctcttcttggtggcagcagctacaagtgcccactcccaggtgcagctggtgcagtctggggctgaggtgaaga
IGHV1-8*02     ---------------------------------------------------------caggtgcagctggtgcagtctggggctgaggtgaaga
IP_V1-8*p04    ---------------------------------------------------------caggtgcagctggtgcagtctggggctgaggtgaaga
IP_V1-8*p04    -------------------------------------------------------------------------------------aggtgaaga
IP_V1-8*p05    ---------------------------------------------------------caggtgcagctggtgcagtctggggctgaggtgaaga
V_V1-8*01      ---------------------------------------------------------caggtgcagctggtgcagtctggggctgaggtgaaga

V1-8_01        agcctggggcctcagtgaaggtctcctgcaaggcttctggatacaccttcaccagttatgatatcaactgggtgcgacaggccact
V1-8_02        agcctggggcctcagtgaaggtctcctgcaaggcttctggatacaccttcaccagctatgatatcaactgggtgcgacaggccact
V1-8_03        agcctggggcctcagtgaaggtctcctgcaaggcttctggatacaccttcaccagctatgatatcaactgggtgcgacaggccact
V1-8_04        agcctggggcctcagtgaaggtctcctgcaaggcttctggatacaccttcaccagttatgatatcaactgggtgcgacaggccact
V1-8_05        agcctggggcctcagtgaaggtctcctgcaaggcttctggatacaccttcaccagttatgatatcaactgggtgcgacaggccact
IGHV1-8*01     agcctggggcctcagtgaaggtctcctgcaaggcttctggatacaccttcaccagttatgatatcaactgggtgcgacaggccact
IGHV1-8*03     agcctggggcctcagtgaaggtctcctgcaaggcttctggatacaccttcaccagctatgatatcaactgggtgcgacaggccact
IGHV1-8*02     agcctggggcctcagtgaaggtctcctgcaaggcttctggatacaccttcaccagctatgatatcaactgggtgcgacaggccact
IP_V1-8*p04    agcctggggcctcagtgaaggtctcctgcaaggcttctggatacaccttcaccagctatgatatcaactgggtgcgacaggccact
IP_V1-8*p04    agcctggggcctcagtgaaggtctcctgcaaggcttctggatacaccttcaccagctatgatatcaactgggtgcgacaggccact
IP_V1-8*p05    agcctggggcctcagtgaaggtctcctgcaaggcttctggatacaccttcaccagctatgatatcaactgggtgcgacaggccact
V_V1-8*01      agcctggggcctcagtgaaggtctcctgcaaggcttctggatacaccttcaccagttatgatatcaactgggtgcgacaggccact

V1-8_01        ggacaagggcttgagtggatgggatggatgaaccctaacagtggtaacacaggctatgcacagaagttccagggcagagtcaccatgacca
V1-8_02        ggacaagggcttgagtggatgggatggatgaaccctaacagtggtaacacaggctatgcacagaagttccagggcagagtcaccatgacca
V1-8_03        ggacaagggcttgagtggatgggatggatgaaccctaacagtggtaacacaggctatgcacagaagttccagggcagagtcaccattacca
V1-8_04        ggacaagggcttgagtggatgggatggatgaaccctaacagtggtaacacaggctatgcacagaagttccagggcagagtcaccatgacca
V1-8_05        ggacaagggcttgagtggatgggatggatgaaccctaacagtggtaacacaggctatgcacagaagttccagggcagagtcaccattacca
IGHV1-8*01     ggacaagggcttgagtggatgggatggatgaaccctaacagtggtaacacaggctatgcacagaagttccagggcagagtcaccatgacca
IGHV1-8*03     ggacaagggcttgagtggatgggatggatgaaccctaacagtggtaacacaggctatgcacagaagttccagggcagagtcaccattacca
IGHV1-8*02     ggacaagggcttgagtggatgggatggatgaaccctaacagtggtaacacaggctatgcacagaagttccagggcagagtcaccatgacca
IP_V1-8*p04    ggacaagggcttgagtggatgggatggatgaaccctaacagtggtaacacaggctatgcacagaagttccagggcagagtcaccattacca
IP_V1-8*p04    ggacaagggcttgagtggatgggatggatgaaccctaacagtggtaacacaggctatgcacagaagttccagggcagagtcaccattacca
IP_V1-8*p05    ggacaagggcttgagtggatgggatggatgaaccctaacagtggtaacacaggctatgcacagaagttccagggcagagtcaccatgacca
V_V1-8*01      ggacaagggcttgagtggatgggatggatgaaccctaacagtggtaacacaggctatgcacagaagttccagggcagagtcaccatgacca
```

```
V1-8_01         ggaacacctccataagcacagcctacatggagctgagcagcctgagatctgaggacacggccgtgtattactgtgcgaga--
V1-8_02         ggaacacctccataagcacagcctacatggagctgagcagcctgagatctgaggacacggccgtgtattactgtgcgaga--
V1-8_03         ggaacacctccataagcacagcctacatggagctgagcagcctgagatctgaggacacggccgtgtattactgtgcgaga--
V1-8_04         ggaacacctccataagcacagcctacatggagctgagcagcctgagatctgaggacacggccgtgtattactgtgcgaga--
V1-8_05         ggaacacctccataagcacagcctacatggagctgagcagcctgagatctgaggacacggccgtgtattactgtgcgaga--
IGHV1-8*01      ggaacacctccataagcacagcctacatggagctgagcagcctgagatctgaggacacggccgtgtattactgtgcgagagg
IGHV1-8*03      ggaacacctccataagcacagcctacatggagctgagcagcctgagatctgaggacacggccgtgtattactgtgcgagagg
IGHV1-8*02      ggaacacctccataagcacagcctacatggagctgagcagcctgagatctgaggacacggccgtgtattactgtgcgagagg
IP_V1-8*p04     ggaacacctccataagcacagcctacatggagctgagcagcctgagatctgaggacacggccgtgtattactgtgcgagagg
IP_V1-8*p04     ggaacacctccataagcacagcctacatggagctgagcagcctgagatctgaggacacggccg-------------------
IP_V1-8*p05     ggaacacctccgtaagcacagcctacatggagctgagcagcctgagatctgaggacacggccgtgtattactgtgcgagagg
V_V1-8*01       ggaacacctccataagcacagcctacatggagctgagcagcctgagatctgaggacacggccgtgtattactgtgcgaga--
```

IGHV1-18

```
V1-18_01        atggactggacctggagcatcctt ttcttggtggcagcag caacaggtgcccactcccaggtt cagctggtgcagtctgga gctgaggtgaa
```

```
V1-18_01        atggactggacctggagcatcctttcttggtggcagcagcaacaggtgcccactcccaggttcagctggtgcagtctggagctgaggtgaa
V1-18_02        atggactggacctggagcatcctttcttggtggcagcagcaacaggtgcccactcccaggttcagctggtgcagtctggagctgaggtgaa
V1-18_03        atggactggacctggagcatcctttcttggtggcagcagcaacaggtgcccactcccaggttcagctggtgcagtctggagctgaggtgaa
V1-18_04        atggactggacctggagcatcctttcttggtggcagcagcaacaggtgcccactcccaggttcagctggtgcagtctggagctgaggtgaa
V1-18_05        atggactggacctggagcatcctcttcttggtggcagcagcaacaggtgcccactcccaggttcagctggtgcagtctggagctgaggtgaa
IGHV1-18*01     atggactggacctggagcatcctttcttggtggcagcaccaacaggtgcccactcccaggttcagctggtgcagtctggagctgaggtgaa
IGHV1-18*02     atggactggacctggagcatcctttcttggtggcagcagcaacaggtgcccactcccaggttcagctggtgcagtctggagctgaggtgaa
IGHV1-18*04     atggactggacctggagcatcctttcttggtggcagcagcaacaggtgcccactcccaggttcagctggtgcagtctggagctgaggtgaa
IGHV1-18*03     ---------------------------------------------------------caggttcagctggtgcagtctggagctgaggtgaa
IP_V1-18*p03    ---------------------------------------------------------caggttcagctggtgcagtctggagctgaggtgaa
IP_V1-18*p04    --------------------------------------------------------------------------------------------
IP_V1-18*p05    ---------------------------------------------------------caggttcagctggtgcagtctggagctgaggtgaa
IP_V1-18*p06    ---------------------------------------------------------caggtccagctggtgcagtctgggctgaggtgaa
IP_V1-18*p07    ---------------------------------------------------------caggtgcagctggtgcagtctggagctgaggtgaa
IP_V1-18*p08    ---------------------------------------------------------caggtgcagctggtgcagtctgggctgaggtgaa
V_V1-18*01      ---------------------------------------------------------caggttcagctggtgcagtctggagctgaggtgaa
```

```
V1-18_01        gaagcctggggcctcagtgaaggtctcctgcaaggcttctggttacacctttaccagctatggtatcagctgggtgcgacaggcccct
V1-18_02        gaagcctggggcctcagtgaaggtctcctgcaaggcttctggttacacctttaccagctacggtatcagctgggtgcgacaggcccct
V1-18_03        gaagcctggggcctcagtgaaggtctcctgcaaggcttctggttacacctttaccagctatggtatcagctgggtgcgacaggcccct
V1-18_04        gaagcctggggcctcagtgaaggtctcctgcaaggcttctggttacacctttaccagctatggtatcagctgggtgcgacaggcccct
V1-18_05        gaagcctggggcctcagtgaaggtctcctgcaaggcttctggttacacctttaccagctatggtatcagctgggtgcgacaggcccct
IGHV1-18*01     gaagcctggggcctcagtgaaggtctcctgcaaggcttctggttacacctttaccagctatggtatcagctgggtgcgacaggcccct
IGHV1-18*02     gaagcctggggcctcagtgaaggtctcctgcaaggcttctggttacacctttaccagctatggtatcagctgggtgcgacaggcccct
IGHV1-18*04     gaagcctggggcctcagtgaaggtctcctgcaaggcttctggttacacctttaccagctacggtatcagctgggtgcgacaggcccct
IGHV1-18*03     gaagcctggggcctcagtgaaggtctcctgcaaggcttctggttacacctttaccagctatggtatcagctgggtgcgacaggcccct
IP_V1-18*p03    gaagcctggggcctcagtgaaggtctcctgcaaggcttctggttacacctttaccagctatggtatcagctgggtgcgacaggcccct
IP_V1-18*p04    ----------------------------------gcttctggttacacctttaccagctatggtatcagctgggtgcgacaggcccct
IP_V1-18*p05    gaagcctggggcctcagtgaaggtctcctgcaaggcttctggttacacctttaccagctatggtatcagctgggtgcgacaggcccct
IP_V1-18*p06    gaagcctggggcctcagtgaaggtctcctgcaaggcttctggttacacctttaccagctatggtatcagctgggtgcgacaggcccct
IP_V1-18*p07    gaagcctggggcctcagtgaaggtctcctgcaaggcttctggttacacctttaccagctatggtatcagctgggtgcgacaggcccct
IP_V1-18*p08    gaagcctggggcctcagtgaaggtctcctgcaaggcttctggttacacctttaccagctatggtatcagctgggtgcgacaggcccct
V_V1-18*01      gaagcctggggcctcagtgaaggtctcctgcaaggcttctggttacacctttaccagctatggtatcagctgggtgcgacaggcccct
```

```
V1-18_01         ggacaagggcttgagtggatgggatggatcagcgcttacaatggtaacacaaactatgcacagaagctccagggcagagtcaccatgaccacagac
V1-18_02         ggacaagggcttgagtggatgggatggatcagcgcttacaatggtaacacaaactatgcacagaagctccagggcagagtcaccatgaccacagac
V1-18_03         ggacaagggcttgagtggatgggatggatcagcgcttacaatggtgacacaaactatgcacagaagctccagggcagagtcaccatgaccacagac
V1-18_04         ggacaagggcttgagtggatgggatggatcagcgcttacaatggtaacacaaactatgcacagaagctccaggacagagtcaccatgaccacagac
V1-18_05         ggacaagggcttgagtggatgggatggatcagcgcttacaatggtaacacaaactatgcacagaagctccagggcagagtcaccatgaccacagac
IGHV1-18*01      ggacaagggcttgagtggatgggatggatcagcgcttacaatggtaacacaaactatgcacagaagctccagggcagagtcaccatgaccacagac
IGHV1-18*02      ggacaagggcttgagtggatgggatggatcagcgcttacaatggtaacacaaactatgcacagaagctccagggcagagtcaccatgaccacagac
IGHV1-18*04      ggacaagggcttgagtggatgggatggatcagcgcttacaatggtaacacaaactatgcacagaagctccagggcagagtcaccatgaccacagac
IGHV1-18*03      ggacaagggcttgagtggatgggatggatcagcgcttacaatggtaacacaaactatgcacagaagctccagggcagagtcaccatgaccacagac
IP_V1-18*p03     ggacaagggcttgagtggatgggatggatcagcgcttacaatggtaacacaaactatgcacagaagctccagggcagagtcaccatgaccacagac
IP_V1-18*p04     ggacaagggcttgagtggatgggatggatcagcgcttacaatggtaacacaaactatgcacagaagctccagggcagagtcaccatgaccacagac
IP_V1-18*p05     ggacaagggcttgagtggatgggatggatcagcgcttacaatggtgacacaaactatgcacagaagctccagggcagagtcaccatgaccacagac
IP_V1-18*p06     ggacaagggcttgagtggatgggatggatcagcgcttacaatggtaacacaaactatgcacagaagctccagggcagagtcaccatgaccacagac
IP_V1-18*p07     ggacaagggcttgagtggatgggatggatcagcgcttacaatggtaacacaaactatgcacagaagctccagggcagagtcaccatgaccacagac
IP_V1-18*p08     ggacaagggcttgagtggatgggatggatcagcgcttacaatggtaacacaaactatgcacagaagctccagggcagagtcaccatgaccacagac
V_V1-18*01       ggacaagggcttgagtggatgggatggatcagcgcttacaatggtaacacaaactatgcacagaagctccagggcagagtcaccatgaccacagac

V1-18_01         acatccacgagcacagcctacatggagctgaggagcctgagatctgacgacacggccgtgtattactgtgcgaga--
V1-18_02         acatccacgagcacagcctacatggagctgaggagcctgagatctgacgacacggccgtgtattactgtgcgaga--
V1-18_03         acatccacgagcacagcctacatggagctgaggagcctgagatctgacgacacggccgtgtattactgtgcgaga--
V1-18_04         acatccacgagcacagcctacatggagctgaggagcctgagatctgacgacacggccgtgtattactgtgcgaga--
V1-18_05         acatccacgagcacagcctacatggagctgaggagcctgagatctgacgacacggccgtgtattactgtgcgaga--
IGHV1-18*01      acatccacgagcacagcctacatggagctgaggagcctgagatctgacgacacggccgtgtattactgtgcgagaga
IGHV1-18*02      acatccacgagcacagcctacatggagctgaggagcctaagatctgacgacacggcc--------------------
IGHV1-18*04      acatccacgagcacagcctacatggagctgaggagcctgagatctgacgacacggccgtgtattactgtgcgagaga
IGHV1-18*03      acatccacgagcacagcctacatggagctgaggagcctgagatctgacgacatggccgtgtattactgtgcgagaga
IP_V1-18*p03     acatccacgagcacagcctacatggagctgaggagcctaagatctgacgacacggccgtgtattactgtgcgagaga
IP_V1-18*p04     acatccacgagcacagcctacatggagctgaggagcctaagatctgacgacacggccgtgtattactgtgcgagaga
IP_V1-18*p05     acatccacgagcacagcctacatggagctgaggagcctgagatctgacgacacggccgtgtattactgtgcgagaga
IP_V1-18*p06     acatccacgagcacagcctacatggagctgaggagcctgagatctgacgacacggccgtgtattactgtgcgagaga
IP_V1-18*p07     acatccacgagcacagcctacatggagctgaggagcctgagatctgacgacacggccgtgtattactgtgcgagaga
IP_V1-18*p08     acatccacgagcacagcctacatggagctgaggagcctgagatctgacgacacggccgtgtattactgtgcgagaga
V_V1-18*01       acatccacgagcacagcctacatggagctgaggagcctgagatctgacgacacggccgtgtattactgtgcgaga--
```

IGHV1-24

```
V1-24_01        atggactgcacctggaggatcctcttcttggtggcagcagctacaggcacccacgcccaggtccagctggtacagtctggggctgaggtgaa
V1-24_02        atggactgcacctggaggatcctcctcttggtggcagcagctacaggcacccacgcccaggtccagctggtacagtctggggctgaggtgaa
V1-24_03        atggactgcacctggaggatcctcctcttggtggcagcagctacaggcacccacgcccaggtccagctggtacagtctggggctgaggtgaa
V1-24_04        atggactgcacctggaggatcctcttcttggtggcagcagctacaggcacccacgcccaggtccagctggtacagtctggggctgaggtgaa
V1-24_05        atggactgcacctggaggatcctcttcttggtggcagcagctacaggcacccacgtccaggtccagctggtacagtctggggctgaggtgaa
IGHV1-24*01     atggactgcacctggaggatcctcttcttggtggcagcagctacaggcacccacgcccaggtccagctggtacagtctggggctgaggtgaa
IP_V1-24*p02    ---------------------------------------------------------caggtccagctggtacagtctggggctgaggtgaa
IP_V1-24*p03    -------------------------------------------------------------------------------------aggtgaa
V_V1-24*01      ---------------------------------------------------------caggtccagctggtacagtctggggctgaggtgaa

V1-24_01        gaagcctggggcctcagtgaaggtctcctgcaaggtttccggatacaccctcactgaattatccatgcactgggtgcgacaggctcct
V1-24_02        gaagcctggggcctcagtgaaggtctcctgcaaggtttccggatacaccctcaccgaattatccaggcactgggtgcgacaggctccc
V1-24_03        gaagcctggggcctcagtgaaggtctcctgcaaggtttccggatacaccctcactgaattatccatgcactgggtgcgacaggctcct
V1-24_04        gaagcctggggcctcagtgaaggtctcctgcaaggtttccggatacaccctcactgaattatccatgcactgggtgcgacaggctcct
V1-24_05        gaagcctggggcctcagtgaaggtctcctgcaaggtttccggatacaccctcactgaattatccatgcactgggtgcgacaggctcct
IGHV1-24*01     gaagcctggggcctcagtgaaggtctcctgcaaggtttccggatacaccctcactgaattatccatgcactgggtgcgacaggctcct
IP_V1-24*p02    gaagcctggggcctcagtgaaggtctcctgcaaggtttccggatacaccctcactgaattatccatgcactgggtgtgacaggctcct
IP_V1-24*p03    gaagcctggggcctcagtgaaggtctcctgcaaggtttccggatacaccctcaccgaattatccaggcactgggtgcgacaggctccc
V_V1-24*01      gaagcctggggcctcagtgaaggtctcctgcaaggtttccggatacaccctcactgaattatccatgcactgggtgcgacaggctcct

V1-24_01        ggaaaagggcttgagtggatgggaggttttgatcctgaagatggtgaaacaatctacgcacagaagttccagggcagagtcaccatgaccg
V1-24_02        ggaaaagggcttgagtggatgggaggttttgatcctgaagatggtgaaacaatctacgcacagaagttccagggcagagtcaccatgaccg
V1-24_03        ggaaaagggcttgagtggatgggaggttttgatcctgaagatggtgaaacaatctacgcacagaagttccagggcagagtcaccatgaccg
V1-24_04        ggaaaagggcttgagtggatggggggttttgatcctgaagatggtgaaacaatctacgcacagaagttccagggcagagtcaccatgaccg
V1-24_05        ggaaaagggcttgagtggatgggaggttttgatcctgaagatggtgaaacaatctacgcacagaagttccagggcagagtcaccatgaccg
IGHV1-24*01     ggaaaagggcttgagtggatgggaggttttgatcctgaagatggtgaaacaatctacgcacagaagttccagggcagagtcaccatgaccg
IP_V1-24*p02    ggaaaagggcttgagtggatgggaggttttgatcctgaagatggtgaaacaatctacgcacagaagttccagggcagagtcaccatgaccg
IP_V1-24*p03    ggaaaagggcttgagtggatgggaggttttgatcctgaagatggtgaaacaatctacgcacagaagttccagggcagagtcaccatgaccg
V_V1-24*01      ggaaaagggcttgagtggatgggaggttttgatcctgaagatggtgaaacaatctacgcacagaagttccagggcagagtcaccatgaccg

V1-24_01        aggacacatctacagacacagcctacatggagctgagcagcctgagatctgaggacacggccgtgtattactgtgcaaca
V1-24_02        aggacacatctacagacacagcctacatggagctgagcagcctgagatctgaggacacggccgtgtattactgtgcaaca
V1-24_03        aggacacatctacagacacagcctacatggagctgagcagcctgagatctgaggacacggccgtgtattactgtgcaaca
V1-24_04        aggacacatctacagacacagcctacatggagctgagcagcctgagatctgaggacacggccgtgtattactgtgcaaca
V1-24_05        aggacacatctacagacacagcctacatggagctgagcagcctgagatctgaggacacggccgtgtattactgtgcaaca
IGHV1-24*01     aggacacatctacagacacagcctacatggagctgagcagcctgagatctgaggacacggccgtgtattactgtgcaaca
IP_V1-24*p02    aggacacatctacagacacagcctacatggagctgagcagcctgagatctgaggacacggccgtgtattactgtgcaaca
IP_V1-24*p03    aggacacatctacagacacagcctacatggagctgagcagcctgagatctgaggacacggccgtgtattactgtgcaaca
V_V1-24*01      aggacacatctacagacacagcctacatggagctgagcagcctgagatctgaggacacggccgtgtattactgtgcaaca
```

IGHV1-45

```
V1-45_01        atggactggacctggagaatcctcttcttggtggcagcagccacagaatgcctactcccagatgcagctggtgcagtctggggctgaggtga
V1-45_02        atggactggacctggagaatcctcttcttggtggcagcagccacagaatgcctactcccagatgcagctggtgcagtctggggctgaggtga
V1-45_03        atggactggacctggagaatcctcttcttggtggcagcagtcacagaatgcctactcccagatgcagctggtgcagtctggggctgaggtga
IGHV1-45*01     atggactggacctggagaatcctcttcttggtggcagcagccacagntgcctactcccagatgcagctggtgcagtctggggctgaggtga
IGHV1-45*02     atggactggacctggagaatcctcttcttggtggcagcagccacagaatgcctactcccagatgcagctggtgcagtctggggctgaggtga
IGHV1-45*03     -----------------aatcctcttcttggtggcagcagccacagaatgcctactcccagatgcagctggtgcagtctggggctgaggtga
V_V1-45*01      ----------------------------------------------------------cagatgcagctggtgcagtctggggctgaggtga
V_V1-45*02      ----------------------------------------------------------cagatgcagctggtgcagtctggggctgaggtga

V1-45_01        agaagactgggtcctcagtgaaggtttcctgcaaggcttccggatacaccttcacctaccgctacctgcactgggtgcgacaggcccccc
V1-45_02        agaagactgggtcctcagtgaaggtttcctgcaaggcttccggatacaccttcacctaccgctacctgcactgggtgcgacaggcccccc
V1-45_03        agaagactgggtcctcagtgaaggtttcctgcaaggcttccggatacaccttcacctaccgctacctgcactgggtgcgacaggcccccc
IGHV1-45*01     agaagactgggtcctcagtgaaggtttcctgcaaggcttccggatacaccttcacctaccgctacctgcactgggtgcgacaggcccccc
IGHV1-45*02     agaagactgggtcctcagtgaaggtttcctgcaaggcttccggatacaccttcacctaccgctacctgcactgggtgcgacaggcccccc
IGHV1-45*03     agaagactgggtcctcagtgaaggtttcctgcaaggcttccggatacaccttcacctaccgctacctgcactgggtgcgacaggcccccc
V_V1-45*01      agaagactgggtcctcagtgaaggtttcctgcaaggcttccggatacaccttcacctaccgctacctgcactgggtgcgacaggcccccc
V_V1-45*02      agaagactgggtcctcagtgaaggtttcctgcaaggcttccggatacaccttcacctaccgctacctgcactgggtgcgacaggcccccc

V1-45_01        ggacaagcgcttgagtggatgggatggatcacacctttcaatggtaacaccaactacgcacagaaattccaggacagagtcaccattaccagg
V1-45_02        agacaagcgcttgagtggatgggatggatcacacctttcaatggtaacaccaactacgcacagaaattccaggacagagtcaccattaccagg
V1-45_03        ggacaagcgcttgagtggatgggatggatcacacctttcaatggtaacaccaactacgcacagaaattccaggacagagtcaccattaccagg
IGHV1-45*01     ggacaagcgcttgagtggatgggatggatcacacctttcaatggtaacaccaactacgcacagaaattccaggacagagtcaccattactagg
IGHV1-45*02     ggacaagcgcttgagtggatgggatggatcacacctttcaatggtaacaccaactacgcacagaaattccaggacagagtcaccattaccagg
IGHV1-45*03     agacaagcgcttgagtggatgggatggatcacacctttcaatggtaacaccaactacgcacagaaattccaggacagagtcaccattaccagg
V_V1-45*01      ggacaagcgcttgagtggatgggatggatcacacctttcaatggtaacaccaactacgcacagaaattccaggacagagtcaccattactagg
V_V1-45*02      ggacaagcgcttgagtggatgggatggatcacacctttcaatggtaacaccaactacgcacagaaattccaggacagagtcaccattaccagg

V1-45_01        gacaggtctatgagcacagcctacatggagctgagcagcctgagatctgaggacacagccatgtattactgtgcaaga
V1-45_02        gacaggtctatgagcacagcctacatggagctgagcagcctgagatctgaggacacagccatgtattactgtgcaaga
V1-45_03        gacaggtctatgagcacagcctacatggagctgagcagcctgagatctgaggacacagccatgtattactgtgcaaga
IGHV1-45*01     gacaggtctatgagcacagcctacatggagctgagcagcctgagatctgaggacacagccatgtattactgtgcaaga
IGHV1-45*02     gacaggtctatgagcacagcctacatggagctgagcagcctgagatctgaggacacagccatgtattactgtgcaaga
IGHV1-45*03     gacaggtctatgagcacagcctacatggagctgagcagcctgagatctgaggacacagccatgtattactgtgcaaga
V_V1-45*01      gacaggtctatgagcacagcctacatggagctgagcagcctgagatctgaggacacagccatgtattactgtgcaaga
V_V1-45*02      gacaggtctatgagcacagcctacatggagctgagcagcctgagatctgaggacacagccatgtattactgtgcaaga
```

IGHV1-46

```
V1-46_01        atggactggacctggagggtcttctgcttgctggctgtagctccaggtgctcactcccaggtgcagctggtgcagtctggggctgaggtgaag
V1-46_02        atggactggacctggagggtcttctgcttgctggctgtagctccaggtgctcactcccaggtgcagctggtgcagtctggggctgaggtgaag
V1-46_03        atggactggacctggagggtcttctgcttgctggctgtagctccaggtgctcactcccaggtgcagctggtgcagtctggggctgaggtgaag
V1-46_04        atggactggacctggagggtcttctgcttgctggctgtagctccaggtgctcactcccaggtgcagctggtgcagtctggggctgaggtgaag
V1-46_05        atggactggacctggagggtcttctgcttgctggctgtagctccaggtgctcactcccaggtgcagctggtgcagtctggggctgaggtgaag
IGHV1-46*01     atggactggacctggagggtcttctgcttgctggctgtagctccaggtgctcactcccaggtgcagctggtgcagtctggggctgaggtgaag
IGHV1-46*02     atggactggacctggagggtcttctgcttgctggctgtagcaccaggtgcccactcccaggtgcagctggtgcagtctggggctgaggtgaag
IGHV1-46*03     atggactggacctggagggtcttctgcttgctggctgtagctccaggtgctcactcccaggtgcagctggtgcagtctggggctgaggtgaag
IGHV1-46*04     atggactggacctggagggtcttctgcttgctggctgtagctccaggtgctcactcccaggtgcagctggtgcagtctggggctgaggtgaag
IP_V1-46*p04    ---------------------------------------------------------------------------------------------
IP_V1-46*p04a   ---------------------------------------------------------------------------------------------
V_V1-46*01      ---------------------------------------------------------caggtgcagctggtgcagtctggggctgaggtgaag
V_V1-46*02      ---------------------------------------------------------caggtgcagctggtgcagtctggggctgaggtgaag
```

```
V1-46_01        aagcctggggcctcagtgaaggtttcctgcaaggcatctggatacaccttcaccagctactatatgcactgggtgcgacaggcccct
V1-46_02        aagcctggggcctcagtgaaggtttcctgcaaggcatctggatacaccttcaccagctactatatgcactgggtgcgacaggcccct
V1-46_03        aagcctggggcctcagtgaaggtttcctgcaaggcatctggatacaccttcaccagctactatatgcactgggtgcgacaggcccct
V1-46_04        aagcctggggcctcagtgaaggtttcctgcaaggcatctggatacaccttcaccagctactatatgcactgggtgcgacaggcccct
V1-46_05        aagcctggggcctcagtgaaggtttcctgcatggcatctggatacaccttcaccagctactatatgcactgggtgcgacaggcccct
IGHV1-46*01     aagcctggggcctcagtgaaggtttcctgcaaggcatctggatacaccttcaccagctactatatgcactgggtgcgacaggcccct
IGHV1-46*02     aagcctggggcctcagtgaaggtttcctgcaaggcatctggatacaccttcaacagctactatatgcactgggtgcgacaggcccct
IGHV1-46*03     aagcctggggcctcagtgaaggtttcctgcaaggcatctggatacaccttcaccagctactatatgcactgggtgcgacaggcccct
IGHV1-46*04     aagcctggggcctcagtgaaggtttcctgcaaggcatctggatacaccttcaccagctactatatgcactgggtgcgacaggcccct
IP_V1-46*p04    ---------------------------------------------------------------------------------------
IP_V1-46*p04a   ---------------------------------gcatctggatacaccttcaccagctactatatgcactgggtgcgacaggcccct
V_V1-46*01      aagcctggggcctcagtgaaggtttcctgcaaggcatctggatacaccttcaccagctactatatgcactgggtgcgacaggcccct
V_V1-46*02      aagcctggggcctcagtgaaggtttcctgcaaggcatctggatacaccttcaacagctactatatgcactgggtgcgacaggcccct
```

```
V1-46_01        ggacaagggcttgagtggatgggaataatcaaccctagtggtggtagcacaagctacgcacagaagttccagggcagagtcaccatgaccagg
V1-46_02        ggacaagggcttgagtggatgggaataatcaaccctagtggtggtagcacaagctacgcacagaagttccagggcagagtcaccatgaccagg
V1-46_03        ggacaagggcttgagtggatgggaataatcaaccctagtggtggtagcacaagctacgcacagaagttccagggcagagtcaccatgaccagg
V1-46_04        ggacaagggcttgagtggatgggaataatcaaccctagtggtggtagcacaagctacgcacagaagttgcagggcagagtcaccatgaccagg
V1-46_05        ggacaagggcttgagtggatgggaataatcaaccctagtggtggtagcacaagctacgcacagaagttccagggcagagtcaccatgaccagg
IGHV1-46*01     ggacaagggcttgagtggatgggaataatcaaccctagtggtggtagcacaagctacgcacagaagttccagggcagagtcaccatgaccagg
IGHV1-46*02     ggacaagggcttgagtggatgggaataatcaaccctagtggtggtagcacaagctacgcacagaagttccagggcagagtcaccatgaccagg
IGHV1-46*03     ggacaagggcttgagtggatgggaataatcaaccctagtggtggtagcacaagctacgcacagaagttccagggcagagtcaccatgaccagg
IGHV1-46*04     ggacaagggcttgagtggatgggaataatcaaccctagtggtggtagcacaagctacgcacagaagttgcagggcagagtcaccatgaccagg
IP_V1-46*p04    ------gggcttgagtggatgggaataatcaaccctagtggtggtagcacaagctacgcacagaagttgcagggcagagtcaccatgaccagg
IP_V1-46*p04a   ggacaagggcttgagtggatgggaataatcaaccctagtggtggtagcacaagctacgcacagaagttgcagggcagagtcaccatgaccagg
V_V1-46*01      ggacaagggcttgagtggatgggaataatcaaccctagtggtggtagcacaagctacgcacagaagttccagggcagagtcaccatgaccagg
V_V1-46*02      ggacaagggcttgagtggatgggaataatcaaccctagtggtggtagcacaagctacgcacagaagttccagggcagagtcaccatgaccagg
```

V1-46_01	gacacgtccacgagcacagtctacatggagctgagcagcctgagatctgaggacacggccgtgtattactgtgcgaga--
V1-46_02	gacacgtccacgagcacagtctacatggagctgagcagcctgagatctgaggacacggccgtgtattactgtgctaga--
V1-46_03	gacatgtccacgagcacagtctacatggagctgagcagcctgagatctgaggacacggccgtgtattactgtgcgaga--
V1-46_04	gacacgtccacgagcacagtctacatggagctgagcagcctgagatctgaggacacggccgtgtattactgtgcgaga--
V1-46_05	gacacgtccacgagcacagtctacatggagctgagcagcctgagatctgaggacacggccgtgtattactgtgcgaga--
IGHV1-46*01	gacacgtccacgagcacagtctacatggagctgagcagcctgagatctgaggacacggccgtgtattactgtgcgagaga
IGHV1-46*02	gacacgtccacgagcacagtctacatggagctgagcagcctgagatctgaggacacggccgtgtattactgtgcgagaga
IGHV1-46*03	gacacgtccacgagcacagtctacatggagctgagcagcctgagatctgaggacacggccgtgtattactgtgctagaga
IGHV1-46*04	gacacgtccacgagcacagtctacatggagctgagcagcctgagatctgaggacacggccgtgtattactgtgcgagaga
IP_V1-46*p04	gacacgtccacgagcacagtctacatggagctgagcagcctgagatctgaggacacggccgtgtattactgtgcgagaga
IP_V1-46*p04a	gacacgtccacgagcacagtctacatggagctgagcagcctgagatctgaggacacggccgtgtattactgtgcgagaga
V_V1-46*01	gacacgtccacgagcacagtctacatggagctgagcagcctgagatctgaggacacggccgtgtattactgtgcgaga--
V_V1-46*02	gacacgtccacgagcacagtctacatggagctgagcagcctgagatctgaggacacggccgtgtattactgtgcgaga--

IGHV1-58

```
V1-58_01       atggactggatttggaggatcctcttcttggtgggagcagcgacaggtgcccactcccaaatgcagctggtgcagtctgggcctgaggt
V1-58_02       atggactggatttggagggtcctcttcttggtgggagcagcgacaggtgcccactcccaaatgcagctggtgcagtctgggcctgaggt
V1-58_03       atggactggatttggaggatcctcttcttggtgggagcagcgacaggtgcccactcccaaatgcagctggtgcagtctgggcctgaggt
V1-58_04       atggactggatttggagggtcctcttcttggtgggagcagcgacaggtgcccactcccaaatgcagctggtgcagtctgggcctgaggt
V1-58_05       atggactggatttggaggatcctcttcttggtgggagcagcgacaggtgcccactcccaaatgcagctggtgcagtctgggcctgaggt
V1-58_06       atggactggatttggagggtcctcttcttggtgggagcagcgacaggtgcccactcccaaatgcagctggtgcagtctgggcctgaggt
V1-58_07       atggactggatttggaggatcctcttcttggtgggagcagcgacaggtgcccactcccaaatgcagctggtgcagtctgggcctgaggt
V1-58_08       atggactggatttggaggatcctcttcttggtgagagcagcgacaggtgcccactcccaaatgcagctggtgcagtctgggcctgaggt
V1-58_09       atggactggatttggaggatcctcttcttggtgagagcagcgacaggtgcccactcccaaatgcagctggtgcagtctgggcctgaggt
V1-58_10       atggactggatttggagggtcctcttcttggtgggagcagcgacaggtgcccactcccaaatgcagctggtgcagtctgggcctgaggt
V1-58_11       atggactggatttggagggtcctcttcttggtgagagcagcgacaggtgcccactcccaaatgcagctggtgcagtctgggcctgaggt
IGHV1-58*01    atggactggatttggaggatcctcttcttggtgggagcagcgacaggtgcccactcccaaatgcagctggtgcagtctgggcctgaggt
IGHV1-58*02    atggactggatttggagggtcctcttcttggtgggagcagcgacaggtgcccactcccaaatgcagctggtgcagtctgggcctgaggt
IGHV1-58*03    ----------------------------------------------------------caaatgcagctggtgcagtctgggcctgaagt
IP_V1-58*p03   ----------------------------------------------------------caaatgcagctggtgcagtctgggcctgaggt
IP_V1-58*p04   ----------------------------------------------------------caaatgcagctggtgcagtctgggcctgaagt
V_V1-58*01     ----------------------------------------------------------caaatgcagctggtgcagtctgggcctgaggt
V_V1-58*02     ----------------------------------------------------------caaatgcagctggtgcagtctgggcctgaggt
```

```
V1-58_01       gaagaagcctgggacctcagtgaaggtctcctgcaaggcttctggattcacctttactagctctgctgtgcagtgggtgcgacaggctcgt
V1-58_02       gaagaagcctgggacctcagtgaaggtctcctgcaaggcttctggattcacctttactagctctgctatgcagtgggtgcgacaggctcgt
V1-58_03       gaagaagcctgggacctcagtgaaggtctcctgcaaggcttctggattcacctttactagctctgctatgcagtgggtgcgacaggctcgt
V1-58_04       gaagaagcctgggacctcagtgaaggtctcctgcaaggcttctggattcacctttactagctctgctgtgcagtgggtgcgacaggctcgt
V1-58_05       gaagaagcctgggacctcagtgaagttctcctgcaaggcttctggattcacctttactagctctgctatgcagtgggtgcgacaggctcgt
V1-58_06       gaagaagcctgggacctcagtgaagttctcctgcaaggcttctggattcacctttactagctctgctatgcagtgggtgcgacaggctcgt
V1-58_07       gaagaagcctgggacctcagtgaagttctcctgcaaggcttctggattcacctttactagctctgctgtgcagtgggtgcgacaggctcgt
V1-58_08       gaagaagcctgggacctcagtgaaggtctcctgcaaggcttctggattcacctttactagctctgctgtgcagtgggtgcgacaggctcgt
V1-58_09       gaagaagcctgggacctcagtgaagttctcctgcaaggcttctggattcacctttactagctctgctgtgcagtgggtgcgacaggctcgt
V1-58_10       gaagaagcctgggacctcagtgaaggtctcctgcaaggcttctggattcacctttactagctctgctatgcagtgggtgcgacaggctcgt
V1-58_11       gaagaagcctgggacctcagtgaaggtctcctgcaaggcttctggattcacctttactagctctgctgtgcagtgggtgcgacaggctcgt
IGHV1-58*01    gaagaagcctgggacctcagtgaaggtctcctgcaaggcttctggattcacctttactagctctgctgtgcagtgggtgcgacaggctcgt
IGHV1-58*02    gaagaagcctgggacctcagtgaaggtctcctgcaaggcttctggattcacctttactagctctgctatgcagtgggtgcgacaggctcgt
IGHV1-58*03    gaagaagcctgggacctcagtgaaggtctcctgcaaggcttctggattcacctttactagctctgctgtgcagtgggtgcgacaggctcgt
IP_V1-58*p03   gaagaagcctgggacctcagtgaagttctcctgcaaggcttctggattcacctttactagctctgctatgcagtgggtgcgacaggctcgt
IP_V1-58*p04   gaagaagcctgggacctcagtgaaggtctcctgcaaggcttctggattcacctttactagctctgctgtgcagtgggtgcgacaggctcgt
V_V1-58*01     gaagaagcctgggacctcagtgaaggtctcctgcaaggcttctggattcacctttactagctctgctgtgcagtgggtgcgacaggctcgt
V_V1-58*02     gaagaagcctgggacctcagtgaaggtctcctgcaaggcttctggattcacctttactagctctgctatgcagtgggtgcgacaggctcgt
```

```
V1-58_01        ggacaacgccttgagtggataggatggatcgtcgttggcagtggtaacacaaactacgcacagaagttccaggaaagagtcaccattaccagg
V1-58_02        ggacaacgccttgagtggataggatggatcgtcgttggcagtggtaacacaaactacgcacagaagttccaggaaagagtcaccattaccagg
V1-58_03        ggacaacgccttgagtggataggatggatcgtcgttggcagtggtaacacaaactacgcacagaagttccaggaaagagtcaccattaccagg
V1-58_04        ggacaacgccttgagtggataggatggatcgtcgttggcagtggtaacacaaactacgcacagaagttccaggaaagagtcaccattaccagg
V1-58_05        ggacaacgccttgagtggataggatggatcgtcgttggcagtggtaacacaaactacgcacagaagttccaggaaagagtcaccattaccagg
V1-58_06        ggacaacgccttgagtggataggatggatcgtcgttggcagtggtaacacaaactacgcacagaagttccaggaaagagtcaccattaccagg
V1-58_07        ggacaacgccttgagtggataggatggatcgtcgttggcagtggtaacacaaactacgcacagaagttccaggaaagagtcaccattaccagg
V1-58_08        ggacaacgccttgagtggataggatggatcgtcgttggcagtggtaacacaaactacgcacagaagttccaggaaagagtcaccattaccagg
V1-58_09        ggacaacgccttgagtggataggatggatcgtcgttggcagtggtaacacaaactacgcacagaagttccaggaaagagtcaccattaccagg
V1-58_10        ggacaacgccttgagtggataggatggatcgtcgttggcagtggtaacacaaactacgcacagaagttccaggaaagagtcaccattaccagg
V1-58_11        ggacaacgccttgagtggataggatggatcgtcgttggcagtggtaacacaaactacgcacagaagttccaggaaagagtcaccattaccagg
IGHV1-58*01     ggacaacgccttgagtggataggatggatcgtcgttggcagtggtaacacaaactacgcacagaagttccaggaaagagtcaccattaccagg
IGHV1-58*02     ggacaacgccttgagtggataggatggatcgtcgttggcagtggtaacacaaactacgcacagaagttccaggaaagagtcaccattaccagg
IGHV1-58*03     ggacaacgccttgagtggataggatggatcgtcgttggcagtggtaacacaaactacgcacagaagttccaggaaagagtcaccattaccagg
IP_V1-58*p03    ggacaacgccttgagtggataggatggatcgtcgttggcagtggtaacacaaactacgcacagaagttccaggaaagagtcaccattaccagg
IP_V1-58*p04    ggacaacgccttgagtggataggatggatcgtcgttggcagtggtaacacaaactacgcacagaagttccaggaaagagtcaccattaccagg
V_V1-58*01      ggacaacgccttgagtggataggatggatcgtcgttggcagtggtaacacaaactacgcacagaagttccaggaaagagtcaccattaccagg
V_V1-58*02      ggacaacgccttgagtggataggatggatcgtcgttggcagtggtaacacaaactacgcacagaagttccaggaaagagtcaccattaccagg
```

```
V1-58_01        gacatgtccacaagcacagcctacatggagctgagcagcctgagatccgaggacacggccgtgtattactgtgcggca
V1-58_02        gacatgtccacaagcacagcctacatggagctgagcagcctgagatccgaggacacggccgtgtattactgtgcggca
V1-58_03        gacatgtccacaagcacagcctacatggagctgagcagcctgagatccgaggacacggccgtgtattactgtgcggca
V1-58_04        gacatgtccacaagcacagcctacatggagctgagcagcctgagatccgaggacacggccgtgtattactgtgcggca
V1-58_05        gacatgtccacaagcacagcctacatggagctgagcagcctgagatccgaggacacggccgtgtattactgtgcggca
V1-58_06        gacatgtccacaagcacagcctacatggagctgagcagcctgagatccgaggacacggccgtgtattactgtgcggca
V1-58_07        gacatgtccacaagcacagcctacatggagctgagcagcctgagatccgaggacacggccgtgtattactgtgcggca
V1-58_08        gacatgtccacaagcacagcctacatggagctgagcagcctgagatccgaggacacggccgtgtattactgtgcggca
V1-58_09        gacatgtccacaagcacagcctacatggagctgagcagcctgagatccgaggacacggccgtgtattactgtgcggca
V1-58_10        gacatgtccacaagcacagcctacatggagctgagcagcctgagatccgaggacatggccgtgtattactgtgcggca
V1-58_11        gacatgtccacaagcacagcctacatggagctgagcagcctgagatccgaggacacggccgtgtattactgtgcggca
IGHV1-58*01     gacatgtccacaagcacagcctacatggagctgagcagcctgagatccgaggacacggccgtgtattactgtgcggca
IGHV1-58*02     gacatgtccacaagcacagcctacatggagctgagcagcctgagatccgaggacacggccgtgtattactgtgcggca
IGHV1-58*03     gacatgtccacaagcacagcctacatggagctgagcagcctgagatccgaggacacggccgtgtattactgtgcggca
IP_V1-58*p03    gacatgtccacaagcacagcctacatggagctgagcagcctgagatccgaggacacggccgtgtattactgtgcggca
IP_V1-58*p04    gacatgtccacaagcacagcctacatggagctgagcagcctgagatccgaggacacggccgtgtattactgtgcggca
V_V1-58*01      gacatgtccacaagcacagcctacatggagctgagcagcctgagatccgaggacacggccgtgtattactgtgcggca
V_V1-58*02      gacatgtccacaagcacagcctacatggagctgagcagcctgagatccgaggacacggccgtgtattactgtgcggca
```

IGHV1-69

V1-69_01	atggactggacctggaggttcctctttgtggtggcagcagcagctacaggtgtccagtcccaggtgcagctggtgcagtctggggctgagg
V1-69_02	atggactggacctggaggttcctctttgtggtggcagcagcagctacaggtgtccagtcccaggtccagctggtgcagtctggggctgagg
V1-69_03	atggactggacctggaggttcctctttgtggtggcagcagcagctacaggtgtccagtcccaggtgcagctggtgcagtctggggctgagg
V1-69_04	atggactggacctggaggttcctctttgtggtggcagcagcagctacaggtgtccagtcccaggtccagctggtgcagtctggggctgagg
V1-69_05	atggactggacctggaggttcctctttgtggtggcagcagcagctacaggtgtccagtcccaggtccagctggtgcagtctggggctgagg
V1-69_06	atggactggacctggaggttcctctttgtggtggcagcagcagctacaggtgtccagtcccaggtccagctggtgcagtctggggctgagg
V1-69_07	atggactggacctggaggttcctctttgtggtggcagcagcagctacaggtgtccagtcccaggtccagctggtgcagtctggggctgagg
V1-69_08	atggactggacctggaggttcctctttgtggtggcagcagcagctacaggtgtccagtcccaggtccagctggtgcagtctggggctgagg
V1-69_09	atggactggacctggaggttcctctttgtggtggcagcagcagctacaggtgtccagtcccaggtccagctggtgcagtctggggctgagg
V1-69_10	atggactggacctggaggttcctctttgtggtggcagcagcagctacaggtgtccagtcccaggtccagctggtgcaatctggggctgagg
V1-69_11	atggactggacctggaggttcctctttgtggtggcagcagcagctacaggtgtccagtcccaggtccagctggtgcagtctggggctgagg
V1-69_12	atggactggacctggaggttcctctttgtggtggcagcagcagctacaggtgtccagtcccaggtccagctggtgcagtctggggctgagg
V1-69_13	atggactggacctggaggttcctctttgtggtggcagcagcagctacaggtgtccagtcccaggtgcagctggtgcagtctggggctgagg
V1-69_14	atggactggacctggaggttcctctttgtggtggcagcagcagctacaggtgtccagtcccaggtccagctggtgcagtctggggctgagg
V1-69_15	atggactggacctggaggttcctctttgtggtggcagcagcagctacaggtgtccagtcccaggtccagctggtgcagtctggggctgagg
V1-69_16	atggactggacctggaggttcctctttgtggtggcagcagcagctacaggtgtccagtcccaggtccagctggtgcagtctggggctgagg
V1-69_17	atggactggacctggaggttcctctttgtggtggcagcagcagctacaggtgtccagtcccaggtccagctggtgcagtctggggctgagg
V1-69_18	atggactggacctggaggttcctctttgtggtggcagcagcagctacaggtgtccagtcccaggtccagctggtgcaatctggggctgagg
V1-69_19	atggactggacctggaggttcctctttgtggtggcagcagcagctacaggtgtccagtcccaggtccagctggtgcagtctggggctgagg
V1-69_20	atggactggacctggaggttcctctttgtggtggcagcagcagctacaggtgtccagtcccaggtccagctggtgcaatctggggctgagg
V1-69_21	atggactggacctggaggttcctctttgtggtggcagcagcagctacaggtgtccagtcccaggtgcagctggtgcaatctggggctgagg
V1-69_22	atggactggacctggaggttcctctttgtggtggcagcagcagctacaggtgtccagtcccaggtccagctggtgcagtctggggctgagg
V1-69_23	atggactggacctggaggttcctctttgtggtggcagcagcagctacaggtgtccagtcccaggtccagctggtgcagtctggggctgagg
V1-69_24	atggactggacctggaggttcctctttgtggtggcagcagcagctacaggtgtccagtcccaggtccagctggtgcagtctggggctgagg
V1-69_25	atggactggacctggaggttcctctttgtggtggcagcagcagctacaggtgtccagtcccaggtccagctggtgcagtctggggctgagg
V1-69_26	atggactggacctggaggttcctctttgtggtggcagcagcagctacaggtgtccagtcccaggtccagctggtgcagtctggggctgagg
V1-69_27	atggactggacctggaggttcctctttgtggtggcagcagcagctacaggtgtccagtcccaggtccagctggtgcagtctggggctgagg
V1-69_28	atggactggacctggaggttcctctttgtggtggcagcagcagctacaggtgtccagtcccaggtgcagctggtgcagtctggggctgagg
V1-69_29	atggactggacctggaggttcctctttgtggtggcagcagcagctacaggtgtccagtcccaggtccagctggtgcaatctggggctgagg
V1-69_30	atggactggacctggaggttcctctttgtggtggcagcagcagctacaggtgtccagtcccaggtgcagctggtgcagtctggggctgagg
V1-69_32	atggactggacctggaggttcctctttgtggtggcagcagcagctacaggtgtccagtcccaggtccagctggtgcagtctggggctgagg
V1-69_33	atggactggacctggaggttcctctttgtggtggcagcagcagctacaggtgtccagtcccaggtccagctggtgcagtctggggctgagg
IGHV1-69*01	atggactggacctggaggttcctctttgtggtg---gcagcagctacaggtgtccagtcccaggtgcagctggtgcagtctggggctgagg
IGHV1-69*03	-----------ctggaggttcctctttgtggtg---gcagcagctacaggtgtccactcccaggtgcagctggtgcagtctggggctgagg
IGHV1-69*04	atggactggacctggaggttcctctttgtggtg---gcagcagctacaggtgtccagtcccaggtccagctggtgcagtctggggctgagg
IGHV1-69*06	atggactggacctggaggttcctctttgtggtg---gcagcagctacaggtgtccagtcccaggtgcagctggtgcagtctggggctgagg
IGHV1-69*14	atggactggacctggaggttcctctttgtggtg---gcagcagctacaggtgtccagtcccaggtccagctggtgcagtctggggctgagg
IGHV1-69*15	-----------------gttcctctttgtggtg---gcagcagctacaggtgtccagttccaggtccagctggtgcagtctggggctgagg
IGHV1-69*16	-----------------gttcctctttgtggtg---gcagcagctacaggtgtccagtcccaggtccagctggtgcagtctggggctgagg
IGHV1-69*17	atggactggacctggaggttcctctttgtggtg---gcagcagctacaggtgtccagtcccaggtgcagctggtgcagtctggggctgagg
IGHV1-69*02	--caggtccagctggtgcaatctggggctgagg
IGHV1-69*05	--caggtccagctggtgcagtctggggctgagg
IGHV1-69*07	---
IGHV1-69*08	--caggtccagctggtgcaatctggggctgagg

```
IGHV1-69*09      ------------------------------------------------------------caggtgcagctggtgcagtctggggctgagg
IGHV1-69*10      ------------------------------------------------------------caggtccagctggtgcagtctggggctgagg
IGHV1-69*11      ------------------------------------------------------------caggtccagctggtgcagtctggggctgagg
IGHV1-69*12      ------------------------------------------------------------caggtccagctggtgcagtctggggctgagg
IGHV1-69*13      ------------------------------------------------------------caggtccagctggtgcagtctggggctgagg
IGHV1-69D*01     atggactggacctggaggttcctctttgtggtg---gcagcagctacaggtgtccagtcccaggtgcagctggtgcagtctggggctgagg
IP_V1-69-2*p03   -----------------------------------------------------------------------------------------gg
IP_V1-69-2*p04   ----------------------------------------------------------------------------------------agg
IP_V1-69*p14     -------------------------------------------------------------------------------------------
IP_V1-69*p18     ------------------------------------------------------------caggtccagctggtgcagtctggggctgagg
IP_V1-69*p19     ------------------------------------------------------------caggtccagctggtgcagtctggggctgagg
IP_V1-69*p19     -----------------------------------------------------------------------------------------gg
IP_V1-69*p20     ------------------------------------------------------------caggtccagctggtgcagtctggggctgagg
IP_V1-69*p21     ------------------------------------------------------------caggtgcagctggtgcagtctggggctgagg
IP_V1-69*p25     ------------------------------------------------------------caggtgcagctggtgcagtctggggctgagg
IP_V1-69*p26     ------------------------------------------------------------caggtgcagctggtgcagtctggggctgagg
IP_V1-69*p27     -----------------------------------------------------------------------------------------gg
IP_V1-69*p28     -----------------------------------------------------------------------------------------gg
IP_V1-69*p29     ----------------------------------------------------------------------------------------agg
IP_V1-69*p30     ----------------------------------------------------------------------------------------agg
IP_V1-69*p31     -----------------------------------------------------------------------------------------gg
IP_V1-69*p32     -----------------------------------------------------------------------------------------gg
IP_V1-69*p33     ----------------------------------------------------------------------------------------agg
IP_V1-69*p34     ------------------------------------------------------------caggtccagctggtgcaatctggggctgagg
IP_V1-69*p35     ------------------------------------------------------------caggtccagctggtgcagtctggggctgagg
IP_V1-69*p36     ------------------------------------------------------------caggtccagctggtgcagtctggggctgagg
IP_V1-69*p37     ------------------------------------------------------------caggtccagctggtgcagtctggggctgagg
IP_V1-69*p38     ------------------------------------------------------------caggtccagctggtgcagtctggggctgagg
V_V1-69*01       ------------------------------------------------------------caggtgcagctggtgcagtctggggctgagg
V_V1-69*04       ------------------------------------------------------------caggtccagctggtgcagtctggggctgagg
V_V1-69*06       ------------------------------------------------------------caggtgcagctggtgcagtctggggctgagg
```

```
V1-69_01         tgaagaagcctgggtcctcggtgaaggtctcctgcaaggcttctggaggcaccttcagcagctatgctatcagctgggtgcgacaggcc
V1-69_02         tgaagaagcctgggtcctcggtgaaggtctcctgcaaggcttctggaggcaccttcagcagctatgctatcagctgggtgcgacaggcc
V1-69_03         tgaagaagcctgggtcctcggtgaaggtctcctgcaaggcttctggaggcaccttcagcagctatgctatcagctgggtgcgacaggcc
V1-69_04         tgaagaagcctgggtcctcggtgaaggtctcctgcaaggcttctggaggcaccttcagcagctatgctatcagctgggtgcgacaggcc
V1-69_05         tgaagaagcctgggtcctcggtgaaggtctcctgcaaggcttctggaggcaccttcagcagctatgctatcagctgggtgcgacaggcc
V1-69_06         tgaagaagcctgggtcctcggtgaaggtctcctgcaaggcttctggaggcaccttcagcagctatgctatcagctgggtgcgacaggcc
V1-69_07         tgaagaagcctgggtcctcagtgaaggtctcctgcaaggcttctggaggcaccttcagcagctatgctatcagctgggtgcgacaggcc
V1-69_08         tgaagaagcctgggtcctcggtgaaggtctcctgcaaggcttctggaggcaccttcagcagctatgctatcagctgggtgcgacaggcc
V1-69_09         tgaagaagcctgggtcctcggtgaaggtctcctgcaaggcttctggaggcaccttcagcagctatgctatcagctgggtgcgacaggcc
V1-69_10         tgaagaagcctgggtcctcggtgaaggtctcctgcaaggcttctggaggcaccttcagcagctatgctatcagctgggtgcgacaggcc
V1-69_11         tgaagaagcctgggtcctcagtgaaggtctcctgcaaggcttctggaggcaccttcagcagctatgctatcagctgggtgcgacaggcc
V1-69_12         tgaagaagcctgggtcctcggtgaaggtctcctgcaaggcttctggaggcaccttcagcagctatgctatcagctgggtgcgacaggcc
V1-69_13         tgaagaagcctgggtcctcggtgaaggtctcctgcaaggcttctggaggcaccttcagcagctatgctatcagctgggtgcgacaggcc
V1-69_14         tgaagaagcctggatcctcagtgaaggtctcctgcaaggcttctggaggcaccttcagcagctatgctatcagctgggtgcgacaggcc
```

```
V1-69_15         tgaagaagcctggatcctcagtgaaggtctcctgcaaggcttctggaggcaccttcagcagctatgctatcagctgggtgcgacaggcc
V1-69_16         tgaagaagcctgggtcctcggtgaaggtctcctgcaaggcttctggaggcaccttcagcagctatgctatcagctgggtgcgacaggcc
V1-69_17         tgaagaagcctgggtcctcggtgaaggtctcctgcaaggcttctggaggcaccttcagcagctatgctatcagctgggtgcgacaggcc
V1-69_18         tgaagaagcctgggtcctcggtgaaggtctcctgcaaggcttctggaggcaccttcagcagctatgctatcagctgggtgcgacaggcc
V1-69_19         tgaagaagcctgggtcctcagtgaaggtctcctgcaaggcttctggaggcaccttcagcagctatgctatcagctgggtgcgacaggcc
V1-69_20         tgaagaagcctgggtcctcagtgaaggtctcctgcaaggcttctggaggcaccttcagcagctatgctatcagctgggtgcgacaggcc
V1-69_21         tgaagaagcctgggtcctcggtgaaggtctcctgcaaggcttctggaggcaccttcagcagctatgctatcagctgggtgcgacaggcc
V1-69_22         tgaagaagcctgggtcctcagtgaaggtctcctgcaaggcttctggaggcaccttcagcagctatgctatcagctgggtgcgacaggcc
V1-69_23         tgaagaagcctgggtcctcagtgaaggtctcctgcaaggcttctggaggcaccttcagcagctatgctatcagctgggtgcgacaggcc
V1-69_24         tgaagaagcctgggtcctcggtgaaggtctcctgcaaggcttctggaggcaccttcagcagctatgctatcagctgggtgcgacaggcc
V1-69_25         tgaagaagcctgggtcctcagtgaaggtctcctgcaaggcttctggaggcaccttcagcagctatgctatcagctgggtgcgacaggcc
V1-69_26         tgaagaagcctgggtcctcggtgaaggtctcctgcaaggcttctggaggcaccttcagcagctatgctatcagctgggtgcgacaggcc
V1-69_27         tgaagaagcctgggtcctcggtgaaggtctcctgcaaggcttctggaggcacctccagcagctatgctatcagctgggtgcgacaggcc
V1-69_28         tgaagaagcctgggtcctcggtgaaggtctcctgcaaggcttctggaggcaccttcagcagctatgctatcagctgggtgcgacaggcc
V1-69_29         tgaagaagcctgggtcctcggtgaaggtctcctgcaaggcttctggaggcaccttcagcagctatgctatcagctgggtgcgacaggcc
V1-69_30         tgaagaagcctgggtcctcagtgaaggtctcctgcaaggcttctggaggcaccttcagcagctatgctatcagctgggtgcgacaggcc
V1-69_32         tgaagaagcctgggtcctcagtgaaggtctcctgcaaggcttctggaggcaccttcagcagctatgctatcagctgggtgcgacaggcc
V1-69_33         tgaagaagcctgggtcctcagtgaaggtctcctgcaaggcttctggaggcaccttcagcagctatgctatcagctgggtgcgacaggcc
IGHV1-69*01      tgaagaagcctgggtcctcggtgaaggtctcctgcaaggcttctggaggcaccttcagcagctatgctatcagctgggtgcgacaggcc
IGHV1-69*03      tgaagaagcctgggtcctcggtgaaggtctcctgcaaggcttctggaggcaccttcagcagctatgctatcagctgggtgcgacaggcc
IGHV1-69*04      tgaagaagcctgggtcctcggtgaaggtctcctgcaaggcttctggaggcaccttcagcagctatgctatcagctgggtgcgacaggcc
IGHV1-69*06      tgaagaagcctgggtcctcggtgaaggtctcctgcaaggcttctggaggcaccttcagcagctatgctatcagctgggtgcgacaggcc
IGHV1-69*14      tgaagaagcctgggtcctcggtgaaggtctcctgcaaggcttctggaggcaccttcagcagctatgctatcagctgggtgcgacaggcc
IGHV1-69*15      tgaagaagcctgggtcctcggtgaaggtctcctgcaaggcttctggaggcaccttcagcagctatgctatcagctgggtgcgacaggcc
IGHV1-69*16      tgaagaagcctgggtcctcggtgaaggtctcctgcaaggcttctggaggcaccttcagcagctatactatcagctgggtgcgacaggcc
IGHV1-69*17      tgaagaagcctgggtcctcggtgaaggtctcctgcaaggcttctggaggcaccttcagcagctatgctatcagctgggtgcgacaggcc
IGHV1-69*02      tgaagaagcctgggtcctcggtgaaggtctcctgcaaggcttctggaggcaccttcagcagctatactatcagctgggtgcgacaggcc
IGHV1-69*05      tgaagaagcctgggtcctcggtgaaggtctcctgcaaggcttctggaggcaccttcagcagctatgctatcagctgggtgcgacaggcc
IGHV1-69*07      ---agaagcctgggtcctcggtgaaggtctcctgcaaggcttctggaggcaccttcagcagctatgctatcagctgggtgcgacaggcc
IGHV1-69*08      tgaagaagcctgggtcctcggtgaaggtctcctgcaaggcttctggaggcaccttcagcagctatactatcagctgggtgcgacaggcc
IGHV1-69*09      tgaagaagcctgggtcctcggtgaaggtctcctgcaaggcttctggaggcaccttcagcagctatgctatcagctgggtgcgacaggcc
IGHV1-69*10      tgaagaagcctgggtcctcagtgaaggtctcctgcaaggcttctggaggcaccttcagcagctatgctatcagctgggtgcgacaggcc
IGHV1-69*11      tgaagaagcctgggtcctcggtgaaggtctcctgcaaggcttctggaggcaccttcagcagctatgctatcagctgggtgcgacaggcc
IGHV1-69*12      tgaagaagcctgggtcctcggtgaaggtctcctgcaaggcttctggaggcaccttcagcagctatgctatcagctgggtgcgacaggcc
IGHV1-69*13      tgaagaagcctgggtcctcagtgaaggtctcctgcaaggcttctggaggcaccttcagcagctatgctatcagctgggtgcgacaggcc
IGHV1-69D*01     tgaagaagcctgggtcctcggtgaaggtctcctgcaaggcttctggaggcaccttcagcagctatgctatcagctgggtgcgacaggcc
IP_V1-69-2*p03   tgaagaagcctggggccaccgtgaaaatctcctgcaaggcttctggatacaccttcaccgactactacatgcactgggtgcaacaggcc
IP_V1-69-2*p04   tgaagaagcctggggccaccgtgaaaatctcctgcaaggcttctggatacaccttcaccgactactacatgcactgggtgcaacaggcc
IP_V1-69*p14     --------------------------------------gcttctggaggcaccttcagcagctatgctatcagctgggtgcgacaggcc
IP_V1-69*p18     tgaagaagcctgggtcctcggtgaaggtctcctgcaaggcttctggaggcaccttcagcagctatgctatcagctgggtgcgacaggcc
IP_V1-69*p19     tgaagaagcctgggtcctcagtgaaggtctcctgcaaggcttctggaggcaccttcagcagctatgctatcagctgggtgcgacaggcc
IP_V1-69*p19     tgaagaagcctgggtcctcagtgaaggtctcctgcaaggcttctggaggcaccttcagcagctatgctatcagctgggtgcgacaggcc
IP_V1-69*p20     tgaagaagcctgggtcctcggtgaaggtctcctgcaaggcttctggaggcaccttcagcagctatgctatcagctgggtgcgacaggcc
IP_V1-69*p21     tgaagaagcctgggtcctcggtgaaggtctcctgcaaggcttctggaggcaccttcagcagctatgctatcagctgggtgcgacaggcc
IP_V1-69*p25     tgaagaagcctgggtcctcggtgaaggtctcctgcaaggcttctggaggcaccttcagcagctatgctatcagctgggtgcgacaggcc
IP_V1-69*p26     tgaagaagcctgggtcctcggtgaaggtctcctgcaaggcttctggaggcaccttcagcagctatactatcagctgggtgcgacaggcc
IP_V1-69*p27     tgaagaagcctgggtcctcggtgaaggtctcctgcaaggcttctggaggcaccttcagcagctatactatcagctgggtgcgacaggcc
```

IP_V1-69*p28	tgaagaagcctgggtcctcggtgaaggtctcctgcaaggcttctggaggcaccttcagcagctatactatcagctgggtgcgacaggcc
IP_V1-69*p29	tgaagaagcctgggtcctcggtgaaggtctcctgcaaggcttctggaggcaccttcagcagctatgctatcagctgggtgcgacaggcc
IP_V1-69*p30	tgaagaagcctgggtcctcggtgaaggtctcctgcaaggcttctggaggcaccttcagcagctatgctatcagctgggtgcgacaggcc
IP_V1-69*p31	tgaagaagcctgggtcctcggtgaaggtctcctgcaaggcttctggaggcaccttcagcagctatgctatcagctgggtgcgacaggcc
IP_V1-69*p32	tgaagaagcctgggtcctcggtgaaggtctcctgcaaggcttctggaggcaccttcagcagctatgctatcagctgggtgcgacaggcc
IP_V1-69*p33	tgaagaagcctgggtcctcagtgaaggtctcctgcaaggcttctggaggcaccttcagcagctatgctatcagctgggtgcgacaggcc
IP_V1-69*p34	tgaagaagcctgggtcctcggtgaaggtctcctgcaaggcttctggaggcaccttcagcagctatactatcagctgggtgcgacaggcc
IP_V1-69*p35	tgaagaagcctgggtcctcggtgaaggtctcctgcaaggcttctggaggcaccttcagcagctatgctatcagctgggtgcgacaggcc
IP_V1-69*p36	tgaagaagcctgggtcctcggtgaaggtctcctgcaaggcttctggaggcaccttcagcagctatgctatcagctgggtgcgacaggcc
IP_V1-69*p37	tgaagaagcctgggtcctcagtgaaggtctcctgcaaggcttctggaggcaccttcagcagctatgctatcagctgggtgcgacaggcc
IP_V1-69*p38	tgaagaagcctggatcctcagtgaaggtctcctgcaaggcttctggaggcaccttcagcagctatgctatcagctgggtgcgacaggcc
V_V1-69*01	tgaagaagcctgggtcctcggtgaaggtctcctgcaaggcttctggaggcaccttcagcagctatgctatcagctgggtgcgacaggcc
V_V1-69*04	tgaagaagcctgggtcctcggtgaaggtctcctgcaaggcttctggaggcaccttcagcagctatgctatcagctgggtgcgacaggcc
V_V1-69*06	tgaagaagcctgggtcctcggtgaaggtctcctgcaaggcttctggaggcaccttcagcagctatgctatcagctgggtgcgacaggcc

V1-69_01	cctggacaagggcttgagtggatgggagggatcatccctatctttggtacagcaaactacgcacagaagttccagggcagagtcacgattac
V1-69_02	cctggacaagggcttgagtggatgggagggatcatccctatctttggtacagcaaactacgcacagaagttccagggcagagtcacgattac
V1-69_03	cctggacaagggcttgagtggatgggagggatcatccctatctttggtacagcaaactacgcacagaagttccagggcagagtcacgattac
V1-69_04	cctggacaagggcttgagtggatgggaaggatcatccctatctttggtacagcaaactacgcacagaagttccagggcagagtcacgattac
V1-69_05	cctggacaagggcttgagtggatgggaaggatcatccctatccttggtacagcaaactacgcacagaagttccagggcagagtcacgattac
V1-69_06	cctggacaagggcttgagtggatgggagggatcatccctatctttggtacagcaaactacgcacagaagttccagggcagagtcacgattac
V1-69_07	cctggacaagggcttgagtggatgggagggatcatccctatctttggtacagcaaactacgcacagaagttccagggcagagtcacgattac
V1-69_08	cctggacaagggcttgagtggatgggagggatcatccctatctttggtacagcaaactacgcacagaagttccagggcagagtcacgattac
V1-69_09	cctggacaagggcttgagtggatgggaaggatcatccctatccttggtacagcaaactacgcacagaagttccagggcagagtcacgattac
V1-69_10	cctggacaagggcttgagtggatgggaaggatcatccctatccttggtacagcaaactacgcacagaagttccagggcagagtcacgattac
V1-69_11	cctggacaagggcttgagtggatgggagggatcatccctatccttggtacagcaaactacgcacagaagttccagggcagagtcacgattac
V1-69_12	cctggacaagggcttgagtggatgggagggatcatccctatccttggtacagcaaactacgcacagaagttccagggcagagtcacgattac
V1-69_13	cctggacaagggcttgagtggatgggaaggatcatccctatccttggtacagcaaactacgcacagaagttccagggcagagtcacgattac
V1-69_14	cctggacaagggcttgagtggatgggagggatcatccctatccttggtacagcaaactacgcacagaagttccagggcagagtcacgattac
V1-69_15	cctggacaagggcttgagtggatgggagggatcatccctatctttggtacagcaaactacgcacagaagttccagggcagagtcacgattac
V1-69_16	cctggacaagggcttgagtggatgggaaggatcatccctatctttggtacagcaaactacgcacagaagttccagggcagagtcacgattac
V1-69_17	cctggacaagggcttgagtggatgggaaggatcatccctatctttggtacagcaaactacgcacagaagttccagggcagagtcacgattac
V1-69_18	cctggacaagggcttgagtggatgggaaggatcatccctatctttggtacagcaaactacgcacagaagttccagggcagagtcacgattac
V1-69_19	cctggacaagggcttgagtggatgggagggatcatccctatctttggtacagcaaactacgcacagaagttccagggcagagtcacgattac
V1-69_20	cctggacaagggcttgagtggatgggaaggatcatccctatccttggtacagcaaactacgcacagaagttccagggcagagtcacgattac
V1-69_21	cctggacaagggcttgagtggatgggaaggatcatccctatccttggtacagcaaactacgcacagaagttccagggcagagtcacgattac
V1-69_22	cctggacaagggcttgagtggatgggagggatcatccctatccttggtacagcaaactacgcacagaagttccagggcagagtcacgattac
V1-69_23	cctggacaagggcttgagtggatgggaaggatcatccctatctttggtacagcaaactacgcacagaagttccagggcagagtcacgattac
V1-69_24	cctggacaagggcttgagtggatgggaaggatcatccctatctttggtacagcaaactacgcacagaagttccagggcagagtcacgattac
V1-69_25	cctggacaagggcttgagtggatgggagggatcatccctatctttggtacagcaaactacgcacagaagttccagggcagagtcacgattac
V1-69_26	cctggacaagggcttgagtggatgggagggatcatccctatccttggtacagcaaactacgcacagaagttccagggcagagtcacgattac

V1-69_27	cctggacaagggcttgagtggatgggaaggatcatccctatccttggtacagcaaactacgcacagaagttccagggcagagtcacgattac
V1-69_28	cctggacaagggcttgagtggatgggaaggatcatccctatccttggtacagcaaactacgcacagaagttccagggcagagtcacgattac
V1-69_29	cctggacaagggcttgagtggatgggaaggatcatccctatccttggtacagcaaactacgcacagaagttccagggcagagtcacgattac
V1-69_30	cctggacaagggcttgagtggatgggagggatcatccctatctttggtacagcaaactacgcacagaagttccagggcagagtcacgattac
V1-69_32	cctggacaagggcttgagtggatgggaaggatcatccctatctttggtacagcaaactacgcacagaagttccagggcagagtcacgattac
V1-69_33	cctggacaagggcttgagtggatgggaaggatcatccctatctttggtacagcaaactacgcacagaagttccagggcagagtcacgattac
IGHV1-69*01	cctggacaagggcttgagtggatgggagggatcatccctatctttggtacagcaaactacgcacagaagttccagggcagagtcacgattac
IGHV1-69*03	cctggacaagggcttgagtggatgggagggatcatccctatctttggtacagcaaactacgcacagaagttccagggcagagtcacgattac
IGHV1-69*04	cctggacaagggcttgagtggatgggaaggatcatccctatccttggtatagcaaactacgcacagaagttccagggcagagtcacgattac
IGHV1-69*06	cctggacaagggcttgagtggatgggagggatcatccctatctttggtacagcaaactacgcacagaagttccagggcagagtcacgattac
IGHV1-69*14	cctggacaagggcttgagtggatgggagggatcatccctatctttggtacagcaaactacgcacagaagttccagggcagagtcacgattac
IGHV1-69*15	cctggacaagggcttgagtggatgggaaggatcatccctatctttggtacagcaaactacgcacagaagttccagggcagagtcacgattac
IGHV1-69*16	cctggacaagggcttgagtggatgggagggatcatccctatccttggtacagcaaactacgcacagaagttccagggcagagtcacgattac
IGHV1-69*17	cctggacaagggcttgagtggatgggagggatcatccctatctttggtatagcaaactacgcacagaagttccagggcagagtcacgattac
IGHV1-69*02	cctggacaagggcttgagtggatgggaaggatcatccctatccttggtatagcaaactacgcacagaagttccagggcagagtcacgattac
IGHV1-69*05	cctggacaagggcttgagtggatgggagggatcatccctatctttggtacagcaaactacgcacagaagttccagggcagagtcacgattac
IGHV1-69*07	cctggacaagggcttgagtggatgggaaggatcatccctatctttggtacagcaaactacgcacagaagttccagggcagagtcacgattac
IGHV1-69*08	cctggacaagggcttgagtggatgggaaggatcatccctatccttggtacagcaaactacgcacagaagttccagggcagagtcacgattac
IGHV1-69*09	cctggacaagggcttgagtggatgggaaggatcatccctatccttggtatagcaaactacgcacagaagttccagggcagagtcacgattac
IGHV1-69*10	cctggacaagggcttgagtggatgggagggatcatccctatccttggtatagcaaactacgcacagaagttccagggcagagtcacgattac
IGHV1-69*11	cctggacaagggcttgagtggatgggaaggatcatccctatccttggtacagcaaactacgcacagaagttccagggcagagtcacgattac
IGHV1-69*12	cctggacaagggcttgagtggatgggagggatcatccctatctttggtacagcaaactacgcacagaagttccagggcagagtcacgattac
IGHV1-69*13	cctggacaagggcttgagtggatgggagggatcatccctatctttggtacagcaaactacgcacagaagttccagggcagagtcacgattac
IGHV1-69D*01	cctggacaagggcttgagtggatgggagggatcatccctatctttggtacagcaaactacgcacagaagttccagggcagagtcacgattac
IP_V1-69-2*p03	cctggaaaagggcttgagtggatgggatgtgttgatcctgaagatggtgaaacaatatatgcagagaagttccagggcagagtcaccataac
IP_V1-69-2*p04	cctggaaaagggcttgagtggatgggacgtgttgatcctgaagatggtgaaacaatatatgcagagaagttccagggcagagtcaccataac
IP_V1-69*p14	cctggacaagggcttgagtggatgggagggatcatccctatctttggtatagcaaactacgcacagaagttccagggcagagtcacgattac
IP_V1-69*p18	cctggacaagggcttgagtggatgggaaggatcatccctatctttggtacagcaaactacgcacagaagttccagggcagagtcacgattac
IP_V1-69*p19	cctggacaagggcttgagtggatgggaaggatcatccctatctttggtacagcaaactacgcacagaagttccagggcagagtcacgattac
IP_V1-69*p19	cctggacaagggcttgagtggatgggaaggatcatccctatctttggtacagcaaactacgcacagaagttccagggcagagtcacgattac
IP_V1-69*p20	cctggacaagggcttgagtggatgggaaggatcatccctatctttggtatagcaaactacgcacagaagttccagggcagagtcacgattac
IP_V1-69*p21	cctggacaagggcttgagtggatgggaaggatcatccctatccttggtatagcaaactacgcacagaagttccagggcagagtcacgattac
IP_V1-69*p25	cctggacaagggcttgagtggatgggagggatcatccctatctttggtatagcaaactacgcacagaagttccagggcagagtcacgattac
IP_V1-69*p26	cctggacaagggcttgagtggatgggaaggatcatccctatccttggtatagcaaactacgcacagaagttccagggcagagtcacgattac
IP_V1-69*p27	cctggacaagggcttgagtggatgggaaggatcatccctatccttggtatagcaaactacgcacagaagttccagggcagagtcacgattac
IP_V1-69*p28	cctggacaagggcttgagtggatgggagggatcatccctatccttggtacagcaaactacgcacagaagttccagggcagagtcacgattac
IP_V1-69*p29	cctggacaagggcttgagtggatgggaaggatcatccctatctttggtacagcaaactacgcacagaagttccagggcagagtcacgattac
IP_V1-69*p30	cctggacaagggcttgagtggatgggagggatcatccctatccttggtatagcaaactacgcacagaagttccagggcagagtcacgattac
IP_V1-69*p31	cctggacaagggcttgagtggatgggaaggatcatccctatccttggtatagcaaactacgcacagaagttccagggcagagtcacgattac
IP_V1-69*p32	cctggacaagggcttgagtggatgggagggatcatccctatctttggtatagcaaactacgcacagaagttccagggcagagtcacgattac
IP_V1-69*p33	cctggacaagggcttgagtggatgggagggatcatccctatctttggtacagcaaactacgcacagaagttccagggcagagtcacgattac
IP_V1-69*p34	cctggacaagggcttgagtggatgggaaggatcatccctatccttggtatagcaaactacgcacagaagttccagggcagagtcacgattac
IP_V1-69*p35	cctggacaagggcttgagtggatgggagggatcatccctatctttggtacagcaaactacgcacagaagttccagggcagagtcacgattac
IP_V1-69*p36	cctggacaagggcttgagtggatgggaaggatcatccctatctttggtacagcaaactacgcacagaagttccagggcagagtcacgattac
IP_V1-69*p37	cctggacaagggcttgagtggatgggagggatcatccctatctttggtacagcaaactacgcacagaagttccagggcagagtcacgattac
IP_V1-69*p38	cctggacaagggcttgagtggatgggagggatcatccctatctttggtacagcaaactacgcacagaagttccagggcagagtcacgattac
V_V1-69*01	cctggacaagggcttgagtggatgggagggatcatccctatctttggtacagcaaactacgcacagaagttccagggcagagtcacgattac

```
V_V1-69*04      cctggacaagggcttgagtggatgggaaggatcatccctatccttggtatagcaaactacgcacagaagttccagggcagagtcacgattac
V_V1-69*06      cctggacaagggcttgagtggatgggagggatcatccctatcttggtacagcaaactacgcacagaagttccagggcagagtcacgattac


V1-69_01        cgcggacgaatccacgagcacagcctacatggagctgagcagcctgagatctgaggacacggccgtgtattactgtgcgaga--
V1-69_02        cacggacgaatccacgagcacagcctacatggagctgagcagcctgagatctgaggacacggccgtgtattactgtgcgaga--
V1-69_03        cgcggacaaatccacgagcacagcctacatggagctgagcagcctgagatctgaggacacggccgtgtattactgtgcgaga--
V1-69_04        cgcggacgaatccacgagcacagcctacatggagctgagcagcctgagatctgaggacacggccgtgtattactgtgcgaga--
V1-69_05        cgcggacgaatccacgagcacagcctacatggagctgagcagcctgagatctgaggacacggccgtgtattactgtgcgaga--
V1-69_06        cgcggacgaatccacgagcacagcctacatggagctgagcagcctgagatctgaggacacggccgtgtattactgtgcgaga--
V1-69_07        cgcggacgaatccacgagcacagcctacatggagctgagcagcctgagatctgaggacacggccgtgtattactgtgcgaga--
V1-69_08        cgcggacaaatccacgagcacagcctacatggagctgagcagcctgagatctgaggacacggccgtgtattactgtgcgaga--
V1-69_09        cgcggacaaatccacgagcacagcctacatggagctgagcagcctgagatctgaggacacggccgtgtattactgtgcgaga--
V1-69_10        cgcggacaaatccacgagcacagcctacatggagctgagcagcctgagatctgaggacacggccgtgtattactgtgcgaga--
V1-69_11        cgcggacaaatccacgagcacagcctacatggagctgagcagcctgagatctgaggacacggccgtgtattactgtgcgaga--
V1-69_12        cgcggacaaatccacgagcacagcctacatggagctgagcagcctgagatctgaggacacggccgtgtattactgtgcgaga--
V1-69_13        cgcggacaaatccacgagcacagcctacatggagctgagcagcctgagatctgaggacacggccgtgtattactgtgcgaga--
V1-69_14        cgcggacaaatccacgagcacagcctacatggagctgagcagcctgagatctgaggacacggccgtgtattactgtgcgaga--
V1-69_15        cgcggacgaatccacgagcacagcctacatggagctgagcagcctgagatctgaggacacggccgtgtattactgtgcgaga--
V1-69_16        cgcggacaaatccacgagcacagcctacatggagctgagcagcctgagatctgaggacacggccgtgtattactgtgcgaga--
V1-69_17        cacggacaaatccacgagcacagcctacatggagctgagcagcctgagatctgaggacacggccgtgtattactgtgcgaga--
V1-69_18        cgcggacaaatccacgagcacagcctacatggagctgagcagcctgagatctgaggacacggccgtgtattactgtgcgaga--
V1-69_19        cacggacgaatccacgagcacagcctacatggagctgagcagcctgagatctgaggacacggccgtgtattactgtgcgaga--
V1-69_20        cgcggacaaatccacgagcacagcctacatggagctgagcagcctgagatctgaggacacggccgtgtattactgtgcgaga--
V1-69_21        cgcggacaaatccacgagcacagcctacatggagctgagcagcctgagatctgaggacacggccgtgtattactgtgcgaga--
V1-69_22        cgcggacgaatccacgagcacagcctacatggagctgagcagcctgagatctgaggacacggccgtgtattactgtgcgaga--
V1-69_23        cacggacgaatccacgagcacagcctacatggagctgagcagcctgagatctgaggacacggccgtgtattactgtgcgaga--
V1-69_24        cacggacgaatccacgagcacagcctacatggagctgagcagcctgagatctgaggacacggccgtgtattactgtgcgaga--
V1-69_25        cgcggacaaatccacgagcacagcctacatggagctgagcagcctgagatctgaggacacggccgtgtattactgtgcgaga--
V1-69_26        cgcggacgaatccacgagcacagcctacatggagctgagcagcctgagatctgaggacacggccgtgtattactgtgcgaga--
V1-69_27        cgcggacaaatccacgagcacagcctacatggagctgagcagcctgagatctgaggacacggccgtgtattactgtgcgaga--
V1-69_28        cgcggacgaatccacgagcacagcctacatggagctgagcagcctgagatctgaggacacggccgtgtattactgtgcgaga--
V1-69_29        cacggacaaatccacgagcacagcctacatggagctgagcagcctgagatctgaggacacggccgtgtattactgtgcgaga--
V1-69_30        cgcggacgaatccacgagcacagcctacatggagctgagcagcctgagatctgaggacacggccgtgtattactgtgcgaga--
V1-69_32        cgcggacaaatccacgagcacagcctacatggagctgagcagcctgagatctgaggacacggccgtgtattactgtgcgaga--
V1-69_33        cgcggacgaatccacgagcacagcctacatggagctgagcagcctgagatctgaggacacggccgtgtattactgtgcgaga--
IGHV1-69*01     cgcggacgaatccacgagcacagcctacatggagctgagcagcctgagatctgaggacacggccgtgtattactgtgcgagaga
IGHV1-69*03     cgcggacgaatccacgagcacagcctacatggagctgagcagcctgagatctgatgacacggc---------------------
IGHV1-69*04     cgcggacaaatccacgagcacagcctacatggagctgagcagcctgagatctgaggacacggccgtgtattactgtgcgagaga
IGHV1-69*06     cgcggacaaatccacgagcacagcctacatggagctgagcagcctgagatctgaggacacggccgtgtattactgtgcgagaga
IGHV1-69*14     cgcggacaaatccacgagcacagcctacatggagctgagcagcctgagatctgaggacacggccgtgtattactgtgcgagaga
IGHV1-69*15     cgcggacgaatccacgagcacagcctacatggagctgagcagcctgagatctgaggacacggccgtgtattactgtgcgagaga
IGHV1-69*16     cacggacgaatccacgagcacagcctacatggagctgagcagcctgagatctgaggacacggccgtgtattactgtgcgagaga
IGHV1-69*17     cgcggacaaatccacgagcacagcctacatggagctgagcagcctgagatctgaggacacggccgtgtattactgtgcgagaga
IGHV1-69*02     cgcggacaaatccacgagcacagcctacatggagctgagcagcctgagatctgaggacacggccgtgtattactgtgcgaga--
IGHV1-69*05     cacggacgaatccacgagcacagcctacatggagctgagcagcctgagatctgaggacacggccgtgtattactgtgcgaga--
IGHV1-69*07     cgcggacgaatccacgagcacagcctacatggagctgagcagcctgagatctgag-----------------------------
```

```
IGHV1-69*08      cgcggacaaatccacgagcacagcctacatggagctgagcagcctgagatctgaggacacggccgtgtattactgtgcgagaga
IGHV1-69*09      cgcggacaaatccacgagcacagcctacatggagctgagcagcctgagatctgaggacacggccgtgtattactgtgcgagaga
IGHV1-69*10      cgcggacaaatccacgagcacagcctacatggagctgagcagcctgagatctgaggacacggccgtgtattactgtgcgagaga
IGHV1-69*11      cgcggacgaatccacgagcacagcctacatggagctgagcagcctgagatctgaggacacggccgtgtattactgtgcgagaga
IGHV1-69*12      cgcggacgaatccacgagcacagcctacatggagctgagcagcctgagatctgaggacacggccgtgtattactgtgcgagaga
IGHV1-69*13      cgcggacgaatccacgagcacagcctacatggagctgagcagcctgagatctgaggacacggccgtgtattactgtgcgagaga
IGHV1-69D*01     cgcggacgaatccacgagcacagcctacatggagctgagcagcctgagatctgaggacacggccgtgtattactgtgcgaga--
IP_V1-69-2*p03   cgcagacacgtctacagacacagcctacatggagctgagcagcctgagatctgaggacacggccgtgtattactgtgcaaca--
IP_V1-69-2*p04   cgcagacacgtctacagacacagcctacatggagctgagcagcctgagatctgaggacacggccgtgtattactgtgcaaca--
IP_V1-69*p14     cgcggacaaatccacgagcacagcctacatggagctgagcagc-------------------------------------------
IP_V1-69*p18     ------caaatccacgagcacagcctacatggagctgagcagcctgagatctgaggacacggccgtgtattactgtgcgagaga
IP_V1-69*p19     cgcggacgaatccacgagcacagcctacatggagctgagcagcctgagatctgaggacacggccgtgtattactgtgcgagaga
IP_V1-69*p19     cgcggacgaatccacgagcacagcctacatggagctgagcagcctgagatctgaggacacggccgtgtattactgtgcgaga--
IP_V1-69*p20     cgcggacaaatccacgagcacagcctacatggagctgagcagcctgagatctgaggacacggccgtgtattactgtgcgagaga
IP_V1-69*p21     cgcggacgaatccacgagcacagcctacatggagctgagcagcctgagatctgaggacacggccgtgtattactgtgcgagaga
IP_V1-69*p25     cgcggacaaatccacgagcacagcctacatggagctgagcagcctgagatctgaggacacggccgtgtattactgtgcgagaga
IP_V1-69*p26     cgcggacaaatccacgagcacagcctacatggagctgagcagcctgagatctgaggacacggccgtgtattactgtgcgagaga
IP_V1-69*p27     cgcggacgaatccacgagcacagcctacatggagctgagcagcctgagatctgaggacacggccgtgtattactgtgcgaga--
IP_V1-69*p28     cacggacgaatccacgagcacagcctacatggagctgagcagcctgagatctgaggacacggccgtgtattactgtgcgaga--
IP_V1-69*p29     cgcggacaaatccacgagcacagcctacatggagctgagcagcctgagatctgaggacacggccgtgtattactgtgcgaga--
IP_V1-69*p30     cgcggacaaatccacgagcacagcctacatggagctgagcagcctgagatctgaggacacggccgtgtattactgtgcgagaga
IP_V1-69*p31     cgcggacgaatccacgagcacagcctacatggagctgagcagcctgagatctgaggacacggccgtgtattactgtgcgagaga
IP_V1-69*p32     cgcggacgaatccacgagcacagcctacatggagctgagcagcctgagatctgaggacacggccgtgtattactgtgcgagaga
IP_V1-69*p33     cgcggacaaatccacgagcacagcctacatggagctgagcagcctgagatctgaggacacggccgtgtattactgtgcgagaga
IP_V1-69*p34     cgcggacaaatccacgagcacagcctacatggagctgagcagcctgagatctgaggacacggccgtgtattactgtgcgagaga
IP_V1-69*p35     cacggacgaatccacgagcacagcctacatggagctgagcagcctgagatctgaggacacggccgtgtattactgtgcgagaga
IP_V1-69*p36     cgcggacgaatccacgagcacagcctacatggagctgagcagcctgagatctgaggacacggccgtgtattactgtgcgagaga
IP_V1-69*p37     cacggacgaatccacgagcacagcctacatggagctgagcagcctgagatctgaggacacggccgtgtattactgtgcgagaga
IP_V1-69*p38     cgcggacgaatccacgagcacagcctacatggagctgagcagcctgagatctgaggacacggccgtgtattactgtgcgagaga
V_V1-69*01       cgcggacgaatccacgagcacagcctacatggagctgagcagcctgagatctgaggacacggccgtgtattactgtgcgaga--
V_V1-69*04       cgcggacaaatccacgagcacagcctacatggagctgagcagcctgagatctgaggacacggccgtgtattactgtgcgaga--
V_V1-69*06       cgcggacaaatccacgagcacagcctacatggagctgagcagcctgagatctgaggacacggccgtgtattactgtgcgaga--
```

IGHV2-5

```
V2-5_01        atggacacactttgctccacgctcctgctgctgaccatcccttcatgggtcttgtcccagatcaccttgaaggagtctggtcctacgctggtga
V2-5_02        atggacacactttgctccacgctcctgctgctgaccatcccttcatgggtcttgtcccagatcaccttgaaggagtctggtcctacgctggtga
V2-5_03        atggacacactttgctccacgctcctgctgctgaccatcccttcatgggtcttgtcccagatcaccttgaaggagtctggtcctacgctggtga
IGHV2-5*01     atggacacactttgctccacgctcctgctgctgaccatcccttcatgggtcttgtcccagatcaccttgaaggagtctggtcctacgctggtga
IGHV2-5*02     atggacacactttgctccacgctcctgctgctgaccatcccttcatgggtcttgtcccagatcaccttgaaggagtctggtcctacgctggtga
IGHV2-5*04     atggacatactttgttccacgctcctgctgctgaccatcccttcatgggtcttgtcccagatcaccttgaaggagtctggtcctacgctggtga
IGHV2-5*05     atggacatactttgttccacgctcctgctgctgaccatcccttcatgggtcttgtcccagatcaccttgaaggagtctggtcctacgctggtga
IGHV2-5*06     atggacatactttgttccacgctcctgctgctgaccatcccttcatgggtcttgtcccagatcaccttgaaggagtctggtcctacgctggtaa
IGHV2-5*08     atggacatactttgttccacgctcctgctactgactgtcccgtcctgggtcttatcccaggtcaccttgaaggagtctggtcctgcgctggtga
IGHV2-5*09     atggacatactttgttccacgctcctgctactgactgtcccgtcctgggtcttatcccaggtcaccttgaaggagtctggtcctacgctggtga
IGHV2-5*03     -------------------------------------------------------------------------------------gctggtga
IP_V2-5*p11    ---------------------------------------------------------cagatcaccttgaaggagtctggtcctacgctggtga
IP_V2-5*p12    ---------------------------------------------------------caggtcaccttgaaggagtctggtcctgcgctggtga
IP_V2-5*p13    ---------------------------------------------------------caggtcaccttgaaggagtctggtcctacgctggtga
IP_V2-5*p14    ---------------------------------------------------------cagatcaccttgaaggagtctggtcctacgctggtga
IP_V2-5*p15    ---------------------------------------------------------cagatcaccttgaaggagtctggtcctacgctggtga
V_V2-5*10      ---------------------------------------------------------cagatcaccttgaaggagtctggtcctacgctggtga
V_V2-5*01      ---------------------------------------------------------cagatcaccttgaaggagtctggtcctacgctggtga

V2-5_01        aacccacacagaccctcacgctgacctgcaccttctctgggttctcactcagcactagtggagtgggtgtgggctggatccgtcag
V2-5_02        aacccacacagaccctcacgctgacctgcaccttctctgggttctcactcagcactagtggagtgggtgtgggctggatccgtcag
V2-5_03        aacccacacagaccctcacgctgacctgcaccttctctgggttctcactcagcactagtggagtgggtgtgggctggatccgtcag
IGHV2-5*01     aacccacacagaccctcacgctgacctgcaccttctctgggttctcactcagcactagtggagtgggtgtgggctggatccgtcag
IGHV2-5*02     aacccacacagaccctcacgctgacctgcaccttctctgggttctcactcagcactagtggagtgggtgtgggctggatccgtcag
IGHV2-5*04     aacccacacagaccctcacgctgacctgcaccttctctgggttctcactcagcactagtggagtgggtgtgggctggatccgtcag
IGHV2-5*05     aacccacacagaccctcacgctgacctgcaccttctctgggttctcactcagcactagtggagtgggtgtgggctggatccgtcag
IGHV2-5*06     aacccacacagaccctcacgctgacctgcaccttctctgggttctcactcagcactagtggagtgggtgtgggctggatccgtcag
IGHV2-5*08     aacccacacagaccctcacactgacctgcaccttctctgggttctcactcagcactagtggaatgcgtgtgactggatccgtcag
IGHV2-5*09     aacccacacagaccctcacgctgacctgcaccttctctgggttctcactcagcactagtggagtgggtgtgggctggatccgtcag
IGHV2-5*03     aacccacacagaccctcacgctgacctgcaccttctctgggttctcactcagcactagtggagtgggtgtgggctggatccgtcag
IP_V2-5*p11    aacccacacagaccctcacgctgacctgcaccttctctgggttctcactcagcactagtggagtgggtgtgggctggatccgtcag
IP_V2-5*p12    aacccacacagaccctcacgctgacctgcaccttctctgggttctcactcagcactagtggagtgggtgtgggctggatccgtcag
IP_V2-5*p13    aacccacacagaccctcacgctgacctgcaccttctctgggttctcactcagcactagtggagtgggtgtgggctggatccgtcag
IP_V2-5*p14    aacccacacagaccctcacgctgacctgcaccttctctgggttctcactcagcactagtggagtgggtgtgggctggatccgtcag
IP_V2-5*p15    aacccacacagaccctcacgctgacctgcaccttctctgggttctcactcagcactagtggagtgggtgtgggctggatccgtcag
V_V2-5*10      aacccacacagaccctcacgctgacctgcaccttctctgggttctcactcagcactagtggagtgggtgtgggctggatccgtcag
V_V2-5*01      aacccacacagaccctcacgctgacctgcaccttctctgggttctcactcagcactagtggagtgggtgtgggctggatccgtcag
```

```
V2-5_01       cccccaggaaaggccctggagtggcttgcactcatttattggaatgatgataagcgctacagcccatctctgaagagcaggctcaccatcacc
V2-5_02       cccccaggaaaggccctggagtggcttgcactcatttattgggatgatgataagcgctacagcccatctctgaagagcaggctcaccatcacc
V2-5_03       cccccaggaaaggccctggagtggcttgcactcatttattgggatgatgataagcgctacggcccatctctgaagagcaggctcaccatcacc
IGHV2-5*01    cccccaggaaaggccctggagtggcttgcactcatttattggaatgatgataagcgctacagcccatctctgaagagcaggctcaccatcacc
IGHV2-5*02    cccccaggaaaggccctggagtggcttgcactcatttattgggatgatgataagcgctacagcccatctctgaagagcaggctcaccatcacc
IGHV2-5*04    cccccaggaaaggccctggagtggcttgcactcatttattggaatgatgataagcgctacagcccatctctgaagagcaggctcaccatcacc
IGHV2-5*05    cccccaggaaaggccctggagtggcttgcactcatttattgggatgatgataagcgctacggcccatctctgaagagcaggctcaccatcacc
IGHV2-5*06    cccccaggaaaggccctggagtggcttgcactcatttattgggatgatgataagcgctacggcccatctctgaagagcaggctcaccatcacc
IGHV2-5*08    cccccaggaaaggccctggagtggcttgcactcatttattgggatgatgataagcgctacagcccatctctgaagagcaggctcaccatcacc
IGHV2-5*09    cccccaggaaaggccctggagtggcttgcactcatttattgggatgatgataagcgctacggcccatctctgaagagcaggctcaccatcacc
IGHV2-5*03    cccccaggaaaggccctggagtggcttgcactcatttattgggatgatgataagcgctacagcccatctctgaagagcaggctcaccattacc
IP_V2-5*p11   cccccaggaaaggccctggagtggcttgcactcatttattgggatgatgataagcgctacagcccatctctgaagagcaggctcaccatcacc
IP_V2-5*p12   cccccaggaaaggccctggagtggcttgcactcatttattggaatgatgataagcgctacagcccatctctgaagagcaggctcaccatcacc
IP_V2-5*p13   cccccaggaaaggccctggagtggcttgcactcatttattgggatgatgataagcgctacagcccatctctgaagagcaggctcaccatcacc
IP_V2-5*p14   cccccaggaaaggccctggagtggcttgcactcatttattgggatgatgataagcgctacagcccatctctgaagagcaggctcaccatcacc
IP_V2-5*p15   cccccaggaaaggccctggagtggcttgcactcatttattggaatgatgataagcgctacagcccatctctgaagagcaggctcaccatcacc
V_V2-5*10     cccccaggaaaggccctggagtggcttgcactcatttattgggatgatgataagcgctacagcccatctctgaagagcaggctcaccatcacc
V_V2-5*01     cccccaggaaaggccctggagtggcttgcactcatttattggaatgatgataagcgctacagcccatctctgaagagcaggctcaccatcacc
```

```
V2-5_01       aaggacacctccaaaaaccaggtggtccttacaatgaccaacatggaccctgtggacacagccacatattactgtgcacacaga
V2-5_02       aaggacacctccaaaaaccaggtggtccttacaatgaccaacatggaccctgtggacacagccacatattactgtgcacacaga
V2-5_03       aaggacacctccaaaaaccaggtggtccttacaatgaccaacatggaccctgtggacacagccacatattactgtgcacacaga
IGHV2-5*01    aaggacacctccaaaaaccaggtggtccttacaatgaccaacatggaccctgtggacacagccacatattactgtgcacacaga
IGHV2-5*02    aaggacacctccaaaaaccaggtggtccttacaatgaccaacatggaccctgtggacacagccacatattactgtgcacacaga
IGHV2-5*04    aaggacacctccaaaaaccaggtggtccttacaatgaccaacatggaccctgtggacacaggcacatattactgtgtacgg---
IGHV2-5*05    aaggacacctccaaaaaccaggtggtccttacaatgaccaacatggaccctgtggacacagccacatattactgtgcacacaga
IGHV2-5*06    aaggacacctccaaaaaccaggtggtccttacaatgaccaacatggaccctgtggacacagccacatattactgtgcacacaga
IGHV2-5*08    aaggacacctccaaaaaccaggtggtccttacaatgaccaacatggaccctgtggacacagccacatattactgtgcacacaga
IGHV2-5*09    aaggacacctccaaaaaccaggtggtccttacaatgaccaacatggaccctgtggacacagccacatattactgtgcacacaga
IGHV2-5*03    aaggacacctccaaaaaccaggt-------------------------------------------------------------
IP_V2-5*p11   aaggacacctccaaaaaccaggtggtccttacaatgaccaacatggaccctgtggacacagccacatattactgtgcacacaga
IP_V2-5*p12   aaggacacctccaaaaaccaggtggtccttacaatgaccaacatggaccctgtggacacagccacatattactgtgcacacag-
IP_V2-5*p13   aaggacacctccaaaaaccaggtggtccttacaatgaccaacatggaccctgtggacacagccacatattactgtgcacacag-
IP_V2-5*p14   aaggacacctccaaaaaccaggtggtccttacaatgaccaacatggaccctgtggacacagccacgtattactgtgcacg----
IP_V2-5*p15   aaggacacctccaaaaaccaggtggtccttacaatgaccaacatggaccctgtggacacagccacatattactgtgcacgcaga
V_V2-5*10     aaggacacctccaaaaaccaggtggtccttacaatgaccaacatggaccctgtggacacagccacatattactgtgcacac---
V_V2-5*01     aaggacacctccaaaaaccaggtggtccttacaatgaccaacatggaccctgtggacacagccacatattactgtgcacac---
```

IGHV2-26

```
V2-26_01        atggacacactttgctacacactcctgctgctgaccaccccttcctgggtcttgtcccaggtcaccttgaaggagtctggtcctgtgctgg
V2-26_02        atggacacgctttgctacacactcctgctgctgaccaccccttcctgggtcttgtcccaggtcaccttgaaggagtctggtcctgtgctgg
V2-26_03        atggacacgctttgctacacactcctgctgctgaccaccccttcctgggtcttgtcccaggtcaccttgaaggagtctggtcctgtgctgg
V2-26_04        atggacacgctttgctacacactcctgctgctgaccaccccttcctgggtcttgtcccaggtcaccttgaaggagtctggtcctgtgctgg
V2-26_05        atggacacgctttgctacacactcctgctgctgaccaccccttcctgggtcttgtcccaggtcaccttgaaggagtctggtcctgtgctgg
V2-26_06        atggacacactttgctacacactcctgctgctgaccaccccttcctgggtcttgtcccaggtcaccttgaaggagtctggtcctgtgctgg
IGHV2-26*01     atggacacactttgctacacactcctgctgctgaccaccccttcctgggtcttgtcccaggtcaccttgaaggagtctggtcctgtgctgg
IGHV2-26*02     atggacacgctttgctacacactcctgctgctgaccaccccttcctgggtcttgtcccaggtcaccttgaaggagtctggtcctgtgctgg
IGHV2-26*03     atggacacactttgctacacactcctgctgctgaccaccccttcctgggtcttgtcccaggtcaccttgaaggagtctggtcctgtgctgg
IP_V2-26*p02    --------------------------------------------------------caggtcaccttgaaggagtctggtcctgtgctgg
IP_V2-26*p03    --------------------------------------------------------caggtcaccttgaaggagtctggtcctgtgctgg
IP_V2-26*p04    --------------------------------------------------------caggtcaccttgaaggagtctggtcctgtgctgg
IP_V2-26*p05    --------------------------------------------------------caggtcaccttgaaggagtctggtcctgtgctgg
IP_V2-26*p06    --------------------------------------------------------caggtcaccttgaaggagtctggtcctgtgctgg
IP_V2-26*p07    --------------------------------------------------------caggtcaccttgaaggagtctggtcctgtgctgg
IP_V2-26*p08    --------------------------------------------------------caggtcaccttgaaggagtctggtcctgcgctgg
IP_V2-26*p09    --------------------------------------------------------caggtcaccttgaaggagtctggtcctgtgctgg
IP_V2-26*p10    --------------------------------------------------------caggtcaccttgaaggagtctggtcctgtgctgg
IP_V2-26*p11    --------------------------------------------------------caggtcaccttgaaggagtctggtcctgtgctgg
IP_V2-26*p12    --------------------------------------------------------caggtcaccttgaaggagtctggtcctgtgctgg
IP_V2-26*p13    --------------------------------------------------------caggtcaccttgagggagtctggtcctgcgctgg
V_V2-26*01      --------------------------------------------------------caggtcaccttgaaggagtctggtcctgtgctgg

V2-26_01        tgaaacccacagagaccctcacgctgacctgcaccgtctctgggttctcactcagcaatgctagaatgggtgtgagctggatccgtcag
V2-26_02        tgaaacccacagagaccctcacgctgacctgcaccgtctctgggttctcactcagcaatgctagaatgggtgtgagctggatccgtcag
V2-26_03        tgaaacccacagagaccctcacgctgacctgcaccgtctctgggttctcactcagcaatgctagaatgggtgtgagctggatccgtcag
V2-26_04        tgaaacccacagagaccctcacgctgacctgcaccgtctctgggttctcactcagcaatgctagaatgggtgtgagctggatccatcag
V2-26_05        tgaaacccacagagaccctcacgctgacctgcaccgtctctgggttctcagtcagcaatgctagaatgggtgtgagctggatccgtcag
V2-26_06        taaaacccacagagaccctcacgctgacctgcaccgtctctgggttctcactcagcaatgctagaatgggtgtgagctggatccgtcag
IGHV2-26*01     tgaaacccacagagaccctcacgctgacctgcaccgtctctgggttctcactcagcaatgctagaatgggtgtgagctggatccgtcag
IGHV2-26*02     tgaaacccacagagaccctcacgctgacctgcaccgtctctgggttctcactcagcaatgctagaatgggtgtgagctggatccgtcag
IGHV2-26*03     tgaaacccacagagaccctcacgctgacctgcaccatctctgggttctcactcagcaatgctagaatgggtgtgagctggatccgtcag
IP_V2-26*p02    tgaaacccacagagaccctcacgctgacctgcaccgtctctgggttctcactcagcaatgctagaatgggtgtgagctggatccgtcag
IP_V2-26*p03    tgaaacccacagagaccctcacgctgacctgcaccgtctctgggttctcactcagcaatgctagaatgggtgtgagctggatccgtcag
IP_V2-26*p04    tgaaacccacagagaccctcacgctgacctgcaccgtctctgggttctcactcagcaatgctagaatgggtgtgagctggatccgtcag
IP_V2-26*p05    tgaaacccacagagaccctcacgctgacctgcaccgtctctgggttctcactcagcaatgctagaatgggtgtgagctggatccgtcag
IP_V2-26*p06    tgaaacccacagagaccctcacgctgacctgcaccgtctctgggttctcactcagcaatgctagaatgggtgtgagctggatccgtcag
IP_V2-26*p07    tgaaacccacagagaccctcacgctgacctgcaccatctctgggttctcactcagcaatgctagaatgggtgtgagctggatccgtcag
IP_V2-26*p08    tgaaacccacagagaccctcacgctgacctgcaccgtctctgggttctcactcagcaatgctagaatgggtgtgagctggatccgtcag
IP_V2-26*p09    tgaaacccacagagaccctcacgctgacctgcaccgtctctgggttctcactcagcaatgctagaatgggtgtgagctggatccgtcag
IP_V2-26*p10    tgaaacccacagagaccctcacgctgacctgcaccgtctctgggttctcactcagcaatgctagaatgggtgtgagctggatccatcag
IP_V2-26*p11    tgaaacccacagagaccctcacgctgacctgcaccgtctctgggttctcagtcagcaatgctagaatgggtgtgagctggatccgtcag
IP_V2-26*p12    tgaaacccacagagaccctcacgctgacctgcaccgtctctgggttctcactcagcaatgctagaatgggtgtgagctggatccgtcag
IP_V2-26*p13    tgaaacccacacagaccctcacactgacctgcacctctctgggttctcactcagcactagtggaatgtgtgtgagctggatccgtcag
V_V2-26*01      tgaaacccacagagaccctcacgctgacctgcaccgtctctgggttctcactcagcaatgctagaatgggtgtgagctggatccgtcag
```

```
V2-26_01        cccccagggaaggccctggagtggcttgcacacattttttcgaatgacgaaaaatcctacagcacatctctgaagagcaggctcaccatc
V2-26_02        cccccagggaaggccctggagtggcttgcacacattttttcgaatgacgaaaaatcctacagcacatctctgaagagcaggctcaccatc
V2-26_03        cccccagggaaggccctggagtggcttgcacacattttttcgaatgacgaaaaatcctacagcacatctctgaagagcaggctcaccatc
V2-26_04        cccccagggaaggccctggagtggcttgcacacattttttcgaatgacgaaaaatcctacagcacatctctgaagagcaggctcaccatc
V2-26_05        cccccagggaaggccctggagtggcttgaacacattttttcgaatgacgaaaaatcctacagcacatctctgaagagcaggctcaccatc
V2-26_06        cccccagggaaggccctggagtggcttgcacacattttttcgaatgacgaaaaatcctacagcacatctctgaagagcaggctcaccatc
IGHV2-26*01     cccccagggaaggccctggagtggcttgcacacattttttcgaatgacgaaaaatcctacagcacatctctgaagagcaggctcaccatc
IGHV2-26*02     cccccagggaaggccctggagtggcttgcacacattttttcgaatgacgaaaaatcctacagcacatctctgaagagcaggctcaccatc
IGHV2-26*03     cccccagggaaggccctggagtggcttgcacacattttttcgaatgacgaaaaatcctacagcacatctctgaagagcaggctcaccatc
IP_V2-26*p02    cccccagggaaggccctggagtggcttgcacacattttttcgaatgacgaaaaatcctacagcacatctctgaagagcaggctcaccatc
IP_V2-26*p03    cccccagggaaggccctggagtggcttgcacacattttttcgaatgacgaaaaatcctacagcacatctctgaagagcaggctcaccatc
IP_V2-26*p04    cccccagggaaggccctggagtggcttgcacacatttttacgaatgacgaaaaatcctacagcacatctctgaagagcaggctcaccatc
IP_V2-26*p05    cccccagggaaggccctggagtggcttgcacacatttttccgaatgacgaaaaatcctacagcacatctctgaagagcaggctcaccatc
IP_V2-26*p06    cgcccagggaaggccctggagtggcttgcacacattttttcgaatgacgaaaaatcctacagcacatctctgaagagcaggctcaccatc
IP_V2-26*p07    cccccagggaaggccctggagtggcttgcacacattttttcgaatgacgaaaaatcctacagcacatctctgaagagcaggctcaccatc
IP_V2-26*p08    cccccagggaaggccctggagtggcttgcacacattttttcgaatgacgaaaaatcctacagcacatctctgaagagcaggctcaccatc
IP_V2-26*p09    cccccagggaaggccctggagtggcttgcacacattttttcgaatgacgaaaaatcctacagcacatctctgaagagcaggctcaccatc
IP_V2-26*p10    cccccagggaaggccctggagtggcttgcacacattttttcgaatgacgaaaaatcctacagcacatctctgaagagcaggctcaccatc
IP_V2-26*p11    cccccagggaaggccctggagtggcttgaacacattttttcgaatgacgaaaaatcctacagcacatctctgaagagcaggctcaccatc
IP_V2-26*p12    cccccagggaaggccctggagtggcttgcacacattttttcgaatgacgaaaaatcctacagcacatctctgaagagcaggctcaccatc
IP_V2-26*p13    cccccagggaaggccctggagtggcttgcacacattttttcgaatgacgaaaaatcctacagcacatctctgaagagcaggctcaccatc
V_V2-26*01      cccccagggaaggccctggagtggcttgcacacattttttcgaatgacgaaaaatcctacagcacatctctgaagagcaggctcaccatc

V2-26_01        tccaaggacacctccaaaagccaggtggtccttaccatgaccaacatggaccctgtggacacagccacatattactgtgcacggata
V2-26_02        tccaaggacacctccaaaagccaggtggtccttaccatgaccaatatggaccctgtggacacagccacatattactgtgcacggata
V2-26_03        tccaaggacacctccaaaagccaggtggtccttaccatgaccaatatggaccctgtggacacagccacatattactgtgcatggata
V2-26_04        tccaaggacacctccaaaagccaggtggtccttaccatgaccaatatggaccctgtggacacagccacatattactgtgcacggata
V2-26_05        tccaaggacacctccaaaagccaggtggtccttaccatgaccaatatggaccctgtggacacagccacatattactgtgcacggata
V2-26_06        tccaaggacacctccaaaagccaggtggtccttaccatgaccaacatggaccctgtggacacagccacatattactgtgcacggata
IGHV2-26*01     tccaaggacacctccaaaagccaggtggtccttaccatgaccaacatggaccctgtggacacagccacatattactgtgcacggata
IGHV2-26*02     tccaaggacacctccaaaagccaggtggtccttaccatgaccaatatggaccctgtggacacagccacatattactgtgcacggata
IGHV2-26*03     tccaaggacacctccaaaagccaggtggtccttaccatgaccaacatggaccctgtggacacagccacatattactgtgcacggata
IP_V2-26*p02    tccaaggacacctccaaaagccaggtggtccttaccatgaccaatatggaccctgtggacacagccacatattactgtgcacgga--
IP_V2-26*p03    tccaaggacacctccaaaagccaggtggtccttaccatgaccaacatggaccctgtggacacagccacgtattactgtgcacggat-
IP_V2-26*p04    tccaaggacacct--------------------------------------------------------------------------
IP_V2-26*p05    tccaaggacacctccaaaagccaggtggtccttaccatgaccaacatggaccctgtggacacagccacatattactgtgcacggat-
IP_V2-26*p06    tccaaggacacctccaaaagccaggtggtccttaccatgaccaacatggaccctgtggacacagccacatattactgtgcacggat-
IP_V2-26*p07    tccaaggacacctccaaaagccaggtggtccttaccatgaccaacatggaccctgtggacacagccacatattactgtgcacggata
IP_V2-26*p08    tccaaggacacctccaaaagccaggtggtccttaccatgaccaacatggaccctgtggacacagccacatattactgtgcacggat-
IP_V2-26*p09    tccaaggacacctccaaaagccaggtggtccttaccatgaccaacatggaccctgtggacacagccacatattactgtgcacag---
IP_V2-26*p10    tccaaggacacctccaaaagccaggtggtccttaccatgaccaatatggaccctgtggacacagccacatattactgtgcacggat-
IP_V2-26*p11    tccaaggacacctccaaaagccaggtggtccttaccatgaccaatatggaccctgtggacacagccacatattactgtgcacggat-
IP_V2-26*p12    tccaaggacacctccaaaagccaggtggtccttaccatgaccaatatggaccctgtggacacagccacatattactgtgcatggat-
IP_V2-26*p13    tccaaggacacctccaaaagccaggtggtccttaccatgaccaacatggaccctgtggacacagccacatattactgtgcacggat-
V_V2-26*01      tccaaggacacctccaaaagccaggtggtccttaccatgaccaacatggaccctgtggacacagccacatattactgtgcacgg---
```

IGHV2-70

```
V2-70_01        atggacatactttgttccacgctcctgctactgactgtcccgtcctgggtcttatcccaggtcaccttgagggagtctggtcctgcgctgg
V2-70_02        atggacatactttgttccacgctcctgctactgactgtcccgtcctgggtcttatcccaggtcaccttgagggagtctggtcctgcgctgg
V2-70_03        atggacatactttgttccacgctcctgctactgactgtcccgtcctgggtcttatcccaggtcaccttgagggagtctggtcctgcgctgg
V2-70_04        atggacatactttgttccatgctcctgctactgactgtcccgtcctgggtcttatcccaggtcaccttgagggagtctggtcctgcgctgg
V2-70_05        atggacatactttgttccatgctcctgctactgactgtcccgtcctgggtcttatcccaggtcaccttgagggagtctggtcctgcgctgg
V2-70_06        atggacatactttgttccatgctcctgctactgactgtcccgtcctgggtcttatcccaggtcaccttgagggagtctggtcctgcgctgg
V2-70_07        atggacatactttgttccatgctcctgctactgactgtcccgtcctgggtcttatcccaggtcaccttgagggagtctggtcctgcgctgg
V2-70_08        atggacatactttgttccatgctcctgctactgactgtcccgtcctgggtcttatcccaggtcaccttgagggagtctggtcctgcgctgg
V2-70_09        atggacatactttgttccatgctcctgctactgactgtcccgtcctgggtcttatcccaggtcaccttgagggagtctggtcctgcgctgg
V2-70_10        atggacatactttgttccacgctcctgctactgactgtcccgtcctgggtcttatcccaggtcaccttgagggagtctggtcctgcgctgg
V2-70_11        atggacatactttgttccacgctcctgctactgactgtcccgtcctgggtcttatcccaggtcaccttgagggagtctggtcctgcgctgg
V2-70_12        atggacatactttgttccacgctcctgctactgactgtcccgtcctgggtcttatcccaggtcaccttgagggagtctggtcctgcgctgg
IGHV2-70*01     atggacatactttgttccacgctcctgctactgactgtcccgtcctgggtcttatcccaggtcaccttgagggagtctggtcctgcgctgg
IGHV2-70*02     atggacatactttgttccacgctcctgctactgactgtcccgtcctgggtcttatcccaggtcaccttgagggagtctggtcctgcgctgg
IGHV2-70*03     atggacatactttgttccacgctcctgctactgactgtcccgtcctgggtcttatcccaggtcaccttgaaggagtctggtcctgcgctgg
IGHV2-70*04     atggacatactttgttccacgctcctgctactgactgtcccgtcctgggtcttatcccaggtcaccttgaaggagtctggtcctgcgctgg
IGHV2-70*06     atggacatactttgttccacgctcctgctactgactgtcccgtcctgggtcttatcccaggtcaccttgaaggagtctggtcctgcgctgg
IGHV2-70*07     atggacatactttgttccacgctcctgctactgactgtcccgtcctgggtcttatcccaggtcaccttgagggagtctggtcctgcgctgg
IGHV2-70*08     atggacatactttgttccacgctcctgctactgactgtcccgtcctgggtcttatcccaggtcaccttgagggagtctggtcctgcgctgg
IGHV2-70*09     atggacatactttgttccacgctcctgctgctgaccatccctcatgggtcttgtctcagatcaccttgaaggagtctggtcctacgctgg
IGHV2-70*10     atggacatactttgttccacgctcctgctgctgaccatccctcatgggtcttatcccaggtcaccttgagggagtctggtcctgcgctgg
IGHV2-70*11     atggacatactttgttccacgctcctgctactgactgtcccgtcctgggtcttatcccgggtcaccttgagggagtctggtcctgcgctgg
IGHV2-70*12     atggacatactttgttccacgctcctgctactgactgtcccgtcctgggtcttgtcccagatcaccttgaaggagtctggtcctacgctgg
IGHV2-70*13     atggacatactttgttccacgctcctgctactgactgtcccgtcctgggtcttatcccaggtcaccttgagggagtctggtcctgcgctgg
IGHV2-70*15     atggacatactttgttccacgctcctgctactgactgtcccgtcctgggtcttatcccaggtcaccttgagggagtctggtcctgcgctgg
IGHV2-70*16     atggacatactttgttccacgctcctgctactgactgtcccgtcctgggtcttatcccaggtcaccttgaaggagtctggtcctgtgctgg
IGHV2-70*17     atggacatactttgttccacgctcctgctactgactgtcccgtcctgggtcttatcccaggtcaccttgagggagtctggtcctgcgctgg
IGHV2-70*18     atggacatactttgttccacgctcctgctactgactgtcccgtcctgggtcttatcccaggtcaccttgagggagtctggtcctgcgctgg
IGHV2-70*19     atggacatactttgttccacgctcctgctactgactgtcccgtcctgggtcttatcccaggtcaccttgagggagtctggtcctgcgctgg
IGHV2-70D*04    atggacatactttgttccacgctcctgctactgactgtcccgtcctgggtcttatcccaggtcaccttgaaggagtctggtcctgcgctgg
IGHV2-70D*14    atggacatactttgttccacgctcctgctactgactgtcccgtcctgggtcttatcccaggtcaccttgaaggagtctggtcctgcgctgg
IP_V2-70*p14    ---------------------------------------------------------caggtcaccttgaaggagtctggtcctgcgctgg
IP_V2-70*p15    -------------------------------------------------------------------------------------------
IP_V2-70*p16    ---------------------------------------------------------caggtcaccttgagggagtctggtcctgcgctgg
IP_V2-70*p17    ---------------------------------------------------------caggtcaccttgaaggagtctggtcctgcgctgg
IP_V2-70*p18    ---------------------------------------------------------caggtcaccttgagggagtctggtcctgcgctgg
IP_V2-70*p19    ---------------------------------------------------------caggtcaccttgaaggagtctggtcctgtgctgg
IP_V2-70*p20    ---------------------------------------------------------caggtcaccttgaaggagtctggtcctgtgctgg
IP_V2-70*p21    ---------------------------------------------------------caggtcaccttgaaggagtctggtcctgcgctgg
IP_V2-70*p22    ---------------------------------------------------------caggtcaccttgagggagtctggtcctgcgctgg
IP_V2-70*p23    ---------------------------------------------------------caggtcaccttgaaggagtctggtcctgcgctgg
IP_V2-70*p24    ---------------------------------------------------------caggtcaccttgaaggagtctggtcctgcgctgg
IP_V2-70*p25    ---------------------------------------------------------caggtcaccttgaaggagtctggtcctgcgctgg
IP_V2-70*p26    ---------------------------------------------------------cagatcaccttgaaggagtctggtcctacgctgg
IP_V2-70*p27    ---------------------------------------------------------cagatcaccttgaaggagtctggtcctacgctgg
```

```
IP_V2-70*p28     ---------------------------------------------------------caggtcaccttgaaggagtctggtcctgcgctgg
IP_V2-70*p29     ---------------------------------------------------------caggtcaccttgaaggagtctggtcctgcgctgg
V_V2-70*01       ---------------------------------------------------------caggtcaccttgagggagtctggtcctgcgctgg
V_V2-70*13       ---------------------------------------------------------caggtcaccttgagggagtctggtcctgcgctgg


V2-70_01         tgaaacccacacagaccctcacactgacctgcaccttctctgggttctcactcagcactagtggaatgtgtgtgagctggatccgtcag
V2-70_02         tgaaacccacacagaccctcacactgacctgcaccttctctgggttctcactcagcactagtggaatgtgtgtgagctggatccgtcag
V2-70_03         tgaaacccacacagaccctcacactgacctgcaccttctctgggttctcactcagcactagtggaatgtgtgtgagctgggtccgtcag
V2-70_04         tgaaacccacacagaccctcacgctgacctgcaccttctctgggttctcactcagcactagtggaatgcgtgtgagctggatccgtcag
V2-70_05         tgaaacccacacagaccctcacactgacctgcaccttctctgggttctcactcagcactagtggaatgcgtgtgagctggatccgtcag
V2-70_06         tgaaacccacacagaccctcacactgacctgcaccttctctgggttctcactcagcactagtggaatgtgtgtgagctggatccgtcag
V2-70_07         tgaaacccacacagaccctcacgctgacctgcaccttctctgggttctcactcagcactagtggaatgcgtgtgagctggatccgtcag
V2-70_08         tgaaacccacacagaccctcacactgacctgcaccttctctgggttctcactcagcactagtggaatgcgtgtgagctggatccgtcag
V2-70_09         tgaaacccacacagaccctcacactgacctgcaccttctctgggttctcactcagcactagtggaatgtgtgtgagctggatccgtcag
V2-70_10         tgaaacccacacagaccctcacactgacctgcaccttctctgggttctcactcagcactagtggaatgtgtgtgagctggatccgtcag
V2-70_11         tgaaacccacacagaccctcacactgacctgcaccttctctgggttctcactcagcactagtggaatgtgtgtgagctggatccgtcag
V2-70_12         tgaaacccacacagaccctcacactgacctgcaccttctctgggttctcactcagcactagtggaatgtgtgtgagctggatccgtcag
IGHV2-70*01      tgaaacccacacagaccctcacactgacctgcaccttctctgggttctcactcagcactagtggaatgtgtgtgagctggatccgtcag
IGHV2-70*02      tgaaacccacacagaccctcacactgacctgcaccttctctgggttctcactcagcactagtggaatgtgtgtgagctggatccgtcag
IGHV2-70*03      tgaaacccacacagaccctcacactgacctgcaccttctctgggttctcactcagcactagtggaatgcgtgtgagctggatccgtcag
IGHV2-70*04      tgaaacccacacagaccctcacactgacctgcaccttctctgggttctcactcagcactagtggaatgcgtgtgagctggatccgtcag
IGHV2-70*06      tgaaacccacacagaccctcacactgacctgcaccttctctgggttctcactcagcactagtggaatgcgtgtgagctggatccgtcag
IGHV2-70*07      tgaaacccacacagaccctcacactgacctgcaccttctctgggttctcactcagcactagtggaatgtgtgtgagctggatccgtcag
IGHV2-70*08      tgaaacccacacagaccctcacactgacctgcgccttctctgggttctcactcagcactagtggaatgtgtgtgagctggatccgtcag
IGHV2-70*09      tgaaacccacacagaccctcacgctgacccgcaccttctctgggttctcactcagcactagtggaatgtgtgtgagctggatccgtcag
IGHV2-70*10      tgaaacccacacagaccctcacactgacctgcaccttctctgggttctcactcagcactagtggaatgcgtgtgagctggatccgtcag
IGHV2-70*11      tgaaacccacacagaccctcacactgacctgcaccttctctgggttctcactcagcactagtggaatgtgtgtgagctggatccgtcag
IGHV2-70*12      tgaaacccacacagaccctcacgctgacctgcaccttctctgggttctcactcagcactagtggaatgtgtgtgagctggatccgtcag
IGHV2-70*13      tgaaacccacacagaccctcacactgacctgcaccttctctgggttctcactcagcactagtggaatgtgtgtgagctggatccgtcag
IGHV2-70*15      tgaaacccacacagaccctcacactgacctgcaccttctctgggttctcactcagcactagtggaatgtgtgtgagctggatccgtcag
IGHV2-70*16      tgaaacccacacagaccctcacactgacctgcaccttctctgggttctcactcagcactagtggaatgtgtgtgagctggatccgtcag
IGHV2-70*17      tgaaacccacacagaccctcacactgacctgcaccttctctgggttctcactcagcactagtggaatgtgtgtgagctggatccgtcag
IGHV2-70*18      tgaaacccacacagaccctcaccctgacctgcaccttctctgggttctcactcagcactagtgaaatgtgtgtgagctgggtccgtcag
IGHV2-70*19      tgaaacccacacagaccctcacactgacctgcaccttctctgggttctcactcagcactagtggaatgtgtgtgagctgggtccgtcag
IGHV2-70D*04     tgaaacccacacagaccctcacactgacctgcaccttctctgggttctcactcagcactagtggaatgcgtgtgagctggatccgtcag
IGHV2-70D*14     tgaaacccacacagaccctcacactgacctgcaccttctctgggttctcactcagcactagtggaatgcgtgtgagctggatccgtcag
IP_V2-70*p14     tgaaacccacacagaccctcacactgacctgcaccttctctgggttctcactcagcactagtggaatgcgtgtgagctggatccgtcag
IP_V2-70*p15     -----------------------------------------------------------------------------------------
IP_V2-70*p16     tgaaacccacacagaccctcacactgacctgcaccttctctgggttctcactcagcactagtggaatgtgtgtgagctgggtccgtcag
IP_V2-70*p17     tgaaacccacacagaccctcacactgacctgcaccttctctgggttctcactcagcactagtggaatgtgtgtgagctgggtccgtcag
IP_V2-70*p18     tgaaacccacacagaccctcacactgacctgcaccttctctgggttctcactcagcactagtggaatgtgtgtgagctggatccgtcag
IP_V2-70*p19     tgaaacccacacagaccctcacactgacctgcaccttctctgggttctcactcagcactagtggaatgtgtgtgagctggatccgtcag
IP_V2-70*p20     tgaaacccacacagaccctcacactgacctgcaccttctctgggttctcactcagcactagtggaatgtgtgtgagctggatccgtcag
IP_V2-70*p21     tgaaacccacacagaccctcacactgacctgcaccttctctgggttctcactcagcactagtggaatgtgtgtgagctggatccgtcag
IP_V2-70*p22     tgaaacccacacagaccctcacactgacctgcaccttctctgggttctcactcagcactagtggaatgtgtgtgagctggatccgtcag
IP_V2-70*p23     tgaaacccacacagaccctcacactgacctgcaccttctctgggttctcactcagcactagtggaatgcgtgtgagctggatccgtcag
```

```
IP_V2-70*p24    tgaaacccacacagaccctcacgctgacctgcaccttctctgggttctcactcagcactagtggaatgcgtgtgagctggatccgtcag
IP_V2-70*p25    tgaaacccacacagaccctcacgctgacctgcaccttctctgggttctcactcagcactagtggaatgcgtgtgagctggatccgtcag
IP_V2-70*p26    tgaaacccacacagaccctcacgctgacctgcaccttctctgggttctcactcagcactagtggaatgcgtgtgagctggatccgtcag
IP_V2-70*p27    tgaaacccacacagaccctcacgctgacctgcaccttctctgggttctcactcagcactagtggaatgcgtgtgtgctggatccgtcag
IP_V2-70*p28    tgaaacccacacagaccctcacgctgacctgcaccttctctgggttctcactcagcactagtggaatgcgtgtgtgctggatccgtcag
IP_V2-70*p29    tgaaacccacacagaccctcacgctgacctgcaccttctctgggttctcactcagcactagtggaatgcgtgtgagctggatccgtcag
V_V2-70*01      tgaaacccacacagaccctcacactgacctgcaccttctctgggttctcactcagcactagtggaatgtgtgtgagctggatccgtcag
V_V2-70*13      tgaaacccacacagaccctcacactgacctgcaccttctctgggttctcactcagcactagtggaatgtgtgtgagctggatccgtcag
```

```
V2-70_01        cccccagggaaggccctggagtggcttgcactcattgattgggatgatgataaatactacagcacatctctgaagaccaggctcaccatct
V2-70_02        cccccagggaaggccctggagtggcttgcacgcattgattgggatgatgataaatactacagcacatctctgaagaccaggctcaccatct
V2-70_03        cccccagggaaggccctggagtggcttgcactcattgattgggatgatgataaatactacagcacatctctgaagaccaggctcaccatct
V2-70_04        cccccagggaaggccctggagtggcttgcacgcattgattgggatgatgataaatactgcagcacatctctgaagaccaggctcaccatct
V2-70_05        cccccagggaaggccctggagtggcttgcacgcattgattgggatgatgataaatactgcagcacatctctgaagaccaggctcaccatct
V2-70_06        cccccagggaaggccctggagtggcttgcacgcattgattgggatgatgataaatactgcagcacatctctgaagaccaggctcaccatct
V2-70_07        cccccagggaaggccctggagtggcttgcacgcattgattgggatgatgataaatactgcagcacatctctgaagaccaggctcaccatct
V2-70_08        cccccagggaaggccctggagtggcttgcacgcattgattgggatgatgataaatactgcagcacatctctgaagaccaggctcaccatct
V2-70_09        cccccagggaaggccctggagtggcttgcacgcattgattgggatgatgataaatactgcagcacatctctgaagaccaggctcaccatct
V2-70_10        cccccagggaaggccctggagtggcttgcacgcattgattgggatgatgataaatactgcagcacatctctgaagaccaggctcaccatct
V2-70_11        cccccagggaaggccctggagtggcttgcacgcattgattgggatgatgataaatactgcagcacatctctgaagaccaggctcaccatct
V2-70_12        cccccagggaaggccctggagtggcttgcacgcattgattgggatgatgataaatactacaacacatctctgaagaccaggctcaccatct
IGHV2-70*01     cccccagggaaggccctggagtggcttgcactcattgattgggatgatgataaatactacagcacatctctgaagaccaggctcaccatct
IGHV2-70*02     cccccagggaaggccctggagtggcttgcactcattgattgggatgatgataaatactacagcacatctctgaagaccaggctcaccatct
IGHV2-70*03     cccccagggaaggccctggagtggcttgcacgcattgattgggatgatgataaattctacagcacatctctgaagaccaggctcaccatct
IGHV2-70*04     cccccagggaaggccctggagtggcttgcacgcattgattgggatgatgataaattctacagcacatctctgaagaccaggctcaccatct
IGHV2-70*06     cccccagggaaggccctggagtggcttgcacgcattgattgggatgatgataaattctacagcacatccctgaagaccaggctcaccatct
IGHV2-70*07     cccccggggaaggccctggagtggcttgcactcattgattgggatgatgataaatactacagcacatctctgaagaccaggctcaccatct
IGHV2-70*08     cccccagggaaggccctggagtggcttgcacgcattgattgggatgatgataaatactacagcacatctctgaagaccaggctcaccatct
IGHV2-70*09     cccccagggaaggccctggagtggcttgcactcattgattgggatgatgataaatactacagcacatctctgaacaccaggctcaccatct
IGHV2-70*10     cccccagggaaggccctggagtggattgcacgcattgattgggatgatgataaatactacagcacatctctgaagaccaggctcaccatct
IGHV2-70*11     cccccagggaaggccctggagtggcttgcacgcattgattgggatgatgataaatactacagcacatctctgaagaccaggctcaccatct
IGHV2-70*12     cccccagggaaggccctggagtggcttgcactcattgattgggatgatgataaatactacagcacatctctgaagaccaggctcaccatct
IGHV2-70*13     cccccagggaaggccctggagtggcttgcactcattgattgggatgatgataaatactacagcacatctctgaagaccaggctcaccatct
IGHV2-70*15     cccccagggaaggccctggagtggcttgcacgcattgattgggatgatgataaatactacagcacatctctgaagaccaggctcaccatct
IGHV2-70*16     cccccagggaaggccctggagtggcttgcacgcattgattgggatgatgataaattctacagcacatctctgaagaccaggctcaccatct
IGHV2-70*17     cccccagggaaggccctggagtggcttgcacgcattgattgggatgatgataaattctacagcacatctctgaagaccaggctcaccatct
IGHV2-70*18     cccccagggaaggccctggagtggcttgcactcattgattgggatgatgataaatactacagcacatctctgaagaccaggctcaccatct
IGHV2-70*19     cccccagggaaggccctggagtggcttgcactcattgattgggatgatgataaacactacagcacatctctgaagaccaggctcaccatct
IGHV2-70D*04    cccccagggaaggccctggagtggcttgcacgcattgattgggatgatgataaattctacagcacatctctgaagaccaggctcaccatct
IGHV2-70D*14    cccccaggtaaggccctggagtggcttgcacgcattgattgggatgatgataaattctacagcacatctctgaagaccaggctcaccatct
IP_V2-70*p14    cccccagggaaggccctggagtggcttgcacgcattgattgggatgatgataaattctacagcacatctctgaagaccaggctcaccatct
IP_V2-70*p15    -----------ccctggagtggcttgcgctcattgattgggatgatgataaatactacagcacatctctgaagaccaggctcaccatct
IP_V2-70*p16    cccccagggaaggccctggagtggcttgcactcattgattgggatgatgataaatactacagcacatctctgaagaccaggctcaccatct
IP_V2-70*p17    cccccagggaaggccctggagtggcttgcactcattgattgggatgatgataaatactacagcacatctctgaagaccaggctcaccatct
```

```
IP_V2-70*p18    cccccagggaaggccctggagtggcttgcacgcattgattggatgatgataaatactacagcacatctctgaagaccaggctcaccatct
IP_V2-70*p19    cccccagggaaggccctggagtggcttgcacgcattgattggatgatgataaatactacagcacatctctgaagaccaggctcaccatct
IP_V2-70*p20    cccccagggaaggccctggagtggcttgcacgcattgattggatgatgataaattctacagcacatctctgaagaccaggctcaccatct
IP_V2-70*p21    cccccagggaaggccctggagtggcttgcacgcattgattggatgatgataaatactacagcacatctctgaagaccaggctcaccatct
IP_V2-70*p22    cccccagggaaggccctggagtggcttgcacgcattgattggatgatgataaattctacagcacatctctgaagaccaggctcaccatct
IP_V2-70*p23    cccccagggaaggccctggagtggcttgcacgcattgattggatgatgataaatactacagcacatctctgaagaccaggctcaccatct
IP_V2-70*p24    cccccagggaaggccctggagtggcttgcacgcattgattggatgatgataaatactgcagcacatctctgaagaccaggctcaccatct
IP_V2-70*p25    cccccagggaaggccctggagtggcttgcacgcattgattggatgatgataaatactgcagcacatctctgaagaccaggctcaccatct
IP_V2-70*p26    cccccagggaaggccctggagtggcttgcacgcattgattggatgatgataaatactgcagcacatctctgaagaccaggctcaccatct
IP_V2-70*p27    cccccagggaaggccctggagtggcttgcacgcattgattggatgatgataaatactgcagcacatctctgaagaccaggctcaccatct
IP_V2-70*p28    cccccagggaaggccctggagtggcttgcacgcattgattggatgatgataaatactgcagcacatctctgaagaccaggctcaccatct
IP_V2-70*p29    cccccagggaaggccctggagtggcttgcacgcattgattggatgatgataaattctacagcacatctctgaagaccaggctcaccatct
V_V2-70*01      cccccagggaaggccctggagtggcttgcactcattgattggatgatgataaatactacagcacatctctgaagaccaggctcaccatct
V_V2-70*13      cccccagggaaggccctggagtggcttgcactcattgattggatgatgataaatactacagcacatctctgaagaccaggctcaccatct

V2-70_01        ccaaggacacctccaaaaaccaggtggtccttacaatgaccaacatggaccctgtggacacagccacgtattattgtgcacggata-
V2-70_02        ccaaggacacctccaaaaaccaggtggtccttacaatgaccaacatggaccctgtggacacagccacgtattattgtgcacggata-
V2-70_03        ccaaggacacctccaaaaaccaggtggtccttacaatgaccaacatggaccctgtggacacagccacgtattattgtgcacggata-
V2-70_04        ccaaggacacctccaaaaaccaggtggtccttacaatgaccaacatggaccctgtggacacagccacgtattattgtgcacggata-
V2-70_05        ccaaggccacctccaaaaaccaggtggtccttacaatgaccaacatggaccctgtggacacagccacgtattattgtgcacggata-
V2-70_06        ccaaggccacctccaaaaaccaggtggtccttacaatgaccaacatggaccctgtggacacagccacgtattattgtgcacggata-
V2-70_07        ccaaggccacctccaaaaaccaggtggtccttacaatgaccaacatggaccctgtggacacagccacgtattattgtgcacggata-
V2-70_08        ccaaggacacctccaaaaaccaggtggtccttacaatgaccaacatggaccctgtggacacagccacgtattattgtgcacggata-
V2-70_09        ccaaggacacctccaaaaaccaggtggtccttacaatgaccaacatggaccctgtggacacagccacgtattattgtgcacggata-
V2-70_10        ccaaggccacctccaaaaaccaggtggtccttacaatgaccaacatggaccctgtggacacagccacgtattattgtgcacggata-
V2-70_11        ccaaggacacctccaaaaaccaggtggtccttacaatgaccaacatggaccctgtggacacagccacgtattattgtgcacggata-
V2-70_12        ccaaggacacctccaaaaaccaggtggtccttacaatgaccaacatggaccctgtggacacagccacgtattattgtgcacggata-
IGHV2-70*01     ccaaggacacctccaaaaaccaggtggtccttacaatgaccaacatggaccctgtggacacagccacgtattactgtgcacggatac
IGHV2-70*02     ccaaggacacctccaaaaaccaggtggtccttacaatgaccaacatggaccctgtggacacggccgtgtattactg-----------
IGHV2-70*03     ccaaggacacctccaaaaaccaggtggtccttacaatgaccaacatggaccctgtggacacggccgtgtattactg-----------
IGHV2-70*04     ccaaggacacctccaaaaaccaggtggtccttacaatgaccaacatggaccctgtggacacagccacgtattactgtgcacggatac
IGHV2-70*06     ccaaggacacctccaaaaaccaggtggtccttacaatgaccaacatggaccctgtggacacggccgtgtattactg-----------
IGHV2-70*07     ccaaggacacctccaaaaaccaggtggtccttacaatgaccaacatggaccctgtggacacggccgtgtattactg-----------
IGHV2-70*08     ccaaggacacctccaaaaaccaggtggtccttacaatgaccaacatggaccctgtggacacggccgtgtattactg-----------
IGHV2-70*09     ccaaggacacctccaaaaaccaggtggtccttacaatgaccaacatggaccctgtggacacaggcacatattactgtgtacgg----
IGHV2-70*10     ccaaggacacctccaaaaaccaggtggtccttacaatgaccaacatggaccctgtggacacagccacgtattactgtgcacggatac
IGHV2-70*11     ccaaggacacctccaaaaaccaggtggtccttacaatgaccaacatggaccctgtggacacagccacgtattactgtgcacggatac
IGHV2-70*12     ccaaggacacctccaaaaaccaggtggtccttacaatgaccaacatggaccctgtggacacagccacatattactgtgcacacagac
IGHV2-70*13     ccaaggacacctccaaaaaccaggtggtccttacaatgaccaacatggaccctgtggacacagccacgtattattgtgcacggatac
IGHV2-70*15     ccaaggacacctccaaaaaccaggtggtccttacaatgaccaacatggaccctgtggacacagccacgtattactgtgcacggatac
IGHV2-70*16     ccaaggacacctccaaaaaccaggtggtccttacaatgaccaacatggaccctgtggacacagccacgtattactgtgcacggatac
IGHV2-70*17     ccaaggacacctccaaaaaccaggtggtccttacaatgaccaacatggaccctgtggacacagccacgtattactgtgcacggatac
IGHV2-70*18     ccaaggacacctccaaaaaccaggtggtccttacaatgaccaacatggaccctgtggacacagccacgtattactgtgcacggatac
IGHV2-70*19     ccaaggacacctccaaaaaccaggtggtccttacaatgaccaacatggaccctgtggacacagccacgtattactgtgcacggatac
IGHV2-70D*04    ccaaggacacctccaaaaaccaggtggtccttacaatgaccaacatggaccctgtggacacagccacgtattactgtgcacggatac
IGHV2-70D*14    ccaaggacacctccaaaaaccaggtggtccttacaatgaccaacatggaccctgtggacacagccacgtattactgtgcacggatac
```

```
IP_V2-70*p14    ccaaggacacctccaaaaaccaggtggtccttacaatgaccaacatggaccctgtggacacagccacgtattactgtgcacggatac
IP_V2-70*p15    ccaaggacacctccaaaaaccaggtggtccttacaatgaccaacatggaccctgtggacacagccacgtattactgtgcacggatac
IP_V2-70*p16    ccaaggacacctccaaaaaccaggtggtccttacaatgaccaacatggaccctgtggacacagccacgtattactgtgcacggat--
IP_V2-70*p17    ccaaggacacctccaaaaaccaggtggtccttacaatgaccaacatggaccctgtggacacagccacgtattactgtgcacggat--
IP_V2-70*p18    ccaaggacacctccaaaaaccaggtggtccttacaatgaccaacatggaccctgtggacacagccacgtattactgtgcacgga---
IP_V2-70*p19    ccaaggacacctccaaaaaccaggtggtccttacaatgaccaacatggaccctgtggacacagccacgtattactgtgcacggat--
IP_V2-70*p20    ccaaggacacctccaaaaaccaggtggtccttacaatgaccaacatggaccctgtggacacagccacgtattactgtgcacggat--
IP_V2-70*p21    ccaaggacacctccaaaaaccaggtggtccttacaatgaccaacatggaccctgtggacacagccacgtattactgtgcacgga---
IP_V2-70*p22    ccaaggacacctccaaaaaccaggtggtccttacaatgaccaacatggaccctgtggacacagccacgtattactgtgcacggat--
IP_V2-70*p23    ccaaggacacctccaaaaaccaggtggtccttacaatgaccaacatggaccctgtggacacagccacgtattactgtgcacggat--
IP_V2-70*p24    ccaaggccacctccaaaaaccaggtggtccttacaatgaccaacatggaccctgtggacacagccacgtattactgtgcacggat--
IP_V2-70*p25    ccaaggacacctccaaaaaccaggtggtccttacaatgaccaacatggaccctgtggacacagccacgtattactgtgcacggat--
IP_V2-70*p26    ccaaggccacctccaaaaaccaggtggtccttacaatgaccaacatggaccctgtggacacagccacgtattactgtgcacggat--
IP_V2-70*p27    ccaaggccacctccaaaaaccaggtggtccttacaatgaccaacatggaccctgtggacacagccacgtattactgtgcacggat--
IP_V2-70*p28    ccaaggccacctccaaaaaccaggtggtccttacaatgaccaacatggaccctgtggacacagccacgtattactgtgcacggat--
IP_V2-70*p29    ccaaggacacctccaaaaaccaggtggtccttacaatgaccaacatggaccctgtggacacagccacgtattactgtgcacggat--
V_V2-70*01      ccaaggacacctccaaaaaccaggtggtccttacaatgaccaacatggaccctgtggacacagccacgtattactgtgcacgg----
V_V2-70*13      ccaaggacacctccaaaaaccaggtggtccttacaatgaccaacatggaccctgtggacacagccacgtattattgtgcacgg----
```

IGHV3-7

V3-7_01	atggaattggggctgagctgggttttccttgttgctattttagaaggtgtccagtgtgaggtgcagctggtggagtctgggggaggcttggtc
V3-7_02	atggagttggggctgagctgggttttccttgttgctattttagaaggtgtccagtgtgaggtgcagctggtggagtctgggggaggcttggtc
V3-7_03	atggagttggggctgagctgggttttccttgttgctattttagaaggtgtccagtgtgaggtgcagctggtggagtctgggggaggcttggtc
V3-7_04	atggagttggggctgagctgggttttccttgttgctattttagaaggtgtccagtgtgaggtgcagctggtggagtctgggggaggcttggtc
V3-7_05	atggaattggggctgagctgggttttccttgttgctattttagaaggtgtccagtgtgaggtgcagctggtggagtctgggggaggcttggtc
V3-7_06	atggagttggggctgagctgggttttccttgttgctattttagaaggtgtccagtgtgaggtgcagctggtggagtctgggggaggcttggtc
V3-7_07	atggaattggggctgagctgggttttccttgttgctattttagaaggtgtccagtgtgaggtgcagctggtggagtctgggggaggcttggtc
V3-7_08	atggaattggggctgagctgggttttccttgttgctattttagaaggtgtccagtgtgaggtgcagctggtggagtctgggggaggcttggtc
V3-7_09	atggaattggggctgagctgggttttccttgttgctattttagaaggtgtccagtgtgaggtgcggctggtggagtctgggggaggcttggtc
V3-7_10	atggaattggggctgagctgggttttccttgttgctattttagaaggtgtccagtgtgaggtgcagctggtggagtctgggggaggcttggtc
V3-7_11	atggagttggggctgagctgggttttccttgttgctattttagaaggtgtccagtgtgaggtgcagctggtggagtctgggggaggcttggtc
V3-7_12	atggagttggggctgagctgggttttccttgttgctattttagaaggtgtccagtgtgaggtgcggctggtggagtctgggggaggcttggtc
IGHV3-7*01	atggaattggggctgagctgggttttccttgttgctattttagaaggtgtccagtgtgaggtgcagctggtggagtctgggggaggcttggtc
IGHV3-7*02	---gaggtgcagctggtggagtctgggggaggcttggtc
IGHV3-7*03	---gaggtgcagctggtggagtctgggggaggcttggtc
IP_V3-7*p04	---gaggtgcggctggtggagtctgggggaggcttggtc
IP_V3-7*p05	---gaggtgcagctggtggagtctgggggaggcttggtc
V_V3-7*01	---gaggtgcagctggtggagtctgggggaggcttggtc

V3-7_01	cagcctggggggtccctgagactctcctgtgcagcctctggattcacctttagtagctattggatgagctgggtccgccaggctcca
V3-7_02	cagcctggggggtccctgagactctcctgtgcagcctctggattcacctttagtagctattggatgagctgggtccgccaggctcca
V3-7_03	cagcctggggggtccctgagactctcctgtgcagcctctggattcacctttagtagctattggatgagctgggtccgccaggctcca
V3-7_04	cagcctggggggtccctgagactctcctgtgcagcctctggattcacctttagtagctattggatgagctgggtccgccaggctcca
V3-7_05	cagcctggggggtccctgagactctcctgtgcagcctctggattcacctttagtagctattggatgagctgggtccgccaggctcca
V3-7_06	cagcctggggggtccctgagactctcctgtgcagcctctggattcacctttagtagctattggatgagctgggtccgccaggctcca
V3-7_07	cagcctggggggtccctgagactctcctgtgcagcctctggattcacctttagtagctattggatgagctgggtccgccaggctcca
V3-7_08	cagcctggggggtccctgagactctcctgtgcagcctctggattcacctttagtagctattggatgagctgggtccgccaggctcca
V3-7_09	cagcctggggggtccctgagactctcctgtgcagcctctggattcacctttagtagctattggatgagctgggtccgccaggctcca
V3-7_10	cagcctggggggtccctgagactctcctgtgcagcctctggattcacctttagtagctattggatgagctgggtccgccaggctcca
V3-7_11	cagcctggggggtccctgagactctcctgtgcagcctctggattcacctttagtagctattggatgagctgggtccgccaggctcca
V3-7_12	cagcctggggggtccctgagactctcctgtgcagcctctggattcacctttagtagctattggatgagctgggtccgccaggctcca
IGHV3-7*01	cagcctggggggtccctgagactctcctgtgcagcctctggattcacctttagtagctattggatgagctgggtccgccaggctcca
IGHV3-7*02	cagcctggggggtccctgagactctcctgtgcagcctctggattcacctttagtagctattggatgagctgggtccgccaggctcca
IGHV3-7*03	cagcctggggggtccctgagactctcctgtgcagcctctggattcacctttagtagctattggatgagctgggtccgccaggctcca
IP_V3-7*p04	cagcctggggggtccctgagactctcctgtgcagcctctggattcacctttagtagctattggatgagctgggtccgccaggctcca
IP_V3-7*p05	cagcctggggggtccctgagactctcctgtgcagcctctggattcacctttagtagctattggatgagctgggtccgccaggctcca
V_V3-7*01	cagcctggggggtccctgagactctcctgtgcagcctctggattcacctttagtagctattggatgagctgggtccgccaggctcca

```
V3-7_01        gggaagggctggagtgggtggccaacataaagcaagatggaagtgagaaatactatgtggactctgtgaagggccgattcaccatctccaga
V3-7_02        gggaagggctggagtgggtggccaacataaagcaagatggaagtgagaaatactatgtggactctgtgaagggccgattcaccatctccaga
V3-7_03        gggaagggctggagtgggtggccaacataaagcaagatggaagtgagaaatactatgtggactctgtgaagggccgattcaccatctccaga
V3-7_04        gggaaagggctggagtgggtggccaacataaagcaagatggaagtgagaaatactatgtggactctgtgaagggccgattcaccatctccaga
V3-7_05        gggaagggctggagtgggtggccaacataaagcaagatggaagtgagaaatactatgtggactctgtgaagggccgattcaccatctccaga
V3-7_06        gggaaagggctggagtgggtggccaacataaagcaagatggaagtgagaaatactatgtggactctgtgaagggccgattcaccatctccaga
V3-7_07        gggaaagggctggagtgggtggccaacataaagcaagatggaagtgagaaatactatgtggactctgtgaagggccgattcaccatctccaga
V3-7_08        gggaaagggctggagtgggtggccaacataaagcaagatggaagtgagaaatactatgtggactctgtgaagggccgattcaccatctccaga
V3-7_09        gggaagggctggagtgggtggccaacataaagcaagatggaagtgagaaatactatgtggactctgtgaagggccgattcaccatctccaga
V3-7_10        gggaagggctggagtgggtggccaacataaagcaagatggaagtgagaaatactatgtggactctgtgaagggccgattcaccatctccaga
V3-7_11        gggaagggctggagtgggtggccaacataaagcaagatggaagtgagaaatactatgtggactctgtgaagggccgattcaccatctccaga
V3-7_12        gggaagggctggagtgggtggccaacataaagcaagatggaagtgagaaatactatgtggactctgtgaagggccgattcaccatctccaga
IGHV3-7*01     gggaagggctggagtgggtggccaacataaagcaagatggaagtgagaaatactatgtggactctgtgaagggccgattcaccatctccaga
IGHV3-7*02     gggaaagggctggagtgggtggccaacataaagcaagatggaagtgagaaatactatgtggactctgtgaagggccgattcaccatctccaga
IGHV3-7*03     gggaagggctggagtgggtggccaacataaagcaagatggaagtgagaaatactatgtggactctgtgaagggccgattcaccatctccaga
IP_V3-7*p04    gggaagggctggagtgggtggccaacataaagcaagatggaagtgagaaatactatgtggactctgtgaagggccgattcaccatctccaga
IP_V3-7*p05    gggaaagggctggagtgggtggccaacataaagcaagatggaagtgagaaatactatgtggactctgtgaagggccgattcaccatctccaga
V_V3-7*01      gggaagggctggagtgggtggccaacataaagcaagatggaagtgagaaatactatgtggactctgtgaagggccgattcaccatctccaga

V3-7_01        gacaacgccaagaactcactgtatctgcaaatgaacagcctgagagccgaggacacggctgtgtattactgtgcgaga
V3-7_02        gacaacgccaagaactcactgtatctgcaaatgaacagcctgagagccgaggacacggccgtgtattactgtgcgaga
V3-7_03        gacaacgccaagaactcactgtatctgcaaatgaacagcctgagagccgaggacacggctgtgtattactgtgcgaga
V3-7_04        gacaacgccaagaactcactgtatctgcaaatgaacagcctgagagccgaggacacggccgtgtattactgtgcgaga
V3-7_05        gacaacgccaagaactcactgtatctgcaaatgaacagcctgagagccgaggacacggccgtgtattactgtgcgaga
V3-7_06        gacaacgccaagaactcactgtatctgcaaatgaacagcctgagagccgaggacacggctgtgtattactgtgcgagg
V3-7_07        gacaacgccaagaactcactgtatctgcaaatgaacagcctgagagccgaggacacggccgtgtattactgtgcgaga
V3-7_08        gacaacgccaagaactcactgtatctgcaaatgaacagcctgagagccgaggacacggctgtgtattactgtgcgagg
V3-7_09        gacaacgccaagaactcactgtatctgcaaatgaacagcctgagagccgaggacacggctgtgtattactgtgcgaga
V3-7_10        gacaacgccaagaactcactgtatctgcaaatgaacagcctgagagccgaggacacggctgtttattactgtgcgaga
V3-7_11        gacaacgccaagaactcactgtatctgcaaatgaacagcctgagagccgaggacacggctgtttattactgtgcgaga
V3-7_12        gacaacgccaagaactcactgtatctgcaaatgaacagcctgagagccgaggacacggctgtgtattactgtgcgaga
IGHV3-7*01     gacaacgccaagaactcactgtatctgcaaatgaacagcctgagagccgaggacacggctgtgtattactgtgcgaga
IGHV3-7*02     gacaacgccaagaactcactgtatctgcaaatgaacagcctgagagccgaggacacggctgtgtattactgtgcgaga
IGHV3-7*03     gacaacgccaagaactcactgtatctgcaaatgaacagcctgagagccgaggacacggccgtgtattactgtgcgaga
IP_V3-7*p04    gacaacgccaagaactcactgtatctgcaaatgaacagcctgagagccgaggacacggct------------------
IP_V3-7*p05    gacaacgccaagaactcactgtatctgcaaatgaacagcctgagagccgaggacacggcc------------------
V_V3-7*01      gacaacgccaagaactcactgtatctgcaaatgaacagcctgagagccgaggacacggctgtgtattactgtgcgaga
```

IGHV3-9

```
V3-9_01       atggagttgggactgagctggattttccttttggctattttaaaaggtgtccagtgtgaagtgcagctggtggagtctgggggaggcttggt
V3-9_02       atggagttgggactgagctggattttccttttggctattttaaaaggtgtccagtgtgaagtgcagctggtggagtctgggggaggcttggt
V3-9_03       atggagttgggactgagctggattttccttttggctattttaaaaggtgtccagtgtgaagtgcagctggtggagtctgggggaggcttggt
V3-9_04       atggagttgggactgagctggattttccttttggctattttaaaaggtgtccagtgtgaagtgcagctggtggagtctgggggaggcttggt
V3-9_06       atggagttgggactgagctggattttccttttggctattttaaaaggtgtccagtgtgaagtgcagctggtggagtctgggggaggcttggt
V3-9_07       atggagttgggactgagctggattttccttttggctattttaaaaggtgtccagtgtgaagtgcagctggtggagtctgggggaggcttggt
IGHV3-9*01    atggagttgggactgagctggattttccttttggctattttaaaaggtgtccagtgtgaagtgcagctggtggagtctgggggaggcttggt
IGHV3-9*03    atggagttgggactgagctggattttccttttggctattttaaaaggtgtccagtgtgaagtgcagctggtggagtctgggggaggcttggt
IGHV3-9*02    ---------------------------------------------------------gaagtgcagctggtggagtctgggggaggcttggt
IP_V3-9*p04   ---------------------------------------------------------gaagtgcagctggtggagtctgggggaggcttggt
V_V3-9*01     ---------------------------------------------------------gaagtgcagctggtggagtctgggggaggcttggt

V3-9_01       acagcctggcaggtccctgagactctcctgtgcagcctctggattcacctttgatgattatgccatgcactgggtccggcaagctcca
V3-9_02       acagcctggcaggtccctgagactctcctgtgcagcctctggattcacctttgatgattatgccatgcactgggtccggcaagctcca
V3-9_03       acagcctggcaggtccctgagactctcctgtgcagcctctggattcacctttggtgattatgccatgcactgggtccggcaagctcca
V3-9_04       acagcctgggaggtccctgagactctcctgtgcagcctctggattcacctttgatgattatgccatgcactgggtccggcaagctcca
V3-9_06       acagcctggcaggtccctgagactctcctgtgcagcctctggattcacctttgatgattatgccatgcactgggtccggcaagctcca
V3-9_07       acagcctggcaggtccctgagactctcctgtgcagcctctggattcacctttgataattatgccatgcactgggtccggcaagctcca
IGHV3-9*01    acagcctggcaggtccctgagactctcctgtgcagcctctggattcacctttgatgattatgccatgcactgggtccggcaagctcca
IGHV3-9*03    acagcctggcaggtccctgagactctcctgtgcagcctctggattcacctttgatgattatgccatgcactgggtccggcaagctcca
IGHV3-9*02    acagcctggcaggtccctgagactctcctgtgcagcctctggattcacctctgatgattatgccatgcactgggtccggcaagctcca
IP_V3-9*p04   acagcctggcaggtccctgagactctcctgtgcagcctctggattcacctttgatgattatgccatgcactgggtccggcaagctcca
V_V3-9*01     acagcctggcaggtccctgagactctcctgtgcagcctctggattcacctttgatgattatgccatgcactgggtccggcaagctcca

V3-9_01       gggaagggcctggagtgggtctcaggtattagttggaatagtggtagcataggctatgcggactctgtgaagggccgattcaccatctcc
V3-9_02       gggaagggcctggagtgggtctcaggtattagttggaatagtggtagcataggctatgcggactctgtgaagggccgattcaccatctcc
V3-9_03       gggaagggcctggagtgggtctcaggtattagttggaatagtggtagcataggctatgcggactctgtgaagggccgattcaccatctcc
V3-9_04       gggaagggcctggagtgggtctcaggtattagttggaatagtggtagcataggctatgcggactctgtgaagggccgattcaccatctcc
V3-9_06       gggaagggcctggagtgggtctcaggtattagttggaatagtggtagcacaggctatgcggactctgtgaagggccgattcaccatctcc
V3-9_07       gggaagggcctggagtgggtctcaggtattagttggaatagtggtagcataggctatgcggactctgtgaagggccgattcaccatctcc
IGHV3-9*01    gggaagggcctggagtgggtctcaggtattagttggaatagtggtagcataggctatgcggactctgtgaagggccgattcaccatctcc
IGHV3-9*03    gggaagggcctggagtgggtctcaggtattagttggaatagtggtagcataggctatgcggactctgtgaagggccgattcaccatctcc
IGHV3-9*02    gggaagggcctggagtgggtctcaggtattagttggaatagtggtagcataggctatgcggactctgtgaagggccgattcaccatctcc
IP_V3-9*p04   gggaagggcctggagtgggtctcaggtattagttggaatagtggtagcataggctatgcggactctgtgaagggccgattcaccatctcc
V_V3-9*01     gggaagggcctggagtgggtctcaggtattagttggaatagtggtagcataggctatgcggactctgtgaagggccgattcaccatctcc
```

```
V3-9_01        agagacaacgccaagaactccctgtatctgcaaatgaacagtctgagagctgaggacacggccttgtattactgtgcaaaagat
V3-9_02        agagacaacgccaagaactccctgtatctgcaaatgaacagtctgagagctgaggacatggccttgtattactgtgcaaaagat
V3-9_03        agagacaacgccaagaactccctgtatctgcaaatgaacagtctgagagctgaggacacggccttgtattactgtgcaaaagat
V3-9_04        agagacaacgccaagaactccctgtatctgcaaatgaacagtctgagagctgaggacacggccttgtattactgtgcaaaagat
V3-9_06        agagacaacgccaagaactccctgtatctgcaaatgaacagtctgagagctgaggacacggccttgtattactgtgcaaaagat
V3-9_07        agagacaacgccaagaactccctgtatctgcaaatgaacagtctgagagctgaggacacggccttgtattactgtgcaaaagat
IGHV3-9*01     agagacaacgccaagaactccctgtatctgcaaatgaacagtctgagagctgaggacacggccttgtattactgtgcaaaagat
IGHV3-9*03     agagacaacgccaagaactccctgtatctgcaaatgaacagtctgagagctgaggacatggccttgtattactgtgcaaaagat
IGHV3-9*02     agagacaacgccaagaactccctgtatctgcaaatgaacagtctgagagctgaggacacggccttgtattactgtgcaaaagat
IP_V3-9*p04    agagacaacgccaagaactccctgtatctgcaaatgaacagtctgagagctgaggacacggccttgtattactgtgcgaggagat
V_V3-9*01      agagacaacgccaagaactccctgtatctgcaaatgaacagtctgagagctgaggacacggccttgtattactgtgcaaaa---
```

IGHV3-11

```
V3-11_01       atggagtttgggctgagctgggttttccttgttgctatttataaaaggtgtccagtgtcaggtgcag-ctggtggagtctg
V3-11_02       atggagtttgggctgagctgggttttccttgttgctatttataaaaggtgtccagtgtcaggtgcag-ctggtggagtctg
V3-11_03       atggagtttgggctgagctgggttttccttgttgctatttataaaaggtgtccagtgtcaggtgcag-ctggtggagtctg
V3-11_04       atggagtttgggctgagctgggttttccttgttgctatttataaaaggtgtccagtgtcaggtgcag-ctggtggagtctg
V3-11_05       atggagtttgggctgagctgggttttccttgttgctatttataaaaggtgtccagtgtcaggtgcag-ctggtggagtctg
V3-11_06       atggagtttgggctgagccgggttttccttgttgctatttataaaaggtgtccagtgtcaggtgcag-ctggtggagtctg
V3-11_07       atggagtttgggctgagccgggttttccttgttgctatttataaaaggtgtccagtgtcaggtgcag-ctggtggagtctg
IGHV3-11*01    atggagtttgggctgagctgggttttccttgttgctataataaaaggtgtccagtgtcaggtgcag-ctggtggagtctg
IGHV3-11*02    ---------------------------------------attaaagctgtccagtgtcaggtgcagtctggtggagtctg
IGHV3-11*06    atggagtttgggctgagctgggttttccttgttgctatttataaaaggtgtccagtgtcaggtgcag-ctggtggagtctg
IGHV3-11*03    ---------------------------------------------------------caggtgcag-ctgttggagtctg
IGHV3-11*04    ---------------------------------------------------------caggtgcag-ctggtggagtctg
IGHV3-11*05    ---------------------------------------------------------caggtgcag-ctggtggagtctg
IP_V3-11*p04   ---------------------------------------------------------caggtgcag-ctggtggagtctg
IP_V3-11*p05   ---------------------------------------------------------caggtgcag-ctggtggagtctg
IP_V3-11*p07   ---------------------------------------------------------caggtgcag-ctggtggagtctg
V_V3-11*01     ---------------------------------------------------------caggtgcag-ctggtggagtctg
```

```
V3-11_01       ggggaggcttggtcaagcctggagggtccctgagactctcctgtgcagcctctggattcaccttcagtgactactacatgagctgga
V3-11_02       ggggaggcttggtcaagcctggagggtccctgagactctcctgtgcagcctctggattcaccttcagtgactactacatgagctgga
V3-11_03       ggggaggcttggtcaagcctggagggtccctgagactctcctgtgcagcctctggattcaccttcagtgactactacatgagctgga
V3-11_04       ggggaggcttggtcaagcctggagggtccctgagactctcctgtgcagcctctggattcaccttcagtgactactacatgagctgga
V3-11_05       ggggaggcttggtcaagcctggagggtccctgagactctcctgtgcagcctctggattcaccttcagtgattattacatgagctgga
V3-11_06       ggggaggcttggtcaagcctggagggtccctgagactctcctgtgcagcctctggattcaccttcagtgactactacatgagctgga
V3-11_07       ggggaggcttggtcaagcctggagggtccctgagactctcctgtgcagcctctggattcaccttcagtgactactacatgagctgga
IGHV3-11*01    ggggaggcttggtcaagcctggagggtccctgagactctcctgtgcagcctctggattcaccttcagtgactactacatgagctgga
IGHV3-11*02    gggga-cttggtcaagcct-gagggtccctgagactctcctgtgcagcctctggattcaccttcagtgactactacatgagctgga
IGHV3-11*06    ggggaggcttggtcaagcctggagggtccctgagactctcctgtgcagcctctggattcaccttcagtgactactacatgagctgga
IGHV3-11*03    ggggaggcttggtcaagcctggagggtccctgagactctcctgtgcagcctctggattcaccttcagtgactactacatgagctgga
IGHV3-11*04    ggggaggcttggtcaagcctggagggtccctgagactctcctgtgcagcctctggattcaccttcagtgactactacatgagctgga
IGHV3-11*05    ggggaggcttggtcaagcctggagggtccctgagactctcctgtgcagcctctggattcaccttcagtgactactacatgagctgga
IP_V3-11*p04   ggggaggcttggtcaagcctggagggtccctgagactctcctgtgcagcctctggattcaccttcagtgactactacatgagctgga
IP_V3-11*p05   ggggaggcttggtcaagcctggagggtccctgagactctcctgtgcagcctctggattcaccttcagtgactactacatgagctgga
IP_V3-11*p07   ggggaggcttggtcaagcctggagggtccctgagactctcctgtgcagcctctggattcaccttcagtgactactacatgagctgga
V_V3-11*01     ggggaggcttggtcaagcctggagggtccctgagactctcctgtgcagcctctggattcaccttcagtgactactacatgagctgga
```

```
V3-11_01        tccgccaggctccagggaaggggctggagtgggtttcatacattagtagtagtggtagta---ccatatactacgcagaactctgtgaagggcc
V3-11_02        tccgccaggctccagggaaggggctggagtgggtttcatacattagtagtagtggtagta---ccatatactacgcagaactctgtgaagggcc
V3-11_03        tccgccaggctccagggaaggggctggagtgggtttcatacattagtagtagtggtagta---ccatatactacgcagaactctgtgaagggcc
V3-11_04        tccgccaggctccagggaaggggctggagtgggtttcatacattagtagtagtggtagta---ccatatactacgcagaactctgtgaagggcc
V3-11_05        tccgccaggctccagggaaggggctggagtgggtttcatacattagtagtagtggtagta---ccatatactacgcagaactctgtgaagggcc
V3-11_06        tccgccaggctccagggaaggggctggagtgggtttcatacattagtagtagtggtagta---ccatatactacgcagaactctgtgaagggcc
V3-11_07        tccgccaggctccagggaaggggctggagtgggtttcatacattagtagtagtggtagta---ccatatactacgcagaactctgtgaagggcc
IGHV3-11*01     tccgccaggctccagggaaggggctggagtgggtttcatacattagtagtagtggtagta---ccatatactacgcagaactctgtgaagggcc
IGHV3-11*02     tccgccaggctccagggaaggggctggagtgggtttcatacattagtagtagtggtagta---ccatatactacgcag-ctctgtgaagggcc
IGHV3-11*06     tccgccaggctccagggaaggggctggagtgggtttcatacattagtagtagtagtagtt---acacaaactacgcagaactctgtgaagggcc
IGHV3-11*03     tccgccaggctccagggaaggggctggagtgggtttcatacattagtagtagtagtagtt---acacaaactacgcagaactctgtgaagggcc
IGHV3-11*04     tccgccaggctccagggaaggggctggagtgggtttcatacattagtagtagtggtagta---ccatatactacgcagaactctgtgaagggcc
IGHV3-11*05     tccgccaggctccagggaaggggctggagtgggtttcatacattagtagtagtagtagtt---acacaaactacgcagaactctgtgaagggcc
IP_V3-11*p04    tccgccaggctccagggaaggggctggagtgggtttcatacattagtagtagtagtagtt---acacaaactacgcagaactctgtgaagggcc
IP_V3-11*p05    tccgccaggctccagggaaggggctggagtgggtttcatacattagtagtagtagtagtt---acacaaactacgcagaactctgtgaagggcc
IP_V3-11*p07    tccgccaggctccagggaaggggctggagtgggtttcatacattagtagtagtagtagtagttacacaaactacgcagaactctgtgaagggcc
V_V3-11*01      tccgccaggctccagggaaggggctggagtgggtttcatacattagtagtagtggtagta---ccatatactacgcagaactctgtgaagggcc
```

```
V3-11_01        gattcaccatctccagggacaacgccaagaactcactgtatctgcaaatgaacagcctgagagccgaggacacggctgtgtattactgtgcgaga
V3-11_02        gattcaccatctccagggacaacgccaagaactcactgtatctgcaaatgaacagcctgagagccgaggacacggccgtgtattactgtgcgaga
V3-11_03        gattcaccatctccagagacaacgccaagaactcactgtatctgcaaatgaacagcctgagagccgaggacacggctgtgtattactgtgcgaga
V3-11_04        gattcaccatctccagagacaacgccaagaactcactgtatctgcaaatgaacagcctgagagccgaggacacggccgtgtattactgtgcgaga
V3-11_05        gattcaccatctccagagacaacgccaagaactcactgtatctgcaaatgaacagcctgagagccgaggacacggctgtgtattactgtgcgaga
V3-11_06        gattcaccatctccagagacaacgccaagaactcactgtatctgcaaatgaacagcctgagagccgaggacacggctgtgtattactgtgcgaga
V3-11_07        gattcaccatctccagggacaacgccaagaactcactgtatctgcaaatgaacagcctgagagccgaggacacggctgtgtattactgtgcgaga
IGHV3-11*01     gattcaccatctccagggacaacgccaagaactcactgtatctgcaaatgaacagcctgagagccgaggacacggccgtgtattactgtgcgaga
IGHV3-11*02     gattcaccatctccagggagaacgccaagaactcactgtatctgcaaatgaacagcctgagagccgaggacagccttacttact----tgcgaga
IGHV3-11*06     gattcaccatctccagagacaacgccaagaactcactgtatctgcaaatgaacagcctgagagccgaggacacggctgtgtattactgtgcgaga
IGHV3-11*03     gattcaccatctccagagacaacgccaagaactcactgtatctgcaaatgaacagcctgagagccgaggacacggccgtgtattactgtgcgaga
IGHV3-11*04     gattcaccatctccagggacaacgccaagaactcactgtatctgcaaatgaacagcctgagagccgaggacacggctgtgtattactgtgcgaga
IGHV3-11*05     gattcaccatctccagagacaacgccaagaactcactgtatctgcaaatgaacagcctgagagccgaggacacggccgtgtattactgtgcgaga
IP_V3-11*p04    gattcaccatctccagagacaacgccaagaactcactgtatctgcaaatgaacagcctgagagccgaggacacggccgtgtattactgtgcgaga
IP_V3-11*p05    gattcaccatctccagagacaacgccaagaactcactgtatctgcaaatgaacagcctgagagccgaggacacggctgtgtattactgtgcgaga
IP_V3-11*p07    gattcaccatctccagggacaacgccaagaactcactgtatctgcaaatgaacagcctgagagccgaggacacggct------------------
V_V3-11*01      gattcaccatctccagggacaacgccaagaactcactgtatctgcaaatgaacagcctgagagccgaggacacggccgtgtattactgtgcgaga
```

IGHV3-13

```
V3-13_01        atggagttggggctgagctgggttttccttgttgctatattagaaggtgtccagtgtgaggtgcagctggtggagtctgggggaggct
V3-13_02        atggagttggggctgagctgggttttccttgttgctatattagaaggtgtccagtgtgaggtgcagctggtggagtctgggggaggct
V3-13_03        atggagttggggctgagctgggttttccttgttgctatattagaaggtgtccagtgtgaggtgcagctggtggagtctgggggaggct
V3-13_04        atggagttggggctgagctgggttttccttgttgctatattagaaggtgtccagtgtgaggtgcagctggtggagtctgggggaggct
V3-13_05        atggagttggggctgagctgggttttccttgttgctatattagaaggtgtccagtgtgaggtgcagctggtggagtctgggggaggct
V3-13_06        atggagttggggctgagctgggttttccttgttgctatattagaaggtgtccagtgtgaggtgcagctggtggagtctgggggaggct
V3-13_07        atggagttggggctgagctgggttttccttgttgctatattagaaggtgtccagtgtgaggtgcagctggtggagtctgggggaggct
V3-13_08        atggagttggggctgagctgggttttccttgttgctatattagaaggtgtccagtgtgaggtgcagctggtggagtctgggggaggct
V3-13_09        atggagttggggctgagctgggttttccttgttgctatattagaaggtgtccagtgtgaggtgcagctggtggagtctgggggaggct
V3-13_10        atggagttggggctgagctgggttttccttgttgctatattagaaggtgtccagtgtgaggtgcagctggtggagtctgggggaggct
V3-13_11        atggagttggggctgagctgggttttccttgttgctatattagaaggtgtccagtgtgaggtgcagctggtggagtctgggggaggct
V3-13_12        atggagttggggctgagctgggttttccttgttgctatattagaaggtgtccagtgtgaggtgcagctggtggagtctgggggaggct
V3-13_13        atggagttggggctgagctgggttttccttgttgctatattagaaggtgtccagtgtgaggtgcagctggtggagtctgggggaggct
IGHV3-13*01     atggagttggggctgagctgggttttccttgttgctatattagaaggtgtccagtgtgaggtgcagctggtggagtctgggggaggct
IGHV3-13*02     atggagttggggctgagctgggttttccttgttgctatattagaaggtgtccagtgtgaggtgcatctggtggagtctgggggaggct
IGHV3-13*05     atggagttggggctgagctgggttttccttgttgctatattagaaggtgtccagtgtgaggtgcagctggtggagtctgggggaggct
IGHV3-13*03     ---------------------------------------------------------gaggtgcagctggtggagtctgggggaggct
IGHV3-13*04     ---------------------------------------------------------gaggtgcagctggtggagtctgggggaggct
IP_V3-13*p04    ----------------------------------------------------------------------------------------
IP_V3-13*p06    ---------------------------------------------------------gaggtgcagctggtggagtctgggggaggct
V_V3-13*01      ---------------------------------------------------------gaggtgcagctggtggagtctgggggaggct
V_V3-13*02      ---------------------------------------------------------gaggtgcatctggtggagtctgggggaggct

V3-13_01        tggtacagcctggggggtccctgagactctcctgtgcagcctctggattcaccttcagtagctacgacatgcactgggtccgccaagctaca
V3-13_02        tggtacagcctggggggtccctgagactctcctgtgcagcctgtggattcaccttcagtagctacgacatgcactgggtccgccaagctaca
V3-13_03        tggtacagcctggggggtccctgagactctcctgtgcagcctctggattcaccttcagtagctacgacatgcactgggtccgccaagctaca
V3-13_04        tggtacagcctggggggtccctgagactctcctgtgcagcctctggattcaccttcagtagctacgacatgcactgggtccgccaagctaca
V3-13_05        tggtacagcctggggggtccctgagactctcctgtgcagcctctggattcaccttcagtagctacgacatgcactgggtccgccaagctaca
V3-13_06        tggtacagcctggggggtccctgagactctcctgtgcagcctctggattcaccttcagtagctacgacatgcactgggtccgccaagctaca
V3-13_07        tggtacagcctggggggtccctgagactctcctgtgcagcctgtggattcaccttcagtagctacgacatgcactgggtccgccaagctaca
V3-13_08        tggtacagcctggggggtccctgagactctcctgtgcagcctctggattcaccttcagtagctacgacatgcactgggtccgccaagctaca
V3-13_09        tggtacagcctggggggtccctgagactctcctgtgcagcctgtggattcaccttcagtagctacgacatgcactgggtccgccaagctaca
V3-13_10        tggtacagcctggggggtccctgagactctcctgtgcagcctctggattcaccttcagtagctacgacatgcactgggtccgccaagctaca
V3-13_11        tggtacagcctggggggtccctgagactctcctgtgcagcctgtggattcaccttcagtagctacgacatgcactgggtccgccaagctaca
V3-13_12        tggtacagcctggggggtccctgagactctcctgtgcagcctctggattcaccttcagtagctacgacatgcactgggtccgccaagctaca
V3-13_13        tggtacagcctggggggtccctgagactctcctgtgcagcctctggattcaccttcagtagctacgacatgcactgggtccgccaagctaca
IGHV3-13*01     tggtacagcctggggggtccctgagactctcctgtgcagcctctggattcaccttcagtagctacgacatgcactgggtccgccaagctaca
IGHV3-13*02     tggtacagcctgggggggccctgagactctcctgtgcagcctctggattcaccttcagtaactacgacatgcactgggtccgccaagctaca
IGHV3-13*05     tggtacagcctggggggtccctgagactctcctgtgcagcctctggattcaccttcagtagctacgacatgcactgggtccgccaagctaca
IGHV3-13*03     tggtacagcctggggggtccctgagactctcctgtgcagcctgtggattcaccttcagtagctacgacatgcactgggtccgccaagctaca
IGHV3-13*04     tggtacagcctggggggtccctgagactctcctgtgcagcctctggattcaccttcagtagctacgacatgcactgggtccgccaagctaca
IP_V3-13*p04    -----------------------------------gcagcctctggattcaccttcagtagctacgacatgcactgggtccgccaagctaca
IP_V3-13*p06    tggtacagcctggggggtccctgagactctcctgtgcagcctgtggattcaccttcagtagctacgacatgcactgggtccgccaagctaca
V_V3-13*01      tggtacagcctggggggtccctgagactctcctgtgcagcctctggattcaccttcagtagctacgacatgcactgggtccgccaagctaca
V_V3-13*02      tggtacagcctgggggggccctgagactctcctgtgcagcctctggattcaccttcagtaactacgacatgcactgggtccgccaagctaca
```

```
V3-13_01      ggaaaaggtctggagtgggtctcagctattggtactgctggtgacacatactatccaggctccgtgaagggccgattcaccatctccagaga
V3-13_02      ggaaaaggtctggagtgggtctcagctattggtactgctggtgacacatactatccaggctccgtgaagggccaattcaccatctccagaga
V3-13_03      ggaaaaggtctggaatgggtctcagctattggtactgctggtgacacatactatccaggctccgtgaagggccgattcaccatctccagaga
V3-13_04      ggaaaaggtctggagtgggtctcagctattggtactgctggtgaccacatactatccaggctccgtgaagggccgattcaccatctccagaga
V3-13_05      ggaaaaggtctggagtgggtctcagctattggtactgctggtgacacatactatccaggctccgtgaagggccgattcaccatctccagaga
V3-13_06      ggaaaaggtctggaatgggtctcagctattggtactgctggtgaccacatactatccaggctccgtgaagggccgattcaccatctccagaga
V3-13_07      ggaaaaggtctggagtgggtctcagctattggtactgctggtgacacatactatccaggctccgtgaagggccaattcaccatctccagaga
V3-13_08      ggaaaaggtctggagtgggtctcagctattggtactgctggtgaccacatactatccaggctccgtgaagggccgattcaccatctccagaga
V3-13_09      ggaaaaggtctggaatgggtctcagctattggtactgctggtgacacatactatccaggctccgtgaagggccaattcaccatctccagaga
V3-13_10      ggaaaaggtctggagtgggtctcagctattggtactgctggtgacacatactatccaggctctgtgaagggccgattcaccatctccagaga
V3-13_11      ggaaaaggtctggagtgggtctcagctattggtactgctggtgaccacatactatccaggctccgtgaagggccaattcaccatctccagaga
V3-13_12      ggaaaaggtctggagtgggtctcagctattggtactgctggtgacacatactatccaggctccgtgaagggccaattcaccatctccagaga
V3-13_13      ggaaaaggtctggagtgggtctcagctattggtactgctggtgacacatactatccaggctccgtgaagggccgattcaccatctccagaga
IGHV3-13*01   ggaaaaggtctggagtgggtctcagctattggtactgctggtgacacatactatccaggctccgtgaagggccgattcaccatctccagaga
IGHV3-13*02   ggaaaaggtctggagtgggtctcagccaatggtactgctggtgacacatactatccaggctccgtgaagggggcgattcaccatctccagaga
IGHV3-13*05   ggaaaaggtctggagtgggtctcagctattggtactgctggtgaccacatactatccaggctccgtgaagggccgattcaccatctccagaga
IGHV3-13*03   ggaaaaggtctggagtgggtctcagctattggtactgctggtgacacatactatccaggctccgtgaagggccaattcaccatctccagaga
IGHV3-13*04   ggaaaaggtctggaatgggtctcagctattggtactgctggtgacacatactatccaggctccgtgaagggccgattcaccatctccagaga
IP_V3-13*p04  ggaaaaggtctggagtgggtctcagctattggtactgctggtgaccacatactatccaggctccgtgaagggccgattcaccatctccagaga
IP_V3-13*p06  ggaaaaggtctggagtgggtctcagctattggtactgctggtgacacatactatccaggctccgtgaagggccaattcaccatctccagaga
V_V3-13*01    ggaaaaggtctggagtgggtctcagctattggtactgctggtgacacatactatccaggctccgtgaagggccgattcaccatctccagaga
V_V3-13*02    ggaaaaggtctggagtgggtctcagccaatggtactgctggtgacacatactatccaggctccgtgaagggggcgattcaccatctccagaga

V3-13_01      aaatgccaagaactccttgtatcttcaaatgaacagcctgagagccggggacacggctgtgtattactgtgcaaga
V3-13_02      aaatgccaagaactccttgtatcttcaaatgaacagcctgagagccggggacacggctgtgtattactgtgcaaga
V3-13_03      aaatgccaagaactccttgtatcttcaaatgaacagcctgagagccggggacacggctgtgtattactgtgcaaga
V3-13_04      aaatgccaagaactccttgtatcttcaaatgaacagcctgagagccggggacacggctgtgtattactgtgcaaga
V3-13_05      aaatgccaagaactccttgtatcttcaaatgaacagcctgagagccgaggacacggccgtgtattactgtgcaaga
V3-13_06      aaatgccaagaactccttgtatcttcaaatgaacagcctgagagccggggacacggctgtgtattactgtgcaaga
V3-13_07      aaatgccaagaactccttgtatcttcaaatgaacagcctgagagccggggacatggctgtgtattactgtgcaaga
V3-13_08      aaatgccaagaactccttgtatcttcaaatgaacagcctgagagccgaggacacggccgtgtattactgtgcaaga
V3-13_09      aaatgccaagaactccttgtatcttcaaatgaacagcctgagagccggggacacggctgtgtattactgtgcaaga
V3-13_10      aaatgccaagaactccttgtatcttcaaatgaacagcctgagagccggggacacggctgtgtattactgtgcaaga
V3-13_11      aaatgccaagaactccttgtatcttcaaatgaacagcctgagagccggggacacggctgtgtattactgtgcaaga
V3-13_12      aaatgccaagaactccttgtatcttcaaatgaacagcctgagagccggggacacggctgtgtattactgtgcaaga
V3-13_13      aaatgccaagaactccttgtatcttcaaatgaacagcctgagagccgaggacacggctgtgtattactgtgcaaga
IGHV3-13*01   aaatgccaagaactccttgtatcttcaaatgaacagcctgagagccggggacacggctgtgtattactgtgcaaga
IGHV3-13*02   aaatgccaagaactccttgtatcttcaaatgaacagcctgagagccggggacacggctgtgtattactgtgcaaga
IGHV3-13*05   aaatgccaagaactccttgtatcttcaaatgaacagcctgagagccggggacacggctgtgtattactgtgcaaga
IGHV3-13*03   aaatgccaagaactccttgtatcttcaaatgaacagcctgagagccggggacacggctgtgtattactgtgcaaga
IGHV3-13*04   aaatgccaagaactccttgtatcttcaaatgaacagcctgagagccggggacacggctgtgtattactgtgcaaga
IP_V3-13*p04  aaatgccaagaactccttgtatcttcaaatgaacagcctgagagccggggacacggctgtgtattactgtgcaaga
IP_V3-13*p06  aaatgccaagaactccttgtatcttcaaatgaacagcctgagagccggggacatggctgtgtattactgtgcaaga
V_V3-13*01    aaatgccaagaactccttgtatcttcaaatgaacagcctgagagccggggacacggctgtgtattactgtgcaaga
V_V3-13*02    aaatgccaagaactccttgtatcttcaaatgaacagcctgagagccggggacacggctgtgtattactgtgcaaga
```

IGHV3-15

```
V3-15_01        atggagtttgggctgagctggattttccttgctgctattttaaaaggtgtccagtgtgaggtgcagctggtggagtctgggggaggct
V3-15_02        atggagtttgggctgagctggattttccttgctgctattttaaaaggtgtccagtgtgaggtgcagctggtggagtctgggggaggct
V3-15_03        atggagtttgggctgagctggattttccttgctgctattttaaaaggtgtccagtgtgaggtgcagctggtggagtctgggggaggct
V3-15_04        atggagtttgggctgagctggattttccttgctgctattttaaaaggtgtccagtgtgaggtgcagctggtggagtctgggggaggct
V3-15_05        atggagtttgggctgagctggattttccttgctgctattttaaaaggtgtccagtgtgaggtgcagctggtggagtctgggggaggct
V3-15_06        atggagtttgggctgagctggattttccttgctgctattttaaaaggtgtccagtgtgaggtgcagctggtggagtctgggggaggct
V3-15_07        atggagtttgggctgagctggattttccttgctgctattttaaaaggtgtccagtgtgaggtgcagctggtggagtctgggggaggct
V3-15_08        atggagtttgggctgagctggattttccttgctgctattttaaaaggtgtccagtgtgaggtgcagctggtggagtctgggggaggct
V3-15_09        atggagtttgggctgagctggattttccttgctgctattttaaaaggtgtccagtgtgaggtgcagctggtggagtctgggggaggct
IGHV3-15*01     atggagtttgggctgagctggattttccttgctgctattttaaaaggtgtccagtgtgaggtgcagctggtggagtctgggggaggct
IGHV3-15*02     atggagtttgggctgagctggattttccttcctgctattttaaaaggtgtccagtgtgaggtgcagctggtggagtctgggggagcct
IGHV3-15*03     ---------------------------------gctattttaaaaggtgtccagtgtgaggtgcagctggtggagtctgccggagcct
IGHV3-15*04     ---------------------------------gctattttaaaaggtgtccagtgtgaggtgcagctggtggagtctgggggaggct
IGHV3-15*05     ---------------------------------gctattttaaaaggtgtccagtgtgaggtgcagctggtggagtctgggggaggct
IGHV3-15*06     ---------------------------------gctattttaaaaggtgtccagtgtgaggtgcagctggtggagtctgggggaggct
IGHV3-15*07     ---------------------------------gctattttaaaaggtgtccagtgtgaggtgcagctggtggagtctgggggaggct
IGHV3-15*08     ---------------------------------gctattttaaaaggtgtccagtgtgaggtgcagctggtggagtctgcgggaggct
IP_V3-15*p09    ---------------------------------------------------------gaggtgcagctggtggagtctgggggaggct
IP_V3-15*p10    ---------------------------------------------------------gaggtgcagctggtggagtctgggggaggct
V_V3-15*01      ---------------------------------------------------------gaggtgcagctggtggagtctgggggaggct
V_V3-15*02      ---------------------------------------------------------gaggtgcagctggtggagtctgggggagcct

V3-15_01        tggtaaagcctggggggtcccttagactctcctgtgcagcctctggattcactttcagtaacgcctggatgagctgggtccgccaggctcca
V3-15_02        tggtaaagcctggggggtcccttagactctcctgtgcagcctctggtttcactttcagtaacgcctggatgaactgggtccgccaggctcca
V3-15_03        tggtaaagcctggggggtcccttagactctcctgtgcagcctctggtttcactttcagtaacgcctggatgagctgggtccgccaggctcca
V3-15_04        tggtaaagcctggggggtcccttagactctcctgtgcagcctctggattcactttcagtaacgcctggatgaactgggtccgccaggctcca
V3-15_05        tggtaaagcctagggggtcccttagactctcctgtgcagcctctggattcactttcagtaacgcctggatgagctgggtccgccaggctcca
V3-15_06        tggtaaagcctggggggtcccttagactctcctgtgcagcctctggattcactttcagtaacgcctggatgagctgggtccgccaggctcca
V3-15_07        tggtaaagcctggggggtcccttagactctcctgtgcagcctctggtttcactttcagtaacgcctggatgagctgggtccgccaggctcca
V3-15_08        tggtaaagcctggggggtcccttagactctcctgtgcagcctctggtttcactttcagtaacgcctggatgaactgggtccgccaggctcca
V3-15_09        tggtaaagcctggggggtcccttagactctcctgtgcagcctctggattcactttcagtaacgcctggatgagctgggtccgccaggctcca
IGHV3-15*01     tggtaaagcctggggggtcccttagactctcctgtgcagcctctggattcactttcagtaacgcctggatgagctgggtccgccaggctcca
IGHV3-15*02     tggtaaagcctggggggtcccttagactctcctgtgcagcctctggattcactttcagtaacgcctggatgagctgggtccgccaggctcca
IGHV3-15*03     tggtacagcctggggggtcccttagactctcctgtgcagcctctggattcacttgcagtaacgcctggatgagctgggtccgccaggctcca
IGHV3-15*04     tggtaaagcctggggggtcccttagactctcctgtgcagcctctggattcactttcagtaacgcctggatgagctgggtccgccaggctcca
IGHV3-15*05     tggtaaagcctggggggtcccttagactctcctgtgcagcctctggattcactttcagtaacgcctggatgagctgggtccgccaggctcca
IGHV3-15*06     tggtaaagcctggggggtcccttagactctcctgtgcagcctctggattcactttcagtaacgcctggatgagctgggtccgccaggctcca
IGHV3-15*07     tggtaaagcctggggggtcccttagactctcctgtgcagcctctggtttcactttcagtaacgcctggatgaactgggtccgccaggctcca
IGHV3-15*08     tggtacagcctggggggtcccttagactctcctgtgcagcctctggattcacttgcagtaacgcctggatgagctgggtccgccaggctcca
IP_V3-15*p09    tggtaaagcctagggggtcccttagactctcctgtgcagcctctggattcactttcagtaacgcctggatgagctgggtccgccaggctcca
IP_V3-15*p10    tggtaaagcctggggggtcccttagactctcctgtgcagcctctggattcactttcagtaacgcctggatgagctgggtccgccaggctcca
V_V3-15*01      tggtaaagcctggggggtcccttagactctcctgtgcagcctctggattcactttcagtaacgcctggatgagctgggtccgccaggctcca
V_V3-15*02      tggtaaagcctggggggtcccttagactctcctgtgcagcctctggattcactttcagtaacgcctggatgagctgggtccgccaggctcca
```

```
V3-15_01       gggaaggggctggagtgggttggccgtattaaaagcaaaactgatggtgggacaacagactacgctgcacccgtgaaaggcagattcacc
V3-15_02       gggaaggggctggagtgggtcggccgtattaaaagcaaaactgatggtgggacaacagactacgctgcacccgtgaaaggcagattcacc
V3-15_03       gggaaggggctggagtgggtcggccgtattaaaagcaaaactgatggtgggacaacagactacgctgcacccgtgaaaggcagattcacc
V3-15_04       gggaaggggctggagtgggttggccgtattaaaagcaaaactgatggtgggacaacagactacgctgcacccgtgaaaggcagattcacc
V3-15_05       gggaaggggctggagtgggttggccgtattaaaagcaaaactgatggtgggacaacagactacgctgcacccgtgaaaggcagattcacc
V3-15_06       gggaagggactggagtgggttggccgtattaaaagcaaaactgatggtgggacaacagactacgctgcacccgtgaaaggcagattcacc
V3-15_07       gggaaggggctggagtgggttggccgtattaaaagcaaaactgatggtgggacaacagactacgctgcacccgtgaaaggcagattcacc
V3-15_08       gggaaggggctggagtgggttggccgtattaaaagcaaaactgatggtgggacaacagactacgctgcacccgtgaaaggcagattcacc
V3-15_09       gggaaggggctggagtgggttggccgtattaaaagcaaaactgatggtgggacaacagactacgctgcacccgtgaaaggcagattcacc
IGHV3-15*01    gggaaggggctggagtgggttggccgtattaaaagcaaaactgatggtgggacaacagactacgctgcacccgtgaaaggcagattcacc
IGHV3-15*02    gggaaggggctggagtgggttggccgtattaaaagcaaaactgatggtgggacaacagactacgctgcacccgtgaaaggcagattcacc
IGHV3-15*03    gggaaggggctggagtgggttggccgtattaaaagcaaagctaatggtgggacaacagactacgctgcacctgtgaaaggcagattcacc
IGHV3-15*04    gggaaggggctggagtgggttggccgtattgaaagcaaaactgatggtgggacaacagactacgctgcacccgtgaaaggcagattcacc
IGHV3-15*05    gggaaggggctggagtgggttggccgtattaaaagcaaaactgatggtgggacaacagactacgctgcacccgtgaaaggcagattcacc
IGHV3-15*06    gggaaggggctggagtgggtcggccgtattaaaagcaaaactgatggtgggacaacaaactacgctgcacccgtgaaaggcagattcacc
IGHV3-15*07    gggaaggggctggagtgggtcggccgtattaaaagcaaaactgatggtgggacaacagactacgctgcacccgtgaaaggcagattcacc
IGHV3-15*08    gggaaggggctggagtgggttggctgtattaaaagcaaagctaatggtgggacaacagactacgctgcacctgtgaaaggcagattcacc
IP_V3-15*p09   gggaaggggctggagtgggttggccgtattaaaagcaaaactgatggtgggacaacagactacgctgcacccgtgaaaggcagattcacc
IP_V3-15*p10   gggaagggactggagtgggttggccgtattaaaagcaaaactgatggtgggacaacagactacgctgcacccgtgaaaggcagattcacc
V_V3-15*01     gggaaggggctggagtgggttggccgtattaaaagcaaaactgatggtgggacaacagactacgctgcacccgtgaaaggcagattcacc
V_V3-15*02     gggaaggggctggagtgggttggccgtattaaaagcaaaactgatggtgggacaacagactacgctgcacccgtgaaaggcagattcacc

V3-15_01       atctcaagagatgattcaaaaaacacgctgtatctgcaaatgaacagcctgaaaaccgaggacacagccgtgtattactgtaccaca
V3-15_02       atctcaagagatgattcaaaaaacacgctgtatctgcaaatgaacagcctgaaaaccgaggacacagccgtgtattactgtaccaca
V3-15_03       atctcaagagatgattcaaaaaacacgctgtatctgcaaatgaacagcctgaaaaccgaggacacagccgtgtattactgtaccaca
V3-15_04       atctcaagagatgattcaaaaaacacgctgtatctgcaaatgaacagcctgaaaaccgaggacacagccgtgtattactgtaccaca
V3-15_05       atctcaagagatgattcaaaaaacacgctgtatctgcaaatgaacagcctgaaaaccgaggacacagccgtgtattactgtaccaca
V3-15_06       atctcaagagatgattcaaaaaacacgctgtatctgcaaatgaacagcctgaaaaccgaggacacagccgtgtattactgtaccaca
V3-15_07       atctcaagagatgattcaaaaaacacgctgtatctgcaaatgaacagcctgaaaaccgaggacacagccgtgtattactgtaccaca
V3-15_08       atctcaagagatgattcaaaaaacacgctgtatctgcaaatgaacagcctgaaaaccgaggacacagccgtgtattactgtaccaca
V3-15_09       atctcaagagatgatccaaaaaacacgctgtatctgcaaatgaacagcctgaaaaccgaggacacagccgtgtattactgtaccaca
IGHV3-15*01    atctcaagagatgattcaaaaaacacgctgtatctgcaaatgaacagcctgaaaaccgaggacacagccgtgtattactgtaccaca
IGHV3-15*02    atctcaagagatgattcaaaaaacacgctgtatctgcaaatgaacagcctgaaaaccgaggacacagccgtgtattactgtaccaca
IGHV3-15*03    atctcaagagttgattcaaaaaacacgctgtatctgcaaatgaacagcctgaaaaccgaggacacagccgtgtattactgtaccaca
IGHV3-15*04    atctcaagagatgattcaaaaaacacgctgtatctgcaaatgaacagcctgaaaaccgaggacacagccgtgtattactgtaccaca
IGHV3-15*05    atctcaagagatgattcaaaaaacacgctgtatctgcaaatgaacagtctgaaaaccgaggacacagccgtgtattactgtaccaca
IGHV3-15*06    atctcaagagatgattcaaaaaacacgctgtatctgcaaatgaacagcctgaaaaccgaggacacagccgtgtattactgtaccaca
IGHV3-15*07    atctcaagagatgattcaaaaaacacgctgtatctgcaaatgaacagcctgaaaaccgaggacacagccgtgtattactgtaccaca
IGHV3-15*08    atctcaagagatgattcaaaaaacacgctgtatctgcaaatgatcagcctgaaaaccgaggacacggccgtgtattactgtaccaca
IP_V3-15*p09   atctcaagagatgattcaaaaaacacgctgtatctgcaaatgaacagcctgaaaaccgaggacacagccgtgtattactgtaccaca
IP_V3-15*p10   atctcaagagatgattcaaaaaacacgctgtatctgcaaatgaacagcctgaaaaccgaggacacagccgtgtattactgtaccaca
V_V3-15*01     atctcaagagatgattcaaaaaacacgctgtatctgcaaatgaacagcctgaaaaccgaggacacagccgtgtattactgtaccaca
V_V3-15*02     atctcaagagatgattcaaaaaacacgctgtatctgcaaatgaacagcctgaaaaccgaggacacagccgtgtattactgtaccaca
```

IGHV3-16

```
V3-16_01      atggaatttgggctgagctgggttttcttgctggtattttaaaaggtgtccagtgtgaggtgcagctggtggagtctgggggaggcttggt
V3-16_02      atggaatttgggctgagctgggttttcttgctggtattttaaaaggtgtccagtgtgaggtgcagctggtggagtctgggggaggcttggt
V3-16_03      atggaatttgggctgagctgggttttcttgctggtattttaaaaggtgtccagtgtgaggtgcagctggtggagtctgggggaggcttggt
V3-16_04      atggaatttgggctgagctgggttttcttgctggtattttaaaaggtgtccagtgtgaggtgcagctggtggagtctgggggaggcttggt
V3-16_05      atggaatttgggctgagctgggttttcttgctggtattttaaaaggtgtccagtgtgaggtacagctggtggagtctgggggaggcttggt
IGHV3-16*01   atggaatttgggctgagctgggttttcttgctggtattttaaaaggtgtccagtgtgaggtacaactggtggagtctgggggaggcttggt
IGHV3-16*02   atggaatttgggctgagctgggttttcttgctggtattttaaaaggtgtccagtgtgaggtgcagctggtggagtctgggggaggcttggt
V_V3-16*02    --------------------------------------------------------gaggtgcagctggtggagtctgggggaggcttggt

V3-16_01      acagcctggggggtccctgagactctcctgtgcagcctctggattcaccttcagtaacagtgacatgaactgggcccgcaaggctcca
V3-16_02      acagcctggggggtccctgagactctcctgtgcagcctctggattcaccttcagtaacagtgacatgaactgggcctgcaaggctcca
V3-16_03      acagcctggggggtccctgatactctcctgtgcagcctctggattcaccttcagtaacagtgacatgaactgggcccgcaaggctcca
V3-16_04      acagcctggggggtccctgatactctcctgtgcagcctctggattcaccttcagtaacagtgacatgaactgggcccgcgaggctcca
V3-16_05      acagcctggggggtccctgagactctcctgtgcagcctctggattcaccttcagtaacagtgacatgaactgggcccgcaaggctcca
IGHV3-16*01   acagcctggggggtccctgagactctcctgtgcagcctctggattcaccttcagtaacagtgacatgaactgggcccgcaaggctcca
IGHV3-16*02   acagcctggggggtccctgagactctcctgtgcagcctctggattcaccttcagtaacagtgacatgaactgggcccgcaaggctcca
V_V3-16*02    acagcctggggggtccctgagactctcctgtgcagcctctggattcaccttcagtaacagtgacatgaactgggcccgcaaggctcca

V3-16_01      ggaaaggggctggagtgggtatcgggtgttagttggaatggcagtaggacgcactatgtggactccgtgaagcgccgattcatcatctccag
V3-16_02      ggaaaggggctggagtgggtatcgggtgttagttggaatggcagtaggacgcactatgtggactccgtgaagcgccgattcatcatctccag
V3-16_03      ggaaaggggctgaagtgggtatcgggtgttagttggaatggcagtaggacgcactatgtggactccgtgaagcgccgattcatcatctccag
V3-16_04      ggaaaggggctggagtgggtatcgggtgttagttggaatggcagtaggacgcactatgtggactccgtgaagcgccgattcatcatctccag
V3-16_05      ggaaaggggctggagtgggtatcgggtgttagttggaatggcagtaggacgcactatgtggactccgtgaagcgccgattcatcatctccag
IGHV3-16*01   ggaaaggggctggagtgggtatcgggtgttagttggaatggcagtaggacgcactatgtggactccgtgaagcgccgattcatcatctccag
IGHV3-16*02   ggaaaggggctggagtgggtatcgggtgttagttggaatggcagtaggacgcactatgtggactccgtgaagcgccgattcatcatctccag
V_V3-16*02    ggaaaggggctggagtgggtatcgggtgttagttggaatggcagtaggacgcactatgtggactccgtgaagcgccgattcatcatctccag

V3-16_01      agacaattccaggaactccctgtatctgcaaaagaacagacggagagccgaggacatggctgtgtattactgtgtgaga
V3-16_02      agacaattccaggaactccctgtatctgcaaaagaacagacggagagccgaggacatggctgtgtattactgtgtgaga
V3-16_03      agacaattccaggaactccctgtatctgcaaaagaacagacggagagccgaggacatggctgtgtattactgtgtgaga
V3-16_04      agacaattccaggaactccctgtatctgcaaaagaacagacggagagccgaggacatggctgtgtattactgtgtgaga
V3-16_05      agacaattccaggaactccctgtatctgcaaaagaacagacggagagccgaggacatggctgtgtattactgtgtgaga
IGHV3-16*01   agacaattccaggaactccctgtatctgcaaaagaacagacggagagccgaggacatggctgtgtattactgtgtgaga
IGHV3-16*02   agacaattccaggaactccctgtatctgcaaaagaacagacggagagccgaggacatggctgtgtattactgtgtgaga
V_V3-16*02    agacaattccaggaactccctgtatctgcaaaagaacagacggagagccgaggacatggctgtgtattactgtgtgaga
```

IGHV3-20

```
V3-20_01        atggagtttgggctgagctgggttttccttgttgctattttaaaaggtgtccagtgtgaggtgcagctggtggagtctgggggaggtgtgg
V3-20_02        atggagtttgggctgagctgggttttccttgttgctattttaaaaggtgtccagtgtgaggtgcagctggtggagtctgggggaggtgtgg
V3-20_03        atggagtttgggctgagctgggttttccttgttgctattttaaaaggtgtccagtgtgaggtgcagctggtggagtctgggggaggtgtgg
V3-20_04        atggagtttgggctgagctgggttttccttgttgctattttaaaaggtgtccagtgtgaggtgcagctggtggagtctgggggaggtgtgg
V3-20_05        atggagtttgggctgagctgggttttccttgttgctattttaaaaggtgtccagtgtgaggtgcagctggtggagtctgggggaggtgtgg
IGHV3-20*01     atggagtttgggctgagctgggttttccttgttgctattttaaaaggtgtccagtgtgaggtgcagctggtggagtctgggggaggtgtgg
IGHV3-20*02     atggagtttgggctgagctgggttttccttgttgctattttaaaaggtgtccagtgtgaggtgcagctggtggagtctgggggaggtgtgg
IGHV3-20*03     atggagtttgggctgagctgggttttccttgttgctattttaaaaggtgtccagtgtgaggtgcagctggtggagtctgggggaggtgtgg
IGHV3-20*04     atggagtttgggctgagctgggttttccttgttgctattttaaaaggtgtccagtgtgaggtgcagctggtggagtctgggggaggtgtgg
IP_V3-20*p02    ---------------------------------------------------------gaggtgcagctggtggagtctgggggaggtgtgg
IP_V3-20*p03    ---------------------------------------------------------gaggtgcagctggtggagtctgggggaggtgtgg
V_V3-20*01      ---------------------------------------------------------gaggtgcagctggtggagtctgggggaggtgtgg

V3-20_01        tacggcctggggggtccctgagactctcctgtgcagcctctggattcacctttgatgattatggcatgagctgggtccgccaagctcca
V3-20_02        tacggcctggggggtccctgagactctccttggcagcctctggattcacctttgatgattatggcatgagctgggtccgccaagctcca
V3-20_03        tacggcctggggggtccctgagactctcctgtgcagcctctggattcacctttgatgattatggcatgagctgggtccgccaagctcca
V3-20_04        tacggcctggggggtccctgagactctcctgtgcagcctctggattcacctttggtgattatggcatgagctgggtccgccaagctcca
V3-20_05        tacggcctggggggtccctgagactctcctgtgcagcctctggattcacctttggtgattatggcatgagctgggtccgccaagctcca
IGHV3-20*01     tacggcctggggggtccctgagactctcctgtgcagcctctggattcacctttgatgattatggcatgagctgggtccgccaagctcca
IGHV3-20*02     tacggcctggggggtccctgagactctccttggcagcctctggattcacctttgatgattatggcatgagctgggtccgccaagctcca
IGHV3-20*03     tacggcctggggggtccctgagactctccttggcagcctctggattcacctttgatgattatggcatgagctgggtccgccaagctcca
IGHV3-20*04     tacggcctggggggtccctgagactctcctgtgcagcctctggattcacctttgatgattatggcatgagctgggtccgccaagctcca
IP_V3-20*p02    tacggcctggggggtccctgagactctcctgtgcagcctctggattcacctttgatgattatggcatgagctgggtccgccaagctcca
IP_V3-20*p03    tacggcctggggggtccctgagactctcctgtgcagcctctggattcacctttggtgattatggcatgagctgggtccgccaagctcca
V_V3-20*01      tacggcctggggggtccctgagactctcctgtgcagcctctggattcacctttgatgattatggcatgagctgggtccgccaagctcca

V3-20_01        gggaaggggctggagtgggtctctggtattaattggaatggtggtagcacaggttatgcagactctgtgaagggccgattcaccatctcca
V3-20_02        gggaaggggctggagtgggtctctggtattaattggaatggtggtagcacaggttatgcagactctgtgaagggccgattcaccatctcca
V3-20_03        gggaaggggctggagtgggtctctggtattaattggaatggtggtagcacaggttatgcagactctgtgaagggccgattcaccatctcca
V3-20_04        gggaaggggctggagtgggtctctggtattaattggaatggtggtagcacaggttatgcagactctgtgaagggccgattcaccatctcca
V3-20_05        gggaaggggctggagtgggtctctggtattaattggaatggtggtagcacaggttatgcagactctgtgaagggccgattcaccatctcca
IGHV3-20*01     gggaaggggctggagtgggtctctggtattaattggaatggtggtagcacaggttatgcagactctgtgaagggccgattcaccatctcca
IGHV3-20*02     gggaaggggctggagtgggtctctggtattaattggaatggtggtagcacaggttatgcagactctgtgaagggccgattcaccatctcca
IGHV3-20*03     gggaaggggctggagtgggtctctggtattaattggaatggtggtagcacaggttatgcagactctgtgaagggccgattcaccatctcca
IGHV3-20*04     gggaaggggctggagtgggtctctggtattaattggaatggtggtagcacaggttatgcagactctgtgaagggccgattcaccatctcca
IP_V3-20*p02    gggaaggggctggagtgggtctctggtattaattggaatggtggtagcacaggttatgcagactctgtgaagggccgattcaccatctcca
IP_V3-20*p03    gggaaggggctggagtgggtctctggtattaattggaatggtggtagcacaggttatgcagactctgtgaagggccgattcaccatctcca
V_V3-20*01      gggaaggggctggagtgggtctctggtattaattggaatggtggtagcacaggttatgcagactctgtgaagggccgattcaccatctcca
```

```
V3-20_01        gagacaacgccaagaactccctgtatctgcaaatgaacagtctgagagccgaggacacggccttgtatcactgtgcgaga
V3-20_02        gagacaacgccaagaactccctgtatctgcaaatgaacagtctgagagccgaggacacggccttgtatcactgtgcgaga
V3-20_03        gagacaacgccaagaactccctgtatctgcaaatgaacagtctgagagccgaggacacggccttgtattactgtgcgaga
V3-20_04        gagacaacgccaagaactccctgtatctgcaaatgaacagtctgagagccgaggacacggccttgtattactgtgcgaga
V3-20_05        gagacaacgccaagaactccctgtatctgcaaatgaacagtctgagagccgaggacacggccttgtatcactgtgcgaga
IGHV3-20*01     gagacaacgccaagaactccctgtatctgcaaatgaacagtctgagagccgaggacacggccttgtatcactgtgcgaga
IGHV3-20*02     gagacaacgccaagaactccctgtatctgcaaatgaacagtctgagagccgaggacacggccttgtatcactgtgcgaga
IGHV3-20*03     gagacaacgccaagaactccctgtatctgcaaatgaacagtctgagagccgaggacacggccttgtattactgtgcgaga
IGHV3-20*04     gagacaacgccaagaactccctgtatctgcaaatgaacagtctgagagccgaggacacggccttgtattactgtgcgaga
IP_V3-20*p02    gagacaacgccaagaactccctgtatctgcaaatgaacagtctgagagccgaggacacggccttgtattactgtgcgaga
IP_V3-20*p03    gagacaacgccaagaactccctgtatctgcaaatgaacagtctgagagccgaggacacggccttgtattactgtgcgaga
V_V3-20*01      gagacaacgccaagaactccctgtatctgcaaatgaacagtctgagagccgaggacacggccttgtatcactgtgcgaga
```

IGHV3-21

```
V3-21_01      atggaactggggctccgctgggttttccttgttgctattttagaaggtgtccagtgtgaggtgcagctggtggagtctgggggaggcctg
V3-21_02      atggaactggggctccgctgggttttccttgttgctattttagaaggtgtccagtgtgaggtgcagctggtggagtctgggggaggcctg
V3-21_03      atggaactggggctccgctgggttttccttgttgctattttagaaggtgtccagtgtgaggtgcagctggtggagtctgggggaggcctg
V3-21_04      atggaactggggctccgctgggttttccttgttgctattttagaaggtgtccagtgtgaggtgcagctggtggagtctgggggaggcttg
IGHV3-21*01   atggaactggggctccgctgggttttccttgttgctattttagaaggtgtccagtgtgaggtgcagctggtggagtctgggggaggcctg
IGHV3-21*02   atggaactggggctccgctgggttttccttgttgctattttagaaggtgtccagtgtgaggtgcaactggtggagtctgggggaggcctg
IGHV3-21*03   ---------------------------------------------------------gaggtgcagctggtggagtctgggggaggcctg
IGHV3-21*04   ---------------------------------------------------------gaggtgcagctggtggagtctgggggaggcctg
IP_V3-21*p05  ---------------------------------------------------------gaggtgcagctggtggagtctgggggaggcctg
IP_V3-21*p06  ---------------------------------------------------------gaggtgcagctggtggagtctgggggaggcctg
V_V3-21*01    ---------------------------------------------------------gaggtgcagctggtggagtctgggggaggcctg
V_V3-21*02    ---------------------------------------------------------gaggtgcaactggtggagtctgggggaggcctg
```

```
V3-21_01      gtcaagcctggggggtccctgagactctcctgtgcagcctctggattcaccttcagtagctatagcatgaactgggtccgccaggctcca
V3-21_02      gtcaagcctggggggtccctgagactctcctgtgcagcctctggattcaccttcagtagctatagcatgaactgggtccgccaggctcca
V3-21_03      gtcaagcctggggggtccctgagactctcctgtgcagcctctggattcaccttcagtagctatagcatgaactgggtccgccaggctcca
V3-21_04      gtcaagcctggggggtccctgagactctcctgtgcagcctctggattcaccttcagtagctatagcatgaactgggtccgccaggctcca
IGHV3-21*01   gtcaagcctggggggtccctgagactctcctgtgcagcctctggattcaccttcagtagctatagcatgaactgggtccgccaggctcca
IGHV3-21*02   gtcaagcctggggggtccctgagactctcctgtgcagcctctggattcaccttcagtagctatagcatgaactgggtccgccaggctcca
IGHV3-21*03   gtcaagcctggggggtccctgagactctcctgtgcagcctctggattcaccttcagtagctatagcatgaactgggtccgccaggctcca
IGHV3-21*04   gtcaagcctggggggtccctgagactctcctgtgcagcctctggattcaccttcagtagctatagcatgaactgggtccgccaggctcca
IP_V3-21*p05  gtcaagcctggggggtccctgagactctcctgtgcagcctctggattcaccttcagtagctatagcatgaactgggtccgccaggctcca
IP_V3-21*p06  gtcaagcctggggggtccctgagactctcctgtgcagcctctggattcaccttcagtagctatagcatgaactgggtccgccaggctcca
V_V3-21*01    gtcaagcctggggggtccctgagactctcctgtgcagcctctggattcaccttcagtagctatagcatgaactgggtccgccaggctcca
V_V3-21*02    gtcaagcctggggggtccctgagactctcctgtgcagcctctggattcaccttcagtagctatagcatgaactgggtccgccaggctcca
```

```
V3-21_01      gggaaggggctggagtgggtctcatccattagtagtagtagtagttacatatactacgcagactcagtgaagggccgattcaccatctcca
V3-21_02      gggaaggggctggagtgggtctcatccattagtagtagtagtagttacatatactacgcagactcagtgaagggccgattcaccatctcca
V3-21_03      gggaaggggctggagtgggtctcatccattagtagtagtagtagttacatatactacgcagactcagtgaagggccgattcaccatctcca
V3-21_04      gggaaggggctggagtgggtctcatccattagtagtagtagtagttacatatactacgcagactcagtgaagggccgattcaccatctcca
IGHV3-21*01   gggaaggggctggagtgggtctcatccattagtagtagtagtagttacatatactacgcagactcagtgaagggccgattcaccatctcca
IGHV3-21*02   gggaaggggctggagtgggtctcatccattagtagtagtagtagttacatatactacgcagactcagtgaagggccgattcaccatctcca
IGHV3-21*03   gggaaggggctggagtgggtctcatccattagtagtagtagtagttacatatactacgcagactcagtgaagggccgattcaccatctcca
IGHV3-21*04   gggaaggggctggagtgggtctcatccattagtagtagtagtagttacatatactacgcagactcagtgaagggccgattcaccatctcca
IP_V3-21*p05  gggaaggggctggagtgggtctcatccattagtagtagtagtagttacatatactacgcagactcagtgaagggccgattcaccatctcca
IP_V3-21*p06  gggaaggggctggagtgggtctcatccattagtagtagtagtagttacatatactacgcagactcagtgaagggccgattcaccatctcca
V_V3-21*01    gggaaggggctggagtgggtctcatccattagtagtagtagtagttacatatactacgcagactcagtgaagggccgattcaccatctcca
V_V3-21*02    gggaaggggctggagtgggtctcatccattagtagtagtagtagttacatatactacgcagactcagtgaagggccgattcaccatctcca
```

```
V3-21_01        gagacaacgccaagaactcactgtatctgcaaatgaacagcctgagagccgaggacacggctgtgtattactgtgcgaga
V3-21_02        gagacaacgccaagaactcactgtatctgcaaatgaacagcctgagagccgaggacacagctgtgtattactgtgcgaga
V3-21_03        gagacaacgccaagaactcactgtatctgcaaatgaacagcctgagagccgaggacacggctgtgtattactgtgcaaga
V3-21_04        gagacaacgccaagaactcactgtatctgcaaatgaacagcctgagagccgaggacacggctgtgtattactgtgcgaga
IGHV3-21*01     gagacaacgccaagaactcactgtatctgcaaatgaacagcctgagagccgaggacacggctgtgtattactgtgcgaga
IGHV3-21*02     gagacaacgccaagaactcactgtatctgcaaatgaacagcctgagagccgaggacacggctgtgtattactgtgcgaga
IGHV3-21*03     gagacaacgccaagaactcactgtatctgcaaatgaacagcctgagagccgaggacacagctgtgtattactgtgcgaga
IGHV3-21*04     gagacaacgccaagaactcactgtatctgcaaatgaacagcctgagagccgaggacacggccgtgtattactgtgcgaga
IP_V3-21*p05    gagacaacgccaagaactcactgtatctgcaaatgaacagcctgagagccgaggacacggctgtgtattactgtgcaaga
IP_V3-21*p06    gagacaacgccaagaactcactgtatctgcaaatgaacagcctgagagccgaggacacggctgtgtattactgtactaga
V_V3-21*01      gagacaacgccaagaactcactgtatctgcaaatgaacagcctgagagccgaggacacggctgtgtattactgtgcgaga
V_V3-21*02      gagacaacgccaagaactcactgtatctgcaaatgaacagcctgagagccgaggacacggctgtgtattactgtgcgaga
```

IGHV3-23

```
V3-23_01       atggagtttgggctgagctggctttttcttgtggctatttaaaaggtgtccagtgtgaggtgcagctgttggagtctgggggaggcttgg
V3-23_02       atggagtttgggctgagctggctttttcttgtggctatttaaaaggtgtccagtgtgaggtgcagctggtggagtctgggggaggcttgg
V3-23_03       atggagtttgggctgagctggctttttcttgtggctatttaaaaggtgtccagtgtgaggtgcagctgttggagtctgggggaggcttgg
V3-23_04       atggagtttgggctgagctggctttttcttgtggctatttaaaaggtgtccagtgtgaggtgcagctgttggagtctgggggaggcttgg
V3-23_05       atggagtttgggctgagctggctttttcttgtggctatttaaaaggtgtccagtgtgaggtgcagctgttggagtctgggggaggcttgg
V3-23_06       atggagtttgggctgagctggctttttcttgtggctatttaaaaggtgtccagtgtgaggtgcagctggtggagtctgggggaggcttgg
V3-23_07       atggagtttgggctgagctggctttttcttgtggctatttaaaaggtgtccagtgtgaggtgcagctgttggagtctgggggaggcttgg
V3-23_08       atggagtttgggctgagctggctttttcttgtggctatttaaaaggtgtccagtgtgaggtgcagctggtggagtctgggggaggcttgg
V3-23_09       atggagtttgggctgagctggctttttcttgtggctatttaaaaggtgtccagtgtgaggtgcagctgttggagtctgggggaggcttgg
V3-23_10       atggagtttgggctgagctggctttttcttgtggctatttaaaaggtgtccagtgtgaggtgcagctgttggagtctgggggaggcttgg
V3-23_11       atggagtttgggctgagctggctttttcttgtggctatttaaaaggtgtccagtgtgaggtgcagctggtggagtctgggggaggcttgg
IGHV3-23*01    atggagtttgggctgagctggctttttcttgtggctaaaataaaaggtgtccagtgtgaggtgcagctgttggagtctgggggaggcttgg
IGHV3-23*02    atggagtttgggctgagctggctttttcttgtggctatttaaaaggtgtccagtgtgaggtgcagctgttggagtctgggggaggcttgg
IGHV3-23*04    atggagtttgggctgagctggctttttcttgtggctatttaaaaggtgtccagtgtgaggtgcagctggtggagtctgggggaggcttgg
IGHV3-23*03    ---------------------------------------------------------gaggtgcagctgttggagtctgggggaggcttgg
IGHV3-23*05    ---------------------------------------------------------gaggtgcagctgttggagtctgggggaggcttgg
IGHV3-23D*01   atggagtttgggctgagctggctttttcttgtggctatttaaaaggtgtccagtgtgaggtgcagctgttggagtctgggggaggcttgg
IP_V3-23*p06   ---------------------------------------------------------gaggtgcagctggtggagtctgggggaggcttgg
IP_V3-23*p07   ---------------------------------------------------------gaggtgcagctggtggagtctgggggaggcttgg
V_V3-23*01     ---------------------------------------------------------gaggtgcagctgttggagtctgggggaggcttgg
V_V3-23*02     ---------------------------------------------------------gaggtgcagctgttggagtctgggggaggcttgg
V_V3-23*03     ---------------------------------------------------------gaggtgcagctgttggagtctgggggaggcttgg

V3-23_01       tacagcctgggggtccctgagactctcctgtgcagcctctggattcacctttagcagctatgccatgagctgggtccgccaggctcca
V3-23_02       tacagcctgggggtccctgagactctcctgtgcagcctctggattcacctttagcagctatgccatgagctgggtccgccaggctcca
V3-23_03       tacagcctgggggtccctgagactctcctgtgcagcctctggattcacctttagcagctatgccatgagctgggtccgccaggctcca
V3-23_04       tacagcctgggggtccctgagactctcctgtgcagcctctggattcacctttagcagctatgccatgagctgggtccgccaggctcca
V3-23_05       tacagcctgggggtccctgagactctcctgtgcagcctctggattcacctttagcagctatgccatgagctgggtccgccaggctcca
V3-23_06       tacagcctgggggtccctgagactctcctgtgcagcctctggattcacctttagcagctatgccatgagctgggtccgccaggctcca
V3-23_07       tacagcctggggggaccctgagactctcctgtgcagcctctggattcacctttagcagctatgccatgagctgggtccgccaggctcca
V3-23_08       tacagcctggggggaccctgagactctcctgtgcagcctctggattcacctttagcagctatggcatgagctgggtccgccaggctcca
V3-23_09       tacagcctgggggttccctgagactctcctgtgcagcctctggattcacctttagcagctatgccatgagctgggtccgccaggctcca
V3-23_10       tacagcctgggggttccctgagactctcctgtgcagcctctggattcacctttagcagctatgccatgagctgggtccgccaggctcca
V3-23_11       tacagcctgggggtccctgagactctcctgtgcagcctctggattcacctttagcagctatggcatgagctgggtccgccaggctcca
IGHV3-23*01    tacagcctgggggtccctgagactctcctgtgcagcctctggattcacctttagcagctatgccatgagctgggtccgccaggctcca
IGHV3-23*02    tacagcctgggggtccctgagactctcctgtgcagcctctggattcacctttagcagctatgccatgagctgggtccgccaggctcca
IGHV3-23*04    tacagcctgggggtccctgagactctcctgtgcagcctctggattcacctttagcagctatgccatgagctgggtccgccaggctcca
IGHV3-23*03    tacagcctgggggtccctgagactctcctgtgcagcctctggattcacctttagcagctatgccatgagctgggtccgccaggctcca
IGHV3-23*05    tacagcctgggggtccctgagactctcctgtgcagcctctggattcacctttagcagctatgccatgagctgggtccgccaggctcca
IGHV3-23D*01   tacagcctgggggtccctgagactctcctgtgcagcctctggattcacctttagcagctatgccatgagctgggtccgccaggctcca
IP_V3-23*p06   tacagcctgggggtccctgagactctcctgtgcagcctctggattcacctttagcagctatgccatgagctgggtccgccaggctcca
IP_V3-23*p07   tacagcctggggggaccctgagactctcctgtgcagcctctggattcacctttagcagctatggcatgagctgggtccgccaggctcca
V_V3-23*01     tacagcctgggggtccctgagactctcctgtgcagcctctggattcacctttagcagctatgccatgagctgggtccgccaggctcca
V_V3-23*02     tacagcctgggggtccctgagactctcctgtgcagcctctggattcacctttagcagctatgccatgagctgggtccgccaggctcca
V_V3-23*03     tacagcctgggggtccctgagactctcctgtgcagcctctggattcacctttagcagctatgccatgagctgggtccgccaggctcca
```

```
V3-23_01      gggaaggggctggagtgggtctcagttatttatagcggtggtagtagcacatactatgcagactccgtgaagggccggttcaccatctc
V3-23_02      gggaaggggctggagtgggtctcagctattagtggtagtggtggtagcacatactacgcagactccgtgaagggccggttcaccatctc
V3-23_03      gggaaggggctggagtgggtctcagctattagtggtagtggtggtagcacatactacgcagactccgtgaagggccggttcaccatctc
V3-23_04      gggaaggggctggagtgggtctcagctattagtggtagtggtggtagcacatactatgcagactccgtgaagggccggttcaccatctc
V3-23_05      gggaaggggctggagtgggtctcagttatttatagcggtggtagtagcacatactacgcagactccgtgaagggccggttcaccatctc
V3-23_06      gggaaggggctggagtgggtctcagctattagtggtagtggtggtagcacatactatgcagactccgtgaagggccggttcaccatctc
V3-23_07      gggaaggggctggagtgggtctcagctattagtggtagtggtggtagcacatactacgcagactccgtgaagggccggttcaccatctc
V3-23_08      gggaaggggctggagtgggtctcagctattagtggtagtggtggtagcacatactacgcagactccgtgaagggccggttcaccatctc
V3-23_09      gggaaggggctggagtgggtctcagctattagtggtagtggtggtagcacatactatgcagactccgtgaagggccggttcaccatctc
V3-23_10      gggaaggggctggagtgggtctcagttatttatagcggtggtagtagcacatactatgcagactccgtgaagggccggttcaccatctc
V3-23_11      gggaaggggctggagtgggtctcagctattagtggtagtggtggtagcacatactacgcagactccgtgaagggccggttcaccatctc
IGHV3-23*01   gggaaggggctggagtgggtctcagctattagtggtagtggtggtagcacatactacgcagactccgtgaagggccggttcaccatctc
IGHV3-23*02   gggaaggggctggagtgggtctcagctattagtggtagtggtggtagcacatactacggagactccgtgaagggccggttcaccatctc
IGHV3-23*04   gggaaggggctggagtgggtctcagctattagtggtagtggtggtagcacatactacgcagactccgtgaagggccggttcaccatctc
IGHV3-23*03   gggaaggggctggagtgggtctcagttatttatagcggtggtagtagcacatactatgcagactccgtgaagggccggttcaccatctc
IGHV3-23*05   gggaaggggctggagtgggtctcagctatttatagcagtggtagtagcacatactatgcagactccgtgaagggccggttcaccatctc
IGHV3-23D*01  gggaaggggctggagtgggtctcagctattagtggtagtggtggtagcacatactacgcagactccgtgaagggccggttcaccatctc
IP_V3-23*p06  gggaaggggctggagtgggtctcagctattagtggtagtggtggtagcacatactacgcagactccgtgaagggccggttcaccatctc
IP_V3-23*p07  gggaaggggctggagtgggtctcagctattagtggtagtggtggtagcacatactacgcagactccgtgaagggccggttcaccatctc
V_V3-23*01    gggaaggggctggagtgggtctcagctattagtggtagtggtggtagcacatactacgcagactccgtgaagggccggttcaccatctc
V_V3-23*02    gggaaggggctggagtgggtctcagctattagtggtagtggtggtagcacatactacggagactccgtgaagggccggttcaccatctc
V_V3-23*03    gggaaggggctggagtgggtctcagttatttatagcggtggtagtagcacatactatgcagactccgtgaagggccggttcaccatctc

V3-23_01      cagagataattccaagaacacgctgtatctgcaaatgaacagcctgagagccgaggacacggccgtatattactgtgcgaaa
V3-23_02      cagagacaattccaagaacacgctgtatctgcaaatgaacagcctgagagccgaggacacggccgtatattactgtgcgaaa
V3-23_03      cagagacaattccaagaacacgctgtatctgcaaatgaacagcctgagagccgaggacacggccgtatattactgtgcgaaa
V3-23_04      cagagataattccaagaacacgctgtatctgcaaatgaacagcctgagagccgaggacacggccgtatattactgtgcgaaa
V3-23_05      cagagacaattccaagaacacgctgtatctgcaaatgaacagcctgagagccgaggacacggccgtatattactgtgcgaaa
V3-23_06      cagagataattccaagaacacgctgtatctgcaaatgaacagcctgagagccgaggacacggccgtatattactgtgcgaaa
V3-23_07      cagagacaattccaagaacacgctgtatctgcaaatgaacagcctgagagccgaggacacggccgtatattactgtgcgaaa
V3-23_08      cagagacaattccaagaacacgctgtatctgcaaatgaacagcctgagagccgaggacacggccgtatattactgtgcgaaa
V3-23_09      cagagataattccaagaacacgctgtatctgcaaatgaacagcctgagagccgaggacacggccgtatattactgtgcgaaa
V3-23_10      cagagataattccaagaacacgctgtatctgcaaatgaacagcctgagagccgaggacacggccgtatattactgtgcgaaa
V3-23_11      cagagacaattccaagaacacgctgtatctgcaaatgaacagcctgagagccgaggacacggccgtatattactgtgcgaaa
IGHV3-23*01   cagagacaattccaagaacacgctgtatctgcaaatgaacagcctgagagccgaggacacggccgtatattactgtgcgaaa
IGHV3-23*02   aagagacaattccaagaacacgctgtatctgcaaatgaacagcctgagagccgaggacacggccgtatattactgtgcgaaa
IGHV3-23*04   cagagacaattccaagaacacgctgtatctgcaaatgaacagcctgagagccgaggacacggccgtatattactgtgcgaaa
IGHV3-23*03   cagagataattccaagaacacgctgtatctgcaaatgaacagcctgagagccgaggacacggccgtatattactgtgcgaaa
IGHV3-23*05   cagagacaattccaagaacacgctgtatctgcaaatgaacagcctgagagccgaggacacggccgtatattactgtgcgaaa
IGHV3-23D*01  cagagacaattccaagaacacgctgtatctgcaaatgaacagcctgagagccgaggacacggccgtatattactgtgcgaaa
IP_V3-23*p06  cagagacaattccaagaacacgctgtatctgcaaatgaacagcctgagagccgaggacacggccgtatattactgtgtcaaa
IP_V3-23*p07  cagagacaattccaagaacacgctgtatctgcaaatgaacagcctgagagccgaggacacggccgtatattactgtgcgaaa
V_V3-23*01    cagagacaattccaagaacacgctgtatctgcaaatgaacagcctgagagccgaggacacggccgtatattactgtgcgaaa
V_V3-23*02    aagagacaattccaagaacacgctgtatctgcaaatgaacagcctgagagccgaggacacggccgtatattactgtgcgaaa
V_V3-23*03    cagagataattccaagaacacgctgtatctgcaaatgaacagcctgagagccgaggacacggccgtatattactgtgcgaaa
```

IGHV3-30

```
V3-30_01        atggagtttgggctgagctggttttcctcgttgctcttttaagaggtgtccagtgtcaggtgcagctggtggagtctgggggaggcgt
V3-30_02        atggagtttgggctgagctggttttcctcgttgctcttttaagaggtgtccagtgtcaggtgcagctggtggagtctgggggaggcgt
V3-30_03        atggagtttgggctgagctggttttcctcgttgctcttttaagaggtgtccagtgtcaggtgcagctggtggagtctgggggaggcgt
V3-30_04        atggagtttgggctgagctggttttcctcgttgctcttttaagaggtgtccagtgtcaggtgcagctggtggagtctgggggaggcgt
V3-30_05        atggagtttgggctgagctggttttcctcgttgctcttttaagaggtgtccagtgtcaggtgcagctggtggagtctgggggaggcgt
V3-30_06        atggagtttgggctgagctggttttcctcgttgctcttttaagaggtgtccagtgtcaggtgcagctggtggagtctgggggaggcgt
V3-30_07        atggagtttgggctgagctggttttcctcgttgctcttttaagaggtgtccagtgtcaggtgcagctggtggagtctgggggaggcgt
V3-30_08        atggagtttgggctgagctggttttcctcgttgctcttttaagaggtgtccagtgtcaggtgcagctggtggagtctgggggaggcgt
V3-30_09        atggagtttgggctgagctggttttcctcgttgctcttttaagaggtgtccagtgtcaggtgcagctggtggagtctgggggaggcgt
V3-30_10        atggagtttgggctgagctggttttcctcgttgctcttttaagaggtgtccagtgtcaggtgcagctggtggagtctgggggaggcgt
V3-30_11        atggagtttgggctgagctggttttcctcgttgctcttttaagaggtgtccagtgtcaggtgcagctggtggagtctgggggaggcgt
V3-30_12        atggagtttgggctgagctggttttcctcgttgctcttttaagaggtgtccagtgtcaggtgcagctggtggagtctgggggaggcgt
V3-30_13        atggagtttgggctgaggtggttttcctcgttgctcttttaagaggtgtccagtgtcaggtgcagctggtggagtctgggggaggcgt
V3-30_14        atggagtttgggctgagctggttttcctcgttgctcttttaagaggtgtccagtgtcaggtgcagctggtggagtctgggggaggcgt
V3-30_15        atggagtttgggctgagctggttttcctcgttgctcttttaagaggtgtccagtgtcaggtgcagctggtggagtctgggggaggcgt
V3-30_16        atggagtttgggctgagctggttttcctcgttgctcttttaagaggtgtccagtgtcaggtgcagctggtggagtctgggggaggcgt
V3-30_17        atggagtttgggctgaggtggttttcctcgttgctcttttaagaggtgtccagtgtcaggtgcagctggtggagtctgggggaggcgt
V3-30_18        atggagtttgggctgagctggttttcctcgttgctcttttaagaggtgtccagtgtcaggtgcagctggtggagtctgggggaggcgt
V3-30_19        atggagtttgggctgagctggttttcctcgttgctcttttaagaggtgtccagtgtcaggtgcagctggtggagtctgggggaggcgt
V3-30_20        atggagtttgggctgaggtggttttcctcgttgctcttttaagaggtgtccagtgtcaggtgcagctggtggagtctgggggaggcgt
V3-30_21        atggagtttgggctgagctggttttcctcgttgctcttttaagaggtgtccagtgtcaggtgcagctggtggagtctgggggaggcgt
V3-30_22        atggagtttgggctgagctggttttcctcgttgctcttttaagaggtgtccagtgtcaggtgcagctggtggagtctgggggaggcgt
V3-30_23        atggagtttgggctgaggtggttttcctcgttgctcttttaagaggtgtccagtgtcaggtgcagctggtggagtctgggggaggcgt
V3-30_25        atggagtttgggctgagctggttttcctcgttgctcttttaagaggtgtccagtgtcaggtgcagctggtggagtctgggggaggcgt
IGHV3-30*01     atggagtttgggctgagctggttttcctcgttgctcttttaagaggtgtccagtgtcaggtgcagctggtggagtctgggggaggcgt
IGHV3-30*03     atggagtttgggctgagctggttttcctcgttgctcttttaagaggtgtccagtgtcaggtgcagctggtggagtctgggggaggcgt
IGHV3-30*18     atggagtttgggctgagctggttttcctcgttgctcttttaagaggtgtccagtgtcaggtgcagctggtggagtctgggggaggcgt
IGHV3-30*02     --------------------------------------------------------caggtgcagctggtggagtctgggggaggcgt
IGHV3-30*04     --------------------------------------------------------caggtgcagctggtggagtctgggggaggcgt
IGHV3-30*05     --------------------------------------------------------caggtgcagctggtggagtctgggggaggcgt
IGHV3-30*06     --------------------------------------------------------caggtgcagctggtggagtctgggggaggcgt
IGHV3-30*07     --------------------------------------------------------caggtgcagctggtggagtctgggggaggcgt
IGHV3-30*08     --------------------------------------------------------caggtgcagctggtggactctgggggaggcgt
IGHV3-30*09     --------------------------------------------------------caggtgcagctggtggagtctgggggaggcgt
IGHV3-30*10     --------------------------------------------------------caggtgcagctggtggagtctgggggaggcgt
IGHV3-30*11     --------------------------------------------------------caggtgcagctggtggagtctgggggaggcgt
IGHV3-30*12     --------------------------------------------------------caggtgcagctggtggagtctggggggggcgt
IGHV3-30*13     --------------------------------------------------------caggtgcagctggtggagtctgggggaggcgt
IGHV3-30*14     --------------------------------------------------------caggtgcagctggtggagtctgggggaggcgt
IGHV3-30*15     --------------------------------------------------------caggtgcagctggtggagtctgggggaggcgt
IGHV3-30*16     --------------------------------------------------------caggtgcagctggtggagtctgggggaggcgt
IGHV3-30*17     --------------------------------------------------------caggtgcagctggtggagtctgggggaggcgt
IGHV3-30*19     --------------------------------------------------------caggtgcagctggtggagtctgggggaggcgt
IP_V3-30*p20    --------------------------------------------------------caggtgcagctggtggagtctgggggaggcgt
```

```
IP_V3-30*p20      ---------------------------------------------------------caggtgcagctggtggagtctgggggaggcgt
IP_V3-30*p21      -----------------------------------------------------------------------------------------
IP_V3-30*p22      ---------------------------------------------------------caggtgcagctggtggagtctgggggaggcgt
IP_V3-30*p23      ---------------------------------------------------------caggtgcagctggtggagtctgggggaggcgt
IP_V3-30*p24      ---------------------------------------------------------caggtgcagctggtggagtctgggggaggcgt
IP_V3-30*p25      ---------------------------------------------------------caggtgcagctggtggagtctgggggaggcgt
IP_V3-30*p27      ---------------------------------------------------------caggtgcagctggtggagtctgggggaggcgt
V_V3-30*03        ---------------------------------------------------------caggtgcagctggtggagtctgggggaggcgt
V_V3-30*01        ---------------------------------------------------------caggtgcagctggtggagtctgggggaggcgt
V_V3-30*04        ---------------------------------------------------------caggtgcagctggtggagtctgggggaggcgt

IGHV3-30-5*01     atggagtttgggctgagctgggttttcctcgttgctcttttaagaggtgtccagtgtcaggtgcagctggtggagtctgggggaggcgt
IGHV3-30-5*02     atggagtttgggctgagctgggttttcctcgttgctcttttaagaggtgtccagtgtcaggtgcagctggtggagtctgggggaggcgt
IGHV3-30-22*01    atggggtgtgaattaagctgaattttcttgttggtatttaaaaggtgttcagtgtgaggtggagctgatagagtccatagaggacct
IGHV3-30-33*01    atggagcttgggctgagctgggttttcactgttgctgttttaaaaggtgtctagtgagaggtacagctcgtggagtccggagaggaccc
IGHV3-30-42*01    atggggtgtgaattaagctgaattttcttgttggtatttaaaaggtgttcagtgtgaggtggagctgatagagcccacagaggacct
IGHV3-30-52*01    atggagcttgggctgagctgggttttcactgttgctgttttaaaaggtgtctagtgagaggtacagctcgtggagtccggagaggaccc
```

```
V3-30_01       ggtccagcctggggaggtccctgagactctcctgtgcagcctctggattcaccttcagtagctatgctatgcactgggtccgccaggctcca
V3-30_02       ggtccagcctggggaggtccctgagactctcctgtgcagcctctggattcaccttcagtagctatggcatgcactgggtccgccaggctcca
V3-30_03       ggtccagcctggggaggtccctgagactctcctgtgcagcctctggattcaccttcagtagctatgctatgcactgggtccgccaggctcca
V3-30_04       ggtccagcctggggaggtccctgagactctcctgtgcagcctctggattcaccttcagtagctatggcatgcactgggtccgccaggctcca
V3-30_05       ggtccagcctggggaggtccctgagactctcctgtgcagcctctggattcaccttcagtagctatggcatgcactgggtccgccaggctcca
V3-30_06       ggtccagcctggggaggtccctgagactctcctgtgcagcctctggattcaccttcagtagctatgctatgcactgggtccgccaggctcca
V3-30_07       ggtccagcctggggaggtccctgagactctcctgtgcagcctctggattcaccttcagtagctatgctatgcactgggtccgccaggctcca
V3-30_08       ggtccagcctggggaggtccctgagactctcctgtgcagcctctggattcaccttcagtagctatggcatgcactgggtccgccaggctcca
V3-30_09       ggtccagcctggggaggtccctgagactctcctgtgcagcctctggattcaccttcagtagctatgctatgcactgggtccgccaggctcca
V3-30_10       ggtccagcctggggaggtccctgagactctcctgtgcagcctctggattcaccttcagtagctatggcatgcactgggtccgccaggctcca
V3-30_11       ggtccagcctggggaggtccctgagactctcctgtgcagcctctggattcaccttcagtagctatgctatgcactgggtccgccaggctcca
V3-30_12       ggtccagcctggggaggtccctgagactctcctgtgcagcctctggattcaccttcagtagctatgctatgcactgggtccgccaggctcca
V3-30_13       ggtccagcctggggaggtccctgagactctcctgtgcagcctctggattcaccttcagtagctatgctatgcactgggtccgccaggctcca
V3-30_14       ggtccagcctggggaggtccctgagactctcctgtgcagcctctggattcaccttcagtagctatggcatgcactgggtccgccaggctcca
V3-30_15       ggtccagcctggggaggtccctgagactctcctgtgcagcgtctggattcaccttcagtagctatggcatgcactgggtccgccaggctcca
V3-30_16       ggtccagcctggggaggtccctgagactctcctgtgcagcctctggattcaccttcagtagctatgctatgcactgggtccgccaggctcca
V3-30_17       ggtccagcctggggaggtccctgagactctcctgtgcagcctctggattcaccttcagtagctatgctatgcactgggtccgccaggctcca
V3-30_18       ggtccagcctggggaggtccctgagactctcctgtgcagcctctggattcaccttcagtagctatgctatgcactgggtccgccaggctcca
V3-30_19       ggtccagcctggggaggtccctgagactctcctgtgcagcctctggattcaccttcagtagctatggcatgcactgggtccgccaggctcca
V3-30_20       ggtccagcctggggaggtccctgagactctcctgtgcagcctctggattcaccttcagtagctatgctatgcactgggtccgccaggctcca
V3-30_21       ggtccagcctggggaggtccctgagactctcctgtgcagcgtctggattcaccttcagtagctatggcatgcactgggtccgccaggctcca
V3-30_22       ggtccagcctggggaggtccctgagactctcctgtgcagcctctggattcaccttcagtagctatgctatgcactgggtccgccaggctcca
V3-30_23       ggtccagcctggggaggtccctgagactctcctgtgcagcctctggattcaccttcagtagctatgctatgcactgggtccgccaggctcca
V3-30_25       ggtccagcctggggaggtccctgagactctcctgtgcagcctctggattcaccttcagtagctatggcatgcactgggtccgccaggctcca
IGHV3-30*01    ggtccagcctggggaggtccctgagactctcctgtgcagcctctggattcaccttcagtagctatgctatgcactgggtccgccaggctcca
IGHV3-30*03    ggtccagcctggggaggtccctgagactctcctgtgcagcctctggattcaccttcagtagctatggcatgcactgggtccgccaggctcca
IGHV3-30*18    ggtccagcctggggaggtccctgagactctcctgtgcagcctctggattcaccttcagtagctatggcatgcactgggtccgccaggctcca
IGHV3-30*02    ggtccagcctgggggggtccctgagactctcctgtgcagcgtctggattcaccttcagtagctatggcatgcactgggtccgccaggctcca
IGHV3-30*04    ggtccagcctggggaggtccctgagactctcctgtgcagcctctggattcaccttcagtagctatgctatgcactgggtccgccaggctcca
IGHV3-30*05    ggtccagcctggggaggtccctgagactctcctgtgcagcctctggattcaccttcagtagctatggcatgcactgggtccgccaggctcca
IGHV3-30*06    ggtccagcctggggaggtccctgagactctcctgtgcagcgtctggattcaccttcagtagctatggcatgcactgggtccgccaggctcca
IGHV3-30*07    ggtccagcctggggaggtccctgagactctcctgtgcagcctctggattcaccttcagtagctatgctatgcactgggtccgccaggctcca
IGHV3-30*08    ggtccagcctggggaggtccctgagactctcctgtgcagcctctgcattcaccttcagtagctatgctatgcactgggtccgccaggctcca
IGHV3-30*09    ggtccagcctggggaggtccctgagactctcctgtgcagcctctggattcaccttcagtagctatgctatgcactgggtccgccaggctcca
IGHV3-30*10    ggtccagcctggggaggtccctgagactctcctgtgcagcctctggattcaccttcagtagctatgctatgcactgggtccgccaggctcca
IGHV3-30*11    ggtccagcctggggaggtccctgagactctcctgtgcagcgtctggattcaccttcagtagctatgctatgcactgggtccgccaggctcca
IGHV3-30*12    ggtccagcctggggaggtccctgagactctcctgtgcagcgtctggattcaccttcagtagctatggcatgcactgggtccgccaggctcca
IGHV3-30*13    ggtccagcctggggaggtccctgagactctcctgtgcagcctctggattcaccttcagtagctatggcatgcactgggtccgccaggctcca
IGHV3-30*14    ggtccagcctggggaggtccctgagactctcctgtgcagcctctggattcaccttcagtagctatgctatgcactgggtccgccaggctcca
IGHV3-30*15    ggtccagcctggggaggtccctgagactctcctgtgcagcctctggattcaccttcagtagctatgctatgcactgggtccgccaggctcca
IGHV3-30*16    ggtccagcctggggaggtccctgagactctcctgtgcagcctctggattcaccttcagtagctatgctatgcactgggtccgccaggcccca
IGHV3-30*17    ggtccagcctggggaggtccctgagactctcctgtgcagcctctggattcaccttcagtagctatgctatgcactgggtccgccaggctccg
IGHV3-30*19    ggtccagcctggggaggtccctgagactctcctgtgcagcgtctggattcaccttcagtagctatggcatgcactgggtccgccaggctcca
IP_V3-30*p20   ggtccagcctgggggggtccctgagactctcctgtgcagcgtctggattcaccttcagtagctatggcatgcactgggtccgccaggctcca
IP_V3-30*p20   ggtccagcctgggggggtccctgagactctcctgtgcagcgtctggattcaccttcagtagctatggcatgcactgggtccgccaggctcca
IP_V3-30*p21   --------------------------------------------------------------------------------------------
IP_V3-30*p22   ggtccagcctgggggggtccctgagactctcctgtgcagcgtctggattcaccttcagtagctatggcatgcactgggtccgccaggctcca
```

```
IP_V3-30*p23    ggtccagcctggggaggtccctgagactctcctgtgcagcgtctggattcaccttcagtagctatggcatgcactgggtccgccaggctcca
IP_V3-30*p24    ggtccagcctggggaggtccctgagactctcctgtgcagcctctggattcaccttcagtagctatggcatgcactgggtccgccaggctcca
IP_V3-30*p25    ggtccagcctggggaggtctctgagactctcctgtgcagcctctggattcaccttcagtagctatggcatgcactgggtccgccaggctcca
IP_V3-30*p27    ggtccagcctggggaggtccctgagactctcctgtgcagcgtctggattcaccttcagtagctatggcatgcactgggtccgccaggctcca
V_V3-30*03      ggtccagcctggggaggtccctgagactctcctgtgcagcctctggattcaccttcagtagctatggcatgcactgggtccgccaggctcca
V_V3-30*01      ggtccagcctggggaggtccctgagactctcctgtgcagcctctggattcaccttcagtagctatgctatgcactgggtccgccaggctcca
V_V3-30*04      ggtccagcctggggaggtccctgagactctcctgtgcagcctctggattcaccttcagtagctatgctatgcactgggtccgccaggctcca

IGHV3-30-5*01   ggtccagcctggggaggtccctgagactctcctgtgcagcctctggattcaccttcagtagctatggcatgcactgggtccgccaggctcca
IGHV3-30-5*02   ggtccagcctggggggtccctgagactctcctgtgcagcgtctggattcaccttcagtagctatggcatgcactgggtccgccaggctcca
IGHV3-30-22*01  gagacaacctggggaagtccctgagactctcctgtgtagcctctagattcgccttcagtagcttctgaatgagccgagttcaccagtctcca
IGHV3-30-33*01  aagacaacctggggggatccctgagactctcctgtgcagactctggattaaccttcagtagctactgaaggagctcggttcccaggctcca
IGHV3-30-42*01  gagacaacctggggaagtccctgagactctcctgtgtagcctctagattcgccttcagtagcttctgaatgagccagttcaccagtctgca
IGHV3-30-52*01  aagacaacctggggggatccctgagactctcctgtgcagactctggattaaccttcagtagctactgaaggaactcggttcccaggctcca
```

V3-30_01	ggcaaggggctagagtgggtggcagttatatcatatgatggaagtaataaatactacgcagactccgtgaagggccgattcaccatctcc
V3-30_02	ggcaaggggctggagtgggtggcagttatatcatatgatggaagtaataaatactatgcagactccgtgaagggccgattcaccatctcc
V3-30_03	ggcaaggggctggagtgggtggcagttatatcatatgatggaagtaataaatactacgcagactccgtgaagggccgattcaccatctcc
V3-30_04	ggcaaggggctggagtgggtggcagttatatcatatgatggaagtaataaatactatgcagactccgtgaagggccgattcaccatctcc
V3-30_05	ggcaaggggctggagtgggtggcagttatatcatatgatggaagtaataaatactacgcagactccgtgaagggccgattcaccatctcc
V3-30_06	ggcaaggggctggagtgggtggcagttatatcatatgatggaagtaataaatactacgcagactccgtgaagggccgattcaccatctcc
V3-30_07	ggcaaggggctggagtgggtggcagttatatcatatgatggaagtaataaatactatgcagactccgtgaagggccgattcaccatctcc
V3-30_08	ggcaaggggctggagtgggtggcagttatatcatatgatggaagtaataaatactacgcagactccgtgaagggccgattcaccatctcc
V3-30_09	ggcaaggggctggagtgggtggcagttatatcatatgatggaagtaataaatactatgcagactccgtgaagggccgattcaccatctcc
V3-30_10	ggcaaggggctggagtgggtggcagttatatcatatgatggaagtaataaatactacgcagactccgtgaagggccgattcaccatctcc
V3-30_11	ggcaaggggctggagtgggtggcagttatatcatatgatggaagtaataaatactatgcagactccgtgaagggccgattcaccatctcc
V3-30_12	ggcaaggggctggagtgggtggcagttatatcatatgatggaagtaataaatactacgtagactccgtgaagggccgattcaccatctcc
V3-30_13	ggcaaggggctggagtgggtggcagttatatcatatgatggaagtaataaatactacgcagactccgtgaagggccgattcaccatctcc
V3-30_14	ggcaaggggctagagtgggtggcagttatatcatatgatggaagtaataaatactatgcagactccgtgaagggccgattcaccatctcc
V3-30_15	ggcaaggggctggagtgggtggcagttatatcatatgatggaagtaataaatactacgcagactccgtgaagggccgattcaccatctcc
V3-30_16	ggcaaggggctagagtgggtggcagttatatcatatgatggaagtaataaatactacgcagactccgtgaagggccgattcaccatctcc
V3-30_17	ggcaaggggctggagtgggtggcagttatatcatatgatggaagtaataaatactacgcagactccgtgaagggccgattcaccatctcc
V3-30_18	ggcaaggggctagagtgggtggcagttatatcatatgatggaagtaataaatactatgcagactccgtgaagggccgattcaccatctcc
V3-30_19	ggcaaggggctggagtgggtggcagttatatcatatgatggaagtaataaatactatgtagactccgtgaagggccgattcaccatctcc
V3-30_20	ggcaaggggctggagtgggtggcagttatatcatatgatggaagtaataaatactatgcagactccgtgaagggccgattcaccatctcc
V3-30_21	ggcaaggggctggagtgggtggcagttatatcatatgatggaagtaataaatactatgcagactccgtgaagggccgattcaccatctcc
V3-30_22	ggcaaggggctagagtgggtggcagttatatcatatgatggaagtaataaatactatgcagactccgtgaagggccgattcaccatctcc
V3-30_23	ggcaaggggctggagtgggtggcagttatatcatatgatggaagtaataaatactatgcagactccgtgaagggccgattcaccatctcc
V3-30_25	ggcaaggggctagagtgggtggcagttatatcatatgatggaagtaataaatactacgcagactccgtgaagggccgattcaccatctcc
IGHV3-30*01	ggcaaggggctagagtgggtggcagttatatcatatgatggaagtaataaatactacgcagactccgtgaagggccgattcaccatctcc
IGHV3-30*03	ggcaaggggctggagtgggtggcagttatatcatatgatggaagtaataaatactatgcagactccgtgaagggccgattcaccatctcc
IGHV3-30*18	ggcaaggggctggagtgggtggcagttatatcatatgatggaagtaataaatactatgcagactccgtgaagggccgattcaccatctcc
IGHV3-30*02	ggcaaggggctggagtgggtggcatttatacggtatgatggaagtaataaatactatgcagactccgtgaagggccgattcaccatctcc
IGHV3-30*04	ggcaaggggctggagtgggtggcagttatatcatatgatggaagtaataaatactacgcagactccgtgaagggccgattcaccatctcc
IGHV3-30*05	ggcaaggggctagagtgggtggcagttatatcatatgatggaagtaataaatactacgcagactccgtgaagggccgattcaccatctcc
IGHV3-30*06	ggcaaggggctagagtgggtggcagttatatcatatgatggaagtaataaatactacgcagactccgtgaagggccgattcaccatctcc
IGHV3-30*07	ggcaaggggctagagtgggtggcagttatatcatatgatggaagtaataaatactacgcagactccgtgaagggccgattcaccatctcc
IGHV3-30*08	ggcaaggggctagagtgggtggcagttatatcatatgatggaagtaataaatactacgcagactccgtgaagggccgattcaccatctcc
IGHV3-30*09	ggcaaggggctggagtgggtggcagttatatcatatgatggaagtaataaatactacgcagactccgtgaagggccgattcgccatctcc
IGHV3-30*10	ggcaaggggctagagtgggtggcagttatatcatatgatggaagtaataaatactacacagactccgtgaagggccgattcaccatctcc
IGHV3-30*11	ggcaaggggctagagtgggtggcagttatatcatatgatggaagtaataaatactacgcagactccgtgaagggccgattcaccatctcc
IGHV3-30*12	ggcaaggggctagagtgggtggcagttatatcatatgatggaagtaataaatactacgcagactccgtgaagggccgattcaccatctcc
IGHV3-30*13	ggcaaggggctagagtgggtggcagttatatcatatgatggaagtaataaatactacgcagactccgtgaagggccgattcaccatctcc
IGHV3-30*14	ggcaaggggctggagtgggtggcagttatatcatatgatggaagtaataaatactacgcagactccgtgaagggccgattcaccatctcc
IGHV3-30*15	ggcaaggggctagagtgggtggcagttatatcatatgatggaagtaataaatactacgcagactccgtgaagggccgattcaccatctcc
IGHV3-30*16	ggcaaggggctagagtgggtggcagttatatcatatgatggaagtaataaatactacgcagactccgtgaagggccgattcaccatctcc
IGHV3-30*17	ggcaaggggctagagtgggtggcagttatatcatatgatggaagtaataaatactacgcagactccgtgaagggccgattcaccatctcc
IGHV3-30*19	ggcaaggggctggagtgggtggcagttatatcatatgatggaagtaataaatactacgcagactccgtgaagggccgattcaccatctcc
IP_V3-30*p20	ggcaaggggctggagtgggtggcatttatacggtatgatggaagtaataaatactacgcagactccgtgaagggccgattcaccatctcc
IP_V3-30*p20	ggcaaggggctggagtgggtggcatttatacggtatgatggaagtaataaatactacgcagactccgtgaagggccgattcaccatctcc

```
IP_V3-30*p21     ---aaggggctggagtgggtggcagttatatcatatgatggaagtaataaatactacgtagactccgtgaagggccgattcaccatctcc
IP_V3-30*p22     ggcaaggggctggagtgggtggcattttatacagtatgatggaagtaataaatactatgcagactccgtgaagggccgattcaccatctcc
IP_V3-30*p23     ggcaaggggctggagtgggtggcattttatacggtatgatggaagtaataaatactatgcagactccgtgaagggccgattcaccatctcc
IP_V3-30*p24     ggcaaggggctagagtgggtggcagttatatcatatgatggaagtaataaatactacgcagactccgtgaagggccgattcaccatctcc
IP_V3-30*p25     ggcaaggggctggagtgggtggcagttatatcatatgatggaagtaataaatactatgcagactccgtgaagggccgattcaccatctcc
IP_V3-30*p27     ggcaaggggctggagtgggtggcagttatatcatatgatggaagtaataaatactatgcagactccgtgaagggccgattcaccatctcc
V_V3-30*03       ggcaaggggctggagtgggtggcagttatatcatatgatggaagtaataaatactatgcagactccgtgaagggccgattcaccatctcc
V_V3-30*01       ggcaaggggctagagtgggtggcagttatatcatatgatggaagtaataaatactacgcagactccgtgaagggccgattcaccatctcc
V_V3-30*04       ggcaaggggctggagtgggtggcagttatatcatatgatggaagtaataaatactacgcagactccgtgaagggccgattcaccatctcc

IGHV3-30-5*01    ggcaaggggctggagtgggtggcagttatatcatatgatggaagtaataaatactatgcagactccgtgaagggccgattcaccatctcc
IGHV3-30-5*02    ggcaaggggctggagtgggtggcattttatacggtatgatggaagtaataaatactatgcagactccgtgaagggccgattcaccatctcc
IGHV3-30-22*01   ggcaaggggctggagtgagtaatagatataaaagatgatggaagtcagatacaccatgcagactctgtgaagggcagattctccatctcc
IGHV3-30-33*01   gggaaggggctggagtgagtagtagatatacagtgtgatggaagtcagatatgttatgcataatctttgaagagcaaattcaccatctcc
IGHV3-30-42*01   ggcaaggggctggagtgagtaatagatataaaagatgatggaagtcagatacaccatgcagactctgtgaagggcagattctccatctcc
IGHV3-30-52*01   gggaaggggctggagtgagtagtagatatacagtgtgatggaagtcagatatgttatgcataatctttgaagagcaaattcaccatctcc
```

V3-30_01	agagacaattccaagaacacgctgtatctgcaaatgaacagcctgagagctgaggacacggctgtgtattactgtgcgaga
V3-30_02	agagacaattccaagaacacgctgtatctgcaaatgaacagcctgagagctgaggacacggctgtgtattactgtgcgaga
V3-30_03	agagacaattccaagaacacgctgtatctgcaaatgaacagcctgagagctgaggacacggctgtgtattactgtgcgaga
V3-30_04	agagacaattccaagaacacgctgtatctgcaaatgaacagcctgagagctgaggacacggctgtgtattactgtgcgaaa
V3-30_05	agagacaattccaagaacacgctgtatctgcaaatgaacagcctgagagctgaggacacggctgtgtattactgtgcgaaa
V3-30_06	agagacaattccaagaacacgctgtatctgcaaatgaacagcctgagagctgaggacacggctgtgtattactgtgcgaaa
V3-30_07	agagacaattccaagaacacgctgtatctgcaaatgaacagcctgagagctgaggacacggctgtgtattactgtgcgaga
V3-30_08	agagacaattccaagaacacgctgtatctgcaaatgaacagcctgagagctgaggacacggctgtgtattactgtgcgaga
V3-30_09	agagacaattccaagaacacgctgtatctgcaaatgaacagcctgagagctgaggacacggctgtgtattactgtgcgaaa
V3-30_10	agagacaattccaagaacacgctgtatctgcaaatgaacagcctgagagctgaggacacggctgtgtattactgtgcgaga
V3-30_11	agagacaattccaagaacacgctgtatctgcaaatgaacagcctgagagctgaggacacggctgtgtattactgtgcgaaa
V3-30_12	agagacaattccaagaacacgctgtatctgcaaatgaacagcctgagagctgaggacacggctgtgtattactgtgcgaga
V3-30_13	agagacaattccaagaacacgctgtatctgcaaatgaacagcctgagagctgaggacacggctgtgtattactgtgcgaga
V3-30_14	agagacaattccaagaacacgctgtatctgcaaatgaacagcctgagagctgaggacacggctgtgtattactgtgcgaaa
V3-30_15	agagacaattccaagaacacgctgtatctgcaaatgaacagcctgagagctgaggacacggctgtgtattactgtgcgaaa
V3-30_16	agagacaattccaagaacacgctgtatctgcaaatgaacagcctgagagctgaggacacggctgtgtattactgtgcgaaa
V3-30_17	agagacaattccaagaacacgctgtatctgcaaatgaacagcctgagagctgaggacacggctgtgtattactgtgcgaaa
V3-30_18	agagacaattccaagaacacgctgtatctgcaaatgaacagcctgagagctgaggacacggctgtgtattactgtgcgaaa
V3-30_19	agagacaattccaagaacacgctgtatctgcaaatgaacagcctgagagctgaggacacggctgtgtattactgtgcgaaa
V3-30_20	agagacaattccaagaacacgctgtatctgcaaatgaacagcctgagagctgaggacacggctgtgtattactgtgcgaaa
V3-30_21	agagacaattccaagaacacgctgtatctgcaaatgaacagcctgagagctgaggacacggctgtgtattactgtgcgaaa
V3-30_22	agagacaattccaagaacacgctgtatctgcaaatgaacagcctgagagctgaggacacggctgtgtattactgtgcgaga
V3-30_23	agagacaattccaagaacacgctgtatctgcaaatgaacagcctgagagctgaggacacggctgtgtattactgtgcgaga
V3-30_25	agagacaattccaagaacacgctgtatctgcaaatgaacagcctgagagctgaggacacggctgtgtattactgtgcgaaa
IGHV3-30*01	agagacaattccaagaacacgctgtatctgcaaatgaacagcctgagagctgaggacacggctgtgtattactgtgcgaga
IGHV3-30*03	agagacaattccaagaacacgctgtatctgcaaatgaacagcctgagagctgaggacacggctgtgtattactgtgcgaga
IGHV3-30*18	agagacaattccaagaacacgctgtatctgcaaatgaacagcctgagagctgaggacacggctgtgtattactgtgcgaaa
IGHV3-30*02	agagacaattccaagaacacgctgtatctgcaaatgaacagcctgagagctgaggacacggctgtgtattactgtgcgaaa
IGHV3-30*04	agagacaattccaagaacacgctgtatctgcaaatgaacagcctgagagctgaggacacggctgtgtattactgtgcgaga
IGHV3-30*05	agagacaattccaagaacacgctgtatctgcaaatgaacagcctgagagctgagggcacggctgtgtattactgtgcgaga
IGHV3-30*06	agagacaattccaagaacacgctgtatctgcaaatgaacagcctgagagctgaggacacggctgtgtattactgtgcgaga
IGHV3-30*07	agagacaattccaagaacacgctgtatctgcaaatgaacagcctgagagccgaggacacggctgtgtattactgtgcgaga
IGHV3-30*08	agagacaattccaagaacacgctgtatctgcaaatgaacagcctgagagctgaggacacggctgtgtattactgtgcgaga
IGHV3-30*09	agagacaattccaagaacacgctgtatctgcaaatgaacagcctgagagctgaggacacggctgtgtattactgtgcgaga
IGHV3-30*10	agagacaattccaagaacacgctgtatctgcaaatgaacagcctgagagctgaggacacggctgtgtattactgtgcgaga
IGHV3-30*11	agagacaattccaagaacacgctgtatctgcaaatgaacagcctgagagctgaggacacggctgtgtattactgtgcgaga
IGHV3-30*12	agagacaattccaagaacacgctgtatctgcaaatgaacagcctgagagccgaggacacggctgtgtattactgtgcgaga
IGHV3-30*13	agagacaattccaagaacaggctgtatctgcaaatgaacagcctgagagctgaggacacggctgtgtattactgtgcgaga
IGHV3-30*14	agagacaattccaagaacacgctgtatcttcaaatgaacagcctgagagctgaggacacggctgtgtattactgtgcgaga
IGHV3-30*15	agagacaattccaagaacacgctgtatctgcaaatgagcagcctgagagctgaggacacggctgtgtattactgtgcgaga
IGHV3-30*16	agagacaattccaagaacacgctgtatctgcaaatgaacagcctgagagctgaggacacggctgtgtattactgtgcgaga
IGHV3-30*17	agagacaattccaagaacacgctgtatctgcaaatgaacagcctgagagctgaggacacggctgtgtattactgtgcgaga
IGHV3-30*19	agagacaattccaagaacacgctgtatctgcaaatgaacagcctgagagctgaggacacggctgtgtattactgtgcgaga
IP_V3-30*p20	agagacaattccaagaacacgctgtatctgcaaatgaacagcctgagagctgaggacacggctgtgtattactgtgcgaaa
IP_V3-30*p20	agagacaattccaagaacacgctgtatctgcaaatgaacagcctgagagctgaggacacggctgtgtattactgtgcgaaa
IP_V3-30*p21	agagacaattccaagaacacgctgtatctgcaaatgaacagcctgagagctgaggacacggctgtgtattactgtgcgaga

```
IP_V3-30*p22    agagacaattccaagaacacgctgtatctgcaaatgaacagcctgagagctgaggacacggctgtgtattactgtgcgaaa
IP_V3-30*p23    agagacaattccaagaacacgctgtatctgcaaatgaacagcctgagagctgaggacacggctgtgtattactgtgcgaaa
IP_V3-30*p24    agagacaattccaagaacacgctgtatctgcaaatgaacagcctgagagctgaggacacggctgtgtattactgtgcgaaa
IP_V3-30*p25    agagacaattccaagaacacgctgtatctgcaaatgaacagcctgagagctgaggacacggctgtgtattactgtgcga--
IP_V3-30*p27    agagacaattccaagaacacgctgtatctgcaaatgaacagcctgagagctgaggacacggctgtgtattactgtgcgaaa
V_V3-30*03      agagacaattccaagaacacgctgtatctgcaaatgaacagcctgagagctgaggacacggctgtgtattactgtgcgaga
V_V3-30*01      agagacaattccaagaacacgctgtatctgcaaatgaacagcctgagagctgaggacacggctgtgtattactgtgcgaga
V_V3-30*04      agagacaattccaagaacacgctgtatctgcaaatgaacagcctgagagctgaggacacggctgtgtattactgtgcgaga

IGHV3-30-5*01   agagacaattccaagaacacgctgtatctgcaaatgaacagcctgagagctgaggacacggctgtgtattactgtgcgaaa
IGHV3-30-5*02   agagacaattccaagaacacgctgtatctgcaaatgaacagcctgagagctgaggacacggctgtgtattactgtgcgaaa
IGHV3-30-22*01  aaagacaatgctaagaactctctgtatctgcaaatgaacagtcagagagctgaggacatggacgtgtatggctgtacataa
IGHV3-30-33*01  aaagaaaatgccaagaactcactgtatttgctaatgaacagtctgagagcagagggcacagctgtgtgttactgtatgtga
IGHV3-30-42*01  aaagacaatgctaagaactctctgtatctgcaaatgaacagtcagagaactgaggacatggctgtgtatggctgtacataa
IGHV3-30-52*01  aaagaaaatgccaagaactcactgtatttgctaatgaacagtctgagagcagcgggcacagctgtgtgttactgtatgtga
```

IGHV3-33

```
V3-33_01        atggagtttgggctgagctgggttttcctcgttgctcttttaagaggtgtccagtgtcaggtgcagctggtggagtctgggggaggcgtg
V3-33_02        atggagtttgggctgagctgggttttcctcgttgctcttttaagaggtgtccagtgtcaggtgcagctggtggagtctgggggaggcgtg
V3-33_03        atggagtttgggctgagctgggttttcctcgttgctcttttaagaggtgtccagtgtcaggtgcagctggtggagtctgggggaggcgtg
V3-33_04        atggagtttgggctgagctgggttttcctcgttgctcttttaagaggtgtccagtgtcaggtgcagctggtggagtctgggggaggcgtg
V3-33_05        atggagtttgggctgagctgggttttcctcgttgctcttttaagaggtgtccagtgtcaggtgcagctggtggagtctgggggaggcgtg
V3-33_06        atggagtttgggctgagctgggttttcctcgttgctcttttaagaggtgtccagtgtcaggtgcagctggtggagtctgggggaggcgtg
V3-33_07        atggagtttgggctgagctgggttttcctcgttgctcttttaagaggtgtccagtgtcaggtgcagctggtggagtctgggggaggcgtg
V3-33_08        atggagtttgggctgagctgggttttcctcgttgctcttttaagaggtgtccagtgtcaggtgcagctggtggagtctgggggaggcgtg
V3-33_09        atggagtttgggctgagctgggttttcctcgttgctcttttaagaggtgtccagtgtcaggtgcagctggtggagtctgggggaggcgtg
V3-33_10        atggagtttgggctgagctgggttttcctcgttgctcttttaagaggtgtccagtgtcaggtgcagctggtggagtctgggggaggcgtg
V3-33_11        atggagtttgggctgagctgggttttcctcgttgctcttttaagaggtgtccagtgtcaggtgcagctggtggagtctgggggaggcgtg
V3-33_12        atggagtttgggctgagctgggttttcctcgttgctcttttaagaggtgtccagtgtcaggtgcagctggtggagtctgggggaggcgtg
V3-33_13        atggagtttgggctgagctgggttttcctcgttgctcttttaagaggtgtccagtgtcaggtgcagctggtggagtctgggggaggcgtg
V3-33_14        atggagtttgggctgagctgggttttcctcgttgctcttttaagaggtgtccagtgtcaggtgcagctggtggagtctgggggaggcgtg
IGHV3-33*01     atggagtttgggctgagctgggttttcctcgttgctcttttaagaggtgtccagtgtcaggtgcagctggtggagtctgggggaggcgtg
IGHV3-33*02     atggagtttgggctgagctgggttttcctcgttgctcttttaagaggtgtccagtgtcaggtacagctggtggagtctgggggaggcgtg
IGHV3-33*03     ---------------------------------------------------------caggtgcagctggtggagtctgggggaggcgtg
IGHV3-33*04     ---------------------------------------------------------caggtgcagctggtggagtctgggggaggcgtg
IGHV3-33*05     ---------------------------------------------------------caggtgcagctggtggagtctgggggaggcgtg
IGHV3-33*06     ---------------------------------------------------------caggtgcagctggtggagtctgggggaggcgtg
IP_V3-33*p07    ---------------------------------------------------------caggtgcagctggtggagtctgggggaggcgtg
IP_V3-33*p08    ---------------------------------------------------------caggtgcagctggtggagtctgggggaggcgtg
IP_V3-33*p08    ---------------------------------------------------------caggtgcagctggtggagtctgggggaggcgtg
IP_V3-33*p09    ---------------------------------------------------------caggtgcagctggtggagtctgggggaggcgtg
IP_V3-33*p10    ---------------------------------------------------------caggtgcagctggtggagtctgggggaggcgtg
IP_V3-33*p11    ---------------------------------------------------------caggtgcagctggtggagtctgggggaggcgtg
IP_V3-33*p12    ---------------------------------------------------------caggtgcagctggtggagtctgggggaggcgtg
V_V3-33*01      ---------------------------------------------------------caggtgcagctggtggagtctgggggaggcgtg
V_V3-33*02      ---------------------------------------------------------caggtacagctggtggagtctgggggaggcgtg

IGHV3-33-2*01   atggagcttgggctgagctgggttttcactgttgctgttttaaaaggtgtctagtgagaggtacagctcgtggagtccggagaggaccca
```

V3-33_01	gtccagcctgggaggtccctgagactctcctgtgcagcgtctggattcaccttcagtagctatggcatgcactgggtccgccaggctcca
V3-33_02	gtccagcctgggaggtccctgagactctcctgtgcagcgtctggattcaccttcagtagctatggcatgcactgggtccgccaggctcca
V3-33_03	gtccagcctggggggtccctgagactctcctgtgcagcgtctggattcaccttcagtagctatggcatgcactgggtccgccaggctcca
V3-33_04	gtccagcctgggaggtccctgagactctcctgtgcagcctctggattcaccttcagtagctatggcatgcactgggtccgccaggctcca
V3-33_05	gtccagcctggggggtccctgagactctcctgtgcagcgtctggattcaccttcagtagctatggcatgcactgggtccgccaggctcca
V3-33_06	gtccagcctggggggtccctgagactctcctgtgcagcgtctggattcaccttcagtagctatggcatgcactgggtccgccaggctcca
V3-33_07	gtccagcctggggggtccctgagactctcctgtgcagcgtctggattcaccttcagtagctatggcatgcactgggtccgccaggctcca
V3-33_08	gtccagcctgggaggtccctgagactctcctgtgcagcgtctggattcaccttcagtagctatggcatgcactgggtccgccaggctcca
V3-33_09	gtccagcctgggaggtccctgagactctcctgtgcagcgtctggattcaccttcagtagctatggcatgcactgggtccgccaggctcca
V3-33_10	gtccagcctggggggtccctgagactctcctgtgcagcgtctggattcaccttcagtagctatggcatgcactgggtccgccaggctcca
V3-33_11	gtccagcctgggaggtccctgagactctcctgtgcagcctctggattcaccttcagtagctatggcatgcactgggtccgccaggctcca
V3-33_12	gtccagcctgggaggtccctgagactctcctgtgcagcgtctggattcaccttcagtagctatggcatgcactgggtccgccaggctcca
V3-33_13	gtccagcctggggggtccctgagactctcctgtgcagcgtctggattcaccttcagtagctatggcatgcactgggtccgccaggctcca
V3-33_14	gtccagcctggggggtccctgagactctcctgtgcagcgtctggattcaccttcagtagctatggcatgcactgggtccgccaggctcca
IGHV3-33*01	gtccagcctgggaggtccctgagactctcctgtgcagcgtctggattcaccttcagtagctatggcatgcactgggtccgccaggctcca
IGHV3-33*02	gtccagcctgggaggtccctgagactctcctgtgcagcgtctggattcaccttcagtagctatggcatgcactgggtccgccaggctcca
IGHV3-33*03	gtccagcctgggaggtccctgagactctcctgtgcagcgtctggattcaccttcagtagctatggcatgcactgggtccgccaggctcca
IGHV3-33*04	gtccagcctgggaggtccctgagactctcctgtgcagcgtctggattcaccttcagtagctatggcatgcactgggtccgccaggctcca
IGHV3-33*05	gtccagcctgggaggtccctgagactctcctgtgcagcgtctggattcaccttcagtagctatggcatgcactgggtccgccaggctcca
IGHV3-33*06	gtccagcctgggaggtccctgagactctcctgtgcagcgtctggattcaccttcagtagctatggcatgcactgggtccgccaggctcca
IP_V3-33*p07	gtccagcctgggaggtccctgagactctcctgtgcagcctctggattcaccttcagtagctatggcatgcactgggtccgccaggctcca
IP_V3-33*p08	gtccagcctgggaggtccctgagactctcctgtgcagcgtctggattcaccttcagtagctatggcatgcactgggtccgccaggctcca
IP_V3-33*p08	gtccagcctgggaggtccctgagactctcctgtgcagcctctggattcaccttcagtagctatggcatgcactgggtccgccaggctcca
IP_V3-33*p09	gtccagcctgggaggtccctgagactctcctgtgcagcgtctggattcaccttcagtagctatggcatgcactgggtccgccaggctcca
IP_V3-33*p10	gtccagcctgggaggtccctgagactctcctgtgcagcgtctggattcaccttcagtagctatggcatgcactgggtccgccaggctcca
IP_V3-33*p11	gtccagcctgggaggtccctgagactctcctgtgcagcctctggattcaccttcagtagctatggcatgcactgggtccgccaggctcca
IP_V3-33*p12	gtccagcctgggaggtccctgagactctcctgtgcagcctctggattcaccttcagtagctatggcatgcactgggtccgccaggctcca
V_V3-33*01	gtccagcctgggaggtccctgagactctcctgtgcagcgtctggattcaccttcagtagctatggcatgcactgggtccgccaggctcca
V_V3-33*02	gtccagcctgggaggtccctgagactctcctgtgcagcgtctggattcaccttcagtagctatggcatgcactgggtccgccaggctcca
IGHV3-33-2*01	agacaacctgggggatccttgagactctcctgtgcagactctggattaaccttcagtagctactgaatgagctcggtttcccaggctcca

```
V3-33_01          ggcaaggggctggagtgggtggcagttatatggtatgatggaagtaataaatactatgcagactccgtgaagggccgattcaccatctccag
V3-33_02          ggcaaggggctggagtgggtggcagttatatggtatgatggaagtaataaatactatgcagactccgtgaagggccgattcaccatctccag
V3-33_03          ggcaaggggctggagtgggtggcattttatacggtatgatggaagtaataaatactatgcagactccgtgaagggccgattcaccatctccag
V3-33_04          ggcaaggggctggagtgggtggcagttatatggtatgatggaagtaataaatactatgcagactccgtgaagggccgattcaccatctccag
V3-33_05          ggcaaggggctggagtgggtggcattttatacggtatgatggaagtaataaatactacgcagactccgtgaagggccgattcaccatctccag
V3-33_06          ggcaaggggctggagtgggtggcattttatacggtatgatggaagtaataaatactatgcagactccgtgaagggccgattcaccatctccag
V3-33_07          ggcaaggggctggagtgggtggcattttatacggtatgatggaagtaataaatactatgcagactccgtgaagggccgattcaccatctccag
V3-33_08          ggcaaggggctggagtgggtggcattttatacggtatgatggaagtaataaatactatgcagactccgtgaagggccgattcaccatctccag
V3-33_09          ggcaaggggctggagtgggtggcattttatacggtatgatggaagtaataaatactatgcagactccgtgaagggccgattcaccatctccag
V3-33_10          ggcaaggggctggagtgggtggcagttatatggtatgatggaagtaataaatactatgcagactccgtgaagggccgattcaccatctccag
V3-33_11          ggcaaggggctggagtgggtggcagttatatggtatgatggaagtaataaatactatgcagactccgtgaagggccgattcaccatctccag
V3-33_12          ggcaaggggctggagtgggtggcagttatatggtatgatggaagtaataaatactacgcagactccgtgaagggccgattcaccatctccag
V3-33_13          ggcaaggggctggagtgggtggcattttatacggtatgatggaagtaataaatactacgcagactccgtgaagggccgattcaccatctccag
V3-33_14          ggcaaggggctggagtgggtggcagttatatggtatgatggaagtaataaatactatgcagactccgtgaagggccgattcaccatctccag
IGHV3-33*01       ggcaaggggctggagtgggtggcagttatatggtatgatggaagtaataaatactatgcagactccgtgaagggccgattcaccatctccag
IGHV3-33*02       ggcaaggggctggagtgggtggcagttatatggtatgatggaagtaataaatactatgcagactccgcgaagggccgattcaccatctccag
IGHV3-33*03       ggcaaggggctggagtgggtggcagttatatggtatgatggaagtaataaatactatgcagactccgtgaagggccgattcaccatctccag
IGHV3-33*04       ggcaaggggctagagtgggtggcagttatatggtatgacggaagtaataaatactatgcagactccgtgaagggccgattcaccatctccag
IGHV3-33*05       ggcaaggggctggagtgggtggcagttatatcatatgatggaagtaataaatactatgcagactccgtgaagggccgattcaccatctccag
IGHV3-33*06       ggcaaggggctggagtgggtggcagttatatggtatgatggaagtaataaatactatgcagactccgtgaagggccgattcaccatctccag
IP_V3-33*p07      ggcaaggggctggagtgggtggcagttatatggtatgatggaagtaataaatactatgcagactccgtgaagggccgattcaccatctccag
IP_V3-33*p08      ggcaaggggctggagtgggtggcagttatatggtatgatggaagtaataaatactatgcagactccgtgaagggccgattcaccatctccag
IP_V3-33*p08      ggcaaggggctggagtgggtggcagttatatggtatgatggaagtaataaatactatgcagactccgtgaagggccgattcaccatctccag
IP_V3-33*p09      ggcaaggggctggagtgggtggcagttatatggtatgatggaagtaataaatactatgcagactccgtgaagggccgattcaccatctccag
IP_V3-33*p10      ggcaaggggctggagtgggtggcagttatatggtatgatggaagtaataaatactatgcagactccgtgaagggccgattcaccatctccag
IP_V3-33*p11      ggcaaggggctggagtgggtggcagttatatggtatgatggaagtaataaatactatgcagactccgtgaagggccgattcaccatctccag
IP_V3-33*p12      ggcaaggggctggagtgggtggcagttatatggtatgatggaagtaataaatactatgcagactccgtgaagggccgattcaccatctccag
V_V3-33*01        ggcaaggggctggagtgggtggcagttatatggtatgatggaagtaataaatactatgcagactccgtgaagggccgattcaccatctccag
V_V3-33*02        ggcaaggggctggagtgggtggcagttatatggtatgatggaagtaataaatactatgcagactccgcgaagggccgattcaccatctccag


IGHV3-33-2*01     gggaaggggctggagtgagtagtagatatacagtgtgatggaagtcagatatgttatgccatctgtgaagagcaaattcaccatctccaa
```

V3-33_01	agacaattccaagaacacgctgtatctgcaaatgaacagcctgagagccgaggacacggctgtgtattactgtgcgaga
V3-33_02	agacaattccaagaacacgctgtatctgcaaatgaacagcctgagagccgaggacacggctgtgtattactgtgcgaaa
V3-33_03	agacaattccaagaacacgctgtatctgcaaatgaacagcctgagagccgaggacacggctgtgtattactgtgcgaaa
V3-33_04	agacaattccaagaacacgctgtatctgcaaatgaacagcctgagagccgaggacacggctgtgtattactgtgcgaga
V3-33_05	agacaattccaagaacacgctgtatctgcaaatgaacagcctgagagccgaggacacggctgtgtattactgtgcgaaa
V3-33_06	agacaattccaagaacacgctgtatctgcaaatgaacagcctgagagccgaggacacggctgtgtattactgtgcgaga
V3-33_07	agacaattccaagaacacgctgtatctgcaaatgaacagcctgagagccgaggacacggctgtgtattactgtgcgaga
V3-33_08	agacaattccaagaacacgctgtatctgcaaatgaacagcctgagagccgaggacacggctgtgtattactgtgcgaga
V3-33_09	agacaattccaagaacacgctgtatctgcaaatgaacagcctgagagccgaggacacggctgtgtattactgtgcgaaa
V3-33_10	agacaattccaagaacacgctgtatctgcaaatgaacagcctgagagccgaggacacggctgtgtattactgtgcgaga
V3-33_11	agacaattccaagaacacgctgtatctgcaaatgaacagcctgagagccgaggacacggctgtgtattactgtgcgaaa
V3-33_12	agacaattccaagaacacgctgtatctgcaaatgaacagcctgagagccgaggacacggctgtgtattactgtgcgaaa
V3-33_13	agacaattccaagaacacgctgtatctgcaaatgaacagcctgagagccgaggacacggctgtgtattactgtgcgaga
V3-33_14	agacaattccaagaacacgctgtatctgcaaatgaacagcctgagagccgaggacacggctgtgtattactgtgcgaaa
IGHV3-33*01	agacaattccaagaacacgctgtatctgcaaatgaacagcctgagagccgaggacacggctgtgtattactgtgcgaga
IGHV3-33*02	agacaattccacgaacacgctgtttctgcaaatgaacagcctgagagccgaggacacggctgtgtattactgtgcgaga
IGHV3-33*03	agacaactccaagaacacgctgtatctgcaaatgaacagcctgagagccgaggacacggctgtgtattactgtgcgaaa
IGHV3-33*04	agacaattccaagaacacgctgtatctgcaaatgaacagcctgagagccgaggacacggctgtgtattactgtgcgaga
IGHV3-33*05	agacaattccaagaacacgctgtatctgcaaatgaacagcctgagagccgaggacacggctgtgtattactgtgcgaga
IGHV3-33*06	agacaattccaagaacacgctgtatctgcaaatgaacagcctgagagccgaggacacggctgtgtattactgtgcgaaa
IP_V3-33*p07	agacaattccaagaacacgctgtatctgcaaatgaacagcctgagagccgaggacacggctgtgtattactgtgcgaga
IP_V3-33*p08	agacaattccaagaacacgctgtatctgcaaatgaacagcctgagagctgaggacacggctgtgtattactgtgcgaaa
IP_V3-33*p08	agacaattccaagaacacgctgtatctgcaaatgaacagcctgagagctgaggacacggctatgtattactgtgcgaaa
IP_V3-33*p09	agacaattccaagaacacgctgtatctgcaaatgaacagcctgagagccgaggacacggctgtgtattactgtactaaa
IP_V3-33*p10	agacaattccaagaacacgctgtatctgcaaatgaacagcctgagagccgaggacacggctgtgtattactgtaataaa
IP_V3-33*p11	agacaattccaagaacacgctgtatctgcaaatgaacagcctgagagccgaggacacggct------------------
IP_V3-33*p12	agacaattccaagaacacgctgtatctgcaaatgaacagcctgagagctgaggacacggctgtgtattactgtgcgaaa
V_V3-33*01	agacaattccaagaacacgctgtatctgcaaatgaacagcctgagagccgaggacacggctgtgtattactgtgcgaga
V_V3-33*02	agacaattccacgaacacgctgtttctgcaaatgaacagcctgagagccgaggacacggctgtgtattactgtgcgaga

IGHV3-33-2*01	agaaaatgccaagaactcactgtatttgcaaatgaacagtctgagagcagagggcacagctgtgtgttactgtatgtga

IGHV3-35

```
V3-35_01        atggaatttgggctgagctgggttttcttgctgctattttaaaaggtgtccagtgtgaggtgcagctggtggagtctgggggaggcttggta
V3-35_02        atggaatttgggctgagctgggttttcttgctgctattttaaaaggtgtccagtgtgaggtgcagctggtggagtctgggggaggcttggta
V3-35_03        atggaatttgggctgagctgggttttcttgctgctattttaaaaggtgtccagtgtgaggtgcagctggtggagtctgggggaggcttggta
V3-35_04        atggaatttgggctgagctgggttttcttgctgctattttaaaaggtgtccagtgtgaggtgcagctggtggagtctgggggaggcttggta
V3-35_05        atggaatttgggctgagctgggttttcttgctgctattttaaaaggtgtccagtgtgaggtgcagctggtggagtctgggggaggcttggta
V3-35_06        atggaatttgggctgagctgggttttcttgctgctattttaaaatgtgtccagtgtgaggtgcagctggtggagtctgggggaggcttggta
IGHV3-35*01     atggaatttggcctgagctgggttttccttgctgctattttaaaaggtgtccagtgtgaggtgcagctggtggagtctgggggaggcttggta
V_V3-35*01      ----------------------------------------------------------gaggtgcagctggtggagtctgggggaggcttggta
```

```
V3-35_01        cagcctggggggatccctgagactctcctgtgcagcctctggattcaccttcagtaacagtgacatgaactgggtccatcaggctcca
V3-35_02        cagcctggggggatccctgagactctcctgtgcagcctctggattcaccttcagtaacagtgacatgaactgggtccatcaggctcca
V3-35_03        cagcctggggggatccctgagactctcctgtgcagcctctggattcaccttcagtaacagtgacatgaactgggtccatcaggctcca
V3-35_04        cagcctggggggatccctgagactctcctgtgcagcctctggattcaccttcagtaacagtgacatgaactgggtccatcaggctcca
V3-35_05        cagcctggggggatccctgagactctcctgtgcagcctctggattcaccttcagtaacagtgacatgaactgggtccatcaggctcca
V3-35_06        cagcctggggggatccctgagactctcctgtgcagcctctggattcaccttcagtaacagtgacatgaactgggtccatcaggctcca
IGHV3-35*01     cagcctggggggatccctgagactctcctgtgcagcctctggattcaccttcagtaacagtgacatgaactgggtccatcaggctcca
V_V3-35*01      cagcctggggggatccctgagactctcctgtgcagcctctggattcaccttcagtaacagtgacatgaactgggtccatcaggctcca
```

```
V3-35_01        ggaaaggggctggagtgggtatcgggtgttagttggaatggcagtaggacgcactatgcagactctgtgaagggccgattcatcatctccagaga
V3-35_02        ggaaaggggctggagtgggtatcgggtgttagttggaatggcagtaggacgcactatgcagactctgtgaagggccaattcatcatctccagaga
V3-35_03        ggaaagcggctggagtgggtatcgggtgttagttggaatggcagtaggacgcactatgcagactctgtgaagggccgattcatcatctccagaga
V3-35_04        ggaaagcggctggagtgggtatcgggtgttagttggaatggcagtaggacgcactatgcagactctgtgaagggccaattcatcatctccagaga
V3-35_05        ggaaaggggctggagtgggtattgggtgttagttggaatggcagtaggacgcactatgcagactctgtgaagggccaattcatcatctccagaga
V3-35_06        ggaaaggggctggagtgggtatcgggtgttagttggaatggcagtaggacgcactatgcagactctgtgaagggccgattcatcatctccagaga
IGHV3-35*01     ggaaaggggctggagtgggtatcgggtgttagttggaatggcagtaggacgcactatgcagactctgtgaagggccgattcatcatctccagaga
V_V3-35*01      ggaaaggggctggagtgggtatcgggtgttagttggaatggcagtaggacgcactatgcagactctgtgaagggccgattcatcatctccagaga
```

```
V3-35_01        caattccaggaacaccctgtatctgcaaacgaatagcctgagggccgaggacacggctgtgtattactgtgtgaga
V3-35_02        caattccaggaacaccctgtatctgcaaacgaatagcctgagggccgaggacacggctgtgtattactgtgtgaga
V3-35_03        caattccaggaacaccctgtatctgcaaacgaatagcctgagggccgaggacacggctgtgtattactgtgtgaga
V3-35_04        caattccaggaacaccctgtatctgcaaacgaatagcctgagggccgaggacacggctgtgtattactgtgtgaga
V3-35_05        caattccaggaacaccctgtatctgcaaacgaatagcctgagggccgaggacacggctgtgtattactgtgtgaga
V3-35_06        caattccaggaacaccctgtatctgcaaacgaatagcctgagggccgaggacacggctgtgtattactgtgtgaga
IGHV3-35*01     caattccaggaacaccctgtatctgcaaacgaatagcctgagggccgaggacacggctgtgtattactgtgtgaga
V_V3-35*01      caattccaggaacaccctgtatctgcaaacgaatagcctgagggccgaggacacggctgtgtattactgtgtgaga
```

IGHV3-38

```
V3-38_01       atgcagtttgtgctgagctgggttttcctttgttggtattttaaaaggtgtccagtgtgaggtgcagctggtggagtctgggggaggcttggt
V3-38_02       atgcagtttgtgctgagctgggttttcctttgttggtattttaaaaggtgtccagtgtgaggtgcagctggtggagtctgggggaggcttggt
V3-38_03       atgcagtttgtgctgagctgggttttcctttgttggtattttaaaaggtgtccagtgtgaggtgcagctggtggagtctgggggaggcttggt
V3-38_04       atgcagtttgtgctgagctgggttttcctttgttggtattttaaaaggtgtccagtgtgaggtgcagctggtggagtctgggggaggcttggt
V3-38_05       atgcagtttgtgctgagctgggttttcctttgttggtattttaaaaggtgtccagtgtgaggtgcagctggtggagtctgggggaggcttggt
V3-38_06       atgcagtttgtgctgagctgggttttcctttgttggtattttaaaaggtgtccagtgtgaggtgcagctggtggagtctgggggaggcttggt
V3-38_07       atgcagtttgtgctgagctgggttttcctttgttggtattttaaaaggtgtccagtgtgaggtgcagctggtggagtctgggggaggcttggt
V3-38_08       atgcagtttgtgctgagctgggttttcccttgttggtattttaaaaggtgtccagtgtgaggtgcagctggtggagtctgggggaggcttggt
V3-38_09       atgcagtttgtgctgagctgggttttcctttgttggtattttaaaaggtgtccagtgtgaggtgcagctggtggagtctgggggaggcttcgt
V3-38_10       atgcagtttgtgctgagctgggttttcctttgttggtattttaaaaggtgtccagtgtgaggtgcagctggtggagtctgggggaggcttcgt
V3-38_11       atgcagtttgtgctgagctgggttttcctttgttggtattttaaaaggtgtccagtgtgaggtgcagctggtggagtctgggggaggcttcgt
IGHV3-38*01    atgcagtttgtgctgagctgggttttcctttgttggtattttaaaaggtgtccagtgtgaggtgcagctggtggagtctgggggaggcttggt
IGHV3-38*02    atgcagtttgtgctgagctgggttttcctttgttggtattttaaaaggtgtccagtgtgaggtgcagctggtggagtctgggggaggcttggt
IGHV3-38*03    atgcagtttgtgctgagctgggttttcctttgttggtattttaaaaggtgtccagtgtgaggtgcagctggtggagtctgggggaggcttggt
IGHV3-38-3*01  atgcagtttgtgctgagctgggttttcctttgttgctattttaaaaggtgtccagtgtgaggtgcagctggtggagtctcggggagtcttggt
V_V3-38*01     ----------------------------------------------------------gaggtgcagctggtggagtctgggggaggcttggt
V_V3-38*02     ----------------------------------------------------------gaggtgcagctggtggagtctgggggaggcttggt
```

```
V3-38_01       acagcctaggggtccctgagactctcctgtgcagcctctggattcaccgtcagtagcaatgagatgagctggatccgccaggctcca
V3-38_02       acagcctaggggtccctgagactctcctgtgcagcctctggattcaccgtcagtagcaatgagatgagctggatccgccaggctcca
V3-38_03       acagcctaggggtccctgagactctcctgtgcagcctctggattcaccgtcagtagcaatgagatgagctggatccgccaggctcca
V3-38_04       acagcctaggggtccctgagactctcctgtgcagcctctggattcaccgtcagtagcaatgagatgagctggatccgccaggctcca
V3-38_05       acagcctaggggtccctgagactctcctgtgcagcctctggattcaccgtcagtagcaatgagatgagctggatccgccaggctcca
V3-38_06       acagcctaggggtccctgagactctcctgtgcagcctctggattcaccgtcagtagcaatgagatgagctggatccgccaggctcca
V3-38_07       acagcctaggggtccctgagactctcctgtgcagcctctggattcaccgtcagtagcaatgagatgagctggatccgccaggctcca
V3-38_08       acagcctaggggtccctgagactctcctgtgcagcctctggattcaccgtcagtagcaatgagatgagctggatccgccaggctcca
V3-38_09       acagcctaggggtccctgagactctcctgtgcagcctctggattcaccgtcagtagcaatgagatgagctggatccgccaggctcca
V3-38_10       acagcctaggggtccctgagactctcctgtgcagcctctggattcaccgtcagtagcaatgagatgagctggatccgccaggctcca
V3-38_11       acagcctaggggtccctgagactctcctgtgcagcctctggattcaccgtcagtagcaatgagatgagctggatccgccaggctcca
IGHV3-38*01    acagcctaggggtccctgagactctcctgtgcagcctctggattcaccgtcagtagcaatgagatgagctggatccgccaggctcca
IGHV3-38*02    acagcctaggggtccctgagactctcctgtgcagcctctggattcaccgtcagtagcaatgagatgagctggatccgccaggctcca
IGHV3-38*03    acagcctaggggtccctgagactctcctgtgcagcctctggattcaccgtcagtagcaatgagatgagctggatccgccaggctcca
IGHV3-38-3*01  acagcctgggggtccctgagactctcctgtgcagcctctggattcaccgtcagtagcaatgagatgagctgggtccgccaggctcca
V_V3-38*01     acagcctaggggtccctgagactctcctgtgcagcctctggattcaccgtcagtagcaatgagatgagctggatccgccaggctcca
V_V3-38*02     acagcctaggggtccctgagactctcctgtgcagcctctggattcaccgtcagtagcaatgagatgagctggatccgccaggctcca
```

```
V3-38_01       gggaagggg ctggagtgggtctcatccattagtggtggtagcacatactacgcagactccaggaagggcagattcaccatctccagagacaat
V3-38_02       gggaaggg tctggagtgggtctcatccattagtggtggtagcacatactacgcagactccaggaagggcagattcaccatctccagagacaat
```

```
V3-38_01       gggaaggggctggagtgggtctcatccattagtggtggtagcacatactacgcagactccaggaagggcagattcaccatctccagagacaat
V3-38_02       gggaagggtctggagtgggtctcatccattagtggtggtagcacatactacgcagactccaggaagggcagattcaccatctccagagacaat
V3-38_03       gggaaggggctggagtgggtctcatccattagtggtggtagcacatactacgcagactccaggaagggcagattcaccatctccagagacaat
V3-38_04       gggaagggtctggagtgggtctcatccattagtggtggtagcacatactacgcagactccaggaagggcagattcaccatctccagagacaat
V3-38_05       gggaaggggctggagtgggtctcatccattagtggtggtagcacatactacgcagactccaggaagggcagattcaccatctccagagacaat
V3-38_06       gggaagggtctggagtgggtctcatccattagtggtggtagcacatactacgcagactccaggaagggcagattcaccatctccagagacaat
V3-38_07       gggaagggtctggagtgggtctcatccattagtggtggtagcacatactacgcagactccgtgaagggcagattcaccatctccagagacaat
V3-38_08       gggaaggggctggagtgggtctcatccattagtggtggtagcacatactacgcagactccaggaagggcagattcaccatctccagagacaat
V3-38_09       gggaaggggctggagtgggtctcatccattagtggtggtagcacatactacgcagactccaggaagggcagattcaccatctccagagacaat
V3-38_10       gggaaggggctggagtgggtctcatccattagtggtggtagcacatactacgcagactccaggaagggcagattcaccatctccagagacaat
V3-38_11       gggaaggggctggagtgggtctcatccattagtggtggtagcacatactacgcagactccaggaagggcagattcaccatctccagagacaat
IGHV3-38*01    gggaaggggctggagtgggtctcatccattagtggtggtagcacatactacgcagactccaggaagggcagattcaccatctccagagacaat
IGHV3-38*02    gggaaggggctggagtgggtctcatccattagtggtggtagcacatactacgcagactccaggaagggcagattcaccatctccagagacaat
IGHV3-38*03    gggaagggtctggagtgggtctcatccattagtggtggtagcacatactacgcagactccaggaagggcagattcaccatctccagagacaat
IGHV3-38-3*01  gggaagggtctggagtgggtctcatccattagtggtggtagcacatactacgcagactccaggaagggcagattcaccatctccagagacaat
V_V3-38*01     gggaaggggctggagtgggtctcatccattagtggtggtagcacatactacgcagactccaggaagggcagattcaccatctccagagacaat
V_V3-38*02     gggaaggggctggagtgggtctcatccattagtggtggtagcacatactacgcagactccaggaagggcagattcaccatctccagagacaat

V3-38_01       tccaagaacacgctgtatcttcaaatgaacaacctgagagctgagggcacggccgtgtattactgtgccagatat
V3-38_02       tccaagaacacgctgtatcttcaaatgaacaacctgagagctgagggcacggccgtgtattactgtgccagatat
V3-38_03       tccaagaacacgctgtatcttcaaatgaacagcctgagagctgagggcacggccgtgtattactgtgccagatat
V3-38_04       tccaagaacacgctgtatcttcaaatgaacagcctgagagctgagggcacggccgtgtattactgtgccagatat
V3-38_05       tccaagaacacgctgtatcttcaaatgaagagcctgagagctgagggcacggccgtgtattactgtgccagatat
V3-38_06       tccaagaacacgctgtatcttcaaatgaagagcctgagagctgagggcacggccgtgtattactgtgccagatat
V3-38_07       tccaagaacacgctgtatcttcaaatgaacagcctgagagctgagggcacggccgtgtattactgtgccagatat
V3-38_08       tccaagaacacgctgtatcttcaaatgaacaacctgagagctgagggcacggccgtgtattactgtgccagatat
V3-38_09       tccaagaacacgctgtatcttcaaatgaacaacctgagagctgagggcacggccgtgtattactgtgccagatat
V3-38_10       tccaagaacacgctgtatcttcaaatgaagagcctgagagctgagggcacggccgtgtattactgtgccagatat
V3-38_11       tccaagaacacgctgtatcttcaaatgaacagcctgagagctgagggcacggccgtgtattactgtgccagatat
IGHV3-38*01    tccaagaacacgctgtatcttcaaatgaacaacctgagagctgagggcacggccgcgtattactgtgccagatat
IGHV3-38*02    tccaagaacacgctgtatcttcaaatgaacaacctgagagctgagggcacggccgtgtattactgtgccagatat
IGHV3-38*03    tccaagaacacgctgtatcttcaaatgaacaacctgagagctgagggcacggccgtgtattactgtgccagatat
IGHV3-38-3*01  tccaagaacacgctgcatcttcaaatgaacagcctgagagctgaggacacggctgtgtattactgtaagaaga-
V_V3-38*01     tccaagaacacgctgtatcttcaaatgaacaacctgagagctgagggcacggccgcgtattactgtgccaga---
V_V3-38*02     tccaagaacacgctgtatcttcaaatgaacaacctgagagctgagggcacggccgtgtattactgtgccaga---
```

IGHV3-43

```
V3-43_01         atggagtttggactgagctgggttttccttgttgctattttaaaaggtgtccagtgtgaagtgcagctggtggagtctgggggagtcgtggt
V3-43_02         atggagtttggactgagctgggttttccttgttgctattttaaaaggtgtccagtgtgaagtgcagctggtggagtctgggggagtcgtggt
V3-43_03         atggagtttggactgagctgggttttccttgttgctattttaaaaggtgtccagtgtgaagtgcagctggtggagtctgggggaggcgtggt
V3-43_04         atggagtttggactgagctgggttttccttgttgctattttaaaaggtgtccagtgtgaagtgcagctggtggagtctgggggaggcgtggt
V3-43_05         atggagtttggactgagctgggttttccttgttgctattttaaaaggtgtccagtgtgaagtgcagctggtggagtctgggggagtcgtggt
V3-43_06         atggagtttggactgagctgggttttccttgttgctattttaaaaggtgtccagtgtgaagtgcagctggtggagtctgggggagtcgtggt
V3-43_07         atggagtttggactgagctgggttttccttgttgctattttaaaaggtgtccagtgtgaagtgcagctggtggagtctgggggagtcgtggt
V3-43_08         atggagtttggactgagctgggttttccttgttgctattttaaaaggtgtccagtgtgaagtgcagctggtggagtctgggggagtcgtggt
V3-43_10         atggagtttggactgagctgggttttccttgttgctattttaaaaggtgtccagtgtgaagtgcagctggtggagtctgggggagtcgtggt
V3-43_11         atggagtttggactgagctgggttttccttgttgctattttaaaaggtgtccagtgtgaaatgcagctggtggagtctgggggagtcgtggt
IGHV3-43*01      atggagtttggactgagctgggttttccttgttgctattttaaaaggtgtccagtgtgaagtgcagctggtggagtctgggggagtcgtggt
IGHV3-43*02      ---------------------------------------------------------gaagtgcagctggtggagtctgggggaggcgtggt
IGHV3-43D*03     atggagtttggactgagctgggttttccttgttgctattttaaaaggtgtccagtgtgaagtgcagctggtggagtctgggggagtcgtggt
IGHV3-43D*04     atggagtttggactgagctgggttttccttgttgctattttaaaaggtgtccagtgtgaagtgcagctggtggagtctgggggagtcgtggt
IP_V3-43*p03     ---------------------------------------------------------gaagtgcagctggtggagtctgggggaggcgtggt
IP_V3-43*p04     ---------------------------------------------------------gaagtgcagctggtggagtctgggggagtcgtggt
V_V3-43*01       ---------------------------------------------------------gaagtgcagctggtggagtctgggggagtcgtggt
```

```
V3-43_01         acagcctggggggtccctgagactctcctgtgcagcctctggattcacctttgatgattataccatgcactgggtccgtcaagctccg
V3-43_02         acagcctggggggtccctgagactctcctgtgcagcctctggattcacctttgatgattataccatgcactgggtccgtcaagctccg
V3-43_03         acagcctggggggtccctgagactctcctgtgcagcctctggattcacctttgatgattataccatgcactgggtccgtcaagctcca
V3-43_04         acagcctggggggtccctgagactctcctgtgcagcctctggattcacctttgatgattataccatgcactgggtccgtcaagctcca
V3-43_05         acagcctggggggtccctgagactctcctgtgcagcctctggattcacctttgatgattataccatgcactgggtccgtcaagctccg
V3-43_06         acagcctggggggtccctgagactctcctgtgcagcctctggattcacctttgatgattataccatgcactgggtccgtcaagctccg
V3-43_07         acagcctggggggtccctgagactctcctgtgcagcctctggattcacctttgatgattataccatgcactgggtccgtcaagctccg
V3-43_08         acagcctggggggttcctgagactctcctgtgcagcctctggattcacctttgatgattataccatgcactgggtccgtcaagctccg
V3-43_10         acagcctggggggttcctgagactctcctgtgcagcctctggattcacctttgatgattataccatgcactgggtccgtcaagctccg
V3-43_11         acagcctggggggtccctgagactctcctgtgcagcctctggattcacctttgatgattataccatgcactgggtccgtcaagctccg
IGHV3-43*01      acagcctggggggtccctgagactctcctgtgcagcctctggattcacctttgatgattataccatgcactgggtccgtcaagctccg
IGHV3-43*02      acagcctggggggtccctgagactctcctgtgcagcctctggattcacctttgatgattatgccatgcactgggtccgtcaagctcca
IGHV3-43D*03     acagcctggggggtccctgagactctcctgtgcagcctctggattcacctttgatgattatgccatgcactgggtccgtcaagctccg
IGHV3-43D*04     acagcctggggggtccctgagactctcctgtgcagcctctggattcacctttgatgattatgccatgcactgggtccgtcaagctccg
IP_V3-43*p03     acagcctggggggtccctgagactctcctgtgcagcctctggattcacctttgatgattatgccatgcactgggtccgtcaagctcca
IP_V3-43*p04     acagcctggggggtccctgagactctcctgtgcagcctctggattcacctttgatgattatgccatgcactgggtccgtcaagctccg
V_V3-43*01       acagcctggggggtccctgagactctcctgtgcagcctctggattcacctttgatgattataccatgcactgggtccgtcaagctccg
```

V3-43_01	gggaagggtctggagtgggtctctcttattagttgggatggtggtagcacatactatgcagactctgtgaagggccgattcaccatctccaga
V3-43_02	gggaagggtctggagtgggtctctcttattagttgggatggtggtagcacctactatgcagactctgtgaagggccgattcaccatctccaga
V3-43_03	gggaagggtctggagtgggtctctcttattagtggggatggtggtagcacatactatgcagactctgtgaagggccgattcaccatctccaga
V3-43_04	gggaagggtctggagtgggtctctcttattagttgggatggtggtagcacatactatgcagactctgtgaagggccgattcaccatctccaga
V3-43_05	gggaagggtctggagtgggtctctcttattagtggggatggtggtagcacatactatgcagactctgtgaagggccgattcaccatctccaga
V3-43_06	gggaagggtctggagtgggtctctcttattagttgggatggtggtagcacatactatgcagactctgtgaagggccgattcaccatctccaga
V3-43_07	gggaagggtctggagtgggtctctcttattagtggggatggtggtagcacctactatgcagactctgtgaagggccgattcaccatctccaga
V3-43_08	gggaagggtctggagtgggtctctcttattagttgggatggtggtagcacatactatgcagactctgtgaagggccgattcaccatctccaga
V3-43_10	gggaagggtctggagtgggtctctcttattagttgggatggtggtagcacctactatgcagactctgtgaagggccgattcaccatctccaga
V3-43_11	gggaagggtctggagtgggtctctcttattagttgggatggtggtagcacatactatgcagactctgtgaagggccgattcaccatctccaga
IGHV3-43*01	gggaagggtctggagtgggtctctcttattagttgggatggtggtagcacatactatgcagactctgtgaagggccgattcaccatctccaga
IGHV3-43*02	gggaagggtctggagtgggtctctcttattagtggggatggtggtagcacatactatgcagactctgtgaagggccgattcaccatctccaga
IGHV3-43D*03	gggaagggtctggagtgggtctctcttattagttgggatggtggtagcacctactatgcagactctgtgaagggtcgattcaccatctccaga
IGHV3-43D*04	gggaagggtctggagtgggtctctcttattagttgggatggtggtagcacatactatgcagactctgtgaagggtcgattcaccatctccaga
IP_V3-43*p03	gggaagggtctggagtgggtctctcttattagtggggatggtggtagcacatactatgcagactctgtgaagggccgattcaccatctccaga
IP_V3-43*p04	gggaagggtctggagtgggtctctcttattagttgggatggtggtagcacatactatgcagactctgtgaagggtcgattcaccatctccaga
V_V3-43*01	gggaagggtctggagtgggtctctcttattagttgggatggtggtagcacatactatgcagactctgtgaagggccgattcaccatctccaga

V3-43_01	gacaacagcaaaaactccctgtatctgcaaatgaacagtctgagaactgaggacaccgccttgtattactgtgcaaaagat-
V3-43_02	gacaacagcaaaaactccctgtatctgcaaatgaacagtctgagaactgaggacaccgccttgtattactgtgcaaaagat-
V3-43_03	gacaacagcaaaaactccctgtatctgcaaatgaacagtctgagaactgaggacaccgccttgtattactgtgcaaaagat-
V3-43_04	gacaacagcaaaaactccctgtatctgcaaatgaacagtctgagaactgaggacaccgccttgtattactgtgcaaaagat-
V3-43_05	gacaacagcaaaaactccctgtatctgcaaatgaacagtctgagaactgaggacaccgccttgtattactgtgcaaaagat-
V3-43_06	gacaacagcaaaaactccctgtatctgcaaatgaacagtctgagaactgaggacaccaccttgtattactgtgcaaaagat-
V3-43_07	gacaacagcaaaaactccctgtatctgcaaatgaacagtctgagaactgaggacaccgccttgtattactgtgcaaaagat-
V3-43_08	gacaacagcaaaaactccctgtatctgcaaatgaacagtctgagaactgaggacaccgccttgtattactgtgcaaaagat-
V3-43_10	gacaacagcaaaaactccctgtatctgcaaatgaacagtctgagaactgaggacaccgccttgtattactgtgcaaaagat-
V3-43_11	gacaacagcaaaaactccctgtatctgcaaatgaacagtctgagaactgaggacaccgccttgtattactgtgcaaaagat-
IGHV3-43*01	gacaacagcaaaaactccctgtatctgcaaatgaacagtctgagaactgaggacaccgccttgtattactgtgcaaaagata
IGHV3-43*02	gacaacagcaaaaactccctgtatctgcaaatgaacagtctgagaactgaggacaccgccttgtattactgtgcaaaagata
IGHV3-43D*03	gacaacagcaaaaactccctgtatctgcaaatgaacagtctgagagctgaggacaccgccttgtattactgtgcaaaagata
IGHV3-43D*04	gacaacagcaaaaactccctgtatctgcaaatgaacagtctgagagctgaggacaccgccttgtattactgtgcaaaagata
IP_V3-43*p03	gacaacagcaaaaactccctgtatctgcaaatgaacagtctgagaactgaggacaccgccttgtattactgtgcaaaagata
IP_V3-43*p04	gacaacagcaaaaactccctgtatctgcaaatgaacagtctgagagctgaggaca-------------------------
V_V3-43*01	gacaacagcaaaaactccctgtatctgcaaatgaacagtctgagaactgaggacaccgccttgtattactgtgcaaaa----

IGHV3-48

```
V3-48_01       atggagttggggctgtgctgggttttccttgttgctattttagaaggtgtccagtgtgaggtgcagctggtggagtctgggggaggcttggt
V3-48_02       atggagttggggctgtgctgggttttccttgttgctattttagaaggtgtccagtgtgaggtgcagctggtggagtctgggggaggcttggt
V3-48_03       atggagttggggctgtgctgggttttccttgttgctattttagaaggtgtccagtgtgaggtgcagctggtggagtctgggggaggcttggt
V3-48_04       atggagttggggctgtgctgggttttccttgttgctattttagaaggtgtccagtgtgaggtgcagctggtggagtctgggggaggcttggt
V3-48_05       atggagttggggctgtgctgggttttccttgttgctattttagaaggtgtccagtgtgaggtgcagctggtggagtctgggggaggcttggt
V3-48_06       atggagttggggctgtgctgggttttccttgttgctattttagaaggtgtccagtgtgaggtgcagctggtggagtctgggggaggcttggt
V3-48_07       atggagttggggctgtgctgggttttccttgttgctattttagaaggtgtccagtgtgaggtgcagctggtggagtctgggggaggcttggt
V3-48_08       atggagttggggctgtgctgggttttccttgttgctattttagaaggtgtccagtgtgaggtgcagctggtggagtctgggggaggcttggt
V3-48_09       atggagttggggctgtgctgggttttccttgttgctattttagaaggtgtccagtgtgaggtgcagctggtggagtctgggggaggcttggt
V3-48_10       atggagttggggctgtgctgggttttccttgttgctattttagaaggtgtccagtgtgaggtgcagctggtggagtctgggggaggcttggt
V3-48_11       atggagttggggctgtgctgggttttccttgttgctattttagaaggtgtccagtgtgaggtgcagctggtggagtctgggggaggcttggt
V3-48_12       atggagttggggctgtgctgggttttccttgttgctattttagaaggtgtccagtgtgaggtgcagctggtggagtctgggggaggcttggt
IGHV3-48*01    atggagttggggctgtgctgggttttccttgttgctattttagaaggtgtccagtgtgaggtgcagctggtggagtctgggggaggcttggt
IGHV3-48*02    atggagttggggctgtgctgggttttccttgttgctattttagaaggtgtccagtgtgaggtgcagctggtggagtctgggggaggcttggt
IGHV3-48*03    atggagttggggctgtgctgggttttccttgttgctattttagaaggtgtccagtgtgaggtgcagctggtggagtctgggggaggcttggt
IGHV3-48*04    ---------------------------------------------------------gaggtgcagctggtggagtctgggggaggcttggt
IP_V3-48*p05   ---------------------------------------------------------gaggtgcagctggtggagtctgggggaggcttggt
V_V3-48*01     ---------------------------------------------------------gaggtgcagctggtggagtctgggggaggcttggt
V_V3-48*02     ---------------------------------------------------------gaggtgcagctggtggagtctgggggaggcttggt
V_V3-48*03     ---------------------------------------------------------gaggtgcagctggtggagtctgggggaggcttggt
```

```
V3-48_01       acagcctggggggtccctgagactctcctgtgcagcctctggattcaccttcagtagctatagcatgaactgggtccgccaggctcca
V3-48_02       acagcctggggggtccctgagactctcctgtgcagcctctggattcaccttcagtagctatagcatgaactgggtccgccaggctcca
V3-48_03       acagcctggggggtccctgagactctcctgtgcagcctctggattcaccttcagtagctatagcatgaactgggtccgccaggctcca
V3-48_04       acagcctggggggtccctgagactctcctgtgcagcctctggattcaccttcagtagttatgaaatgaactgggtccgccaggctcca
V3-48_05       acagcctggggggtccctgagactctcctgtgcagcctctggattcaccttcagtagttatgaaatgaactgggtccgccaggctcca
V3-48_06       acagcctggggggtccctgagactctcctgtgcagcctctggattcaccttcagtagttatgaaatgaactgggtccgccaggctcca
V3-48_07       acagcctggggggtccctgagactctcctgtgcagcctctggattcaccttcagtagttatgaaatgaactgggtccgccaggctcca
V3-48_08       acagcctggggggtccctgagactctcctgtgcagcctctggattcaccttcagtagctatagcatgaactgggtccgccaggctcca
V3-48_09       acagcctggggggtccctgagactctcctgtgcagcctctggattcaccttcagtagctatagcatgaactgggtccgccaggctcca
V3-48_10       acagcctggggggtccctgagactctcctgtgcagcctctggattcaccttcagtagctatagcatgaactgggtccgccaggctcca
V3-48_11       acagcctggggggtccctgagactctcctgtgcagcctctggattcaccttcagtagttatgaaatgaactgggtccgccaggctcca
V3-48_12       acagcctggggggtccctgagactctcctgtgcagcctctggattcaccttcagtagctatagcatgaactgggtccgccaggctcca
IGHV3-48*01    acagcctggggggtccctgagactctcctgtgcagcctctggattcaccttcagtagctatagcatgaactgggtccgccaggctcca
IGHV3-48*02    acagcctggggggtccctgagactctcctgtgcagcctctggattcaccttcagtagctatagcatgaactgggtccgccaggctcca
IGHV3-48*03    acagcctggagggtccctgagactctcctgtgcagcctctggattcaccttcagtagttatgaaatgaactgggtccgccaggctcca
IGHV3-48*04    acagcctggggggtccctgagactctcctgtgcagcctctggattcaccttcagtagctatagcatgaactgggtccgccaggctcca
IP_V3-48*p05   acagcctggagggtccctgagactctcctgtgcagcctctggattcaccttcagtagttatgaaatgaactgggtccgccaggctcca
V_V3-48*01     acagcctggggggtccctgagactctcctgtgcagcctctggattcaccttcagtagctatagcatgaactgggtccgccaggctcca
V_V3-48*02     acagcctggggggtccctgagactctcctgtgcagcctctggattcaccttcagtagctatagcatgaactgggtccgccaggctcca
V_V3-48*03     acagcctggagggtccctgagactctcctgtgcagcctctggattcaccttcagtagttatgaaatgaactgggtccgccaggctcca
```

```
V3-48_01      gggaaggggctggagtgggtttcatacattagtagtagtagtagtaccatatactacgcagactctgtgaagggccgattcaccatctccaga
V3-48_02      gggaaggggctggagtgggtttcatacattagtagtagtagtagtaccatatactacgcagactctgtgaagggccgattcaccatctccaga
V3-48_03      gggaaggggctggagtgggtttcatacattagtagtagtagtagtaccatatactacgcagactctgtgaagggccgattcaccatctccaga
V3-48_04      gggaaggggctggagtgggtttcatacattagtagtagtagtagtaccatatactacgcagactctgtgaagggccgattcaccatctccaga
V3-48_05      gggaaggggctggagtgggtttcatacattagtagtagtagtagtaccatatactacgcagactctgtgaagggccgattcaccatctccaga
V3-48_06      gggaaggggctggagtgggtttcatacattagtagtagtagtagtaccatatactacgcagactctgtgaagggccgattcaccatctccaga
V3-48_07      gggaaggggctggagtgggtttcatacattagtagtagtagtagttacacaaactacgcagactctgtgaagggccgattcaccatctccaga
V3-48_08      gggaaggggctggagtgggtttcatacattagtagtagtagtagttacacaaactacgcagactctgtgaagggccgattcaccatctccaga
V3-48_09      gggaaggggctggagtgggtttcatacattagtagtagtagtagttacacaaactacgcagactctgtgaagggccgattcaccatctccaga
V3-48_10      gggaaggggctggagtgggtttcatacattagtagtagtagtagttacacaaactacgcagactctgtgaagggccgattcaccatctccaga
V3-48_11      gggaaggggctggagtgggtttcatacattagtagtagtagtagtaccatatactacgcagactctgtgaagggccgattcaccatctccaga
V3-48_12      gggaaggggctggagtgggtttcatacattagtagtagtagtagtaccatatactacgcagactctgtgaagggccgattcaccatctccaga
IGHV3-48*01   gggaaggggctggagtgggtttcatacattagtagtagtagtagtaccatatactacgcagactctgtgaagggccgattcaccatctccaga
IGHV3-48*02   gggaaggggctggagtgggtttcatacattagtagtagtagtagtaccatatactacgcagactctgtgaagggccgattcaccatctccaga
IGHV3-48*03   gggaaggggctggagtgggtttcatacattagtagtagtggtagtaccatatactacgcagactctgtgaagggccgattcaccatctccaga
IGHV3-48*04   gggaaggggctggagtgggtttcatacattagtagtagtagtagtaccatatactacgcagactctgtgaagggccgattcaccatctccaga
IP_V3-48*p05  gggaaggggctggagtgggtttcatacattagtagtagtggtagtaccatatactacgcagactctgtgaagggccgattcaccatctccaga
V_V3-48*01    gggaaggggctggagtgggtttcatacattagtagtagtagtagtaccatatactacgcagactctgtgaagggccgattcaccatctccaga
V_V3-48*02    gggaaggggctggagtgggtttcatacattagtagtagtagtagtaccatatactacgcagactctgtgaagggccgattcaccatctccaga
V_V3-48*03    gggaaggggctggagtgggtttcatacattagtagtagtggtagtaccatatactacgcagactctgtgaagggccgattcaccatctccaga

V3-48_01      gacaatgccaagaactcactgtatctgcaaatgaacagcctgagagccgaggacacggctgtgtattactgtgcgaga
V3-48_02      gacaatgccaagaactcactgtatctgcaaatgaacagcctgagaacgaggacacggctgtgtattactgtgcgaga
V3-48_03      gacaacgccaagaactcactgtatctgcaaatgaacagcctgagagccgaggacacggctgtgtattactgtgcgaga
V3-48_04      gacaacgccaagaactcactgtatctgcaaatgaacagcctgagagccgaggacacggctgtgtattactgtgcgaga
V3-48_05      gacaatgccaagaactcactgtatctgcaaatgaacagcctgagaacgaggacacggctgtgtattactgtgcgaga
V3-48_06      gacaatgccaagaactcactgtatctgcaaatgaacagcctgagagccgaggacacggctgtgtattactgtgcgaga
V3-48_07      gacaacgccaagaactcactgtatctgcaaatgaacagcctgagagccgaggacacggctgtgtattactgtgcgaga
V3-48_08      gacaacgccaagaactcactgtatctgcaaatgaacagcctgagagccgaggacacggctgtgtattactgtgcgaga
V3-48_09      gacaatgccaagaactcactgtatctgcaaatgaacagcctgagaacgaggacacggctgtgtattactgtgcgaga
V3-48_10      gacaatgccaagaactcactgtatctgcaaatgaacagcctgagagccgaggacacggctgtgtattactgtgcgaga
V3-48_11      gacaacgccaagaactcactgtatctgcaaatgaacagcctgagaacgaggacacggctgtgtattactgtgcgaga
V3-48_12      gacaacgccaagaactcactgtatctgcaaatgaacagcctgagagccgaggacatggctgtgtattactgtgcgaga
IGHV3-48*01   gacaatgccaagaactcactgtatctgcaaatgaacagcctgagagccgaggacacggctgtgtattactgtgcgaga
IGHV3-48*02   gacaatgccaagaactcactgtatctgcaaatgaacagcctgagaacgaggacacggctgtgtattactgtgcgaga
IGHV3-48*03   gacaacgccaagaactcactgtatctgcaaatgaacagcctgagagccgaggacacggctgtttattactgtgcgaga
IGHV3-48*04   gacaacgccaagaactcactgtatctgcaaatgaacagcctgagagccgaggacacggctgtgtattactgtgcgaga
IP_V3-48*p05  gacaacgccaagaactcactgtatctgcaaatgaacagcctgagagccgaggacacggctgtgtattactgtgtgaaa
V_V3-48*01    gacaatgccaagaactcactgtatctgcaaatgaacagcctgagagccgaggacacggctgtgtattactgtgcgaga
V_V3-48*02    gacaatgccaagaactcactgtatctgcaaatgaacagcctgagaacgaggacacggctgtgtattactgtgcgaga
V_V3-48*03    gacaacgccaagaactcactgtatctgcaaatgaacagcctgagagccgaggacacggctgtttattactgtgcgaga
```

IGHV3-49

```
V3-49_01      atggagtttgggcttagctgggttttccttgttgctattttaaaaggtgtccaatgtgaggtgcagctggtggagtctgggggaggcttgg
V3-49_02      atggagtttgggcttagctgggttttccttgttgctattttaaaaggtgtccaatgtgaggtgcagctggtggagtctgggggaggcttgg
V3-49_03      atggagtttgggcttagctgggttttccttgttgctattttaaaaggtgtccaatgtgaggtgcagctggtggagtctgggggaggcttgg
V3-49_04      atggagtttgggcttagctgggttttccttgttgctattttaaaaggtgtccaatgtgaggtgcagctggtggagtctgggggaggcttgg
V3-49_05      atggagtttgggcttagctgggttttccttgttgctattttaaaaggtgtccaatgtgaggtgcagctggtggagtctgggggaggcttgg
IGHV3-49*01   atggagtttgggcttagctgggttttccttgttgctattttaaaaggtgtccaatgtgaggtgcagctggtggagtctgggggaggcttgg
IGHV3-49*02   ---------------------------------gctattttaaaaggtgtccaatgtgaggtgcagctggtggagtctgggggaggcttgg
IGHV3-49*03   atggagtttgggcttagctgggttttccttgttgctattttaaaaggtgtccaatgtgaggtgcagctggtggagtctgggggaggcttgg
IGHV3-49*04   ---------------------------------------------------------gaggtgcagctggtggagtctgggggaggcttgg
IGHV3-49*05   ---------------------------------------------------------gaggtgcagctggtggagtctgggggaggcttgg
V_V3-49*03    ---------------------------------------------------------gaggtgcagctggtggagtctgggggaggcttgg

V3-49_01      tacagccagggcggtccctgagactctcctgtacagcttctggattcacctttggtgattatgctatgagctggttccgccaggctcca
V3-49_02      tacagccagggcggtccctgagactctcctgtacagcttctggattcacctttggtgattatgctatgagctgggtccgccaggctcca
V3-49_03      taaagccagggcggtccctgagactctcctgtacagcttctggattcacctttggtgattatgctatgagctggttccgccaggctcca
V3-49_04      taaagccagggcggtccctgagactctcctgtacagcttctggattcacctttggtgattatgctatgagctgggtccgccaggctcca
V3-49_05      tacagccagggcggtccctgagactctcctgtacagcttctggattcacctttggtgattatgctatgagctgggtccgccaggctcca
IGHV3-49*01   tacagccagggcggtccctgagactctcctgtacagcttctggattcacctttggtgattatgctatgagctggttccgccaggctcca
IGHV3-49*02   tacagccagggccgtccctgagactctcctgtacagcttctggattcacctttgggtattatcctatgagctgggtccgccaggctcca
IGHV3-49*03   tacagccagggcggtccctgagactctcctgtacagcttctggattcacctttggtgattatgctatgagctggttccgccaggctcca
IGHV3-49*04   tacagccagggcggtccctgagactctcctgtacagcttctggattcacctttggtgattatgctatgagctgggtccgccaggctcca
IGHV3-49*05   taaagccagggcggtccctgagactctcctgtacagcttctggattcacctttggtgattatgctatgagctggttccgccaggctcca
V_V3-49*03    tacagccagggcggtccctgagactctcctgtacagcttctggattcacctttggtgattatgctatgagctggttccgccaggctcca

V3-49_01      gggaaggggctggagtgggtaggtttcattagaagcaaagcttatggtgggacaacagaatacgccgcgtctgtgaaaggcagattcaccat
V3-49_02      gggaaggggctggagtgggtaggtttcattagaagcaaagcttatggtgggacaacagaatacgccgcgtctgtgaaaggcagattcaccat
V3-49_03      gggaaggggctggagtgggtaggtttcattagaagcaaagcttatggtgggacaacagaatacgccgcgtctgtgaaaggcagattcaccat
V3-49_04      gggaaggggctggagtgggtaggtttcattagaagcaaagcttatggtgggacaacagaatacgccgcgtctgtgaaaggcagattcaccat
V3-49_05      gggaaggggctggagtgggtaggtttcattagaagcaaagcttatggtgggacaacagaatacgccgcgtctgtgaaaggcagattcaccat
IGHV3-49*01   gggaaggggctggagtgggtaggtttcattagaagcaaagcttatggtgggacaacagaatacaccgcgtctgtgaaaggcagattcaccat
IGHV3-49*02   gggaaggggctggagtgggtaggtttcattagaagcaaagcttatggtgggacaacagaatacgccgcgtctgtgaaaggcagattcaccat
IGHV3-49*03   gggaaggggctggagtgggtaggtttcattagaagcaaagcttatggtgggacaacagaatacgccgcgtctgtgaaaggcagattcaccat
IGHV3-49*04   gggaaggggctggagtgggtaggtttcattagaagcaaagcttatggtgggacaacagaatacgccgcgtctgtgaaaggcagattcaccat
IGHV3-49*05   gggaaggggctggagtgggtaggtttcattagaagcaaagcttatggtgggacaacagaatacgccgcgtctgtgaaaggcagattcaccat
V_V3-49*03    gggaaggggctggagtgggtaggtttcattagaagcaaagcttatggtgggacaacagaatacgccgcgtctgtgaaaggcagattcaccat
```

V3-49_01	ctcaagagatggattccaaaagcatcgcctatctgcaaatgaacagcctgaaaaccgaggacacagccgtgtattactgtactaga
V3-49_02	ctcaagagatggattccaaaagcatcgcctatctgcaaatgaacagcctgaaaaccgaggacacagccgtgtattactgtactaga
V3-49_03	ctcaagagatggattccaaaagcatcgcctatctgcaaatgaacagcctgaaaaccgaggacacagccgtgtattactgtactaga
V3-49_04	ctcaagagatggattccaaaagcatcgcctatctgcaaatgaacagcctgaaaaccgaggacacagccgtgtattactgtactaga
V3-49_05	ctcaagagat-gattccaaaagcatcgcctatctgcaaatgaacagcctgaaaaccgaggacacagccgtgtattactgtactaga
IGHV3-49*01	ctcaagagat-ggttccaaaagcatcgcctatctgcaaatgaacagcctgaaaaccgaggacacagccgtgtattactgtactaga
IGHV3-49*02	ctcaagagat-gattccaaaagcatcgcctatctgcaaatgaacagcctgaaaaccgaggacacagccgtgtattactgtactaga
IGHV3-49*03	ctcaagagat-gattccaaaagcatcgcctatctgcaaatgaacagcctgaaaaccgaggacacagccgtgtattactgtactaga
IGHV3-49*04	ctcaagagat-gattccaaaagcatcgcctatctgcaaatgaacagcctgaaaaccgaggacacagccgtgtattactgtactaga
IGHV3-49*05	ctcaagagat-gattccaaaagcatcgcctatctgcaaatgaacagcctgaaaaccgaggacacagccgtgtattactgtactaga
V_V3-49*03	ctcaagagat-gattccaaaagcatcgcctatctgcaaatgaacagcctgaaaaccgaggacacagccgtgtattactgtactaga

IGHV3-53

V3-53_01	atggagttttggctgagctgggttttccttgttgctatttaaaaaggtgtccagtgtgaggtgcagctggtggagtctggaggaggctt
V3-53_02	atggagttttggctgagctgggttttccttgttgctatttcaaaaggtgtccagtgtgaggtgcagctggtggagactggaggaggctt
V3-53_03	atggagttttggctgagctgggttttccttgttgctatttaaaaaggtgtccagtgtgaggtgcagctggtggagtctggaggaggctt
V3-53_04	atggagttttggctgagctgggttttccttgttgctatttaaaaaggtgtccagtgtgaggtgcagctggtggagtctggggaggctt
V3-53_05	atggagttttggctgagctgggttttccttgttgctatttaaaaaggtgtccagtgtgaggtgcagctggtggagtctggggaggctt
V3-53_06	atggagttttggctgagctgggttttccttgttgctatttcaaaaggtgtccagtgtgaggtgcagctggtggagactggggaggctt
V3-53_07	atggagttttggctgagctgggttttccttgttgctatttcaaaaggtgtccagtgtgaggtgcagctggtggagactggaggaggctt
V3-53_08	atggagttttggctgagctgggttttccttgttgctatttcaaaaggtgtccagtgtgaggtgcagctggtggagactggggaggctt
V3-53_09	atggagttttggctgagctgggttttccttgttgctatttaaaaaggtgtccagtgtgaggtgcagctggtggagtctggaggaggctt
V3-53_10	atggagttttggctgagctgggttttccttgttgctatttcaaaaggtgtccagtgtgaggtgcagctggtggagtctggaggaggctt
V3-53_11	atggagttttggctgagctgggttttccttgttgctatttcaaaaggtgtccagtgtgaggtgcagctggtggagtctggggaggctt
V3-53_12	atggagttttggctgagctgggttttccttgttgctatttcaaaaggtgtccagtgtgaggtgcagctggtggagtctggaggaggctt
V3-53_13	atggagttttggctgagctgggttttccttgttgctatttcaaaaggtgtccagtgtgaggtgcagctggtggagactggaggaggctt
V3-53_14	atggagttttggctgagctgggttttccttgttgctatttaaaaaggtgtccagtgtgaggtgcagctggtggagtctggaggaggctt
V3-53_15	atggagttttggctgagctgggttttccttgttgctatttaaaaaggtgtccagtgtgaggtgcagctggtggagtctggggaggctt
V3-53_16	atggagttttggctgagctgggttttccttgttgctatttcaaaaggtgtccagtgtgaggtgcagctggtggagtctggggaggctt
V3-53_17	atggagttttggctgagctgggttttccttgttgctatttaaaaaggtgtccagtgtgaggtgcagctggtggagtctggggaggctt
V3-53_18	atggagttttggctgagctgggttttccttgttgctatttaaaaaggtgtccagtgtgaggtgcagctggtggagtctggaggaggctt
V3-53_19	atggagttttggctgagctgggttttccttgttgctatttaaaaaggtgtccagtgtgaggtgcagctggtggagtctggggaggctt
IGHV3-53*01	atggagttttggctgagctgggttttccttgttgctatttaaaaaggtgtccagtgtgaggtgcagctggtggagtctggaggaggctt
IGHV3-53*02	atggagttttggctgagctgggttttccttgttgctatttcaaaaggtgtccagtgtgaggtgcagctggtggagactggaggaggctt
IGHV3-53*03	atggagttttggctgagctgggttttccttgttgctatttaaaaaggtgtccagtgtgaggtgcagctggtggagtctggaggaggctt
IGHV3-53*04	---gaggtgcagctggtggagtctggaggaggctt
IP_V3-53*p05	---gaggtgcagctggtggagtctggaggaggctt
IP_V3-53*p06	---gaggtgcagctggtggagtctggaggaggctt
IP_V3-53*p07	--
V_V3-53*01	---gaggtgcagctggtggagtctggaggaggctt
V_V3-53*02	---gaggtgcagctggtggagactggaggaggctt
V_V3-53*03	---gaggtgcagctggtggagtctggaggaggctt

```
V3-53_01        gatccagcctggggggtccctgagactctcctgtgcagcctctgggttcaccgtcagtagcaactacatgagctgggtccgccaggctcca
V3-53_02        gatccagcctggggggtccctgagactctcctgtgcagcctctgggttcaccgtcagtagcaactacatgagctgggtccgccaggctcca
V3-53_03        ggtccagcctggggggtccctgagactctcctgtgcagcctctgggttcaccgtcagtagcaactacatgagctgggtccgccaggctcca
V3-53_04        ggtccagcctggggggtccctgagactctcctgtgcagcctctgggttcaccgtcagtagcaactacatgagctgggtccgccaggctcca
V3-53_05        gatccagcctggggggtccctgagactctcctgtgcagcctctgggttcaccgtcagtagcaactacatgagctgggtccgccaggctcca
V3-53_06        ggtccagcctggggggtccctgagactctcctgtgcagcctctgggttcaccgtcagtagcaactacatgagctgggtccgccaggctcca
V3-53_07        ggtccagcctggggggtccctgagactctcctgtgcagcctctgggttcaccgtcagtagcaactacatgagctgggtccgccaggctcca
V3-53_08        gatccagcctggggggtccctgagactctcctgtgcagcctctgggttcaccgtcagtagcaactacatgagctgggtccgccaggctcca
V3-53_09        gatccagcctggggggtccctgagactctcctgtgcagcctctgggttcaccgtcagtagcaactacatgagctgggtccgccaggctcca
V3-53_10        gatccagcctggggggtccctgagactctcctgtgcagcctctgggttcaccgtcagtagcaactacatgagctgggtccgccaggctcca
V3-53_11        ggtccagcctggggggtccctgagactctcctgtgcagcctctgggttcaccgtcagtagcaactacatgagctgggtccgccaggctcca
V3-53_12        ggtccagcctggggggtccctgagactctcctgtgcagcctctgggttcaccgtcagtagcaactacatgagctgggtccgccaggctcca
V3-53_13        gatccagcctggggggtccctgagactctcctgtgcagcctctgggttcaccgtcagtagcaactacatgagctgggtccgccaggctcca
V3-53_14        ggtccagcctggggggtccctgagactctcctgtgcagcctctgggttcaccgtcagtagcaactacatgagctgggtccgccaggctcca
V3-53_15        gatccagcctggggggtccctgagactctcctgtgcagcctctgggttcaccgtcagtagcaactacatgagctgggtccgccaggctcca
V3-53_16        gatccagcctggggggtccctgagactctcctgtgcagcctctgggttcaccgtcagtagcaactacatgagctgggtccgccaggctcca
V3-53_17        ggtccagcctggggggtccctgagactctcctgtgcagcctctgggttcaccgtcagtagcaactacatgagctgggtccgccaggctcca
V3-53_18        gatccagcctggggggtccctgagactctcctgtgcagcctctgggttcaccgtcagtagcaactacatgagctgggtccgccaggctcca
V3-53_19        ggtccagcctggggggtccctgagactctcctgtgcagcctctgggttcaccgtcagtagcaactacatgagctgggtccgccaggctcca
IGHV3-53*01     gatccagcctggggggtccctgagactctcctgtgcagcctctgggttcaccgtcagtagcaactacatgagctgggtccgccaggctcca
IGHV3-53*02     gatccagcctggggggtccctgagactctcctgtgcagcctctgggttcaccgtcagtagcaactacatgagctgggtccgccaggctcca
IGHV3-53*03     gatccagcctggggggtccctgagactctcctgtgcagcctctgggttcaccgtcagtagcaactacatgagctgggtccgccagcctcca
IGHV3-53*04     ggtccagcctggggggtccctgagactctcctgtgcagcctctgggttcaccgtcagtagcaactacatgagctgggtccgccaggctcca
IP_V3-53*p05    gatccagcctggggggtccctgagactctcctgtgcagcctctgggttcaccgtcagtagcaactacatgagctgggtccgccaggctcca
IP_V3-53*p06    gatccagcctggggggtccctgagactctcctgtgcagcctctgggttcaccgtcagtagcaactacatgagctgggtccgccaggctcca
IP_V3-53*p07    ---------------------------------tgcagcctctgggttcaccatcagtagcaactacatgagctgggtccgccaggctcca
V_V3-53*01      gatccagcctggggggtccctgagactctcctgtgcagcctctgggttcaccgtcagtagcaactacatgagctgggtccgccaggctcca
V_V3-53*02      gatccagcctggggggtccctgagactctcctgtgcagcctctgggttcaccgtcagtagcaactacatgagctgggtccgccaggctcca
V_V3-53*03      gatccagcctggggggtccctgagactctcctgtgcagcctctgggttcaccgtcagtagcaactacatgagctgggtccgccagcctcca
```

```
V3-53_01        gggaaggggctggagtgggtctcagttatttatagcggtggtagcacatactacgcagactccgtgaagggccgattcaccatctccagag
V3-53_02        gggaaggggctggagtgggtctcagttatttatagcggtggtagcacatactacgcagactccgtgaagggccgattcaccatctccagag
V3-53_03        gggaaggggctggagtgggtctcagttatttatagcggtggtagcacatactacgcagactccgtgaagggccgattcaccatctccagag
V3-53_04        gggaaggggctggagtgggtctcagttatttatagcggtggtagcacatactacgcagactccgtgaagggccgattcaccatctccagag
V3-53_05        gggaaggggctggagtgggtctcagttatttatagcggtggtagcacatactacgcagactccgtgaagggccgattcaccatctccagag
V3-53_06        gggaaggggctggagtgggtctcagttatttatagcggtggtagcacatactacgcagactccgtgaagggccgattcaccatctccagag
V3-53_07        gggaaggggctggagtgggtctcagttatttatagcggtggtagcacatactacgcagactccgtgaagggccgattcaccatctccagag
V3-53_08        gggaaggggctggagtgggtctcagttatttatagcggtggtagcacatactacgcagactccgtgaagggccgattcaccatctccagag
V3-53_09        gggaaggggctggagtgggtctcagttatttatagcggtggtagcacatactacgcagactccgtgaagggccgattcaccatctccagag
V3-53_10        gggaaggggctggagtgggtctcagttatttatagcggtggtagcacatactacgcagactccgtgaagggccgattcaccatctccagag
V3-53_11        gggaaggggctggagtgggtctcagttatttatagcggtggtagcacatactacgcagactccgtgaagggccgattcaccatctccagag
V3-53_12        gggaaggggctggagtgggtctcagttatttatagcggtggtagcacatactacgcagactccgtgaagggccgattcaccatctccagag
V3-53_13        gggaaggggctggagtgggtctcagttatttatagcggtggtagcacatactacgcagactccgtgaagggccgattcaccatctccagag
V3-53_14        gggaaggggctggagtgggtctcagttatttatagcggtggtagcacatactacgcagactccgtgaagggccgattcaccatctccagag
V3-53_15        gggaaggggctggagtgggtctcagttatttatagcggtggtagcacatactacgcagactccgtgaagggccgattcaccatctccagag
V3-53_16        gggaaggggctggagtgggtctcagttatttatagcggtggtagcacatactacgcagactccgtgaagggccgattcaccatctccagag
V3-53_17        gggaaggggctggagtgggtctcagttatttatagcggtggtagcacatactacgcagactccgtgaagggccgattcaccatctccagag
V3-53_18        gggaaggggctggagtgggtctcagttatttatagcggtggtagcacatactatgcagactccgtgaagggccgattcaccatctccagag
V3-53_19        gggaaggggctggagtgggtctcagttatttatagcggtggtagcacatactatgcagactccgtgaagggccgattcaccatctccagag
IGHV3-53*01     gggaaggggctggagtgggtctcagttatttatagcggtggtagcacatactacgcagactccgtgaagggccgattcaccatctccagag
IGHV3-53*02     gggaaggggctggagtgggtctcagttatttatagcggtggtagcacatactacgcagactccgtgaagggccgattcaccatctccagag
IGHV3-53*03     gggaaggggctggagtgggtctcagttatttatagcggtggtagcacatactacgcagactctgtgaagggccgattcaccatctccagag
IGHV3-53*04     gggaaggggctggagtgggtctcagttatttatagcggtggtagcacatactacgcagactccgtgaagggccgattcaccatctccagac
IP_V3-53*p05    gggaaggggctggagtgggtctcagttatttatagcggtggtagcacatactacgcagactccgtgaagggccgattcaccatctccagag
IP_V3-53*p06    gggaaggggctggagtgggtctcagttatttatagcggtggtagcacatactacgcagactccgtgaagggccgattcaccatctccagac
IP_V3-53*p07    gggaaggggctggagtgggtctcagttatttatagcggtggtagcacatactacgcagactccgtgaagggccgattcaccatctccagag
V_V3-53*01      gggaaggggctggagtgggtctcagttatttatagcggtggtagcacatactacgcagactccgtgaagggccgattcaccatctccagag
V_V3-53*02      gggaaggggctggagtgggtctcagttatttatagcggtggtagcacatactacgcagactccgtgaagggccgattcaccatctccagag
V_V3-53*03      gggaaggggctggagtgggtctcagttatttatagcggtggtagcacatactacgcagactctgtgaagggccgattcaccatctccagag
```

```
V3-53_01       acaattccaagaacacgctgtattcttcaaatgaacagcctgagagccgaggacacggccgtgtattactgtgcgaga
V3-53_02       acaattccaagaacacgctgtattcttcaaatgaacagcctgagagccgaggacacggccgtgtattactgtgcgaga
V3-53_03       acaattccaagaacacgctgtattcttcaaatgaacagcctgagagccgaggacacggccgtgtattactgtgcgaga
V3-53_04       acaattccaagaacacgctgtattcttcaaatgaacagcctgagagccgaggacacggccgtgtattactgtgcgaga
V3-53_05       acaattccaagaacacgctgtattcttcaaatgaacagcctgagagccgaggacacggccgtgtattactgtgcgaga
V3-53_06       acaattccaagaacacgctgtattcttcaaatgaacagcctgagagccgaggacacggccgtgtattactgtgcgaga
V3-53_07       acaattccaagaacacgctgtattcttcaaatgaacagcctgagagccgaggacacggccgtgtattactgtgcgaga
V3-53_08       acaattccaagaacacgctgtattcttcaaatgaacagcctgagagccgaggacacggccgtgtattactgtgcgaga
V3-53_09       acaattccaagaacacgctgtattcttcaaataaacagcctgagagccgaggacacggccgtgtattactgtgcgaga
V3-53_10       acaattccaagaacacgctgtattcttcaaatgaacagcctgagagccgaggacacggccgtgtattactgtgcgaga
V3-53_11       acaattccaagaacacgctgtattcttcaaatgaacagcctgagagccgaggacacggccgtgtattactgtgcgaga
V3-53_12       acaattccaagaacacgctgtattcttcaaatgaacagcctgagagccgaggacacggccgtgtattactgtgcgaga
V3-53_13       acaattccaagaacacgctgta-tcttcaaatgaacagcctgagagccgaggacacggccgtgtattactgtgcgaga
V3-53_14       acaattccaagaacacgctgtattcttcaaataaacagcctgagagccgaggacacggccgtgtattactgtgcgaga
V3-53_15       acaattccaagaacacgctgtattcttcaaataaacagcctgagagccgaggacacggccgtgtattactgtgcgaga
V3-53_16       acaattccaagaacacgctgtattcttcaaatgaacagcctgagagccgaggacacggccgtgtattactgtgcgaga
V3-53_17       acaattccaagaacacgctgtattcttcaaataaacagcctgagagccgaggacacggccgtgtattactgtgcgaga
V3-53_18       acaattccaagaacacgctgtattcttcaaatgaacagcctgagagccgaggacacggccgtgtattactgtgcgaga
V3-53_19       acaattccaagaacacgctgtattcttcaaatgaacagcctgagagccgaggacacggccgtgtattactgtgcgaga
IGHV3-53*01    acaattccaagaacacgctgta-tcttcaaatgaacagcctgagagccgaggacacggccgtgtattactgtgcgaga
IGHV3-53*02    acaattccaagaacacgctgta-tcttcaaatgaacagcctgagagccgaggacacggccgtgtattactgtgcgaga
IGHV3-53*03    acaattccaagaacacgctgta-tcttcaaatgaacagcctgagagccgaggacacggccgtgtattactgtgctagg
IGHV3-53*04    acaattccaagaacacgctgta-tcttcaaatgaacagcctgagagctgaggacacggccgtgtattactgtgcgaga
IP_V3-53*p05   acaattccaagaacacgctgta-tcttcaaatgaacagcctgagagccgaggacacggccgtgtattactgtgctaga
IP_V3-53*p06   acaattccaagaacacgctgta-tcttcaaatgaacagcctgagagctgaggacacggcc------------------
IP_V3-53*p07   acaattccaagaacacgctgta-tcttcaaatgaacagcctgagagccgaggacacggccgtgtattactgtgcg---
V_V3-53*01     acaattccaagaacacgctgta-tcttcaaatgaacagcctgagagccgaggacacggccgtgtattactgtgcgaga
V_V3-53*02     acaattccaagaacacgctgta-tcttcaaatgaacagcctgagagccgaggacacggccgtgtattactgtgcgaga
V_V3-53*03     acaattccaagaacacgctgta-tcttcaaatgaacagcctgagagccgaggacacggccgtgtattactgtgctagg
```

IGHV3-64

```
V3-64_01        atgacggagtttgggctgagctgggttttccttgttgctatttttaaaggtgtccagtgtgaggtgcagctggtggagtctgggggagg
V3-64_02        atgatggagtttgggctgagctgggttttccttgttgctatttttaaaggtgtccagtgtgaggtgcagctggtggagtctggggaagg
V3-64_03        atgatggagtttgggctgagctgggttttccttgttgctatttttaaaggtgtccagtgtgaggtgcagctggtggagtctgggggagg
V3-64_04        atgacggagtttgggctgagctgggttttccttgttgctatttttaaaggtgtccagtgtgaggtgcagctggtggagtctgggggagg
V3-64_05        atgacggagtttgggctgagctgggttttccttgttgctatttttaaaggtgtccagtgtgaggtgcagctggtggagtctgggggagg
IGHV3-64*01     atgacggagtttgggctgagctgggttttccttgttgctatttttaaaggtgtccagtgtgaggtgcagctggtggagtctgggggagg
IGHV3-64*02     ---atggagtttgggctgagctgggttttccttgttgctatttttaaaggtgtccagtgtgaggtgcagctggtggagtctggggaagg
IGHV3-64*07     ---atggagtttgggctgagctgggttttccttgttgctatttttaaaggtgtccagtgtgaggtgcagctggtggagtctgggggagg
IGHV3-64*03     ------------------------------------------------------------gaggtgcagctggtggagtctgggggagg
IGHV3-64*04     ------------------------------------------------------------caggtgcagctggtggagtctgggggagg
IGHV3-64*05     ------------------------------------------------------------gaggtgcagctggtggagtctgggggagg
IP_V3-64*p06    ------------------------------------------------------------gaggtgcagctggtggagtctgggggagg
IP_V3-64*p07    ------------------------------------------------------------gaggtgcagctggtggagtctgggggagg
IP_V3-64*p08    ------------------------------------------------------------gaggtgcagctggtggagtctgggggagg
IP_V3-64*p09    ------------------------------------------------------------gaggtgcagctggtggagtctgggggagg
IP_V3-64*p10    ------------------------------------------------------------gaggtgcagctggtggagtctggggaagg
IP_V3-64*p11    ------------------------------------------------------------gaggtgcagctggtggagtctgggggagg
IP_V3-64*p12    ------------------------------------------------------------gaggtgcagctggtggagtctgggggagg
V_V3-64*01      ------------------------------------------------------------gaggtgcagctggtggagtctgggggagg
V_V3-64*02      ------------------------------------------------------------gaggtgcagctggtggagtctggggaagg
IGHV3-64D*06    ---atggagttctggctgagctgggttctccttgttgccattttaaaagatgtccagtgtgaggtgcagctggtggagtctgggggagg

V3-64_01        cttggtccagcctggggggtccctgagactctcctgtgcagcctctggattcaccttcagtagctatgctatgcactgggtccgccaggct
V3-64_02        cttggtccagcctggggggtccctgagactctcctgtgcagcctctggattcaccttcagtagctatgctatgcactgggtccgccaggct
V3-64_03        cttggtccagcctggggggtccctgagactctcctgtgcagcctctggattcaccttcagtagctatgctatgcactgggtccgccaggct
V3-64_04        cttggtccagcctggggggtccctgagactctcctgtgcagcctctggattcaccttcagtagctatgctatgcactgggtccgccaggct
V3-64_05        cttggtccagcctggggggtccctgagactctcctgtgcagcctctggattcaccttcagtagctatgctatgcactgggtccgccaggct
IGHV3-64*01     cttggtccagcctggggggtccctgagactctcctgtgcagcctctggattcaccttcagtagctatgctatgcactgggtccgccaggct
IGHV3-64*02     cttggtccagcctggggggtccctgagactctcctgtgcagcctctggattcaccttcagtagctatgctatgcactgggtccgccaggct
IGHV3-64*07     cttggtccagcctggggggtccctgagactctcctgtgcagcctctggattcaccttcagtagctatgctatgcactgggtccgccaggct
IGHV3-64*03     cttggtccagcctggggggtccctgagactctcctgttcagcctctggattcaccttcagtagctatgctatgcactgggtccgccaggct
IGHV3-64*04     cttggtccagcctggggggtccctgagactctcctgttcagcctctggattcaccttcagtagctatgctatgcactgggtccgccaggct
IGHV3-64*05     cttggtccagcctggggggtccctgagactctcctgttcagcctctggattcaccttcagtagctatgctatgcactgggtccgccaggct
IP_V3-64*p06    cttggtccagcctggggggtccctgagactctcctgttcagcctctggattcaccttcagtagctatgctatgcactgggtccgccaggct
IP_V3-64*p07    cttggtccagcctggggggtccctgagactctcctgttcagcctctggattcaccttcagtagctatgctatgcactgggtccgccaggct
IP_V3-64*p08    cttggtccagcctggggggtccctgagactctcctgtgcagcctctggattcaccttcagtagctatgctatgcactgggtccgccaggct
IP_V3-64*p09    cttggtccagcctggggggtccctgagactctcctgtgcagcctctggattcaccttcagtagctatgctatgcactgggtccgccaggct
IP_V3-64*p10    cttggtccagcctggggggtccctgagactctcctgtgcagcctctggattcaccttcagtagctatgctatgcactgggtccgccaggct
IP_V3-64*p11    cttggtccagcctggggggtccctgagactctcctgttcagcctctggattcaccttcagtagctatgctatgcactgggtccgccaggct
IP_V3-64*p12    cttggtccagcctggggggtccctgagactctcctgttcagcctctggattcaccttcagtagctatgctatgcactgggtccgccaggct
V_V3-64*01      cttggtccagcctggggggtccctgagactctcctgtgcagcctctggattcaccttcagtagctatgctatgcactgggtccgccaggct
V_V3-64*02      cttggtccagcctggggggtccctgagactctcctgtgcagcctctggattcaccttcagtagctatgctatgcactgggtccgccaggct
IGHV3-64D*06    cttggtccagcctggggggtccctgagactctcctgttcagcctctggattcaccttcagtagctatgctatgcactgggtccgccaggct
```

```
V3-64_01         ccagggaagggactggaatatgtttcagctattagtagtaatgggggtagcacatattatgcaaactctgtgaagggcagattcaccat
V3-64_02         ccagggaagggactggaatatgtttcagctattagtagtaatgggggtagcacatattatgcagactctgtgaagggcagattcaccat
V3-64_03         ccagggaagggactggaatatgtttcagctattagtagtaatgggggtagcacatattatgcagactctgtgaagggcagattcaccat
V3-64_04         ccagggaagggactggaatatgtttcagctattagtagtaatgggggtagcacatattatgcagactctgtgaagggcagattcaccat
V3-64_05         ccagggaagggactggaatatgtttcagctattagtagtaatgggggtagcacatattatgcaaactctgtgaagggcagattcaccat
IGHV3-64*01      ccagggaagggactggaatatgtttcagctattagtagtaatgggggtagcacatattatgcaaactctgtgaagggcagattcaccat
IGHV3-64*02      ccagggaagggactggaatatgtttcagctattagtagtaatgggggtagcacatattatgcagactctgtgaagggcagattcaccat
IGHV3-64*07      ccagggaagggactggaatatgtttcagctattagtagtaatgggggtagcacatattatgcagactctgtgaagggcagattcaccat
IGHV3-64*03      ccagggaagggactggaatatgtttcagctattagtagtaatgggggtagcacatactacgcagactcagtgaagggcagattcaccat
IGHV3-64*04      ccagggaagggactggaatatgtttcagctattagtagtaatgggggtagcacatactacgcagactcagtgaagggcagattcaccat
IGHV3-64*05      ccagggaagggactggaatatgtttcagctattagtagtaatgggggtagcacatactacgcagactcagtgaagggcagattcaccat
IP_V3-64*p06     ccagggaagggactggaatatgtttcagctattagtagtaatgggggtagcacatactacgcagactcagtgaagggcagattcaccat
IP_V3-64*p07     ccagggaagggactggaatatgtttcagctattagtagtaatgggggtagcacatactacgcagactccgtgaagggcagattcaccat
IP_V3-64*p08     ccagggaagggactggaatatgtttcagctattagtagtaatgggggtagcacatattatgcaaactctgtgaagggcagattcaccat
IP_V3-64*p09     ccagggaagggactggaatatgtttcagctattagtagtaatgggggtagcacatattatgcagactctgtgaagggcagattcaccat
IP_V3-64*p10     ccagggaagggactggaatatgtttcagctattagtagtaatgggggtagcacatattatgcagactctgtgaagggcagattcaccat
IP_V3-64*p11     ccagggaagggactggaatatgtttcagctattagtagtaatgggggtagcacatactacgcagactcagtgaagggcagattcaccat
IP_V3-64*p12     ccagggaagggactggaatatgtttcagctattagtagtaatgggggtagcacatactacgcagactccgtgaagggcagattcaccat
V_V3-64*01       ccagggaagggactggaatatgtttcagctattagtagtaatgggggtagcacatattatgcaaactctgtgaagggcagattcaccat
V_V3-64*02       ccagggaagggactggaatatgtttcagctattagtagtaatgggggtagcacatattatgcagactctgtgaagggcagattcaccat
IGHV3-64D*06     ccagggaagggactggaatatgtttcagctattagtagtaatgggggtagcacatactacgcagactccgtgaagggcagattcaccat

V3-64_01         ctccagagacaattccaagaacacgctgtatcttcaaatgggcagcctgagagctgaggacatggctgtgtattactgtgcgaga
V3-64_02         ctccagagacaattccaagaacacgctgtatcttcaaatgggcagcctgagagctgaggacatggctgtgtattactgtgcgaga
V3-64_03         ctccagagacaattccaagaacacgctgtatcttcaaatgggcagcctgagagctgaggacatggctgtgtattactgtgcgaga
V3-64_04         ctccagagacaattccaagaacacgctgtatcttcaaatgggcagcctgagagctgaggacatggctgtgtattactgtgcgaga
V3-64_05         ctccagagacaattccaagaacacgctgtatcttcaaatgggcagcctgagagctgaggacacggctgtgtattactgtgcgaga
IGHV3-64*01      ctccagagacaattccaagaacacgctgtatcttcaaatgggcagcctgagagctgaggacatggctgtgtattactgtgcgaga
IGHV3-64*02      ctccagagacaattccaagaacacgctgtatcttcaaatgggcagcctgagagctgaggacatggctgtgtattactgtgcgaga
IGHV3-64*07      ctccagagacaattccaagaacacgctgtatcttcaaatgggcagcctgagagctgaggacatggctgtgtattactgtgcgaga
IGHV3-64*03      ctccagagacaattccaagaacacgctgtatg ccaaatgagcagtctgagagctgaggacacggctgtgtattactgtgtgaaa
IGHV3-64*04      ctccagagacaattccaagaacacgctgtatctgcaaatgaacagcctgagagctgaggacacggctgtgtattactgtgcgaga
IGHV3-64*05      ctccagagacaattccaagaacacgctgtatg tcaaatgagcagtctgagagctgaggacacggctgtgtattactgtgtgaaa
IP_V3-64*p06     ctccagagacaattccaagaacacgctgtatcttcaaatgagcagtctgagagctgaggacacggctgtgtattactgtgtgaaa
IP_V3-64*p07     ctccagagacaattccaagaacacgctgtatcttcaaatgagcagtctgagagctgaggacacggctgtgtattactgtgtgaaa
IP_V3-64*p08     ctccagagacaattccaagaacacgctgtatcttcaaatgggcagcctgagagctgaggacacggctgtgtattactgtatgaga
IP_V3-64*p09     ctccagagacaattccaagaacacgctgtatcttcaaatgggcagcctgagagctgaggacatggctgtgtattactgtgcgaga
IP_V3-64*p10     ctccagagacaattccaagaacacgctgtatcttcaaatgggcagcctgagagctgaggacatggctgtgtactagtatacgaga
IP_V3-64*p11     ctccagagacaattccaagaacacgctgtatcttcaaatgagcagtctgagagctgaggacacggctgtgtattactgtgtgaga
IP_V3-64*p12     ctccagagacaattccaagaacacgctgtatcttcaaatgagcagtctgagagctgaggacacggctgtgtattactgtgtgaga
V_V3-64*01       ctccagagacaattccaagaacacgctgtatcttcaaatgggcagcctgagagctgaggacatggctgtgtattactgtgcgaga
V_V3-64*02       ctccagagacaattccaagaacacgctgtatcttcaaatgggcagcctgagagctgaggacatggctgtgtattactgtgcgaga
IGHV3-64D*06     ctccagagacaattccaagaacacgctgtatcttcaaatgagcagtctgagagctgaggacacggctgtgtattactgtgtgaaa
```

IGHV3-66

```
V3-66_01        atggagtttgggctgagctgggttttccttgttgctattttaaaaggtgtccagtgtgaggtgcagctggtggagtctggaggaggcttg
V3-66_02        atggagtttgggctgagctgggttttccttgttgctattttaaaaggtgtccagtgtgaggtgcagctggtggagtctggggggaggcttg
V3-66_03        atggagtttgggctgagctgggttttccttgttgctattttaaaaggtgtccagtgtgaggtgcagctggtggagtctggggggaggcttg
V3-66_04        atggagtttgggctgagctgggttttccttgttgctattttaaaaggtgtccagtgtgaggtgcagctggtggagtctggaggaggcttg
V3-66_05        atggagtttgggctgagctgggttttccttgttgctattttaaaaggtgtccagtgtgaggtgcagctggtggagtctgcgggaggcttg
V3-66_06        atggagtttgggctgagctgggttttccttgttgctattttaaaaggtgtccagtgtgaggtgcagctggtggagtctgcgggaggcttg
V3-66_07        atggagtttgggctgagctgggttttccttgttgctattttaaaaggtgtccagtgtgaggtgcagctggtggagtctgcaggaggcttg
V3-66_08        atggagtttgggctgagctgggttttccttgttgctattttaaaaggtgtccagtgtgaggtgcagctggtggagtctggaggaggcttg
V3-66_09        atggagtttgggctgagctgggttttccttgttgctattttaaaaggtgtccagtgtgaggtgcagctggtggagtctgcgggaggcttg
V3-66_10        atggagtttgggctgagctgggttttccttgttgctattttaaaaggtgtccagtgtgaggtgcagctggtggagtctgcaggaggcttg
IGHV3-66*01     atggagtttgggctgagctgggttttccttgttgctattttaaaaggtgtccagtgtgaggtgcagctggtggagtctggggggaggcttg
IGHV3-66*03     atggagtttgggctgagctgggttttccttgttgctattttaaaaggtgtccagtgtgaggtgcagctggtggagtctggaggaggcttg
IGHV3-66*04     atggagtttgggctgagctgggttttccttgttgctattttaaaaggtgtccagtgtgaggtgcagctggtggagtctggggggaggcttg
IGHV3-66*02     ---------------------------------------------------------gaggtgcagctggtggagtctggggggaggcttg
IP_V3-66*p05    ---------------------------------------------------------gaggtgcagctggtggagtctggggggaggcttg
IP_V3-66*p06    ---------------------------------------------------------gaggtgcagctggtggagtctggggggaggcttg
IP_V3-66*p07    ---------------------------------------------------------gaggtgcagctggtggagtctggaggaggcttg
IP_V3-66*p08    ---------------------------------------------------------gaggtgcagctggtggagactggaggaggcttg
IP_V3-66*p09    ---------------------------------------------------------gaggtgcagctggtggagtctggggggaggcttg
IP_V3-66*p10    ---------------------------------------------------------gaggtgcagctggtggagtctggggggaggcttg
V_V3-66*04      ---------------------------------------------------------gaggtgcagctggtggagtctggggggaggcttg
V_V3-66*03      ---------------------------------------------------------gaggtgcagctggtggagtctggaggaggcttg

V3-66_01        atccagcctggggggtccctgagactctcctgtgcagcctctgggttcaccgtcagtagcaactacatgagctgggtccgccaggctcca
V3-66_02        gtccagcctggggggtccctgagactctcctgtgcagcctctgggttcaccgtcagtagcaactacatgagctgggtccgccaggctcca
V3-66_03        gtccagcctggggggtccctgagactctcctgtgcagcctctgggttcaccgtcagtagcaactacatgagctgggtccgccaggctcca
V3-66_04        atccagcctggggggtccctgagactctcctgtgcagcctctgggttcaccgtcagtagcaactacatgagctgggtccgccaggctcca
V3-66_05        gtccagcctggggggtccctgagactctcctgtgcagcctctgggttcaccgtcagtagcaactacatgagctgggtccgccaggctcca
V3-66_06        gtccagcctggggggtccctgagactctcctgtgcagcctctgggttcaccgtcagtagcaactacatgagctgggtccgccaggctcca
V3-66_07        atccagcctggggggtccctgagactctcctgtgcagcctctgggttcaccgtcagtagcaactacatgagctgggtccgccaggctcca
V3-66_08        gtccagcctggggggtccctgagactctcctgtgcagcctctgggttcaccgtcagtagcaactacatgagctgggtccgccaggctcca
V3-66_09        gtccagcctggggggtccctgagactctcctgtgcagcctctgggttcaccgtcagtagcaactacatgagctgggtccgccaggctcca
V3-66_10        atccagcctggggggtccctgagactctcctgtgcagcctctgggttcaccgtcagtagcaactacatgagctgggtccgccaggctcca
IGHV3-66*01     gtccagcctggggggtccctgagactctcctgtgcagcctctggattcaccgtcagtagcaactacatgagctgggtccgccaggctcca
IGHV3-66*03     atccagcctggggggtccctgagactctcctgtgcagcctctgggttcaccgtcagtagcaactacatgagctgggtccgccaggctcca
IGHV3-66*04     gtccagcctggggggtccctgagactctcctgtgcagcctctggattcaccgtcagtagcaactacatgagctgggtccgccaggctcca
IGHV3-66*02     gtccagcctggggggtccctgagactctcctgtgcagcctctggattcaccgtcagtagcaactacatgagctgggtccgccaggctcca
IP_V3-66*p05    gtccagcctggggggtccctgagactctcctgtgcagcctctggattcaccgtcagtagcaactacatgagccgggtccgccaggctcca
IP_V3-66*p06    gtccagcctggggggtccctgagactctcctgtgcagcctctggattcaccgtcagtagcaactacatgagctgggtccgccaggctcca
IP_V3-66*p07    atccagcctggggggtccctgagactctcctgtgcagcctctgggttcaccgtcagtagcaactacatgagctgggtccgccaggctcca
IP_V3-66*p08    atccagcctggggggtccctgagactctcctgtgcagcctctgggttcaccgtcagtagcaactacatgagctgggtccgccaggctcca
IP_V3-66*p09    gtccagcctggggggtccctgagactctcctgtgcagcctctgggttcaccgtcagtagcaactacatgagctgggtccgccaggctcca
IP_V3-66*p10    gtccagcctggggggtccctgagactctcctgtgcagcctctggattcaccgtcagtagcaactacatgagctgggtccgccaggctcca
V_V3-66*04      gtccagcctggggggtccctgagactctcctgtgcagcctctggattcaccgtcagtagcaactacatgagctgggtccgccaggctcca
V_V3-66*03      atccagcctggggggtccctgagactctcctgtgcagcctctgggttcaccgtcagtagcaactacatgagctgggtccgccaggctcca
```

```
V3-66_01        gggaaggggctggagtgggtctcagttatttatagctgtggtagcacatactacgcagactccgtgaagggccgattcaccatctccagag
V3-66_02        gggaaggggctggagtgggtctcagttatttatagctgtggtagcacatactacgcagactccgtgaagggccgattcaccatctccagag
V3-66_03        gggaaggggctggagtgggtctcagttatttatagcggtggtagcacatactacgcagactccgtgaagggccgattcaccatctccagag
V3-66_04        gggaaggggctggagtgggtctcagttatttatagcggtggtagcacatactacgcagactccgtgaagggccgattcaccatctccagag
V3-66_05        gggaaggggctggagtgggtctcagttatttatagcggtggtagcacatactacgcagactccgtgaagggccgattcaccatctccagag
V3-66_06        gggaaggggctggagtgggtctcagttatttatagctgtggtagcacatactacgcagactccgtgaagggccgattcaccatctccagag
V3-66_07        gggaaggggctggagtgggtctcagttatttatagctgtggtagcacatactacgcagactccgtgaagggccgattcaccatctccagag
V3-66_08        gggaaggggctggagtgggtctcagttatttatagcggtggtagcacatactacgcagactccgtgaagggccgattcaccatctccagag
V3-66_09        gggaaggggctggagtgggtctcagttatttatagctgtggtagcacatactacgcagactccgtgaagggccgattcaccatctccagag
V3-66_10        gggaaggggctggagtgggtctcagttatttatagcggtggtagcacatactacgcagactccgtgaagggccgattcaccatctccagag
IGHV3-66*01     gggaaggggctggagtgggtctcagttatttatagcggtggtagcacatactacgcagactccgtgaagggcagattcaccatctccagag
IGHV3-66*03     gggaaggggctggagtgggtctcagttatttatagctgtggtagcacatactacgcagactccgtgaagggccgattcaccatctccagag
IGHV3-66*04     gggaaggggctggagtgggtctcagttatttatagcggtggtagcacatactacgcagactccgtgaagggcagattcaccatctccagag
IGHV3-66*02     gggaaggggctggagtgggtctcagttatttatagcggtggtagcacatactacgcagactccgtgaagggccgattcaccatctccagag
IP_V3-66*p05    gggaaggggctggagtgggtctcagttatttatagcggtggtagcacatactacgcagactccgtgaagggccgattcaccatctccagag
IP_V3-66*p06    gggaaggggctggagtgggtctcagttatttatagctgtggtagcacatactacgcagactccgtgaagggccgattcaccatctccagag
IP_V3-66*p07    gggaaggggctggagtgggtctcagttatttatagctgtggtagcacatactacgcagactccgtgaagggccgattcaccatctccagag
IP_V3-66*p08    gggaaggggctggagtgggtctcagttatttatagctgtggtagcacatactacgcagactccgtgaagggccgattcaccatctccagag
IP_V3-66*p09    gggaaggggctggagtgggtctcagttatttatagcggtggtagcacatactacgcagactccgtgaagggcagattcaccatctccagag
IP_V3-66*p10    gggaaggggctggagtgggtctcagttatttatagcggtggtagcgcatactacgcagactccgtgaagggcagattcaccatctccagag
V_V3-66*04      gggaaggggctggagtgggtctcagttatttatagcggtggtagcacatactacgcagactccgtgaagggcagattcaccatctccagag
V_V3-66*03      gggaaggggctggagtgggtctcagttatttatagctgtggtagcacatactacgcagactccgtgaagggccgattcaccatctccagag

V3-66_01        acaattccaagaacacgctgtatcttcaaatgaacagcctgagagctgaggacacggctgtgtattactgtgcgaga
V3-66_02        acaattccaagaacacgctgtatcttcaaatgaacagcctgagagctgaggacacggctgtgtattactgtgcgaga
V3-66_03        acaattccaagaacacgctgtatcttcaaatgaacagcctgagagctgaggacacggctgtgtattactgtgcgaga
V3-66_04        acaattccaagaacacgctgtatcttcaaatgaacagcctgagagctgaggacacggctgtgtattactgtgcgaga
V3-66_05        acaattccaagaacacgctgtatcttcaaatgaacagcctgagagctgaggacacggctgtgtattactgtgcgaga
V3-66_06        acaattccaagaacacgctgtatcttcaaatgaacagcctgagagctgaggacacggctgtgtattactgtgcgaga
V3-66_07        acaattccaagaacacgctgtatcttcaaatgaacagcctgagagctgaggacacggctgtgtattactgtgcgaga
V3-66_08        acaattccaagaacacgctgtatcttcaaatgaacagcctgagagctgaggacacggctgtgtattactgtgcgaga
V3-66_09        acaattccaagaacacgctgtatcttcaaatgaacagcctgagagctgaggacacggctgtgtattactgtgccaga
V3-66_10        acaattccaagaacacgctgtatcttcaaatgaacagcctgagagctgaggacacggctgtgtattactgtgcgaga
IGHV3-66*01     acaattccaagaacacgctgtatcttcaaatgaacagcctgagagccgaggacacggctgtgtattactgtgcgaga
IGHV3-66*03     acaattccaagaacacgctgtatcttcaaatgaacagcctgagagctgaggacacggctgtgtattactgtgcgaga
IGHV3-66*04     acaattccaagaacacgctgtatcttcaaatgaacagcctgagagccgaggacacggctgtgtattactgtgcgaga
IGHV3-66*02     acaattccaagaacacgctgtatcttcaaatgaacagcctgagagctgaggacacggctgtgtattactgtgcgaga
IP_V3-66*p05    acaattccaagaacacgctgtatcttcaaatgaacagcctgagagctgaggacacggct------------------
IP_V3-66*p06    acaattccaagaacacgctgtatcttcaaatgaacagcctgagagctgaggacacggct------------------
IP_V3-66*p07    acaattccaagaacacgctgtatcttcaaataaacagcctgagagctgaggacacggct------------------
IP_V3-66*p08    acaattccaagaacacgctgtatcttcaaatgaacagcctgagagctgaggacacggcc------------------
IP_V3-66*p09    acaattccaagaacacgctgtatcttcaaatgaacagcctgagagccgaggacacggct------------------
IP_V3-66*p10    acaattccaagaacacgctgtatcttcaaatgaacagcctgagagccgaggacacggct------------------
V_V3-66*04      acaattccaagaacacgctgtatcttcaaatgaacagcctgagagccgaggacacggctgtgtattactgtgcgaga
V_V3-66*03      acaattccaagaacacgctgtatcttcaaatgaacagcctgagagctgaggacacggctgtgtattactgtgcgaga
```

IGHV3-72

```
V3-72_01        atggagtttgggctgagctgggttttccttgttgttattttacaaggtgtccagtgtgaggtgcagctggtggagtctgggggaggcttgg
V3-72_02        atggagtttgggctgagctgggttttccttgttgttattttacaaggtgtccagtgtgaggtgcagctggtggagtctgggggaggcttgg
V3-72_03        atggagtttgggctgagctgggttttccttgttgttattttacaaggtgtccagtgtgaggtgcagctggtggagtctgggggaggcttgg
IGHV3-72*01     atggagtttgggctgagctgggttttccttgttgttattttacaaggtgtccagtgtgaggtgcagctggtggagtctgggggaggcttgg
IGHV3-72*02     ------------------------------------------------------------------------------------------
IP_V3-72*p03    ---------------------------------------------------------gaggtgcagctggtggagtctgggggaggcttgg
V_V3-72*01      ---------------------------------------------------------gaggtgcagctggtggagtctgggggaggcttgg

V3-72_01        tccagcctggagggtccctgagactctcctgtgcagcctctggattcaccttcagtgaccactacatggactgggtccgccaggctcca
V3-72_02        tccagcctggagggtccctgagactctcctgtgcagcctctggattcaccttcagtgaccactacatggactgggtccgccaggctcca
V3-72_03        tccagcctggagggtctctgagactctcctgtgcagcctctggattcaccttcagtgaccactacatggactgggtccgccaggctcca
IGHV3-72*01     tccagcctggagggtccctgagactctcctgtgcagcctctggattcaccttcagtgaccactacatggactgggtccgccaggctcca
IGHV3-72*02     -----------------------------------------------accttcagtgaccactacatggactgggtccgccaggctcca
IP_V3-72*p03    tccagcctggagggtctctgagactctcctgtgcagcctctggattcaccttcagtgaccactacatggactgggtccgccaggctcca
V_V3-72*01      tccagcctggagggtccctgagactctcctgtgcagcctctggattcaccttcagtgaccactacatggactgggtccgccaggctcca

V3-72_01        gggaaggggctggagtgggttggccgtactagaaacaaagctaacagttacaccacagaatacgccgcgtctgtgaaaggcagattcaccatc
V3-72_02        gggaaggggctggagtgggttggccgtactagaaacaaagctaacagctacaccacagaatacgccgcgtctgtgaaaggcagattcaccatc
V3-72_03        gggaaggggctggagtgggttggccgtactagaaacaaagctaacagttacaccacagaatacgccgcgtctgtgaaaggcagattcaccatc
IGHV3-72*01     gggaaggggctggagtgggttggccgtactagaaacaaagctaacagttacaccacagaatacgccgcgtctgtgaaaggcagattcaccatc
IGHV3-72*02     gggaaggggctggagtgggttggccgtactagaaacaaagctaacagctacaccacagaatacgccgcgtctgtgaaaggcagattcaccatc
IP_V3-72*p03    gggaaggggctggagtgggttggccgtactagaaacaaagctaacagttacaccacagaatacgccgcgtctgtgaaaggcagattcaccatc
V_V3-72*01      gggaaggggctggagtgggttggccgtactagaaacaaagctaacagttacaccacagaatacgccgcgtctgtgaaaggcagattcaccatc

V3-72_01        tcaagagatgattcaaagaactcactgtatctgcaaatgaacagcctgaaaaccgaggacacggccgtgtattactgtgctaga
V3-72_02        tcaagagatgattcaaagaactcactgtatctgcaaatgaacagcctgaaaaccgaggacacggccgtgtattactgtgctaga
V3-72_03        tcaagagatgattcaaagaactcactgtatctgcaaatgaacagcctgaaaaccgaggacacggccgtgtattactgtgctaga
IGHV3-72*01     tcaagagatgattcaaagaactcactgtatctgcaaatgaacagcctgaaaaccgaggacacggccgtgtattactgtgctaga
IGHV3-72*02     tcaagagatgattcaaagaactcactgtat------------------------------------------------------
IP_V3-72*p03    tcaagagatgattcaaagaactcactgtatctgcaaatgaacagcctgaaaaccgaggacacggccgtgtattactgtgctaga
V_V3-72*01      tcaagagatgattcaaagaactcactgtatctgcaaatgaacagcctgaaaaccgaggacacggccgtgtattactgtgctaga
```

IGHV3-73

```
V3-73_01      atggagtttgggctgagctgggttttccttgttgctattttaaaaggtgtccagtgtgaggtgcagctggtggagtctgggggaggcttggt
V3-73_02      atggagtttgggctgagctgggttttccttgttgctattttaaaaggtgtccagtgtgaggtgcagctggtggagtccgggggaggcttggt
V3-73_03      atggagtttgggctgagctgcgttttccttgttgctattttaaaaggtgtccagtgtgaggtgcagctggtggagtccgggggaggcttggt
V3-73_04      atggagtttgggctgagctgggttttccttgttgctattttaaaaggtgtccagtgtgaggtgcagctggtggagtctgggggaggcttggt
V3-73_05      atggagtttgggctgagctgggttttccttgttgctattttaaaaggtgtccagtgtgaggtgcagctggtggagtccgggggaggcttggt
V3-73_06      atggagtttgggctgagctgcgttttccttgttgctattttaaaaggtgtccagtgtgaggtgcagctggtggagtccgggggaggcttggt
V3-73_07      atggagtttgggctgagctgcgttttccttgttgctattttaaaaggtgtccagtgtgaggtgcagctggtggagtctgggggaggcttggt
IGHV3-73*01   atggagtttgggctgagctgggttttccttgttgctattttaaaaggtgtccagtgtgaggtgcagctggtggagtctgggggaggcttggt
IGHV3-73*02   atggagtttgggctgagctgggttttccttgttgctattttaaaaggtgtccagtgtgaggtgcagctggtggagtccgggggaggcttggt
V_V3-73*01    ---------------------------------------------------------gaggtgcagctggtggagtctgggggaggcttggt
V_V3-73*02    ---------------------------------------------------------gaggtgcagctggtggagtccgggggaggcttggt

V3-73_01      ccagcctggggggtccctgaaaactctcctgtgcagcctctgggttcaccttcagtggctctgctatgcactgggtccgccaggcttcc
V3-73_02      ccagcctggggggtccctgaaaactctcctgtgcagcctctgggttcaccttcagtggctctgctatgcactgggtccgccaggcttcc
V3-73_03      ccagcctggggggtccctgaaaactctcctgtgcagcctctgggttcaccttcagtggctctgctatgcactgggtccgccaggcttcc
V3-73_04      ccagcctggggggtccctgagactctcctgtgcagcctctgggttcaccttcagtggctctgctatgcactgggtccgccaggcttcc
V3-73_05      ccagcctggggggtccctgagactctcctgtgcagcctctgggttcaccttcagtggctctgctatgcactgggtccgccaggcttcc
V3-73_06      ccagcctggggggtccctgagactctcctgtgcagcctctgggttcaccttcagtggctctgctatgcactgggtccgccaggcttcc
V3-73_07      ccagcctggggggtccctgaaaactctcctgtgcagcctctgggttcaccttcagtggctctgctatgcactgggtccgccaggcttcc
IGHV3-73*01   ccagcctggggggtccctgaaaactctcctgtgcagcctctgggttcaccttcagtggctctgctatgcactgggtccgccaggcttcc
IGHV3-73*02   ccagcctggggggtccctgaaaactctcctgtgcagcctctgggttcaccttcagtggctctgctatgcactgggtccgccaggcttcc
V_V3-73*01    ccagcctggggggtccctgaaaactctcctgtgcagcctctgggttcaccttcagtggctctgctatgcactgggtccgccaggcttcc
V_V3-73*02    ccagcctggggggtccctgaaaactctcctgtgcagcctctgggttcaccttcagtggctctgctatgcactgggtccgccaggcttcc

V3-73_01      gggaaagggctggagtgggttggccgtattagaagcaaagctaacagttacgcgacagcatatgctgcgtcggtgaaaggcaggttcaccatc
V3-73_02      gggaaagggctggagtgggttggccgtattagaagcaaagctaacagttacgcgacagcatatgctgcgtcggtgaaaggcaggttcaccatc
V3-73_03      gggaaagggctggagtgggttggccgtattagaagcaaagctaacagttacgcgacagcatatgctgcgtcggtgaaaggcaggttcaccatc
V3-73_04      gggaaagggctggagtgggttggccgtattagaagcaaagctaacagttacgcgacagcatatgctgcgtcggtgaaaggcaggttcaccatc
V3-73_05      gggaaagggctggagtgggttggccgtattagaagcaaagctaacagttacgcgacagcatatgctgcgtcggtgaaaggcaggttcaccatc
V3-73_06      gggaaagggctggagtgggttggccgtattagaagcaaagctaacagttacgcgacagcatatgctgcgtcggtgaaaggcaggttcaccatc
V3-73_07      gggaaagggctggagtgggttggccgtattagaagcaaagctaacagttacgcgacagcatatgctgcgtcggtgaaaggcaggttcaccatc
IGHV3-73*01   gggaaagggctggagtgggttggccgtattagaagcaaagctaacagttacgcgacagcatatgctgcgtcggtgaaaggcaggttcaccatc
IGHV3-73*02   gggaaagggctggagtgggttggccgtattagaagcaaagctaacagttacgcgacagcatatgctgcgtcggtgaaaggcaggttcaccatc
V_V3-73*01    gggaaagggctggagtgggttggccgtattagaagcaaagctaacagttacgcgacagcatatgctgcgtcggtgaaaggcaggttcaccatc
V_V3-73*02    gggaaagggctggagtgggttggccgtattagaagcaaagctaacagttacgcgacagcatatgctgcgtcggtgaaaggcaggttcaccatc
```

```
V3-73_01        tccagagatgattcaaagaacacggcgtatctgcaaatgaacagcctgaaaaccgaggacacggccgtgtattactgtactaga
V3-73_02        tccagagatgattcaaagaacacggcgtatctgcaaatgaacagcctgaaaaccgaggacacggccgtgtattactgtactaga
V3-73_03        tccagagatgattcaaagaacacggcgtatctgcaaatgaacagcctgaaaaccgaggacacggccgtgtattactgtactaga
V3-73_04        tccagagatgattcaaagaacacggcgtatctgcaaatgaacagcctgaaaaccgaggacacggccgtgtattactgtactaga
V3-73_05        tccagagatgattcaaagaacacggcgtatctgcaaatgaacagcctgaaaaccgaggacacggccgtgtattactgtactaga
V3-73_06        tccagagatgattcaaagaacacggcgtatctgcaaatgaacagcctgaaaaccgaggacacggccgtgtattactgtactaga
V3-73_07        tccagagatgattcaaagaacacggcgtatctgcaaatgaacagcctgaaaaccgaggacacggccgtgtattactgtactaga
IGHV3-73*01     tccagagatgattcaaagaacacggcgtatctgcaaatgaacagcctgaaaaccgaggacacggccgtgtattactgtactaga
IGHV3-73*02     tccagagatgattcaaagaacacggcgtatctgcaaatgaacagcctgaaaaccgaggacacggccgtgtattactgtactaga
V_V3-73*01      tccagagatgattcaaagaacacggcgtatctgcaaatgaacagcctgaaaaccgaggacacggccgtgtattactgtactaga
V_V3-73*02      tccagagatgattcaaagaacacggcgtatctgcaaatgaacagcctgaaaaccgaggacacggccgtgtattactgtactaga
```

IGHV3-74

```
V3-74_01       atggagtttgggctgagctgggttttccttgttgctattttaaaaggtgtccagtgtgaggtgcagctggtggagtccgggggaggcttagtt
V3-74_02       atggagtttgggctgagctgggttttccttgttgctattttaaaaggtgtccagtgtgaggtgcagctggtggagtctgggggaggcttagtt
V3-74_03       atggagtttgggctgagctgggttttccttgttgctattttaaaaggtgtccagtgtgaggtgcagctggtggagtccgggggaggcttagtt
IGHV3-74*01    atggagtttgggctgagctgggttttccttgttgctattttaaaaggtgtccagtgtgaggtgcagctggtggagtccgggggaggcttagtt
IGHV3-74*03    atggagtttgggctgagctgggttttccttgttgctattttaaaaggtgtccagtgtgaggtgcagctggtggagtccgggggaggcttagtt
IGHV3-74*02    ---------------------------------------------------------gaggtgcagctggtggagtctgggggaggcttagtt
IP_V3-74*p04   ---------------------------------------------------------------------------------------------
IP_V3-74*p05   ---------------------------------------------------------gaggtgcagctggtggagtccgggggaggcttagtt
V_V3-74*01     ---------------------------------------------------------gaggtgcagctggtggagtccgggggaggcttagtt
V_V3-74*03     ---------------------------------------------------------gaggtgcagctggtggagtccgggggaggcttagtt

V3-74_01       cagcctggggggtccctgagactctcctgtgcagcctctggattcaccttcagtagctactggatgcactgggtccgccaagctcca
V3-74_02       cagcctggggggtccctgagactctcctgtgcagcctctggattcaccttcagtagctactggatgcactgggtccgccaagctcca
V3-74_03       cagcctggggggtccctgagactctcctgtgcagcctctggattcaccttcagtagctactggatgcactgggtccgccaagctcca
IGHV3-74*01    cagcctggggggtccctgagactctcctgtgcagcctctggattcaccttcagtagctactggatgcactgggtccgccaagctcca
IGHV3-74*03    cagcctggggggtccctgagactctcctgtgcagcctctggattcaccttcagtagctactggatgcactgggtccgccaagctcca
IGHV3-74*02    cagcctggggggtccctgagactctcctgtgcagcctctggattcaccttcagtagctactggatgcactgggtccgccaagctcca
IP_V3-74*p04   ---------------------------------------------------------------------------------------
IP_V3-74*p05   cagcctggggggtccctgagactctcctgtgcagcctctggattcaccttcagtagctactggatgcactgggtccgccaagctcca
V_V3-74*01     cagcctggggggtccctgagactctcctgtgcagcctctggattcaccttcagtagctactggatgcactgggtccgccaagctcca
V_V3-74*03     cagcctggggggtccctgagactctcctgtgcagcctctggattcaccttcagtagctactggatgcactgggtccgccaagctcca

V3-74_01       gggaaggggctggtgtgggtctcacgtattaatagtgatgggagtagcacaagctacgcggactccgtgaagggccgattcaccatctcc
V3-74_02       gggaaggggctggtgtgggtctcacgtattaatagtgatgggagtagcacaagctacgcggactccgtgaagggccgattcaccatctcc
V3-74_03       gggaaggggctggtgtgggtctcacgtattaatactgatgggagtagcacaagctacgcggactccgtgaagggccgattcaccatctcc
IGHV3-74*01    gggaaggggctggtgtgggtctcacgtattaatagtgatgggagtagcacaagctacgcggactccgtgaagggccgattcaccatctcc
IGHV3-74*03    gggaaggggctggtgtgggtctcacgtattaatagtgatgggagtagcacaacgtacgcggactccgtgaagggccgattcaccatctcc
IGHV3-74*02    gggaaggggctggtgtgggtctcacgtattaatagtgatgggagtagcacaagctacgcggactccgtgaagggccgattcaccatctcc
IP_V3-74*p04   ---aaggggctggtgtgggtctcacgtattaatactgatgggagtagcacaagctacgcggactccgtgaagggccgattcaccatctcc
IP_V3-74*p05   gggaaggggctggtgtgggtctcacgtattaatagtgatgggagtagcacaagctacgcggactccgtgaagggccgattcaccatctcc
V_V3-74*01     gggaaggggctggtgtgggtctcacgtattaatagtgatgggagtagcacaagctacgcggactccgtgaagggccgattcaccatctcc
V_V3-74*03     gggaaggggctggtgtgggtctcacgtattaatagtgatgggagtagcacaacgtacgcggactccgtgaagggccgattcaccatctcc
```

```
V3-74_01        agagacaacgccaagaacacgctgtatctgcaaatgaacagtctgagagccgaggacacggctgtgtattactgtgcaaga--
V3-74_02        agagacaacgccaagaacacgctgtatctgcaaatgaacagtctgagagccgaggacacggctgtgtattactgtgcaaga--
V3-74_03        agagacaacgccaagaacacgctgtatctgcaaatgaacagtctgagagccgaggacacggctgtgtattactgtgcaaga--
IGHV3-74*01     agagacaacgccaagaacacgctgtatctgcaaatgaacagtctgagagccgaggacacggctgtgtattactgtgcaagaga
IGHV3-74*03     agagacaacgccaagaacacgctgtatctgcaaatgaacagtctgagagccgaggacacggctgtgtattactgtgcaagaga
IGHV3-74*02     agagacaacgccaagaacacgctgtatctgcaaatgaacagtctgagagccgaggacacggctgtgtattactgtgcaaga--
IP_V3-74*p04    agagacaacgccaagaacacgctgtatctgcaaatgaacagtctgagagccgaggacacggctgtgtattactgtgcaagaga
IP_V3-74*p05    agagacaacgccaagaacacgctgtatctgcaaatgaacagtctgagagccgaggacacagct--------------------
V_V3-74*01      agagacaacgccaagaacacgctgtatctgcaaatgaacagtctgagagccgaggacacggctgtgtattactgtgcaaga--
V_V3-74*03      agagacaacgccaagaacacgctgtatctgcaaatgaacagtctgagagccgaggacacggctgtgtattactgtgcaaga--
```

IGHV4-4

```
V4-4_01        atgaaacacctgtggttcttcctcctgctggtggcagctcccagatgggtcctgtcccaggtgcagctgcaggagtcgggcccag
V4-4_02        atgaaacacctgtggttcttcctcctcctggtggcagctcccagatgggtcctgtcccaggtgcagctgcaggagtcgggcccag
V4-4_03        atgaaacacctgtggttcttcctcctcctggtggcagctcccagatgggtcctgtctcaggtgcagctgcaggagtcgggcccag
V4-4_04        atgaaacacctgtggttcttcctcctcctggtggcagctcccagatgggtcctgtctcaggtgcagctgcaggagtcgggcccag
V4-4_05        atgaaacacctgtggttcttcctcctcctggtggcagctcccagatgggtcctgtcccaggtgcagctgcaggagtcgggcccag
V4-4_08        atgaaacacctgtggttcttcctcctcctggtggcagctcccagatgggtcctgtctcaggtgcagctgcaggagtcgggcccag
V4-4_10        atgaaacacctgtggttcttcctcctcctggtggcagctcccagatgggtcctgtctcaggtgcagctgcaggagtcgggcccag
V4-4_13        atgaaacacctgtggttcttcctcctcctggtggcagctcccagatgggtcctgtctcaggtgcagctgcaggagtcgggcccag
V4-4_14        atgaaacacctgtggttcttcctcctcctggtggcagctcccagatgggtcctgtctcaggtgcagctgcaggagtcgggcccag
V4-4_15        atgaaacacctgtggttcttcctcctcctggtggcagctcccagatgggtcctgtcccaggtgcagctgcaggagtcgggcccag
V4-4_16        atgaaacacctgtggttcttcctcctgctggtggcagctcccagatgggtcctgtcccaggtgcagctgcaggagtcgggcccag
V4-4_17        atgaaacacctgtggttcttcctcctcctggtggcagctcccagatgggtcctgtctcaggtgcagctgcaggagtcgggcccag
V4-4_18        atgaaacacctgtggttcttcctcctcctggtggcagctcccagatgggtcctgtctcaggtgcagctgcaggagtcgggcccag
V4-4_20        atgaaacacctgtggttcttcctcctcctggtggcagctcccagatgggtcctgtctcaggtgcagctgcaggagtcgggcccag
V4-4_21        atgaaacacctgtggttcttcctcctcctggtggcagctcccagatgggtcctgtctcaggtgcagctgcaggagtcgggcccag
V4-4_22        atgaaacacctgtggttcttcctcctgctggtggcagctcccagatgggtcctgtcccaggtgcagctgcaggagtcgggcccag
V4-4_23        atgaaacacctgtggttcttcctcctcctggtggcagctcccagatgggtcctgtctcaggtgcagctgcaggagtcgggcccag
V4-4_25        atgaaacacctgtggttcttcctcctcctggtggcagctcccagatgggtcctgtcccaggtgcagctgcaggagtcgggcccag
V4-4_26        atgaaacacctgtggttcttcctcctcctggtggcagctcccagatgggtcctgtctcaggtgcagctgcaggagtcgggcccag
V4-4_27        atgaaacacctgtggttcttcctcctcctggtggcagctcccagatgggtcctgtcccaggtgcagctgcaggagtcgggcccag
V4-4_30        atgaagcacctgtggttcttcctcctgctggtggcagctcccagatgggtcctgtctcaggtgcagctgcaggagtcgggcccag
V4-4_31        atgaaacacctgtggttcttcctcctgctggtggcagctcccagatgggtcctgtcccaggtgcagctgcaggagtcgggcccag
V4-4_32        atgaaacacctgtggttcttcctcctcctggtggcagctcccagatgggtcctgtcccaggtgcagctgcaggagtcgggcccag
V4-4_34        atgaaacacctgtggttcttcctcctcctggtggcagctcccagatgggtcctgtctcaggtgcagctgcaggagtcgggcccag
V4-4_35        atgaaacacctgtggttcttcctcctgctggtggcagctcccagatgggtcctgtcccaggtgcagctgcaggagtcgggcccag
V4-4_36        atgaaacacctgtggttcttcctcctcctggtggcagctcccagatgggtcctgtctcaggtgcagctgcaggagtcgggcccag
V4-4_40        atgaaacacctgtggttcttcctcctcctggtggcagctcccagatgggtcctgtcccaggtgcagctgcaggagtcgggcccag
V4-4_42        atgaaacacctgtggttcttcctcctcctggtggcagctcccagatgggtcctgtcccaggtgcagctgcaggagtcgggcccag
V4-4_43        atgaaacacctgtggttcttcctcctcctggtggcagctcccagatgggtcctgtcccaggtgcagctgcaggagtcgggcccag
V4-4_44        atgaaacacctgtggttcttcctcctcctggtggcagctcccagatgggtcctgtcccaggtgcagctgcaggagtcgggcccag
IGHV4-4*01     atgaaacacctgtggttcttcctcctcctggtggcagctcccagatgggtcctgtctcaggtgcagctgcaggagtcgggcccag
IGHV4-4*02     atgaaacacctgtggttctttctcctcctggtggcagctcccagatgggtcctgtctcaggtgcagctgcaggagtcgggcccag
IGHV4-4*04     atgaaacacctgtggttcttcctcctcctggtggcagctcccagatgggtcctgtctcaggtgcagctgcaggagtcgggcccag
IGHV4-4*05     atgaaacacctgtggttcttcctcctcctggtggcagctcccagatgggtcctgtctcaggtgcagctgcaggagttgggcccag
IGHV4-4*07     atgaaacacctgtggttcttcctcctgctggtggcagctcccagatgggtcctgtcccaggtgcagctgcaggagtcgggcccag
IGHV4-4*08     atgaaacacctgtggttcttcctcctcctggtggcagctcccagatgggtcctgtcccaggtgcagctgcaggagtcgggcccag
IGHV4-4*03     ---------------------------------------------------------caggtgcagctgcaggagtcgggcccag
IGHV4-4*06     -------------------------------------------------------------------------------------
IP_V4-4*p09    ---------------------------------------------------------caggtgcagctgcaggagtcgggcccag
IP_V4-4*p10    ---------------------------------------------------------caggtgcagctgcaggagtcgggcccag
IP_V4-4*p11    ---------------------------------------------------------caggtgcagctgcaggagtcgggcccag
IP_V4-4*p12    ---------------------------------------------------------caggtgcagctgcaggagtcgggcccag
V_V4-4*01      ---------------------------------------------------------caggtgcagctgcaggagtcgggcccag
V_V4-4*02      ---------------------------------------------------------caggtgcagctgcaggagtcgggcccag
V_V4-4*07      ---------------------------------------------------------caggtgcagctgcaggagtcgggcccag
```

```
V4-4_01      gactggtgaagccttcggagaccctgtccctcacctgcactgtctctggtggctccat---cagtagttactactggagctggatccggca
V4-4_02      gactggtgaagccttcggagaccctgtccctcacctgcactgtctctggtggctccat---cagtagttactactggagctggatccggca
V4-4_03      gactggtgaagccttcggagaccctgtccctcacctgcactgtctctggtggctccat---cagtagttactactggagctggatccggca
V4-4_04      gactggtgaagccttcggggaccctgtccctcacctgcgctgtctctggtggctccat---cagtagttactactggagctggatccggca
V4-4_05      gactggtgaagccttcggagaccctgtccctcacctgcactgtctctggtggctccat---cagtagttactactggagctggatccggca
V4-4_08      gactggtgaagccttcggagaccctgtccctcacctgcactgtctctggtggctccat---cagtagttactactggagctggatccggca
V4-4_10      gactggtgaagcctccggagaccctgtccctcacctgcactgtctctggtggctccat---cagtagttactactggagctggatccggca
V4-4_13      gactggtgaagccttcggggaccctgtccctcacctgcactgtctctggtggctccat---cagtagttactactggagctggatccggca
V4-4_14      gactggtgaagcctccggggaccctgtccctcacctgcgctgtctctggtggctccat---cagtagttactactggagctggatccggca
V4-4_15      gactggtgaagccttcggggaccctgtccctcacctgcgctgtctctggtggctccat---cagtagttactactggagctggatccggca
V4-4_16      gactggtgaagccttcggggaccctgtccctcacctgcactgtctctggtggctccat---cagtagttactactggagctggatccggca
V4-4_17      gactggtgaagccttcggggaccctgtccctcacctgcgctgtctctggtggctccat---cagtagttactactggagctggatccggca
V4-4_18      gactggtgaagccttcggagaccctgtccctcacctgcactgtctctggtggctccat---cagtagttactactggagctggatccggca
V4-4_20      gactggtgaagccttcggagaccctgtccctcacctgcgctgtctctggtggctccat---cagtagttactactggagctggatccggca
V4-4_21      gactggtgaagccttcggggaccctgtccctcacctgcactgtctctggtggctccat---cagtagttactactggagctggatccggca
V4-4_22      gactggtgaagccttcggggaccctgtccctcacctgcgctgtctctggtggctccat---cagtagttactactggagctggatccggca
V4-4_23      gactggtgaagcctccggggaccctgtccctcacctgcactgtctctggtggctccat---cagtagttactactggagctggatccggca
V4-4_25      gactggtgaagccttcacagaccctgtccctcacctgcactgtctctggtggctccat---cagtagttactactggagctggatccggca
V4-4_26      gactggtgaagccttcggggaccctgtccctcacctgcgctgtctctggtggctccat---cagtagttactactggagctggatccggca
V4-4_27      gactggtgaagccttcggggaccctgtccctcacctgcactgtctctggtggctccat---cagtagttactactggagctggatccggca
V4-4_30      gactggtgaagccttcggagaccctgtccctcacctgcactgtctctggtggctccat---cagtagttactactggagctggatccggca
V4-4_31      gactggtgaagccttcggagaccctgtccctcacctgcactgtctctggtggctccat---cagtagttactactggagctggatccggca
V4-4_32      gactggtgaagccttcggggaccctgtccctcacctgcgctgtctctggtggctccat---cagtagttactactggagctggatccggca
V4-4_34      gactggtgaagcctccggggaccctgtccctcacctgcgctgtctctggtggctccat---cagtagttactactggagctggatccggca
V4-4_35      gactggtgaagccttcacagaccctgtccctcacctgcactgtctctggtggctccat---cagtagttactactggagctggatccggca
V4-4_36      gactggtgaagcctccggagaccctgtccctcacctgcactgtctctggtggctccat---cagtagttactactggagctggatccggca
V4-4_40      gactggtgaagccttcggagaccctgtccctcacctgcactgtctctggtggctccat---cagtagttactactggagctggatccggca
V4-4_42      gactggtgaagccttcacagaccctgtccctcacctgcactgtctctggtggctccat---cagtagttactactggagctggatccggca
V4-4_43      gactggtgaagccttcggagaccctgtccctcacctgcgctgtctctggtggctccat---cagtagttactactggagctggatccggca
V4-4_44      gactggtgaagccttcggagaccctgtccctcacctgcgctgtctctggtggctccat---cagtagttactactggagctggatccggca
IGHV4-4*01   gactggtgaagcctccggggaccctgtccctcacctgcgctgtctctggtggctccatcagcagtagtaactggtggagttgggtccgcca
IGHV4-4*02   gactggtgaagccttcggggaccctgtccctcacctgcgctgtctctggtggctccatcagcagtagtaactggtggagttgggtccgcca
IGHV4-4*04   gactggtgaagcctccggggaccctgtccctcacctgcgctatctctggtggctccatcagcagtagtaactggtggagttgggtccgcca
IGHV4-4*05   gactggtgaagcctccggggaccctgtccctcacctgcgctgtctctggtggctccatcagcagtagtaactggtggagttgggtccgcca
IGHV4-4*07   gactggtgaagccttcggagaccctgtccctcacctgcactgtctctggtggctccat---cagtagttactactggagctggatccggca
IGHV4-4*08   gactggtgaagccttcggagaccctgtccctcacctgcactgtctctggtggctccat---cagtagttactactggagctggatccggca
IGHV4-4*03   gactggtgaagcctccggggaccctgtccctcacctgcgctgtctctggtggctccatcagcagtagtaactggtggagttgggtccgcca
IGHV4-4*06   --------------------------------------------tctggtggctccatcagcagtagtaactggtggagttgggtccgcca
IP_V4-4*p09  gactggtgaagccttcggagaccctgtccctcacctgcactgtctctggtggctccat---cagtagttactactggagctggatccggca
IP_V4-4*p10  gactggtgaagccttcggagaccctgtccctcacctgcactgtctctggtggctccat---cagtagttactactggagctggatccggca
IP_V4-4*p11  gactggtgaagccttcggagaccctgtccctcacctgcactgtctctggtggctccat---cagtagttactactggagctggatccggca
IP_V4-4*p12  gactggtgaagccttcggagaccctgtccctcacctgcactgtctctggtggctccat---cagtagttactactggagctggatccggca
V_V4-4*01    gactggtgaagcctccggggaccctgtccctcacctgcgctgtctctggtggctccatcagcagtagtaactggtggagttgggtccgcca
V_V4-4*02    gactggtgaagccttcggggaccctgtccctcacctgcgctgtctctggtggctccatcagcagtagtaactggtggagttgggtccgcca
V_V4-4*07    gactggtgaagccttcggagaccctgtccctcacctgcactgtctctggtggctccat---cagtagttactactggagctggatccggca
```

```
V4-4_01        gcccgccgggaagggactggagtggattgggcgtatctataccagtgggagcaccaactacaacccctccctcaagagtcgagtcaccatg
V4-4_02        gcccgccgggaagggactggagtggattgggcgtatctataccagtgggagcaccaactacaacccctccctcaagagtcgagtcaccatg
V4-4_03        gcccgccgggaagggactggagtggattgggcgtatctataccagtgggagcaccaactacaacccctccctcaagagtcgagtcaccata
V4-4_04        gcccgccgggaagggactggagtggattgggcgtatctataccagtgggagcaccaactacaacccctccctcaagagtcgagtcaccata
V4-4_05        gcccgccgggaagggactggagtggattgggcgtatctataccagtgggagcaccaactacaacccctccctcaagagtcgagtcaccata
V4-4_08        gcccgccgggaagggactggagtggattgggcgtatctataccagtgggagcaccaactacaacccctccctcaagagtcgagtcaccatg
V4-4_10        gcccgccgggaagggactggagtggattgggcgtatctataccagtgggagcaccaactacaacccctccctcaagagtcgagtcaccata
V4-4_13        gcccgccgggaagggactggagtggattgggcgtatctataccagtgggagcaccaactacaacccctccctcaagagtcgagtcaccata
V4-4_14        gcccgccgggaagggactggagtggattgggcgtatctataccagtgggagcaccaactacaacccctccctcaagagtcgagtcaccata
V4-4_15        gcccgccgggaagggactggagtggattgggcgtatctataccagtgggagcaccaactacaacccctccctcaagagtcgagtcaccata
V4-4_16        gcccgccgggaagggactggagtggattgggcgtatctataccagtgggagcaccaactacaacccctccctcaagagtcgagtcaccatg
V4-4_17        gcccgccgggaagggactggagtggattgggcgtatctataccagtgggagcaccaactacaacccgtccctcaagagtcgagtcaccata
V4-4_18        gcccgccgggaagggactggagtggattgggcatatctataccagtgggagcaccaactacaacccctccctcaagagtcgagtcaccata
V4-4_20        gcccgccgggaagggactggagtggattgggcgtatctataccagtgggagcaccaactacaacccctccctcaagagtcgagtcaccata
V4-4_21        gcccgccgggaagggactggagtggattgggcgtatctataccagtgggagcaccaactacaacccctccctcaagagtcgagtcaccatg
V4-4_22        gcccgccgggaagggactggagtggattgggcgtatctataccagtgggagcaccaactacaacccctccctcaagagtcgagtcaccatg
V4-4_23        gcccgccgggaagggactggagtggattgggcgtatctataccagtgggagcaccaactacaacccctccctcaagagtcgagtcaccata
V4-4_25        gcccgccgggaagggactggagtggattgggcgtatctataccagtgggagcaccaactacaacccctccctcaagagtcgagtcaccata
V4-4_26        gcccgccgggaagggactggagtggattgggcgtatctataccagtgggagcaccaactacaacccctccctcaagagtcgagtcaccatg
V4-4_27        gcccgccgggaagggactggagtggattgggcgtatctataccagtgggagcaccaactacaacccctccctcaagagtcgagtcaccatg
V4-4_30        gcccgccgggaagggactggagtggattgggcgtatctataccagtgggagcaccaactacaacccctccctcaagagtcgagtcaccatg
V4-4_31        gcccgccgggaagggactggagtggattgggcgtatctataccagtgggagcaccaactacaacccctccctcaagagtcgagtcaccata
V4-4_32        gcccgccgggaagggactggagtggattgggcgtatctataccagtgggagcaccaactacaacccctccctcaagagtcgagtcaccatg
V4-4_34        gcccgccgggaagggactggagtggattgggcgtatctataccagtgggagcaccaactacaacccctccctcaagagtcgagtcaccata
V4-4_35        gcccgccgggaagggactggagtggattgggcgtatctataccagtgggagcaccaactacaacccctccctcaagagtcgagtcaccatg
V4-4_36        gcccgccgggaagggactggagtggattgggcgtatctataccagtgggagcaccaactacaacccctccctcaagagtcgagtcaccatg
V4-4_40        gcccgccgggaagggactggagtggattgggcgtatctataccagtgggagcaccaactacaacccctccctcaagagtcgagtcaccatg
V4-4_42        gcccgccgggaagggactggagtggattgggcgtatctataccagtgggagcaccaactacaacccctccctcaagagtcgagtcaccatg
V4-4_43        gcccgccgggaagggactggagtggattgggcgtatctataccagtgggagcaccaactacaacccctccctcaagagtcgagtcaccatg
V4-4_44        gcccgccgggaagggactggagtggattgggcgtatctataccagtgggagcaccaactacaacccctccctcaagagtcgagtcaccata
IGHV4-4*01     gcccccagggaaggggctggagtggattggggaaatctatcatagtgggagcaccaactacaacccgtccctcaagagtcgagtcaccata
IGHV4-4*02     gcccccagggaaggggctggagtggattggggaaatctatcatagtgggagcaccaactacaacccgtccctcaagagtcgagtcaccata
IGHV4-4*04     gcccccagggaaggggctggagtggattggggaaatctatcatagtgggagcaccaactacaacccgtccctcaagagtcgagtcaccata
IGHV4-4*05     gcccccagggaaggggctggagtggattggggaaatctatcatagtgggagcaccaactacaacccgtccctcaagagtcgagtcaccata
IGHV4-4*07     gcccgccgggaagggactggagtggattgggcgtatctataccagtgggagcaccaactacaacccctccctcaagagtcgagtcaccatg
IGHV4-4*08     gcccccagggaagggactggagtggattgggtatatctataccagtgggagcaccaactacaacccctccctcaagagtcgagtcaccata
IGHV4-4*03     gcccccagggaaggggctggagtggattggggaaatctatcatagtgggagcaccaactacaacccgtccctcaagagtcgagtcaccata
IGHV4-4*06     gcccccagggannngggctggagtggattggggaaatctatcatagtgggagcaccaactacaacccgtccctcaagagtcgagtcaccatg
IP_V4-4*p09    gcccgccgggaagggactggagtggattgggcgtatctataccagtgggagcaccaactacaacccgtccctcaagagtcgagtcaccatg
IP_V4-4*p10    gcccgccgggaagggactggagtggattgggcgtatctataccagtgggagcaccaactacaacccctccttcaagagtcgagtcaccatg
IP_V4-4*p11    gcccgccgggaagggactggagtggattgggcgtatctataccagtgggagcaccaactacaacccctccctcaagagtcgagtcaccatg
IP_V4-4*p12    gcccgccgggaagggactggagtggattgggcgtatctataccagtgggagcaccaactacaacccctccctcaagagtcgagtcaccatg
V_V4-4*01      gcccccagggaaggggctggagtggattggggaaatctatcatagtgggagcaccaactacaacccgtccctcaagagtcgagtcaccata
V_V4-4*02      gcccccagggaaggggctggagtggattggggaaatctatcatagtgggagcaccaactacaacccgtccctcaagagtcgagtcaccata
V_V4-4*07      gcccgccgggaagggactggagtggattgggcgtatctataccagtgggagcaccaactacaacccctccctcaagagtcgagtcaccatg
```

```
V4-4_01        tcagtagacacgtccaagaaccagttctccctgaagctgagctctgtgaccgccgcggacacggccgtgtattactgtgcgaga
V4-4_02        tcagtagacacgtccaagaaccagttctccctgaagctgagctctgtgaccgccgcggacacggccgtgtattactgtgcgaga
V4-4_03        tcagtagacacgtccaagaaccagttctccctgaagctgagctctgtgaccgccgcggacacggccgtgtattactgtgcgaga
V4-4_04        tcagtagacacgtccaagaaccagttctccctgaagctgagctctgtgaccgccgcggacacggccgtgtattactgtgcgaga
V4-4_05        tcagtagacacgtccaagaaccagttctccctgaagctgagctctgtgaccgccgcggacacggccgtgtattactgtgcgaga
V4-4_08        tcagtagacacgtccaagaaccagttctccctgaagctgagctctgtgaccgccgcggacacggccgtgtattactgtgcgaga
V4-4_10        tcagtagacacgtccaagaaccagttctccctgaagctgagctctgtgaccgccgcggacacggccgtgtattgctgtgcgaga
V4-4_13        tcagtagacacgtccaagaaccagttctccctgaagctgagctctgtgaccgccgcggacacggccgtgtattactgtgcgaga
V4-4_14        tcagtagacacgtccaagaaccagttctccctgaagctgagctctgtgaccgccgcggacacggccgtgtattgctgtgcgaga
V4-4_15        tcagtagacacgtccaagaaccagttctccctgaagctgagctctgtgaccgccgcggacacggccgtgtattactgtgcgaga
V4-4_16        tcagtagacacgtccaagaaccagttctccctgaagctgagctctgtgaccgccgcggacacggccgtgtattactgtgcgaga
V4-4_17        tcagtagacacgtccaagaaccagttctccctgaagctgagctctgtgaccgccgcggacacggccgtgtattactgtgcgaga
V4-4_18        tcagtagacacgtccaagaaccagttctccctgaagctgagctctgtgaccgccgcggacacggccgtgtattactgtgcgaga
V4-4_20        tcagtagacacgtccaagaaccagttctccctgaagctgagctctgtgaccgccgcggacacggccgtgtattactgtgcgaga
V4-4_21        tcagtagacacgtccaagaaccagttctccctgaagctgagctctgtgaccgccgcggacacggccgtgtattactgtgcgaga
V4-4_22        tcagtagacacgtccaagaaccagttctccctgaagctgagctctgtgaccgccgcggacacggccgtgtattactgtgcgaga
V4-4_23        tcagtagacacgtccaagaaccagttctccctgaagctgagctctgtgaccgccgcggacacggccgtgtattgctgtgcgaga
V4-4_25        tcagtagacacgtccaagaaccagttctccctgaagctgagctctgtgaccgccgcggacacggccgtgtattactgtgcgaga
V4-4_26        tcagtagacacgtccaagaaccagttctccctgaagctgagctctgtgaccgccgcggacacggccgtgtattactgtgcgaga
V4-4_27        tcagtagacacgtccaagaaccagttctccctgaagctgagctctgtgaccgccgcggacacggccgtgtattactgtgcgaga
V4-4_30        tcagtagacacgtccaagaaccagttctccctgaagctgagctctgtgaccgccgcggacacggccgtgtattactgtgcgaga
V4-4_31        tcagtagacacgtccaagaaccagttctccctgaagctgagctctgtgaccgccgcggacacggccgtgtattactgtgcgaga
V4-4_32        tcagtagacacgtccaagaaccagttctccctgaagctgagctctgtgaccgccgcggacacggccgtgtattactgtgcgaga
V4-4_34        tcagtagacacgtccaagaaccagttctccctgaagctgagctctgtgaccgccgcggacacggccgtgtattactgtgcgaga
V4-4_35        tcagtagacacgtccaagaaccagttctccctgaagctgagctctgtgaccgccgcggacacggccgtgtattactgtgcgaga
V4-4_36        tcagtagacacgtccaagaaccagttctccctgaagctgagctctgtgaccgccgcggacacggccgtgtattgctgtgcgaga
V4-4_40        tcagtagacacgtccaagaaccagttctccctgaagctgagctctgtgaccgccgcggacacggccgtgtattgctgtgcgaga
V4-4_42        tcagtagacacgtccaagaaccagttctccctgaagctgagctctgtgaccgccgcggacacggccgtgtattactgtgcgaga
V4-4_43        tcagtagacacgtccaagaaccagttctccctgaagctgagctctgtgaccgccgcggacacggccgtgtattactgtgcgaga
V4-4_44        tcagtagacacgtccaagaaccagttctccctgaagctgagctctgtgaccgccgcggacacggccgtgtattactgtgcgaga
IGHV4-4*01     tcagtagacaagtccaagaaccagttctccctgaagctgagctctgtgaccgccgcggacacggccgtgtattgctgtgcgaga
IGHV4-4*02     tcagtagacaagtccaagaaccagttctccctgaagctgagctctgtgaccgccgcggacacggccgtgtattactgtgcgaga
IGHV4-4*04     tcagtagacaagtccaagaaccagttctccctgaagctgagctctgtgaccgccgcggacacggccgtgtattactg-------
IGHV4-4*05     tcagtagacaagtccaagaaccagttctccctgaagctgagctctgtgaccgccgcggacacggccgtgtattactg-------
IGHV4-4*07     tcagtagacacgtccaagaaccagttctccctgaagctgagctctgtgaccgccgcggacacggccgtgtattactgtgcgaga
IGHV4-4*08     tccgtagacacgtccaagaaccagttctccctgaagctgagctctgtgaccgccgcagacacggccgtgtattactgtgcgaga
IGHV4-4*03     tcagtagacaagtccaagaaccagttctccctgaagctgagctctgtgaccgccgcggacacggccgtgtattactgtgcgaga
IGHV4-4*06     tcagtagacacgtccaagaaccagttctccctgaagctgagctctgtgaccgccgcggacacggccgtgtattactgtgcgaga
IP_V4-4*p09    tcagtagacacgtccaagaaccagttctccctgaagctgagctctgtg------------------------------------
IP_V4-4*p10    tcagtagacacgtccaagaaccagttctccctgaagctgagctctgtgaccgccgcggacacggccgtgtattactgtgcgaga
IP_V4-4*p11    tcagtagacacgtccaagaaccagttctccctgaagctgagctctgtgaccgccgcggacacggccgtgtattgctgtgcgaga
IP_V4-4*p12    tcagtagacacgtccaagaaccagttctccctgaagctgagctctgtgaccgccgcggacacggctgtgtattactgtgcgaga
V_V4-4*01      tcagtagacaagtccaagaaccagttctccctgaagctgagctctgtgaccgccgcggacacggccgtgtattgctgtgcgaga
V_V4-4*02      tcagtagacaagtccaagaaccagttctccctgaagctgagctctgtgaccgccgcggacacggccgtgtattactgtgcgaga
V_V4-4*07      tcagtagacacgtccaagaaccagttctccctgaagctgagctctgtgaccgccgcggacacggccgtgtattactgtgcgaga
```

IGHV4-28

```
V4-28_01        atgaaacacctgtggttcttcctcctgctggtggcagctcccagatgggtcctgtcccaggtgcagctgcaggagtcgggcccaggactgg
V4-28_02        atgaaacacctgtggttcttcctcctgctggtggcagctcccagatgggtcctgtcccaggtgcagctgcaggagtcgggcccaggactgg
V4-28_03        atgaaacacctgtggttcttcctcctgctggtggcagctcccagatgggtcctgtcccaggtgcagctgcaggagtcgggcccaggactgg
V4-28_04        atgaaacacctgtggttcttcctcctgctggtggcagctcccagatgggtcctgtcccaggtacagctgcaggagtcgggcccaggactgg
V4-28_05        atgaaacacctgtggttcttcctcctgctggtggcagctcccagattggtcctgtcccaggtgcagctgcaggagtcgggcccaggactgg
V4-28_06        atgaaacacctgtggttcttcctcctgctggtggcagctcccagatgggtcctgtcccaggtacagctgcaggagtcgggcccaggactgg
V4-28_07        atgaaacacctgtggttcttcctcctgctggtggcagctcccagattggtcctgtcccaggtacagctgcaggagtcgggcccaggactgg
V4-28_08        atgaaacacctgtggttcttcctcctgctggtggcagctcccagatgggtcctgtcccaggtgcagctgcaggagtcgggcccaggactgg
V4-28_09        atgaaacacctgtggttcttcctcctgctggtggcagctcccagattggtcctgtcccaggtgcagctgcaggagtcgggcccaggactgg
V4-28_10        atgaaacacctgtggttcttcctcctgctggtggcagctcccagattggtcctgtcccaggtgcagctgcaggagtcgggcccaggactgg
V4-28_11        atgaaacacctgtggttcttcctcctgctggtggcagctcccagatgggtcctgtcccaggtgcagctgcaggagtcgggcccaggactgg
V4-28_12        atgaaacacctgtggttcttcctcctgctggtggcagctcccagatgggtcctgtcccaggtgcagctgcaggagtcgggcccaggactgg
V4-28_13        atgaaacacctgtggttcttcctcctgctggtggcagctcccagatgggtcctgtcccaggtgcagctgcaggagtcgggcccaggactgg
V4-28_14        atgaaacacctgtggttcttcctcctgctggtggcagctcccagatgggtcctgtcccaggtgcagctgcaggagtcgggcccaggactgg
V4-28_15        atgaaacacctgtggttcttcctcctgctggtggcagctcccagatgggtcctgtcccaggtacagctgcaggagtcgggcccaggactgg
V4-28_16        atgaaacacctgtggttcttcctcctgctggtggcagctcccagatgggtcctgtcccaggtgcagctgcaggagttgggcccaggactgg
V4-28_17        atgaaacacctgtggttcttcctcctgctggtggcagctcccagatgggtcctgtcccaggtgcagctgcaggagtcgggcccaggactgg
V4-28_18        atgaaacacctgtggttcttcctcctgctggtggcagctcccagattggtcctgtcccaggtgcagctgcaggagtcgggcccaggactgg
IGHV4-28*01     atgaaacacctgtggttcttcctcctgctggtggcagctcccagatgggtcctgtcccaggtgcagctgcaggagtcgggcccaggactgg
IGHV4-28*02     atgaaacacctgtggttcttcctcctgctggtggcagctcccagatgggtcctgtcccaggtgcagctgcaggagtcgggcccaggactgg
IGHV4-28*03     atgaaacacctgtggttcttcctcctgctggtggcagctcccagatgggtcctgtctcaggtgcagctgcaggagtcgggcccaggactgg
IGHV4-28*07     atgaaacacctgtggttcttcctcctgctggtggcagctcccagatgggtcctgtcccaggtacagctgcaggagtcgggcccaggactgg
IGHV4-28*04     ---------------------------------------------------------caggtgcagctgcaggagtcgggcccaggactgg
IGHV4-28*05     ---------------------------------------------------------caggtgcagctgcaggagtcgggcccaggactgg
IGHV4-28*06     ---------------------------------------------------------caggtgcagctacaggagtcgggcccaggactgg
IP_V4-28*p08    ---------------------------------------------------------caggtgcagctgcaggagtcgggcccaggactgg
IP_V4-28*p09    ---------------------------------------------------------caggtgcagctgcaggagtcgggcccaggactgg
V_V4-28*01      ---------------------------------------------------------caggtgcagctgcaggagtcgggcccaggactgg
V_V4-28*02      ---------------------------------------------------------caggtgcagctgcaggagtcgggcccaggactgg
V_V4-28*05      ---------------------------------------------------------caggtgcagctgcaggagtcgggcccaggactgg
```

V4-28_01	tgaagccttcggacaccctgtccctcacctgcgctgtctctggttactccatcagcagtagtaactggtggggctggatccggcagccc
V4-28_02	tgaagccttcacagaccctgtccctcacctgcgctgtctctggttactccatcagcagtagtaactggtggggctggatccggcagccc
V4-28_03	tgaagccttcggacaccctgtccctcacctgcgctgtctctggttactccatcagcagtagtaactggtggggctggatccggcagccc
V4-28_04	tgaagccttcggacaccctgtccctcacctgcgctgtctctggttactccatcagcagtagtaactggtggggctggatccggcagccc
V4-28_05	tgaagccttcggacaccctgtccctcacctgcgctgtctctggttactccatcagcagtagtaactggtggggctggatccggcagccc
V4-28_06	tgaagccttcggacaccctgtccctcacctgcgctgtctctggttactccatcagcagtagtaactggtggggctggatccggcagccc
V4-28_07	tgaagccttcggacaccctgtccctcacctgcgctgtctctggttactccatcagcagtagtaactggtggggctggatccggcagccc
V4-28_08	tgaagccttcggacaccctgtccctcacctgcgctgtctctggttactccatcagcagtagtaactggtggggctggatccggcagccc
V4-28_09	tgaagccttcggacaccctgtccctcacctgcgctgtctctggttactccatcagcagtagtaactggtggggctggatccggcagccc
V4-28_10	tgaagccttcacagaccctgtccctcacctgcgctgtctctggttactccatcagcagtagtaactggtggggctggatccggcagccc
V4-28_11	tgaagccttcggacaccctgtccctcacctgcgctgtctctggttactccatcagcagtagtaactggtggggctggatccggcagccc
V4-28_12	tgaagccttcggacaccctgtccctcacctgcgctgtctctggttactccatcagcagtagtaactggtggggctggatccggcagccc
V4-28_13	tgaagccttcggggaccctgtccctcacctgcactgtctctggtggctccatcagcagtagtaactggtggggctggatccggcagccc
V4-28_14	tgaagccttcggacaccctgtccctcacctgcgctgtctctggttactccatcagcagtagtaactggtggggctggatccggcagccc
V4-28_15	tgaagccttcacagaccctgtccctcacctgcgctgtctctggttactccatcagcagtagtaactggtggggctggatccggcagccc
V4-28_16	tgaagccttcggacaccctgtccctcacctgcgctgtctctggttactccatcagcagtagtaactggtggggctggatccggcagccc
V4-28_17	tgaagccttcgggcaccctgtccctcacctgcactgtctctggtggctccatcagcagtagtaactggtggggctggatccggcagccc
V4-28_18	tgaagccttcacacaccctgtccctcacctgcgctgtctctggttactccatcagcagtagtaactggtggggctggatccggcagccc
IGHV4-28*01	tgaagccttcggacaccctgtccctcacctgcgctgtctctggttactccatcagcagtagtaactggtggggctggatccggcagccc
IGHV4-28*02	tgaagccttcacagaccctgtccctcacctgcgctgtctctggttactccatcagcagtagtaactggtggggctggatccggcagccc
IGHV4-28*03	tgaagccttcggacaccctgtccctcacctgcgctgtctctggttactccatcagcagtagtaactggtggggctggatccggcagccc
IGHV4-28*07	tgaagccttcggacaccctgtccctcacctgcgctgtctctggttactccatcagcagtagtaactggtggggctggatccggcagccc
IGHV4-28*04	tgaagccttcggacaccctgtccctcacctgcgctgtctctggttactccatcagcagtagtaactggtggggctggatccggcagccc
IGHV4-28*05	tgaagccttcggacaccctgtccctcacctgcgctgtctctggttactccatcagcagtagtaactggtggggctggatccggcagccc
IGHV4-28*06	tgaagccttcggacaccctgtccctcacctgcgctgtctctggttactccatcagcagtagtaactggtggggctggatccggcagccc
IP_V4-28*p08	tgaagccttcggacaccctgtccctcacctgcgctgtctctggttactccatcagcagtagtaactggtggggctggatccggcagccc
IP_V4-28*p09	tgaagccttcggggaccctgtccctcacctgcactgtctctggtggctccatcagcagtagtaactggtggggctggatccggcagccc
V_V4-28*01	tgaagccttcggacaccctgtccctcacctgcgctgtctctggttactccatcagcagtagtaactggtggggctggatccggcagccc
V_V4-28*02	tgaagccttcacagaccctgtccctcacctgcgctgtctctggttactccatcagcagtagtaactggtggggctggatccggcagccc
V_V4-28*05	tgaagccttcggacaccctgtccctcacctgcgctgtctctggttactccatcagcagtagtaactggtggggctggatccggcagccc

V4-28_01	ccagggaagggactggagtggattgggtacatctattatagtgggagcacctactacaacccgtccctcaagagtcgagtcaccatgtca
V4-28_02	ccagggaagggactggagtggattgggtacatctattatagtgggagcatctactacaacccgtccctcaagagtcgagtcaccatgtca
V4-28_03	ccagggaagggactggagtggattgggtacatctattatagtgggagcatctactacaacccgtccctcaagagtcgagtcaccatgtca
V4-28_04	ccagggaagggactggagtggattgggtacatctattatagtgggagcacctactacaacccgtccctcaagagtcgagtcaccatgtca
V4-28_05	ccagggaagggactggagtggattgggtacatctattatagtgggagcacctactacaacccgtccctcaagagtcgagtcaccatgtca
V4-28_06	ccagggaagggactggagtggattgggtacatctattatagtgggagcatctactacaacccgtccctcaagagtcgagtcaccatgtca
V4-28_07	ccagggaagggactggagtggattgggtacatctattatagtgggagcacctactacaacccgtccctcaagagtcgagtcaccatgtca
V4-28_08	ccagggaagggactggagtggattgggtacatctattatagtgggagcaccaactacaacccgtccctcaagagtcgagtcaccatgtca
V4-28_09	ccagggaagggactggagtggattgggtacatctattatagtgggagcatctactacaacccgtccctcaagagtcgagtcaccatgtca
V4-28_10	ccagggaagggactggagtggattgggtacatctattatagtgggagcatctactacaacccgtccctcaagagtcgagtcaccatgtca
V4-28_11	ccagggaagggactggcgtggattgggtacatctattatagtgggagcacctactacaacccgtccctcaagagtcgagtcaccatgtca
V4-28_12	ccagggaagggactggagtggattgggtacatctattatagtgggagcatcaactacaacccgtccctcaagagtcgagtcaccatgtca
V4-28_13	ccagggaagggactggagtggattgggtacatctattatagtgggagcaccaactacaacccgtccctcaagagtcgagtcaccatgtca
V4-28_14	ccagggaagggactggcgtggattgggtacatctattatagtgggagcatctactacaacccgtccctcaagagtcgagtcaccatgtca
V4-28_15	ccagggaagggactggagtggattgggtacatctattatagtgggagcatctactacaacccgtccctcaagagtcgagtcaccatgtca
V4-28_16	ccagggaagggactggagtggattgggtacatctattatagtgggagcatctactacaacccgtccctcaagagtcgagtcaccatgtca
V4-28_17	ccagggaagggactggagtggattgggtacatctattatagtgggagcaccaactacaacccgtccctcaagagtcgagtcaccatgtca
V4-28_18	ccagggaagggactggagtggattgggtacatctattatagtgggagcatctactacaacccgtccctcaagagtcgagtcaccatgtca
IGHV4-28*01	ccagggaagggactggagtggattgggtacatctattatagtgggagcacctactacaacccgtccctcaagagtcgagtcaccatgtca
IGHV4-28*02	ccagggaagggactggagtggattgggtacatctattatagtgggagcatctactacaacccgtccctcaagagtcgagtcaccatgtca
IGHV4-28*03	ccagggaagggactggagtggattgggtacatctattatagtgggagcacctactacaacccgtccctcaagagtcgagtcaccatgtca
IGHV4-28*07	ccagggaagggactggagtggattgggtacatctattatagtgggagcacctactacaacccgtccctcaagagtcgagtcaccatgtca
IGHV4-28*04	ccagggaagggactggagtggattgggtacatctattatagtgggagcacctactacaacccgtccctcaagagtcgagtcaccatgtca
IGHV4-28*05	ccagggaagggactggagtggattgggtacatctattatagtgggagcatctactacaacccgtccctcaagagtcgagtcaccatgtca
IGHV4-28*06	ccagggaagggactggagtggattgggtacatctattatagtgggagcaccaactacaacccgtccctcaagagtcgagtcaccatgtca
IP_V4-28*p08	ccagggaagggactggagtggattgggtacatctattatagtgggagcaccaactacaacccgtccctcaagagtcgagtcaccatgtca
IP_V4-28*p09	ccagggaagggactggagtggattgggtacatctattatagtgggagcaccaactacaacccgtccctcaagagtcgagtcaccatgtca
V_V4-28*01	ccagggaagggactggagtggattgggtacatctattatagtgggagcacctactacaacccgtccctcaagagtcgagtcaccatgtca
V_V4-28*02	ccagggaagggactggagtggattgggtacatctattatagtgggagcatctactacaacccgtccctcaagagtcgagtcaccatgtca
V_V4-28*05	ccagggaagggactggagtggattgggtacatctattatagtgggagcatctactacaacccgtccctcaagagtcgagtcaccatgtca

```
V4-28_01       gtagacacgtccaagaaccagttctccctgaagctgagctctgtgaccgccgtggacacggccgtgtattactgtgcgaga
V4-28_02       gtagacacgtccaagaaccagttctccctgaagctgagctctgtgaccgccgtggacacggccgtgtattactgtgcgaga
V4-28_03       gtagacacgtccaagaaccagttctccctgaagctgagctctgtgaccgccgtggacacggccgtgtattactgtgcgaga
V4-28_04       gtagacacgtccaagaaccagttctccctgaagctgagctctgtgaccgccgtggacacggccgtgtattactgtgcgaga
V4-28_05       gtagacacgtccaagaaccagttctccctgaagctgagctctgtgaccgccgtggacacggccgtgtattactgtgcgaga
V4-28_06       gtagacacgtccaagaaccagttctccctgaagctgagctctgtgaccgccgtggacacggccgtgtattactgtgcgaga
V4-28_07       gtagacacgtccaagaaccagttctccctgaagctgagctctgtgaccgccgtggacacggccgtgtattactgtgcgaga
V4-28_08       gtagacacgtccaagaaccagttctccctgaagctgagctctgtgaccgccttggacacggccgtgtattactgtgcgaga
V4-28_09       gtagacacgtccaagaaccagttctccctgaagctgagctctgtgaccgccgtggacacggccgtgtattactgtgcgaga
V4-28_10       gtagacacgtccaagaaccagttctccctgaagctgagctctgtgaccgccgtggacacggccgtgtattactgtgcgaga
V4-28_11       gtagacacgtccaagaaccagttctccctgaagctgagctctgtgaccgccgtggacacggccgtgtattactgtgcgaga
V4-28_12       gtagacacgtccaagaaccagttctccctgaagctgagctctgtgaccgccttggacacggccgtgtattactgtgcgaga
V4-28_13       gtagacacgtccaagaaccagttctccctgaagctgagctctgtgaccgccttggacacggccgtgtattactgtgcgaga
V4-28_14       gtagacacgtccaagaaccagttctccctgaagctgagctctgtgaccgccgtggacacggccgtgtattactgtgcgaga
V4-28_15       gtagacacgtccaagaaccagttctccctgaagctgagctctgtgaccgccgtggacacggccgtgtattactgtgcgaga
V4-28_16       gtagacacgtccaagaaccagttctccctgaagctgagctctgtgaccgccgtggacacggccgtgtattactgtgcgaga
V4-28_17       gtagacacgtccaagaaccagttctccctgaagctgagctctgtgaccgccttggacacggccgtgtattactgtgcgaga
V4-28_18       gtagacacgtccaagaaccagttctccctgaagctgagctctgtgaccgccgtggacacggccgtgtattactgtgcgaga
IGHV4-28*01    gtagacacgtccaagaaccagttctccctgaagctgagctctgtgaccgccgtggacacggccgtgtattactgtgcgaga
IGHV4-28*02    gtagacacgtccaagaaccagttctccctgaagctgagctctgtgaccgccgtggacacggccgtgtattactgtgcgaga
IGHV4-28*03    gtagacacgtccaagaaccagttctccctgaagctgagctctgtgaccgccgtggacacggccgtgtattactgtgcgaga
IGHV4-28*07    gtagacacgtccaagaaccagttctccctgaagctgagctctgtgaccgccgtggacacggccgtgtattactgtgcgaga
IGHV4-28*04    gtagacacgtccaagaaccagttctccctgaagctgagctctgtgaccgccgtggacaccggcgtgtattactgtgcgaga
IGHV4-28*05    gtagacacgtccaagaaccagttctccctgaagctgagctctgtgaccgccgtggacacggccgtgtattactgtgcgaga
IGHV4-28*06    gtagacacgtccaagaaccagttctccctgaagctgagctctgtgaccgccttggacacggccgtgtattactgtgcgaga
IP_V4-28*p08   gtagacacgtccaagaaccagttctccctgaagctgagctctgtgaccgccttggacacggccgtgtattactgtgcgaga
IP_V4-28*p09   gtagacacgtccaagaaccagttctccctgaagctgagctctgtgaccgccttggacacggccgtgtattactgtgcgaga
V_V4-28*01     gtagacacgtccaagaaccagttctccctgaagctgagctctgtgaccgccgtggacacggccgtgtattactgtgcgaga
V_V4-28*02     gtagacacgtccaagaaccagttctccctgaagctgagctctgtgaccgccgtggacacggccgtgtattactgtgcgaga
V_V4-28*05     gtagacacgtccaagaaccagttctccctgaagctgagctctgtgaccgccgtggacacggccgtgtattactgtgcgaga
```

IGHV4-31

```
V4-31_01      atgaaacacctgtggttcttcctcctgctggtggcagctcccagatgggtcctgccccaggtgcagctgcaggagtcgggcccaggactg
V4-31_02      atgaaacacctgtggttcttcctcctgctggtggcagctcccagatgggtcctgccccaggtgcagctgcaggagtcgggcccaggactg
V4-31_03      atgaaacacctgtggttcttcctcctgctggtggcagctcccagatgggtcctgccccaggtgcagctgcaggagtcgggcccaggactg
V4-31_04      atgaaacacctgtggttcttcctcctgctggtggcagctcccagatgggtcctgccccaggtgcagctgcaggagtcgggcccaggactg
V4-31_05      atgaaacacctgtggttcttcctcctgctggtggcagctcccagatgggtcctgccccaggtgcagctgcaggagtcgggcccaggactg
V4-31_06      atgaaacacctgtggttcttcctcctgctggtggcagctcccagatgggtcctgccccaggtgcagctgcaggagtcgggcccaggactg
V4-31_07      atgaaacacctgtggttcttcctcctgctggtggcagctcccagatgggtcctgccccaggtgcagctgcaggagtcgggcccaggactg
V4-31_08      atgaaacacctgtggttcttcctcctgctggtggcagctcccagatgggtcctgccccaggtgcagctgcaggagtcgggcccaggactg
V4-31_09      atgaaacacctgtggttcttcctcctgctggtggcagctcccagatgggtcctgccccaggtgcagctgcaggagtcgggcccaggactg
V4-31_10      atgaaacacctgtggttcttcctcctgctggtggcagctcccagatgggtcctgccccaggtgcagctgcaggagtcgggcccaggactg
V4-31_11      atgaaacacctgtggttcttcctcctgctggtggcagctcccagatgggtcctgccccaggtgcagctgcaggagtcgggcccaggactg
V4-31_12      atgaaacatctgtggttcttcctcctcctggtggcagctcccagatgggtcctgccccaggtgcagctgcaggagtcgggcccaggattg
V4-31_13      atgaaacacctgtggttcttcctcctgctggtggcagctcccagatgggtcctgccccaggtgcagctgcaggagtcgggcccaggactg
V4-31_14      atgaaacatctgtggttcttcctcctcctggtggcagctcccagatgggtcctgccccaggtgcagctgcaggagtcgggcccaggactg
V4-31_15      atgaaacatctgtggttcttcctcctcctggtggcagctcccagatgggtcctgccccaggtgcagctgcaggagtcgggcccaggactg
V4-31_16      atgaaacacctgtggttcttcctcctgctggtggcagctcccagatgggtcctgccccaggtgcagctgcaggagtcgggcccaggactg
V4-31_17      atgaaacacctgtggttcttcctcctgctggtggcagctcccagatgggtcctgccccaggtgcagctgcaggagtcgggcccaggactg
V4-31_18      atgaaacatctgtggttcttcctcctcctggtggcagctcccagatgggtcctgccccaggtgcagctgcaggagtcgggcccaggactg
V4-31_19      atgaaacacctgtggttcttcctcctgctggtggcagctcccagatgggtcctgccccaggtgcagctgcaggagtcgggcccaggactg
V4-31_20      atgaaacacctgtggttcttcctcctgctggtggcagctcccagatgggtcctgccccaggtgcagctgcaggagtcgggcccaggactg
V4-31_21      atgaaacacctgtggttcttcctcctgctggtggcagctcccagatgggtcctgccccaggtgcagctgcaggagtcgggcccaggactg
V4-31_22      atgaaacacctgtggttcttcctcctgctggtggcagctcccagatgggtcctgccccaggtgcagctgcaggagtcgggcccaggactg
V4-31_23      atgaaacacctgtggttcttcctcctgctggtggcagctcccagatgggtcctgccccaggtgcagctgcaggagtcgggcccaggactg
V4-31_24      atgaaacacctgtggttcttcctcctgctggtggcagctcccagatgggtcctgccccaggtgcagctgcaggagtcgggcccaggactg
V4-31_25      atgaaacacctgtggttcttcctcctgctggtggcagctcccagatgggtcctgccccaggtgcagctgcaggagtcgggcccaggactg
V4-31_26      atgaaacacctgtggttcttcctcctgctggtggcagctcccagatgggtcctgccccaggtgcagctgcaggagtcgggcccaggattg
IGHV4-31*02   atgaaacacctgtggttcttcctcctgctggtggcagctcccagatgggtcctgccccaggtgcagctgcaggagtcgggcccaggactg
IGHV4-31*03   atgaaacacctgtggttcttcctcctgctggtggcagctcccagatgggtcctgtcccaggtgcagctgcaggagtcgggcccaggactg
IGHV4-31*06   atgaaacacctgtggttcttcctcctgctggtggcagctcccagatgggtcctgtcccaggtgcagctgcaggagtcgggcccaggactg
IGHV4-31*07   atgaaacacctgtggttcttcctcctgctggtggcagctcccagatgggtcctgtcccaggtgcagctgcaggagtcgggcccaggactg
IGHV4-31*08   atgaaacacctgtggttcttcctcctgctggtggcagctcccagatgggtcctgtcccaggtgcagctgcaggagtcgggcccaggactg
IGHV4-31*09   atgaaacacctgtggttcttcctcctgctggtggcagctcccagatgggtcctgtcccaggtgcagctgcaggagtcgggcccaggactg
IGHV4-31*10   atgaaacacctgtggttcttcctcctgctggtggcagctcccagatgggtcctgtcccaggtgcagctgcaggagtcgggcccaggactg
IGHV4-31*01   ---------------------------------------------------------caggtgcagctgcaggagtcgggcccaggactg
IGHV4-31*04   ---------------------------------------------------------caggtgcggctgcaggagtcgggcccaggactg
IGHV4-31*05   ---------------------------------------------------------caggtgcagctgcaggagtcgggcccaggactg
IGHV4-31*11   ---------------------------------------------------------caggtgcagctgcaggagtcgggcccaggactg
IP_V4-31*p11  ---------------------------------------------------------caggtgcagctgcaggagtcgggcccaggactg
IP_V4-31*p12  ---------------------------------------------------------caggtgcagctgcaggagtcgggcccaggactg
IP_V4-31*p13  ---------------------------------------------------------caggtgcagctgcaggagtcgggcccaggactg
V_V4-31*02    ---------------------------------------------------------caggtgcagctgcaggagtcgggcccaggactg
V_V4-31*03    ---------------------------------------------------------caggtgcagctgcaggagtcgggcccaggactg
```

```
V4-31_01        gtgaagccttcacagaccctgtccctcacctgtactgtctctggtggctccatcagcagtggtggttactactggagctggatccgccag
V4-31_02        gtgaagccttcacagaccctgtccctcacctgtactgtctctggtggctccatcagcagtggtggttactactggagctggatccgccag
V4-31_03        gtgaagccttcacagaccctgtccctcacctgtactgtctctggtggctccatcagcagtggtggttactactggagctggatccgccag
V4-31_04        gtgaagccttcacagaccctgtccctcacctgtactgtctctggtggctccatcagcagtggtggttactactggagctggatccgccag
V4-31_05        gtgaagccttcacagaccctgtccctcacctgtactgtctctggtggctccatcagcagtggtggttactactggagctggatccgccag
V4-31_06        gtgaagccttcacagaccctgtccctcacctgtactgtctctggtggctccatcagcagtggtggttactactggagctggatccgccag
V4-31_07        gtgaagccttcacagaccctgtccctcacctgtactgtctctggtggctccatcagcagtggtggttactactggagctggatccgccag
V4-31_08        gtgaagccttcacagaccctgtccctcacctgtactgtctctggtggctccatcagcagtggtggttactactggagctggatccgccag
V4-31_09        gtgaagccttcacagaccctgtccctcacctgtgctgtctctggtggctccatcagcagtggtggttactactggagctggatccgccag
V4-31_10        gtgaagccttcacagaccctgtccctcacctgtactgtctctggtggctccatcagcagtggtggttactactggagctggatccgccag
V4-31_11        gtgaagccttcacagaccctgtccctcacctgtgctgtctctggtggctccatcagcagtggtggttactactggagctggatccgccag
V4-31_12        gtgaagccttcacagaccctgtccctcacctgtactgtctctggtggctccatcagcagtggtagttactactggagctggatccggcag
V4-31_13        gtgaagccttcacagaccctgtccctcacctgtgctgtctctggtggctccatcagcagtggtggttactactggagctggatccgccag
V4-31_14        gtgaagccttcacagaccctgtccctcacctgtactgtctctggtggctccatcagcagtggtagttactactggagctggatccggcag
V4-31_15        gtgaagccttcacagaccctgtccctcacctgtactgtctctggtggctccatcagcagtggtagttactactggagctggatccgccag
V4-31_16        gtgaagccttcacagaccctgtccctcacctgtactgtctctggtggctccatcagcagtggtggttactactggagctggatccgccag
V4-31_17        gtgaagccttcacagaccctgtccctcacctgtactgtctctggtggctccattagcagtggtggttactactggagctggatccgccag
V4-31_18        gtgaagccttcacagaccctgtccctcacctgtactgtctctggtggctccatcagcagtggtagttactactggagctggatccggcag
V4-31_19        gtgaagccttcacagaccctgtccctcacctgtactgtctctggtggctccatcagcagtggtggttactactggagctggatccggcag
V4-31_20        gtgaagccttcacagaccctgtccctcacctgtactgtctctggtggctccatcagcagtggtggttactactggagctggatccggcag
V4-31_21        gtgaagccttcacagaccctgtccctcacctgtactgtctctggtggctccatcagcagtggtggttactactggagctggatccgccag
V4-31_22        gtgaagccttcacagaccctgtccctcacctgtactgtctctggtggctccatcagcagtggtggttactactggagctggatccgccag
V4-31_23        gtgaagccttcacagaccctgtccctcacctgtgctgtctctggtggctccatcagcagtggtggttactactggagctggatccgccag
V4-31_24        gtgaagccttcacagaccctgtccctcacctgtgctgtctctggtggctccatcagcagtggtggttactactggagctggatccgccag
V4-31_25        gtgaagccttcacagaccctgtccctcacctgtactgtctctggtggctccatcagcagtggtggttactactggagctggatccgccag
V4-31_26        gtgaagccttcacagaccctgtccctcacctgtactgtctctggtggctccatcagcagtggtggttactactggagctggatccgccag
IGHV4-31*02     gtgaagccttcacagaccctgtccctcacctgtactgtctctggtggctccatcagcagtggtggttactactggagctggatccgccag
IGHV4-31*03     gtgaagccttcacagaccctgtccctcacctgcactgtctctggtggctccatcagcagtggtggttactactggagctggatccgccag
IGHV4-31*06     gtgaagccttcacagaccctgtccctcacctgcactgtctctggtggctccatcagcagtggtagttactactggagctggatccgccag
IGHV4-31*07     gtgaagccttcacagaccctgtccctcacctgcactgtctctggtggatccatcagcagtggtggttactactggagctggatccgccag
IGHV4-31*08     gtgaagccttcacagaccctgtccctcacctgcactgtctctggtggctccatcagcagtggtggttactactggagctggatccgccag
IGHV4-31*09     gtgaagccttcacagaccctgtccctcacctgcactgtctctggtggctccatcagcagtggtggttactactggagctggatccgccag
IGHV4-31*10     ttgaagccttcacagaccctgtccctcacctgcactgtctctggtggctccatcagcagtggtggttactactggagctggatccgccag
IGHV4-31*01     gtgaagccttcacagaccctgtccctcacctgcactgtctctggtggctccatcagcagtggtggttactactggagctggatccgccag
IGHV4-31*04     gtgaagccttcacagaccctgtccctcacctgcactgtctctggtggctccatcagcagtggtggttactactggagctggatccgccag
IGHV4-31*05     gtgaagccttcacagaccctgtccctcacctgcactgtctctggtggctccatcagcagtggtggttactactggagctggatccgccag
IGHV4-31*11     gtgaagccttcacagaccctgtccctcacctgcgctgtctctggtggctccatcagcagtggtggttactactggagctggatccgccag
IP_V4-31*p11    gtgaagccttcacagaccctgtccctcacctgcactgtctctggtggctccatcagcagtggtggttactactggagctggatccgccag
IP_V4-31*p12    gtgaagccttcacagaccctgtccctcacctgcactgtctctggtggctccatcagcagtggtggttactactggagctggatccgccag
IP_V4-31*p13    gtgaagccttcacagaccctgtccctcacctgcgctgtctctggtggctccatcagcagtggtggttactactggagctggatccgccag
V_V4-31*02      gtgaagccttcacagaccctgtccctcacctgtactgtctctggtggctccatcagcagtggtggttactactggagctggatccgccag
V_V4-31*03      gtgaagccttcacagaccctgtccctcacctgcactgtctctggtggctccatcagcagtggtggttactactggagctggatccgccag
```

```
V4-31_01       cacccagggaagggcctggagtggattgggtacatctattacagtgggagcacctactacaacccgtccctcaagagtcgagttaccatat
V4-31_02       cacccagggaagggcctggagtggattgggtacatctattacagtgggagcacctactacaacccgtccctcaagagtctagttaccatat
V4-31_03       cacccagggaagggcctggagtggattgggtacatctattacagtgggagcacctattacaacccgtccctcaagagtcgagttaccatat
V4-31_04       cacccagggaagggcctggagtggattgggtacatctattacagtgggagcacctactacaacccgtccctcaagagtcgagttaccatat
V4-31_05       cacccagggaagggcctggagtggattgggtacatctattacagtgggagcacctactacaacccgtccctcaagagtcgagttaccatat
V4-31_06       cacccagggaagggcctggagtggattgggtacatctattacagtgggagcacctactacaacccgtccctcaagagtcgagttaccatat
V4-31_07       cacccagggaagggcctggagtggattgggtacatctatcacagtgggagcacctactacaacccgtccctcaagagtcgagttaccatat
V4-31_08       cacccagggaagggcctggagtggattgggtacatctattacagtgggagcacctactacaacccgtccctcaagagtcgagttaccatat
V4-31_09       cacccagggaagggcctggagtggattgggtacatctattacagtgggagcacctactacaacccgtccctcaagagtcgagttaccatat
V4-31_10       cacccagggaagggcctggagtggattgggtacatctatcacagtgggagcacctactacaacccgtccctcaagagtcgagttaccatat
V4-31_11       cccccagggaagggcctggagtggattgggtacatctatcacagtgggagcacctactacaacccgtccctcaagagtcgagttaccatat
V4-31_12       cccgccgggaagggactggagtggattgggcatatctattccagtgggagcaccaactacaacccgtccctcaagagtcgagttaccatat
V4-31_13       cccccagggaagggcctggagtggattgggtacatctatcacagtgggagcacctactacaacccgtccctcaagagtcgagttaccatat
V4-31_14       cccgccgggaagggactggagtggattgggcgtatctattccagtgggagcaccaactacaacccgtccctcaagagtcgagttaccatat
V4-31_15       cccgccgggaagggcctggagtggattgggcatatctattccagtgggagcacctactacaacccgtccctcaagagtcgagttaccatat
V4-31_16       cacccagggaagggcctggagtggattgggtacatctattacagtgggagcacctactacaacccgtccctcaagagtcgagttaccatat
V4-31_17       cacccagggaagggcctggagtggattgggtacatctattacagtgggagcacctactacaacccgtccctcaagagtcgagttaccatat
V4-31_18       cccgccgggaagggactggagtggattgggcatatctattccagtgggagcaccaactacaacccgtccctcaagagtcgagttaccatat
V4-31_19       cacccagggaagggactggagtggattgggtacatctattacagtgggagcaccaactacaacccgtccctcaagagtcgagttaccatat
V4-31_20       cccccagggaagggactggagtggattgggtatatctattacagtgggagcaccaactacaacccgtccctcaagagtcgagttaccatat
V4-31_21       cccccagggaagggactggagtggattgggtatatctattacagtgggagcaccaactacaacccgtccctcaagagtcgagttaccatat
V4-31_22       cccccagggaagggcctggagtggattgggtacatctatcacagtgggagcacctactacaacccgtccctcaagagtcgagttaccatat
V4-31_23       cccccagggaagggcctggagtggattgggtatatctattacagtgggagcacctactacaacccgtccctcaagagtcgagttaccatat
V4-31_24       cccccagggaagggcctggagtggattgggtatatctattacagtgggagcaccaactacaacccgtccctcaagagtcgagttaccatat
V4-31_25       cacccagggaagggcctggagtggattgggtacatctatcacagtgggagcacctactacaacccgtccctcaagagtcgagttaccatat
V4-31_26       cacccagggaagggcctggagtggattgggtacatctattacagtgggagcacctactacaacccgtccctcaagagtcgagttaccatat
IGHV4-31*02    cacccagggaagggcctggagtggattgggtacatctattacagtgggagcacctactacaacccgtccctcaagagtcgagttaccatat
IGHV4-31*03    cacccagggaagggcctggagtggattgggtacatctattacagtgggagcacctactacaacccgtccctcaagagtcgagttaccatat
IGHV4-31*06    cacccagggaagggcctggagtggattgggtacatctattacagtgggagcacctactacaacccgtccctcaagagtcgagttaccatat
IGHV4-31*07    cacccagggaagggcctggagtggattgggtacatctattacagtgggagcacctactacaacccgtccctcaagagtcgagttaccatat
IGHV4-31*08    cacccagggaagggcctggagtggattgggtacatctattacagtgggagcacctactacaacccgtccctcaagagtcgagttaccatat
IGHV4-31*09    cacccagggaagggcctggagtggattgggtacatctattacagtgggagcacctactacaacccgtccctcaagagtcgagttaccatat
IGHV4-31*10    cacccagggaagggcctggagtggattgggtgcatctattacagtgggagcacctactacaacccgtccctcaagagtcgagttaccatat
IGHV4-31*01    cacccagggaagggcctggagtggattgggtacatctattacagtgggagcacctactacaacccgtccctcaagagtctagttaccatat
IGHV4-31*04    cacccagggaagggcctggagtggattgggtacatctattacagtgggagcacctactacaacccgtccctcaagagtcgagttaccatat
IGHV4-31*05    cacccagggaagggcctggagtggattgggtacatctattacagtgggagcacctactacaacccgtccctcaagagtcgagttaccatat
IGHV4-31*11    cacccagggaagggcctggagtggattgggtacatctattacagtgggagcacctactacaacccgtccctcaagagtcgagttaccatat
IP_V4-31*p11   cacccagggaagggcctggagtggattgggtacatctattacagtgggagcacctattacaacccgtccctcaagagtcgagttaccatat
IP_V4-31*p12   cacccagggaagggcctggagtggattgggtacatctattacagtgggagcacctactacaacccgtccctcaagagtcgagttaccatat
IP_V4-31*p13   cacccagggaagggcctggagtggattgggtacatctattacagtgggagcacctactacaacccgtccctcaagagtcgagttaccatat
V_V4-31*02     cacccagggaagggcctggagtggattgggtacatctattacagtgggagcacctactacaacccgtccctcaagagtcgagttaccatat
V_V4-31*03     cacccagggaagggcctggagtggattgggtacatctattacagtgggagcacctactacaacccgtccctcaagagtcgagttaccatat
```

V4-31_01	cagtagacacgtctaagaaccagttctccctgaagctgagctctgtgactgccgcggacacggccgtgtattactgtgcgaga
V4-31_02	cagtagacacgtctaagaaccagttctccctgaagctgagctctgtgactgccgcggacacggccgtgtattactgtgcgaga
V4-31_03	cagtagacacgtctaagaaccagttctccctgaagctgagctctgtgactgccgcggacacggccgtgtattactgtgcgaga
V4-31_04	cagtagacacgtctaagaaccagttctccctgaagctgagctctgtgaccgccgcggacacggccgtgtattactgtgcgaga
V4-31_05	cagtagacaggtctaagaaccagttctccctgaagctgagctctgtgaccgccgcggacacggccgtgtattactgtgcgaga
V4-31_06	cagtagacacgtccaagaaccagttctccctgaagctgagctctgtgactgccgcggacacggccgtgtattactgtgcgaga
V4-31_07	cagtagacaggtctaagaaccagttctccctgaagctgagctctgtgaccgccgcggacacggccgtgtattactgtgcgaga
V4-31_08	cagtagacacgtctaagaaccagttctccctgaagctgagctctgtgaccgccgcagacacggccgtgtattactgtgcgaga
V4-31_09	cagtagacacgtctaagaaccagttctccctgaagctgagctctgtgactgccgcggacacggccgtgtattactgtgcgaga
V4-31_10	cagtagacaggtctaagaaccagttctccctgaagctgagctctgtgaccgccgcagacacggccgtgtattactgtgcgaga
V4-31_11	cagtagacaggtctaagaaccagttctccctgaagctgagctctgtgaccgccgcagacacggccgtgtattactgtgcgaga
V4-31_12	cagtagacacgtccaagaaccagttctccctgaagctgagctctgtgaccgccgcagacacggccgtgtattactgtgcgaga
V4-31_13	cagtagacaggtctaagaaccagttctccctgaagctgagctctgtgaccgccgcggacacggccgtgtattactgtgcgaga
V4-31_14	cagtagacacgtccaagaaccagttctccctgaagctgagctctgtgaccgccgcagacacggccgtgtattactgtgcgaga
V4-31_15	cagtagacacgtctaagaaccagttctccctgaagctgagctctgtgaccgccgcagacacggccgtgtattactgtgcgaga
V4-31_16	cagtagacaggtctaagaaccagttctccctgaagctgagctctgtgactgccgcggacacggccgtgtattactgtgcgaga
V4-31_17	cagtagacacgtctaagaaccagttctccctgaagctgagctctgtgactgccgcggacacggccgtgtattactgtgcgaga
V4-31_18	cagtagacacgtccaagaaccagttctccctgaagctgagctctgtgaccgccgcagacacggccgtgtattactgtgcgaga
V4-31_19	cagtagacacgtccaagaaccagttctccctgaagctgagctctgtgactgccgcggacacggccgtgtattactgtgcgaga
V4-31_20	cagtagacacgtccaagaaccagttctccctgaagctgagctctgtgaccgccgcagacacggccgtgtattactgtgcgaga
V4-31_21	cagtagacacgtccaagaaccagttctccctgaagctgagctctgtgaccgccgcagacacggccgtgtattactgtgcgaga
V4-31_22	cagtagacaggtctaagaaccagttctccctgaagctgagctctgtgaccgccgcggacacggccgtgtattactgtgcgaga
V4-31_23	cagtagacacgtctaagaaccagttctccctgaagctgagctctgtgaccgccgcagacacggccgtgtattactgtgcgaga
V4-31_24	cagtagacacgtccaagaaccagttctccctgaagctgagctctgtgaccgccgcagacacggccgtgtattactgtgcgaga
V4-31_25	cagtagacaggtccaagaaccagttctccctgaagctgagctctgtgaccgccgcagacacggccgtgtattactgtgcgaga
V4-31_26	cagtagacacgtctaagaaccagttctccctgaagctgagctctgtgactgccgcggacacggccgtgtattactgtgcgaga
IGHV4-31*02	cagtagacacgtctaagaaccagttctccctgaagctgagctctgtgactgccgcggacacggccgtgtattactgtgcgaga
IGHV4-31*03	cagtagacacgtctaagaaccagttctccctgaagctgagctctgtgactgccgcggacacggccgtgtattactgtgcgaga
IGHV4-31*06	cagtagacacgtctaagaaccagttctccctgaagctgagctctgtgactgccgcggacacggccgtgtattactg-------
IGHV4-31*07	cagtagacacgtctaagaaccagttctccctgaagctgagctctgtgactgccgcggacacggccgtgtattactg-------
IGHV4-31*08	ccgtagacacgtccaagaaccagttctccctgaagctgagctctgtgactgccgcggacacggccgtgtattactg-------
IGHV4-31*09	cagtagacaagtccaagaaccagttctccctgaagctgagctctgtgaccgccgcggacacggccgtgtattactg-------
IGHV4-31*10	cagtagacccgtccaagaaccagttctccctgaagccgagctctgtgactgccgcggacacggccgtggattactgtgcgaga
IGHV4-31*01	cagtagacacgtctaagaaccagttctccctgaagctgagctctgtgactgccgcggacacggccgtgtattactgtgcgaga
IGHV4-31*04	cagtagacacgtctaagaaccagttctccctgaagctgagctctgtgactgccgcggacacggccgtgtattactgtgcg---
IGHV4-31*05	cagtagacacgtctaagaaccagttctccctgaagctgagctctgtga---ccgcggacgcggccgtgtattactgtgcg---
IGHV4-31*11	cagtagacacgtctaagaaccagttctccctgaagctgagctctgtgactgccgcggacacggccgtgtattactgtgcgaga
IP_V4-31*p11	cagtagacacgtctaagaaccagttctccctgaagctgagctctgtgactgccgcggacacggccgtgtattactgtgcgaga
IP_V4-31*p12	cagtagacacgtccaagaaccagttctccctgaagctgagctctgtgactgccgcagacacggccgtgtattactgtgccaga
IP_V4-31*p13	cagtagacacgtctaagaaccagttctccctgaagctgagctctgtgactgccgcggacacggccgtgtattactgtgcgaga
V_V4-31*02	cagtagacacgtctaagaaccagttctccctgaagctgagctctgtgactgccgcggacacggccgtgtattactgtgcgaga
V_V4-31*03	cagtagacacgtctaagaaccagttctccctgaagctgagctctgtgactgccgcggacacggccgtgtattactgtgcgaga

IGHV4-34

V4-34_01	atgaaacacctgtggttcttcctcctcctggtggcagctcccagatgggtcctgtcccaggtgcagctacagcagtggggcgcaggactgt
V4-34_02	atgaaacacctgtggttcttcctcctcctggtggcagctcccagatgggtcctgtcccaggtgcagctacagcagtggggcgcaggactgt
V4-34_03	atgaaacacctgtggttcttcctcctcctggtggcagctcccagatgggtcctgtcccaggtacagctacagcagtggggcgcaggactgt
IGHV4-34*01	atgaaacacctgtggttcttcctcctcctggtggcagctcccagatgggtcctgtcccaggtgcagctacagcagtggggcgcaggactgt
IGHV4-34*02	atgaaacacctgtggttcttcctcctcctggtggcagctcccagatgggtcctgtcccaggtgcagctacaacagtggggcgcaggactgt
IGHV4-34*03	atgaaacacctgtggttcttcctcctcctggtggcagctcccagatgggtcctgtcccaggtgcagctacagcagtggggcgcaggactgt
IGHV4-34*04	atgaaacacctgtggttcttcctcctcctggtggcagctcccagatgggtcctgtcccaggtgcagctacagcagtggggcgcaggactgt
IGHV4-34*05	atgaaacacctgtggttcttcctgctcctggtggcagctcccagatgggtcctgtcccaggtgcagctacagcagtggggcgcaggactgt
IGHV4-34*06	atgaaacacctgtggttcttcctcctcctggtggcagctcccagatgggtcctgtcccaggtgcagctacagcagtggggcgcaggactgt
IGHV4-34*07	atgaaacacctgtggttcttcctcctcctggtggcagctcccagatgggtcctgtcccaggtgcagctacagcagtggggcgcaggactgt
IGHV4-34*09	atgaaacacctgtggttcttcctcctgctggtggcagctcccagatgggtcctgtcccaggtgcagctgcaggagtcgggcccaggactgg
IGHV4-34*10	atgaaacacctgtggttcttcctcctcctggtggcagctcccagatgggtcctgtcccaggtgcagctgcaggagtcgggcccaggactgg
IGHV4-34*11	atgaaacacctgtggttcttcctcctcctggtggcagctcccagatgggtcctgtcccaggtgcagctacagcagtggggcgcaggactgt
IP_V4-34*p14	--caggtgcagctacagcagtggggcgcaggactgt
IP_V4-34*p15	--caggtgcagctacagcagtggggcgcaggactgt
IP_V4-34*p16	--caggtgcagctacagcagtggggcgcaggactgt
IP_V4-34*p17	--caggtgcagctgcaggagtcgggcccaggactgg
V_V4-34*01	--caggtgcagctacagcagtggggcgcaggactgt
V_V4-34*02	--caggtgcagctacaacagtggggcgcaggactgt
V_V4-34*11	--caggtgcagctacagcagtggggcgcaggactgt

V4-34_01	tgaagccttcggagaccctgtccctcacctgcgctgtctatggtgggtccttcagtggttactactggagctggatccgccagccccca
V4-34_02	tgaagccttcggagaccctgtccctcacctgcgctgtctatggtgggtccttcagtggttactactggagctggatccgccagccccca
V4-34_03	tgaagccttcggagaccctgtccctcacctgcgctgtctatggtgggtccttcagtggttactactggagctggatccgccagccccca
IGHV4-34*01	tgaagccttcggagaccctgtccctcacctgcgctgtctatggtgggtccttcagtggttactactggagctggatccgccagccccca
IGHV4-34*02	tgaagccttcggagaccctgtccctcacctgcgctgtctatggtgggtccttcagtggttactactggagctggatccgccagccccca
IGHV4-34*03	tgaagccttcggagaccctgtccctcacctgcgctgtctatggtgggtccttcagtggttactactggagctggatccgccagccccca
IGHV4-34*04	tgaagccttcggagaccctgtccctcacctgcgctgtctatggtgggtccttcagtggttactactggagctggatccgccagccccca
IGHV4-34*05	tgaagccttcggagaccctgtccctcacctgcgctgtctatggtgggtccttcagtggttactactggtgctggatccgccagcccccta
IGHV4-34*06	tgaagccttcggagaccctgtccctcacctgcgctgtctatggtgggtccttcagtggttactactggagctggatccgccagccccca
IGHV4-34*07	tgaagccttcggagaccctgtccctcacctgcgctgtctatggtgggtccttcagtggttactactggagctggatccgccagccccca
IGHV4-34*09	tgaagccttcacagaccctgtccctcacctgcgctgtctatggtgggtccttcagtggttactactggagctggatccgccagccccca
IGHV4-34*10	tgaagccttcggagaccctgtccctcacctgcgctgtctatggtgggtccttcagtggttactactggagctggatccgccagccccca
IGHV4-34*11	tgaagccttcggagaccctgtccctcacctgcgctgtctatggtgggtccgtcagtggttactactggagctggatccggcagccccca
IP_V4-34*p14	tgaagccttcggagaccctgtccctcacctgcgctgtctatggtgggtccttcagtggttactactggagctggatccgccagccccca
IP_V4-34*p15	tgaagccttcggagaccctgtccctcacctgcgctgtctatggtgggtccttcagtggttactactggagctggatccgccagccccca
IP_V4-34*p16	tgaagccttcggagaccctgtccctcacctgcgctgtctatggtgggtccttcagtggttactactggagctggatccgccagccccccg
IP_V4-34*p17	tgaagccttcggagaccctgtccctcacctgcgctgtctatggtgggtccttcagtggttactactggagctggatccgccagccccca
V_V4-34*01	tgaagccttcggagaccctgtccctcacctgcgctgtctatggtgggtccttcagtggttactactggagctggatccgccagccccca
V_V4-34*02	tgaagccttcggagaccctgtccctcacctgcgctgtctatggtgggtccttcagtggttactactggagctggatccgccagccccca
V_V4-34*11	tgaagccttcggagaccctgtccctcacctgcgctgtctatggtgggtccgtcagtggttactactggagctggatccggcagccccca

```
V4-34_01        gggaaggggctggagtggattggggaaatcaatcatagtggaagcaccaactacaacccgtccctcaagagtcgagtcaccatatcagtag
V4-34_02        gggaaggggctggagtggattggggaaatcaatcatagtggaagcaccaactacaacccgtccctcaagagtcgagtcaccatatcagtag
V4-34_03        gggaaggggctggagtggattggggaaatcaatcatagtggaagcaccaactacaacccgtccctcaagagtcgagtcaccgtatcagtag
IGHV4-34*01     gggaaggggctggagtggattggggaaatcaatcatagtggaagcaccaactacaacccgtccctcaagagtcgagtcaccatatcagtag
IGHV4-34*02     gggaaggggctggagtggattggggaaatcaatcatagtggaagcaccaactacaacccgtccctcaagagtcgagtcaccatatcagtag
IGHV4-34*03     gggaaggggctggagtggattggggaaatcaatcatagtggaagcaccaactacaacccgtccctcaagagtcgagtcaccatatcagtag
IGHV4-34*04     gggaaggggctggagtggattggggaaatcaatcatagtggaagcaccaacaacaacccgtccctcaagagtcgagccaccatatcagtag
IGHV4-34*05     gggaaggggctggagtggattggggaaatcaatcatagtggaagcaccaacaacaacccgtccctcaagagtcgagccaccatatcagtag
IGHV4-34*06     gggaaggggctggagtggattggggaaatcaatcatagtggaagcaccaactacaacccgtccctcaagagtcgagtcaccatatcagtag
IGHV4-34*07     gggaaggggctggagtggattggggaaatcaaccatagtggaagcaccaactacaacccgtccctcaagagtcgagtcaccatatcagtag
IGHV4-34*09     gggaagggactggagtggattggggaaatcaatcatagtggaagcaccaactacaacccgtccctcaagagtcgagttaccatatcagtag
IGHV4-34*10     gggaagggactggagtggattggggaaatcaatcatagtggaagcaccaactacaacccgtccctcaagagtcgaatcaccatgtcagtag
IGHV4-34*11     gggaaggggctggagtggattgggtatatctattatagtgggagcaccaacaacaacccctccctcaagagtcgagccaccatatcagtag
IP_V4-34*p14    gggaaggggctggagtggattggggaaatcaatcatagtggaagcaccaactacaacccgtccctcaagagtcgagtcaccatatcagtag
IP_V4-34*p15    gggaaggggctgcagtggattggggaaatcaatcatagtggaagcaccaactacaacccgtccctcaagagtcgagtcaccatatcagtag
IP_V4-34*p16    gggaaggggctggagtggattggggaaatcaatcatagtggaagcaccaactacaacccgtccctcaagagtcgagtcaccatatcagtag
IP_V4-34*p17    gggaaggggctggagtggattggggaaatcaatcatagtggaagcaccaactacaacccgtccctcaagagtcgagtcaccatatcagtag
V_V4-34*01      gggaaggggctggagtggattggggaaatcaatcatagtggaagcaccaactacaacccgtccctcaagagtcgagtcaccatatcagtag
V_V4-34*02      gggaaggggctggagtggattggggaaatcaatcatagtggaagcaccaactacaacccgtccctcaagagtcgagtcaccatatcagtag
V_V4-34*11      gggaaggggctggagtggattgggtatatctattatagtgggagcaccaacaacaacccctccctcaagagtcgagccaccatatcagtag

V4-34_01        acacgtccaagaaccagttctccctgaagctgagctctgtgaccgccgcggacacggctgtgtattactgtgcgaga
V4-34_02        acacgtccaagaaccagttctccctgaagctgagctctgtgaccgccgcggatacggctgtgtattactgtgcgaga
V4-34_03        acacgtccaagaaccagttctccctgaagctgagctctgtgaccgccgcggacacggctgtgtattactgtgcgaga
IGHV4-34*01     acacgtccaagaaccagttctccctgaagctgagctctgtgaccgccgcggacacggctgtgtattactgtgcgaga
IGHV4-34*02     acacgtccaagaaccagttctccctgaagctgagctctgtgaccgccgcggacacggctgtgtattactgtgcgaga
IGHV4-34*03     acacgtccaagaaccagttctccctgaagctgagctctgtgaccgccgcggacacggccgtgtattactg-------
IGHV4-34*04     acacgtccaagaaccagttctccctgaagctgagctctgtgaccgccgcggacacggctgtgtattactgtgcgaga
IGHV4-34*05     acacgtccaagaaccagttctccctgaagctgagctctgtgaccgccgcggacacggctgtgtattactgtgcgaga
IGHV4-34*06     acacgtccaagaaccagttctccctgaagctgggctctgtgaccgccgcggacacggccgtgtattactg-------
IGHV4-34*07     acacgtccaagaaccagttctccctgaagctgagctctgtgaccgccgcggacacggccgtgtattactg-------
IGHV4-34*09     acacgtctaagaaccagttctccctgaagctgagctctgtgactgccgcggacacggccgtgtattactgtgcgaga
IGHV4-34*10     acacgtccaagaaccagttctacctgaagctgagctctgtgaccgccgcggacacggccgtgtattactgtgcgaga
IGHV4-34*11     acacgtccaagaaccagttctccctgaacctgagctctgtgaccgccgcggacacggccgtgtattgctgtgcgaga
IP_V4-34*p14    acacgtccaagaaccagttctccctgaagctgagctctgtgaccgccgcggacacggccgtgtattactgtgcgaga
IP_V4-34*p15    acacgtccaagaaccagttctccctgaagctgagctctgtgaccgccgcggacacggctgtgtattactgtgcgaga
IP_V4-34*p16    acacgtccaagaaccagttctccctgaagctgagctctgtgaccgccgcggacacggctgtgtattactgtgcgaga
IP_V4-34*p17    acacgtccaagaaccagttctccctgaagctgagctctgtgaccgccgcggacacggctgtgtattactgtgcgaga
V_V4-34*01      acacgtccaagaaccagttctccctgaagctgagctctgtgaccgccgcggacacggctgtgtattactgtgcgaga
V_V4-34*02      acacgtccaagaaccagttctccctgaagctgagctctgtgaccgccgcggacacggctgtgtattactgtgcgaga
V_V4-34*11      acacgtccaagaaccagttctccctgaacctgagctctgtgaccgccgcggacacggccgtgtattgctgtgcgaga
```

IGHV4-39

```
V4-39_01        atgaagcacctgtggttcttcctcctgctggtggcggctcccagatgggtcctgtcccagctgcagctgcaggagtcgggcccaggactg
V4-39_02        atgaagcacctgtggttcttcctcctgctggtggcggctcccagatgggtcctgtcccagctgcagctgcaggagtcgggcccaggactg
V4-39_03        atgaagcacctgtggttcttcctcctgctggtggcggctcccagatgggtcctgtcccagctgcagctgcaggagtcgggcccaggactg
V4-39_04        atgaagcacctgtggttcttcctcctgctggtggcggctcccagatgggtcctgtcccagctgcagctgcaggagtcgggcccaggactg
V4-39_06        atgaagcacctgtggttcttcctcctgctggtggcggctcccagatgggtcctgtcccagctgcagctgcaggagtcgggcccaggactg
IGHV4-39*01     atgaagcacctgtggttcttcctcctgctggtggcggctcccagatgggtcctgtcccagctgcagctgcaggagtcgggcccaggactg
IGHV4-39*02     atgaagcacctgtggttcttcctcctgctggtggcggctcccagatgggtcctgtcccagctgcagctgcaggagtcgggcccaggactg
IGHV4-39*03     atgaaacacctgtggttcttcctcctgctggtggcggctcccagatgggtcctgtcccagctgcagctgcaggagtcgggcccaggactg
IGHV4-39*06     atgaaacacctgtggttcttcctcctgctggtggcggctcccagatgggtcctgtcccggctgcagctgcaggagtcgggcccaggactg
IGHV4-39*04     ------------------------------------------------------------------------------------------
IGHV4-39*05     ---------------------------------------------------------cagctgcagctgcaggagtcgggcccaggactg
IGHV4-39*07     ---------------------------------------------------------cagctgcagctgcaggagtcgggcccaggactg
IP_V4-39*p08    ------------------------------------------------------------------------------------------
IP_V4-39*p09    ---------------------------------------------------------caggtgcagctgcaggagtcgggcccaggactg
IP_V4-39*p10    ---------------------------------------------------------caggtgcagctgcaggagtcgggcccaggactg
IP_V4-39*p11    ---------------------------------------------------------cagctgcagctgcaggagtcgggcccaggactg
IP_V4-39*p12    ---------------------------------------------------------caggtgcagctgcaggagtcgggcccaggactg
V_V4-39*01      ---------------------------------------------------------cagctgcagctgcaggagtcgggcccaggactg
V_V4-39*02      ---------------------------------------------------------cagctgcagctgcaggagtcgggcccaggactg
```

```
V4-39_01        gtgaagccttcggagaccctgtccctcacctgcactgtctctggtggctccatcagcagtagtagttactactggggctggatccgccag
V4-39_02        gtgaagccttcggagaccctgtccctcacctgcactgtctctggtggctccatcagcagtagtagttactactggggctggatccgccag
V4-39_03        gtgaagccttcggagaccctgtccctcacctgcactgtctctggtggctccatcagcagtagtagttactactggggctggatccgccag
V4-39_04        gtgaagccttcggagaccctgtccctcacctgcactgtctctggtggctccatcagcagtagtagttactactggggctggatccgccag
V4-39_06        gtgaagccttcggagaccctgtccctcacctgcactgtctctggtggctccatcagcagtagtagttactactggggctggatccgccag
IGHV4-39*01     gtgaagccttcggagaccctgtccctcacctgcactgtctctggtggctccatcagcagtagtagttactactggggctggatccgccag
IGHV4-39*02     gtgaagccttcggagaccctgtccctcacctgcactgtctctggtggctccatcagcagtagtagttactactggggctggatccgccag
IGHV4-39*03     gtgaagccttcggagaccctgtccctcacctgcactgtctctggtggctccatcagcagtagtagttactactggggctggatccgccag
IGHV4-39*06     gtgaagccttcggagaccctgtccctcacctgcactgtctctggtggctccatcagcagtagtagttactactggggctggatccgccag
IGHV4-39*04     ----------------------------------------------gctccatcagcagtagtagttactactggggctggatccgccag
IGHV4-39*05     gtgaagccttcggagaccccgtccctcacctgcactgtctctggtggctccatcagcagtagtagttactactggggctggatccgccag
IGHV4-39*07     gtgaagccttcggagaccctgtccctcacctgcactgtctctggtggctccatcagcagtagtagttactactggggctggatccgccag
IP_V4-39*p08    ------------------------------------------------------------------------------------------
IP_V4-39*p09    gtgaagccttcggagaccctgtccctcacctgcactgtctctggtggctccatcagcagtagtagttactactggggctggatccgccag
IP_V4-39*p10    gtgaagccttcggagaccctgtccctcacctgcactgtctctggtggctccatcagcagtagtagttactactggggctggatccgccag
IP_V4-39*p11    gtgaagccttcggagaccctgtccctcacctgcactgtctctggtggctccatcagcagtagtagttactactggggctggatccgccag
IP_V4-39*p12    gtgaagccttcggagaccctgtccctcacctgcactgtctctggtggctccatcagcagtagtagttactactggggctggatccgccag
V_V4-39*01      gtgaagccttcggagaccctgtccctcacctgcactgtctctggtggctccatcagcagtagtagttactactggggctggatccgccag
V_V4-39*02      gtgaagccttcggagaccctgtccctcacctgcactgtctctggtggctccatcagcagtagtagttactactggggctggatccgccag
```

```
V4-39_01        cccccagggaaggggctggagtggattgggagtatctattatagtgggagcacctactacaacccgtccctcaagagtcgagtcaccata
V4-39_02        cccccagggaaggggctggagtggattgggagtatctattatagtgggagcacctactacaacccgtccctcaagagtcgagtcaccata
V4-39_03        cccccagggaaggggctggagtggattgggagtatctattatagtgggagcacctactacaacccgtccctcaagagtcgagtcaccata
V4-39_04        cccccagggaaggggctggagtggattgggagtatctattatagtgggagcacctactacaacccgtccctcaagagtcgagtcaccata
V4-39_06        cccccagggaaggggctggagtggattgggagtatctattatagtgggagcacctactacaacccgtccctcaagagtcgagtcaccata
IGHV4-39*01     cccccagggaaggggctggagtggattgggagtatctattatagtgggagcacctactacaacccgtccctcaagagtcgagtcaccata
IGHV4-39*02     cccccagggaaggggctggagtggattgggagtatctattatagtgggagcacctactacaacccgtccctcaagagtcgagtcaccata
IGHV4-39*03     cccccagggaaggggctggagtggattgggagtatctattatagtgggagcacctactacaacccgtccctcaagagtcgagtcaccata
IGHV4-39*06     cccccagggaaggggctggagtggattgggagtatctattatagtgggagcacctactacaacccgtccctcaagagtcgagtcaccata
IGHV4-39*04     cccccagggaaggggctggagtggattgggagtatctattatagtgggagcacctactacaacccgtccctcaagagtcgagtcaccata
IGHV4-39*05     cccccagggaaggggctggagtggattgggagtatctattatagtgggagcacctactacaacccgtccctcaagagtcgagtcaccata
IGHV4-39*07     cccccagggaaggggctggagtggattgggagtatctattatagtgggagcacctactacaacccgtccctcaagagtcgagtcaccata
IP_V4-39*p08    ------------gggctggagtggattgggagtatctattatagtgggagcacctactacaacccgtccctcaagagtcgagtcaccata
IP_V4-39*p09    cccccagggaaggggctggagtggattgggagtatctattatagtgggagcacctactacaacccgtccctcaagagtcgagtcaccata
IP_V4-39*p10    cccccagggaaggggctggagtggattgggagtatctattatagtgggagcacctactacaacccgtccctcaagagtcgagtcaccata
IP_V4-39*p11    cccccagggaaggggctggagtggattgggagtatctattatagtgggagcacctactacaacccgtccctcaagagtcgagtcaccata
IP_V4-39*p12    cccccagggaaggggctggagtggattgggagtatctattatagtgggagcacctactacaacccgtccctcaagagtcgagtcaccata
V_V4-39*01      cccccagggaaggggctggagtggattgggagtatctattatagtgggagcacctactacaacccgtccctcaagagtcgagtcaccata
V_V4-39*02      cccccagggaaggggctggagtggattgggagtatctattatagtgggagcacctactacaacccgtccctcaagagtcgagtcaccata

V4-39_01        tccgtagacacgtccaagaaccagttctccctgaagctgagctctgtgaccgccgcagacacggctgtgtattactgtgcgaga
V4-39_02        tccgtagacacgtccaagaaccagttctccctgaagctgagctctgtgaccgccgcggacacggctgtgtattactgtgcgaga
V4-39_03        tcagtagacacgtccaagaaccagttctccctgaagctgagctctgtgaccgccgcggacacggctgtgtattactgtgcgaga
V4-39_04        tcagtagacacgtccaagaaccagttctccctgaagctgagctctgtgaccgccgcagacacggctgtgtattactgtgcgaga
V4-39_06        tcagtagacacgtccaagaaccagttctccctgaagctgagctctgtgaccgcagcggacacggctgtgtattactgtgcgaga
IGHV4-39*01     tccgtagacacgtccaagaaccagttctccctgaagctgagctctgtgaccgccgcagacacggctgtgtattactgtgcgaga
IGHV4-39*02     tccgtagacacgtccaagaaccacttctccctgaagctgagctctgtgaccgccgcagacacggctgtgtattactgtgcgaga
IGHV4-39*03     tccgtagacacgtccaagaaccagttctccctgaagctgagctctgtgaccgccgcagacacggccgtgtattactg-------
IGHV4-39*06     tcagtagacacgtccaagaaccagttccccctgaagctgagctctgtgaccgccgcggacacggccgtgtattactgtgcgaga
IGHV4-39*04     tccgtagacacgtccaagaaccagttctccctgaagctgagctctgtgaccgccgcggacac----------------------
IGHV4-39*05     tccgtagacacgtccaagaaccagttctccctgaagctgagctctgtgaccgccgcagacacggctgtgtattactgtgcg---
IGHV4-39*07     tcagtagacacgtccaagaaccagttctccctgaagctgagctctgtgaccgccgcggacacggccgtgtattactgtgcgaga
IP_V4-39*p08    tcagtagacacgtccaagaaccagttctccctgaagctgagctctgtgaccgcagcggacacggccgtgtattactgtgcgaga
IP_V4-39*p09    tccgtagacacgtccaagaaccagttctccctgaagctgagctctgtgaccgccgcagacacggctgtgtattactgtgcgaga
IP_V4-39*p10    tcagtagacacgtccaagaaccagttctccctgaagctgagctctgtgaccgccgcggacacggccgtgtattactgtgcgaga
IP_V4-39*p11    tcagtagacacgtccaagaaccagttctccctgaagctgagctctgtgaccgccgcggacacggccgtgtattactgtgcgaga
IP_V4-39*p12    tcagtagacacgtccaagaaccagttctccctgaagctgagctctgtgaccgccgcggacacggccgtgtattactgtgcgaga
V_V4-39*01      tccgtagacacgtccaagaaccagttctccctgaagctgagctctgtgaccgccgcagacacggctgtgtattactgtgcgaga
V_V4-39*02      tccgtagacacgtccaagaaccacttctccctgaagctgagctctgtgaccgccgcagacacggctgtgtattactgtgcgaga
```

IGHV4-59

V4-59_01	atgaaacatctgtggttcttccttctcctggtggcagctcccagatgggtcctgtcccaggtgcagctgcaggagtcgggcccaggactg
V4-59_02	atgaaacatctgtggttcttccttctcctggtggcagctcccagatgggtcctgtcccaggtgcagctgcaggagtcgggcccaggactg
V4-59_03	atgaaacatctgtggttcttcctcctcctggtggcagctcccagatgggtcctgtcccaggtgcagctgcaggagtcgggcccaggactg
V4-59_04	atgaaacatctgtggttcttccttctcctggtggcagctcccagatgggtcctgtcccaggtgcagctgcaggagtcgggcccaggactg
V4-59_05	atgaaacatctgtggttcttccttctcctggtggcagctcccagatgggtcctgtcccaggtgcagctgcaggagtcgggcccaggactg
V4-59_06	atgaaacatctgtggttcttccttctcctggtggcagctcccagatgggtcctgtcccaggtgcagctgcaggagtcgggcccaggactg
V4-59_07	atgaaacatctgtggttcttccttctcctggtggcagctcccagatgggtcctgtcccaggtgcagctgcaggagtcgggcccaggactg
V4-59_08	atgaaacatctgtggttcttccttctcctggtggcagctcccagatgggtcctgtcccaggtgcagctgcaggagtcgggcccaggactg
V4-59_09	atgaaacatctgtggttcttccttctcctggtggcagctcccagatgggtcctgtcccaggtgcagctgcaggagtcgggcccaggactg
V4-59_10	atgaaacatctgtggttcttccttctcctggtggcagctcccagatgggtcctgtcccaggtgcagctgcaggagtcgggcccaggactg
V4-59_11	atgaaacatctgtggttcttccttctcctggtggcagctcccagatgggtcctgtcccaggtgcagctgcaggagtcgggcccaggactg
V4-59_12	atgaaacatctgtggttcttccttctcctggtggcagctcccagatgggtcctgtcccaggtgcagctgcaggagtcgggcccaggactg
V4-59_13	atgaaacatctgtggttcttccttctcctggtggcagctcccagatgggtcctgtcccaggtgcagctgcaggagtcgggcccaggactg
IGHV4-59*01	atgaaacatctgtggttcttccttctcctggtggcagctcccagatgggtcctgtcccaggtgcagctgcaggagtcgggcccaggactg
IGHV4-59*02	atgaaacatctgtggttcttccttctcctggtggcagctcccagatgggtcctgtcccaggtgcagctgcaggagtcgggcccaggactg
IGHV4-59*10	atgaaacacctgtggttcttcctcctcctggtggcagctcccagatgggtcctgtcccaggtgcagctacagcagtggggcgcaggactg
IGHV4-59*11	atgaaacatctgtggttcttccttctcctggtggcagctcccagatgggtcctgtcccaggtgcagctgcaggagtcgggcccaggactg
IGHV4-59*03	---caggtgcagctgcaggagtcgggcccaggactg
IGHV4-59*04	---caggtgcagctgcaggagtcgggcccaggactg
IGHV4-59*05	---caggtgcagctgcaggagtcgggcccaggactg
IGHV4-59*06	---caggtgcagctgcaggagtcgggcccaggactg
IGHV4-59*07	---caggtgcagctgcaggagtcgggcccaggactg
IGHV4-59*08	---caggtgcagctgcaggagtcgggcccaggactg
IGHV4-59*09	--
IGHV4-59*12	---caggtgcagctgcaggagtcgggcccaggactg
IP_V4-59*p11	--
IP_V4-59*p12	--
IP_V4-59*p13	--
IP_V4-59*p14	---caggtgcagctgcaggagtcgggcccaggactg
IP_V4-59*p15	---caggtgcagctgcaggagtcgggcccaggactg
IP_V4-59*p16	---caggtgcagctgcaggagtcgggcccaggactg
IP_V4-59*p17	---caggtgcagctgcaggagtcgggcccaggactg
IP_V4-59*p18	---caggtgcagctgcaggagtcgggcccaggactg
IP_V4-59*p19	--aggtgcagctgcaggagtccggctcaggactg
IP_V4-59*p20	---caggtgcagctgcaggagtcgggcccaggactg
IP_V4-59*p21	---caggtgcagctgcaggagtcgggcccaggactg
IP_V4-59*p22	---caggtgcagctgcaggagtcgggcccaggactg
V_V4-59*01	---caggtgcagctgcaggagtcgggcccaggactg
V_V4-59*02	---caggtgcagctgcaggagtcgggcccaggactg

V4-59_01	gtgaagccttcggagaccctgtccctcacctgcactgtctctggtggctccatcagtagttactactggagctggatccggcagccccca
V4-59_02	gtgaagccttcggagaccctgtccctcacctgcactgtctctggtggctccatcagtagttactactggagctggatccggcagccccca
V4-59_03	gtgaagccttcggagaccctgtccctcacctgcactgtctctggtggctccatcagtagttactactggagctggatccggcagccccca
V4-59_04	gtgaagccttcggagaccctgtccctcacctgcactgtctctggtggctccatcagtagttactactggagctggatccggcagcccccg
V4-59_05	gtgaagccttcggagaccctgtccctcacctgcactgtctctggtggctccatcagtagtcactactggagctggatccggcagccccca
V4-59_06	gtgaagccttcacagaccctgtccctcacctgcactgtctctggtggctccatcagtagttactactggagctggatccggcagccccca
V4-59_07	gtgaagccttcggagaccctgtccctcacctgcactgtctctggtggctccatcagtagtcactactggagctggatccggcagccccca
V4-59_08	gtgaagccttcacagaccctgtccctcacctgcactgtctctggtggctccatcagtagtcactactggagctggatccggcagccccca
V4-59_09	gtgaagccttcggagaccctgtccctcacctgcactgtctctggtggctccatcagtagtcactactggagctggatccggcagccccca
V4-59_10	gtgaagccttcggagaccctgtccctcacctgcactgtctctggtggctccatcagtagttactactggagctggatccggcagcccccg
V4-59_11	gtgaagccttcggagaccctgtccctcacctgcactgtctctggtggctccatcagtagtcactactggagctggatccggcagccccca
V4-59_12	gtgaagccttcggagaccctgtccctcacctgcactgtctctggtggctccatcagtagttactactggagctggatccggcagccccca
V4-59_13	gtgaagccttcggagaccctgtccctcacctgcactgtctctggtggctccatcagtagttactactggagctggatccggcagccccca
IGHV4-59*01	gtgaagccttcggagaccctgtccctcacctgcactgtctctggtggctccatcagtagttactactggagctggatccggcagccccca
IGHV4-59*02	gtgaagccttcggagaccctgtccctcacctgcactgtctctggtggctccgtcagtagttactactggagctggatccggcagccccca
IGHV4-59*10	ttgaagccttcggagaccctgtccctcacctgcgctgtctatggtggctccatcagtagttactactggagctggatccggcagcccgcc
IGHV4-59*11	gtgaagccttcggagaccctgtccctcacctgcactgtctctggtggctccatcagtagtcactactggagctggatccggcagccccca
IGHV4-59*03	gtgaagccttcggagaccctgtccctcacctgcactgtctctggtggctccatcagtagttactactggagctggatccggcagccccca
IGHV4-59*04	gtgaagccttcggagaccctgtccctcacctgcactgtctctggtggctccatcagtagttactactggagctggatccggcagccccca
IGHV4-59*05	gtgaagccttcggagaccctgtccctcacctgcactgtctctggtggctccatcagtagttactactggagctggatccggcagccgccg
IGHV4-59*06	gtgaagccttcggagaccctgtccctcacctgcactgtcactggtggctccatcagtagttactactggagctggatccggcagcccgct
IGHV4-59*07	gtgaagccttcggacaccctgtccctcacctgcactgtctctggtggctccatcagtagttactactggagctggatccggcagccccca
IGHV4-59*08	gtgaagccttcggagaccctgtccctcacctgcactgtctctggtggctccatcagtagttactactggagctggatccggcagccccca
IGHV4-59*09	---------------------------------------tctggtggctccatcagtagttactactggagctggatccggcagccccca
IGHV4-59*12	gtgaagccttcggagaccctgtccctcacctgcactgtctctggtggctccatcagtagttactactggagctggatccggcagccccca
IP_V4-59*p11	--
IP_V4-59*p12	--------------------------------cactgtctctggtggctccatcagtagtcactactggagctggatccggcagccccca
IP_V4-59*p13	--------------------------------cactgtctctggtggctccatcagtagttactactggagctggatccggcagcccccg
IP_V4-59*p14	gtgaagccttcggagaccctgtccctcacctgcactgtctctggtggctccatcagtagttactactggagctggatccggcagccccca
IP_V4-59*p15	gtgaagccttcggagaccctgtccctcacctgcactgtctctggtggctccatcagtagtcactactggagctggatccggcagccccca
IP_V4-59*p16	gtgaagccttcggagaccctgtccctcacctgcactgtctctggtggctccatcagtagttactactggagctggatccggcagccccca
IP_V4-59*p17	gtgaagccttcggagaccctgtccctcacctgcactgtctctggtggctccatcagtagttactactggagctggatccggcagcccccg
IP_V4-59*p18	gtgaagccttcggagaccctgtccctcacctgcactgtctctggtggctccatcagtagttactactggagctggatccggcagccccca
IP_V4-59*p19	gtgaagccttcggagaccctgtccctcacctgcactgtctctggtggctccatcagtagttactactggagctggatccggcagccccca
IP_V4-59*p20	gtgaagccttcggagaccctgtccctcacctgcactgtctctggtggctccatcagtagttactactggagctggatccggcagccccca
IP_V4-59*p21	gtgaagccttcggagaccctgtccctcacctgcactgtctctggtggctccatcagtagttactactggagctggatccggcagccccca
IP_V4-59*p22	gtgaagccttcggagaccctgtccctcacctgcactgtctctggtggctccatcagtagttactactggagctggatccggcagccccca
V_V4-59*01	gtgaagccttcggagaccctgtccctcacctgcactgtctctggtggctccatcagtagttactactggagctggatccggcagccccca
V_V4-59*02	gtgaagccttcggagaccctgtccctcacctgcactgtctctggtggctccgtcagtagttactactggagctggatccggcagccccca

V4-59_01	gggaagggactggagtggattgggtatatctattacagtgggagcaccaactacaacccctccctcaagagtcgagtcaccatatcagtaga
V4-59_02	gggaagggactggagtggattgggtatatctattacagtgggagcaccaactacaacccctccctcaagagtcgagtcaccatatcagtaga
V4-59_03	gggaagggactggagtggattgggtatatctattacagtgggagcaccaactacaacccctccctcaagagtcgagtcaccatatcagtaga
V4-59_04	gggaagggactggagtggattgggtatatctattacagtgggagcaccaactacaacccctccctcaagagtcgagtcaccatatcagtaga
V4-59_05	gggaagggactggagtggattgggtatatctattacagtgggagcaccaactacaacccctccctcaagagtcgagtcaccatatcagtaga
V4-59_06	gggaagggactggagtggattgggtatatctattacagtgggagcaccaactacaacccctccctcaagagtcgagtcaccatatcagtaga
V4-59_07	gggaagggactggagtggattgggtacatctataccagtgggagcaccaactacaacccctccctcaagagtcgagtcaccatatcagtaga
V4-59_08	gggaagggactggagtggattgggtatatctattacagtgggagcaccaactacaacccctccctcaagagtcgagtcaccatatcagtaga
V4-59_09	gggaagggactggagtggattgggtacatctataccagtgggagcaccaactacaacccctccctcaagagtcgagtcaccatatcagtaga
V4-59_10	gggaagggactggagtggattgggtatatctattacagtgggagcaccaactacaacccctccctcaagagtcgagtcaccatatcagtaga
V4-59_11	gggaaggggctggagtggattgggtatatctattacagtgggagcaccaactacaacccctccctcaagagtcgagtcaccatatcagtaga
V4-59_12	gggaagggactggagtggattgggtacatctataccagtgggagcaccaactacaacccctccctcaagagtcgagtcaccatatcagtaga
V4-59_13	gggaagggactggagtggattgggtatatctattacagtgggagcaccaactacaacccctccctcaagagtcgagtcaccatatcagtaga
IGHV4-59*01	gggaagggactggagtggattgggtatatctattacagtgggagcaccaactacaacccctccctcaagagtcgagtcaccatatcagtaga
IGHV4-59*02	gggaagggactggagtggattgggtatatctattacagtgggagcaccaactacaacccctccctcaagagtcgagtcaccatatcagtaga
IGHV4-59*10	gggaaggggctggagtggattgggcgtatctataccagtgggagcaccaactacaacccctccctcaagagtcgagtcaccatgtcagtaga
IGHV4-59*11	gggaagggactggagtggattgggtatatctattacagtgggagcaccaactacaacccctccctcaagagtcgagtcaccatatcagtaga
IGHV4-59*03	gggaagggactggagtggattgggtatatctattacagtgggagcaccaactacaacccctccctcaagagtcgagtcaccatatcagtaga
IGHV4-59*04	gggaagggactggagtggattgggtatatctattatagtgggagcacctactacaacccgtccctcaagagtcgagtcaccatgtcagtaga
IGHV4-59*05	gggaagggactggagtggattgggcgtatctattatagtgggagcacctactacaacccgtccctcaagagtcgagtcaccatatccgtaga
IGHV4-59*06	gggaagggcctggagtggattgggtacatctattacagtgggagcacctactacaacccgtccctcaagagtcgagttaccatatcagtaga
IGHV4-59*07	gggaagggactggagtggattgggtatatctattacagtgggagcaccaactacaacccctccctcaagagtcgagtcaccatatcagtaga
IGHV4-59*08	gggaagggactggagtggattgggtatatctattacagtgggagcaccaactacaacccctccctcaagagtcgagtcaccatatcagtaga
IGHV4-59*09	ggnannngactggagtggattgggtatatctattacagtgggagcaccaactacaacccctccctcaagagtcgagtcaccatatcagtaga
IGHV4-59*12	gggaagggactggagtggattgggtatatctattacagtgggagcaccaactacaacccctccctcaagagtcgagtcaccatatcagtaga
IP_V4-59*p11	-------gactggagtggattgggtatatctattacagtgggagcaccaactacaacccctccctcaagagtcgagtcaccatatcagtaga
IP_V4-59*p12	gggaaggggctggagtggattgggagtatctattacagtgggagcaccaactacaacccctccctcaagagtcgagtcaccatatcagtaga
IP_V4-59*p13	gggaagggactggagtggattgggtatatctattacagtgggagcaccaactacaacccctccctcaagagtcgagtcaccatatcagtaga
IP_V4-59*p14	gggaagggactggagtggattgggtatatctattacagtgggagcaccaactacaacccgtccctcaagagtcgagtcaccatatcagtaga
IP_V4-59*p15	gggaagggactggagtggattgggtatatctattacagtgggagcaccaactacaacccctccctcaagagtcgagtcaccatatcagtaga
IP_V4-59*p16	gggaagggactggagtggattgggtgtatctattacagtgggagcaccaactacaacccctccctcaagagtcgagtcaccatatcagtaga
IP_V4-59*p17	gggaagggactggagtggattgggtatatctattacagtgggagcaccaactacaacccgtccctcaagagtcgagtcaccatatcagtaga
IP_V4-59*p18	gggaagggactggagtggattgggtacatctatac--atgggagcaccaactacaacccctccctcaagagtcgagtcaccatatcagtaga
IP_V4-59*p19	gggaagggactggagtggattgggtatatctattacagtgggagcaccaactacaacccctccctcaagagtcgagtcaccatatcagtaga
IP_V4-59*p20	gggaagggactggagtggattgggtatatctattacagtgggagcaccaactacaacccctccctcaagagtcgagtcaccatatcagtaga
IP_V4-59*p21	gggaagggactggagtggattgggtatatctattacagtgggagcaccaactacaacccctccctcaagagtcgagtcaccatatcagtaga
IP_V4-59*p22	gggaagggactggagtggattgggtatatctattacagtgggagcaccaactacaacccctccctcaagagtcgagtcaccatatcagtaga
V_V4-59*01	gggaagggactggagtggattgggtatatctattacagtgggagcaccaactacaacccctccctcaagagtcgagtcaccatatcagtaga
V_V4-59*02	gggaagggactggagtggattgggtatatctattacagtgggagcaccaactacaacccctccctcaagagtcgagtcaccatatcagtaga

V4-59_01	cacgtccaagaaccagttctccctgaagctgagctctgtgaccgctgcggacacggccgtgtattactgtgcgagaga
V4-59_02	cacgtccaagaaccagttctccctgaagctgagctctgtgaccgccgcagacacggccgtgtattactgtgcgagaga
V4-59_03	cacgtccaagaaccagttctccctgaagctgagctctgtgaccgctgcggacacggccgtgtattactgtgcgagaga
V4-59_04	cacgtccaagaaccagttctccctgaagctgagctctgtgaccgctgcggacacggccgtgtattactgtgcgagaga
V4-59_05	cacgtccaagaaccagttctccctgaagctgagctctgtgaccgctgcggacacggccgtgtattactgtgcgagaga
V4-59_06	cacgtccaagaaccagttctccctgaagctgagctctgtgaccgctgcggacacggccgtgtattactgtgcgagaga
V4-59_07	cacgtccaagaaccagttctccctgaagctgagctctgtgaccgctgcggacacggccgtgtattactgtgcgagaga
V4-59_08	cacgtccaagaaccagttctccctgaagctgagctctgtgaccgctgcggacacggccgtgtattactgtgcgagaga
V4-59_09	cacgtccaagaaccagttctccctgaagctgagctctgtgaccgccgcagacacggccgtgtattactgtgcgagaga
V4-59_10	cacgtccaagaaccagttctccctgaagctgagctctgtgaccgctgcggacacggccgtgtattactgtgcgagaga
V4-59_11	cacgtccaagaaccagttctccctgaagctgagctctgtgaccgctgcggacacggccgtgtattactgtgcgagaga
V4-59_12	cacgtccaagaaccagttctccctgaagctgagctctgtgaccgctgcggacacggccgtgtattactgtgcgagaga
V4-59_13	cacttccaagaaccagttctccctgaagctgagctctgtgaccgctgcggacacggccgtgtattactgtgcgagaga
IGHV4-59*01	cacgtccaagaaccagttctccctgaagctgagctctgtgaccgctgcggacacggccgtgtattactgtgcgagaga
IGHV4-59*02	cacgtccaagaaccagttctccctgaagctgagctctgtgaccgctgcggacacggccgtgtattactgtgcgagaga
IGHV4-59*10	cacgtccaagaaccagttctccctgaagctgagctctgtgaccgccgcggacacggccgtgtattactgtgcgagata
IGHV4-59*11	cacgtccaagaaccagttctccctgaagctgagctctgtgaccgctgcggacacggccgtgtattactgtgcgagaga
IGHV4-59*03	cacgtccaagaaccaattctccctgaagctgagctctgtgaccgctgcggacacggccgtgtattactgtgcg-----
IGHV4-59*04	cacgtccaagaaccagttctccctgaagctgagctctgtgaccgccgcagacacggctgtgtattactgtgcg-----
IGHV4-59*05	cacgtccaagaaccagttctccctgaagctgagctctgtgaccgccgcagacacggctgtgtattactgtgcg-----
IGHV4-59*06	cacgtctaagaaccagttctccctgaagctgagctctgtgactgccgcggacacggccgtgtattactgtgcg-----
IGHV4-59*07	cacgtccaagaaccagttctccctgaagctgagctctgtgaccgctgcggacacggccgtgtattactgtgcgaga--
IGHV4-59*08	cacgtccaagaaccagttctccctgaagctgagctctgtgaccgccgcagacacggccgtgtattactgtgcgagaca
IGHV4-59*09	cacgtccaagaaccagttctccctgaagctgagctctgtgaccgctgcggacacggccgtgtattactgtgcgagagg
IGHV4-59*12	cacgtccaagaaccagttctccctgaagctgagctctgtgaccgccgcggacacggccgtgtattactgtgcgagaga
IP_V4-59*p11	cacgtccaagaaccagttctccctgaagctgagctctgtgaccgctgcggacacagccgtgtattactgtgcgagaga
IP_V4-59*p12	cacgtccaagaaccagttctccctgaagctgagctctgtgaccgctgcggacacggccgtgtattactgtgcgagaga
IP_V4-59*p13	cacgtccaagaaccagttctccctgaagctgagctctgtgaccgctgcggacacggccgtgtattactgtgcgagaga
IP_V4-59*p14	cacgtccaagaaccagttctccctgaagctgagctctgtgaccgctgcggacacggccgtgtattactgtgcgagaga
IP_V4-59*p15	cacgtccaagaaccagttctccctgaagctgagctctgtgaccgctgcggacacggccgtgtattactgtgcgagaga
IP_V4-59*p16	cacgtccaagaaccagttctccctgaagctgagctctgtgaccgctgcggacacggccgtgtattactgtgcgagaca
IP_V4-59*p17	cacgtccaagaaccagttctccctgaagctgagctctgtgaccgctgcggacacggccgtgtattac-----------
IP_V4-59*p18	cacgtccaagaaccagttctccctgaagctgagctctgtgaccgccgcagacacggccgtgtattactgtgcgagaca
IP_V4-59*p19	cacgtccaagaaccagttctccctgaagctgagctctgtgaccgctgcggacacggccgtgtattactgtgcgagaga
IP_V4-59*p20	cacgtccaagaaccagttctccctgaagctgagctctgtgaccgccgcggacacggccgtgtattactgtgcgagaga
IP_V4-59*p21	cacgtccaagaaccagttctccctgaagctgagctctgtgaccgccgcggacacggccgtgtattactgtgcgagaca
IP_V4-59*p22	cacgtccaagaaccagttctccctgaagctgagctctgtgaccgctgcggacacggccgtgtattactgtgcgagaca
V_V4-59*01	cacgtccaagaaccagttctccctgaagctgagctctgtgaccgctgcggacacggccgtgtattactgtgcgaga--
V_V4-59*02	cacgtccaagaaccagttctccctgaagctgagctctgtgaccgctgcggacacggccgtgtattactgtgcgaga--

IGHV4-61

```
V4-61_01        atgaaacacctgtggttcttcctcctcctggtggcagctcccagatgggtcctgtccaggtgcagctgcaggagtcgggcccaggactg
V4-61_02        atgaaacatctgtggttcttcctcctcctggtggcagctcccagatgggtcctgtccaggtgcagctgcaggagtcgggcccaggactg
V4-61_03        atgaaacacctgtggttcttcctcctcctggtggcagctcccagatgggtcctgtccaggtgcagctgcaggagtcgggcccaggactg
V4-61_04        atgaaacatctgtggttcttcctcctcctggtggcagctcccagatgggtcctgtccaggtgcagctgcaggagtcgggcccaggactg
V4-61_05        atgaaacacctgtggttcttcctcctcctggtggcagctcccagatgggtcctgtccaggtgcagctgcaggagtcgggcccaggactg
V4-61_06        atgaaacacctgtggttcttcctcctcctggtggcagctcccagatgggtcctgtccaggtgcagctgcaggagtcgggcccaggactg
V4-61_07        atgaaacatctgtggttcttcctcctcctggtggcagctcccagatgggtcctgtccaggtgcagctgcaggagtcgggcccaggactg
V4-61_08        atgaaacatctgtggttcttcctcctcctggtggcagctcccagatgggtcctgtccaggtgcagctgcaggagtcgggcccaggactg
V4-61_09        atgaaacacctgtggttcttcctcctcctggtggcagctcccagatgggtcctgtctcaggtgcagctgcaggagtcgggcccaggactg
V4-61_10        atgaaacacctgtggttcttcctcctcctggtggcagctcccagatgggtcctgtctcaggtgcagctgcaggagtcgggcccaggactg
V4-61_11        atgaaacatctgtggttcttcctcctcctggtggcagctcccagatgggtcctgtccaggtgcagctgcaggagtcgggcccaggactg
V4-61_12        atgaaacatctgtggttcttcctcctcctggtggcagctcccagatgggtcctgtctcaggtgcagctgcaggagtcgggcccaggactg
V4-61_13        atgaaacatctgtggttcttcctcctcctggtggcagctcccagatgggtcctgtctcaggtgcagctgcaggagtcgggcccaggactg
V4-61_14        atgaaacacctgtggttcttcctcctcctggtggcagctcccagatgggtcctgtccaggtgcagctgcaggagtcgggcccaggactg
V4-61_15        atgaaacatctgtggttcttcctcctcctggtggcagctcccagatgggtcctgtccaggtgcagctgcaggagtcgggcccaggactg
V4-61_16        atgaaacatctgtggttcttcctcctcctggtggcagctcccagatgggtcctgtctcaggtgcagctgcaggagtcgggcccaggactg
V4-61_17        atgaaacatctgtggttcttcctcctcctggtggcagctcccagatgggtcctgtccaggtgcagctgcaggagtcgggcccaggactg
V4-61_18        atgaaacacctgtggttcttcctcctcctggtggcagctcccagatgggtcctgtccaggtgcagctgcaggagtcgggcccaggactg
V4-61_19        atgaaacacctgtggttcttcctcctcctggtggcagctcccagatgggtcctgtctcaggtgcagctgcaggagtcgggcccaggactg
V4-61_20        atgaaacatctgtggttcttcctcctcctggtggcagctcccagatgggtcctgtccaggtgcagctgcaggagtcgggcccaggactg
V4-61_21        atgaaacacctgtggttcttcctcctcctggtggcagctcccagatgggtcctgtctcaggtgcagctgcaggagtcgggcccaggactg
V4-61_22        atgaaacacctgtggttcttcctcctcctggtggcagctcccagatgggtcctgtccaggtgcagctgcaggagtccggctcaggactg
V4-61_23        atgaaacacctgtggttcttcctcctcctggtggcagctcccagatgggtcctgtccaggtgcagctgcaggagtcgggcccaggactg
V4-61_24        atgaaacacctgtggttcttcctcctcctggtggcagctcccagatgggtcctgtccaggtgcagctgcaggagtcgggcccaggactg
IGHV4-61*01     atgaaacacctgtggttcttcctcctcctggtggcagctcccagatgggtcctgtccaggtgcagctgcaggagtcgggcccaggactg
IGHV4-61*03     atgaaacacctgtggttcttcctcctcctggtggcagctcccagatgggtcctgtccaggtgcagctgcaggagtcgggcccaggactg
IGHV4-61*04     atgaaacacctgtggttcttcctcctcctggtggcagctcccagatgggtcctgtccaggtgcagctgcaggagtcgggcccaggactg
IGHV4-61*08     atgaaacacctgtggttcttcctcctcctggtggcagctcccagatgggtcctgtccaggtgcagctgcaggagtcgggcccaggactg
IGHV4-61*09     atgaaacatctgtggttcttccttctcctggtggcagctcccagatgggtcctgtctcaggtgcagctgcaggagtcgggcccaggattg
IGHV4-61*02     ---------------------------------------------------------caggtgcagctgcaggagtcgggcccaggactg
IGHV4-61*05     ---------------------------------------------------------cagctgcagctgcaggagtcgggcccaggactg
IGHV4-61*06     -----------------------------------------------------------------------------------------
IGHV4-61*07     -----------------------------------------------------------------------------------------
IP_V4-61*p09    -----------------------------------------------------------------------------------------
IP_V4-61*p10    -----------------------------------------------------------------------------------------
IP_V4-61*p11    ---------------------------------------------------------caggtgcagctgcaggagtcgggcccaggattg
V_V4-61*01      ---------------------------------------------------------caggtgcagctgcaggagtcgggcccaggactg
V_V4-61*08      ---------------------------------------------------------caggtgcagctgcaggagtcgggcccaggactg
```

V4-61_01	gtgaagccttcggagaccctgtccctcacctgcactgtctctggtggctccgtcagcagtggtagttactactggagctggatccggcag
V4-61_02	gtgaagccttcggagaccctgtccctcacctgcactgtctctggtggctccgtcagcagtggtagttactactggagctggatccggcag
V4-61_03	gtgaagccttcggagaccctgtccctcacctgcactgtctctggtggctccgtcagcagtggtagttactactggagctggatccggcag
V4-61_04	gtgaagccttcacagaccctgtccctcacctgcactgtctctggtggctccgtcagcagtggtagttactactggagctggatccggcag
V4-61_05	gtgaagccttcggagaccctgtccctcacctgcactgtctctggtggctccgtcagcagtggtagttactactggagctggatccggcag
V4-61_06	gtgaagccttcggagaccctgtccctcacctgcactgtctctggtggctccgtcagtagtggtagttactactggagctggatccggcag
V4-61_07	gtgaagccttcacagaccctgtccctcacctgcactgtctctggtggctccgtcagcagtggtagttactactggagctggatccggcag
V4-61_08	gtgaagccttcggagaccctgtccctcacctgcactgtctctggtggctccgtcagcagtggtagttactactggagctggatccggcag
V4-61_09	gtgaagccttcggggaccctgtccctcacctgcgctgtctctggtggctccgtcagcagtggtagttactactggagctggatccggcag
V4-61_10	gtgaagccttcggggaccctgtccctcacctgcgctgtctctggtggctccgtcagtagtggtagttactactggagctggatccggcag
V4-61_11	gtgaagccttcacagaccctgtccctcacctgcactgtctctggtggctccgtcagcagtggtagttactactggagctggatccggcag
V4-61_12	gtgaagccttcacggaccctgtccctcacctgcactgtctctggtggctccgtcagcagtggtagttactactggagctggatccggcag
V4-61_13	gtgaagccttcacagaccctgtccctcacctgcactgtctctggtggctccgtcagcagtggtagttactactggagctggatccggcag
V4-61_14	gtgaagccttcggagaccctgtccctcacctgcactgtctctggtggctccgtcagtagtggtagttactactggagctggatccggcag
V4-61_15	gtgaagccttcacagaccctgtccctcacctgcactgtctctggtggctccgtcagcagtggtagttactactggagctggatccggcag
V4-61_16	gtgaagccttcacagaccctgtccctcacctgcactgtctctggtggctccgtcagcagtggtagttactactggagctggatccggcag
V4-61_17	gtgaagccttcggagaccctgtccctcacctgcactgtctctggtggctccgtcagcagtggtagttactactggagctggatccggcag
V4-61_18	gtgaagccttcggagaccctgtccctcacctgcactgtctctggtggctccgtcagcagtggtagttactactggagctggatccggcag
V4-61_19	gtgaagccttcacggaccctgtccctcacctgcgctgtctctggtggctccgtcagcagtggtggttactcctggagctggatccggcag
V4-61_20	gtgaagccttcggagaccctgtccctcacctgcactgtctctggtggctccgtcagcagtggtagttactactggagctggatccggcag
V4-61_21	gtgaagccttcggagaccctgtccctcacctgcactgtctctggtggctccgtcagtagtggtagttactactggagctggatccggcag
V4-61_22	gtgaagccttcggagaccctgtccctcacctgcactgtctctggtggctccgtcagtagtggtagttactactggagctggatccggcag
V4-61_23	gtgaagccttcggagaccctgtccctcacctgcactgtctctggtggctccgtcagcagtggtagttactactggagctggatccggcag
V4-61_24	gtgaagccttcggagaccctgtccctcacctgcactgtctctggtggctccgtcagcagtggtggttactcctggagctggatccggcag
IGHV4-61*01	gtgaagccttcggagaccctgtccctcacctgcactgtctctggtggctccgtcagcagtggtagttactactggagctggatccggcag
IGHV4-61*03	gtgaagccttcggagaccctgtccctcacctgcactgtctctggtggctccgtcagcagtggtagttactactggagctggatccggcag
IGHV4-61*04	gtgaagccttcggagaccctgtccctcacctgcactgtctctggtggctccgtcagcagtggtagttactactggagctggatccggcag
IGHV4-61*08	gtgaagccttcggagaccctgtccctcacctgcactgtctctggtggctccgtcagcagtggtggttactactggagctggatccggcag
IGHV4-61*09	gtgaagccttcacagaccctgtccctcacctgcactgtctctggtggctccatcagcagtggtagttactactggagctggatccggcag
IGHV4-61*02	gtgaagccttcacagaccctgtccctcacctgcactgtctctggtggctccatcagcagtggtagttactactggagctggatccggcag
IGHV4-61*05	gtgaagccttcggagaccctgtccctcacctgcactgtctctggtggctccatcagcagtagtagttactactgggctggatccggcag
IGHV4-61*06	-------------------------------------tctggtggctccgtcagcagtggtagttactactggagctggatccggcag
IGHV4-61*07	-------------------------------------tctggtggctccgtcagcagtggtagttactactggagctggatccggcag
IP_V4-61*p09	--------------------------------cactgtctctggtggctccatcagcagtggtagttactactggagctggatccggcag
IP_V4-61*p10	--------------------------------cactgtctctggtggctccatcagcagtggtagttactactggagctggatccggcag
IP_V4-61*p11	gtgaagccttcacagaccctgtccctcacctgcactgtctctggtggctccatcagcagtggtagttactactggagctggatccggcag
V_V4-61*01	gtgaagccttcggagaccctgtccctcacctgcactgtctctggtggctccgtcagcagtggtagttactactggagctggatccggcag
V_V4-61*08	gtgaagccttcggagaccctgtccctcacctgcactgtctctggtggctccgtcagcagtggtggttactactggagctggatccggcag

V4-61_01	cccccagggaagggactggagtggattgggtatatctattacagtgggagcaccaactacaacccctccctcaagagtcgagtcaccat
V4-61_02	cccccagggaagggactggagtggattgggtatatctattacagtgggagcaccaactacaacccctccctcaagagtcgagtcaccat
V4-61_03	cccccagggaagggactggagtggattgggtatatctattacagtgggagcaccaactacaacccctccctcaagagtcgagtcaccat
V4-61_04	cccgccgggaagggactggagtggattgggcgtatctatacagtgggagcaccaactacaacccctccctcaagagtcgagtcaccat
V4-61_05	cccccagggaagggactggagtggattgggtatatctattacagtgggagcaccaactacaacccctccctcaagagtcgagtcaccat
V4-61_06	cccccagggaagggactggagtggattgggtatatctattacagtgggagcaccaactacaacccctccctcaagagtcgagtcaccat
V4-61_07	cccgccgggaagggactggagtggattgggcatatctatacagtgggagcaccaactacaacccctccctcaagagtcgagtcaccat
V4-61_08	cccccagggaagggactggagtggattgggtatatctattacagtgggagcaccaactacaacccctccctcaagagtcgagtcaccat
V4-61_09	cccccagggaagggactggagtggattgggtatatctattacagtgggagcaccaactacaacccctccctcaagagtcgagtcaccat
V4-61_10	cccccagggaagggactggagtggattgggtatatctattacagtgggagcaccaactacaacccctccctcaagagtcgagtcaccat
V4-61_11	cccgccgggaagggactggagtggattgggtatatctattacagtgggagcaccaactacaacccctccctcaagagtcgagtcaccat
V4-61_12	cccgccgggaagggactggagtggattgggcgtatctatacagtgggagcaccaactacaacccctccctcaagagtcgagtcaccat
V4-61_13	cccgccgggaagggactggagtggattgggcgtatctatacagtgggagcaccaactacaacccctccctcaagagtcgagtcaccat
V4-61_14	cccccagggaagggactggagtggattgggtatatctattacagtgggagcaccaactacaacccctccctcaagagtcgagtcaccat
V4-61_15	cccgccgggaagggactggagtggattgggcgtatctatacagtgggagcaccaactacaacccctccctcaagagtcgagtcaccat
V4-61_16	cccgccgggaagggactggagtggattgggcatatctatacagtgggagcaccaactacaacccctccctcaagagtcgagtcaccat
V4-61_17	cccgccgggaagggactggagtggattgggtatatctattacagtgggagcaccaactacaacccctccctcaagagtcgagtcaccat
V4-61_18	ccaccagggaagggactggagtggattgggtatatctattacagtgggagcaccaactacaacccctccctcaagagtcgagtcaccat
V4-61_19	ccaccagggaagggactggagtggattgggtatatctattacagtgggagcaccaactacaacccctccctcaagagtcgagtcaccat
V4-61_20	cccgccgggaagggactggagtggattgggcgtatctatacagtgggagcaccaactacaacccctccctcaagagtcgagtcaccat
V4-61_21	cccccagggaagggactggagtggattgggtatatctattacagtgggagcaccaactacaacccctccctcaagagtcgagtcaccat
V4-61_22	cccccagggaagggactggagtggattgggtatatctattacagtgggagcaccaactacaacccctccctcaagagtcgagtcaccat
V4-61_23	cccgccgggaagggactggagtggattgggcatatctatacagtgggagcaccaactacaacccctccctcaagagtcgagtcaccat
V4-61_24	ccaccagggaagggactggagtggattgggtatatctattacagtgggagcaccaactacaacccctccctcaagagtcgagtcaccat
IGHV4-61*01	cccccagggaagggactggagtggattgggtatatctattacagtgggagcaccaactacaacccctccctcaagagtcgagtcaccat
IGHV4-61*03	cccccagggaagggactggagtggattgggtatatctattacagtgggagcaccaactacaacccctccctcaagagtcgagtcaccat
IGHV4-61*04	cccccagggaagggactggagtggattggatatatctattacagtgggagcaccaactacaacccctccctcaagagtcgagtcaccat
IGHV4-61*08	cccccagggaagggactggagtggattgggtatatctattacagtgggagcaccaactacaacccctccctcaagagtcgagtcaccat
IGHV4-61*09	cccgccgggaagggactggagtggattgggcatatctatacagtgggagcaccaactacaacccctccctcaagagtcgagtcaccat
IGHV4-61*02	cccgccgggaagggactggagtggattgggcgtatctatacagtgggagcaccaactacaacccctccctcaagagtcgagtcaccat
IGHV4-61*05	cccccagggaagggactggagtggattgggtatatctattacagtgggagcaccaactacaacccctccctcaagagtcgagtcaccat
IGHV4-61*06	cccccagggaagggactggagtggattgggtatatctattacagtgggagcaccaactacaacccctccctcaagagtcgagtcaccat
IGHV4-61*07	cccccagggaagggactggagtggattgggtatatctattacagtgggagcaccaactacaacccctccctcaagagtcgagtcaccat
IP_V4-61*p09	cccgccgggaagggactggagtggattgggcgtatctatacagtgggagcaccaactacaacccctccctcaagagtcgagtcaccat
IP_V4-61*p10	cccgccgggaagggactggagtggattgggcatatctatacagtgggagcaccaactacaacccctccctcaagagtcgagtcaccat
IP_V4-61*p11	ccccc-gggaagggactggagtggattgggcatatctat-acagtgggagcaccaactacaacccctccctcaagagtcgagtcaccat
V_V4-61*01	cccccagggaagggactggagtggattgggtatatctattacagtgggagcaccaactacaacccctccctcaagagtcgagtcaccat
V_V4-61*08	cccccagggaagggactggagtggattgggtatatctattacagtgggagcaccaactacaacccctccctcaagagtcgagtcaccat

V4-61_01	atcagtagacacgtccaagaaccagttctccctgaagctgagctctgtgaccgctgcggacacggccgtgtattactgtgcgaga--
V4-61_02	atcagtagacacgtccaagaaccagttctccctgaagctgagctctgtgaccgctgcggacacggccgtgtattactgtgcgaga--
V4-61_03	atcagtagacacgtccaagaaccagttctccctgaagctgagctctgtgaccgccgcagacacggccgtgtattactgtgcgaga--
V4-61_04	atcagtagacacgtccaagaaccagttctccctgaagctgagctctgtgaccgccgcagacacggccgtgtattactgtgcgaga--
V4-61_05	atcagtagacacgtccaagaaccagttctccctgaagctgagctctgtgaccgccgcggacacggccgtgtattactgtgcgaga--
V4-61_06	atcagtagacacgtccaagaaccagttctccctgaagctgagctctgtgaccgccgcagacacggccgtgtattactgtgcgaga--
V4-61_07	atcagtagacacgtccaagaaccagttctccctgaagctgagctctgtgaccgccgcagacacggccgtgtattactgtgcgaga--
V4-61_08	atcagtagacacgtccaagaaccagttctccctgaagctgagctctgtgaccgccgcagacacggccgtgtattactgtgcgaga--
V4-61_09	atcagtagacacgtccaagaaccagttctccctgaagctgagctctgtgaccgccgcagacacggccgtgtattactgtgcgaga--
V4-61_10	atcagtagacacgtccaagaaccagttctccctgaagctgagctctgtgaccgccgcagacacggccgtgtattactgtgcgaga--
V4-61_11	atcagtagacacgtccaagaaccagttctccctgaagctgagctctgtgaccgccgcggacacggccgtgtattactgtgcgaga--
V4-61_12	atcagtagacacgtccaagaaccagttctccctgaagctgagctctgtgaccgccgcagacacggccgtgtattactgtgcgaga--
V4-61_13	atcagtagacacgtccaagaaccagttctccctgaagctgagctctgtgaccgccgcagacacggccgtgtattactgtgcgaga--
V4-61_14	atcagtagacacgtccaagaaccagttctccctgaagctgagctctgtgaccgccgcggacacggccgtgtattactgtgcgaga--
V4-61_15	atcagtagacacgtccaagaaccagttctccctgaagctgagctctgtgaccgccgcggacacggccgtgtattactgtgcgaga--
V4-61_16	atcagtagacacgtccaagaaccagttctccctgaagctgagctctgtgaccgccgcagacacggccgtgtattactgtgcgaga--
V4-61_17	atcagtagacacgtccaagaaccagttctccctgaagctgagctctgtgaccgctgcggacacggccgtgtattactgtgcgaga--
V4-61_18	atcagtagacacgtccaagaaccagttctccctgaagctgagctctgtgaccgctgcggacacggccgtgtattactgtgcgaga--
V4-61_19	atcagtagacacgtccaagaaccagttctccctgaagctgagctctgtgaccgccgcagacacggccgtgtattactgtgcgaga--
V4-61_20	atcagtagacacgtccaagaaccagttctccctgaagctgagctctgtgaccgccgcagacacggccgtgtattactgtgcgaga--
V4-61_21	atcagtagacacgtccaagaaccagttctccctgaagctgagctctgtgaccgccgcagacacggccgtgtattactgtgcgaga--
V4-61_22	atcagtagacacgtccaagaaccagttctccctgaagctgagctctgtgaccgctgcggacacggccgtgtattactgtgcgaga--
V4-61_23	atcagtagacacgtccaagaaccagttctccctgaagctgagctctgtgaccgccgcggacacggccgtgtattactgtgcgaga--
V4-61_24	atcagtagacacgtccaagaaccagttctccctgaagctgagctctgtgaccgccgcggacacggccgtgtattactgtgcgaga--
IGHV4-61*01	atcagtagacacgtccaagaaccagttctccctgaagctgagctctgtgaccgctgcggacacggccgtgtattactgtgcgagaga
IGHV4-61*03	atcagtagacacgtccaagaaccacttctccctgaagctgagctctgtgaccgctgcggacacggccgtgtattactgtgcgagaga
IGHV4-61*04	atcagtagacacgtccaagaaccagttctccctgaagctgagctctgtgaccgct---gacacggccgtgtattactg---------
IGHV4-61*08	atcagtagacacgtccaagaaccagttctccctgaagctgagctctgtgaccgctgcggacacggccgtgtattactgtgcgagaga
IGHV4-61*09	atcagtagacacgtccaagaaccagttctccctgaagctgagctctgtgaccgccgcagacacggccgtgtattactgtgcgagaga
IGHV4-61*02	atcagtagacacgtccaagaaccagttctccctgaagctgagctctgtgaccgccgcagacacggccgtgtattactgtgcgagaga
IGHV4-61*05	atcagtagacaagtccaagaaccagttctccctgaagctgagctctgtgaccgccgcggacacggccgtgtattactgtgcgaga--
IGHV4-61*06	atcagtagacacgtccaagaaccagttctccctgaagctgagctctgtgaccgccgcggacacggccgtgtattactgtgccagaga
IGHV4-61*07	atcagtagacacgtccaagaaccagttctccctgaagctgagctctgtgaccgctgcggacacggccgtgtattactgtgcgagaca
IP_V4-61*p09	gtcagtagacacgtccaagaaccagttctccctgaagctgagctctgtgaccgccgcagacacggccgtgtattactgtgcgagaga
IP_V4-61*p10	atcagtagacacgtccaagaaccagttctccctgaagctgagctctgtgaccgccgcagacacggccgtgtattactgtgcgagaga
IP_V4-61*p11	atcagtagacacgtccaagaaccagttctccctgaagctgagctctgtgaccgccgcagacacggccgtgtattactgtgcgaga--
V_V4-61*01	atcagtagacacgtccaagaaccagttctccctgaagctgagctctgtgaccgctgcggacacggccgtgtattactgtgcgaga--
V_V4-61*08	atcagtagacacgtccaagaaccagttctccctgaagctgagctctgtgaccgctgcggacacggccgtgtattactgtgcgaga--

IGHV5-51

```
V5-51_01        atggggtcaaccgccatcctcgccctcctcctggctgttctccaaggagtctgtgccgaggtgcagctggtgcagtctggagcagaggtg
V5-51_02        atggggtcaaccgccatcctcgccctcctcctggctgttctccaaggagtctgtgccgaggtgcagctggtgcagtctggagcagaggtg
V5-51_03        atggggtcaaccgccatccttgccctcctcctggctgttctccaaggagtctgtgccgaggtgcagctggtgcagtctggagcagaggtg
V5-51_04        atggggtcaaccgccatcctcgccctcctcctggctgttctccaaggagtctgtgccgaggtgcagctggtgcagtctggagcagaggtg
V5-51_05        atggggtcaaccgccatcctcgccctcctcctggctgttatccaaggagtctgtgccgaggtgcagctggtgcagtctggagcagaggtg
IGHV5-51*01     atggggtcaaccgccatcctcgccctcctcctggctgttctccaaggagtctgttccgaggtgcagctggtgcagtctggagcagaggtg
IGHV5-51*02     atggggtcaaccgccatcctcgccctcctcctagctattctccaaggagtctgtgccgaggtgcagctggtgcagtctggagcagaggtg
IGHV5-51*03     ---------------------------------------------------------gaggtgcagctggtgcagtctggagcagaggtg
IGHV5-51*04     ---------------------------------------------------------gaggtgcagctggtgcagtctggagcagaggtg
IGHV5-51*05     ------------------------------------------------------------------------------------------
IGHV5-51*06     ---------------------------------------------------------gaggtgcagctggtgcagtctggagcagaggtg
IP_V5-51*p010   ---------------------------------------------------------gaggtgcagctggtgcagtctggagcagaggtg
IP_V5-51*p06    ---------------------------------------------------------gagatgcagctggtgcagtctggagcagaggtg
IP_V5-51*p07    ---------------------------------------------------------gagatgcagctggtgcagtctggagcagaggtg
IP_V5-51*p08    ---------------------------------------------------------gaggtgcagctggtgcagtctggagcagaggtg
IP_V5-51*p09    ---------------------------------------------------------gaggtgcagctggtgcagtctggagcagaggtg
IP_V5-51*p11    ---------------------------------------------------------gaggtgcagctggtgcagtctggagcagaggtg
IP_V5-51*p12    ---------------------------------------------------------gaggtgcagctggtgcagtctggagcagaggtg
IP_V5-51*p13    ---------------------------------------------------------gaggtgcagctggtgcagtctggagcagaggtg
V_V5-51*01      ---------------------------------------------------------gaggtgcagctggtgcagtctggagcagaggtg
V_V5-51*03      ---------------------------------------------------------gaggtgcagctggtgcagtctggagcagaggtg
```

```
V5-51_01        aaaaagccggggagtctctgaaagatctcctgtaagggttctggatacagctttaccagctactggatcggctgggtgcgccagatgccc
V5-51_02        aaaaagcccgggagtctctgaaagatctcctgtaagggttctggatacagctttaccagctactggatcggctgggtgcgccagatgccc
V5-51_03        aaaaagcccgggagtctctgaaagatctcctgtaagggttctggatacagctttaccagctactggatcggctgggtgcgccagatgccc
V5-51_04        aaaaagcccgggagtctctgaaagatctcctgtaagggttctggatacagctttaccagctactggatcggctgggtgcaccagatgccc
V5-51_05        aaaaagccggggagtctctgaaagatctcctgtaagggttctggatacagctttaccagctactggatcggctgggtgcgccagatgccc
IGHV5-51*01     aaaaagcccgggagtctctgaaagatctcctgtaagggttctggatacagctttaccagctactggatcggctgggtgcgccagatgccc
IGHV5-51*02     aaaaagcccgggagtctctgaaagatctcctgtaagggttctggatacagctttaccagctactggaccggctgggtgcgccagatgccc
IGHV5-51*03     aaaaagccggggagtctctgaaagatctcctgtaagggttctggatacagctttaccagctactggatcggctgggtgcgccagatgccc
IGHV5-51*04     aaaaagccggggagtctctgaaagatctcctgtaagggttctggatacagctttaccagctactggatcggctgggtgcgccagatgccc
IGHV5-51*05     -aaaagcccgggagtctctgaaagatctcctgtaagggttctggatacagctttaccagctactggatcggctgggtgcgccagatgccc
IGHV5-51*06     aaaaagccggggagtctctgaaagatctcctgtaagggttctggatacagctttaccagctactggatcggctgggtgcgccagatgccc
IP_V5-51*p010   aaaaagccggggagtctctgaaagatctcctgtaagggttctggatacagctttaccagctactggatcggctgggtgcgccagatgccc
IP_V5-51*p06    aaaaagcccgggagtctctgaaagatctcctgtaagggttctggatacagctttaccagctactggatcggctgggtgcgccagatgccc
IP_V5-51*p07    aaaaagccggggagtctctgaaagatctcctgtaagggttctggatacagctttaccagctactggatcggctgggtgcgccagatgccc
IP_V5-51*p08    aaaaagcccgggagtctctgagagatctcctgtaagggttctggatacagctttaccagctactggatcggctgggtgcgccagatgccc
IP_V5-51*p09    aaaaagcccgggagtctctgagagatctcctgtaagggttctggatacagctttaccagctactggatcagctgggtgcgccagatgccc
IP_V5-51*p11    aaaaagccggggagtctctgaaagatctcctgtaagggttctggatacagctttaccagctactggatcggctgggtgcgccagatgccc
IP_V5-51*p12    aaaaagccggggagtctctgaaagatctcctgtaagggttctggatacagctttaccagctactggatcggctgggtgcgccagatgccc
IP_V5-51*p13    aaaaagccggggagtctctgaaagatctcctgtaagggttctggatacagctttaccagctactggatcggctgggtgcgccagatgccc
V_V5-51*01      aaaaagcccgggagtctctgaaagatctcctgtaagggttctggatacagctttaccagctactggatcggctgggtgcgccagatgccc
V_V5-51*03      aaaaagccggggagtctctgaaagatctcctgtaagggttctggatacagctttaccagctactggatcggctgggtgcgccagatgccc
```

V5-51_01	gggaaaggcctggagtggatggggatcatctatcctggtgactctgataccagatacagcccgtccttccaaggccaggtcaccatctcagc
V5-51_02	gggaaaggcctggagtggatggggatcatctatcctggtgactctgataccagatacagcccgtccttccaaggccaggtcaccatctcagc
V5-51_03	gggaaaggcctggagtggatggggatcatctatcctggtgactctgataccagatacagcccgtccttccaaggccaggtcaccatctcagc
V5-51_04	gggaaaggcctggagtggatggggatcatctatcctggtgactctgataccagatacagcccgtccttccaaggccaggtcaccatctcagc
V5-51_05	gggaaaggcctggagtggatggggatcatctatcctggtgactctgataccagatacagcccgtccttccaaggccaggtcaccatctcagc
IGHV5-51*01	gggaaaggcctggagtggatggggatcatctatcctggtgactctgataccagatacagcccgtccttccaaggccaggtcaccatctcagc
IGHV5-51*02	gggaaaggctggagtggatggggatcatctatcctggtgactctgataccagatacagcccgtccttccaaggccaggtcaccatctcagc
IGHV5-51*03	gggaaaggcctggagtggatggggatcatctatcctggtgactctgataccagatacagcccgtccttccaaggccaggtcaccatctcagc
IGHV5-51*04	gggaaaggcctggagtggatggggatcatctatcctggtgactctgataccagatacagcccgtccttccaaggccaggtcaccatctcagc
IGHV5-51*05	aggaaaggcctggagtggatggggatcatctatcctggtgactctgataccagatacagcccgtccttccaaggccaggtcaccatctcagc
IGHV5-51*06	gggaaaggcctggagtggatggggatcatctatcctggtgactctgataccagatacagcccgtccttccaaggccaggttaccatctcagc
IP_V5-51*p010	gggaaaggcctggagtggatggggatcatctatcctggtgactctgataccagatacagcccgtccttccaaggccaggtcaccatctcagc
IP_V5-51*p06	gggaaaggcctggagtggatggggatcatctatcctggtgactctgataccagatacagcccgtccttccaaggccaggtcaccatctcagc
IP_V5-51*p07	gggaaaggcctggagtggatggggatcatctatcctggtgactctgataccagatacagcccgtccttccaaggccaggtcaccatctcagc
IP_V5-51*p08	gggaaaggcctggagtggatggggatcatctatcctggtgactctgataccagatacagcccgtccttccaaggccaggtcaccatctcagc
IP_V5-51*p09	gggaaaggcctggagtggatggggatcatctatcctggtgactctgataccagatacagcccgtccttccaaggccaggtcaccatctcagc
IP_V5-51*p11	gggaaaggcctggagtggatggggatcatctatcctggtgactctgataccagatacagcccgtccttccaaggccaggtcaccatctcagc
IP_V5-51*p12	gggaaaggcctggagtggatggggatcatctatcctggtgactctgataccagatacagcccgtccttccaaggccaggttaccatctcagc
IP_V5-51*p13	gggaaaggcctggagtggatggggatcatctatcctggtgactctgataccagatacagcccgtccttccaaggccaggttaccatctcagc
V_V5-51*01	gggaaaggcctggagtggatggggatcatctatcctggtgactctgataccagatacagcccgtccttccaaggccaggtcaccatctcagc
V_V5-51*03	gggaaaggcctggagtggatggggatcatctatcctggtgactctgataccagatacagcccgtccttccaaggccaggtcaccatctcagc

V5-51_01	cgacaagtccatcagcaccgcctacctgcagtggagcagcctgaaggcctcggacaccgccatgtattactgtgcgaga
V5-51_02	cgacaagtccatcagcaccgcctacctgcagtggagcagcctgaaggcctcggacaccgccatgtattactgtgcgaga
V5-51_03	cgacaagtccatcagcaccgcctacctgcagtggagcagcctgaaggcctcggacaccgccatgtattactgtgcgaga
V5-51_04	cgacaagtccatcagcaccgcctacctgcagtggagcagcctgaaggcctcggacaccgccatgtattactgtgcgaga
V5-51_05	cgacaagtccatcagcaccgcctacctgcagtggagcagcctgaaggcctcggacaccgccatgtattactgtgcgaga
IGHV5-51*01	cgacaagtccatcagcaccgcctacctgcagtggagcagcctgaaggcctcggacaccgccatgtattactgtgcgaga
IGHV5-51*02	cgacaagtccatcagcaccgcctacctgcagtggagcagcctgaaggcctcggacaccgccatgtattactgtgcgaga
IGHV5-51*03	cgacaagtccatcagcaccgcctacctgcagtggagcagcctgaaggcctcggacaccgccatgtattactgtgcgaga
IGHV5-51*04	cgacaagcccatcagcaccgcctacctgcagtggagcagcctgaaggcctcggacaccgccatgtattactgtgcgaga
IGHV5-51*05	cgacaagtccatcagcaccgcctacctgcagtggagcagcctgaaggcctcggacaccgccatg---------------
IGHV5-51*06	cgacaagtccatcagcaccgcctacctgcagtggagcagcctgaaggcctcggacaccgccatgtattactgtgcgaga
IP_V5-51*p010	cgacaagtccatcagcaccgcctacctgcagtgggggcagcctgaaggcctcggacaccgccatgtattactgtgc----
IP_V5-51*p06	cgacaagtccatcagcaccgcctacctgcagtggagcagcctgaaggcctcggacaccgccatgtattactgtgc----
IP_V5-51*p07	cgacaagtccatcagcaccgcctacctgcagtggagcagcctgaaggcctcggacaccgccatgtattactgtgcga--
IP_V5-51*p08	cgacaagtccatcagcaccgcctacctgcagtggagcagcctgaaggcctcggacaccgccatgtattactgtgcga--
IP_V5-51*p09	cgacaagtccatcagcaccgcctacctgcagtggagcagcctgaaggcctcggacaccgccatgtattactgtgcg---
IP_V5-51*p11	cgacaagtccatcagcaccgcctacctgcagtggagcagcctgaaggcctcggacaccgccatgtattactgtgcgaga
IP_V5-51*p12	cgacaagtccatcagcaccgcctacctgcagtggagcagcctgaaggcctcggacaccgccatgtattactgtgcgaga
IP_V5-51*p13	cgacaagtccatcagcaccgcctacctgcagtggagcagcctgaaggcctcggacaccgccatgtattactgtgcgaga
V_V5-51*01	cgacaagtccatcagcaccgcctacctgcagtggagcagcctgaaggcctcggacaccgccatgtattactgtgcgaga
V_V5-51*03	cgacaagtccatcagcaccgcctacctgcagtggagcagcctgaaggcctcggacaccgccatgtattactgtgcgaga

IGHV6-1

```
V6-1_01        atgtctgtctccttcctcatcttcctgcccgtgctgggcctcccatggggtgtcctgtcacaggtacagctgcagcagtcaggtccaggact
V6-1_02        atgtctgtctccttcctcatcttcctgcccgtgctgggcctcccatggggtgtcctgtcacaggtacagctgcagcagtcaggtccaggact
IGHV6-1*01     atgtctgtctccttcctcatcttcctgcccgtgctgggcctcccatggggtgtcctgtcacaggtacagctgcagcagtcaggtccaggact
IGHV6-1*02     atgtctgtctccttcctcatcttcctg-ccgtgctgggcctcc--atgggtgtcctgtcacaggtacagctgcagcagtcaggtccgggact
IP_V6-1*p03    ------------------------------------------------------------caggtacagctgcagcagtcaggtccaggact
IP_V6-1*p04    ------------------------------------------------------------caggtacagctgcagcagtcaggtccaggact
IP_V6-1*p05    ------------------------------------------------------------caggtacagctgcagcagtcaggtccaggact
IP_V6-1*p06    ------------------------------------------------------------caggtacagctgcagcagtcaggtccaggact
IP_V6-1*p07    ------------------------------------------------------------caggtacagctgcagcagtcaggtccaggact
IP_V6-1*p08    ------------------------------------------------------------caggtacagctgcagcagtcaggtccaggact
IP_V6-1*p09    ------------------------------------------------------------caggtacagctgcagcagtcaggtccaggact
IP_V6-1*p10    ------------------------------------------------------------caggtacagctgcagcagtcaggtccaggact
IP_V6-1*p11    ------------------------------------------------------------caggtacagctgcagcagtcaggtccaggact
IP_V6-1*p12    ------------------------------------------------------------caggtacagctgcagcagtcaggtccaggact
IP_V6-1*p133   ------------------------------------------------------------caggtacagctgcagcagtcaggtccaggact
IP_V6-1*p14    ------------------------------------------------------------caggtacagctgcagcagtcaggtccaggact
V_V6-1*01      ------------------------------------------------------------caggtacagctgcagcagtcaggtccaggact
```

```
V6-1_01        ggtgaagccctcgcagaccctctcactcacctgtgccatctccggggacagtgtctctagcaacagtgctgcttggaactggatcagg
V6-1_02        ggtgaagccctcgcagaccctctcactcacctgtgccatctccggggacagtgtctctagcaacagtgctgcttggaactggatcagg
IGHV6-1*01     ggtgaagccctcgcagaccctctcactcacctgtgccatctccggggacagtgtctctagcaacagtgctgcttggaactggatcagg
IGHV6-1*02     ggtgaagccctcgcagaccctctcactcacctgtgccatctccggggacagtgtctctagcaacagtgctgcttggaactggatcagg
IP_V6-1*p03    ggtgaagccctcgcagaccctctcactcacctgtgccatctccggggacagtgtctctagcaacagtgctgcttggaactggatcagg
IP_V6-1*p04    ggtgaagccctcgcagaccctctcactcacctgtgccatctccggggacagtgtctctagcaacagtgctgcttggaactggatcagg
IP_V6-1*p05    ggtgaagccctcgcagaccctctcactcacctgtgccatctccggggacagtgtctctagcaacagtgctgcttggaactggatcagg
IP_V6-1*p06    ggtgaagccctcgcagaccctctcactcgcctgtgccatctccggggacagtgtctctagcaacagtgctgcttggaactggatcagg
IP_V6-1*p07    ggtgaagccctcgcagaccctctcactcacctgtgccatctccggggacagtgtctctagcaacagtgctgcttggaactggatcagg
IP_V6-1*p08    ggtgagggccctcgcagaccctctcactcacctgtgccatctccggggacagtgtctctagcaacagtgctgcttggaactggatcagg
IP_V6-1*p09    ggtgaagccctcgcagaccctctcactcacctgtgccatctccggggacagtgtctctagcaacagtgctgcttgggctggatcagg
IP_V6-1*p10    ggtgaagccctcgcagaccctctcactcacctgtgccatctccggggacagtgtctctagcaacagtgctgcttggaactggatcagg
IP_V6-1*p11    ggtgaagccctcgcagaccctctcactcacctgtgccatctccggggacagtgtctctagcaacagcgctgcttggaactggatcagg
IP_V6-1*p12    ggtgaagccctcgcagaccctctcactcgcctgtgccatctccggggacagtgtctctagcaacagtgctgcttggaactggatcagg
IP_V6-1*p133   ggtgaagccctcgcagaccctctcactcacctgtgccatctccggggacagtgtctctagcaacagtgctgcttgggctggatcagg
IP_V6-1*p14    ggtgaagccctcgcagaccctctcactcacctgtgccatctccggggacagtgtctctagcaacagtgctgcttggaactggatcagg
V_V6-1*01      ggtgaagccctcgcagaccctctcactcacctgtgccatctccggggacagtgtctctagcaacagtgctgcttggaactggatcagg
```

```
V6-1_01        cagtccccatcgagaggccttgagtggctgggaaggacatactacaggtccaagtggtataatgattatgcagtatctgtgaaaagtcgaat
V6-1_02        cagtccccatcgagaggccttgagtggctgggaaggacatactacaggtccaagtggtataatgattatgcagtatctgtgaaaagttgaat
IGHV6-1*01     cagtccccatcgagaggccttgagtggctgggaaggacatactacaggtccaagtggtataatgattatgcagtatctgtgaaaagtcgaat
IGHV6-1*02     cagtccccatcgagaggccttgagtggctgggaaggacatactacaggtccaagtggtataatgattatgcagtatctgtgaaaagtcgaat
IP_V6-1*p03    cagtccccatcgagaggccttgagtggctgggaaggacatactacaggtccaagtggtataatgattatgcagtatctgtgaaaagttgaat
IP_V6-1*p04    cagtccccatcgagaggccttgagtggctgggaaggacatactacaggtccaagtggtataatgattatgcagtatctgtgagaagtcgaat
IP_V6-1*p05    cagtccccatcgagaggccttgagtggctgggaaggacatactacaggtccaagtggtataatgattatgcagtatctgtgaaaagtcgaat
IP_V6-1*p06    cagtccccatcgagaggccttgagtggctgggaaggacatactacaggtccaagtggtataatgattatgcagtatctgtgaaaagtcgaat
IP_V6-1*p07    cagtccccatcgagaggccttgagtggctgggaaggacatactacaggtccaagtggtataatgattacgcagtatctgtgaaaagtcgaat
IP_V6-1*p08    cagtccccatcgagaggccttgagtggctgggaaggacatactacaggtccaagtggtataatgattatgcagtatctgtgaaaagtcgaat
IP_V6-1*p09    cagtccccatcgagaggccttgagtggctgggaaggacatactacaggtccaagtggtataatgattatgcagtatctgtgaaaagtcgaat
IP_V6-1*p10    cagtccccatcgagaggccttgagtggctgggaaggacatactacaggtccaagtggtataatgattatgcagtatctgtgaaaagt-gaat
IP_V6-1*p11    cagtccccatcgagaggccttgagtggctgggaaggacatactacaggtccaagtggtataatgattatgcagtatctgtgaaaagttgaat
IP_V6-1*p12    cagtccccatcgagaggccttgagtggctgggaaggacatactacaggtccaagtggtataatgattatgcagtatctgtgaaaagttgaat
IP_V6-1*p133   cagtccccatcgagaggccttgagtggctgggaaggacatactacaggtccaagtggtataatgattatgcagtatctgtgaaaagttgaat
IP_V6-1*p14    cagtccccatcgagaggccttgagtggctgggaaggacatactacaggtccaagtggtataatgattatgcagtatctgtgaaaagttgaat
V_V6-1*01      cagtccccatcgagaggccttgagtggctgggaaggacatactacaggtccaagtggtataatgattatgcagtatctgtgaaaagtcgaat

V6-1_01        aaccatcaacccagacacatccaagaaccagttctccctgcagctgaactctgtgactcccgaggacacggctgtgtattactgtgcaaga--
V6-1_02        aaccatcaacccagacacatccaagaaccagttctccctgcagctgaactctgtgactcccgaggacacggctgtgtattactgtgcaaga--
IGHV6-1*01     aaccatcaacccagacacatccaagaaccagttctccctgcagctgaactctgtgactcccgaggacacggctgtgtattactgtgcaagaga
IGHV6-1*02     aaccatcaacccagacacatccaagaaccagttctccctgcagctgaactctgtgactcccgaggacacggctgtgtattactgtgcaagaga
IP_V6-1*p03    aaccatcaacccagacacatccaagaaccagttctccctgcagctgaactctgtgactcccgaggacacggctgtgtattactgtgcaag---
IP_V6-1*p04    aaccatcaacccagaca----------------------------------------------------------------------------
IP_V6-1*p05    aaccatcaacccagacacatccaagaaccagttctccctgcagctgaactctgtgactcccgaggacacagctgtgtattactgtgcaagaga
IP_V6-1*p06    aaccatcaacccagacacatccaagaaccagttctccctgcagctgaactctgtgactcccgaggacacggctgtgtattactgtgcaagaga
IP_V6-1*p07    aaccatcaacccagacacatccaagaaccagttctccctgcagctgaactctgtgactcccgaggacacggctgtgtattactgtgcaagaga
IP_V6-1*p08    aaccatcaacccagacacatccaagaaccagttctccctgcagctgaactctgtgactcccgaggacacggctgtgtattactgtgcaag---
IP_V6-1*p09    aaccatcaacccagacacatccaagaaccagttctccctgcagctgaactctgtgactcccgaggacacggctg-------------------
IP_V6-1*p10    aaccatcaacccagacacatccaagaaccagttctccctgcagctgaactctgtgactcccgaggacacggctgtgtattactgtgcaag---
IP_V6-1*p11    aaccatcaacccagacacatccaagaaccagttctccctgcagctgaactctgtgactcccgaggacacggctgtgtattactgtgcaa----
IP_V6-1*p12    aaccatcaacccagacacatccaagaaccagttctccctgcagctgaactctgtgactcccgaggacacggctgtgtattactgtgcaag---
IP_V6-1*p133   aaccatcaacccagacacatccaagaaccagttctccctgcagctgaactctgtgactcccgaggacacggctgtgtattactgtgcaagaga
IP_V6-1*p14    agccatcaacccagacacatccaagaaccagttctccctgcagctgaactctgtgactcccgaggacacggctgtgtattactgtgcaag---
V_V6-1*01      aaccatcaacccagacacatccaagaaccagttctccctgcagctgaactctgtgactcccgaggacacggctgtgtattactgtgcaaga--
```

IGHV7-81

```
V7-81_01      atggactggacctggagcatcctcttcttggtggcagcagcaacaggtacctactcccaggtgcagctggtgcagtctggccatgaggtga
V7-81_02      atggactggacctggagcatcctcttcttggtggcagcagcaacaggtacctactcccaggtgcagctggtgcagtctggccatgaggtga
V7-81_03      atggactggacctggagcatcctcttcttggtggcagcagcaacaggtacctactcccaggtgcagttggtgcagtctggccatgaggtga
V7-81_04      atggactggacctggagcatcctcttcttggtggcagcagcaacaggtacctactcccaggtgcagctggtgcagtctggccatgaggtga
V7-81_05      atggactggacctggagcatcctcttcttggtggcagcagcaacaggtacctactcccaggtgcagctggtgcagtctggccatgaggtga
V7-81_06      atggactggacctggagcatcctcttcttggtggcagcagcaacaggtacctactcccaggtgcagttggtgcagtctggccatgaggtga
V7-81_07      atggactggacctggagcatcctcttcttggtggcagcagcaacaggtacctactcccaggtgcagttggtgcagtctggccatgaggtga
IGHV7-81*01   atggactggacctggagcatcctcttcttggtggcagcagcaacaggtacctactcccaggtgcagctggtgcagtctggccatgaggtga
IGHV7-4-1*02  atggactggacctggaggatcctcttcttggtggcagcagcaacaggtgcccactcccaggtgcagctggtgcaatctgggtctgagttga
IGHV7-4-1*01  ----------------------------------------------------------caggtgcagctggtgcaatctgggtctgagttga
IGHV7-4-1*03  ----------------------------------------------------------caggtgcagctggtgcaatctgggtctgagttga
IGHV7-4-1*04  ----------------------------------------------------------caggtgcagctggtgcaatctgggtctgagttga
IGHV7-4-1*05  ----------------------------------------------------------caggtgcagctggtgcaatctgggtctgagttga
V_V7-81*01    ----------------------------------------------------------caggtgcagctggtgcagtctggccatgaggtga

V7-81_01      agcagcctggggcctcagtgaaggtctcctgcaaggcttctggttacagtttcaccacctatggtatgaattgggtgccacaggcccct
V7-81_02      agcagcctggggcctcagtgaaggtctcctgcaaggcttctggttacagtttcaccacctatggtatgaattgggtgccacaggcccct
V7-81_03      agcagcctggggcctcagtgaaggtctcctgcaaggcttctggttacagtttcaccacctatggtatgaattgggtgccacaggcccct
V7-81_04      agcagcctggggcctcagtgaaggtctcctgcaaggcttctgtttacagtttcaccacctatggtatgaattgggtgccacaggcccct
V7-81_05      agcagcctggggcctcagtgaaggtctcctgcaaggcttctgtttacagtttcaccacctatggtatgaattgggtgccacaggcccct
V7-81_06      agcagcctggggcctcagtgaaggtctcctgcaaggcttctggttacagtttcaccacctatggtatgaattgggtgccacaggcccct
V7-81_07      agcagcctggggcctcagtgaaggtctcctgcaaggcttctggttacagtttcaccacctatggtatgaattgggtgccacaggcccct
IGHV7-81*01   agcagcctggggcctcagtgaaggtctcctgcaaggcttctggttacagtttcaccacctatggtatgaattgggtgccacaggcccct
IGHV7-4-1*02  agaagcctggggcctcagtgaaggtttcctgcaaggcttctggatacaccttcactagctatgctatgaattgggtgcgacaggcccct
IGHV7-4-1*01  agaagcctggggcctcagtgaaggtttcctgcaaggcttctggatacaccttcactagctatgctatgaattgggtgcgacaggcccct
IGHV7-4-1*03  agaagcctggggcctcagtgaaggtttcctgcaaggcttctggatacaccttcactagctatgctatgaattgggtgcgacaggcccct
IGHV7-4-1*04  agaagcctggggcctcagtgaaggtttcctgcaaggcttctggatacaccttcactagctatgctatgaattgggtgcgacaggcccct
IGHV7-4-1*05  agaagcctggggcctcagtgaaggtttcctgcaaggcttctggatacaccttcactagctatgctatgaattgggtgcgacaggcccct
V_V7-81*01    agcagcctggggcctcagtgaaggtctcctgcaaggcttctggttacagtttcaccacctatggtatgaattgggtgccacaggcccct
```

```
V7-81_01        ggacaagggcttgagtggatgggatggttcaacacctacactgggaacccaacatatgcccagggcttcacaggacggtttgtcttctccaa
V7-81_02        ggacaagggcttgagtggatgggatggttcaacacctacactgggaacccaacatatgcccagggcttcacaggacggtttgtcttctccaa
V7-81_03        ggacaagggcttgagtggatgggatggttcaacacctacactgggaacccaacatatgcccagggcttcacaggacggtttgtcttctccaa
V7-81_04        ggacaagggcttgagtggatgggatggttcaacacctacactgggaacccaacatatgcccagggcttcacaggacggtttgtcttctccaa
V7-81_05        ggacaagggcttgagtggatgggatggttcaacacctacactgggaacccaacatatgcccagggcttcacaggacggtttgtcttctccaa
V7-81_06        ggacaagggcttgagtggatgggatggttcaacacctacactgggaacccaacatatgcccagggcttcacaggatggtttgtcttctcct
V7-81_07        ggacaagggcttgagtggatgggatggttcaacacctacactgggaacccaacatatgcccagggcttcacaggacggtttgtcttctccaa
IGHV7-81*01     ggacaagggcttgagtggatgggatggttcaacacctacactgggaacccaacatatgcccagggcttcacaggacggtttgtcttctccaa
IGHV7-4-1*02    ggacaagggcttgagtggatgggatggatcaacaccaacactgggaacccaacgtatgcccagggcttcacaggacggtttgtcttctcct
IGHV7-4-1*01    ggacaagggcttgagtggatgggatggatcaacaccaacactgggaacccaacgtatgcccagggcttcacaggacggtttgtcttctcct
IGHV7-4-1*03    ggacaagggcttgagtggatgggatggatcaacaccaacactgggaacccaacgtatgcccagggcttcacaggacggtttgtcttctcct
IGHV7-4-1*04    ggacaagggcttgagtggatgggatggatcaacaccaacactgggaacccaacgtatgcccagggcttcacaggacggtttgtcttctcct
IGHV7-4-1*05    ggacaagggcttgagtggatgggatggatcaacaccaacactgggaacccaacgtatgcccagggcttcacaggacggtttgtcttctcct
V_V7-81*01      ggacaagggcttgagtggatgggatggttcaacacctacactgggaacccaacatatgcccagggcttcacaggacggtttgtcttctccaa

V7-81_01        tggacacctctgccagcacagcatacctgcagatcagcagcctaaaggctgaggacatggccatgtattactgtgcgaga
V7-81_02        tggacacctctgccagcatagcatacatgcagatcagcagcctaaaggctgaggacatggccatgtattactgtgcgaga
V7-81_03        tggacacctctgccagcacagcatacctgcagatcagcagcctaaaggctgaggacatggccatgtattactgtgcgaga
V7-81_04        tggacacctctgccagcacagcatacctgcagatcagcagcctaaaggctgaggacatggccatgtattactgtgcgaga
V7-81_05        tggacacctctgccagcatagcatacatgcagatcagcagcctaaaggctgaggacatggccatgtattactgtgcgaga
V7-81_06        tggacacctctgccagcacagcatacctgcagatcagcagcctaaaggctgaggacatggccatgtattactgtgcgaga
V7-81_07        tggacacctctgccagcatagcatacatgcagatcagcagcctaaaggctgaggacatggccatgtattactgtgcgaga
IGHV7-81*01     tggacacctctgccagcacagcatacctgcagatcagcagcctaaaggctgaggacatggccatgtattactgtgcgaga
IGHV7-4-1*02    tggacacctctgtcagcacggcatatctgcagatcagcagcctaaaggctgaggacactgccgtgtattactgtgcgaga
IGHV7-4-1*01    tggacacctctgtcagcacggcatatctgcagatctgcagcctaaaggctgaggacactgccgtgtattactgtgcgaga
IGHV7-4-1*03    tggacacctctgtcagcacggcatatctgcagatcagcacgctaaaggctgaggacactg--------------------
IGHV7-4-1*04    tggacacctctgtcagcatggcatatctgcagatcagcagcctaaaggctgaggacactgccgtgtattactgtgcgaga
IGHV7-4-1*05    tggacacctctgtcagcatggcatatctgcagatcagcagcctaaaggctgaggacactgccgtgtgttactgtgcgaga
V_V7-81*01      tggacacctctgccagcacagcatacctgcagatcagcagcctaaaggctgaggacatggccatgtattactgtgcgaga
```

IGKV1-5

```
V1-5_01        atggacatgagggtccccgctcagctcctggggctcctgctgctctggctcccaggtgccaaatgtgacatccagatgacccagtctcct
V1-5_02        atggacatgagggtccccgctcagctcctggggctcctgctgctctggctcccaggtgccaaatgtgacatccagatgacccagtctcct
V1-5_03        atggacatgagggtccccgctcagctcctggggctcctgctgctctggctcccaggtgccaaatgtgacatccagatgacccagtctcct
IGKV1-5*01     atggacatgagggtccccgctcagctcctggggctcctgctgctctggctcccaggtgccaaatgtgacatccagatgacccagtctcct
IGKV1-5*02     atggacatgagggtccccgctcagctcctggggctcctgctgctctggctcccaggtgccaaatgtgacatccagatgacccagtctcct
IGKV1-5*03     ------------------------------------------------------------------gacatccagatgacccagtctcct
IP_V1-5*p04    ------------------------------------------------------------------gacatccagatgacccagtctcct
IP_V1-5*p05    ------------------------------------------------------------------gacatccagatgacccagtctcct
IP_V1-5*p06    ------------------------------------------------------------------gacatccagatgacccagtctcct
IP_V1-5*p07    ------------------------------------------------------------------gacatccagatgacccagtctcct
IP_V1-5*p08    ------------------------------------------------------------------gacatccagatgacccagtctcct
IP_V1-5*p09    ------------------------------------------------------------------gacatccagatgacccagtctcct
IP_V1-5*p10    ------------------------------------------------------------------gacatccagatgacccagtctcct
V_V1-5*01      ------------------------------------------------------------------gacatccagatgacccagtctcct
V_V1-5*02      ------------------------------------------------------------------gacatccagatgacccagtctcct
V_V1-5*03      ------------------------------------------------------------------gacatccagatgacccagtctcct

V1-5_01        tccaccctgtctgcatctgtaggagacagagtcaccatcacttgccgggccagtcagagtattagtagctggttggcctggtatcagcag
V1-5_02        tccaccctgtctgcatctgtaggagacagagtcaccatcacttgccgggccagtcagagtattagtagctggttggcctggtatcagcag
V1-5_03        tccaccctgtctgcatctgtaggagacagagtcaccatcacttgccgggccagtcagagtattagtagctggttggcctggtatcagcag
IGKV1-5*01     tccaccctgtctgcatctgtaggagacagagtcaccatcacttgccgggccagtcagagtattagtagctggttggcctggtatcagcag
IGKV1-5*02     tccaccctgtctgcatctgtaggagacagagtcaccatcatttgccgggccagtcagagtattagtagctggttggcctggtatcagcag
IGKV1-5*03     tccaccctgtctgcatctgtaggagacagagtcaccatcacttgccgggccagtcagagtattagtagctggttggcctggtatcagcag
IP_V1-5*p04    tccaccctgtctgcatctgtaggagacagagtcaccatcacttgccgggccagtcagagtattagtagctggttggcctggtatcagcag
IP_V1-5*p05    tccaccctgtctgcatctgtaggagacagagtcaccatcacttgccgggccagtcagagtattagtagctggttggcctggtatcagcag
IP_V1-5*p06    tccaccctgtctgcatctgtaggagacagagtcaccatcacttgccgggccagtcagagtattagtagctggttggcctggtatcagcag
IP_V1-5*p07    tccaccctgtctgcatctgtaggagacagagtcaccatcacttgccgggccagtcagagtattagtagctggttggcctggtatcagcag
IP_V1-5*p08    tccaccctgtctgcatctgtaggagacagagtcaccatcacttgccgggccagtcagagtattagtagctggttggcctggtatcagcag
IP_V1-5*p09    tccaccctgtctgcatctgtaggagacagagtcaccatcacttgccgggccagtcagagtattagtagctggttggcctggtatcagcag
IP_V1-5*p10    tccaccctgtctgcatctgtaggagacagagtcaccatcacttgccgggccagtcagagtattagtagctggttggcctggtatcagcag
V_V1-5*01      tccaccctgtctgcatctgtaggagacagagtcaccatcacttgccgggccagtcagagtattagtagctggttggcctggtatcagcag
V_V1-5*02      tccaccctgtctgcatctgtaggagacagagtcaccatcatttgccgggccagtcagagtattagtagctggttggcctggtatcagcag
V_V1-5*03      tccaccctgtctgcatctgtaggagacagagtcaccatcacttgccgggccagtcagagtattagtagctggttggcctggtatcagcag
```

```
V1-5_01         aaaccagggaaagcccctaagctcctgatctatgatgcctccagtttggaaagtggggtcccatcaaggttcagcggcagtggatctggg
V1-5_02         aaaccagggaaagcccctaagctcctgatctataaggcgtctagtttagaaagtggggtcccatcaaggttcagcggcagtggatctggg
V1-5_03         aaaccagggaaagcccctaagctcctgatctataaggcatctagtttagaaagtggggtcccatcaaggttcagcggcagtggatctggg
IGKV1-5*01      aaaccagggaaagcccctaagctcctgatctatgatgcctccagtttggaaagtggggtcccatcaaggttcagcggcagtggatctggg
IGKV1-5*02      aaaccagggaaagcccctaagctcctgatctatgatgcctccagtttggaaagtggggtcccatcaaggttcagcggcagtggatctggg
IGKV1-5*03      aaaccagggaaagcccctaagctcctgatctataaggcgtctagtttagaaagtggggtcccatcaaggttcagcggcagtggatctggg
IP_V1-5*p04     aaaccagggaaagcccctaagctcctgatctataaggcgtctagtttagaaagtggggtcccatcaaggttcagcggcagtggatctggg
IP_V1-5*p05     aaaccagggaaagcccctaagctcctgatctataaggcatctagtttagaaagtggggtcccatcaaggttcagcggcagtggatctggg
IP_V1-5*p06     aaaccagggaaagcccctaagctcctgatctataaggcatctagtttagaaagtggggtcccatcaaggttcagcggcagtggatctggg
IP_V1-5*p07     aaaccagggaaagcccctaagctcctgatctataaggcgtctagtttagaaagtggggtcccatcaaggttcagcggcagtggatctggg
IP_V1-5*p08     aaaccagggaaagcccctaagctcctgatctataaggcgtctagtttagaaagtggggtcccatcaaggttcagcggcagtggatctggg
IP_V1-5*p09     aaaccagggaaagcccctaagctcctgatctataaggcgtctagtttagaaagtggggtcccatcaaggttcagcggcagtggatctggg
IP_V1-5*p10     aaaccagggaaagcccctaagctcctgatctataaggcgtctagtttagaaagtggggtcccatcaaggttcagcggcagtggatctggg
V_V1-5*01       aaaccagggaaagcccctaagctcctgatctatgatgcctccagtttggaaagtggggtcccatcaaggttcagcggcagtggatctggg
V_V1-5*02       aaaccagggaaagcccctaagctcctgatctatgatgcctccagtttggaaagtggggtcccatcaaggttcagcggcagtggatctggg
V_V1-5*03       aaaccagggaaagcccctaagctcctgatctataaggcgtctagtttagaaagtggggtcccatcaaggttcagcggcagtggatctggg

V1-5_01         acagaattcactctcaccatcagcagcctgcagcctgatgattttgcaacttattactgccaacagtataatagttattct--
V1-5_02         acagaattcactctcaccatcagcagcctgcagcctgatgattttgcaacttattactgccaacagtataatagttattct--
V1-5_03         acagaattcactctcaccatcagcagcctgcagcctgatgattttgcaacttattactgccaacagtataatagttattct--
IGKV1-5*01      acagaattcactctcaccatcagcagcctgcagcctgatgattttgcaacttattactgccaacagtataatagttattctcc
IGKV1-5*02      acagaattcactctcaccatcagcagcctgcagcctgatgattttgcaacttattactgccaacagtataatagttattctcc
IGKV1-5*03      acagaattcactctcaccatcagcagcctgcagcctgatgattttgcaacttattactgccaacagtataatagttattctcc
IP_V1-5*p04     acagaattcactctcaccatcagcagcctgcagcctgatgattttgcaacttattactgccaacagtataatagtttctctcc
IP_V1-5*p05     acagaattcactctcaccatcagcagcctgcagcctgatgattttgcaacttattactgccaacagtataatagttattctcc
IP_V1-5*p06     acagaattcactctcaccatcagcagcctgcagcctgatgattttgcaacttattactgccaacagtataatagtttctctcc
IP_V1-5*p07     acagaattcactctcaccatcagcagcctgcagcctgatgattttgcaacttattactgccaacagccccggagtttctctcc
IP_V1-5*p08     acagaattcactctcaccatcagcagcctgcagcctgatgattttgcaacttattactgccaacagtataatagctattctcc
IP_V1-5*p09     acagaattcactctcaccatcagcagcctgcagcctgatgattttgcaacttattactgccaacagtataatagtatctctcc
IP_V1-5*p10     acagaattcactctcaccatcagcagcctgcagcctgatgattttgcaacttattactgccaacagtataatagttactctcc
V_V1-5*01       acagaattcactctcaccatcagcagcctgcagcctgatgattttgcaacttattactgccaacagtataatagttat-----
V_V1-5*02       acagaattcactctcaccatcagcagcctgcagcctgatgattttgcaacttattactgccaacagtataatagttat-----
V_V1-5*03       acagaattcactctcaccatcagcagcctgcagcctgatgattttgcaacttattactgccaacagtataatagttat-----
```

IGKV1-6

```
V1-6_01        atggacatgagggtccccgctcagctcctggggctcctgctgctctggctcccaggtgccagatgtgccatccagatgacccagtctccatc
V1-6_02        atggacatgagggtccccgctcagctcctggggctcctgctgctctggctcccaggtgccagatgtgccatccaaatgacccagtctccatc
V1-6_03        atggacatgagggtccccgctcagctcctggggctcctgctgctctggctcccaggtgccagatgtgccatccagatgacccagtctccatc
V1-6_04        atggacatgagggtccccgctcagctcctggggctcctgctgctctggctcccaggtgccagatgtgccatccagatgacccagtctccatc
IGKV1-6*01     atggacatgagggtccccgctcagctcctggggctcctgctgctctggctcccaggtgccagatgtgccatccagatgacccagtctccatc
IGKV1-6*02     ------atgagggtccccgctcagctcctggggctcctgctgctctggctcccaggtgccagatgtgccatccagatgacccagtctccatc
V_V1-6*01      ------------------------------------------------------------------gccatccagatgacccagtctccatc

V1-6_01        ctccctgtctgcatctgtaggagacagagtcaccatcacttgccgggcaagtcagggcattagaaatgatttaggctggtatcagcag
V1-6_02        ctccctgtctgcatctgtaggagacagagtcaccatcacttgccgggcaagtcagggcattagaaatgatttaggctggtatcagcag
V1-6_03        ctccctgtctgcatctgtaggagacagagtcaccatcacttgccgggcaagtcagggcattagaaatgatttaggctggtatcagcag
V1-6_04        ctccctgtctgcatctgtaggagacagagtcaccatcacttgccgggcaagtcagggcattagaaatgatttaggctggtatcagcag
IGKV1-6*01     ctccctgtctgcatctgtaggagacagagtcaccatcacttgccgggcaagtcagggcattagaaatgatttaggctggtatcagcag
IGKV1-6*02     ctccctgtctgcatctgttggagacagagtcaccatcacttgccgggcaagtcagggcattagaaatgatttaggctggtatcagcag
V_V1-6*01      ctccctgtctgcatctgtaggagacagagtcaccatcacttgccgggcaagtcagggcattagaaatgatttaggctggtatcagcag

V1-6_01        aaaccagggaaagcccctaagctcctgatctatgctgcatccagtttacaaagtggggtcccatcaaggttcagcggcagtggatctggc
V1-6_02        aaaccagggaaagcccctaagctcctgatctatgctgcatccagtttacaaagtggggtcccatcaaggttcagcggcagtggatctggc
V1-6_03        aaaccagggaaagcccctaagctcctgatctatgctgcatccagtttacaaagtggggtcccatcaaggttcagcggcagtggatctggg
V1-6_04        aaaccagggaaagcccctaagctcctgatctatactgcatccagtttacaaagtggggtcccatcaaggttcagcggcagtggatctggc
IGKV1-6*01     aaaccagggaaagcccctaagctcctgatctatgctgcatccagtttacaaagtggggtcccatcaaggttcagcggcagtggatctggc
IGKV1-6*02     aaaccagggaaagcccctaagctcctgatctatgctgcatccagtttacaaagtggggtcccatcaaggttcagcggcagtggatctggc
V_V1-6*01      aaaccagggaaagcccctaagctcctgatctatgctgcatccagtttacaaagtggggtcccatcaaggttcagcggcagtggatctggc

V1-6_01        acagatttcactctcaccatcagcagcctgcagcctgaagattttgcaacttattactgtctacaagattacaattaccct
V1-6_02        acagatttcactctcaccatcagcagcctgcagcctgaagattttgcaacttattactgtctacaagattacaattaccct
V1-6_03        acagatttcactctcaccatcagcagcctgcagcctgaagattttgcaacttattactgtctacaagattacaattaccct
V1-6_04        acagatttcactctcaccatcagcagcctgcagcctgaagattttgcaacttattactgtctacaagattacaattaccct
IGKV1-6*01     acagatttcactctcaccatcagcagcctgcagcctgaagattttgcaacttattactgtctacaagattacaattaccct
IGKV1-6*02     acagatttcactctcaccatcagcagcctgcagcctgaagattttgcaacttattactgtctacaagattacaattaccct
V_V1-6*01      acagatttcactctcaccatcagcagcctgcagcctgaagattttgcaacttattactgtctacaagattacaattac---
```

IGKV1-8

```
V1-8_01          atgagggtccccgctcagctcctggggctcctgctgctctggctcccaggtgccagatgtgccatccggatgacccagtctccatcctca
V1-8_02          atgagggtccccgctcagctcctggggctcctgctgctctggctcccaggtgccagatgtgccatccggataacccagtctccatcctca
V1-8_03          atgagggtccccgctcagctcctggggctcctgctgctctggctcccaggtgccagatgtgccatccggatgacccagtctccatcctca
V1-8_04          atgagggtccccgctcagctcctggggctcctgctgctctggctcccaggtgccagatgtgccatccggatgacccagtctccatcctca
IGKV1-8*01       atgagggtccccgctcagctcctggggctcctgctgctctggctcccaggtgccagatgtgccatccggatgacccagtctccatcctca
IP_V1-8*p02      ------------------------------------------------------------gccatccggataacccagtctccatcctca
IP_V1-8*p03      ------------------------------------------------------------gccatccggatgacccagtctccatcctca
V_V1-8*01        ------------------------------------------------------------gccatccggatgacccagtctccatcctca

V1-8_01          ttctctgcatctacaggagacagagtcaccatcacttgtcgggcgagtcagggtattagcagttatttagcctggtatcagcaaaaacca
V1-8_02          ctctctgcatctacaggagacagagtcaccatcacttgtcgggcgagtcagggtattagcagttatttagcctggtatcagcaaaaacca
V1-8_03          ctctctgcatctacaggagacagagtcaccatcacttgtcgggcgagtcagggtattagcagttatttagcctggtatcagcaaaaacca
V1-8_04          ctctctgcatctacaggagacagagtcaccatcacttgtcgggcgagtcagggtattagcagttatttagcctggtatcagcaaaaacca
IGKV1-8*01       ttctctgcatctacaggagacagagtcaccatcacttgtcgggcgagtcagggtattagcagttatttagcctggtatcagcaaaaacca
IP_V1-8*p02      ctctctgcatctacaggagacagagtcaccatcacttgtcgggcgagtcagggtattagcagttatttagcctggtatcagcaaaaacca
IP_V1-8*p03      ctctctgcatctacaggagacagagtcaccatcacttgtcgggcgagtcagggtattagcagttatttagcctggtatcagcaaaaacca
V_V1-8*01        ttctctgcatctacaggagacagagtcaccatcacttgtcgggcgagtcagggtattagcagttatttagcctggtatcagcaaaaacca

V1-8_01          gggaaagcccctaagctcctgatctatgctgcatccactttgcaaagtggggtcccatcaaggttcagcggcagtggatctgggacagattt
V1-8_02          gggaaagcccctaagctcctgatctatgctgcatccactttgcaaagtggggtcccatcaaggttcagcggcagtggatctgggacagattt
V1-8_03          gggaaagcccctaagctcctgatctatgctgcatccactttgcaaagtggggtcccatcaaggttcagcggcagtggatctgggacagattt
V1-8_04          gggaaagcccctaagctcctgatctatgctgcatccactttgcaaagtggggtcccatcaaggttcagtggcagtggatctgggacagattt
IGKV1-8*01       gggaaagcccctaagctcctgatctatgctgcatccactttgcaaagtggggtcccatcaaggttcagcggcagtggatctgggacagattt
IP_V1-8*p02      gggaaagcccctaagctcctgatctatgctgcatccactttgcaaagtggggtcccatcaaggttcagcggcagtggatctgggacagattt
IP_V1-8*p03      gggaaagcccctaagctcctgatctatgctgcatccactttgcaaagtggggtcccatcaaggttcagcggcagtggatctgggacagattt
V_V1-8*01        gggaaagcccctaagctcctgatctatgctgcatccactttgcaaagtggggtcccatcaaggttcagcggcagtggatctgggacagattt

V1-8_01          cactctcaccatcagctgcctgcagtctgaagattttgcaacttattactgtcaacagtattatagttaccct--
V1-8_02          cactctcaccatcagctgcctgcagtctgaagattttgcaacttattactgtcaacagtattatagttaccct--
V1-8_03          cactctcaccatcagctgcctgcagtctgaagattttgcaacttattactgtcaacagtattatagttaccct--
V1-8_04          cactctcaccatcagctgcctgcagtctgaagattttgcaacttattactgtcaacagtattatagttaccct--
IGKV1-8*01       cactctcaccatcagctgcctgcagtctgaagattttgcaacttattactgtcaacagtattatagttaccctcc
IP_V1-8*p02      cactctcaccatcagctgcctgcagtctgaagattttgcaacttattactgtcaacagtattatagttaccctcc
IP_V1-8*p03      cactctcaccatcagctgcctgcagtctgaagattttgcaacttattactgtcaacagtattatagttaccctcc
V_V1-8*01        cactctcaccatcagctgcctgcagtctgaagattttgcaacttattactgtcaacagtattatagttac-----
```

IGKV1-9

```
V1-9_01       atgagggtccccgctcagctcctggggctcctgctgctctggctcccaggtgccagatgtgacatccagttgacccagtctccatc
V1-9_02       atgagggtccccgctcagctcctggggctcctgctgctctggctcccaggtgccagatgtgccatccagttgacccagtctccatc
V1-9_03       atgagggtccccgctcagctcctggggctcctgctgctctggctcccaggtgccagatgtgccatccagttgacccagtctccatc
V1-9_04       atgagggtccccgctcagctcctggggctcctgctgctctggctcccaggtgccagatgtgacatccagttgacccagtctccatc
V1-9_05       atgagggtccccgctcagctcctggggctcctgctgctctggctcccaggtgccagatgtgacatccagttgacccagtctccatc
IGKV1-9*01    atgagggtccccgctcagctcctggggctcctgctgctctggctcccaggtgccagatgtgacatccagttgacccagtctccatc
IP_V1-9*p02   ------------------------------------------------------------gccatccagttgacccagtctccatc
V_V1-9*01     ------------------------------------------------------------gacatccagttgacccagtctccatc

V1-9_01       cttcctgtctgcatctgtaggagacagagtcaccatcacttgccgggccagtcagggcattagcagttatttagcctggtatcagcaa
V1-9_02       ctccctgtctgcatctgtaggagacagagtcaccatcacttgccgggccagtcagggcattagcagttatttagcctggtatcagcaa
V1-9_03       cttcctgtctgcatctgtaggagacagagtcaccatcacttgccgggccagtcagggcattagcagttatttagcctggtatcagcaa
V1-9_04       cttcctgtctgcatctgtaggagacagagtcaccatcacttgctgggccagtcagggcattagcagttatttagcctggtatcagcaa
V1-9_05       cttcctgtctgcatctgtaggagacagagtcaccatcacttgccgggcaagtcagggcattagcagttatttagcctggtatcagcaa
IGKV1-9*01    cttcctgtctgcatctgtaggagacagagtcaccatcacttgccgggccagtcagggcattagcagttatttagcctggtatcagcaa
IP_V1-9*p02   ctccctgtctgcatctgtaggagacagagtcaccatcacttgccgggccagtcagggcattagcagttatttagcctggtatcagcaa
V_V1-9*01     cttcctgtctgcatctgtaggagacagagtcaccatcacttgccgggccagtcagggcattagcagttatttagcctggtatcagcaa

V1-9_01       aaaccagggaaagcccctaagctcctgatctatgctgcatccactttgcaaagtggggtcccatcaaggttcagcggcagtggatctgg
V1-9_02       aaaccagggaaagcccctaagctcctgatctatgctgcatccactttgcaaagtggggtcccatcaaggttcagcggcagtggatctgg
V1-9_03       aaaccagggaaagcccctaagctcctgatctatgctgcatccactttgcaaagtggggtcccatcaaggttcagcggcagtggatctgg
V1-9_04       aaaccagggaaagcccctaagctcctgatctatgctgcatccactttgcaaagtggggtcccatcaaggttcagcggcagtggatctgg
V1-9_05       aaaccagggaaagcccctaagctcctgatctatgctgcatccactttgcaaagtggggtcccatcaaggttcagcggcagtggatctgg
IGKV1-9*01    aaaccagggaaagcccctaagctcctgatctatgctgcatccactttgcaaagtggggtcccatcaaggttcagcggcagtggatctgg
IP_V1-9*p02   aaaccagggaaagcccctaagctcctgatctatgctgcatccactttgcaaagtggggtcccatcaaggttcagcggcagtggatctgg
V_V1-9*01     aaaccagggaaagcccctaagctcctgatctatgctgcatccactttgcaaagtggggtcccatcaaggttcagcggcagtggatctgg

V1-9_01       gacagaattcactctcacaatcagcagcctgcagcctgaagattttgcaacttattactgtcaacagcttaatagttaccct
V1-9_02       gacagatttcactctcaccatcagcagcctgcagcctgaagattttgcaacttattactgtcaacagcttaatagttaccct
V1-9_03       gacagaattcactctcacaatcagcagcctgcagcctgaagattttgcaacttattactgtcaacagcttaatagttaccct
V1-9_04       gacagaattcactctcacaatcagcagcctgcagcctgaagattttgcaacttattactgtcaacagcttaatagttaccct
V1-9_05       gacagaattcactctcacaatcagcagcctgcagcctgaagattttgcaacttattactgtcaacagcttaatagttaccct
IGKV1-9*01    gacagaattcactctcacaatcagcagcctgcagcctgaagattttgcaacttattactgtcaacagcttaatagttaccct
IP_V1-9*p02   gacagatttcactctcaccatcagcagcctgcagcctgaagattttgcaacttattactgtcaacagcttaatagttaccct
V_V1-9*01     gacagaattcactctcacaatcagcagcctgcagcctgaagattttgcaacttattactgtcaacagcttaatagttac---
```

IGKV1-12

```
V1-12_01      ------atgagggtccccgctcagctcctggggctcctgctgctctggttcccaggttccagatgcgacatccagatgacccagtctcca
V1-12_02      ------atgagggtccccgctcagctcctggggctcctgctgctctggttcccaggttccagatgcgacatccagatgacccagtctcca
V1-12_03      ------atgagggtccccgctcagctcctggggctcctgctgctctggttcccaggttccagatgcgacatccagatgacccagtctcca
V1-12_04      ------atgagggtccccgctcagctcctggggctcctgctgctctggttcccaggttccagatgcgacatccagatgacccagtctcca
IGKV1-12*01   atggacatgagggtccccgctcagctcctggggctcctgctgctctggttcccaggttccagatgcgacatccagatgacccagtctcca
IGKV1-12*02   atggacatgatggtccccgctcagctcctggggctcctgctgctctggttcccaggttccagatgcgacatccagatgacccagtctcca
V_V1-12*02    -------------------------------------------------------------------gacatccagatgacccagtctcca
                                                                                 ***********************

V1-12_01      tcttccgtgtctgcatctgtaggagacagagtcaccatcacttgtcgggcgagtcagggtattagcagctggttagcctggtatcagcag
V1-12_02      tcttccgtgtctgcatctgtaggagacagagtcaccatcacttgtcaggcgagtcagggtattagcagctggttagcctggtatcagcag
V1-12_03      tcttccgtgtctgcatctgtaggagacagagtcaccatcacttgtcgggcgagtcagggtattagcagctggttagcctggtatcagcag
V1-12_04      tcttccgtgtctgcatctgtaggagacagagtcatcatcacttgtcgggcgagtcagggtattagcagctggttagcctggtatcagcag
IGKV1-12*01   tcttccgtgtctgcatctgtaggagacagagtcaccatcacttgtcgggcgagtcagggtattagcagctggttagcctggtatcagcag
IGKV1-12*02   tcttccgtgtctgcatctgtaggagacagagtcaccatcacttgtcgggcgagtcagggtattagcagctggttagcctggtatcagcag
V_V1-12*02    tcttccgtgtctgcatctgtaggagacagagtcaccatcacttgtcgggcgagtcagggtattagcagctggttagcctggtatcagcag

V1-12_01      aaaccagggaaagcccctaagctcctgatctatgctgcatccagtttgcaaagtggggtcccatcaaggttcagcggcagtggatctg
V1-12_02      aaaccagggaaagcccctaagctcctgatctatgctgcatccagtttgcaaagtggggtcccatcaaggttcagcggcagtggatctg
V1-12_03      aaaccagggaaagcccctaagctcctgatctatgctgcatccagtttgcaaagtggggtcccatcaaggttcagcggcggtggatctg
V1-12_04      aaaccagggaaagcccctaagctcctgatctatgctgcatccagtttgcaaagtggggtcccatcaaggttcagcggcagtggatctg
IGKV1-12*01   aaaccagggaaagcccctaagctcctgatctatgctgcatccagtttgcaaagtggggtcccatcaaggttcagcggcagtggatctg
IGKV1-12*02   aaaccagggaaagcccctaagctcctgatctatgctgcatccagtttgcaaagtggggtcccatcaaggttcagcggcagtggatctg
V_V1-12*02    aaaccagggaaagcccctaagctcctgatctatgctgcatccagtttgcaaagtggggtcccatcaaggttcagcggcagtggatctg

V1-12_01      ggacagatttcactctcaccatcagcagcctgcagcctgaagattttgcaacttactattgtcaacaggctaacagtttccct
V1-12_02      ggacagatttcactctcaccatcagcagcctgcagcctgaagattttgcaacttactattgtcaacaggctaacagtttccct
V1-12_03      ggacagatttcactctcaccatcagcagcctgcagcctgaagattttgcaacttactattgtcaacaggctaacagtttccct
V1-12_04      ggacagatttcactctcaccatcagcagcctgcagcctgaagattttgcaacttactattgtcaacaggctaacagtttccct
IGKV1-12*01   ggacagatttcactctcaccatcagcagcctgcagcctgaagattttgcaacttactattgtcaacaggctaacagtttccct
IGKV1-12*02   ggacagatttcactctcaccatcagcagcctgcagcctgaagattttgcaacttactattgtcaacaggctaacagtttccct
V_V1-12*02    ggacagatttcactctcaccatcagcagcctgcagcctgaagattttgcaacttactattgtcaacaggctaacagtttc---
```

IGKV1-13

```
V1-13_01         atggacatgagggtccccgctcagctcctggggcttctgctgctctggctcccaggtgccagatgtgccatccagttgacccagtct
V1-13_02         atggacatgagggtccccgctcagctcctggggcttctgctgctctggctcccaggtgccagatgtgccatccagttgacccagtct
V1-13_03         atggacatgagggtccccgctcagctcctggggcttctgctgctctggctcccaggtgccagatgtgccatccagttgacccagtct
V1-13_04         atggacatgagggtccccgctcagctcctggggctcctgctgctctggctcccaggtgccagatgtgccatccagttgacccagtct
V1-13_05         atggacatgagggtccccgctcagctcctggggcttctgctgctctggctcccaggtgccagatgtgccatccagttgacccagtct
IGKV1-13*01      atggacatgagggtccccgctcagctcctggggcttctgctgctctggctccca-ggtgccagatgtgccatccagttgacccagtct
IGKV1-13*02      atggacatgagggtccccgctcagctcctggggcttctgctgctctggctccca-ggtgccagatgtgccatccagttgacccagtct
V_V1-13*01(P)    -------------------------------------------------------------gccatccagttgacccagtct

V1-13_01         ccatcctccctgtctgcatctgtaggagacagagtcaccatcacttgccgggcaagtcagggcattagcagtgctttagcctgaatatcagca
V1-13_02         ccatcctccctgtctgcatctgtaggagacagagtcaccatcacttgccgggcaagtcagggcattagcagtgctttagcctgaatatcagca
V1-13_03         ccatcctccctgtctgcatctgtaggagacagagtcaccatcacttgccgggcaagtcagggcattagcagtgctttagcctgaatatcagca
V1-13_04         ccatcctccctgtctgcatctgtaggagacagagtcaccatcacttgccgggcaagtcagggcattagcagtgctttagcctgaatatcagca
V1-13_05         ccatcctccctgtctgcatctgtaggagacagagtcaccatcacttgccaggcaagtcagggcattagcagtgctttagcctgaatatcagca
IGKV1-13*01      ccatcctccctgtctgcatctgtaggagacagagtcaccatcacttgccgggcaagtcagggcattagcagtgctttagcctgaatatcagca
IGKV1-13*02      ccatcctccctgtctgcatctgtaggagacagagtcaccatcacttgccgggcaagtcagggcattagcagtgctttagcctggtatcagca
V_V1-13*01(P)    ccatcctccctgtctgcatctgtaggagacagagtcaccatcacttgccgggcaagtcagggcattagcagtgctttagcctgaatatcagca

V1-13_01         gaaaccagggaaagctcctaagctcctgatctatgatgcctccagtttggaaagtggggtcccatcaaggttcagcggcagtggatctggg
V1-13_02         gaaaccagggaaagctcctaagctcctgatctatgatgcctccagtttggaaagtggggtcccatcaaggttcagcggcagtggatctggg
V1-13_03         gaaaccagggaaagctcctaagctcctgatctatgatgcctccagtttggaaagtggggtcccatcaaggttcagcggcagtggatctggc
V1-13_04         gaaaccagggaaagctcctaagctcctgatctatgatgcctccagtttggaaagtggggtcccatcaaggttcagcggcagtggatctggg
V1-13_05         gaaaccagggaaagctcctaagctcctgatctatgatgcctccagtttggaaagtggggtcccatcaaggttcagcggcagtggatctggg
IGKV1-13*01      gaaaccagggaaagctcctaagctcctgatctatgatgcctccagtttggaaagtggggtcccatcaaggttcagcggcagtggatctggg
IGKV1-13*02      gaaaccagggaaagctcctaagctcctgatctatgatgcctccagtttggaaagtggggtcccatcaaggttcagcggcagtggatctggg
V_V1-13*01(P)    gaaaccagggaaagctcctaagctcctgatctatgatgcctccagtttggaaagtggggtcccatcaaggttcagcggcagtggatctggg

V1-13_01         acagatttcactctcaccatcagcagcctgcagcctgaagattttgcaacttattactgtcaacagtttaataatt
V1-13_02         acagatttcactctcaccatcagcagcctgcagcctgaagattttgcaacttattactgtcaacagtttaatagtt
V1-13_03         acagatttcactctcaccatcagcagcctgcagcctgaagattttgcaacttattactgtcaacagtttaataatt
V1-13_04         acagatttcactctcaccatcagcagcctgcagcctgaagattttgcaacttattactgtcaacagtttaataatt
V1-13_05         acagatttcactctcaccatcagcagcctgcagcctgaagattttgcaacttattactgtcaacagtttaataatt
IGKV1-13*01      acagatttcactctcaccatcagcagcctgcagcctgaagattttgcaacttattactgtcaacagtttaataatt
IGKV1-13*02      acagatttcactctcaccatcagcagcctgcagcctgaagattttgcaacttattactgtcaacagtttaatagtt
V_V1-13*01(P)    acagatttcactctcaccatcagcagcctgcagcctgaagattttgcaacttattactgtcaacagtttaataatt
```

IGKV1-16

```
V1-16_01      atggacatgagagtcctcgctcagctcctggggctcctgctgctctgtttcccaggtgccagatgtgacatccagatgacccagtctccat
V1-16_02      atggacatgagagtcctcgctcagctcctggggctcctgctgctctgtttctcaggtgccagatgtgacatccagatgacccagtctccat
V1-16_03      atggacatgagagtcctcgctcagctcctggggctcctgctgctctgtttcccaggtgccagatgtgacatccagatgacccagtctccat
IGKV1-16*01   atggacatgagagtcctcgctcagctcctggggctcctgctgctctgtttcccaggtgccagatgtgacatccagatgacccagtctccat
IGKV1-16*02   ------------------------------------------------------------------gacatccagatgacccagtctccat
V_V1-16*01    ------------------------------------------------------------------gacatccagatgacccagtctccat

V1-16_01      cctcactgtctgcatctgtaggagacagagtcaccatcacttgtcgggcgagtcagggcattagcaattatttagcctggtttcagcag
V1-16_02      cctcactgtctgcatctgtaggagacagagtcaccatcacttgtcgggcgagtcagggcattagcaattatttagcctggtttcagcag
V1-16_03      cctcactgtctgcatctgtaggagacagagtcaccatcacttgtcgggcgagtcagggcattagcaattatttagcctggtttcagcag
IGKV1-16*01   cctcactgtctgcatctgtaggagacagagtcaccatcacttgtcgggcgagtcagggcattagcaattatttagcctggtttcagcag
IGKV1-16*02   cctcactgtctgcatctgtaggagacagagtcaccatcacttgtcgggcgagtcagggcattagcaattatttagcctggtttcagcag
V_V1-16*01    cctcactgtctgcatctgtaggagacagagtcaccatcacttgtcgggcgagtcagggcattagcaattatttagcctggtttcagcag

V1-16_01      aaaccagggaaagcccctaagtccctgatctatgctgcatccagtttgcaaagtggggtcccatcaaagttcagcggcagtggatctgg
V1-16_02      aaaccagggaaagcccctaagtccctgatctatgctgcatccagtttgcaaagtggggtcccatcaaagttcagcggcagtggatctgg
V1-16_03      aaaccagggaaagtccctaagtccctgatctatgctgcatccagtttgcaaagtggggtcccatcaaagttcagcggcagtggatctgg
IGKV1-16*01   aaaccagggaaagcccctaagtccctgatctatgctgcatccagtttgcaaagtggggtcccatcaaggttcagcggcagtggatctgg
IGKV1-16*02   aaaccagggaaagcccctaagtccctgatctatgctgcatccagtttgcaaagtggggtcccatcaaagttcagcggcagtggatctgg
V_V1-16*01    aaaccagggaaagcccctaagtccctgatctatgctgcatccagtttgcaaagtggggtcccatcaaggttcagcggcagtggatctgg

V1-16_01      gacagatttcactctcaccatcagcagcctgcagcctgaagattttgcaacttattactgccaacagtataatagttaccct
V1-16_02      gacagatttcactctcaccatcagcagcctgcagcctgaagattttgcaacttattactgccaacagtataatagttaccct
V1-16_03      gacagatttcactctcaccatcagcagcctgcagcctgaagattttgcaacttattactgccaacagtataatagttaccct
IGKV1-16*01   gacagatttcactctcaccatcagcagcctgcagcctgaagattttgcaacttattactgccaacagtataatagttaccct
IGKV1-16*02   gacagatttcactctcaccatcagcagcctgcagcctgaagattttgcaacttattactgccaacagtataatagttaccct
V_V1-16*01    gacagatttcactctcaccatcagcagcctgcagcctgaagattttgcaacttattactgccaacagtataatagttac---
```

IGKV1-17

```
V1-17_01       atggacatgagggtccccgctcagctcctggggctcctgctgctctggttcccaggtgccaggtgtgacatccagatgacccagtctccatc
V1-17_02       atggacatgagggtccccgctcagctcctggggctcctgctgctctggttcccaggtgccaggtgtgacatccagatgacccagtctccatc
V1-17_03       atggacatgagggtccccgctcagctcctggggctcctgctgctctggttcccaggtgccaggtgtgacatccagatgacccagtctccatc
V1-17_04       atggacatgagggtccccgctcagctcctggggctcctgctgctctggttcccaggtgccaggtgtgacatccagatgacccagtctccatc
V1-17_05       atggacatgagggtccccgctcagctcctggggctcctgctgctctggttcccaggtgccaggtgtgacatccagatgacccagtctccatc
V1-17_06       atggacatgagggtccccgctcagctcctggggctcctgctgctctggttcccaggtgccaggtgtgacatccagatgacccagtctccatc
V1-17_07       atggacatgagggtccccgctcagctcctggggctcctgctgctctggttcccaggtgccaggtgtgacatccagatgacccagtctccatc
V1-17_08       atggacatgagggtccccgctcagctcctggggctcctgctgctctggttcccaggtgccaggtgtgacatccagatgacccagtctccatc
IGKV1-17*01    atggacatgagggtccccgctcagctcctggggctcctgctgctctggttcccaggtgccaggtgtgacatccagatgacccagtctccatc
IGKV1-17*02    ------atgagggtccccgctcagctcctggggctcctgctgctctggttcccaggtgccaggtgtgacatccagatgacccagtctccatc
IGKV1-17*03    ------atgagggtccccgctcagctcctggggctcctgctgctctggttcccaggtgccaggtgtgacatccagatgacccagtctccatc
V_V1-17*01     ------------------------------------------------------------------gacatccagatgacccagtctccatc

V1-17_01       ctccctgtctgcatctgtaggagacagagtcaccatcacttgccgggcaagtcagggcattagaaatgatttaggctggtatcagcag
V1-17_02       tgccatgtctgcatctgtaggagacagagtcaccatcacttgtcgggcgagtcagggcattagcaattatttagcctggttcagcag
V1-17_03       ctccctgtctgcatctgtaggagacagagtcaccatcacttgccgggcaagtcagggcattagaaatgatttaggctggtatcagcag
V1-17_04       tgccatgtctgcatctgtaggagacagagtcaccatcacttgtcgggcgagtcagggcattagcaattatttagcctggttcagcag
V1-17_05       tgccatgtctgcatctgtaggagacagagtcaccatcacttgccgggcaagtcagggcattagaaatgatttaggctggtatcagcag
V1-17_06       tgccatgtctgcatctgtaggagacagagtcaccatcacttgccgggcaagtcagggcattagaaatgatttaggctggtatcagcag
V1-17_07       ctccctgtctgcatctgtaggagacagagtcaccatcacttgtcgggcgagtcagggcattagcaattatttagcctggttcagcag
V1-17_08       ctccctgtctgcatctgtaggagacagagtcaccatcacttgccgggcaagtcagggcattagaaatgatttaggctggtatcagcag
IGKV1-17*01    ctccctgtctgcatctgtaggagacagagtcaccatcacttgccgggcaagtcagggcattagaaatgatttaggctggtatcagcag
IGKV1-17*02    ctccctgtctgcatctgtaggagacagagtcaccatcacttgccgggcaagtcagggcattagaaatgatttaggctggtatcagcag
IGKV1-17*03    tgccatgtctgcatctgtaggagacagagtcaccatcacttgtcgggcgagtcagggcattagcaattatttagcctggttcagcag
V_V1-17*01     ctccctgtctgcatctgtaggagacagagtcaccatcacttgccgggcaagtcagggcattagaaatgatttaggctggtatcagcag

V1-17_01       aaaccagggaaagcccctaagcgcctgatctatgctgcatccagtttgcaaagtggggtcccatcaaggttcagcggcagtggatctg
V1-17_02       aaaccagggaaagtccctaagcgcctgatctatgctgcatccagtttgcaaagtggggtcccatcaaggttcagcggcagtggatctg
V1-17_03       aaaccagggaaagtccctaagcgcctgatctatgctgcatccagtttgcaaagtggggtcccatcaaggttcagcggcagtggatctg
V1-17_04       aaaccagggaaagcccctaagcgcctgatctatgctgcatccagtttgcaaagtggggtcccatcaaggttcagcggcagtggatctg
V1-17_05       aaaccagggaaagcccctaagcgcctgatctatgctgcatccagtttgcaaagtggggtcccatcaaggttcagcggcagtggatctg
V1-17_06       aaaccagggaaagtccctaagcgcctgatctatgctgcatccagtttgcaaagtggggtcccatcaaggttcagcggcagtggatctg
V1-17_07       aaaccagggaaagtccctaagcgcctgatctatgctgcatccagtttgcaaagtggggtcccatcaaggttcagcggcagtggatctg
V1-17_08       aaatcagggaaagcccctaagcgcctgatctatgctgcatccagtttgcaaagtggggtcccatcaaggttcagcggcagtggatctg
IGKV1-17*01    aaaccagggaaagcccctaagcgcctgatctatgctgcatccagtttgcaaagtggggtcccatcaaggttcagcggcagtggatctg
IGKV1-17*02    aaaccagggaaagcccctaagcgcctgatctatgctgcatccagtttgcaaagtggggtcccatcaaggttcagcggcagtggatctg
IGKV1-17*03    aaaccagggaaagtccctaagcgcctgatctatgctgcatccagtttgcaaagtggggtcccatcaaggttcagcggcagtggatctg
V_V1-17*01     aaaccagggaaagcccctaagcgcctgatctatgctgcatccagtttgcaaagtggggtcccatcaaggttcagcggcagtggatctg
```

```
V1-17_01        ggacagaattcactctcacaatcagcagcctgcagcctgaagattttgcaacttattactgtctacagcataatagttaccct--
V1-17_02        ggacagaattcactctcacaatcagcagcctgcagcctgaagattttgcaacttattactgtctacagcataatagttaccct--
V1-17_03        ggacagaattcactctcacaatcagcagcctgcagcctgaagattttgcaacttattactgtctacagcataatagttaccct--
V1-17_04        ggacagaattcactctcacaatcagcagcctgcagcctgaagattttgcaacttattactgtctacagcataatagttaccct--
V1-17_05        ggacagaattcactctcacaatcagcagcctgcagcctgaagattttgcaacttattactgtctacagcataatagttaccct--
V1-17_06        ggacagaattcactctcacaatcagcagcctgcagcctgaagattttgcaacttattactgtctacagcataatagttaccct--
V1-17_07        ggacagaattcactctcacaatcagcagcctgcagcctgaagattttgcaacttattactgtctacagcataatagttaccct--
V1-17_08        ggacagaattcactctcacaatcagcagcctgcagcctgaagattttgcaacttattactgtctacagcataatagttaccct--
IGKV1-17*01     ggacagaattcactctcacaatcagcagcctgcagcctgaagattttgcaacttattactgtctacagcataatagttaccctcc
IGKV1-17*02     ggacagaattcactctcacaatcagcaacctgcagcctgaagattttgcaacttattactgtctacagcataatagttaccctcc
IGKV1-17*03     ggacagaattcactctcacaatcagcagcctgcagcctgaagattttgcaacttattactgtctacagcataatagttaccctcc
V_V1-17*01      ggacagaattcactctcacaatcagcagcctgcagcctgaagattttgcaacttattactgtctacagcataatagttac-----
```

IGKV1-27

```
V1-27_01        atggacatgagggtccctgctcagctcctgggactcctgctgctctggctcccagataccagatgtgacatccagatgacccagtctccat
V1-27_02        atggacatgagggtccctgctcagctcctgggactcctgctgctctggctcccagataccagatgtgacatccagatgacccagtctccat
V1-27_03        atggacatgagggtcccCgctcagctcctgggactcctgctgctctggctcccagataccagatgtgacatccagatgacccagtctccat
V1-27_04        atggacatgagggtccctgctcagctcctgggactcctgctgctctggctcccagataccagatgtgacatccagatgacccagtctccat
V1-27_05        atggacatgagggtccctgctcagctcctgggactcctgctgctctggctcccagataccagatgtgacatccagatgacccagtctccat
V1-27_06        atggacatgagggtccctgctcagctcctgggactcctgctgctctggctcccagataccagatgtgacatccagatgacccagtctccat
IGKV1-27*01     atggacatgagggtccctgctcagctcctgggactcctgctgctctggctcccagataccagatgtgacatccagatgacccagtctccat
IP_V1-27*p02    ------------------------------------------------------------------gacatccagatgacccagtctccat
V_V1-27*01      ------------------------------------------------------------------gacatccagatgacccagtctccat

V1-27_01        cctccctgtctgcatctgtaggagacagagtcaccatcacttgccgggcgagtcagggcattagcaattatttagcctggtatcagcag
V1-27_02        cctccctgtctgcatctgtaggagacagagtcaccatcacttgccgggcgagtcagggcattagcaattatttagcctggtatcagcag
V1-27_03        cctccctgtctgcatctgtaggagacagagtcaccatcacttgccgggcgagtcagggcattagcaattatttagcctggtatcagcag
V1-27_04        cctccctgtctgcatctgtaggagacagagtcaccatcacttgccgggcgagtcagggcattagcaattatttagcctggtatcagcag
V1-27_05        cctccctgtctgcatctgtaggagacagagtcaccatcacttgccgggcgagtcagggcattagcaattatttagcctggtatcagcag
V1-27_06        cctccctgtctgcatctgtaggagacagagtcaccatcacttgccgggcgagtcagggcattagcaattatttagcctggtatcagcag
IGKV1-27*01     cctccctgtctgcatctgtaggagacagagtcaccatcacttgccgggcgagtcagggcattagcaattatttagcctggtatcagcag
IP_V1-27*p02    cctccctgtctgcatctgtaggagacagagtcaccatcacttgccgggcgagtcagggcattagcaattatttagcctggtatcagcag
V_V1-27*01      cctccctgtctgcatctgtaggagacagagtcaccatcacttgccgggcgagtcagggcattagcaattatttagcctggtatcagcag

V1-27_01        aaaccagggaaagttcctaagctcctgatctatgctgcatccactttgcaatcaggggtcccatctcggttcagtggcagtggatctgg
V1-27_02        aaaccagggaaagttcctaagctcctgatctatgctgcatccactttgcaatcaggggtcccatctcggttcagcggcagtggatctgg
V1-27_03        aaaccagggaaagttcctaagctcctgatctatgctgcatccactttgcaatcaggggtcccatctcggttcagtggcagtggatctgg
V1-27_04        aaaccagggaaagttcctaagctcctgatctatgctgcatccgctttgcaatcaggggtcccatctcggttcagtggcagtggatctgg
V1-27_05        aaaccagggaaagttcctaagctcctgatctatgctgcatccgctttgcaatcaggggtcccatctcggttcagcggcagtggatctgg
V1-27_06        aaaccagggaaagttcctaagctcctgatctatgctgcatccactttgcaatcaggggtcccatctcggttcagtggcagtggatctgg
IGKV1-27*01     aaaccagggaaagttcctaagctcctgatctatgctgcatccactttgcaatcaggggtcccatctcggttcagtggcagtggatctgg
IP_V1-27*p02    aaaccagggaaagttcctaagctcctgatctatgctgcatccactttgcaatcaggggtcccatctcggttcagcggcagtggatctgg
V_V1-27*01      aaaccagggaaagttcctaagctcctgatctatgctgcatccactttgcaatcaggggtcccatctcggttcagtggcagtggatctgg

V1-27_01        gacagatttcactctcaccatcagcagcctgcagcctgaagatgttgcaacttattactgtcaaaagtataacagtgcccct
V1-27_02        gacagatttcactctcaccatcagcagcctgcagcctgaagatgttgcaacttattactgtcaaaagtataacagtgcccct
V1-27_03        gacagatttcactctcaccatcagcagcctgcagcctgaagatgttgcaacttattactgtcaaaagtataacagtgcccct
V1-27_04        gacagatttcactctcaccatcagcagcctgcagcctgaagatgttgcaacttattactgtcaaaagtataacagtgcccct
V1-27_05        gacagatttcactctcaccatcagcagcctgcagcctgaagatgttgcaacttattactgtcaaaagtataacagtgcccct
V1-27_06        gacagatttcactctcaccatcagcagcctgcagcctgaagatgttgcaacttattattgtcaaaagtataacagtgcccct
IGKV1-27*01     gacagatttcactctcaccatcagcagcctgcagcctgaagatgttgcaacttattactgtcaaaagtataacagtgcccct
IP_V1-27*p02    gacagatttcactctcaccatcagcagcctgcagcctgaagatgttgcaacttattactgtcaaaagtataacagtgcccct
V_V1-27*01      gacagatttcactctcaccatcagcagcctgcagcctgaagatgttgcaacttattactgtcaaaagtataacagtgcc---
```

IGKV1-33

```
V1-33_01        atggacatgagggtccctgctcagctcctggggctcctgctgctctggctctcaggtgccagatgtgacatccagatgacccagtctcca
V1-33_02        atggacatgagggtccctgctcagctcctggggctcctgctgctctggctctcaggtgccagatgtgacatccagatgacccagtctcca
IGKV1-33*01     atggacatgagggtccctgctcagctcctggggctcctgcagctctggctctcaggtgccagatgtgacatccagatgacccagtctcca
IP_V1-33*p02    -------------------------------------------------------------------gacatccagatgacccagtctcca
V_V1-33*01      -------------------------------------------------------------------gacatccagatgacccagtctcca

V1-33_01        tcctccctgtctgcatctgtaggagacagagtcaccatcacttgccaggcgagtcaggacattagcaactatttaaattggtatcagcag
V1-33_02        tcctccctgtctgcatctgtaggagacagagtcaccatcacttgccaggcgagtcaggacattagcaactatttaaattggtatcagcag
IGKV1-33*01     tcctccctgtctgcatctgtaggagacagagtcaccatcacttgccaggcgagtcaggacattagcaactatttaaattggtatcagcag
IP_V1-33*p02    tcctccctgtctgcatctgtaggagacagagtcaccatcacttgccaggcgagtcaggacattagcaactatttaaattggtatcagcag
V_V1-33*01      tcctccctgtctgcatctgtaggagacagagtcaccatcacttgccaggcgagtcaggacattagcaactatttaaattggtatcagcag

V1-33_01        aaaccagggaaagcccctaagctcctgatctacgatgcatccaatttggaaacaggggtcccatcaaggttcagtggaagtggatctg
V1-33_02        aaaccagggaaagcccctaagctcctgatctacgatgcatccaatttggaaacaggggtcccatcaaggttcagtggaagtggatctg
IGKV1-33*01     aaaccagggaaagcccctaagctcctgatctacgatgcatccaatttggaaacaggggtcccatcaaggttcagtggaagtggatctg
IP_V1-33*p02    aaaccagggaaagcccctaagctcctgatctacgatgcatccaatttggaaacaggggtcccatcaaggttcagtggaagtggatctg
V_V1-33*01      aaaccagggaaagcccctaagctcctgatctacgatgcatccaatttggaaacaggggtcccatcaaggttcagtggaagtggatctg

V1-33_01        ggacagattttactttcaccatcagcagcctgcagcctgaagatattgcaacatattactgtcaacagtatgataatctccct
V1-33_02        ggacagattttactttcaccatcagcagcctgcagcctgaagatattgcaacatattactatcaacagtatgataatctccct
IGKV1-33*01     ggacagattttactttcaccatcagcagcctgcagcctgaagatattgcaacatattactgtcaacagtatgataatctccct
IP_V1-33*p02    ggacagattttactttcaccatcagcagcctgcagcctgaagatattgcaacatattactgtcaacagtatgacaactaccct
V_V1-33*01      ggacagattttactttcaccatcagcagcctgcagcctgaagatattgcaacatattactgtcaacagtatgataatctc---
```

IGKV1-37

```
V1-37_01       atggacatgagggtccccgctcagctcctggggctcctactgctctgggtcccaggtgccagatgtgacatccagttgacccagtctcca
V1-37_02       atggacatgagggtccccgctcagctcctggggctcctactgctctgggtcccaggtgccagatgtgacatccagttgacccagtctcca
IGKV1-37*01    atggacatgagggtccccgctcagctcctggggctcctactgctctgggtcccaggtgccagatgtgacatccagttgacccagtctcca

V1-37_01       tcctccctgtctgcatctgtaggagacagagtcaccatcacttgccgggtgagtcagggcattagcagttatttaaattggtatcggcag
V1-37_02       tcctccctgtctgcatctgtaggagacagagtcaccatcacttgccgggtgagtcagggcattagcagttatttaaattggtatcggcag
IGKV1-37*01    tcctccctgtctgcatctgtaggagacagagtcaccatcacttgccgggtgagtcagggcattagcagttatttaaattggtatcggcag

V1-37_01       aaaccagggaaagttcctaagctcctgatctatagtgcatccaatttgcaatctggagtcccatctcggttcagtggcagtggatctggg
V1-37_02       aaaccagggaaagttcctaagctcctgatctatagtgcatccaatttgcaatctggagtcccatctcggttcagtggcagtggatctggg
IGKV1-37*01    aaaccagggaaagttcctaagctcctgatctatagtgcatccaatttgcaatctggagtcccatctcggttcagtggcagtggatctggg

V1-37_01       acagatttcactctcactatcagcagcctgcagcctgaagatgttgcaacttattacggtcaacggacttacaatgcccct
V1-37_02       acagatttcactctcactatcagcagcctgcagcctgaagatgttgcaacttattacggtcaacagacttacaatgcccct
IGKV1-37*01    acagatttcactctcactatcagcagcctgcagcctgaagatgttgcaacttattacggtcaacggacttacaatgcccct
```

IGKV1-39

```
V1-39_01          atggacatgagggtccccgctcagctcctggggctcctgctactctggctccgaggtgccagatgtgacatccagatgacccagtctcc
IGKV1-39*01       atggacatgagggtccccgctcagctcctggggctcctgctactctggctccgaggtgccagatgtgacatccagatgacccagtctcc
IGKV1-39*02       atggacatgagggtccccgctcagctcctggggctcctgctactctggctctgaggtgccagatgtgacatccagatgacccagtctcc
IP_V1-39*p03      ------------------------------------------------------------------gacatccagatgacccagtctcc
IP_V1-39*p04      ------------------------------------------------------------------gacatccagatgacccagtctcc
V_V1-39*02(P)     ------------------------------------------------------------------gacatccagatgacccagtctcc
V_V1-39*01        ------------------------------------------------------------------gacatccagatgacccagtctcc

V1-39_01          atcctccctgtctgcatctgtaggagacagagtcaccatcacttgccgggcaagtcagagcattagcagctatttaaattggtatcagcag
IGKV1-39*01       atcctccctgtctgcatctgtaggagacagagtcaccatcacttgccgggcaagtcagagcattagcagctatttaaattggtatcagcag
IGKV1-39*02       atccttcctgtctgcatctgtaggagacagagtcaccatcacttgccgggcaagtcagagcattagcagctatttaaattggtatcagcag
IP_V1-39*p03      atcctccctgtctgcatctgtaggagacagagtcaccatcacttgccgggcaagtcagagcattagcagctatttaaattggtatcagcag
IP_V1-39*p04      atcctccctgtctgcatctgtaggagacagagtcaccatcacttgccgggcaagtcagagcattagcagctatttaaattggtatcagcag
V_V1-39*02(P)     atccttcctgtctgcatctgtaggagacagagtcaccatcacttgccgggcaagtcagagcattagcagctatttaaattggtatcagcag
V_V1-39*01        atcctccctgtctgcatctgtaggagacagagtcaccatcacttgccgggcaagtcagagcattagcagctatttaaattggtatcagcag

V1-39_01          aaaccagggaaagcccctaagctcctgatctatgctgcatccagtttgcaaagtggggtcccatcaaggttcagtggcagtggatctg
IGKV1-39*01       aaaccagggaaagcccctaagctcctgatctatgctgcatccagtttgcaaagtggggtcccatcaaggttcagtggcagtggatctg
IGKV1-39*02       aaaccagggaaagcccctaagctcctgatctatgctgcatccagtttgcaaagtggggtcccatcaaggttcagtggcagtggatctg
IP_V1-39*p03      aaaccagggaaagcccctaagctcctgatctatgctgcatccagtttgcaaagtggggtcccatcaaggttcagtggcagtggatctg
IP_V1-39*p04      aaaccagggaaagcccctaagctcctgatctatgctgcatccagtttgcaaagtggggtcccatcaaggttcagtggcagtggatctg
V_V1-39*02(P)     aaaccagggaaagcccctaagctcctgatctatgctgcatccagtttgcaaagtggggtcccatcaaggttcagtggcagtggatctg
V_V1-39*01        aaaccagggaaagcccctaagctcctgatctatgctgcatccagtttgcaaagtggggtcccatcaaggttcagtggcagtggatctg

V1-39_01          ggacagatttcactctcaccatcagcagtctgcaacctgaagattttgcaacttactactgtcaacagagttacagtacccctcc
IGKV1-39*01       ggacagatttcactctcaccatcagcagtctgcaacctgaagattttgcaacttactactgtcaacagagttacagtacccctcc
IGKV1-39*02       ggacagatttcactctcaccatcagcagtctgcaacctgaagattttgcaacttattactgtcagtgtggttacagtacacctcc
IP_V1-39*p03      ggacagatttcactctcaccatcagcagtctgcaacctgaagattttgcaacttactactgtcaacagagttacactctccctcc
IP_V1-39*p04      ggacagatttcactctcaccatcagcagtctgcaacctgaagattttgcaacttactactgtcaacagagatacaccttccctcc
V_V1-39*02(P)     ggacagatttcactctcaccatcagcagtctgcaacctgaagattttgcaacttattactgtcagtgtggttacagtaca-----
V_V1-39*01        ggacagatttcactctcaccatcagcagtctgcaacctgaagattttgcaacttactactgtcaacagagttacagtacc-----
```

IGKV1D-8

```
V1D-8_01        atggacatgagggtccccgctcagctcctggggctcctgctgctctggctcccaggtgccagatgtgtcatctggatgacccagtctccat
V1D-8_02        atggacatgagggtccccgctcagctcctggggctcctgctgctctggctcccaggtgccagatgtgccatctggatgacccagtctccat
V1D-8_03        atggacatgagggtccccgctcagctcctggggctcctgctgctctggctcccaggtgccagatgtgtcatctggatgacccagtctccat
V1D-8_04        atggacatgagggtccccgctcagctcctggggctcctgctgctctggctcccaggtgccagatgtgtcatctggatgacccagtctccat
V1D-8_05        atggacatgagggtccccgctcagctcctggggctcctgctgctctggctcccaggtgccagatgtgtcatctggatgacccagtctccat
V1D-8_06        atggacatgagggtccccgctcagctcctggggctcctgctgctctggctcccaggtgccagatgtgtcatctggatgacctagtctccat
IGKV1D-8*01     atggacatgagggtccccgctcagctcctggggctcctgctgctctggctcccaggtgccagatgtgtcatctggatgacccagtctccat
IGKV1D-8*02     ------atgagggtccccgctcagctcctggggctcctgctgctctggctcccaggtgccagatgtgccatctggatgacccagtctccat
IGKV1D-8*03     atggacatgagggtccccgctcagctcctggggctcctgctgctctggctcccaggtgccagatgtgtcatctggatgacccagtctccat
V_V1D-8*01      ------------------------------------------------------------------gtcatctggatgacccagtctccat
```

```
V1D-8_01        ccttactctctgcatctacaggagacagagtcaccatcagttgtcggatgagtcagggcattagcagttatttagcctggtatcagcaa
V1D-8_02        ccttactctctgcatctacaggagacagagtcaccatcagttgtcggatgagtcagggcattagcagttatttagcctggtatcagcaa
V1D-8_03        ccttactctctgcatctacaggagacagagtcaccatcagttgtcggatgagtcagggcattagcagttatttagcctggtatcagcaa
V1D-8_04        ccttactctctgcatctacaggagacagagtcaccatcagttgtcggatgagtcagggcattagcagttatttagcctggtatcagcaa
V1D-8_05        ccttactctctgcatctacaggagacagagtcatcatcagttgtcggatgagtcagggcattagcagttatttagcctggtatcagcaa
V1D-8_06        ccttactctctgcatctacaggagacagagtcaccatcagttgtcggatgagtcagggcattagcagttatttagcctggtatcagcaa
IGKV1D-8*01     ccttactctctgcatctacaggagacagagtcaccatcagttgtcggatgagtcagggcattagcagttatttagcctggtatcagcaa
IGKV1D-8*02     ccttactctctgcatctacaggagacagagtcaccatcagttgtcggatgagtcagggcattagcagttatttagcctggtatcagcaa
IGKV1D-8*03     ccttactctctgcatctacaggagacagagtcaccatcagttgtcggatgagtcagggcattagcagttatttagcctggtatcagcaa
V_V1D-8*01      ccttactctctgcatctacaggagacagagtcaccatcagttgtcggatgagtcagggcattagcagttatttagcctggtatcagcaa
```

```
V1D-8_01        aaaccagggaaagcccctgagctcctgatctatgctgcatccactttgcaaagtggggtcccatcaaggttcagtggcagtggatctgg
V1D-8_02        aaaccagggaaagcccctgagctcctgatctatgctgcatccactttgcaaagtggggtcccatcaaggttcagtggcagtggatctgg
V1D-8_03        aaaccagggaaagcccctgagctcctgatctatgctgcatccactttgcaaagtggggtcccatcaaggttcagtggcagtggatctgg
V1D-8_04        aaaccagggaaagcccctgagctcctgatctatgctgcatccactttgcaaagtggggtcccatcaaggttcagtggcagtggatctgg
V1D-8_05        aaaccagggaaagcccctgagctcctgatctatgctgcatccactttgcaaagtggggtcccatcaaggttcagtggcagtggatctgg
V1D-8_06        aaaccagggaaagcccctgagctcctgatctatgctgcatccactttgcaaagtggggtcccatcaaggttcagtggcagtggatctgg
IGKV1D-8*01     aaaccagggaaagcccctgagctcctgatctatgctgcatccactttgcaaagtggggtcccatcaaggttcagtggcagtggatctgg
IGKV1D-8*02     aaaccagggaaagcccctgagctcctgatctatgctgcatccactttgcaaagtggggtcccatcaaggttcagtggcagtggatctgg
IGKV1D-8*03     aaaccagggaaagcccctgagctcctgatctatgctgcatccactttgcaaagtggggtcccatcaaggttcagtggcagtggatctgg
V_V1D-8*01      aaaccagggaaagcccctgagctcctgatctatgctgcatccactttgcaaagtggggtcccatcaaggttcagtggcagtggatctgg
```

```
V1D-8_01        gacagatttcactctcaccatcagttgcctgcagtctgaagattttgcaacttattactgtcaacagtattatagtttccct--
V1D-8_02        gacagatttcactctcaccatcagctgcctgcagtctgaagattttgcaacttattactgtcaacagtattatagtttccct--
V1D-8_03        gacagatttcactctcaccatcagctgcctgcagtctgaagattttgcaacttattactgtcaacagtattatagtttccct--
V1D-8_04        gacagatttcattctcaccatcagttgcctgcagtctgaagattttgcaacttattactgtcaacagtattatagtttccct--
V1D-8_05        gacagatttcactctcaccatcagttgcctgcagtctgaagattttgcaacttattactgtcaacagtattatagtttccct--
V1D-8_06        gacagatttcactctcaccatcagttgcctgcagtctgaagattttgcaacttattactgtcaacagtattatagtttccct--
IGKV1D-8*01     gacagatttcactctcaccatcagttgcctgcagtctgaagattttgcaacttattactgtcaacagtattatagtttccctcc
IGKV1D-8*02     gacagatttcactctcaccatcagctgcctgcagtctgaagattttgcaacttattactgtcaacagtattatagtttccctcc
IGKV1D-8*03     gacagatttcactctcaccatcagctgcctgcagtctgaagattttgcaacttattactgtcaacagtattatagtttccctcc
V_V1D-8*01      gacagatttcactctcaccatcagttgcctgcagtctgaagattttgcaacttattactgtcaacagtattatagtttc-----
```

IGKV1D-12

```
V1D-12_01       atggacatgagggtccccgctcagctcctggggctcctgctgctctggttcccaggttccagatgcgacatccagatgacccagtctccatc
V1D-12_02       atggacatgagggtccccgctcagctcctgaggctcctgctgctctggttcccaggttccagatgcgacatccagatgacccagtctccatc
V1D-12_03       atggacatgagggtccccgctcagctcctggggctcctgctgctctggttcccaggttccagatgcgacatccagatgacccagtctccatc
IGKV1D-12*01    atggacatgagggtccccgctcagctcctggggctcctgctgctctggttcccaggttccagatgcgacatccagatgacccagtctccatc
IGKV1D-12*02    atggacatgatggtccccgctcagctcctggggctcctgctgctctggttcccaggttccagatgcgacatccagatgacccagtctccatc
V_V1D-12*01     -------------------------------------------------------------------gacatccagatgacccagtctccatc

V1D-12_01       ttctgtgtctgcatctgtaggagacagagtcaccatcacttgtcgggcgagtcagggtattagcagctggttagcctggtatcagcag
V1D-12_02       ttctgtgtctgcatctgtaggagacagagtcaccatcacttgtcgggcgagtcagggtattagcagctggttagcctggtatcagcag
V1D-12_03       ttctgtgtctgcatctgtaggagacagagtcaccatcacttgtcgggcgagtcagggtattagcagctggttagcctggtattagcag
IGKV1D-12*01    ttctgtgtctgcatctgtaggagacagagtcaccatcacttgtcgggcgagtcagggtattagcagctggttagcctggtatcagcag
IGKV1D-12*02    ttccgtgtctgcatctgtaggagacagagtcaccatcacttgtcgggcgagtcagggtattagcagctggttagcctggtatcagcag
V_V1D-12*01     ttctgtgtctgcatctgtaggagacagagtcaccatcacttgtcgggcgagtcagggtattagcagctggttagcctggtatcagcag

V1D-12_01       aaaccagggaaagcccctaagctcctgatctatgctgcatccagtttgcaaagtggggtcccatcaaggttcagcggcagtggatctggg
V1D-12_02       aaaccagggaaagcccctaagctcctgatctatgctgcatccagtttgcaaagtggggtcccatcaaggttcagcggcagtggatctggg
V1D-12_03       aaaccagggaaagcccctaagctcctgatctatgctgcatccagtttgcaaagtggggtcccatcaaggttcagcggcagtggatctggg
IGKV1D-12*01    aaaccagggaaagcccctaagctcctgatctatgctgcatccagtttgcaaagtggggtcccatcaaggttcagcggcagtggatctggg
IGKV1D-12*02    aaaccagggaaagcccctaagctcctgatctatgctgcatccagtttgcaaagtggggtcccatcaaggttcagcggcagtggatctggg
V_V1D-12*01     aaaccagggaaagcccctaagctcctgatctatgctgcatccagtttgcaaagtggggtcccatcaaggttcagcggcagtggatctggg

V1D-12_01       acagatttcactctcactatcagcagcctgcagcctgaagattttgcaacttactattgtcaacaggctaacagtttccct
V1D-12_02       acagatttcactctcactatcagcagcctgcagcctgaagattttgcaacttactattgtcaacaggctaacagtttccct
V1D-12_03       acagatttcactctcactatcagcagcctgcagcctgaagattttgcaacttactattgtcaacaggctaacagtttccct
IGKV1D-12*01    acagatttcactctcactatcagcagcctgcagcctgaagattttgcaacttactattgtcaacaggctaacagtttccct
IGKV1D-12*02    acagatttcactctcaccatcagcagcctgcagcctgaagattttgcaacttactattgtcaacaggctaacagtttccct
V_V1D-12*01     acagatttcactctcactatcagcagcctgcagcctgaagattttgcaacttactattgtcaacaggctaacagtttc---
```

IGKV1D-13

```
V1D-13_01      atggacatgagggtccccgctcagctcctggggcttctgctgctctggctcccaggtgccagatgtgccatccagttgacccagtctcca
V1D-13_02      atggacatgagggtccccgctcagctcctggggcttctgctgctctggctcccaggtgccagatgtgccatccagttgacccagtctcca
V1D-13_03      atggacatgagggtccccgctcagctcctggggcttctgctgctctggctcccaggtgccagatgtgccatccagttgacccagtctcca
V1D-13_04      atggacatgagggtccccactcagctcctggggcttctgctgctctggctcccaggtgccagatgtgccatccagttgacccagtctcca
V1D-13_05      atggacatgagggtccccgctcagctcctggggcttctgctgctctggctcccaggtgccagatgtgccatccagttgacccagtctcca
IGKV1D-13*01   atggacatgagggtccccgctcagctcctggggcttctgctgctctggctcccaggtgccagatgtgccatccagttgacccagtctcca
IGKV1D-13*02   ------atgagggtccccgctcagctcctggggcttctgctgctctggctcccaggtgccagatgtgccatccagttgacccagtctcca
V_V1D-13*01    ------------------------------------------------------------------gccatccagttgacccagtctcca

V1D-13_01      tcctccctgtctgcatctgtaggagacagagtcaccatcacttgccgggcaagtcagggcattagcagtgctttagcctggtatcagcag
V1D-13_02      tcctccctgtctgcatctgtaggagacagagtcaccatcacttgccgggcaagtcagggcattagcagtgctttagcctggtatcagcag
V1D-13_03      tcctccctgtctgcatctgtaggagacagagtcaccatcacttgccgggcaagtcagggcattagcagtgctttagcctggtatcagcag
V1D-13_04      tcctccctgtctgcatctgtaggagacagagtcaccatcacttgccgggcaagtcagggcattagcagtgctttagcctggtatcagcag
V1D-13_05      tcctccctgtctgcatctgtaggagacagagtcaccatcacttgccgggcaagtcagggcattagcagtgctttagcctggtatcagcag
IGKV1D-13*01   tcctccctgtctgcatctgtaggagacagagtcaccatcacttgccgggcaagtcagggcattagcagtgctttagcctggtatcagcag
IGKV1D-13*02   tcctccctgtctgcatctgtaggagacagagtcaccatcacttgccgggcaagtcagggcattagcagtgctttagcctggtatcagcag
V_V1D-13*01    tcctccctgtctgcatctgtaggagacagagtcaccatcacttgccgggcaagtcagggcattagcagtgctttagcctggtatcagcag

V1D-13_01      aaaccagggaaagctcctaagctcctgatctatgatgcctccagtttggaaagtggggtcccatcaaggttcagcggcagtggatctggg
V1D-13_02      aaaccagggaaagctcctaagctcctgatctatgatgcctccagtttggaaagtggggtcccatcaaggttcagcggcagtggatctggg
V1D-13_03      aaaccagggaaagctcctaagctcctgatctatgatgcctccagtttggaaagtggggtcccatcaaggttcagcggcagtaatctggg
V1D-13_04      aaaccagggaaagctcctaagctcctgatctatgatgcctccagtttggaaagtggggtcccatcaaggttcagcggcagtggatctggg
V1D-13_05      aaaccagggaaagctcctaagctcctgatctatgatgcctccagtttggaaagtggggtcccatcaaggttcagtggcagtggatctggg
IGKV1D-13*01   aaaccagggaaagctcctaagctcctgatctatgatgcctccagtttggaaagtggggtcccatcaaggttcagcggcagtggatctggg
IGKV1D-13*02   aaaccagggaaagctcctaagctcctgatctatgatgcctccagtttggaaagtggggtcccatcaaggttcagcggcagtggatctggg
V_V1D-13*01    aaaccagggaaagctcctaagctcctgatctatgatgcctccagtttggaaagtggggtcccatcaaggttcagcggcagtggatctggg

V1D-13_01      acagatttcactctcaccatcagcagcctgcagcctgaagattttgcaacttattactgtcaacagtttaataattaccct
V1D-13_02      acagatttcactctcaccatcagcagcctgcagcctgaagattttgcaacttattactgtcaacagtttaataattaccct
V1D-13_03      acagatttcactctcaccatcagcagcctgcagcctgaagattttgcaacttattactgtcaacagtttaataattaccct
V1D-13_04      acagatttcactctcaccatcagcagcctgcagcctgaagattttgcaacttattactgtcaacagtttaataattaccct
V1D-13_05      acagatttcactctcaccatcagcagcctgcagcctgaagattttgcaacttattactgtcaacagtttaataattaccct
IGKV1D-13*01   acagatttcactctcaccatcagcagcctgcagcctgaagattttgcaacttattactgtcaacagtttaataattaccct
IGKV1D-13*02   acagatttcactctcaccatcagcagcctgcagcctgaagattttgcaacttattactgtcaacagtttaatagttaccct
V_V1D-13*01    acagatttcactctcaccatcagcagcctgcagcctgaagattttgcaacttattactgtcaacagtttaataattac---
```

IGKV1D-16

```
V1D-16_01      atggacatgagggtcctcgctcagctcctggggctcctgctgctctgtttcccaggtgccagatgtgacatccagatgacccagtctcc
V1D-16_02      atggacatgagggtcctcgctcagctcctggggctcctgctgctctgtttcccaggtgccagatgtgacatccagatgacccagtctcc
V1D-16_03      atggacatgagggtcctcgctcagctcctggggctcctgctgctctgtttcccaggtgccagatgtgacatccagatgacccagtctcc
IGKV1D-16*01   atggacatgagggtcctcgctcagctcctggggctcctgctgctctgtttcccaggtgccagatgtgacatccagatgacccagtctcc
IGKV1D-16*02   atggacatgagggtcctcgctcagctcctggggctcctgctgctctgtttcccaggtgccagatgtgacatccagatgacccagtctcc
V_V1D-16*02    ------------------------------------------------------------------gacatccagatgacccagtctcc
V_V1D-16*01    ------------------------------------------------------------------gacatccagatgacccagtctcc

V1D-16_01      atcctcactgtctgcatctgtaggagacagagtcaccatcacttgtcggcgagtcagggtattagcagctggttagcctggtatcagcag
V1D-16_02      atcctcactgtctgcatctgtaggagacagagtcaccatcacttgtcggcgaggcagggtattagcagctggttagcctggtatcagcag
V1D-16_03      atcctcactgtctgcatctgtaggagacagagtcaccatcacttgtcggcgagtcagggtattagcagctggttagcctggtatcagcag
IGKV1D-16*01   atcctcactgtctgcatctgtaggagacagagtcaccatcacttgtcggcgagtcagggtattagcagctggttagcctggtatcagcag
IGKV1D-16*02   atcctcactgtctgcatctgtaggagacagagtcaccatcacttgtcggcgaggcagggtattagcagctggttagcctggtatcagcag
V_V1D-16*02    atcctcactgtctgcatctgtaggagacagagtcaccatcacttgtcggcgaggcagggtattagcagctggttagcctggtatcagcag
V_V1D-16*01    atcctcactgtctgcatctgtaggagacagagtcaccatcacttgtcggcgagtcagggtattagcagctggttagcctggtatcagcag

V1D-16_01      aaaccagagaaagcccctaagtccctgatctatgctgcatccagtttgcaaagtggggtcccatcaaggttcagcggcagtggatctggga
V1D-16_02      aaaccagagaaagcccctaagtccctgatctatgctgcatccagtttgcaaagtggggtcccatcaaggttcagcggcagtggatctggga
V1D-16_03      aaaccagagaaagcccctaagtctctgatctatgctgcatccagtttgcaaagtggggtcccatcaaggttcagcggcagtggatctggga
IGKV1D-16*01   aaaccagagaaagcccctaagtccctgatctatgctgcatccagtttgcaaagtggggtcccatcaaggttcagcggcagtggatctggga
IGKV1D-16*02   aaaccagagaaagcccctaagtccctgatctatgctgcatccagtttgcaaagtggggtcccatcaaggttcagcggcagtggatctggga
V_V1D-16*02    aaaccagagaaagcccctaagtccctgatctatgctgcatccagtttgcaaagtggggtcccatcaaggttcagcggcagtggatctggga
V_V1D-16*01    aaaccagagaaagcccctaagtccctgatctatgctgcatccagtttgcaaagtggggtcccatcaaggttcagcggcagtggatctggga

V1D-16_01      cagatttcactctcaccatcagcagcctgcagcctgaagattttgcaacttattactgccaacagtataatagttaccct
V1D-16_02      cagatttcactctcaccatcagcagcctgcagcctgaagattttgcaacttattactgccaacagtataatagttaccct
V1D-16_03      cagatttcactctcaccatcagcagcctgcagcctgaagattttgcaacttattactgccaacagtataatagttaccct
IGKV1D-16*01   cagatttcactctcaccatcagcagcctgcagcctgaagattttgcaacttattactgccaacagtataatagttaccct
IGKV1D-16*02   cagatttcactctcaccatcagcagcctgcagcctgaagattttgcaacttattactgccaacagtataatagttaccct
V_V1D-16*02    cagatttcactctcaccatcagcagcctgcagcctgaagattttgcaacttattactgccaacagtataatagttac---
V_V1D-16*01    cagatttcactctcaccatcagcagcctgcagcctgaagattttgcaacttattactgccaacagtataatagttac---
```

IGKV1D-17

```
V1D-17_01      atggacatgagggtccccgctcagctcctggggctcctgctgctctggttcccaggtgccagatgtaacatccagatgacccagtctcca
V1D-17_02      atggacatgagggtccccgctcagctcctggggctcctgctgctctggttcccaggtgccagatgtaacatccagatgacccagtctcca
V1D-17_03      atggacatgagggtccctgctcagctcctggggctcctgctgctctggttcccaggtgccagatgtaacatccagatgacccagtctcca
V1D-17_04      atggacatgagggtccctgctcagctcctggggctcctgctgctctggttcccaggtgccagatgtaacatccagatgacccagtctcca
V1D-17_05      atggacatgagggtccccgctcagctcctggggctcctgctgctctggttcccaggtgccagatgtaacatccagatgacccagtctcca
V1D-17_06      atggacatgagggtccccgctcagctcctggggctcctgctgctctggttcccaggtgccagatgtaacatccagatgacccagtctcca
V1D-17_07      atggacatgagggtccccgctcagctcctggggctcctgctgctctggttcccaggtgccagatgtaacatccagatgacccagtctcca
IGKV1D-17*01   atggacatgagggtccccgctcagctcctggggctcctgctgctctggttcccaggtgccagatgtaacatccagatgacccagtctcca
V_V1D-17*01    -------------------------------------------------------------------aacatccagatgacccagtctcca

V1D-17_01      tctgccatgtctgcatctgtaggagacagagtcaccatcacttgtcgggcgaggcagggcattagcaattatttagcctggtttcagcag
V1D-17_02      tctgccatgtctgcatctgtaggagacagagtcaccatcacttgtcgggcgagtcagggcattagcaattatttagcctggtttcagcag
V1D-17_03      tctgccatgtctgcatctgtaggagacagagtcaccatcacttgtcgggcgaggcagggcattagcaattatttagcctggtttcagcag
V1D-17_04      tctgccatgtctgcatctgtaggagacagagtcaccatcacttgtcgggcgagtcagggcattagcaattatttagcctggtttcagcag
V1D-17_05      tctgccatgtctgcatctgtaggagacagagtcaccatcacttgtcgggcgaggcagggcattagcaattatttagcctggtttcagcag
V1D-17_06      tctgccatgtctgcatctgtaggagacagagtcaccatcacttgtcgggcgagtcagggcattagcaattatttagcctggtttcagcag
V1D-17_07      tctgccatgtctgcatctgtaggagacagagtcaccatcacttgtcgggcgaggcagggcattagcaattatttagcctggtttcagcag
IGKV1D-17*01   tctgccatgtctgcatctgtaggagacagagtcaccatcacttgtcgggcgaggcagggcattagcaattatttagcctggtttcagcag
V_V1D-17*01    tctgccatgtctgcatctgtaggagacagagtcaccatcacttgtcgggcgaggcagggcattagcaattatttagcctggtttcagcag

V1D-17_01      aaaccagggaaagtccctaagcacctgatctatgctgcatccagtttgcaaagtggggtcccatcaaggttcagcggcagtggatctggg
V1D-17_02      aaaccagggaaagtccctaagcacctgatctatgctgcatccagtttgcaaagtggggtcccatcaaggttcagcggcagtggatctggg
V1D-17_03      aaaccagggaaagtccctaagcacctgatctatgctgcatccagtttgcaaagtggggtcccatcaaggttcagcggcagtggatctggg
V1D-17_04      aaaccagggaaagtccctaagcacctgatctatgctgcatccagtttgcaaagtggggtcccatcaaggttcagcggcagtggatctggg
V1D-17_05      aaaccagggaaagtccctaagcacctgatctatgctgcatccagtttgcaaagtggggtcccatcaaggttcagcggcagtggatctggg
V1D-17_06      aaaccagggaaagtccctaagcacctgatctatgctgcatccagtttgcaaagtggggtcccatcaaggttcagcggcagtggatctggg
V1D-17_07      aaaccagggaaagtccctaagcacctgatctatgctgcatccagtttgcaaagtggggtcccatcaaggttcagtggcagtggatctggg
IGKV1D-17*01   aaaccagggaaagtccctaagcacctgatctatgctgcatccagtttgcaaagtggggtcccatcaaggttcagcggcagtggatctggg
V_V1D-17*01    aaaccagggaaagtccctaagcacctgatctatgctgcatccagtttgcaaagtggggtcccatcaaggttcagcggcagtggatctggg

V1D-17_01      acagaattcactctcacaatcagcagcctgcagcctgaagattttgcaacttattactgtctacagcataatagttaccct
V1D-17_02      acagaattcactctcacaatcagcagcctgcagcctgaagattttgcaacttattactgtctacagcataatagttaccct
V1D-17_03      acagaattcactctcacaatcagcagcctgcagcctgaagattttgcaacttattactgtctacagcataatagttaccct
V1D-17_04      acagaattcactctcacaatcagcagcctgcagcctgaagattttgcaacttattactgtctacagcataatagttaccct
V1D-17_05      acagaattcactctcacaatcagcagcctgcagcctgatgattttgcaacttattactgtctacagcataatagttaccct
V1D-17_06      acagaattcactctcacaatcagcagcctgcagcctgatgattttgcaacttattactgtctacagcataatagttaccct
V1D-17_07      acagaattcactctcacaatcagcagcctgcagcctgaagattttgcaacttattactgtctacagcataatagttaccct
IGKV1D-17*01   acagaattcactctcacaatcagcagcctgcagcctgaagattttgcaacttattactgtctacagcataatagttaccct
V_V1D-17*01    acagaattcactctcacaatcagcagcctgcagcctgaagattttgcaacttattactgtctacagcataatagttac---
```

IGKV1D-33

```
V1D-33_01       atggacatgagggtccctgctcagctcctggggctcctgctgctctggctctcaggtgccagatgtgacatccagatgacccagtctc
V1D-33_02       atggacatgagggtccctgctcagctcctggggctcctgctgctctggctctcaggtgccagatgtgacatccagatgacccagtctc
IGKV1D-33*01    atggacatgagggtccctgctcagctcctggggctcctgctgctctggctctcaggtgccagatgtgacatccagatgacccagtctc

V1D-33_01       catcctccctgtctgcatctgtaggagacagagtcaccatcacttgccaggcgagtcaggacattagcaactatttaaattggtatcagcag
V1D-33_02       catcctccctgtctgcatctgtaggagacagagtcaccatcacttgccaggcgagtcaggacattagcaactatttaaattggtatcagcag
IGKV1D-33*01    catcctccctgtctgcatctgtaggagacagagtcaccatcacttgccaggcgagtcaggacattagcaactatttaaattggtatcagcag

V1D-33_01       aaaccagggaaagcccctaagctcctgatctacgatgcatccaatttggaaacaggggtcccatcaaggttcagtggaagtggatctggg
V1D-33_02       aaaccagggaaagcccctaagctcctgatctacgatgcatccaatttggaaacaggggtcccatcaaggttcagtggaagtggatctggg
IGKV1D-33*01    aaaccagggaaagcccctaagctcctgatctacgatgcatccaatttggaaacaggggtcccatcaaggttcagtggaagtggatctggg

V1D-33_01       acagattttactttcaccatcagcagcctgcagcctgaagatattgcaacatattactgtcaacagtatgataatctccct
V1D-33_02       acagattttactttcaccatcagcagcctgcaggctgaagatattgcaacatattactgtcaacagtatgataatctccct
IGKV1D-33*01    acagattttactttcaccatcagcagcctgcagcctgaagatattgcaacatattactgtcaacagtatgataatctccct
```

IGKV1D-37

```
V1D-37_01       atggacatgagggtccccgctcagctcctggggctcctactgctctgggtcccaggtgccagatgtgacatccagttgacccagtctc
V1D-37_02       atggacatgagggtccccgctcagctcctggggctcctactgctctgggtcccaggtgccagatgtgacatccagttgacccagtctc
V1D-37_03       atggacatgagggtccccgctcagctcctggggctcctactgctctgggtcccaggtgccagatgtgacatccagttgacccagtctc
IGKV1D-37*01    atggacatgagggtccccgctcagctcctggggctcctactgctctgggtcccaggtgccagatgtgacatccagttgacccagtctc
V_V1D-37*01     ------------------------------------------------------------------gacatccagttgacccagtctc

V1D-37_01       catcctccctgtctgcatctgtaggagacagagtcaccatcacttgccgggtgagtcagggcattagcagttatttaaattggtatcggcag
V1D-37_02       catcctccctgtctgcatctgtaggagacagagtcaccatcacttgccgggtgagtcagggcattagcagttatttaaattggtatcggcag
V1D-37_03       catcctccctgtctgcatctgtaggagacagagtcaccatcacttgccgggtgagtcagggcattagcagttatttaaattggtatcggcag
IGKV1D-37*01    catcctccctgtctgcatctgtaggagacagagtcaccatcacttgccgggtgagtcagggcattagcagttatttaaattggtatcggcag
V_V1D-37*01     catcctccctgtctgcatctgtaggagacagagtcaccatcacttgccgggtgagtcagggcattagcagttatttaaattggtatcggcag

V1D-37_01       aaaccagggaaagttcctaagctcctgatctatagtgcatccaatttgcaatctggagtcccatctcggttcagtggcagtggatctg
V1D-37_02       aaaccagggaaagttcctaagctcctgatctatagtgcatccagtttgcaatctggagtcccatctcggttcagtggcagtggatctg
V1D-37_03       aaaccagggaaagttcctaagctcctgatctatagtgcatccaatttgcaatctggagtcccatctcggttcagtggcagtggatctg
IGKV1D-37*01    aaaccagggaaagttcctaagctcctgatctatagtgcatccaatttgcaatctggagtcccatctcggttcagtggcagtggatctg
V_V1D-37*01     aaaccagggaaagttcctaagctcctgatctatagtgcatccaatttgcaatctggagtcccatctcggttcagtggcagtggatctg

V1D-37_01       ggacagatttcactctcactatcagcagcctgcagcctgaagatgttgcaacttattacggtcaacggacttacaatgcccctcc
V1D-37_02       ggacagatttcactctcactatcagcagcctgcagcctgaagatgttgcaacttattacggtcaacggacttacaatgcccctcc
V1D-37_03       ggacagatttcactctcactatcagcagcctgcagcctgaagatgttgcaacttattacggtcaacggacttacaacgcccctcc
IGKV1D-37*01    ggacagatttcactctcactatcagcagcctgcagcctgaagatgttgcaacttattacggtcaacggacttacaatgcccctcc
V_V1D-37*01     ggacagatttcactctcactatcagcagcctgcagcctgaagatgttgcaacttattacggtcaacggacttacaatgcc-----
```

IGKV1D-39

```
V1D-39_01        atggacatgagggtccccgctcagctcctggggctcctgctactctggctccgaggtgccagatgtgacatccagatgacccagtctc
IGKV1D-39*01     atggacatgagggtccccgctcagctcctggggctcctgctactctggctccgaggtgccagatgtgacatccagatgacccagtctc

V1D-39_01        catcctccctgtctgcatctgtaggagacagagtcaccatcacttgccgggcaagtcagagcattagcagctatttaaattggtatcagcag
IGKV1D-39*01     catcctccctgtctgcatctgtaggagacagagtcaccatcacttgccgggcaagtcagagcattagcagctatttaaattggtatcagcag

V1D-39_01        aaaccagggaaagcccctaagctcctgatctatgctgcatccagtttgcaaagtggggtcccatcaaggttcagtggcagtggatctg
IGKV1D-39*01     aaaccagggaaagcccctaagctcctgatctatgctgcatccagtttgcaaagtggggtcccatcaaggttcagtggcagtggatctg

V1D-39_01        ggacagatttcactctcaccatcagcagtctgcaacctgaagattttgcaacttactactgtcaacagagttacagtacccctcc
IGKV1D-39*01     ggacagatttcactctcaccatcagcagtctgcaacctgaagattttgcaacttactactgtcaacagagttacagtacccctcc
```

IGKV1D-42

```
V1D-42_01       atggacatgagggtccccgctcagctcctggggctcctgctgctctggctcccaggtgtcagatttgacatccagatgatccagtctcca
V1D-42_02       atggacatgagggtccccgctcagctcctggggctcctgctgctctggctcccaggtgtcagatttgacatccagatgaccagtctcca
V1D-42_03       atggacatgagggtccccgctcagctcctggggctcctgctgctctggctcccaggtgtcagatttgacatccagatgaccagtctcca
V1D-42_04       atggacatgagggtccccgctcagctcctggggctcctgctgctctggctcccaggtgtcagatttgacatccagatgatccagcctcca
V1D-42_05       atggacatgagggtccccgctcagctcctggggctcctgctgctctggctcccaggtgtcagatttgacatccagatgatccagtctcca
IGKV1D-42*01    atggacatgagggtccccgctcagctcctggggctcctgctgctctggctcccaggtgtcagatttgacatccagatgatccagtctcca
IGKV1D-42*02    ------atgagggtccccgctcagctcctggggctcctgctgctctggctcccaggtgtcagatttgacatccagatgaccagtctcca
V_V1D-42*01     ------------------------------------------------------------------gacatccagatgatccagtctcca

V1D-42_01       tctttcctgtctgcatctgtaggagacagagtcagtatcatttgctgggcaagtgagggcattagcagtaatttagcctggtatctgcag
V1D-42_02       tctttcctgtctgcatctgtaggagacagagtcagtatcatttgctgggcaagtgagggcattagcagtaatttagcctggtatctgcag
V1D-42_03       tctttcctgtctgcatctgtaggagacagagtcagtatcatttgctgggcaagtgagggcattagcagtaatttagcctggtatctgcag
V1D-42_04       tctttcctgtctgcatctgtaggagacagagtcagtatcatttgctgggcaagtgagggcattagcagtaatttagcctggtatctgcag
V1D-42_05       tctttcctgtctgcatctgtaagagacagagtcagtatcatttgctgggcaagtgagggcattagcagtaatttagcctggtatctgcag
IGKV1D-42*01    tctttcctgtctgcatctgtaggagacagagtcagtatcatttgctgggcaagtgagggcattagcagtaatttagcctggtatctgcag
IGKV1D-42*02    tctttcctgtctgcatctgtaggagacagagtcagtatcatttgctgggcaagtgagggcattagcagtaatttagcctggtatctgcag
V_V1D-42*01     tctttcctgtctgcatctgtaggagacagagtcagtatcatttgctgggcaagtgagggcattagcagtaatttagcctggtatctgcag

V1D-42_01       aaaccagggaaatcccctaagctcttcctctatgatgcaaaagatttgcaccctggggtctcatcgaggttcagtggcaggggatctggga
V1D-42_02       aaaccagggaaatcccctaagctcttcctctatgatgcaaaagatttgcaccctggggtctcatcgaggttcagtggcaggggatctggga
V1D-42_03       aaaccagggaaatcccctaagctcttcctctatgatgcaaaagatttgcaccctggggtctcatcgaggttcagtggcaggggatctggga
V1D-42_04       aaaccagggaaatcccctaagctcttcctctatgatgcaaaagatttgcaccctggggtctcatcgaggttcagtggcaggggatctggga
V1D-42_05       aaaccagggaaatcccctaagctcttcctctatgatgcaaaagatttgcaccctggggtctcatcgaggttcagtggcaggggatctggga
IGKV1D-42*01    aaaccagggaaatcccctaagctcttcctctatgatgcaaaagatttgcaccctggggtctcatcgaggttcagtggcaggggatctggga
IGKV1D-42*02    aaaccagggaaatcccctaagctcttcctctatgatgcaaaagatttgcaccctggggtctcatcgaggttcagtggcaggggatctggga
V_V1D-42*01     aaaccagggaaatcccctaagctcttcctctatgatgcaaaagatttgcaccctggggtctcatcgaggttcagtggcaggggatctggga

V1D-42_01       cggatttcactctcaccatcatcagcctgaagcctgaagattttgcagcttattactgtaaacaggacttcagttaccct
V1D-42_02       cggatttcactctcaccatcatcagcctgaagcctgaagattttgcagcttattactgtaaacaggacttcagttaccct
V1D-42_03       cggatttcactctcaccatcatcagcctgaagcctgaagattttgcagcttattactgtaaacaggactttagttaccct
V1D-42_04       cggatttcactctcaccatcatcagcctgaagcctgaagattttgcagcttattactgtaaacaggacttcagttaccct
V1D-42_05       cggatttcactctcaccatcatcagcctgaagcctgaagattttgcagcttattactgtaaacaggacttcagttaccct
IGKV1D-42*01    cggatttcactctcaccatcatcagcctgaagcctgaagattttgcagcttattactgtaaacaggacttcagttaccct
IGKV1D-42*02    cggatttcactctcaccatcatcagcctgaagcctgaagattttgcagcttattactgtaaacaggacttcagttaccct
V_V1D-42*01     cggatttcactctcaccatcatcagcctgaagcctgaagattttgcagcttattactgtaaacaggacttcagttac---
```

IGKV2-24

```
V2-24_01       atgaggctccttgctcagcttctggggctgctaatgctctgggtccctggatccagtggggatattgtgatgacccagactccactctcctc
V2-24_02       atgaggctccttgctcagcttctggggctgctaatgctctgggtccctggatccagtggggatattgtgatgacccagactccactctcctc
IGKV2-24*01    atgaggctccttgctcagcttctggggctgctaatgctctgggtccctggatccagtggggatattgtgatgacccagactccactctcctc
V_V2-24*01     ------------------------------------------------------------gatattgtgatgacccagactccactctcctc

V2-24_01       acctgtcacccttggacagccggcctccatctcctgcaggtctagtcaaagcctcgtacacagtgatggaaacacctacttgagttgg
V2-24_02       acctgtcacccttggacagccggcctccatctcctgcaggtctagtcaaagcctcgtacacagtgatggaaacacctacttgagttcg
IGKV2-24*01    acctgtcacccttggacagccggcctccatctcctgcaggtctagtcaaagcctcgtacacagtgatggaaacacctacttgagttgg
V_V2-24*01     acctgtcacccttggacagccggcctccatctcctgcaggtctagtcaaagcctcgtacacagtgatggaaacacctacttgagttgg

V2-24_01       cttcagcagaggccaggccagcctccaagactcctaatttataagatttctaaccggttctctggggtcccagacagattcagtggcagtgg
V2-24_02       cttcagcagaggccaggccagcctccaagactcctaatttataagatttctaaccggttctctggggtcccagacagattcagtggcagtgg
IGKV2-24*01    cttcagcagaggccaggccagcctccaagactcctaatttataagatttctaaccggttctctggggtcccagacagattcagtggcagtgg
V_V2-24*01     cttcagcagaggccaggccagcctccaagactcctaatttataagatttctaaccggttctctggggtcccagacagattcagtggcagtgg

V2-24_01       ggcagggacagatttcacactgaaaatcagcagggtggaagctgaggatgtcggggtttattactgcatgcaagctacacaatttcct
V2-24_02       ggcagggacagatttcacactgaaaatcagcagggtggaagctgaggatgtcggggtttattactgcatgcaagctacacaatttcct
IGKV2-24*01    ggcagggacagatttcacactgaaaatcagcagggtggaagctgaggatgtcggggtttattactgcatgcaagctacacaatttcct
V_V2-24*01     ggcagggacagatttcacactgaaaatcagcagggtggaagctgaggatgtcggggtttattactgcatgcaagctacacaattt---
```

IGKV2-28

```
V2-28_01       atgaggctccctgctcagctcctggggctgctaatgctctgggtctctggatccagtggggatattgtgatgactcagtctccactctc
V2-28_02       atgaggctccctgctcagctcctggggctgctaatgctctgggtctctggatccagtggggatattgtgatgactcagtctccactctc
V2-28_03       atgaggctccctgctcagctcctggggctgctaatgctctgggtctctggatccagtggggatattgtgatgactcagtctccactctc
IGKV2-28*01    atgaggctccctgctcagctcctggggctgctaatgctctgggtctctggatccagtggggatattgtgatgactcagtctccactctc

V2-28_01       cctgcccgtcacccctggagagccggcctccatctcctgcaggtctagtcagagcctcctgcatagtaatggatacaactatttggattgg
V2-28_02       cctgcccgtcacccctggagagccggcctccatctcctgcaggtctagtcagagcctcctgcatagtaatggatacaactatttggattgg
V2-28_03       cctgcccgtcacccctggagagccggcctccatctcctgcaggtctagtcagagcctcctgcatagtaatggatacaactatttggattgg
IGKV2-28*01    cctgcccgtcacccctggagagccggcctccatctcctgcaggtctagtcagagcctcctgcatagtaatggatacaactatttggattgg

V2-28_01       tacctgcagaagccagggcagtctccacagctcctgatctatttgggttctaatcgggcctccggggtccctgacaggttcagtggca
V2-28_02       tacctgcagaagccagggaagtctccacagctcctgatctatttgggttctaatcgggcctccggggtccctgacaggttcagtggca
V2-28_03       tacctgcagaagccagggcagtctccacagctcctgatctatttgggttctaatcggtcctccggggtccctgacaggttcagtggca
IGKV2-28*01    tacctgcagaagccagggcagtctccacagctcctgatctatttgggttctaatcgggcctccggggtccctgacaggttcagtggca

V2-28_01       gtggatcaggcacagattttacactgaaaatcagcagagtggaggctgaggatgttggggtttattactgcatgcaagctctacaaactcct
V2-28_02       gtggatcaggcacagattttacactgaaaatcagcagagtggaggctgaggatgttggggtttattactgcatgcaagctctacaaactcct
V2-28_03       gtggatcaggcacagattttacactgaaaatcagcagagtggaggctgaggatgttggggtttattactgcatgcaagctctacaaactcct
IGKV2-28*01    gtggatcaggcacagattttacactgaaaatcagcagagtggaggctgaggatgttggggtttattactgcatgcaagctctacaaactcct
```

IGKV2-29

```
V2-29_01         atgaggctccctgctcagctcctggggctgctaatgctctggatccctggatccagtgcggatattgtgatgacccagactccactctctc
V2-29_02         atgaggctccctgctcagctcctggggctgctaatgctctggatccctggatccagtgcggatattgtgatgacccagactccactctctc
V2-29_03         atgaggctccctgctcagctcctggggctgctaatgctctggatccctggatccagtgtggatattgtgatgacccagactccactctctc
V2-29_04         atgaggctccctgctcagctcctggggctgctaatgctctggatccctggatccagtgcggatattgtgatgacccagactccactctctc
V2-29_05         atgaggctccctgctcagctcctggggctgctaatgctctggatccctggatccagtgtggatattgtgatgacccagactccactctctc
V2-29_06         atgaggctccctgctcagctcctggggctgctaatgctctggatccctggatccagtgcggatattgtgatgacccagactccactctctc
IGKV2-29*01      atgaggctccctgctcagctcctggggctgctaatgctctggatccctggatccagtgcggatattgtgatgacccagactccactctctc
IGKV2-29*02      atgaggctccctgctcagctcctggggctgctaatgctctggatccctggatccagtgcggatattgtgatgacccagactccactctctc
IGKV2-29*03      ------------------------------------------------------------gatattgtgatgacccagactccactctctc
V_V2-29*02       ------------------------------------------------------------gatattgtgatgacccagactccactctctc
V_V2-29*01(P)    ------------------------------------------------------------gatattgtgatgacccagactccactctctc

V2-29_01         tgtccgtcacccctggacagccggcctccatctcctgcaagtctagtcagagcctcctgcatagtgatggaaagacctatttgtattgg
V2-29_02         tgtccgtcacccctggacagccggcctccatctcctgcaagtctagtcagagcctcctgcatagtgatggaaagacctatttgtattgg
V2-29_03         tgtccgtcacccctggacagccggcctccatctcctgcaagtctagtcagagcctcctgcatagtgatggaaagacctatttgtattgg
V2-29_04         tgtccgtcacccctggacagccggcctccatctcctgcaagtctagtcagagcctcctgcatagtgatggaaagacctatttgtattgg
V2-29_05         tgtccgtcacccctggacagccggcctccatctcctgcaagtctagtcagagcctcctgcatagtgatggaaagacctatttgtattgg
V2-29_06         tgtccgtcacccctggacagccggcctccatctcctgcaagtctagtcagagcctcctacatagtgatggaaagacctatttgtattgg
IGKV2-29*01      tgtccgtcacccctggacagccggcctccatctcctgcaagtctagtcagagcctcctgcatagtgatggaaagacctatttgtattgg
IGKV2-29*02      tgtccgtcacccctggacagccggcctccatctcctgcaagtctagtcagagcctcctgcatagtgatggaaagacctatttgtattgg
IGKV2-29*03      tgtccgtcacccctggacagccggcctccatctcctgcaagtctagtcagagcctcctgcatagtgatggaaagacctatttgtattgg
V_V2-29*02       tgtccgtcacccctggacagccggcctccatctcctgcaagtctagtcagagcctcctgcatagtgatggaaagacctatttgtattgg
V_V2-29*01(P)    tgtccgtcacccctggacagccggcctccatctcctgcaagtctagtcagagcctcctgcatagtgatggaaagacctatttgtattgg

V2-29_01         tacctgcagaagccaggccagtctccacagctcctgatctatgaagtttccagccggttctctggagtgccagataggttcagtggcagcg
V2-29_02         tacctgcagaagccaggccagtctccacagctcctaatctatgaagtttccagccggttctctggagtgccagataggttcagtggcagcg
V2-29_03         tacctgcagaagccaggccagtctccacagctcctgatctatgaagtttccagccggttctctggagtgccagataggttcagtggcagcg
V2-29_04         tacctgcagaagccaggccagtctccacagctcctgatctatgaagtttccagccggttctctggagtgccagataggttcagtggcagcg
V2-29_05         tacctgcagaagccaggccagtctccacagctcctaatctatgaagtttccagccggttctctggagtgccagataggttcagtggcagcg
V2-29_06         tacctgcagaagccaggccagtctccacagctcctgatctatgaagtttccagccggttctctggagtgccagataggttcagtggcagcg
IGKV2-29*01      tacctgcagaagccaggccagtctccacagctcctgatctatgaagtttccagccggttctctggagtgccagataggttcagtggcagcg
IGKV2-29*02      tacctgcagaagccaggccagtctccacagctcctaatctatgaagtttccagccggttctctggagtgccagataggttcagtggcagcg
IGKV2-29*03      tacctgcagaagccaggccagtctccacagctcctgatctatgaagtttccagccggttctctggagtgccagataggttcagtggcagcg
V_V2-29*02       tacctgcagaagccaggccagtctccacagctcctaatctatgaagtttccagccggttctctggagtgccagataggttcagtggcagcg
V_V2-29*01(P)    tacctgcagaagccaggccagtctccacagctcctgatctatgaagtttccagccggttctctggagtgccagataggttcagtggcagcg
```

V2-29_01	ggtcagggacagatttcacactgaaaatcagccgggtggaggctgaggatgttggggtttattactgaaatgcaaggtatacaccttcctcccacagtggt
V2-29_02	ggtcagggacagatttcacactgaaaatcagccgggtggaggctgaggatgttggggtttattactgcatgcaaggtatacaccttcctcccacagtggt
V2-29_03	ggtcagggacagatttcacactgaaaatcagccgggtggaggctgaggatgttggggtttattactgcatgcaaggtatacaccttcctcccacagtggt
V2-29_04	ggtcagggacagatttcacactgaaaatcagccgggtggaggctgaggatgttggggtttattactgcatgcaaggtatacaccttcctcccacagtggt
V2-29_05	ggtcagggacagatttcacactgaaaatcagccgggtggaggctgaggatgttggggtttattactgcatgcaaggtatacaccttcctcccacagtggt
V2-29_06	ggtcagggacagatttcacactgaaaatcagccgggtggaggctgaggatgttggggtttattactgaaatgcaaggtatacaccttcctcccacagtggt
IGKV2-29*01	ggtcagggacagatttcacactgaaaatcagccgggtggaggctgaggatgttggggtttattactgaaatgcaaggtatacaccttcctcc---------
IGKV2-29*02	ggtcagggacagatttcacactgaaaatcagccgggtggaggctgaggatgttggggtttattactgcatgcaaggtatacaccttcctcc---------
IGKV2-29*03	ggtcagggacagatttcacactgaaaatcagccgggtggaggctgaggatgttggggtttattactgcatgcaaggtatacaccttcctcc---------
V_V2-29*02	ggtcagggacagatttcacactgaaaatcagccgggtggaggctgaggatgttggggtttattactgcatgcaaggtatacacctt--------------
V_V2-29*01(P)	ggtcagggacagatttcacactgaaaatcagccgggtggaggctgaggatgttggggtttattactgaaatgcaaggtatacacctt--------------

IGKV2-30

```
V2-30_01      atgaggctccctgctcagctcctggggctgctaatgctctgggtcccaggatccagtggggatgttgtgatgactcagtctccactctccc
V2-30_02      atgaggctccctgctcagctcctggggctgctaatgctctgggtcccaggatccagtggggatgttgtgatgactcagtctccactctccc
V2-30_03      atgaggctccctgctcagctcctggggctgctaatgctctgggtcccaggatccagtggggatgttgtgatgactcagtctccactctccc
IGKV2-30*01   atgaggctccctgctcagctcctggggctgctaatgctctgggtcccaggatccagtggggatgttgtgatgactcagtctccactctccc
IGKV2-30*02   ------------------------------------------------------------gatgttgtgatgactcagtctccactctccc
V_V2-30*01    ------------------------------------------------------------gatgttgtgatgactcagtctccactctccc

V2-30_01      tgcccgtcacccttggacagccggcctccatctcctgcaggtctagtcaaagcctcgtatacagtgatggaaacacctacttgaattgg
V2-30_02      tgcccgtcacccttggacagccggcctccatctcctgcaggtctagtcaaagcctcgtacacagtgatggaaacacctacttgaattgg
V2-30_03      tgcccgtcacccttggacagccggcctccatctcctgcaggtctagtcaaagcctcgtatacagtgatggaaacacctacttgaattgg
IGKV2-30*01   tgcccgtcacccttggacagccggcctccatctcctgcaggtctagtcaaagcctcgtatacagtgatggaaacacctacttgaattgg
IGKV2-30*02   tgcccgtcacccttggacagccggcctccatctcctgcaggtctagtcaaagcctcgtacacagtgatggaaacacctacttgaattgg
V_V2-30*01    tgcccgtcacccttggacagccggcctccatctcctgcaggtctagtcaaagcctcgtatacagtgatggaaacacctacttgaattgg

V2-30_01      tttcagcagaggccaggccaatctccaaggcgcctaatttataaggtttctaaccgggactctggggtcccagacagattcagcggcagt
V2-30_02      tttcagcagaggccaggccaatctccaaggcgcctaatttataaggtttctaaccgggactctggggtcccagacagattcagcggcagt
V2-30_03      tttcagcagaggccaggccaatctccaaggcgcctaatttataaggtttctaaccgggactctggggtcccagacagattcagcggcagt
IGKV2-30*01   tttcagcagaggccaggccaatctccaaggcgcctaatttataaggtttctaaccgggactctggggtcccagacagattcagcggcagt
IGKV2-30*02   tttcagcagaggccaggccaatctccaaggcgcctaatttataaggtttctaaccgggactctggggtcccagacagattcagcggcagt
V_V2-30*01    tttcagcagaggccaggccaatctccaaggcgcctaatttataaggtttctaaccgggactctggggtcccagacagattcagcggcagt

V2-30_01      gggtcaggcactgatttcacactgaaaatcagcagggtggaggctgaggatgttggggtttattactgcatgcaaggtacacactggcct
V2-30_02      gggtcaggcactgatttcacactgaaaatcagcagggtggaggctgaggatgttggggtttattactgcatgcaaggtacacactggcct
V2-30_03      gggtcaggcactgatttcacactgaaaatcatcagggtggaggctgaggatgttggggtttattactgcatgcaaggtacacactggcct
IGKV2-30*01   gggtcaggcactgatttcacactgaaaatcagcagggtggaggctgaggatgttggggtttattactgcatgcaaggtacacactggcct
IGKV2-30*02   gggtcaggcactgatttcacactgaaaatcagcagggtggaggctgaggatgttggggtttattactgcatgcaaggtacacactggcct
V_V2-30*01    gggtcaggcactgatttcacactgaaaatcagcagggtggaggctgaggatgttggggtttattactgcatgcaaggtacacactgg---
```

IGKV2-40

```
V2-40_01        atgaggctccctgctcagctcctggggctgctaatgctctgggtccctggatccagtgaggatattgtgatgacccagactccactctcc
IGKV2-40*01     atgaggctccctgctcagctcctggggctgctaatgctctgggtccctggatccagtgaggatattgtgatgacccagactccactctcc
IGKV2-40*02     ------------------------------------------------------------------------------------------

V2-40_01        ctgcccgtcacccctggagagccggcctccatctcctgcaggtctagtcagagcctcttggatagtgatgatggaaacacctatttggac
IGKV2-40*01     ctgcccgtcacccctggagagccggcctccatctcctgcaggtctagtcagagcctcttggatagtgatgatggaaacacctatttggac
IGKV2-40*02     ------------------------gcctccatctcctgcaggtctagtcagagcctcttggatagtgatgatggaaacacctatttggat

V2-40_01        tggtacctgcagaagccagggcagtctccacagctcctgatctatacgctttcctatcgggcctctggagtcccagacaggttcagtggc
IGKV2-40*01     tggtacctgcagaagccagggcagtctccacagctcctgatctatacgctttcctatcgggcctctggagtcccagacaggttcagtggc
IGKV2-40*02     tgttacctgcagaagccagggcagtctccacagctcctgatctatacgctttcctatcgggcctctggagtcccagacaggttcagtgac

V2-40_01        agtgggtcaggcactgatttcacactgaaaatcagcagggtggaggctgaggatgttggagtttattactgcatgcaacgtatagagtttccttc
IGKV2-40*01     agtgggtcaggcactgatttcacactgaaaatcagcagggtggaggctgaggatgttggagtttattactgcatgcaacgtatagagtttccttc
IGKV2-40*02     agtgggtcaggcactgatttcacactgaaaatcagcagggtggaggctgaggatgttggagtttattactgcatgcaacgtatagagtttccttc
```

IGKV2D-24

```
V2D-24_01       atgaggctccttgctcagcttctggggctgctaatgctctgggtccctggatccagtggggatattgtgatgacccagactccactctcc
V2D-24_02       atgaggctccttgctcagcttctggggctgctaatgctctgggtccctggatccagtggggatattgtgatgacccagactccactctcc
IGKV2D-24*01    atgaggctccttgctcagcttctggggctgctaatgctctgggtccctggatccagtggggatattgtgatgacccagactccactctcc
V_V2D-24*01     ------------------------------------------------------------gatattgtgatgacccagactccactctcc

V2D-24_01       tcgcctgtcacccttggacagccggcctccatctccttcaggtctagtcaaagcctcgtacacagtgatggaaacacctacttgagttgg
V2D-24_02       tctcctgtcacccttggacagccggcctccatctccttcaggtctagtcaaagcctcgtacacagtgatggaaacacctacttgagttgg
IGKV2D-24*01    tcgcctgtcacccttggacagccggcctccatctccttcaggtctagtcaaagcctcgtacacagtgatggaaacacctacttgagttgg
V_V2D-24*01     tcgcctgtcacccttggacagccggcctccatctccttcaggtctagtcaaagcctcgtacacagtgatggaaacacctacttgagttgg

V2D-24_01       cttcagcagaggccaggccagcctccaagactcctaatttataaggtttctaaccggttctctggggtcccagacagattcagtggcagtgggg
V2D-24_02       cttcagcagaggccaggccagcctccaagactcctaatttataaggtttctaaccggttctctggggtcccagacagattcagtggcagtgggg
IGKV2D-24*01    cttcagcagaggccaggccagcctccaagactcctaatttataaggtttctaaccggttctctggggtcccagacagattcagtggcagtgggg
V_V2D-24*01     cttcagcagaggccaggccagcctccaagactcctaatttataaggtttctaaccggttctctggggtcccagacagattcagtggcagtgggg

V2D-24_01       cagggacagatttcacactgaaaatcagcagggtggaagctgaggatgtcggggtttattactgcacgcaagctacacaatttcct
V2D-24_02       cagggacagatttcacactgaaaatcagcagggtggaagctgaggatgtcggggtttattactgcacgcaagctacacaatttcct
IGKV2D-24*01    cagggacagatttcacactgaaaatcagcagggtggaagctgaggatgtcggggtttattactgcacgcaagctacacaatttcct
V_V2D-24*01     cagggacagatttcacactgaaaatcagcagggtggaagctgaggatgtcggggtttattactgcacgcaagctacacaattt---
```

IGKV2D-26

```
V2D-26_01       atgaggctccctgctcagctcttggggctgctaatgctctgggtccctggatccagtgcagagattgtgatgacccagactccactctcc
V2D-26_02       atgaggctccctgctcagctcttggggctgctaatgctctgggtccctggatccagtgcagagattgtgatgacccagactccactctcc
V2D-26_03       atgaggctccctgctcagctcttggggctgctaatgctctgggtccctggatccagtgcagagattgtgatgacccagactccactctcc
V2D-26_04       atgaggctccctgctcagctcttggggctgctaatgctctgggtccctggatccagtgcagagattgtgatgacccagactccactctcc
V2D-26_05       atgaggctccctgctcagctcttggggctgctaatgctctgggtccctggatccagtgcagagattgtgatgacccagactccactctcc
IGKV2D-26*01    atgaggctccctgctcagctcttggggctgctaatgctctgggtccctggatccagtgcagagattgtgatgacccagactccactctcc
IGKV2D-26*03    atgaggctccctgctcagctcttggggctgctaatgctctgggtccctggatccagtgcagagattgtgatgacccagactccactctcc
IGKV2D-26*02    ------------------------------------------------------------gagattgtgatgacccagactccactctcc

V2D-26_01       ttgtctatcacccctggagagcaggcctccatgtcctgcaggtctagtcagagcctcctgcatagtgatggatacacctatttgtattgg
V2D-26_02       ttgtctatcacccctggagagcaggcctccatctcctgcaggtctagtcagagcctcctgcatagtgatggatacacctatttgtattgg
V2D-26_03       ttgtctatcacccctggagagcaggcctccatctcctgcaggtctagtcagagcctcctgcatagtgatggatacacctatttgtattgg
V2D-26_04       ttgtctatcacccctggagagcaggcctccatgtcctgcaggtctagtcagagcctcctgcatagtgatggatacacctatttgtattgg
V2D-26_05       ttgtctatcacccctggagagcaggcctccatgtcctgcaggtctagtcagagcctcctgcatagtgatggatacacctatttgtattgg
IGKV2D-26*01    ttgtctatcacccctggagagcaggcctccatctcctgcaggtctagtcagagcctcctgcatagtgatggatacacctatttgtattgg
IGKV2D-26*03    ttgtctatcacccctggagagcaggcctccatgtcctgcaggtctagtcagagcctcctgcatagtgatggatacacctatttgtattgg
IGKV2D-26*02    ttgtctatcacccctggagagcaggcctccatgtcctgcaggtctagtcagagcctcctgcatagtgatggatacacctatttgtattgg

V2D-26_01       tttctgcagaaagccaggccagtctccacgctcctgatctatgaagtttccaaccggttctctggagtgccagataggttcagtggcagcg
V2D-26_02       tttctgcagaaagccaggccagtctccacgctcctgatctatgaagtttccaaccggttctctggagtgccagataggttcagtggcagcg
V2D-26_03       tttctgcagaaagccaggccagtctccacgctcctgatctatgaagtttccaaccggttctctggagtgccagataggttcagtggcagtg
V2D-26_04       tttctgcagaaagccaggccagtctccacactcctgatctatgaagtttccaaccggttctctggagtgccagataggttcagtggcagcg
V2D-26_05       tttctgcagaaagccaggccagtctccacgctcctgatctatgaagtttccaaccggttctctggagtgccagataggttcagtggcagcg
IGKV2D-26*01    tttctgcagaaagccaggccagtctccacactcctgatctatgaagtttccaaccggttctctggagtgccagataggttcagtggcagcg
IGKV2D-26*03    tttctgcagaaagccaggccagtctccacgctcctgatctatgaagtttccaaccggttctctggagtgccagataggttcagtggcagcg
IGKV2D-26*02    tttctgcagaaagccaggccagtctccacgctcctgatctgtgaagtttccaaccggttctctggagtgccagataggttcagtggcagcg

V2D-26_01       ggtcagggacagatttcacactgaaaatcagccgggtggaggctgaggattttggagtttattactgcatgcaagatgcacaagatcct
V2D-26_02       ggtcagggacagatttcacactgaaaatcagccgggtggaggctgaggattttggagtttattactgcatgcaagatgcacaagatcct
V2D-26_03       ggtcagggacagatttcacactgaaaatcagccgggtggaggctgaggattttggagtttattactgcatgcaagatgcacaagatcct
V2D-26_04       ggtcagggacagatttcacactgaaaatcagccgggtggaggctgaggattttggagtttattactgcatgcaagatgcacaagatcct
V2D-26_05       ggtcagggacagatttcacactgaaaatcagccgggtggaggctgaggattttggagttaattactgcatgcaagatgcacaagatcct
IGKV2D-26*01    ggtcagggacagatttcacactgaaaatcagccgggtggaggctgaggattttggagtttattactgcatgcaagatgcacaagatcct
IGKV2D-26*03    ggtcagggacagatttcacactgaaaatcagccgggtggaggctgaggattttggagtttattactgcatgcaagatgcacaagatcct
IGKV2D-26*02    ggtcagggacagatttcacactgaaaatcagccgggtggaggctgaggattttggagtttattactgcatgcaagatgcacaagatcct
```

IGKV2D-28

```
V2D-28_01      atgaggctccctgctcagctcctggggctgctaatgctctgggtctctggatccagtggggatattgtgatgactcagtctccactctccc
V2D-28_02      atgaggctccctgctcagctcctggggctgctaatgctctgggtctctggatccagtggggatattgtgatgactcagtctccactctccc
V2D-28_03      atgaggctccctgctcagctcctggggctgctaatgctctgggtctctggatccagtggggatattgtgatgactcagcctccactctccc
IGKV2D-28*01   atgaggctccctgctcagctcctggggctgctaatgctctgggtctctggatccagtggggatattgtgatgactcagtctccactctccc
V_V2D-28*01    ------------------------------------------------------------gatattgtgatgactcagtctccactctccc

V2D-28_01      tgcccgtcacccctggagagccggcctccatctcctgcaggtctagtcagagcctcctgcatagtaatggatacaactatttggattgg
V2D-28_02      tgcccgtcacccctggagagccggcctccatctcctgcaggtctagtcagagcctcctgcatagtaatggatacaactatttggattgg
V2D-28_03      tgcccgtcacccctggagagccggcctccatctcctgcaggtctagtcagagcctcctgcatagtaatggatacaactatttggattgg
IGKV2D-28*01   tgcccgtcacccctggagagccggcctccatctcctgcaggtctagtcagagcctcctgcatagtaatggatacaactatttggattgg
V_V2D-28*01    tgcccgtcacccctggagagccggcctccatctcctgcaggtctagtcagagcctcctgcatagtaatggatacaactatttggattgg

V2D-28_01      tacctgcagaagccagggcagtctccacagctcctgatctatttgggttctaatcgggcctccggggtccctgacaggttcagtggcag
V2D-28_02      tacctgcagaagccaggacagtctccacagctcctgatctatttgggttctaatcgggcctccggggtccctgacaggttcagtggcag
V2D-28_03      tacctgcagaagccagggcagtctccacagctcctgatctatttgggttctaatcgggcctccggggtccctgacaggttcagtggcag
IGKV2D-28*01   tacctgcagaagccagggcagtctccacagctcctgatctatttgggttctaatcgggcctccggggtccctgacaggttcagtggcag
V_V2D-28*01    tacctgcagaagccagggcagtctccacagctcctgatctatttgggttctaatcgggcctccggggtccctgacaggttcagtggcag

V2D-28_01      tggatcaggcacagattttacactgaaaatcagcagagtggaggctgaggatgttggggtttattactgcatgcaagctctacaaactcct
V2D-28_02      tggatcaggcacagattttacactgaaaatcagcagagtggaggctgaggatgttggggtttattactgcatgcaagctctacaaactcct
V2D-28_03      tggatcaggcacagattttacactgaaaatcagcagagtggaggctgaggatgttggggtttattactgcatgcaagctctacaaactcct
IGKV2D-28*01   tggatcaggcacagattttacactgaaaatcagcagagtggaggctgaggatgttggggtttattactgcatgcaagctctacaaactcct
V_V2D-28*01    tggatcaggcacagattttacactgaaaatcagcagagtggaggctgaggatgttggggtttattactgcatgcaagctctacaaact---
```

IGKV2D-29

```
V2D-29_01        atgaggctccctgctcagctcctggggctgctaatgctctggatacctggatccagtgcagatattgtgatgacccagactccactctct
V2D-29_03        atgaggctccctgctcagctcctggggctgctaatgctctggatacctggatccagtgcagatattgtgatgacccagactccactctct
IGKV2D-29*01     atgaggctccctgctcagctcctggggctgctaatgctctggatacctggatccagtgcagatattgtgatgacccagactccactctct
IGKV2D-29*02     atgaggctccctgctcagctcctggggctgctaatgctctggatacctggatccagtgcagatattgtgatgacccagactccactctct
V_V2D-29*02      -------------------------------------------------------------gatattgtgatgacccagactccactctct
V_V2D-29*01      -------------------------------------------------------------gatattgtgatgacccagactccactctct

V2D-29_01        ctgtccgtcacccctggacagccggcctccatctcctgcaagtctagtcagagcctcctgcatagtgatggaaagacctatttgtattgg
V2D-29_03        ctgtccgtcacccctggacagccggcctccatctcctgcaagtctagtcagagcctcctgcatagtgatggaaagacctatttgtattgg
IGKV2D-29*01     ctgtccgtcacccctggacagccggcctccatctcctgcaagtctagtcagagcctcctgcatagtgatggaaagacctatttgtattgg
IGKV2D-29*02     ctgtccgtcacccctggacagccggcctccatctcctgcaagtctagtcagagcctcctgcatagtgatggaaagacctatttgtattgg
V_V2D-29*02      ctgtccgtcacccctggacagccggcctccatctcctgcaagtctagtcagagcctcctgcatagtgatggaaagacctatttgtattgg
V_V2D-29*01      ctgtccgtcacccctggacagccggcctccatctcctgcaagtctagtcagagcctcctgcatagtgatggaaagacctatttgtattgg

V2D-29_01        tacctgcagaagccaggccagcctccacagctcctgatctatgaagtttccaaccggttctctggagtgccagataggttcagtggcagc
V2D-29_03        tacctgcagaagccaggccagcctccacagctcctgatctatgaagtttccaaccggttctctggagtgccagataggttcagtggcagc
IGKV2D-29*01     tacctgcagaagccaggccagcctccacagctcctgatctatgaagtttccaaccggttctctggagtgccagataggttcagtggcagc
IGKV2D-29*02     tacctgcagaagccaggccagtctccacagctcctgatctatgaagtttccaaccggttctctggagtgccagataggttcagtggcagc
V_V2D-29*02      tacctgcagaagccaggccagtctccacagctcctgatctatgaagtttccaaccggttctctggagtgccagataggttcagtggcagc
V_V2D-29*01      tacctgcagaagccaggccagcctccacagctcctgatctatgaagtttccaaccggttctctggagtgccagataggttcagtggcagc

V2D-29_01        gggtcagggacagatttcacactgaaaatcagccgggtggaggctgaggatgttggggtttattactgcatgcaaagtatacagcttcct
V2D-29_03        ggggcagggacagatttcacactgaaaatcagccgggtggaggctgaggatgttggggtttattactgcatgcaaagtatacagcttcct
IGKV2D-29*01     gggtcagggacagatttcacactgaaaatcagccgggtggaggctgaggatgttggggtttattactgcatgcaaagtatacagcttcct
IGKV2D-29*02     gggtcagggacagatttcacactgaaaatcagccgggtggaggctgaggatgttggggtttattactgcatgcaaagtatacagcttcct
V_V2D-29*02      gggtcagggacagatttcacactgaaaatcagccgggtggaggctgaggatgttggggtttattactgcatgcaaagtatacagctt---
V_V2D-29*01      gggtcagggacagatttcacactgaaaatcagccgggtggaggctgaggatgttggggtttattactgcatgcaaagtatacagctt---
```

IGKV2D-30

```
V2D-30_01      atgaggctccctgctcagctcctggggctgctaatgctctgggtcccaggatccagtggggatgttgtgatgactcagtctccactctcc
V2D-30_02      atgaggcaccctgctcagctcctggggctgctaatgctctgggtcccaggatccagtggggatgttgtgatgactcagtctccactctcc
V2D-30_03      atgaggctccctgctcagctcctggggctgctaatgctctgggtcccaggatccagtggggatgttgtgatgactcagtctccactctcc
V2D-30_04      atgaggctccctgctcagctcctggggctgctaatgctctgggtcccaggatccagtggggatgttgtgatgactcagtctccactctcc
V2D-30_05      atgaggctccctgctcagctcctggggctgctaatgctctgggtcccaggatccagtggggatgttgtgatgactcagtctccactctcc
V2D-30_06      atgaggctccctgctcagctcctggggctgctaatgctctgggtcccaggatccagtggggatgttgtgatgactcagtctccactctcc
IGKV2D-30*01   atgaggctccctgctcagctcctggggctgctaatgctctgggtcccaggatccagtggggatgttgtgatgactcagtctccactctcc
V_V2D-30*01    ------------------------------------------------------------gatgttgtgatgactcagtctccactctcc

V2D-30_01      ctgcccgtcacccttggacagccggcctccatctcctgcaggtctagtcaaagcctcgtatacagtgatggaaacacctacttgaattgg
V2D-30_02      ctgcccgtcacccttggacagccggcctccatctcctgcaggtctagtcaaagcctcgtatacagtgatggaaacacctacttgaattgg
V2D-30_03      ctgcccgtcacccttggacagctggcctccatctcctgcaggtctagtcaaagcctcgtatacagtgatggaaacacctacttgaattgg
V2D-30_04      ctgcccgtcacccttggacagccggcctccatctcctgcaggtctagtcaaagcctcgtatacagtgatggaaacacctacttgaattgg
V2D-30_05      ctgcccgtcacccttggacagccggcctccatctcctgcaggtctagtcaaagcctcgtatacagtgatggaaacacctacttgaattgg
V2D-30_06      ctgcccgtcacccttggacagccggcctccatctcctgcaggtctagtcaaagcctcgtatacagtgatggaaacacctacttgaattgg
IGKV2D-30*01   ctgcccgtcacccttggacagccggcctccatctcctgcaggtctagtcaaagcctcgtatacagtgatggaaacacctacttgaattgg
V_V2D-30*01    ctgcccgtcacccttggacagccggcctccatctcctgcaggtctagtcaaagcctcgtatacagtgatggaaacacctacttgaattgg

V2D-30_01      tttcagcagaggccaggccaatctccaaggcgcctaatttataaggtttctaactgggactctggggtcccagacagattcagcggcagt
V2D-30_02      tttcagcagaggccaggccaatctccaaggcgcctaatttataaggtttctaactgggactctggggtcccagacagattcagcggcagt
V2D-30_03      tttcagcagaggccaggccaatctccaaggcgcctaatttataaggtttctaactgggactctggggtcccagacagattcagcggcagt
V2D-30_04      tttcagcagaggccaggccaatctccaaggcacctaatttataaggtttctaactgggactctggggtcccagacagattcagcggcagt
V2D-30_05      tttcagcagagtccaggccaatctccaaggcgcctaatttataaggtttctaactgggactctggggtcccagacagattcagcggcagt
V2D-30_06      tttcagcagaggccaggccaatctccaaggcgcctaatttataaggtttctaactgggactctggggtcccagacagattcagcggcagt
IGKV2D-30*01   tttcagcagaggccaggccaatctccaaggcgcctaatttataaggtttctaactgggactctggggtcccagacagattcagcggcagt
V_V2D-30*01    tttcagcagaggccaggccaatctccaaggcgcctaatttataaggtttctaactgggactctggggtcccagacagattcagcggcagt

V2D-30_01      gggtcaggcactgatttcacactgaaaatcagcagggtggaggctgaggatgttggggtttattactgcatgcaaggtacacactggcct
V2D-30_02      gggtcaggcactgatttcacactgaaaatcagcagggtggaggctgaggatgttggggtttattactgcatgcaaggtacacactggcct
V2D-30_03      gggtcaggcactgatttcacactgaaaatcagcagggtggaggctgaggatgttggggtttattactgcatgcaaggtacacactggcct
V2D-30_04      gggtcaggcactgatttcacactgaaaatcagcagggtggaggctgaggatgttggggtttattactgcatgcaaggtacacactggcct
V2D-30_05      gggtcaggcactgatttcacactgaaaatcagcagggtggaggctgaggatgttggggtttattactgcatgcaaggtacacactggcct
V2D-30_06      gggtcaggcaccgatttcacactgaaaatcagcagggtggaggctgaggatgttggggtttattactgcatgcaaggtacacactggcct
IGKV2D-30*01   gggtcaggcactgatttcacactgaaaatcagcagggtggaggctgaggatgttggggtttattactgcatgcaaggtacacactggcct
V_V2D-30*01    gggtcaggcactgatttcacactgaaaatcagcagggtggaggctgaggatgttggggtttattactgcatgcaaggtacacactgg---
```

IGKV2D-40

```
V2D-40_01      atgaggctccctgctcagctcctggggctgctaatgctctgggtccctggatccagtgaggatattgtgatgacccagactccactctccc
V2D-40_02      ---------------------------------atgctctgggtccctggatccagtgaggttattgtgatgacccagactccactctccc
V2D-40_03      ---------------------------------atgctctgggtccctggatccagtgaggttattgtgatgacccagactccactctccc
V2D-40_04      ---------------------------------atgctctgggtccctggatccagtgaggttattgtgatgacccagactccactctccc
IGKV2D-40*01   atgaggctccctgctcagctcctggggctgctaatgctctgggtccctggatccagtgaggatattgtgatgacccagactccactctccc
V_V2D-40*01    -------------------------------------------------------------gatattgtgatgacccagactccactctccc

V2D-40_01      tgcccgtcacccctggagagccggcctccatctcctgcaggtctagtcagagcctcttggatagtgatgatggaaacacctatttggac
V2D-40_02      tgcccgtcacccctggagagccggcctccatctcctgcaggtctagtcagagcctcttggatagtgatgatggaaacacctatttggat
V2D-40_03      tgcccgtcacccctggagagccggcctccatctcctgcaggtctagtcagagcctcttggatagtgatgatggaaacacctatttggat
V2D-40_04      tgcccgtcacccctggagagccggcctccatctcctgcaggtctagtcagagcctcttggatagtgatgatggaaacacctatttggac
IGKV2D-40*01   tgcccgtcacccctggagagccggcctccatctcctgcaggtctagtcagagcctcttggatagtgatgatggaaacacctatttggac
V_V2D-40*01    tgcccgtcacccctggagagccggcctccatctcctgcaggtctagtcagagcctcttggatagtgatgatggaaacacctatttggac

V2D-40_01      tggtacctgcagaagccagggcagtctccacagctcctgatctatacgctttcctatcgggcctctggagtcccagacaggttcagtgg
V2D-40_02      tgttacctgcagaagccagggcagtctccacagctcctcatctatacgctttcctatcgggcctctggagtcccagacaggttcagtgg
V2D-40_03      tgttacctgcagaagccagggcagtctccacagctcctgatctatacgctttcctatcgggcctctggagtcccagacaggttcagtgg
V2D-40_04      tggtacctgcagaagccagggcagtctccacagctcctgatctatacgctttcctatcgggcctctggagtcccagacaggttcagtgg
IGKV2D-40*01   tggtacctgcagaagccagggcagtctccacagctcctgatctatacgctttcctatcgggcctctggagtcccagacaggttcagtgg
V_V2D-40*01    tggtacctgcagaagccagggcagtctccacagctcctgatctatacgctttcctatcgggcctctggagtcccagacaggttcagtgg

V2D-40_01      cagtgggtcaggcactgatttcacactgaaaatcagcagggtggaggctgaggatgttggagtttattactgcatgcaacgtatagagtttccttc
V2D-40_02      cagtgggtcaggcactgatttcacactgaaaatcagcagggtggaggctgaggatgttggagtttattactgcatgcaacgtatagagtttccttc
V2D-40_03      cagtgggtcaggcactgatttcacactgaaaatcagcagggtggaggctgaggatgttggagtttattactgcatgcaacgtatagagtttccttc
V2D-40_04      cagtgggtcaggcactgatttcacactgaaaatcagcagggtggaggctgaggatgttggagtttattactgcatgcaacgtatagagtttccttc
IGKV2D-40*01   cagtgggtcaggcactgatttcacactgaaaatcagcagggtggaggctgaggatgttggagtttattactgcatgcaacgtatagagtttccttc
V_V2D-40*01    cagtgggtcaggcactgatttcacactgaaaatcagcagggtggaggctgaggatgttggagtttattactgcatgcaacgtatagagttt-----
```

IGKV3-7

```
V3-7_01      atggaagccccagctcagcttctcttcctcctgctactctggctcccagataccaccagagaaattgtaatgacacagtctccacccaccc
V3-7_02      atggaagccccagcgcagcttctcttcctcctgctactctggctcccagataccaccagagaaattgtaatgacacagtctccacccaccc
V3-7_03      atggaagccccagcgcagcttctcttcctcctgctactctggctcccagataccaccagagaaattgtaatgacacagtctccacccaccc
V3-7_04      atggaagccccagcgcagcttctcttcctcctgctactctggctcccagataccaccagagaaattgtaatgacacagtctccacccaccc
V3-7_05      atggaagccccagcgcagcttctcttcctcctgctactctggctcccagataccaccagagaaattgtaatgacacagtctccacccaccc
V3-7_06      atggaagccccagctcagcttctcttcctcctgctactctggctcccagataccaccagagaaattgtaatgacacagtctccacccaccc
V3-7_07      atggaagccccagcgcagcttctcttcctcctgctactctggctcccagataccaccagagaaattgtaatgacacagtctccacccaccc
V3-7_08      atggaagccccagctcagcttctcttcctcctgctactctggctcccagataccaccagagaaattgtaatgacacagtctccacccaccc
V3-7_09      atggaagccccagcgcagcttctcttcctcctgctactctggctcccagataccaccagagaaattgtaatgacacagtctccacccaccc
V3-7_10      atggaagccccagcgcagcttctcttcctcctgctactctggctcccagataccaccagagaaattgtaatggcacagtctccacccaccc
IGKV3-7*01   atggaagccccagctcagcttctcttcctcctgctactctggctcccagataccaccagagaaattgtaatgacacagtctccacccaccc
IGKV3-7*02   atggaagccccagcgcagcttctcttcctcctgctactctggctcccagataccaccagagaaattgtaatgacacagtctccacccaccc
IGKV3-7*03   atggaagccccagctcagcttctcttcctcctgctactctggctcccagataccaccagagaaattgtaatgacacagtctccacccaccc
IGKV3-7*04   ------------------------------------------------------------gaaattgtaatgacacagtctccacccaccc
V_V3-7*02    ------------------------------------------------------------gaaattgtaatgacacagtctccacccaccc
V_V3-7*01    ------------------------------------------------------------gaaattgtaatgacacagtctccacccaccc

V3-7_01      tgtctttgtctccaggggaaagagtcaccctctcctgcagggccagtcagagtgttagcagcagctacttaacctggtatcagcagaaa
V3-7_02      tgtctttgtctccaggggaaagagtcaccctctcctgcagggccagtcagagtgttagcagcagctacttaacctggtatcagcagaaa
V3-7_03      tgtctttgtctccaggggaaagagtcaccctctcctgcagggccagtcagagtgttagcagcagctacttaacctggtatcagcagaaa
V3-7_04      tgtctttgtctccaggggaaagagccaccctctcctgcagggccagtcagagtgttagcagcagctacttaacctggtatcagcagaaa
V3-7_05      tgtctttgtctccaggggaaagagccaccctctcctgcagggccagtcagagtgttagcagcagctacttaacctggtaccagcagaaa
V3-7_06      tgtctttgtctccaggggaaagagtcaccctctcctgcagggccagtcagagtgttagcagcagctacttaacctggtatcagcagaaa
V3-7_07      tgtctttgtctccaggggaaagagtcaccctctcctgcagggccagtcagagtgttagcagcagctacttaacctggtatcagcagaaa
V3-7_08      tgtctttgtctccaggggaaagagtcaccctctcctgcagggccagtcagagtgttagcagcagctacttaacctggtaccagcagaaa
V3-7_09      tgtctttgtctccaggggaaagagtcaccctctcctgcagggccagtcagagtgttagcagcagctacttaacctggtatcagcagaaa
V3-7_10      tgtctttgtctccaggggaaagagccaccctctcctgcagggccagtcagagtgttagcagcagctacttaacctggtatcagcagaaa
IGKV3-7*01   tgtctttgtctccaggggaaagagtcaccctctcctgcagggccagtcagagtgttagcagcagctacttaacctggtatcagcagaaa
IGKV3-7*02   tgtctttgtctccaggggaaagagtcaccctctcctgcagggccagtcagagtgttagcagcagctactttcctggtaccagcagaaa
IGKV3-7*03   tgtctttgtctccaggggaaagagtcaccctctcctgcagggccagtcagagtgttagcagcagctacttaacctggtatcagcagaaa
IGKV3-7*04   tgtctttgtctccaggggaaagagtcaccctctcctgcagggccagtcagagtgttagcagcagctacttaacctggtatcagcagaaa
V_V3-7*02    tgtctttgtctccaggggaaagagtcaccctctcctgcagggccagtcagagtgttagcagcagctactttcctggtaccagcagaaa
V_V3-7*01    tgtctttgtctccaggggaaagagtcaccctctcctgcagggccagtcagagtgttagcagcagctacttaacctggtatcagcagaaa
```

```
V3-7_01        cctggccaggcgcccaggctcctcatctatggtgcatccaccagggccactagcatcccagccaggttcagtggcagtgggtctgggac
V3-7_02        cctggccaggcgcccaggctcctcatctatggtgcatccaccagggccactggcatcccagccaggttcagtggcagtgggtctgggac
V3-7_03        cctggccaggcgcccaggctcctcatctatggtgcatccaccagggccactagcatcccagccaggttcagtggcagtgggtctgggac
V3-7_04        cctggccaggcgcccaggctcctcatctatggtgcatccaccagggccactagcatcccagccaggttcagtggcagtgggtctgggac
V3-7_05        cctggccaggcgcccaggctcctcatctatggtgcatccaccagggccactagcatcccagccaggttcagtggcagtgggtctgggac
V3-7_06        cctggccaggcgcccaggctcctcatctatggtgcatccaccagggccactggcatcccagccaggttcagtggcagtgggtctgggac
V3-7_07        cctggccaggcgcccaggctcctcatctatggtgcatccaccagggcctctagcatcccagccaggttcagtggcagtgggtctgggac
V3-7_08        cctggccaggcgcccaggctcctcatctatggtgcatccaccagggccactagcatcccagccaggttcagtggcagtgggtctgggac
V3-7_09        cctggccaggctcccaggctcctcatctatggtgcatccaccagggccactagcatcccagccaggttcagtggcagtgggtctgggac
V3-7_10        cctggccaggcgcccaggctcctcatctatggtgcatccaccagggccactagcatcccagccaggttcagtggcagtgggtctgggac
IGKV3-7*01     cctggccaggcgcccaggctcctcatctatggtgcatccaccagggccactagcatcccagccaggttcagtggcagtgggtctgggac
IGKV3-7*02     cctgggcaggctcccaggctcctcatctatggtgcatccaccagggccactggcatcccagccaggttcagtggcagtgggtctgggac
IGKV3-7*03     cctggccaggcgcccaggctcctcatctatggtgcatccaccagggccactagcatcccagccaggttcagtggcagtgggtctgggag
IGKV3-7*04     cctggccaggcgcccaggctcctcatctatggtgcatccaccagggccactagcatcccagccaggttcagtggcagtgggtctgggac
V_V3-7*02      cctgggcaggctcccaggctcctcatctatggtgcatccaccagggccactggcatcccagccaggttcagtggcagtgggtctgggac
V_V3-7*01      cctggccaggcgcccaggctcctcatctatggtgcatccaccagggccactagcatcccagccaggttcagtggcagtgggtctgggac

V3-7_01        agacttcactctcaccatcagcagcctgcagcctgaagattttgcagtttattactgtcagcaggattataacttacct
V3-7_02        agacttcactctcaccatcagcagcctgcagcctgaagattttgcagtttattactgtcagcaggattataacttacct
V3-7_03        agacttcactctcaccatcagcagcctgcagcctgaagattttgcagtttattactgtcagcaggattataacttacct
V3-7_04        agacttcactctcaccatcagcagcctgcagcctgaagattttgcagtttattactgtcagcaggattataacttacct
V3-7_05        agacttcactctcaccatcagcagcctgcagcctgaagattttgcagtttattactgtcagcaggattataacttacct
V3-7_06        agacttcactctcaccatcagcagcctgcagcctgaagattttgcagtttattactgtcagcaggattataacttacct
V3-7_07        agacttcactctcaccatcagcagcctgcagcctgaagattttgcagtttattactgtcagcaggattataacttacct
V3-7_08        agacttcactctcaccatcagcagcctgcagcctgaagattttgcagtttattactgtcagcaggattataacttacct
V3-7_09        agacttcactctcaccatcagcagcctgcagcctgaagattttgcagtttattactgtcagcaggattataacttacct
V3-7_10        agacttcactctcaccatcagcagcctgcagcctgaagattttgcagtttattactgtcagcaggattataacttacct
IGKV3-7*01     agacttcactctcaccatcagcagcctgcagcctgaagattttgcagtttattactgtcagcaggatcataacttacct
IGKV3-7*02     agacttcactctcaccatcagcagcctgcagcctgaagattttgcagtttattactgtcagcaggattataacttacct
IGKV3-7*03     agacttcactctcaccatcagcagcctgcagcctgaagattttgcagtttattactgtcagcaggatcataacttacct
IGKV3-7*04     agacttcactctcaccatcagcagcctgcagcctgaagattttgcagtttattactgtcagcaggattataacttacct
V_V3-7*02      agacttcactctcaccatcagcagcctgcagcctgaagattttgcagtttattactgtcagcaggattataactta---
V_V3-7*01      agacttcactctcaccatcagcagcctgcagcctgaagattttgcagtttattactgtcagcaggattataactta---
```

IGKV3-11

```
V3-11_01         atggaagccccagctcagcttctcttcctcctgctactctggctcccagataccaccggagaaattgtgttgacacagtctccagccaccc
V3-11_02         atggaagccccagctcagcttctcttcctcctgctactctggctcccagataccaccggagaaattgtgttgacacagtctccagccaccc
V3-11_03         atggaagccccagctcagcttctcttcctcctgctactctggctcccagataccactggagaaattgtgttgacacagtctccagccaccc
IGKV3-11*01      atggaagccccagctcagcttctcttcctcctgctactctggctcccagataccaccggagaaattgtgttgacacagtctccagccaccc
IGKV3-11*02      atggaagccccagctcagcttctcttcctcctgctactctggctcccagataccaccggagaaattgtgttgacacagtctccagccaccc
IP_V3-11*p03     ------------------------------------------------------------gaaattgtgttgacacagtctccagccaccc
IP_V3-11*p04     ------------------------------------------------------------gaaattgtgttgacacagtctccagccaccc
IP_V3-11*p05     ------------------------------------------------------------gaaattgtgttgacacagtctccagccaccc
V_V3-11*01       ------------------------------------------------------------gaaattgtgttgacacagtctccagccaccc

V3-11_01         tgtctttgtctccaggggaaagagccaccctctcctgcagggccagtcagagtgttagcagctacttagcctggtaccaacagaaacct
V3-11_02         tgtctttgtctccaggggaaagagccaccctctcctgcagggccagtcagagtgttagcagctacttagcctggtaccaacagaaacct
V3-11_03         tgtctttgtctccaggggaaagagccaccctctcctgcagggccagtcagagtgttagcagctacttagcctggtaccaacagaaacct
IGKV3-11*01      tgtctttgtctccaggggaaagagccaccctctcctgcagggccagtcagagtgttagcagctacttagcctggtaccaacagaaacct
IGKV3-11*02      tgtctttgtctccaggggaaagagccaccctctcctgcagggccagtcagagtgttagcagctacttagcctggtaccaacagaaacct
IP_V3-11*p03     tgtctttgtctccaggggaaagagccaccctctcctgcagggccagtcagagtgttagcagctacttagcctggtaccaacagaaacct
IP_V3-11*p04     tgtctttgtctccaggggaaagagccaccctctcctgcagggccagtcagagtgttagcagctacttagcctggtaccaacagaaacct
IP_V3-11*p05     tgtctttgtctccaggggaaagagccaccctctcctgcagggccagtcagagtgttagcagctacttagcctggtaccaacagaaacct
V_V3-11*01       tgtctttgtctccaggggaaagagccaccctctcctgcagggccagtcagagtgttagcagctacttagcctggtaccaacagaaacct

V3-11_01         ggccaggctcccaggctcctcatctatgatgcatccaacagggccactggcatcccagccaggttcagtggcagtgggtctgggacagact
V3-11_02         ggccaggctcccaggctcctcatctatgatgcatccaacagggccactggcatcccagccaggttcagtggcagtgggtctgggacagact
V3-11_03         ggccaggctcccaggctcctcatctatgatgcatccaacagggccactggcatcccagccaggttcagtggcagtgggtctgggacagact
IGKV3-11*01      ggccaggctcccaggctcctcatctatgatgcatccaacagggccactggcatcccagccaggttcagtggcagtgggtctgggacagact
IGKV3-11*02      ggccaggctcccaggctcctcatctatgatgcatccaacagggccactggcatcccagccaggttcagtggcagtgggtctggggagact
IP_V3-11*p03     ggccaggctcccaggctcctcatctatgatgcatccaacagggccactggcatcccagccaggttcagtggcagtgggtctgggacagact
IP_V3-11*p04     ggccaggctcccaggctcctcatctatgatgcatccaacagggccactggcatcccagccaggttcagtggcagtgggtctgggacagact
IP_V3-11*p05     ggccaggctcccaggctcctcatctatgatgcatccaacagggccactggcatcccagccaggttcagtggcagtgggtctgggacagact
V_V3-11*01       ggccaggctcccaggctcctcatctatgatgcatccaacagggccactggcatcccagccaggttcagtggcagtgggtctgggacagact

V3-11_01         tcactctcaccatcagcagcctagagcctgaagattttgcagtttattactgtcagcagcgtagcaactggcct
V3-11_02         tcactctcaccatcagcagcctagagcctgaagattttgcagtttattactgtcagcagcatagcaactggcct
V3-11_03         tcactctcaccatcagcagcctagagcctgaagattttgcagtttattactgtcagcagcgtagcaactggcct
IGKV3-11*01      tcactctcaccatcagcagcctagagcctgaagattttgcagtttattactgtcagcagcgtagcaactggcct
IGKV3-11*02      tcactctcaccatcagcagcctagagcctgaagattttgcagtttattactgtcagcagcgtagcaactggcct
IP_V3-11*p03     tcactctcaccatcagcagcctagagcctgaagattttgcagtttattactgtcagcagcgtagcacctcgcct
IP_V3-11*p04     tcactctcaccatcagcagcctagagcctgaagattttgcagtttattactgtcagcagcgtaggacttggcct
IP_V3-11*p05     tcactctcaccatcagcagcctagagcctgaagattttgcagtttattactgtcagcagcgtagcaaccggcct
V_V3-11*01       tcactctcaccatcagcagcctagagcctgaagattttgcagtttattactgtcagcagcgtagcaactgg---
```

IGKV3-15

```
V3-15_01       atggaagccccagcgcagcttctcttcctcctgctactctggctcccagataccactggagaaatagtgatgacgcagtctccagccaccct
V3-15_02       atggaagccccagcgcagcttctcttcctcctgctactctggctcccagataccactggagaaatagtgatgacgcagtctccagccaccct
V3-15_03       atggaagccccagcgcagcttctcttcctcctgctactctggctcccagataccactggagaaatagtgatgacgcagtctccagccaccct
V3-15_04       atggaagccccagcgcagcttctcttcctcctgctactctggctcccagataccactggagaaacagtgatgacacagtctccagccaccct
V3-15_05       atggaagccccagcgcagcttctcttcctcctgctactctggctcccagataccactggagaaatagtgatgacgcagtctccagccaccct
V3-15_06       atggaagccccagcgcagcttctcttcctcctgctactctggctcccagataccactggagaaatagtgatgacgcagtctccagccaccct
IGKV3-15*01    atggaagccccagcgcagcttctcttcctcctgctactctggctcccagataccactggagaaatagtgatgacgcagtctccagccaccct
IP_V3-15*p02   ------------------------------------------------------------gaaatagtgatgacgcagtctccagccaccct
IP_V3-15*p03   ------------------------------------------------------------gaaatagtgatgacgcagtctccagccaccct
V_V3-15*01     ------------------------------------------------------------gaaatagtgatgacgcagtctccagccaccct

V3-15_01       gtctgtgtctccaggggaaagagccaccctctcctgcagggccagtcagagtgttagcagcaacttagcctggtaccagcagaaacct
V3-15_02       gtctgtgtctccaggggaaagagccaccctctcctgcagggccagtcagagtgttagcagcaacttagcctggtaccagcagaaacct
V3-15_03       gtctgtgtctccaggggaaagagccaccctctcctgcagggccagtcagagtgttagcagcaacttggcctggtaccagcagaaacct
V3-15_04       gtctgtgtctccaggggaaagagccaccctctcctgcagggccagtcagagtgttagcagcaacttagcctggtaccagcagaaacct
V3-15_05       gtctgtgtctccaggggaaagagccaccctctcctgcagggccagtcagagtgttagcagcaacttagcctggtaccagcagaaacct
V3-15_06       gtctgtgtctccaggggaaagagccaccctctcctgcagggccagtcagagtgttagcagcaacttagcctggtaccagcagaaacct
IGKV3-15*01    gtctgtgtctccaggggaaagagccaccctctcctgcagggccagtcagagtgttagcagcaacttagcctggtaccagcagaaacct
IP_V3-15*p02   gtctgtgtctccaggggaaagagccaccctctcctgcagggccagtcagagtgttagcagcaacttagcctggtaccagcagaaacct
IP_V3-15*p03   gtctgtgtctccaggggaaagagccaccctctcctgcagggccagtcagagtgttagcagcaacttagcctggtaccagcagaaacct
V_V3-15*01     gtctgtgtctccaggggaaagagccaccctctcctgcagggccagtcagagtgttagcagcaacttagcctggtaccagcagaaacct

V3-15_01       ggccaggctcccaggctcctcatctatggtgcatccaccagggccactggtatcccagccaggttcagtggcagtgggtctgggacagagt
V3-15_02       ggccaggctcccaggctcctcatctatggtgcatccaccagggccactggtatcccagccaggttcagtggcagtgggtctgggacagagt
V3-15_03       ggccaggctcccaggctcctcatctatggtgcatccaccagggccactggtatcccagccaggttcagtggcagtgggtctgggacagagt
V3-15_04       ggccaggctcccaggctcctcatctatggtgcatccaccagggccactggtatcccagccaggttcagtggcagtgggtctgggacagagt
V3-15_05       gggcaggctcccaggctcctcatctatggtgcatccaccagggccactggtatcccagccaggttcagtggcagtgggtctgggacagagt
V3-15_06       ggccaggctcccaggctcctcatctatggtgcatccaccagggccactggtatcccagccaggttcagtggcagtgggtctgggacagagt
IGKV3-15*01    ggccaggctcccaggctcctcatctatggtgcatccaccagggccactggtatcccagccaggttcagtggcagtgggtctgggacagagt
IP_V3-15*p02   ggccaggctcccaggctcctcatctatggtgcatccaccagggccactggtatcccagccaggttcagtggcagtgggtctgggacagagt
IP_V3-15*p03   ggccaggctcccaggctcctcatctatggtgcatccaccagggccactggtatcccagccaggttcagtggcagtgggtctgggacagagt
V_V3-15*01     ggccaggctcccaggctcctcatctatggtgcatccaccagggccactggtatcccagccaggttcagtggcagtgggtctgggacagagt
```

```
V3-15_01        tcactctcaccatcagcagcctgcagtctgaagattttgcagtttattactgtcagcagtataataactggcct--
V3-15_02        tcactctcaccatcagcagcatgcagtctgaagattttgcagtttattactgtcagcagtataataactggcct--
V3-15_03        tcactctcaccatcagcagcctgcagtctgaagattttgcagtttattactgtcagcagtataataactggcct--
V3-15_04        tcactctcaccatcagcagcctgcagtctgaagattttgcagtttattactgtcagcagtataataactggcct--
V3-15_05        tcactctcaccatcagcagcctgcagtctgaagattttgcagtttattactgtcagcagtataataactggcct--
V3-15_06        tcactctcaccatcagcagcctgcagtctgaagattttgcagtttattactgtcagcagtataatacctggcct--
IGKV3-15*01     tcactctcaccatcagcagcctgcagtctgaagattttgcagtttattactgtcagcagtataataactggcctcc
IP_V3-15*p02    tcactctcaccatcagcagcctgcagtctgaagattttgcagtttattactgtcagcagtatggcgactggcctcc
IP_V3-15*p03    tcactctcaccatcagcagcatgcagtctgaagattttgcagtttattactgtcagcagtataataactggcctcc
V_V3-15*01      tcactctcaccatcagcagcctgcagtctgaagattttgcagtttattactgtcagcagtataataactgg-----
```

IGKV3-20

```
V3-20_01        atggaaaccccagcgcagcttctcttcctcctgctactctggctcccagataccaccggagaaattgtgttgacgcagtctccaggcaccc
V3-20_02        atggaaaccccagcgcagcttctcttcctcctgctactctggctcccagataccaccggagaaattgtgttgacgcagtctccaggcaccc
IGKV3-20*01     atggaaaccccagcgcagcttctcttcctcctgctactctggctcccagataccaccggagaaattgtgttgacgcagtctccaggcaccc
IGKV3-20*02     ------------------------------------------------------------gaaattgtgttgacacagtctccagccaccc
IP_V3-20*p03    ------------------------------------------------------------gaaattgtgttgacgcagtctccaggcaccc
IP_V3-20*p04    ------------------------------------------------------------gaaattgtgttgacgcagtctccaggcaccc
IP_V3-20*p05    ------------------------------------------------------------gaaattgtgttgacgcagtctccaggcaccc
IP_V3-20*p06    ------------------------------------------------------------gaaattgtgttgacgcagtctccaggcaccc
V_V3-20*01      ------------------------------------------------------------gaaattgtgttgacgcagtctccaggcaccc

V3-20_01        tgtctttgtctccaggggaaagagccaccctctcctgcagggccagtcagagtgttagcagcagctacttagcctggtaccagcagaaa
V3-20_02        tgtctttgtctccaggggaaagagccaccctctcctgcagggccagtcagagtgttagcagcagctacttagcctggtaccaacagaaa
IGKV3-20*01     tgtctttgtctccaggggaaagagccaccctctcctgcagggccagtcagagtgttagcagcagctacttagcctggtaccagcagaaa
IGKV3-20*02     tgtctttgtctccaggggaaagagccaccctctcctgcagggccagtcagagtgttagcagcagctacttagcctggtaccagcagaaa
IP_V3-20*p03    tgtctttgtctccaggggaaagagccaccctctcctgcagggccagtcagagtgttagcagcagctacttagcctggtaccagcagaaa
IP_V3-20*p04    tgtctttgtctccaggggaaagagccaccctctcctgcagggccagtcagagtgttagcagcagctacttagcctggtaccagcagaaa
IP_V3-20*p05    tgtctttgtctccaggggaaagagccaccctctcctgcagggccagtcagagtgttagcagcagctacttagcctggtaccagcagaaa
IP_V3-20*p06    tgtctttgtctccaggggaaagagccaccctctcctgcagggccagtcagagtgttagcagcagctacttagcctggtaccagcagaaa
V_V3-20*01      tgtctttgtctccaggggaaagagccaccctctcctgcagggccagtcagagtgttagcagcagctacttagcctggtaccagcagaaa

V3-20_01        cctggccaggctcccaggctcctcatctatggtgcatccagcagggccactggcatcccagacaggttcagtggcagtgggtctgggacag
V3-20_02        cctggccaggctcccaggctcctcatctatggtgcatccagcagggccactggcatcccagacaggttcagtggcagtgggtctgggacag
IGKV3-20*01     cctggccaggctcccaggctcctcatctatggtgcatccagcagggccactggcatcccagacaggttcagtggcagtgggtctgggacag
IGKV3-20*02     cctggccaggctcccaggctcctcatctatggtgcatccagcagggccactggcatcccagcaaggttcagtggcagtgggtctgggacag
IP_V3-20*p03    cctggccaggctcccaggctcctcatctatggtgcatccagcagggccactggcatcccagacaggttcagtggcagtgggtctgggacag
IP_V3-20*p04    cctggccaggctcccaggctcctcatctatggtgcatccagcagggccactggcatcccagacaggttcagtggcagtgggtctgggacag
IP_V3-20*p05    cctggccaggctcccaggctcctcatctatggtgcatccagcagggccactggcatcccagacaggttcagtggcagtgggtctgggacag
IP_V3-20*p06    cctggccaggctcccaggctcctcatctatggtgcatccagcagggccactggcatcccagacaggttcagtggcagtgggtctgggacag
V_V3-20*01      cctggccaggctcccaggctcctcatctatggtgcatccagcagggccactggcatcccagacaggttcagtggcagtgggtctgggacag

V3-20_01        acttcactctcaccatcagcagactggagcctgaagattttgcagtgtattactgtcagcagtatggtagctcacct--
V3-20_02        acttcactctcaccatcagcagactggagcctgaagattttgcagtgtattactgtcagcagtatggtagctcacct--
IGKV3-20*01     acttcactctcaccatcagcagactggagcctgaagattttgcagtgtattactgtcagcagtatggtagctcacctcc
IGKV3-20*02     acttcactctcaccatcagcagactggagcctgaagattttgcagttattactgtcagc-------------------
IP_V3-20*p03    acttcactctcaccatcagcagactggagcctgaagattttgcagtgtattactgtcagcagtatggtagctcccctcc
IP_V3-20*p04    acttcactctcaccatcagcagactggagcctgaagattttgcagtgtattactgtcagcagtatggtagctctcctcc
IP_V3-20*p05    acttcactctcaccatcagcagactggagcctgaagattttgcagtgtattactgtcagcagtatggtagcccacctcc
IP_V3-20*p06    acttcactctcaccatcagcagactggagcctgaagattttgcagtgtattactgtcagcagtatggtagcccccctcc
V_V3-20*01      acttcactctcaccatcagcagactggagcctgaagattttgcagtgtattactgtcagcagtatggtagctca-----
```

IGKV3D-7

```
V3D-7_01        atggaaccatggaagccccagcacagcttcttcttcctcctgctactctggctcccagataccaccggagaaattgtaatgacacagtctc
IGKV3D-7*01     atggaaccatggaagccccagcacagcttcttcttcctcctgctactctggctcccagataccaccggagaaattgtaatgacacagtctc
V_V3D-7*01      ---------------------------------------------------------------------gaaattgtaatgacacagtctc

V3D-7_01        cagccaccctgtctttgtctccaggggaaagagccaccctctcctgcagggccagtcagagtgttagcagcagctacttatcctggtac
IGKV3D-7*01     cagccaccctgtctttgtctccaggggaaagagccaccctctcctgcagggccagtcagagtgttagcagcagctacttatcctggtac
V_V3D-7*01      cagccaccctgtctttgtctccaggggaaagagccaccctctcctgcagggccagtcagagtgttagcagcagctacttatcctggtac

V3D-7_01        cagcagaaacctgggcaggctcccaggctcctcatctatggtgcatccaccagggccactggcatcccagccaggttcagtggcagtggg
IGKV3D-7*01     cagcagaaacctgggcaggctcccaggctcctcatctatggtgcatccaccagggccactggcatcccagccaggttcagtggcagtggg
V_V3D-7*01      cagcagaaacctgggcaggctcccaggctcctcatctatggtgcatccaccagggccactggcatcccagccaggttcagtggcagtggg

V3D-7_01        tctgggacagacttcactctcaccatcagcagcctgcagcctgaagattttgcagtttattactgtcagcaggattataacttacct
IGKV3D-7*01     tctgggacagacttcactctcaccatcagcagcctgcagcctgaagattttgcagtttattactgtcagcaggattataacttacct
V_V3D-7*01      tctgggacagacttcactctcaccatcagcagcctgcagcctgaagattttgcagtttattactgtcagcaggattataactta---
```

IGKV3D-11

```
V3D-11_01      atggaagccccagcgcagcttctcttcctcctgctactctggctcccagataccaccggagaaattgtgttgacacagtctccagccacc
V3D-11_02      atggaagccccagcgcagcttctcttcctcctgctactctggctcccagataccaccggagaaattgtgttgacacagtctccagccacc
V3D-11_03      atggaagccccagcgcagcttctcttcctcctgctactctggctcccagataccaccggataaattgtgttgacacagtctccagccacc
V3D-11_04      atggaagccccagcgcagcttctcttcctcctgctactctagctcccagataccaccggagaaattgtgttgacacagtctccagccacc
V3D-11_05      atggaagccccagcgcagcttctcttcctcctgctactctggctcccagataccaccggagaaattgtgttgacacagtctccagccacc
V3D-11_06      atggaagccccagcgcagcttctcttcctcctgctactctggctcccagataccaccggagaaattgtgttgacacagtctccagccacc
V3D-11_07      atggaagccccagcgcagcttctcttcctcctgctactctggctcccagataccaccggagaaatttgttgacacagtctccagccacc
IGKV3D-11*01   atggaagccccagcgcagcttctcttcctcctgctactctggctcacagataccaccggagaaattgtgttgacacagtctccagccacc
IGKV3D-11*02   atggaagccccagcgcagcttctcttcctcctgctactctggctcccagataccaccggagaaattgtgttgacacagtctccagccacc
IGKV3D-11*03   -------------------------acctcctgctactctggctcccagataccaccggagaaattgtgttgacacagtctccagccacc
V_V3D-11*01    ------------------------------------------------------------gaaattgtgttgacacagtctccagccacc

V3D-11_01      ctgtctttgtctccaggggaaagagccaccctctcctgcaggccagtcagagtgttagcagctacttagcctggtaccagcagaaacct
V3D-11_02      ctgtctttgtctccaggggaaagagccaccctctcctgcaggccagtcaggtgttagcagctacttagcctggtaccagcagaaacct
V3D-11_03      ctgtctttgtctccaggggaaagagccaccctctcctgcaggccagtcaggtgttagcagctacttagcctggtaccagcagaaacct
V3D-11_04      ctgtctttgtctccaggggaaagagccaccctctcctgcaggccagtcagagtgttagcagctacttagcctggtaccagcagaaacct
V3D-11_05      ctgtctttgtctccaggggaaagagccaccctctcctgcaggccagtcagagtgttagcagctacttagcctggtaccagcagaaacct
V3D-11_06      ctgtctttgtctccaggggaaagagccaccctctcctgcaggccagtcaggtgttagcagctacttagcctggtaccagcagaaacct
V3D-11_07      ctgtctttgtctccaggggaaagagccaccctctcctgcaggccagtcaggtgttagcagctacttagcctggtaccagcagaaacct
IGKV3D-11*01   ctgtctttgtctccaggggaaagagccaccctctcctgcaggccagtcaggtgttagcagctacttagcctggtaccagcagaaacct
IGKV3D-11*02   ctgtctttgtctccaggggaaagagccaccctctcctgcaggccagtcagagtgttagcagctacttagcctggtaccagcagaaacct
IGKV3D-11*03   ctgtctttgtctccaggggaaagagccaccctctcctgcaggccagtcaggtgttagcagcaacttagcctggtaccagcagaaacct
V_V3D-11*01    ctgtctttgtctccaggggaaagagccaccctctcctgcaggccagtcaggtgttagcagctacttagcctggtaccagcagaaacct

V3D-11_01      ggccaggctcccaggctcctcatctatgatgcatccaacagggccactggcatcccagccaggttcagtggcagtgggcctgggacagacttc
V3D-11_02      ggccaggctcccaggctcctcatctatgatgcatccaacagggccactggcatcccagccaggttcagtggcagtgggcctgggacagacttc
V3D-11_03      ggccaggctcccaggctcctcatctatgatgcatccaacagggccactggcatcccagccaggttcagtggcagtgggcctgggacagacttc
V3D-11_04      ggccaggctcccaggctcctcatctatgatgcatccaacagggccactggcatcccagccaggttcagtggcagtgggcctgggacagacttc
V3D-11_05      ggccaggctcccaggctcctcatctatgatgcatccaacagggccactggcatcccagccaggttcagtggcagtgggcctgggacagacttc
V3D-11_06      ggccaggctcccaggctcctcatctatgatgcatccaacagggccacCggcatcccagccaggttcagtggcagtgggcctgggacagacttc
V3D-11_07      ggccaggctcccaggctcctcatctatgatgcatccaacagggccactggcatcccagccaggttcagtggcagtgggcctgggacagacttc
IGKV3D-11*01   ggccaggctcccaggctcctcatctatgatgcatccaacagggccactggcatcccagccaggttcagtggcagtgggcctgggacagacttc
IGKV3D-11*02   ggccaggctcccaggctcctcatctatgatgcatccaacagggccactggcatcccagccaggttcagtggcagtgggcctgggacagacttc
IGKV3D-11*03   ggccaggctcccaggctcctcatctatgatgcatccaacagggccactggcatcccagccaggttcagtggcagtgggcctgggacagacttc
V_V3D-11*01    ggccaggctcccaggctcctcatctatgatgcatccaacagggccactggcatcccagccaggttcagtggcagtgggcctgggacagacttc
```

```
V3D-11_01       actctcaccatcagcagcctagagcctgaagattttgcagtttattactgtcagcagcgtagcaactggcat
V3D-11_02       actctcaccatcagcagcctagagcctgaagattttgcagtttattactgtcagcagcgtagcaactggcat
V3D-11_03       actctcaccatcagcagcctagagcctgaagattttgcagtttattactgtcagcagcgtagcaactggcat
V3D-11_04       actctcaccatcagcagcctagagcctgaagattttgcagtttattactgtcagcagcgtagcaactggcat
V3D-11_05       actctcaccatcagcagcctagagcctgaagattttgcagtttattactgtcagcagcatagcaactggcat
V3D-11_06       actctcaccatcagcagcctagagcctgaagattttgcagtttattactgtcagcagcgtagcaactggcat
V3D-11_07       actctcaccatcagcagcctagagcctgaagattttgcagtttattactgtcagcagcgtagcaactggcat
IGKV3D-11*01    actctcaccatcagcagcctagagcctgaagattttgcagtttattactgtcagcagcgtagcaactggcat
IGKV3D-11*02    actctcaccatcagcagcctagagcctgaagattttgcagtttattactgtcagcagcgtagcaactggcat
IGKV3D-11*03    actctcaccatcagcagcctagagcctgaagattttgcagtgtattactgtcagcagcgtagcaactggcat
V_V3D-11*01     actctcaccatcagcagcctagagcctgaagattttgcagtttattactgtcagcagcgtagcaactgg---
```

IGKV3D-15

V3D-15_01	atggaagccccagcgcagcttctcttcctcctgctactctggctcccagataccactggagaaatagtgatgacgcagtctccagccacc
V3D-15_02	atggaagccccagcgcagcttctcttcctcctgctactctggctcccagataccactggagaaatagtgatgatgcagtctccagccacc
V3D-15_03	atggaagccccagcgcagcttctcttcctcctgctactctggctcccagataccactggagaaatagtgatgacgcagtctccagccacc
V3D-15_04	atggaagccccagcgcagcttctcttcctcctgctactctggctcccagataccactggagaaatagtgatgatgcagtctccagccacc
V3D-15_05	atggaagccccagcgcagcttctcttcctcctgctactctggctcccagataccactggagaaatagtgatgacgcagtctccagccacc
V3D-15_06	atggaagccccagcgcagcttctcttcctcctgctactctggctcccagataccactggagaaatagtgatgacgcagtctccagccacc
V3D-15_07	atggaagccccagcgcagcttctcttcctcctgctagtctggctcccagataccactggagaaatagtgatgacgcagtctccagccacc
V3D-15_08	atggaagccccagcgcagcttctcttcctcctgctactctggctcccagataccactggagaaatagtgatgacgcagtctccagccacc
IGKV3D-15*01	atggaagccccagcgcagcttctcttcctcctgctactctggctcccagataccactggagaaatagtgatgacgcagtctccagccacc
IGKV3D-15*02	atggaagccccagcgcagcttctcttcctcctgctactctggctcccagataccactggagaaatagtgatgatgcagtctccagccacc
IGKV3D-15*03	atggaagccccagcgcagcttctcttcctcctgctactctggctcccagataccactggagaaatagtgatgacgcagtctccagccacc
V_V3D-15*02	--gaaatagtgatgatgcagtctccagccacc
V_V3D-15*01	--gaaatagtgatgacgcagtctccagccacc

V3D-15_01	ctgtctgtgtctccaggggaaagagccaccctctcctgcagggccagtcagagtgttagcagcaacttagcctggtaccagcagaaacct
V3D-15_02	ctgtctgtgtctccaggggaaagagccaccctctcctgcagggccagtcagagtgttagcagcaacttagcctggtaccagcagaaacct
V3D-15_03	ctgtctgtgtctccaggggaaagagccaccctctcctgcagggccagtcagagtgttagcagcaacttagcctggtaccagcagaaacct
V3D-15_04	ctgtctgtgtctccaggggaaagagccaccctctcctgcagggccagtcagagtgttagcagcaacttagcctggtaccagcagaaacct
V3D-15_05	ctgtctgtgtctccaggggaaagagccaccctctcctgcagggccagtcagagtgttagcagcaacttagcctggtaccagcagaaacct
V3D-15_06	ctgtctgtgtctccaggggaaagagccaccctctcctgcagggccagtcagagtgttagcagcaacttagcctggtaccagcagaaacct
V3D-15_07	ctgtctgtgtctccaggggaaagagccaccctctcctgcagggccagtcagagtgttagcagcaacttagcctggtaccagcagaaacct
V3D-15_08	ctgtctgtgtctccaggggaaagagccaccctctcctgcagggccagtcagagtgttagcagcaacttagcctggtaccagcagaaacct
IGKV3D-15*01	ctgtctgtgtctccaggggaaagagccaccctctcctgcagggccagtcagagtgttagcagcaacttagcctggtaccagcagaaacct
IGKV3D-15*02	ctgtctgtgtctccaggggaaagagccaccctctcctgcagggccagtcagagtgttagcagcaacttagcctggtaccagcagaaacct
IGKV3D-15*03	ctgtctgtgtctccaggggaaagagccaccctctcctgcagggccagtcagagtgttagcagcaacttagcctggtaccagcagaaacct
V_V3D-15*02	ctgtctgtgtctccaggggaaagagccaccctctcctgcagggccagtcagagtgttagcagcaacttagcctggtaccagcagaaacct
V_V3D-15*01	ctgtctgtgtctccaggggaaagagccaccctctcctgcagggccagtcagagtgttagcagcaacttagcctggtaccagcagaaacct

V3D-15_01	ggccaggctcccaggctcctcatctatggtgcatccaccagggccactggcatcccagccaggttcagtggcagtgggtctgggacaga
V3D-15_02	ggccaggctcccaggctcctcatctatggtgcatccaccagggccactggcatcccagccaggttcagtggcagtgggtctgggacaga
V3D-15_03	ggccaggctcccaggctcctcatctatggtgcatccatcagggccactggcatcccagccaggttcagtggcagtgggtctgggacaga
V3D-15_04	ggccaggctcccaggctcctcatctatggtgcatccaccagggccactggcatcccagccaggttcagtggcagtgggtctgggacaga
V3D-15_05	ggccaggctcccaggctcctcatctatggtgcatccatcagggccactggcatcccagccaggttcagtggcagtgggtctgggacaga
V3D-15_06	ggccaggctcccaggctcctcatctatggtgcatccatcagggccactggcatcccagccaggttcagtggcagtggatctgggacaga
V3D-15_07	ggccaggctcccaggctcctcatctatggtgcatccaccagggccactggcatcccagccaggttcagtggcagtgggtctgggacaga
V3D-15_08	ggccaggctcccaggctcctcatctatggtgcatccaccagggccattggcatcccagccaggttcagtggcagtgggtctgggacaga
IGKV3D-15*01	ggccaggctcccaggctcctcatctatggtgcatccaccagggccactggcatcccagccaggttcagtggcagtgggtctgggacaga
IGKV3D-15*02	ggccaggctcccaggctcctcatctatggtgcatccaccagggccactggcatcccagccaggttcagtggcagtgggtctgggacaga
IGKV3D-15*03	ggccaggctcccaggctcctcatctatggtgcatccatcagggccactggcatcccagccaggttcagtggcagtgggtctgggacaga
V_V3D-15*02	ggccaggctcccaggctcctcatctatggtgcatccaccagggccactggcatcccagccaggttcagtggcagtgggtctgggacaga
V_V3D-15*01	ggccaggctcccaggctcctcatctatggtgcatccaccagggccactggcatcccagccaggttcagtggcagtgggtctgggacaga

V3D-15_01	gttcactctcaccatcagcagcctgcagtctgaagattttgcagtttattactgtcagcagtataataactgggcct
V3D-15_02	gttcactctcaccatcagcagcctgcagtctgaagattttgcagtttattactgtcagcagtataataactgacct
V3D-15_03	gttcactctcaccatcagcatcctgcagtctgaagattttgcagtttattactgtcagcagtataataactgggcct
V3D-15_04	gttcactctcaccatcagcagcctgcagtctgaagattttgcagtttattactgtcagcagtataataactgggcct
V3D-15_05	gttcactctcaccatcagcagcctgcagtctgaagattttgcagtttattactgtcagcagtataataactgggcct
V3D-15_06	gttcactctcaccatcagcatcctgcagtctgaagattttgcagtttattactgtcagcagtataataactgggcct
V3D-15_07	gttcactctcaccatcagcagcctgcagtctgaagattttgcagtttattactgtcagcagtataataactgggcct
V3D-15_08	gttcactctcaccatcagcagcctgcagtctgaagattttgcagtttattactgtcagcagtataataactgggcct
IGKV3D-15*01	gttcactctcaccatcagcagcctgcagtctgaagattttgcagtttattactgtcagcagtataataactgggcct
IGKV3D-15*02	gttcactctcaccatcagcagcctgcagtctgaagattttgcagtttattactgtcagcagtataataactgacct
IGKV3D-15*03	gttcactctcaccatcagcatcctgcagtctgaagattttgcagtttattactgtcagcagtataataactgggcct
V_V3D-15*02	gttcactctcaccatcagcagcctgcagtctgaagattttgcagtttattactgtcagcagtataataactga---
V_V3D-15*01	gttcactctcaccatcagcagcctgcagtctgaagattttgcagtttattactgtcagcagtataataactggg---

IGKV3D-20

```
V3D-20_01       atggaaaccccagcgcagcttctcttcctcctgctactctggctcccagataccaccggagaaattgtgttgacgcagtctccagcca
V3D-20_02       atgggaaccccagcgcagcttctcttcctcctgctactctggctcccagataccaccggagaaattgtgttgacgcagtctccagcca
V3D-20_03       atggaaaccccagcgcagcttctcttcctcctgctactctggctcccagataccaccggagaaattgtgttgacgcagtctccagcca
IGKV3D-20*01    atggaaaccccagcgcagcttctcttcctcctgctactctggctcccagataccaccggagaaattgtgttgacgcagtctccagcca
IGKV3D-20*02    -------------------------acctcctgctactctggctcccagataccaccggagaaattgtgttgacgcagtctccagcca
V_V3D-20*01     ------------------------------------------------------------gaaattgtgttgacgcagtctccagcca

V3D-20_01       ccctgtctttgtctccaggggaaagagccaccctctcctgcgggccagtcagagtgttagcagcagctacttagcctggtaccagcagaaa
V3D-20_02       ccctgtctttgtctccaggggaaagagccaccctctcctgcgggccagtcagagtgttagcagcagctacttagcctggtaccagcagaaa
V3D-20_03       ccctgtctttgtctccaggggaaagagccaccctctcctgcagggccagtcagagtgttagcagcagctacttagcctggtaccagcagaaa
IGKV3D-20*01    ccctgtctttgtctccaggggaaagagccaccctctcctgcgggccagtcagagtgttagcagcagctacttagcctggtaccagcagaaa
IGKV3D-20*02    ccctgtctttgtctccaggggaaagagccaccctctcctgcagggccagtcagagtgttagcagcagctacttagcctggtaccagcagaaa
V_V3D-20*01     ccctgtctttgtctccaggggaaagagccaccctctcctgcgggccagtcagagtgttagcagcagctacttagcctggtaccagcagaaa

V3D-20_01       cctggcctggcgcccaggctcctcatctatgatgcatccagcagggccactggcatcccagacaggttcagtggcagtgggtctgggacaga
V3D-20_02       cctggcctggcgcccaggctcctcatctatgatgcatccagcagggccactggcatcccagacaggttcagtggcagtgggtctgggacaga
V3D-20_03       cctggcctggcgcccaggctcctcatctatgatgcatccagcagggccactggcatcccagacaggttcagtggcagtgggtctgggacaga
IGKV3D-20*01    cctggcctggcgcccaggctcctcatctatgatgcatccagcagggccactggcatcccagacaggttcagtggcagtgggtctgggacaga
IGKV3D-20*02    cctggccaggctcccaggctcctcatctatgatgcatccagcagggccactggcatcccagacaggttcagtggcagtgggtctgggacaga
V_V3D-20*01     cctggcctggcgcccaggctcctcatctatgatgcatccagcagggccactggcatcccagacaggttcagtggcagtgggtctgggacaga

V3D-20_01       cttcactctcaccatcagcagactggagcctgaagattttgcagtgtattactgtcagcagtatggtagctcacct
V3D-20_02       cttcactctcaccatcagcagactggagcctgaagattttgcagtgtattactgtcagcagtatggtagctcacct
V3D-20_03       cttcactctcaccatcagcagactggagcctgaagattttgcagtgtattactgtcagcagtatggtagctcacct
IGKV3D-20*01    cttcactctcaccatcagcagactggagcctgaagattttgcagtgtattactgtcagcagtatggtagctcacct
IGKV3D-20*02    cttcactctcaccatcagcagactggagcctgaagattttgcagtctattactgtcagcagcgtagccaactggcat
V_V3D-20*01     cttcactctcaccatcagcagactggagcctgaagattttgcagtgtattactgtcagcagtatggtagctca---
```

IGKV4-1

```
V4-1_01        atggtgttgcagacccaggtcttcatttctctgttgctctggatctctggtgaggaattaaaaagtgccacagtcttttcagagtaatatc
V4-1_02        atggtgttgcagacccaggtcttcatttctctgttgctctggatctctggtgaggaattaaaaagtgccacagtcttttcagagtaatatc
IGKV4-1*01     atggtgttgcagacccaggtcttcatttctctgttgctctggatctct-------------------------------------------
V_V4-1*01      -------------------------------------------------------------------------------------------

V4-1_01        tgtgtagaaataaaaaaattaagatatagttagaaataatgactatttccaatatggatccaattatctgctgacttataatactact
V4-1_02        tgtgtagaaataaaaaaattaagatatagttggaaataatgactatttccaatatggatccaattatctgctgacttataatactact
IGKV4-1*01     ----------------------------------------------------------------------------------------
V_V4-1*01      ----------------------------------------------------------------------------------------

V4-1_01        agaaagcaaatttaaatgacatatttcaattatatctgagacagcgtgtataagtttatgtataatcattgtccattactgactacaggt
V4-1_02        agaaagcaaatttaaatgacatatttcaattatatctgagacagcgtgtataagtttatgtataatcattgtccattactgactacaggt
IGKV4-1*01     ---------------------------------------------------------------------------------------ggt
V_V4-1*01      ------------------------------------------------------------------------------------------

V4-1_01        gcctacggggacatcgtgatgacccagtctccagactccctggctgtgtctctgggcgagagggccaccatcaactgcaagtccagccag
V4-1_02        gcctacggggacatcgtgatgacccagtctccagactccctggctgtgtctctgggcgagagggccaccatcaactgcaagtccagccag
IGKV4-1*01     gcctacggggacatcgtgatgacccagtctccagactccctggctgtgtctctgggcgagagggccaccatcaactgcaagtccagccag
V_V4-1*01      ---------gacatcgtgatgacccagtctccagactccctggctgtgtctctgggcgagagggccaccatcaactgcaagtccagccag

V4-1_01        agtgttttatacagctccaacaataagaactacttagcttggtaccagcagaaaccaggacagcctcctaagctgctcatttactgggc
V4-1_02        agtgttttatacagctccaacaataagaactacttagcttggtaccagcagaaaccaggacagcctcctaagctgctcatttactgggc
IGKV4-1*01     agtgttttatacagctccaacaataagaactacttagcttggtaccagcagaaaccaggacagcctcctaagctgctcatttactgggc
V_V4-1*01      agtgttttatacagctccaacaataagaactacttagcttggtaccagcagaaaccaggacagcctcctaagctgctcatttactgggc

V4-1_01        atctacccgggaatccggggtccctgaccgattcagtggcagcgggtctgggacagatttcactctcaccatcagcagcctgcaggctgaa
V4-1_02        atctacccgggaatccggggtccctgaccgattcagtggcagcgggtctgggacagatttcactctcaccatcagcagcctgcaggctgaa
IGKV4-1*01     atctacccgggaatccggggtccctgaccgattcagtggcagcgggtctgggacagatttcactctcaccatcagcagcctgcaggctgaa
V_V4-1*01      atctacccgggaatccggggtccctgaccgattcagtggcagcgggtctgggacagatttcactctcaccatcagcagcctgcaggctgaa

V4-1_01        gatgtggcagtttattactgtcagcaatattatagtactcct
V4-1_02        gatgtggcagtttattactgtcagcagtattatagtactcct
IGKV4-1*01     gatgtggcagtttattactgtcagcaatattatagtactcct
V_V4-1*01      gatgtggcagtttattactgtcagcaatattatagtact---
```

IGKV5-2

```
V5-2_01       atggggtcccaggttcacctcctcagcttcctcctcctttggatctctgataccagggcagaaacgacactcacgcagtctccagcattcat
V5-2_02       atggggtcccaggttcacctcctcagcttcctcctcctttggatctctgataccagggcagaaacgacactcacgcagtctccagcattcat
V5-2_03       atggggtcccaggttcacctcctcagcttcctcctcctttggatctctgataccagggcagaaacgacactcacgcagtctccagcattcat
IGKV5-2*01    atggggtcccaggttcacctcctcagcttcctcctcctttggatctctgataccagggcagaaacgacactcacgcagtctccagcattcat
IGKV5-2*01    atggggtcccaggttcacctcctcagcttcctcctcctttggatctctgataccagggcagaaacgacactcacgcagtctccagcattcat
V_V5-2*01     -------------------------------------------------------------gaaacgacactcacgcagtctccagcattcat

V5-2_01       gtcagcgactccaggagacaaagtcaacatctcctgcaaagccagccaagacattgatgatgatatgaactggtaccaacagaaacca
V5-2_02       gtcagcgactccaggagacaaagtcaacatctcctgcaaagccagccaagacattgatgatgatatgaactggtaccaacagaaacca
V5-2_03       gtcagcgactccaggagacaaagtcaacatctcctgcaaagccagccaagacattgaggatgatatgaactggtaccaacagaaacca
IGKV5-2*01    gtcagcgactccaggagacaaagtcaacatctcctgcaaagccagccaagacattgatgatgatatgaactggtaccaacagaaacca
IGKV5-2*01    gtcagcgactccaggagacaaagtcaacatctcctgcaaagccagccaagacattgatgatgatatgaactggtaccaacagaaacca
V_V5-2*01     gtcagcgactccaggagacaaagtcaacatctcctgcaaagccagccaagacattgatgatgatatgaactggtaccaacagaaacca

V5-2_01       ggagaagctgctattttcattattcaagaagctactactctcgttcctggaatccacctcgattcagtggcagcgggtatggaacagattt
V5-2_02       ggagaagctgctattttcattattcaagaagctactactctcgttcctggaatctcacctcgattcagtggcagcgggtatggaacagattt
V5-2_03       ggagaagctgctattttcattattcaagaagctactactctcgttcctggaatctcacctcgattcagtggcagcgggtatggaacagattt
IGKV5-2*01    ggagaagctgctattttcattattcaagaagctactactctcgttcctggaatccacctcgattcagtggcagcgggtatggaacagattt
IGKV5-2*01    ggagaagctgctattttcattattcaagaagctactactctcgttcctggaatccacctcgattcagtggcagcgggtatggaacagattt
V_V5-2*01     ggagaagctgctattttcattattcaagaagctactactctcgttcctggaatccacctcgattcagtggcagcgggtatggaacagattt

V5-2_01       taccctcacaattaataacatagaatctgaggatgctgcatattacttctgtctacaacatgataatttccct
V5-2_02       taccctcacaattaataacatagaatctgaggatgctgcatattacttctgtctacaacatgataatttccct
V5-2_03       taccctcacaattaataacatagaatctgaggatgctgcatattacttctgtctacaacatgataatttccct
IGKV5-2*01    taccctcacaattaataacatagaatctgaggatgctgcatattacttctgtctacaacatgataatttccct
IGKV5-2*01    taccctcacaattaataacatagaatctgaggatgctgcatattacttctgtctacaacatgataatttccct
V_V5-2*01     taccctcacaattaataacatagaatctgaggatgctgcatattacttctgtctacaacatgataatttc---
```

IGKV6-21

```
V6-21_01       atgttgccatcacaactcattgggtttctgctgctctgggttccagcctccaggggtgaaattgtgctgactcagtctccagactttca
IGKV6-21*01    atgttgccatcacaactcattgggtttctgctgctctgggttccagcctccaggggtgaaattgtgctgactcagtctccagactttca
IGKV6-21*02    atgttgccatcacaactcattgggtttctgctgctctgggttccagcctccaggggtgaaattgtgctgactcagtctccagactttca

V6-21_01       gtctgtgactccaaaggagaaagtcaccatcacctgccgggccagtcagagcattggtagtagcttacactggtaccagcagaaaccagat
IGKV6-21*01    gtctgtgactccaaaggagaaagtcaccatcacctgccgggccagtcagagcattggtagtagcttacactggtaccagcagaaaccagat
IGKV6-21*02    gtctgtgactccaaaggagaaagtcaccatcacctgccgggccagtcagagcattggtagtagcttacactggtaccagcagaaaccagat

V6-21_01       cagtctccaaagctcctcatcaagtatgcttcccagtccttctcaggggtcccctcgaggttcagtggcagtggatctgggacagattt
IGKV6-21*01    cagtctccaaagctcctcatcaagtatgcttcccagtccttctcaggggtcccctcgaggttcagtggcagtggatctgggacagattt
IGKV6-21*02    cagtctccaaagctcctcatcaagtatgcttcccagtccatctcaggggtcccctcgaggttcagtggcagtggatctgggacagattt

V6-21_01       caccctcaccatcaatagcctggaagctgaagatgctgcaacgtattactgtcatcagagtagtagtttacct
IGKV6-21*01    caccctcaccatcaatagcctggaagctgaagatgctgcaacgtattactgtcatcagagtagtagtttacct
IGKV6-21*02    caccctcaccatcaatagcctggaagctgaagatgctgcaacgtattactgtcatcagagtagtagtttacct
```

IGKV6D-21

```
V6D-21_01       atgtcgccatcacaactcattgggtttctgctgctctgggttccagcctccaggggtgaaattgtgctgactcagtctccagactttcagtc
V6D-21_02       atgtcgccatcacaactcattgggtttctgctgctctgggttccagcctccaggggtgaaattgtgctgactcagtctccagactttcagtc
V6D-21_03       atgtcgccatcacaactcattgggtttctgctgctctgggttccagcctccaggggtgaaattgtgctgactcagtctccagactttcagtc
V6D-21_04       atgtcgccatcacaactcattgggtttctgctgctctgggttccagcctccaggggtgaaattgtgctgactcagtctccagactttcagtc
IGKV6D-21*01    atgttgccatcacaactcattgggtttctgctgctctgggttccagcctccaggggtgaaattgtgctgactcagtctccagactttcagtc
IGKV6D-21*02    atgtcgccatcacaactcattgggtttctgctgctctgggttccagcctccaggggtgaaattgtgctgactcagtctccagactttcagtc
V_V6D-21*01     ---------------------------------------------------------gaaattgtgctgactcagtctccagactttcagtc

V6D-21_01       tgtgactccaaaggagaaagtcaccatcacctgccgggccagtcagagcattggtagtagcttacactggtaccagcagaaaccagat
V6D-21_02       tgtgactccaaaggagaaagtcaccatcacctgccgggccagtcagagcattggtagtagcttacactggtaccagcagaaaccagat
V6D-21_03       tgtgactctaaaggagaaagtcaccatcacctgccgggccagtcagagcattggtagtagcttacactggtaccagcagaaaccagat
V6D-21_04       tgtgactccaaaggagaaagtcaccatcacctgccgggccagtcagagcattggtagtagcttacactggtaccagcagaaaccagat
IGKV6D-21*01    tgtgactccaaaggagaaagtcaccatcacctgccgggccagtcagagcattggtagtagcttacactggtaccagcagaaaccagat
IGKV6D-21*02    tgtgactccaaaggagaaagtcaccatcacctgccgggccagtcagagcattggtagtagcttacactggtaccagcagaaaccagat
V_V6D-21*01     tgtgactccaaaggagaaagtcaccatcacctgccgggccagtcagagcattggtagtagcttacactggtaccagcagaaaccagat

V6D-21_01       cagtctccaaagctcctcatcaagtatgcttcccagtccatctcaggggtcccctcgaggttcagtggcagtggatctgggacagatttcac
V6D-21_02       cagtctccaaagctcctcatcaagtatgcttcccagtccatctcaggggtcccctcgaggttcagtggcagtggatctgggacagatttcac
V6D-21_03       cagtctccaaagctcctcatcaagtatgcttcccagtccatctcaggggtcccctcgaggttcagtggcagtggatctgggacagatttcac
V6D-21_04       cagtctccaaagctcctcatcaagtatgcttcccagtccatctcaggggtcccctcgaggttcagtggcagtggatctgggacagatttcac
IGKV6D-21*01    cagtctccaaagctcctcatcaagtatgcttcccagtcctctctcaggggtcccctcgaggttcagtggcagtggatctgggacagatttcac
IGKV6D-21*02    cagtctccaaagctcctcatcaagtatgcttcccagtccatctcaggggtcccctcgaggttcagtggcagtggatctgggacagatttcac
V_V6D-21*01     cagtctccaaagctcctcatcaagtatgcttcccagtcctctctcaggggtcccctcgaggttcagtggcagtggatctgggacagatttcac

V6D-21_01       cctcaccatcaatagcctggaagctgaagatgctgcagcgtattactgtcatcagagtagtagtttacct
V6D-21_02       cctcaccatcaatagcctggaagctgaagatactgcagcgtattactgtcatcagagtagtagtttacct
V6D-21_03       cctcaccatcaatagcctggaagctgaagatgctgcagcgtattactgtcatcagagtagtagtttacct
V6D-21_04       cctcaccatcaatagcctggaagctgaagatgctgccgcgtattactgtcatcagagtagtagtttacct
IGKV6D-21*01    cctcaccatcaatagcctggaagctgaagatgctgcaacgtattactgtcatcagagtagtagtttacct
IGKV6D-21*02    cctcaccatcaatagcctggaagctgaagatgctgcagcgtattactgtcatcagagtagtagtttacct
V_V6D-21*01     cctcaccatcaatagcctggaagctgaagatgctgcaacgtattactgtcatcagagtagtagttta---
```

IGKV6D-41

```
V6D-41_01       atggtgtccccgttgcaattcctgcggcttctgctcctctgggttccagcctccaggggtgatgttgtgatgacacagtctccagctttc
V6D-41_02       atggtgtccccgttgcaattcctgcggcttctgctcctctgggttccagcctccaggggtgatgttgtgatgacacagtctccagctttc
V6D-41_03       atggtgtccccgttgcaattcctgcggcttctgctcctctgggttccagcctccaggggtgatgttgtgatgacacagtctccagctttc
IGKV6D-41*01    atggtgtccccgttgcaattcctgcggcttctgctcctctgggttccagcctccaggggtgatgttgtgatgacacagtctccagctttc
V_V6D-41*01     ------------------------------------------------------------gatgttgtgatgacacagtctccagctttc
                                                                            ******************************

V6D-41_01       ctctctgtgactccaggggagaaagtcaccatcacctgccaggccagtgaaggcattggcaactacttatactggtaccagcagaaacca
V6D-41_02       ctctctgtgactccaggggagaaagtcaccatcacctgccaggccagtgaaggcattggcaactacttatactggtaccagcagaaacca
V6D-41_03       ctctctgtgactccaggggagaaagtcaccatcacctgccaggccagtgaaggcattggcaactacttatactggtaccagcagaaacca
IGKV6D-41*01    ctctctgtgactccaggggagaaagtcaccatcacctgccaggccagtgaaggcattggcaactacttatactggtaccagcagaaacca
V_V6D-41*01     ctctctgtgactccaggggagaaagtcaccatcacctgccaggccagtgaaggcattggcaactacttatactggtaccagcagaaacca
                ******************************************************************************************

V6D-41_01       gatcaagccccaaagctcctc-atcaagtatgcttcccagtccatctcaggggtcccctcgaggttcagtggcagtggatctgggacaga
V6D-41_02       gatcaagccccaaagctcctc-atcaagtatgcttcccagtccatctcaggggtcccctcgaggttcagtggcagtggatctgggacaga
V6D-41_03       gatcaagccccaaagctcctcaatcaagtatgcttcccagtccatctcaggggtcccctcgaggttcagtggcagtggatctgggacaga
IGKV6D-41*01    gatcaagccccaaagctcctc-atcaagtatgcttcccagtccatctcaggggtcccctcgaggttcagtggcagtggatctgggacaga
V_V6D-41*01     gatcaagccccaaagctcctc-atcaagtatgcttcccagtccatctcaggggtcccctcgaggttcagtggcagtggatctgggacaga
                ********************* ********************************************************************

V6D-41_01       tttcacctttaccatcagtagcctggaagctgaagatgctgcaacatattactgtcagcagggcaataagcaccct
V6D-41_02       tttcacctttaccatcagtagcctggaagctgaagatgctgcaacttattactgtcagcagggcaataagcaccct
V6D-41_03       tttcacctttaccatcagtagcctggaagctgaagatgctgcaacatattactgtcagcagggcaataagcaccct
IGKV6D-41*01    tttcacctttaccatcagtagcctggaagctgaagatgctgcaacatattactgtcagcagggcaataagcaccct
V_V6D-41*01     tttcacctttaccatcagtagcctggaagctgaagatgctgcaacatattactgtcagcagggcaataagcac---
                ********************************************* ***************************
```

IGLV1-36

```
V1-36_01      atggcctggtcccctctcttcctcaccctcatcactcactgtgcagggtcctgggcccagtctgtgctgactcagccaccctcggtgtct
V1-36_02      atggcctggtcccctctcttcctcaccctcatcactcactgtgcagggtcctgggcccagtctgtgctgactcagccaccctcggtgtct
V1-36_03      atggcctggtcccctctcttcctcaccctcatcactcactgtgcagggtcctgggcccagtctgtgctgactcagccaccctcggtgtct
V1-36_04      atggcctggtcccctctcttcctcaccctcatcactcactgtgcagggtcctgggcccagtctgtgctgactcagccaccctcggtgtct
V1-36_05      atggcctggtcccctctcttcctcaccctcatcactcactgtgcagggtcctgggcccagtctgtgctgactcagccaccctcggtgtct
V1-36_06      atggcctggtcccctctcttcctcaccctcatcactcactgtgcagggtcctgggcccagtctgtgctgactcagccaccctcggtgtct
V1-36_07      atggcctggtcccctctcttcctcaccctcatcactcactgtgcagggtcctgggcccagtctgtgctgactcagccaccctcggtgtct
IGLV1-36*01   ----------------------------------------------------------cagtctgtgctgactcagccaccctcggtgtct
V_V1-36*01    ----------------------------------------------------------cagtctgtgctgactcagccaccctcggtgtct

V1-36_01      gaagcccccaggcagagggtcaccatctcctgttctggaagcagctccaacatcggaaataatgctgtaaactggtaccagcagctccca
V1-36_02      gaagcccccaggcagagggtcaccatctcctgttctggaagcagctccaacatcggaaataatgctgtaaactggtaccagcagctccca
V1-36_03      gaagcccccaggcagagggtcaccatctcctgttctggaagcagctccaacatcggaaataatactgtaaactggtaccagcagctccca
V1-36_04      gaagcccccaggcagagggtcaccatctcctgttctggaagcagctccaacatcggaaataatgctgtaaactggtaccagcagctccca
V1-36_05      gaagcccccaggcagagggtcaccatctcctgttctggaagcagctccaacatcggaaataatgctgtaaactggtaccagcagctccca
V1-36_06      gaagcccccaggcagagggtcaccatctcctgttctggaagcagctccaacatcggaaataatgctgtaaactggtaccagcagctccca
V1-36_07      gaagcccccaggcagagggtcaccatctcctgttctggaagcagctccaacatcggaaataatgctgtaaactggtaccagcagctccca
IGLV1-36*01   gaagcccccaggcagagggtcaccatctcctgttctggaagcagctccaacatcggaaataatgctgtaaactggtaccagcagctccca
V_V1-36*01    gaagcccccaggcagagggtcaccatctcctgttctggaagcagctccaacatcggaaataatgctgtaaactggtaccagcagctccca

V1-36_01      ggaaaggctcccaaactcctcatctattatgatgatctgctgccctcaggggtctctgaccgattctctggctccaagtctggcacctcagc
V1-36_02      ggaaaggctcccaaactcctcatctattatgatgatctgctgccctcaggggtctctgaccgattctctggctccaagtctggcacctcagc
V1-36_03      ggaaaggctcccaaactcctcatctattatgatgatctgctgccctcaggggtctctgaccgattctctggctccaagtctggcacctcagc
V1-36_04      ggaaaggctcccaaactcctcatctattatgatgatctgctgccctcaggggtctctgaccgattctctggctccaagtctggcacctcagc
V1-36_05      ggaaaggctcccaaactcctcatctattatgatgatctgctgctctcaggggtctctgaccgattctctggctccaagtctggcacctcagc
V1-36_06      ggaaaggctcccaaactcctcatctattatgatgatctgctgccctcaggggtctctgaccgattctctggctccaagtctggcacctcagc
V1-36_07      ggaaaggctcccaaactcctcatctattatgatgatctgctgacctcaggggtctctgaccgattctctggctccaagcctggcacctcagc
IGLV1-36*01   ggaaaggctcccaaactcctcatctattatgatgatctgctgccctcaggggtctctgaccgattctctggctccaagtctggcacctcagc
V_V1-36*01    ggaaaggctcccaaactcctcatctattatgatgatctgctgccctcaggggtctctgaccgattctctggctccaagtctggcacctcagc

V1-36_01      ctccctggccatcagtgggctccagtctgaggaggatgaggctgattattactgtgcagcatgggatgacagcctgaatggtc
V1-36_02      ctccctggccatcagtgggctccagtctgaggaggatgaggctgattattactgtgcagcatgggatgacaggctgaatggtc
V1-36_03      ctccctggccatcagtgggctccagtctgaggaggatgaggctgattattactgtgcagcatgggatgacagcctgaatggtc
V1-36_04      ctccctggccatcagtgggctccagtctgaggaggatgaggctgattattactgtgcagcatgggatgacagcctgagtggtc
V1-36_05      ctccctggccatcagtgggctccagtctgaggaggatgaggctgattattactgtgcagcatgggatgacagcctgaatggtc
V1-36_06      ctccctggccatcagtgggctccagtct---gaggatgaggctgattattactgtgcagcatgggatgacagcctgaatggtc
V1-36_07      ctccctggccatcagtgggctccagtctgaggaggatgaggctgattattactgtgcagcatgggatgacagcctgaatggtc
IGLV1-36*01   ctccctggccatcagtgggctccagtct---gaggatgaggctgattattactgtgcagcatgggatgacagcctgaatggtc
V_V1-36*01    ctccctggccatcagtgggctccagtct---gaggatgaggctgattattactgtgcagcatgggatgacagcctg-------
```

IGLV1-40

```
V1-40_01      atggcctggtctcctctcctcctcactctcctcgctcactgcacagggtcctgggcccagtctgtgctgacgcagccgccctcagtgtctgg
V1-40_02      atggcctggtctcctctcctcctcactctcctcgctcactgcacagggtcctgggcccagtctgtgctgacgcagccgccctcagtgtctgg
V1-40_03      atggcctggtctcctctcctcctcactctcctcgctcactgcacagggtcctgggcccagtctgtgctgacgcagccgccctcagtgtctgg
V1-40_04      atggcctggtctcctctcctcctcactctcctcgctcactgcacagggtcctgggcccagtctgtgctgacgcagccgccctcagtgtctgg
V1-40_05      atggcctggtctcctctcctcctcactctcctcgcttactgcacagggtcctgggcccagtctgtgctgacgcagccgccctcagtgtctgg
V1-40_06      atggcctggtctcctctcctcctcactctcctcgctcactgcacagggtcctgggcccagtctgtgctgacgcagccgccctcagtgtctgg
IGLV1-40*01   atggcctggtctcctctcctcctcactctcctcgctcactgcacagggtcctgggcccagtctgtgctgacgcagccgccctcagtgtctgg
IGLV1-40*02   atggcctggtctcctctcctcctcactctcctcgctcactgcacagggtcctgggcccagtctgtcgtgacgcagccgccctcagtgtctgg
IGLV1-40*03   ----------------------------------------------------------cagtctgtcgtgacgcagccgccctcagtgtctgg
V_V1-40*01    ----------------------------------------------------------cagtctgtgctgacgcagccgccctcagtgtctgg
V_V1-40*02    ----------------------------------------------------------cagtctgtcgtgacgcagccgccctcagtgtctgg
```

```
V1-40_01      ggccccagggcagagggtcaccatctcctgcactgggagcagctccaacatcggggcaggttatgatgtacactggtaccagcagctt
V1-40_02      ggccccagggcagagggtcaccatctcctgcactgggagcagctccaacattggggcaggttatgatgtacactggtaccagcagctt
V1-40_03      ggccccagggcagagggtcaccatctcctgcactgggagcagctccaacatcggggcaggttatgatgtacactggtaccagcagctt
V1-40_04      ggccccagggcagagggtcaccatctcctgcactgggagcagctccaacatcggggcaggttatgatgtacactggtaccagcagctt
V1-40_05      ggccccagggcagagggtcaccatctcctgcactgggagcagctccaacatcggggcaggttatgatgtacactggtaccagcagctt
V1-40_06      ggccccagggcagagggtcaccatctcctgcactgggagcagctccaacatcggggcaggttatgatgtacactggtaccagcagctt
IGLV1-40*01   ggccccagggcagagggtcaccatctcctgcactgggagcagctccaacatcggggcaggttatgatgtacactggtaccagcagctt
IGLV1-40*02   ggccccagggcagagggtcaccatctcctgcactgggagcagctccaacatcggggcaggttatgatgtacactggtaccagcagctt
IGLV1-40*03   ggccccagggcagagggtcaccatctcctgcactgggagcagctccaacatcggggcaggttatgatgtacactggtaccagcagctt
V_V1-40*01    ggccccagggcagagggtcaccatctcctgcactgggagcagctccaacatcggggcaggttatgatgtacactggtaccagcagctt
V_V1-40*02    ggccccagggcagagggtcaccatctcctgcactgggagcagctccaacatcggggcaggttatgatgtacactggtaccagcagctt
```

```
V1-40_01      ccaggaacagcccccaaactcctcatctatggtaacagcaatcggccctcagggtccctgaccgattctctggctccaagtctggcac
V1-40_02      ccaggaacagcccccaaactcctcatctatggtaacagcaatcggccctcagggtccctgaccgattctctggctccaagtctggcac
V1-40_03      ccaggaacagcccccaaactcctcatctatggtaacagcaatcggccctcagggtccctgaccgattctctggctccaagtctggcac
V1-40_04      ccaggaacagcccccaaactcctcatctatggtaacagcaatcggccctcagcggtccctgaccgattctctggctccaagtctggcac
V1-40_05      ccaggaacagcccccaaactcctcatctatggtaacagcaatcggccctcagggtccctgaccgattctctggctccaagtctggcac
V1-40_06      ccaggaacagcccccaaactcctcatctatggtaacagcaatcggcccgcagggtccctgaccgattctctggctccaagtctggcac
IGLV1-40*01   ccaggaacagcccccaaactcctcatctatggtaacagcaatcggccctcagggtccctgaccgattctctggctccaagtctggcac
IGLV1-40*02   ccaggaacagcccccaaactcctcatctatggtaacagcaatcggccctcagggtccctgaccgattctctggctccaagtctggcac
IGLV1-40*03   ccaggaacagcccccaaactcctcatctatggtaacagcaatcggccctcagggtccctgaccgattctctggctccaagtctggcgc
V_V1-40*01    ccaggaacagcccccaaactcctcatctatggtaacagcaatcggccctcagggtccctgaccgattctctggctccaagtctggcac
V_V1-40*02    ccaggaacagcccccaaactcctcatctatggtaacagcaatcggccctcagggtccctgaccgattctctggctccaagtctggcac
```

V1-40_01	ctcagcctccctggccatcactgggctccaggctgaggatgaggctgattattactgccagtcctatgacagcagcctgagtggttc
V1-40_02	ctcagcctccctggccatcactgggctccaggctgaggatgaggctgattattactgccagtcctatgacagcagcctgagtggttc
V1-40_03	ctcagcctccctggccatcagtgggctccaggctgaggatgaggctgattattactgccagtcctatgacagcagcctgagtggttc
V1-40_04	ctcagcctccctggccatcactgggctccaggctgaggatgaggctgattattactgccagtcctatgacagcagcctgagtggttc
V1-40_05	ctcagcctccctggccatcactgggctccaggctgaggatgaggctgattattactgccagtcctatgacagcagcctgagtggttc
V1-40_06	ctcagcctccctggccatcactgggctccaggctgaggatgaggctgattattactgccagtcctatgacagcagcctgagtggttc
IGLV1-40*01	ctcagcctccctggccatcactgggctccaggctgaggatgaggctgattattactgccagtcctatgacagcagcctgagtggttc
IGLV1-40*02	ctcagcctccctggccatcactgggctccaggctgaggatgaggctgattattactgccagtcctatgacagcagcctgagtggttc
IGLV1-40*03	ctcagcctccctggccatcactgggctccaggctgaggatgaggctgattattactgccagtcctatgacagcagcctgagtggt--
V_V1-40*01	ctcagcctccctggccatcactgggctccaggctgaggatgaggctgattattactgccagtcctatgacagcagcctg--------
V_V1-40*02	ctcagcctccctggccatcactgggctccaggctgaggatgaggctgattattactgccagtcctatgacagcagcctg--------

IGLV1-44

```
V1-44_01      atggccagcttccctctcctcctcaccctcctcactcactgtgcagggtcctgggcccagtctgtgctgactcagccaccctcagcgtctggg
V1-44_02      atggccagcttccctctcctcctcaccctcctcactcactgtgcagggtcctgggcccagtctgtgctgactcagccaccctcagcgtctggg
V1-44_03      atggccagcttccctctcctcctcaccctcctcactcactgtgcagggtcctgggcccagtctgtgctgactcagccaccctcagcgtctggg
V1-44_04      atggccagcttccctctcctcctcaccctcctcactcactgtgcagggtcctgggcccagtctgtgctgactcagccaccctcagcgtctggg
V1-44_05      atggccagcttccctctcctcctcaccctcctcactcactgtgcagggtcctgggcccagtctgtgctgactcagccaccctcagcgtctggg
V1-44_06      atggccagcttccctctcctcctcaccctcctcactcactgtgcagggtcctgggcccagtctgtgctgactcagccaccctcagcgtctggg
V1-44_07      atggccagcttccctctcctcctcaccctcctcactcactgtgcagggtcctgggcccagtctgtgctgactcagccaccctcagcgtctggg
IGLV1-44*01   ---------------------------------------------------------cagtctgtgctgactcagccaccctcagcgtctggg
V_V1-44*01    ---------------------------------------------------------cagtctgtgctgactcagccaccctcagcgtctggg

V1-44_01      acccccgggcagagggtcaccatctcttgttctggaagcagctccaacatcggaagtaatactgtaaactggtaccagcagctccca
V1-44_02      acccccgggcagagggtcaccatctcttgttctggaagcagctccaacatcggaagtaatactgtaaactggtaccagcagctccca
V1-44_03      acccccgggcagagggtcaccatctcttgttctggaagcagctccaacatcggaagtaatactgtaaactggtaccagcagctccca
V1-44_04      acccccgggcagagggtcaccatctcttgttctggaagcagctccaacatcggaagtaatactgtaaactggtaccagcagctccca
V1-44_05      acccccgggcagagggtcaccatctcttgttctggaagcagctccaacatcggaagtaatactgtaaactggtaccagcagctccca
V1-44_06      acccccgggcagagggtcaccatctcttgttctggaagcagctccaacatcggaagtaatactgtaaactggtaccagcagctccca
V1-44_07      acccccgggcagagggtcaccatctcttgttctggaagcagctccaacatcggaagtaatactgtaaactggtaccagcagctccca
IGLV1-44*01   acccccgggcagagggtcaccatctcttgttctggaagcagctccaacatcggaagtaatactgtaaactggtaccagcagctccca
V_V1-44*01    acccccgggcagagggtcaccatctcttgttctggaagcagctccaacatcggaagtaatactgtaaactggtaccagcagctccca

V1-44_01      ggaacggccccaaactcctcatctatagtaataatcagcggccctcaggggtccctgaccgattctctggctccaagtctggcacctcagcc
V1-44_02      ggaacggccccaaactcctcatctataggaataatcagcggccctcaggggtccctgaccgattctctggctccaagtctggcacctcagcc
V1-44_03      ggaacggccccaaactcctcatctatagtaataatcagcggccctcaggggtccctgaccgattctctggctccaagtctggcacctcagcc
V1-44_04      ggaacggccccaaactcctcatctataggaataatcagcggccctcaggggtccctgaccgattctctggctccaagtctggcacctcagcc
V1-44_05      ggaacggccccaaactcctcatctataggaataatcagcggccctcaggggtccctgaccgattctctggctccaagtctggcacctcagcc
V1-44_06      ggaacggccccaaactcctcatctatagtaataatcagcggccctcaggggtccctgaccgattctctggctccaagtctggcacctcagcc
V1-44_07      ggaacgggcccaaactcctcatctatagtaataatcagcggccctcaggggtccctgaccgattctctggctccaagtctggcacctcagcc
IGLV1-44*01   ggaacggccccaaactcctcatctatagtaataatcagcggccctcaggggtccctgaccgattctctggctccaagtctggcacctcagcc
V_V1-44*01    ggaacggccccaaactcctcatctatagtaataatcagcggccctcaggggtccctgaccgattctctggctccaagtctggcacctcagcc

V1-44_01      tccctggccatcagtgggctccagtctgaggatgaggctgattattactgtgcagcatgggatgacagcctgaatggtc-
V1-44_02      tccctggccatcagtgggctccagtctgaggatgaggctgattattactgtgcagcatgggatgacagcctgaatggtc-
V1-44_03      tccctggccatcagtgggctccagtctgaggatgaggctgattattactgtgcagcatgggatgacagcctgagtggtc-
V1-44_04      tccctggccatcagtgggctccagtccgaggatgaggctgattattactgtgcagcatgggatgacagcctgaatggtc-
V1-44_05      tccctggccatcagtgggctccaggctgaggatgaggctgattattactgtgcagcatgggatgacagcctgaatggtc-
V1-44_06      tccctggccatcagtgggctccagtccgaggatgaggctgattattactgtgcagcatgggatgacagcctgaatggtc-
V1-44_07      tccctggccatcagtgggctccagtctgaggatgaggctgattattactgtgcagcatgggatgacagcctgaatggtc-
IGLV1-44*01   tccctggccatcagtgggctccagtctgaggatgaggctgattattactgtgcagcatgggatgacagcctgaatggtcc
V_V1-44*01    tccctggccatcagtgggctccagtctgaggatgaggctgattattactgtgcagcatgggatgacagcctg--------
```

IGLV1-47

```
V1-47_01        atggccggcttccctctcctcctcaccctcctcactcactgtgcagggtcctgggcccagtctgtgctgactcagccaccctcagcgtctggg
V1-47_02        atggccggcttccctctcctcctcaccctcctcactcactgtgcagggtcctgggcccagtctgtgctgactcagccaccctcagcgtctggg
V1-47_03        atggccggcttccctctcctcctcaccctcctcactcactgtgcagggtcctgggcccagtctgtgctgactcagccaccctcagcgtctggt
V1-47_04        atggccggcttccctctcctcctcaccctcctcactcactgtgcagggtcctgggcccagtctgtgctgactcagccaccctcagcgtctggg
V1-47_05        atggccggcttccctatcctcctcaccctcctcactcactgtgcagggtcctgggcccagtctgtgctgactcagccaccctcagcgtctggg
V1-47_06        atggccggcttccctctcctcctcaccctcctcactcactgtgcagggtcctgggcccagtctgtgctgactcagccaccctcagcgtctggg
V1-47_07        atggccggcttccctctcctcctcaccctcctcactcactgtgcagggtcctgggcccagtctgtgctgactcagccaccctcagcgtctggg
V1-47_08        atggccggcttccctctcctcctcaccctcctcactcactgtgcagggtcctgggcccagtctgtgctgactcagccaccctcagcgtctggg
IGLV1-47*02     atggccggcttccctctcctcctcaccctcctcactcact------ggtcctgggcccagtctgtgctgactcagccaccctcagcgtctggg
IGLV1-47*01     ----------------------------------------------------------cagtctgtgctgactcagccaccctcagcgtctggg
V_IGLV1-47*01   ----------------------------------------------------------cagtctgtgctgactcagccaccctcagcgtctggg
V_IGLV1-47*02   ----------------------------------------------------------cagtctgtgctgactcagccaccctcagcgtctggg

V1-47_01        acccccgggcagaggtcaccatctcttgttctggaagcagctccaacatcggaagtaattatgtatactggtaccagcagctccca
V1-47_02        acccccgggcagaggtcaccatctcttgttctggaagcagctccaacatcggaagtaattatgtatactggtaccagcagctccca
V1-47_03        acccccgggcagaggtcaccatctcttgttctggaagcagctccaacatcggaagtaattatgtatactggtaccagcagctccca
V1-47_04        acccccgggcagaggtcaccatctcttgttctggaagcagctccaacatcggaagtaattatgtatactggtaccagcagctccca
V1-47_05        acccccgggcagaggtcaccatctcttgttctggaagcagctccaacatcggaagtaattatgtatactggtaccagcagctccca
V1-47_06        acccccgggcagaggtcaccatctcttgttctggaagcagctccaacatcggaagtaattatgtatactggtaccagcagctccca
V1-47_07        acccccgggcagaggtcaccatctcttgttctggaagcagctccaacatcggaagtaattatgtatactggtaccagcagctccca
V1-47_08        acccccgggcagaggtcaccatctcttgttctggaagcagctccaacatcggaagtaagtatgtatactggtaccagcagctccca
IGLV1-47*02     acccccgggcagaggtcaccatctcttgttctggaagcagctccaacatcggaagtaattatgtatactggtaccagcagctccca
IGLV1-47*01     acccccgggcagaggtcaccatctcttgttctggaagcagctccaacatcggaagtaattatgtatactggtaccagcagctccca
V_IGLV1-47*01   acccccgggcagaggtcaccatctcttgttctggaagcagctccaacatcggaagtaattatgtatactggtaccagcagctccca
V_IGLV1-47*02   acccccgggcagaggtcaccatctcttgttctggaagcagctccaacatcggaagtaattatgtatactggtaccagcagctccca

V1-47_01        ggaacggcccccaaactcctcatctataggaataatcagcggccctcaggggtccctgaccgattctctggctccaagtctggcacctcagc
V1-47_02        ggaacggcccccaaactcctcatctatagtaataatcagcggccctcaggggtccctgaccgattctctggctccaagtctggcacctcagc
V1-47_03        ggaacggcccccaaactcctcatctataggaataatcagcggccctcaggggtccctgaccgattctctggctccaagtctggcacctcagc
V1-47_04        ggaacgacccccaaactcctcatctataggaataatcagcggccctcaggggtccctgaccgattctctggctccaagtctggcacctcagc
V1-47_05        ggaacggcccccaaactcctcatctataggaataatcagcggccctcaggggtccctgaccgattctctggctccaagtctggcacctcagc
V1-47_06        ggaacggcccccaaactcctcatctatagtaataatcagcggccctcaggggtccctgaccgattctctggctccaagtctggcacctcagc
V1-47_07        ggaacggcccccaaactcctcatctataggaataatcagcggccctcaggggtccctgaccgattctctggctccaagtctggcacctcagc
V1-47_08        ggaacggcccccaaactcctcatctataggaataatcagcggccctcaggggtccctgaccgattctctggctccaagtctggcacctcagc
IGLV1-47*02     ggaacggcccccaaactcctcatctatagtaataatcagcggccctcaggggtccctgaccgattctctggctccaagtctggcacctcagc
IGLV1-47*01     ggaacggcccccaaactcctcatctataggaataatcagcggccctcaggggtccctgaccgattctctggctccaagtctggcacctcagc
V_IGLV1-47*01   ggaacggcccccaaactcctcatctataggaataatcagcggccctcaggggtccctgaccgattctctggctccaagtctggcacctcagc
V_IGLV1-47*02   ggaacggcccccaaactcctcatctatagtaataatcagcggccctcaggggtccctgaccgattctctggctccaagtctggcacctcagc
```

```
V1-47_01         ctccctggccatcagtgggctccggtccgaggatgaggctgattattactgtgcagcatgggatgacagcctgagtggt
V1-47_02         ctccctggccatcagtgggctccggtccgaggatgaggctgattattactgtgcagcatgggatgacagcctgagtggt
V1-47_03         ctccctggccatcagtgggctccggtccgaggatgaggctgattattactgtgcagcatgggatgacagcctgagtggt
V1-47_04         ctccctggccatcagtgggctccggtccgaggatgaggctgattattactgtgcagcatgggatgacagcctgagtggt
V1-47_05         ctccctggccatcagtgggctccggtccgaggatgaggctgattattactgtgcagcatgggatgacagcctgagtggt
V1-47_06         ctccctggccatcagtgggctccggtccgaagatgaggctgattattactgtgcagcatgggatgacagcctgagtggt
V1-47_07         ctccctggccatcagtgggctctggtccgaggatgaggctgattattactgtgcagcatgggatgacagcctgagtggt
V1-47_08         ctccctggccatcagtgggctccggtccgaggatgaggctgattattactgtgcagcatgggatgacagcctgagtggt
IGLV1-47*02      ctccctggccatcagtgggctccggtccgaggatgaggctgattattactgtgcagcatgggatgacagcctgagtggt
IGLV1-47*01      ctccctggccatcagtgggctccggtccgaggatgaggctgattattactgtgcagcatgggatgacagcctgagtggt
V_IGLV1-47*01    ctccctggccatcagtgggctccggtccgaggatgaggctgattattactgtgcagcatgggatgacagcctg------
V_IGLV1-47*02    ctccctggccatcagtgggctccggtccgaggatgaggctgattattactgtgcagcatgggatgacagcctg------
```

IGLV1-50

```
V1-50_01       atggcctggtctcctctcctcctcactctcctcgctcactgcacagggtcctgggcccagtctgtgctgacgcagccgccctcagtgtctg
V1-50_02       atggcctggtcttctctcctcctcactctcctcgctcactgcacagggtcctgggcccagtctgtgctgacgcagccgccctcagtgtctg
V1-50_03       atggcctggtcttctctcctcctcactctcctcgctcactgcacagggtcctgggcccagtctgtgctgacgcagccgccctcagtgtctg
V1-50_04       atggcctggtcttctctcctcctcactctcctcgctcactgcacagggtcctgggcccagtctgtgctgacgcagccgccctcagtgtctg
V1-50_05       atggcctggtcttctctcctcctcactctcctcgctcactgcacagggtcctgggcccagtctgtgctgacgcagccgccctcagtgtctg
V1-50_06       atggcctggtctcctctcctcctcactctcctcgctcactgcacagggtcctgggcccagtctgtgctgacgcagccgccctcagtgtctg
IGLV1-50*01    atggcctggtctcctctcctcctcactctcctcgctcactgcacagggtcctgggcccagtctgtgctgacgcagccgccctcagtgtctg
V_V1-50*01     ---------------------------------------------------------cagtctgtgctgacgcagccgccctcagtgtctg

V1-50_01       gggccccagggcagagggtcaccatctcctgcactgggagcagctccaacattggggcgggttatgttgtacattggtaccagcagctt
V1-50_02       gggccccagggcagagggtcaccatctcctgcactgggagcagctccaacattggggcgggttatgttgtacattggtaccagcagctt
V1-50_03       gggccccagggcagagggtcaccatctcctgcactgggagcagctccaacatcggggcgggttatgttgtacattggtaccagcagctt
V1-50_04       gggccccagggcagagggtcaccatctcctgcactgggagcagctccaacattggggcgggttatgttgtacattggtaccagcagctt
V1-50_05       gggccccagggcagagggtcaccatctcctgcactgggagcagctccaacattggggcgggttatgttgtacattggtaccagcagctt
V1-50_06       gggccccagggcagagggtcaccatctcctgcactgggagcagctccaacattggggcgggttatgttgtacattggtaccagcagctt
IGLV1-50*01    gggccccagggcagagggtcaccatctcctgcactgggagcagctccaacattggggcgggttatgttgtacattggtaccagcagctt
V_V1-50*01     gggccccagggcagagggtcaccatctcctgcactgggagcagctccaacattggggcgggttatgttgtacattggtaccagcagctt

V1-50_01       ccaggaacagcccccaaactcctcatctatggtaacagcaatcggccctcaggggtccctgaccaattctctggctccaagtctggcacctc
V1-50_02       ccaggaacagcccccaaactcctcatctatggtaacagcaatcggccctcaggggtccctgaccaattctctggctccaagtctggcacctc
V1-50_03       ccaggaacagcccccaaactcctcatctatggtaacagcaatcggccctcaggggtccctgaccaattctctggctccaagtctggcacctc
V1-50_04       ccaggaacagcccccaaactcctcatctatggtaacagcaatcggccctcaggggtccctgaccgattctctggctccaagtctggcacctc
V1-50_05       ccaggaacagcccccaaactcctcatctatggtaacagcagtcggccctcaggggtccctgaccaattctctggctccaagtctggcacctc
V1-50_06       ccaggaacagcccccaaactcctcatctatggtaacagcaatcggccctcggggtccctgaccaattctctggctccaagtctggcacctc
IGLV1-50*01    ccaggaacagcccccaaactcctcatctatggtaacagcaatcggccctcaggggtccctgaccaattctctggctccaagtctggcacctc
V_V1-50*01     ccaggaacagcccccaaactcctcatctatggtaacagcaatcggccctcaggggtccctgaccaattctctggctccaagtctggcacctc

V1-50_01       agcctccctggccatcactggactccagtctgaggatgaggctgattattactgcaaagcatgggataacagcctgaatgct
V1-50_02       agcctccctggccatcactggactccagtctgaggatgaggctgattattactgcaaagcatgggataacagcctgaatgct
V1-50_03       agcctccctggccatcactggactccagtctgaggatgaggctgattattactgcaaagcatgggataacagcctgaatgct
V1-50_04       agcctccctggccatcactggactccagtctgaggatgaggctgattattactgcaaagcatgggataacagcctgaatgct
V1-50_05       agcctccctggccatcactggactccagtctgaggatgaggctgattattactgcaaagcatgggataacagcctgaatgct
V1-50_06       agcctccctggccatcactggactccagtctgaggatgaggctgattattactgcaaagcatgggataacagcctgaatgct
IGLV1-50*01    agcctccctggccatcactggactccagtctgaggatgaggctgattattactgcaaagcatgggataacagcctgaatgct
V_V1-50*01     agcctccctggccatcactggactccagtctgaggatgaggctgattattactgcaaagcatgggataacagcctg------
```

IGLV1-51

```
V1-51_01       atgacctgctcccctctcctcctcacccttctcattcactgcacagggtcctgggcccagtctgtgttgacgcagccgccctcagtgtctgcg
V1-51_02       atgacctgctcccctctcctcctcacccttctcattcactgcacagggtcctgggcccagtctgtgttgacgcagccgccctcagtgtctgcg
V1-51_03       atgacctgctcccctctcctcctcacccttctcattcactgcacagggtcctgggcccagtctgtgttgacgcagccgccctcagtgtctgcg
V1-51_04       atgacctgctcccctctcctcctcacccttctcattcactgcacagggtcctgggcccagtctgtgttgacgcagccgccctcagtgtctgcg
IGLV1-51*01    ---------------------------------------------------------cagtctgtgttgacgcagccgccctcagtgtctgcg
IGLV1-51*02    ---------------------------------------------------------cagtctgtgttgacgcagccgccctcagtgtctgcg
V_V1-51*01     ---------------------------------------------------------cagtctgtgttgacgcagccgccctcagtgtctgcg
V_V1-51*02     ---------------------------------------------------------cagtctgtgttgacgcagccgccctcagtgtctgcg

V1-51_01       gccccaggacagaaggtcaccatctcctgctctggaagcagctccaacattgggaataattatgtatcctggtaccagcagctccca
V1-51_02       gccccaggacagaaggtcaccatctcctgctctggaagcagctccaacattgggaataattatgtatcctggtaccagcagctccca
V1-51_03       gccccaggacagaaggtcaccatctcctgctctggaagcagctccaacattgggaataattatgtatcctggtaccagcagctccca
V1-51_04       gccccaggacagaaggtcaccatctcctgctctggaagcagctccaacattgggaataattatgtatcctggtaccagcagctccca
IGLV1-51*01    gccccaggacagaaggtcaccatctcctgctctggaagcagctccaacattgggaataattatgtatcctggtaccagcagctccca
IGLV1-51*02    gccccaggacagaaggtcaccatctcctgctctggaagcagctccaacattgggaataattatgtatcctggtaccagcagctccca
V_V1-51*01     gccccaggacagaaggtcaccatctcctgctctggaagcagctccaacattgggaataattatgtatcctggtaccagcagctccca
V_V1-51*02     gccccaggacagaaggtcaccatctcctgctctggaagcagctccaacattgggaataattatgtatcctggtaccagcagctccca

V1-51_01       ggaacagcccccaaactcctcatttatgacaataataagcgaccctcagggattcctgaccgattctctggctccaagtctggcacgtcag
V1-51_02       ggaacagcccccaaactcctcatctatgaaaataataagcgaccctcagggattcctgaccgattctctggctccaagtctggcacgtcag
V1-51_03       ggaacagcccccaaactcctcatttatgacaataataagcgaccctcagggattcctgaccgattctctggctccaagtctggcacgtcag
V1-51_04       ggaacagcccccaaactcctcatctatgaaaataataagcgaccctcagggattcctgaccgattctctggctccaagtctggcacgtcag
IGLV1-51*01    ggaacagcccccaaactcctcatttatgacaataataagcgaccctcagggattcctgaccgattctctggctccaagtctggcacgtcag
IGLV1-51*02    ggaacagcccccaaactcctcatctatgaaaataataagcgaccctcagggattcctgaccgattctctggctccaagtctggcacgtcag
V_V1-51*01     ggaacagcccccaaactcctcatttatgacaataataagcgaccctcagggattcctgaccgattctctggctccaagtctggcacgtcag
V_V1-51*02     ggaacagcccccaaactcctcatctatgaaaataataagcgaccctcagggattcctgaccgattctctggctccaagtctggcacgtcag

V1-51_01       ccaccctgggcatcaccggactccagactggggacgaggccgattattactgcggaacatgggatagcagcctgagtgctggcacagtgctcc
V1-51_02       ccaccctgggcatcaccggactccagactggggacgaggccgattattactgcggaacatgggatagcagcctgagtgctggcacagtgctcc
V1-51_03       ccaccctgggcatcaccggactccagactggggacgaggccgattattactgtggaacatgggatagcagcctgagtgctggcacagtgctcc
V1-51_04       ccaccctgggcatcaccggactccagactggggacgaggccgattattactgcggaacatgggatagcagcctgcgtgctggcacagtgctcc
IGLV1-51*01    ccaccctgggcatcaccggactccagactggggacgaggccgattattactgcggaacatgggatagcagcctgagtgctgg-----------
IGLV1-51*02    ccaccctgggcatcaccggactccagactggggacgaggccgattattactgcggaacatgggatagcagcctgagtgctgg-----------
V_V1-51*01     ccaccctgggcatcaccggactccagactggggacgaggccgattattactgcggaacatgggatagcagcctg-------------------
V_V1-51*02     ccaccctgggcatcaccggactccagactggggacgaggccgattattactgcggaacatgggatagcagcctg-------------------
```

IGLV2-8

```
V2-8_01         atggcctgggctctgctcctcctcaccctcctcactcagggcacagggtcctgggcccagtctgccctgactcagcctccctccgcgtccg
V2-8_02         atggcctgggctctgctcctcctcagcctcctcactcagggcacagggtcctgggcccagtctgccctgactcagcctccctccgcgtccg
V2-8_03         atggcctgggctctgctcatcctcaccctcctcactcagggcacagggtcctgggcccagtctgccctgactcagcctccctccgcgtccg
V2-8_04         atggcctgggctctgctcctcctcagcctcctcactcagggcacagggtcctgggcccagtctgccctgactcagcctccctccgcgtccg
IGLV2-8*02      atggcctgggctctgctgcctcctcaccctcctcactcagggcacagggtcctgggcccagtctgccctgactcagcctccctccgcgtcca
IGLV2-8*01      ------------------------------------------------------cagtctgccctgactcagcctccctccgcgtccg
IGLV2-8*03      ----------------------------------------------------------------------------ctccgcgtccg
V_V2-8*01       ------------------------------------------------------cagtctgccctgactcagcctccctccgcgtccg
V_IGLV2-8*02    ------------------------------------------------------cagtctgccctgactcagcctccctccgcgtcca

V2-8_01         ggtctcctggacagtcagtcaccatctcctgcactggaaccagcagtgacgttggtggttataactatgtctcctggtaccaacagcac
V2-8_02         ggtctcctggacagtcagtcaccatctcctgcactggaaccagcagtgacgttggtggttataactatgtctcctggtaccaacagcac
V2-8_03         ggtctcctggacagtcagtcaccatctcctgcactggaaccagcagtgacgttggtggttataactatgtctcctggtaccaacagcac
V2-8_04         ggtctcctggacagtcagtcaccatctcctgcactggaaccagcagtgacgttggtggttataactatgtctcctggtaccaacagcac
IGLV2-8*02      ggtctcctggacagtcagtcaccatctcctgcactggaaccagcagtgacgttggtggttataactatgtctcctggtaccaacagcac
IGLV2-8*01      ggtctcctggacagtcagtcaccatctcctgcactggaaccagcagtgacgttggtggttataactatgtctcctggtaccaacagcac
IGLV2-8*03      ggtctcctggacagtcagtcaccatctcctgcactggaaccagcagtgacgttggtggttataactatgtctcctggtaccaacagcac
V_V2-8*01       ggtctcctggacagtcagtcaccatctcctgcactggaaccagcagtgacgttggtggttataactatgtctcctggtaccaacagcac
V_IGLV2-8*02    ggtctcctggacagtcagtcaccatctcctgcactggaaccagcagtgacgttggtggttataactatgtctcctggtaccaacagcac

V2-8_01         ccaggcaaagcccccaaactcatgatttatgaggtcagtaagcggccctcaggggtccctgatcgcttctctggctccaagtctggcaaca
V2-8_02         ccaggcaaagcccccaaactcatgatttatgaggtcagtaagcggccctcaggggtccctgatcgcttctctggctccaagtctggcaaca
V2-8_03         ccaggcaaagcccccaaactcatgatttatgaggtcagtaagcggccctcaggggtccctgatcgcttctctggctccaagtctggcaaca
V2-8_04         ccaggcaaagcccccaaactcatgatttatgaggtcagtaagcggccctcaggggtccctgatcgcttctctggctccaagtctggcaaca
IGLV2-8*02      ccaggcaaagcccccaaactcatgatttatgaggtcagtaagcggccctcaggggtccctgatcgcttctctggctccaagtctggcaaca
IGLV2-8*01      ccaggcaaagcccccaaactcatgatttatgaggtcagtaagcggccctcaggggtccctgatcgcttctctggctccaagtctggcaaca
IGLV2-8*03      ccaggcaaagcccccaaactcatgatttatgaggtcagtaagcggccctcaggggtccctgatcgcttcttggctccaagtctggcaaca
V_V2-8*01       ccaggcaaagcccccaaactcatgatttatgaggtcagtaagcggccctcaggggtccctgatcgcttctctggctccaagtctggcaaca
V_IGLV2-8*02    ccaggcaaagcccccaaactcatgatttatgaggtcagtaagcggccctcaggggtccctgatcgcttctctggctccaagtctggcaaca

V2-8_01         cggcctccctgaccgtctctgggctccaggctgaggatgaggctgattattactgcagctcatatgcaggcagcaacaatttccaca
V2-8_02         cggcctccctgaccgtctctgggctccaggctgaggatgaggctgattattactgcagctcatatgcaggcagcaacaatttccaca
V2-8_03         cggcctccctgaccgtctctgggctccaggctgaggatgaggctgattattactgcagctcatatgcaggcagcaacaatttccaca
V2-8_04         cggcctccctgaccgtctctgggctccaggctgaggatgaggctgattattactgctgctcatatgcaggcagcaacaatttccaca
IGLV2-8*02      cggcctccctgaccgtctctgggctccaggctgaggatgaggctgattattactgcagctcatatgcaggcagcaacaatttc----
IGLV2-8*01      cggcctccctgaccgtctctgggctccaggctgaggatgaggctgattattactgcagctcatatgcaggcagcaacaatttc----
IGLV2-8*03      cggcctccctgaccgtctctgggctccaggctgaggatgaggctgattattactgcagctcatatgcaggcagcaacaatttc----
V_V2-8*01       cggcctccctgaccgtctctgggctccaggctgaggatgaggctgattattactgcagctcatatgcaggcagcaac----------
V_IGLV2-8*02    cggcctccctgaccgtctctgggctccaggctgaggatgaggctgattattactgcagctcatatgcaggcagcaac----------
```

IGLV2-11

```
V2-11_01      atggccctgggctctgctcctcctcagcctcctcactcagggcacaggatcctgggctcagtctgccctgactcagcctcgctcagtgtccgggt
V2-11_02      atggccctgggctctgctcctcctcagcctcctcactcagggcacaggatcctgggctcagtctgccctgactcagcctcgctcagtgtccgggt
V2-11_03      atgg-cctgggctctgctcctcctcagcctcctcactcagggcacaggatcctgggctcagtctgccctgactcagcctcgctcagtgtccgggt
V2-11_04      atggccctgggctctgctcctcctcagcctcctcactcagggcacaggatcctgggctcagtctgccctgactcagcctcgctcagtgtccgggt
V2-11_05      atggccctgggctctgctcctcctcagcctcctcactcagggcacaggatcctgggctcagtctgccctgactcagcctcgctcagtgtccgggt
V2-11_06      atggccctgggctctgctcctcctcagcctcctcactcagggcacaggatcctgggctcagtctgccctgactcagcctcgctcagtgtccgggt
IGLV2-11*01   ----------------------------------------------------------cagtctgccctgactcagcctcgctcagtgtccgggt
IGLV2-11*02   ----------------------------------------------------------cagtctgccctgactcagcctcgctcagtgtccgggt
IGLV2-11*03   ---------------------------------------------------------------------------------ctcagtgtccgggt
V_V2-11*01    ----------------------------------------------------------cagtctgccctgactcagcctcgctcagtgtccgggt

V2-11_01      ctcctggacagtcagtcaccatctcctgcactggaaccagcagtgatgttggtggttataactatgtctcctggtaccaacagca
V2-11_02      ctcctggacagtcagtcaccatctcctgcactggaaccagcagtgatgttggtggttataactatgtctcctggtaccaacagca
V2-11_03      ctcctggacagtcagtcaccatctcctgcactggaaccagcagtgatgttggtggttataactatgtctcctggtaccaacagca
V2-11_04      ctcctggacagtcagtcaccatctcctgcactggaaccagcagtgatgttggtggttataactatgtctcctggtaccaacagca
V2-11_05      ctcctggacagtcagtcaccatctcctgcactggaaccagcagtgatgttggtggttataactatgtctcctggtaccaacagca
V2-11_06      ctcctggacagtcagtcaccatctcctgcactggaaccagcagtgatgttggtggttataactatgtctcctggtaccaacagca
IGLV2-11*01   ctcctggacagtcagtcaccatctcctgcactggaaccagcagtgatgttggtggttataactatgtctcctggtaccaacagca
IGLV2-11*02   ctcctggacagtcagtcaccatctcctgcactggaaccagcagtgatgttggtgggtataactatgtctcctggtaccaacagca
IGLV2-11*03   ctcctggacagtcagtcaccatctcctgcactggaaccagcagtgatgttggtggttataactatgtctcctggtaccaacaaca
V_V2-11*01    ctcctggacagtcagtcaccatctcctgcactggaaccagcagtgatgttggtggttataactatgtctcctggtaccaacagca

V2-11_01      cccaggcaaagcccccaaactcatgatttatgatgtcagtaagcggccctcaggggtccctgatcgcttctctggctccaagtctggcaac
V2-11_02      cccaggcaaagcccccaaactcatgatttatgatgtcagtaagcggccctcaggggtccctgatcgcttctctggctccaagtctggcaac
V2-11_03      cccaggcaaagcccccaaactcatgatttatgatgtcagtaagcggccctcaggggtccctgatcgcttctctggctccaagtctggcaac
V2-11_04      cccaggcaaagcccccaaactcatgatttatgaggtcagtaagcggccctcaggggtccctgatcgcttctctggctccaagtctggcaac
V2-11_05      cccaggcaaagcccccaaactcatgatttatgatgtcagtaagcggccctcaggggtccctgatcgcttctctggctccaagtctggcaac
V2-11_06      cccaggcaaagcccccaaactcatgatttatgatgtcagtaagcggccctcaggggtttctaatcgcttctctggctccaagtctggcaac
IGLV2-11*01   cccaggcaaagcccccaaactcatgatttatgatgtcagtaagcggccctcaggggtccctgatcgcttctctggctccaagtctggcaac
IGLV2-11*02   cccaggcaaagcccccaaactcatgatttatgatgtcagtaagcggccctcaggggtccctgatcgcttctctggctccaagtctggcaac
IGLV2-11*03   cccaggcaaagcccccaaactcatgatttatgatgtcagtaagcggccctcaggggtccctgatcgcttctctggctccaagtctggcaac
V_V2-11*01    cccaggcaaagcccccaaactcatgatttatgatgtcagtaagcggccctcaggggtccctgatcgcttctctggctccaagtctggcaac

V2-11_01      acggcctccctgaccatctctgggctccaggctgaggatgaggctgattattactgctgctcatatgcaggcagctacactttccaca
V2-11_02      acggcctccctgaccatctctgggctccaggctgaggatgaggctgattattactgcagctcatatgcaggcagctacactttccaca
V2-11_03      acggcctccctgaccatctctgggctccaggctgaggatgaggctgattattactgctgctcatatgcaggcagctacactttccaca
V2-11_04      acggcctccctgaccatctctgggctccaggctgaggatgaggctgattattactgctgctcatatgcaggcagctacactttccaca
V2-11_05      acggcctccctgaccatctctgggctccagggtgaggatgaggctgattattactgctgctcatatgcaggcagctacactttccaca
V2-11_06      acggcctccctgaccatctctgggctccaggctgaggatgaggctgattattactgctgctcatatgcaggcagctacactttccaca
IGLV2-11*01   acggcctccctgaccatctctgggctccaggctgaggatgaggctgattattactgctgctcatatgcaggcagctacactttc----
IGLV2-11*02   acggcctccctgaccatctctgggctccaggctgaggatgaggctgattattactgctgctcatatgcaggcagctacactttc----
IGLV2-11*03   acggcctccctgaccatctctgggctccaggctgaggatgaggctgattattactgctgctcatatgcaggcagctacactttc----
V_V2-11*01    acggcctccctgaccatctctgggctccaggctgaggatgaggctgattattactgctgctcatatgcaggcagctac----------
```

IGLV2-14

```
V2-14_01       atggcctgggctctgctgctcctcacccctcctcactcagggcacaggtcctggcccagtctgccctgactcagcctgcctccgtgtctggg
V2-14_02       atggcctgggctctgctattcctcacccctcctcactcagggcacaggtcctggcccagtctgccctgactcagcctgcctccgtgtctggg
V2-14_03       atggcctgggctctgctattcctcacccctcctcactcagggcacaggtcctggcccagtctgccctgactcagcctgcctccgtgtctggg
V2-14_04       atggcctgggctctgctgctcctcacccctcctcactcagggcacaggtcctggcccagtctgccctgactcagcctgcctccgtgtctggg
V2-14_05       atggcctgggctctgctgttcctcacccctcctcactcagggcacaggtcctggcccagtctgccctgactcagcctgcctccgtgtctggg
V2-14_06       atggcctgggctctgctgttcctcacccctcctcactcagggcacaggtcctggcccagtctgccctgactcagcctgcctccgtgtctggg
V2-14_07       atggcctgggctctgctactcctcacccctcctcactcagggcacaggtcctggcccagtctgccctgactcagcctgcctccgtgtctggg
V2-14_08       atggcctgggctctgctgctcctcacccctcctcactcagggcacaggtcctggcccagtctgccctgactcagcctgcctccgtgtctggg
V2-14_09       atggcctgggctctgctactcctcacccctcctcactcagggcacaggtcctggcccagtctgccctgactcagcctgcctccgtgtctggg
V2-14_10       atggcctgggctctgctgctcctcaacctcctcactcagggcacaggtcctggcccagtctgccctgactcagcctgcctccgtgtctggg
V2-14_11       atggcctgggctctgctattcctcacccctcctcactcagggcacaggtcctggcccagtctgccctgactcagcctgcctccgtgtctggg
V2-14_12       atggcctgggctctgctactcctcacccctcctcactcagggcacaggtcctggcccagtctgccctgactcagcctgcctccgtgtctggg
V2-14_13       atggcctgggctctgctactcctcacccctcctcactcagggcacaggtcctggcccagtctgccctgactcagcctgcctccgtgtctggg
V2-14_14       atggcctgggctctgctgttcctcacccctcctcactcagggcacaggtcctggcccagtctgccctgactcagcctgcctccgtgtctggg
V2-14_15       atggcctgggctctgctactcctcacccctcctcactcagggcacaggtcctggcccagtctgccctgactcagcctgcctccgtgtctggg
V2-14_16       atggcctgggctctgctgctcctcacccctcctcactcagggcacaggtcctggcccagtctgccctgactcagcctgcctccgtgtctggg
V2-14_17       atggcctgggctctgctgctcctcacccctcctcactcagggcacaggtcctggcccagtctgccctgactcagcctgcctccgtgtctggg
V2-14_18       atggcctgggctctgctgctcctcacccctcctcactcagggcacaggtcctggcccagtctgccctgactcagcctgcctccgtgtctggg
V2-14_19       atggcctgggctctgctattcctcacccctcctcactcagggcacaggtcctggcccagtctgccctgactcagcctgcctccgtgtctggg
V2-14_20       atggcctgggctctgctgttcctcacccctcctcactcagggcacaggtcctggcccagtctgccctgactcagcctgcctccgtgtctggg
V2-14_21       atggcctgggctctgctgctcctcacccctcctcactcagggcacaggtcctggcccagtctgccctgactcagcctgcctccgtgtctggg
V2-14_22       atggcctgggctctgctgctcctcacccctcctcactcagggcacaggtcctggcccagtctgccctgactcagcctgcctccgtgtctggg
V2-14_23       atggcctgggctctgctattcctcacccctcctcactcagggcacaggtcctggcccagtctgccctgactcagcctgcctccgtgtctggg
V2-14_24       atggcctgggctctgctgctcctcacccctcctcactcagggcacaggtcctggcccagtctgccctgactcagcctgcctccgtgtctggg
V2-14_25       atggcctgggctctgctattcctcacccctcctcactcagggcacaggtcctggcccagtctgccctgactcagcctgcctccgtgtctggg
V2-14_26       atggcctgggctctgctactcctcacccctcctcactcagggcacaggtcctggcccagtctgccctgactcagcctgcctccgtgtctggg
IGLV2-14*02    atggcctgggctctgctgctcctcactctcctcactcgggacacaggtcctggcccagtctgccctgactcagcctgcctccgtgtctggg
IGLV2-14*01    -------------------------------------------------------cagtctgccctgactcagcctgcctccgtgtctggg
IGLV2-14*03    ------------------------------------------------------------------------------ctccgtgtctggg
IGLV2-14*04    ------------------------------------------------------------------------------ctccgtgtctggg
IP_V2-14*p05   -------------------------------------------------------cagtctgccctgactcagcctgcctccgtgtctggg
IP_V2-14*p06   -------------------------------------------------------cagtctgccctgactcagcctgcctccgtgtctggg
IP_V2-14*p07   -------------------------------------------------------cagtctgccctgactcagcctgcctccgtgtctggg
IP_V2-14*p08   -------------------------------------------------------cagtctgccctgactcagcctgcctccgtgtctggg
IP_V2-14*p05   -------------------------------------------------------cagtctgccctgactcagcctgcctccgtgtctggg
IP_V2-14*p06   -------------------------------------------------------cagtctgccctgactcagcctgcctccgtgtctggg
IP_V2-14*p07   -------------------------------------------------------cagtctgccctgactcagcctgcctccgtgtctggg
IP_V2-14*p08   -------------------------------------------------------cagtctgccctgactcagcctgcctccgtgtctggg
V_V2-14*04     -------------------------------------------------------cagtctgccctgactcagcctgcctccgtgtctggg
V_V2-14*01     -------------------------------------------------------cagtctgccctgactcagcctgcctccgtgtctggg
V_V2-14*02     -------------------------------------------------------cagtctgccctgactcagcctgcctccgtgtctggg
```

```
V2-14_01        tctcctggacagtcgatcaccatctcctgcactggaaccagcagtgacgttggtggttataactatgtctcctggtaccaacagcac
V2-14_02        tctcctggacagtcgatcaccatctcctgcactggaaccagcagtgacgttggtggttataactatgtctcctggtaccaacaacac
V2-14_03        tctcctggacagtcgatcaccatctcctgcactggaaccagcagtgacgttggtggttataactatgtctcctggtaccaacagcac
V2-14_04        tctcctggacagtcgatcaccatctcctgcactggaaccagcagtgacgttggtggttataactatgtctcctggtaccaacagcac
V2-14_05        tctcctggacagtcgatcaccatctcctgcactggaaccagcagtgacgttggtggttataactatgtctcctggtaccaacagcac
V2-14_06        tctcctggacagtcgatcaccatctcctgcactggaaccagcagtgacgttggtggttataactatgtctcctggtaccaacagcac
V2-14_07        tctcctggacagtcgatcaccatctcctgcactggaaccagcagtgacgttggtggttataactatgtctcctggtaccaacagcac
V2-14_08        tctcctggacagtcgatcaccatctcctgcactggaaccagcagtgacgttggtggttataactatgtctcctggtaccaacagcac
V2-14_09        tctcctggacagtcgatcaccatctcctgcactggaaccagcagtgacgttggtggttataactatgtctcctggtaccaacaacac
V2-14_10        tctcctggacagtcgatcaccatctcctgcactggaaccagcagtgacgttggtggttataactatgtctcctggtaccaacagcac
V2-14_11        tctcctggacagtcgatcaccatctcctgcactggaaccagcagtgacgttggtggttataactatgtctcctggtaccaacagcac
V2-14_12        tctcctggacagtcgatcaccatctcctgcactggaaccagcagtgacgttggtggttataactatgtctcctggtaccaacagcac
V2-14_13        tctcctggacagtcgatcaccatctcctgcactggaaccagcagtgacgttggtggttataactatgtctcctggtaccaacaacac
V2-14_14        tctcctggacagtcgatcaccatctcctgcactggaaccagcagtgacgttggtggttataactatgtctcctggtaccaacaacac
V2-14_15        tctcctggacagtcgatcaccatctcctgcactggaaccagcagtgacgttggtggttataactatgtctcctggtaccaacagcac
V2-14_16        tctcctggacagtcgatcaccatctcctgcactggaaccagcagtgacgttggtggttataactatgtctcctggtaccaacaacac
V2-14_17        tctcctggacagtcgatcaccatctcctgcactggaaccagcagtgacgttggtggttataactatgtctcctggtaccaacagcac
V2-14_18        tctcctggacagtcgatcaccatctcctgcactggaaccagcagtgacgttggtggttataactatgtctcctggtaccaacaacac
V2-14_19        tctcctggacagtcgatcaccatctcctgcactggaaccagcagtgacgttggtggttataactatgtctcctggtaccaacagcac
V2-14_20        tctcctggacagtcgatcaccatctcctgcactggaaccagcagtgacgttggtggttataactatgtctcctggtaccaacaacac
V2-14_21        tctcctggacagtcgatcaccatctcctgcactggaaccagcagtgacgttggtggttataactatgtctcctggtaccaacaacac
V2-14_22        tctcctggacagtcgatcaccatctcctgcactggaaccagcagtgacgttggtggttataactatgtctcctggtaccaacagcac
V2-14_23        tctcctggacagtcgatcaccatctcctgcactggaaccagcagtgacgttggtggttataactatgtctcctggtaccaacaacac
V2-14_24        tctcctggacagtcgatcaccatctcctgcactggaaccagcagtgacgttggtggttataactatgtctcctggtaccaacagcac
V2-14_25        tctcctggacagtcgatcaccatctcctgcactggaaccagcagtgacgttggtggttataactatgtctcctggtaccaacagccc
V2-14_26        tctcctggacagtcgatcaccatctcctgcactggaaccagcagtgacgttggtggttataactatgtctcctggtaccaacagcac
IGLV2-14*02     tctcctggacagtcgatcaccatctcctgcactggaaccagcagtgatgttgggagttataacctgtctcctggtaccaacagcac
IGLV2-14*01     tctcctggacagtcgatcaccatctcctgcactggaaccagcagtgacgttggtggttataactatgtctcctggtaccaacagcac
IGLV2-14*03     tctcctggacagtcgatcaccatctcctgcactggaaccagcagtgacgttggtggttataactatgtctcctggtaccaacaacac
IGLV2-14*04     tctcctggacagtcgatcaccatctcctgcactggaaccagcagtgacgttggtggttataactatgtctcctggtaccaacagcac
IP_V2-14*p05    tctcctggacagtcgatcaccatctcctgcactggaaccagcagtgacgttggtggttataactatgtctcctggtaccaacaacac
IP_V2-14*p06    tctcctggacagtcgatcaccatctcctgcactggaaccagcagtgacgttggtggttataactatgtctcctggtaccaacaacac
IP_V2-14*p07    tctcctggacagtcgatcaccatctcctgcactggaaccagcagtgacgttggtggttataactatgtctcctggtaccaacagcac
IP_V2-14*p08    tctcctggacagtcgatcaccatctcctgcactggaaccagcagtgacgttggtggttataactatgtctcctggtaccaacagcac
IP_V2-14*p05    tctcctggacagtcgatcaccatctcctgcactggaaccagcagtgacgttggtggttataactatgtctcctggtaccaacaacac
IP_V2-14*p06    tctcctggacagtcgatcaccatctcctgcactggaaccagcagtgacgttggtggttataactatgtctcctggtaccaacaacac
IP_V2-14*p07    tctcctggacagtcgatcaccatctcctgcactggaaccagcagtgacgttggtggttataactatgtctcctggtaccaacagcac
IP_V2-14*p08    tctcctggacagtcgatcaccatctcctgcactggaaccagcagtgacgttggtggttataactatgtctcctggtaccaacagcac
V_V2-14*04      tctcctggacagtcgatcaccatctcctgcactggaaccagcagtgacgttggtggttataactatgtctcctggtaccaacagcac
V_V2-14*01      tctcctggacagtcgatcaccatctcctgcactggaaccagcagtgacgttggtggttataactatgtctcctggtaccaacagcac
V_V2-14*02      tctcctggacagtcgatcaccatctcctgcactggaaccagcagtgatgttgggagttataacctgtctcctggtaccaacagcac
```

```
V2-14_01        ccaggcaaagcccccaaactcatgatttatgaggtcagtaatcggccctcaggggtttctaatcgcttctctggctccaagtctggcaaca
V2-14_02        ccaggcaaagcccccaaactcatgatttatgatgtcagtaatcggccctcaggggtttctaatcgcttctctggctccaagtctggcaaca
V2-14_03        ccaggcaaagcccccaaactcatgatttatgatgtcagtaatcggccctcaggggtttctaatcgcttctctggctccaagtctggcaaca
V2-14_04        ccaggcaaagcccccaaactcatgatttatgatgtcagtaagcggccctcaggggtttctaatcgcttctctggctccaagtctggcaaca
V2-14_05        ccaggcaaagcccccaaactcatgatttatgaggtcagtaatcggccctcaggggtttctaatcgcttctctggctccaagtctggcaaca
V2-14_06        ccaggcaaagcccccaaactcatgatttatgatgtcagtaatcggccctcaggggtttctaatcgcttctctggctccaagtctggcaaca
V2-14_07        ccaggcaaagcccccaaactcatgatttatgatgtcagtaatcggccctcaggggtttctaatcgcttctctggctccaagtctggcaaca
V2-14_08        ccaggcaaagcccccaaactcatgatttatgatgtcagtaatcggccctcaggggtttctaatcgcttctctggctccaagtctggcaaca
V2-14_09        ccaggcaaagcccccaaactcatgatttatgatgtcagtaatcggccctcaggggtttctaatcgcttctctggctccaagtctggcaaca
V2-14_10        ccaggcaaagcccccaaactcatgatttatgaggtcagtaatcggccctcaggggtttctaatcgcttctctggctccaagtctggcaaca
V2-14_11        ccaggcaaagcccccaaactcatgatttatgaggtcagtaatcggccctcaggggtttctaatcgcttctctggctccaagtctggcaaca
V2-14_12        ccaggcaaagcccccaaactcatgatttatgaggtcagtaatcggccctcaggggtttctaatcgcttctctggctccaagtctggcaaca
V2-14_13        ccaggcaaagcccccaaactcatgatttatgatgtcagtaagcggccctcaggggtttctaatcgcttctctggctccaagtctggcaaca
V2-14_14        ccaggcaaagcccccaaactcatgatttatgatgtcagtaatcggccctcaggggtttctaatcgcttctctggctccaagtctggcaaca
V2-14_15        ccaggcaaagcccccaaactcatgatttatgatgtcagtaagcggccctcaggggtttctaatcgcttctctggctccaagtctggcaaca
V2-14_16        ccaggcaaagcccccaaactcatgatttatgaggtcagtaatcggccctcaggggtttctaatcgcttctctggctccaagtctggcaaca
V2-14_17        ccaggcaaagcccccaaactcatgatttatgaggtcagtaagcggccctcaggggtttctaatcgcttctctggctccaagtctggcaaca
V2-14_18        ccaggcaaagcccccaaactcatgatttatgatgtcagtaatcggccctcaggggtttctaatcgcttctctggctccaagtctggcaaca
V2-14_19        ccaggcaaagcccccaaactcatgatttatgatgtcagtaagcggccctcaggggtttctaatcgcttctctggctccaagtctggcaaca
V2-14_20        ccaggcaaagcccccaaactcatgatttatgaggtcagtaatcggccctcaggggtttctaatcgcttctctggctccaagtctggcaaca
V2-14_21        ccaggcaaagcccccaaactcatgatttatgatgtcagtaagcggccctcaggggtttctaatcgcttctctggctccaagtctggcaaca
V2-14_22        ccaggcaaagcccccaaactcatgatttatgatgtcagtaagcggccctcaggggtttctaatcgcttctctggctccaagtctggcaaca
V2-14_23        ccaggcaaagcccccaaactcatgatttatgatgtcagtaagcggccctcaggggtttctaatcgcttctctggctccaagtctggcaaca
V2-14_24        ccaggcaaagcccccaaactcatgatttatgaggtcagtaagcggccctcaggggtttctaatcgcttctctggctccaagtctggcaaca
V2-14_25        ccaggcaaagcccccaaactcatgatttatgatgtcagtaatcggccctcaggggtttctaatcgcttctctggctccaagtctggcaaca
V2-14_26        ccaggcaaagcccccaaactcatgatttatgatgtcagtaagcggccctcaggggtttctaatcgcttctctggctccaagtctggcaaca
IGLV2-14*02     ccaggcaaagcccccaaactcatgatttatgagggcagtaagcggccctcaggggtttctaatcgcttctctggctccaagtctggcaaca
IGLV2-14*01     ccaggcaaagcccccaaactcatgatttatgaggtcagtaatcggccctcaggggtttctaatcgcttctctggctccaagtctggcaaca
IGLV2-14*03     ccaggcaaagcccccaaactcatgatttatgatgtcagtaatcggccctcaggggtttctaatcgcttctctggctccaagtctggcaaca
IGLV2-14*04     ccaggcaaagcccccaaactcatgatttatgatgtcagtaatcggccctcaggggtttctaatcgcttctctggctccaagtctggcaaca
IP_V2-14*p05    ccaggcaaagcccccaaactcatgatttatgatgtcagtaatcggccctcaggggtttctaatcgcttctctggctccaagtctggcaaca
IP_V2-14*p06    ccaggcaaagcccccaaactcatgatttatgatgtcagtaatcggccctcaggggtttctaatcgcttctctggctccaagtctggcaaca
IP_V2-14*p07    ccaggcaaagcccccaaactcatgatttatgatgtcagtaatcggccctcaggggtttctaatcgcttctctggctccaagtctggcaaca
IP_V2-14*p08    ccaggcaaagcccccaaactcatgatttatgatgtcagtaagcggccctcaggggtttctaatcgcttctctggctccaagtctggcaaca
IP_V2-14*p05    ccaggcaaagcccccaaactcatgatttatgatgtcagtaatcggccctcaggggtttctaatcgcttctctggctccaagtctggcaaca
IP_V2-14*p06    ccaggcaaagcccccaaactcatgatttatgatgtcagtaatcggccctcaggggtttctaatcgcttctctggctccaagtctggcaaca
IP_V2-14*p07    ccaggcaaagcccccaaactcatgatttatgatgtcagtaatcggccctcaggggtttctaatcgcttctctggctccaagtctggcaaca
IP_V2-14*p08    ccaggcaaagcccccaaactcatgatttatgatgtcagtaagcggccctcaggggtttctaatcgcttctctggctccaagtctggcaaca
V_V2-14*04      ccaggcaaagcccccaaactcatgatttatgatgtcagtaatcggccctcaggggtttctaatcgcttctctggctccaagtctggcaaca
V_V2-14*01      ccaggcaaagcccccaaactcatgatttatgaggtcagtaatcggccctcaggggtttctaatcgcttctctggctccaagtctggcaaca
V_V2-14*02      ccaggcaaagcccccaaactcatgatttatgagggcagtaagcggccctcaggggtttctaatcgcttctctggctccaagtctggcaaca
```

V2-14_01	cggcctccctgaccatctctgggctccaggctgaggacgaggctgattattactgcagctcatatacaagcagcagcactctccacagtg
V2-14_02	cggcctccctgaccatctctgggctccaggctgaggacgaggctgattattactgcagctcatatacaagcagcagcactctccacagtg
V2-14_03	cggcctccctgaccatctctgggctccaggctgaggacgaggctgattattactgcagctcatatacaagcagcagcactctccacagtg
V2-14_04	cggcctccctgaccatctctgggctccaggctgaggacgaggctgattattactgcagctcatatacaagcagcagcactttccacagtg
V2-14_05	cggcctccctgaccatctctgggctccaggctgaggacgaggctgattattactgcagctcatatacaagcagcagcactctccacagtg
V2-14_06	cggcctccctgaccatctctgggctccaggctgaggacgaggctgattattactgcagctcatatacaagcagcagcactctccacagtg
V2-14_07	cggcctccctgaccatctctgggctccaggctgaggacgaggctgattattactgcagctcatatacaagcagcagcactctccacagtg
V2-14_08	cggcctccctgaccatctctgggctccaggctgaggacgaggctgattattactgcagctcatatacaagcagcagcactctccacagtg
V2-14_09	cggcctccctgaccatctctgggctccaggctgaggacgaggctgattattactgcagctcatatacaagcagcagcactctccacagtg
V2-14_10	cggcctccctgaccatctctgggctccaggctgaggacgaggctgattattactgcagctcatatacaagcagcagcactctccacagtg
V2-14_11	cggcctccctgaccatctctgggctccaggctgaggacgaggctgattattactgcagctcatatacaagcagcagcactctccacagtg
V2-14_12	cggcctccctgaccatctctgggctccaggctgaggacgaggctgattattactgcagctcatatacaagcagcagcactctccacagtg
V2-14_13	cggcctccctgaccatctctgggctccaggctgaggacgaggctgattattactgcagctcatatacaagcagcagcactttccacagtg
V2-14_14	cggcctccctgaccatctctgggctccaggctgaggacgaggctgattattactgcagctcatatacaagcagcagcactctccacagtg
V2-14_15	cggcctccctgaccatctctgggctccaggctgaggacgaggctgattattactgcagctcatatacaagcagcagcactttccacagtg
V2-14_16	cggcctccctgaccatctctgggctccaggctgaggacgaggctgattattactgcagctcatatacaagcagcagcactctccacagtg
V2-14_17	cggcctccctgaccatctctgggctccaggctgaggacgaggctgattattactgcagctcatatacaagcagcagcactttccacagtg
V2-14_18	cggcctccctgaccatctctgggctccaggctgaggacgaggctgattattactgcagctcatatacaagcagcagcactctccacagtg
V2-14_19	cggcctccctgaccatctctgggctccaggctgaggacgaggctgattattactgcagctcatatacaagcagcagcactctccacagtg
V2-14_20	cggcctccctgaccatctctgggctccaggctgaggacgaggctgattattactgcagctcatatacaagcagcagcactctccacagtg
V2-14_21	cggcctccctgaccatctctgggctccaggctgaggacgaggctgattattactgcagctcatatacaagcagcagcactttccacagtg
V2-14_22	cggcctccctgaccatctctgggctccaggctgaggacgaggctgattattactgcagctcatatacaagcagcagcactctccacagtg
V2-14_23	cggcctccctgaccatctctgggctccaggctgaggacgaggctgattattactgcagctcatatacaagcagcagcactctccacagtg
V2-14_24	cggcctccctgaccatctctgggctccaggctgaggacgaggctgattattactgcagctcatatacaagcagcagcactctccacagtg
V2-14_25	cggcctccctgaccatctctgggctccaggctgaggacgaggctgattattactgcagctcatatacaagcagcagcactctccacagtg
V2-14_26	cggcctccctgaccatctctgggctccaggctgaggacgaggctgattattactgcagctcatatacaagcagcagcactctccacagtg
IGLV2-14*02	cggcctccctgaccatctctgggctccaggctgaggacgaggctgattattactgcagctcatatacaagcagcagcactctc-------
IGLV2-14*01	cggcctccctgaccatctctgggctccaggctgaggacgaggctgattattactgcagctcatatacaagcagcagcactctc-------
IGLV2-14*03	cggcctccctgaccatctctgggctccaggctgaggacgaggctgattattactgcagctcatatacaagcagcagcactctc-------
IGLV2-14*04	cggcctccctgaccatctctgggctccaggctgaggacgaggctgattattactgcagctcatatacaagcagcagcactctc-------
IP_V2-14*p05	cggcctccctgaccatctctgggctccaggctgaggacgaggctgattattactgcagctcatatacaagcagcagcactctc-------
IP_V2-14*p06	cggcctccctgaccatctctgggctccaggctgaggacgaggctgattattactgcagctcatatacaagcagcaccactctc-------
IP_V2-14*p07	cggcctccctgaccatctctgggctccaggctgaggacgaggctgattattactgcagctcatatacaagcagcagcactctc-------
IP_V2-14*p08	cggcctccctgaccatctctgggctccaggctgaggacgaggctgattattactgcagctcatatacaagcagcagcactctc-------
IP_V2-14*p05	cggcctccctgaccatctctgggctccaggctgaggacgaggctgattattactgcagctcatatacaagcagcagcactctc-------
IP_V2-14*p06	cggcctccctgaccatctctgggctccaggctgaggacgaggctgattattactgcagctcatatacaagcagcaccactctc-------
IP_V2-14*p07	cggcctccctgaccatctctgggctccaggctgaggacgaggctgattattactgcagctcatatacaagcagcagcactctc-------
IP_V2-14*p08	cggcctccctgaccatctctgggctccaggctgaggacgaggctgattattactgcagctcatatacaagcagcagcactctc-------
V_V2-14*04	cggcctccctgaccatctctgggctccaggctgaggacgaggctgattattactgcagctcatatacaagcagcagc-------------
V_V2-14*01	cggcctccctgaccatctctgggctccaggctgaggacgaggctgattattactgcagctcatatacaagcagcagc-------------
V_V2-14*02	cggcctccctgaccatctctgggctccaggctgaggacgaggctgattattactgcagctcatatacaagcagcagc-------------

IGLV2-18

```
V2-18_01         atggcctgggctctgctc---ctcctcaccctcctcactcagggcacaggatcctgggctcagtctgccctgactcagcctccctccgtgt
V2-18_02         atggcctgggctctgctc---ctcctcaccctcctcactcagggcacaggatcctgggctcagtctgccctgactcagcctccctccgtgt
V2-18_03         atggcctgggctctgcta---ttcctcaccctcctcactcagggcacaggatcctgggctcagtctgccctgactcagcctccctccgtgt
V2-18_04         atggcctgggctctgctc---ctcctcaccctcctcactcagggcacagggtcctgggctcagtctgccctgactcagcctccctccgtgt
V2-18_05         atggcctgggctctgctc---ctcctcaccctcctcactcagggcacaggatcctgggctcagtctgccctgactcagcctccctccgtgt
V2-18_06         atggcctgggctctgctc---ctcctcaccctcctcactcagggcacaggatcctgggctcagtctgccctgactcagcctccctccgtgt
IGLV2-18*03      atggcctgggctctgctgctcctcctcaccctcctcactcagggcacaggatcctgggctcagtctgccctgactcagcctccctccgtgt
IGLV2-18*04      atggcctgggctctgctg---ctcctcaccctcctcactcagggcacaggatcctgggctcagtctgccctgactcagcctccctccgtgt
IGLV2-18*01      ------------------------------------------------------------cagtctgccctgactcagcctccctccgtgt
IGLV2-18*02      ------------------------------------------------------------cagtctgccctgactcagcctccctccgtgt
V_V2-18*04       ------------------------------------------------------------cagtctgccctgactcagcctccctccgtgt
V_V2-18*03       ------------------------------------------------------------cagtctgccctgactcagcctccctccgtgt
V_V2-18*01/*02   ------------------------------------------------------------cagtctgccctgactcagcctccctccgtgt

V2-18_01         ccgggtctcctggacagtcagtcaccatctcctgcactggaaccagcagtgacgttggtagttataaccgtgtctcctggtaccagcag
V2-18_02         ccgggtctcctggacagtcagtcaccatctcctgcactggaaccagcagtgacgttggtagttataaccgtgtctcctggtaccagcag
V2-18_03         ccgggtctcctggacagtcagtcaccatctcctgcactggaaccagcagtgacgttggtagttataaccgtgtctcctggtaccagcag
V2-18_04         ccgggtctcctggacagtcagtcaccatctcctgcactggaaccagcagtgacgttggtagttataaccgtgtctcctggtaccagcag
V2-18_05         ccgggtctcctggacagtcagtcaccatctcctgcactggaaccagcagtgacgttggtagttataaccgtgtctcctggtaccagcag
V2-18_06         ccgggtctcctggacagtcagtcaccatctcctgcactggaaccagcagtgacgttggtagttataaccgtgtctcctggtaccagcag
IGLV2-18*03      ccgggtctcctggacagtcagtcaccatctcctgcactggaaccagcagtgacgttggtagttataaccgtgtctcctggtaccagcag
IGLV2-18*04      ccgggtctcctggacagtcagtcaccatctcctgcactggaaccagcagtgacgttggtagttataaccgtgtctcctggtaccagcag
IGLV2-18*01      ccgggtctcctggacagtcagtcaccatctcctgcactggaaccagcagtgacgttggtagttataaccgtgtctcctggtaccagcag
IGLV2-18*02      ccgggtctcctggacagtcagtcaccatctcctgcactggaaccagcagtgacgttggtagttataaccgtgtctcctggtaccagcag
V_V2-18*04       ccgggtctcctggacagtcagtcaccatctcctgcactggaaccagcagtgacgttggtagttataaccgtgtctcctggtaccagcag
V_V2-18*03       ccgggtctcctggacagtcagtcaccatctcctgcactggaaccagcagtgacgttggtagttataaccgtgtctcctggtaccagcag
V_V2-18*01/*02   ccgggtctcctggacagtcagtcaccatctcctgcactggaaccagcagtgacgttggtagttataaccgtgtctcctggtaccagcag

V2-18_01         cccccaggcacagcccccaaactcatgatttatgaggtcagtaatcggccctcaggggtccctgatcgcttctctgggtccaagtctggca
V2-18_02         cccccaggcacagcccccaaactcatgatttatgaggtcagtaatcggccctcaggggtccctgatcgcttctctgggtccaagtctggca
V2-18_03         cccccaggcacagcccccaaactcatgatttatgaggtcagtaatcggccctcaggggtccctgatcgcttctctgggtccaagtctggca
V2-18_04         cccccaggcacagcccccaaactcatgatttatgaggtcagtaatcggccctcaggggtccctgatcgcttctctgggtccaagtctggca
V2-18_05         cccccaggcacagcccccaaactcatgatttatgatgtcagtaatcggccctcaggggtccctgatcgcttctctgggtccaagtctggca
V2-18_06         cccccaggcgcagcccccaaactcatgatttatgaggtcagtaatcggccctcaggggtccctgatcgcttctctgggtccaagtctggca
IGLV2-18*03      cccccaggcacagcccccaaactcatgatttatgaggtcagtaatcggccctcaggggtccctgatcgcttctctgggtccaagtctggca
IGLV2-18*04      cccccaggcacagcccccaaactcatgatttatgaggtcagtaatcggccctcaggggtccctgatcgctcctctgggtccaagtccggca
IGLV2-18*01      cccccaggcacagcccccaaactcatgatttatgaggtcagtaatcggccctcaggggtccctgatcgcttctctgggtccaagtctggca
IGLV2-18*02      cccccaggcacagcccccaaactcatgatttatgaggtcagtaatcggccctcaggggtccctgatcgcttctctgggtccaagtctggca
V_V2-18*04       cccccaggcacagcccccaaactcatgatttatgaggtcagtaatcggccctcaggggtccctgatcgctcctctgggtccaagtccggca
V_V2-18*03       cccccaggcacagcccccaaactcatgatttatgaggtcagtaatcggccctcaggggtccctgatcgcttctctgggtccaagtctggca
V_V2-18*01/*02   cccccaggcacagcccccaaactcatgatttatgaggtcagtaatcggccctcaggggtccctgatcgcttctctgggtccaagtctggca
```

```
V2-18_01         acacggcctccctgaccatctctgggctccaggctgaggacgaggctgattattactgcagcttatatacaagcagcagcactttccacagag
V2-18_02         acacggcctccctgaccatctctgggctccaggctgaggacgaggctgattattactgcagctcatatacaagcagcagcactttccacagag
V2-18_03         acacggcctccctgaccatctctgggctccaggctgaggacgaggctgattattactgcagctcatatacaagcagcagcactttccacagag
V2-18_04         acacggcctccctgaccatctctgggctccaggctgaggacgaggctgattattactgcagcttatatacaagcagcagcactttccacagag
V2-18_05         acacggcctccctgaccatctctgggctccaggctgaggacgaggctgattattactgcagctcatatacaagcagcagcactttccacagag
V2-18_06         acacggcctccctgaccatctctgggctccaggctgaggacgaggctgattattactgcagctcatatacaagcagcagcactttccacagag
IGLV2-18*03      acacggcctccctgaccacctctgggctccaggctgaggacgaggctgattattactgcagctcatatacaagcagcagcactttc-------
IGLV2-18*04      acacggcctccctgaccatctctgggctccaggctgaggacgaggctgattattactgcagctcatatacaagcagcagcactttc-------
IGLV2-18*01      acacggcctccctgaccatctctgggctccaggctgaggacgaggctgattattactgcagcttatatacaagcagcagcactttc-------
IGLV2-18*02      acacggcctccctgaccatctctgggctccaggctgaggacgaggctgattattactgcagctcatatacaagcagcagcactttc-------
V_V2-18*04       acacggcctccctgaccatctctgggctccaggctgaggacgaggctgattattactgcagctcatatacaagcagcagc-------------
V_V2-18*03       acacggcctccctgaccacctctgggctccaggctgaggacgaggctgattattactgcagctcatatacaagcagcagc-------------
V_V2-18*01/*02   acacggcctccctgaccatctctgggctccaggctgaggacgaggctgattattactgcagcttatatacaagcagcagc-------------
```

IGLV2-23

```
V2-23_01        atggcctgggctctgctgctcctcactctcctcactcaggacacagggtcctgggcccagtctgccctgactcagcctgcctccgtgtc
V2-23_02        atggcctgggctctgctgctcctcaccctcctcactcaggacacagggtcctgggcccagtctgccctgactcagcctgcctccgtgtc
V2-23_03        atggcctgggctctgctgctcctcaacctcctcactcaggacacagggtcctgggcccagtctgccctgactcagcctgcctccgtgtc
V2-23_04        atggcctgggctctgctgctcctcaacctcctcactcaggacacagggtcctgggcccagtctgccctgactcagcctgcctccgtgtc
V2-23_05        atggcctgggctctgctgctcctcactctcctcactcaggacacagggtcctgggcccagtctgccctgactcagcctgcctccgtgtc
V2-23_06        atggcctgggctctgctgctcctcactctcctcactcaggacacagggtcctgggcccagtctgccctgactcagcctgcctccgagtc
V2-23_07        atggcctgggctctgctgctcctcaccctcctcactcaggacacagggtcctgggcccagtctgccctgactcagcctgcctccgtgtc
V2-23_08        atggcctgggctctgctgctcctcaacctcctcactcaggacacagggtcctgggcccagtctgccctgactcagcctgcctccgtgtc
IGLV2-23*01     atggcctgggctctgctgctcctcactctcctcactcaggacacagggtcctgggcccagtctgccctgactcagcctgcctccgtgtc
IGLV2-23*03     atggcctgggctctgctgctcctcactctcctcactcaggacacagggtcctgggcccagtctgccctgactcagcctgcctccgtgtc
IGLV2-23*02     ---------------------------------------------------------cagtctgccctgactcagcctgcctccgtgtc
IP_V2-23*p04    ---------------------------------------------------------cagtctgccctgactcagcctgcctccgtgtc
IP_V2-23*p04    ---------------------------------------------------------cagtctgccctgactcagcctgcctccgtgtc
V_V2-23*01_*03  ---------------------------------------------------------cagtctgccctgactcagcctgcctccgtgtc

V2-23_01        tgggtctcctggacagtcgatcaccatctcctgcactggaaccagcagtgatgttgggagttataaccttgtctcctggtaccaacagcac
V2-23_02        tgggtctcctggacagtcgatcaccatctcctgcactggaaccagcagtgatgttgggagttataaccttgtctcctggtaccaacagcac
V2-23_03        tgggtctcctggacagtcgatcaccatctcctgcactggaaccagcagtgatgttgggagttataaccttgtctcctggtaccaacagcac
V2-23_04        tgggtctcctggacagtcgatcaccatctcctgcactggaaccagcagtgatgttgggagttataaccttgtctcctggtaccaacagcac
V2-23_05        tgggtctcctggacagtcgatcaccatctcctgcactggaaccagcagtgatgttgggagttataaccttgtctcctggtaccaacagcac
V2-23_06        tgggtctcctggacagtcgatcaccatctcctgcactggaaccagcagtgatgttgggagttataaccttgtctcctggtaccaacagcac
V2-23_07        tgggtctcctggacagtcgatcaccatctcctgcactggaaccagcagtgatgttgggagttataaccttgtctcctggtaccaacagcac
V2-23_08        tgggtctcctggacagtcgatcaccatctcctgcactggaaccagcagtgatgttgggagttataaccttgtctcctggtaccaacagcac
IGLV2-23*01     tgggtctcctggacagtcgatcaccatctcctgcactggaaccagcagtgatgttgggagttataaccttgtctcctggtaccaacagcac
IGLV2-23*03     tgggtctcctggacagtcgatcaccatctcctgcactggaaccagcagtgatgttgggagttataaccttgtctcctggtaccaacagcac
IGLV2-23*02     tgggtctcctggacagtcgatcaccatctcctgcactggaaccagcagtgatgttgggagttataaccttgtctcctggtaccaacagcac
IP_V2-23*p04    tgggtctcctggacagtcgatcaccatctcctgcactggaaccagcagtgatgttgggagttataaccttgtctcctggtaccaacagcac
IP_V2-23*p04    tgggtctcctggacagtcgatcaccatctcctgcactggaaccagcagtgatgttgggagttataaccttgtctcctggtaccaacagcac
V_V2-23*01_*03  tgggtctcctggacagtcgatcaccatctcctgcactggaaccagcagtgatgttgggagttataaccttgtctcctggtaccaacagcac
```

```
V2-23_01         ccaggcaaagcccccaaactcatgatttatgagggcagtaagcggccctcaggggtttctaatcgcttctctggctccaagtctggcaacac
V2-23_02         ccaggcaaagcccccaaactcatgatttatgagggcagtaagcggccctcaggggtttctaatcgcttctctggctccaagtctggcaacac
V2-23_03         ccaggcaaagcccccaaactcatgatttatgagggcagtaagcggccctcaggggtttctaatcgcttctctggctccaagtctggcaacac
V2-23_04         ccaggcaaagcccccaaactcatgatttatgatgtcagtaagcggccctcaggggtttctaatcgcttctctggctccaagtctggcaacac
V2-23_05         ccaggcaaagcccccaaactcatgatttatgatgtcagtaagcggccctcaggggtttctaatcgcttctctggctccaagtctggcaacac
V2-23_06         ccaggcaaagcccccaaactcatgatttatgagggcagtaagcggccctcaggggtttctaatcgcttctctggctccaagtctggcaacac
V2-23_07         ccaggcaaagcccccaaactcatgatttatgatgtcagtaagcggccctcaggggtttctaatcgcttctctggctccaagtctggcaacac
V2-23_08         ccaggcaaagcccccaaactcatgatttatgatgtcagtaagcggccctcaggggtttctaatcgcttctctggctccaagtctggcaacac
IGLV2-23*01      ccaggcaaagcccccaaactcatgatttatgagggcagtaagcggccctcaggggtttctaatcgcttctctggctccaagtctggcaacac
IGLV2-23*03      ccaggcaaagcccccaaactcatgatttatgagggcagtaagcggccctcaggggtttctaatcgcttctctggctccaagtctggcaacac
IGLV2-23*02      ccaggcaaagcccccaaactcatgatttatgaggtcagtaagcggccctcaggggtttctaatcgcttctctggctccaagtctggcaacac
IP_V2-23*p04     ccaggcaaagcccccaaactcatgatttatgacgtcagtaagcggccctcaggggtttctaatcgcttctctggctccaagtctggcaacac
IP_V2-23*p04     ccaggcaaagcccccaaactcatgatttatgacgtcagtaagcggccctcaggggtttctaatcgcttctctggctccaagtctggcaacac
V_V2-23*01_*03   ccaggcaaagcccccaaactcatgatttatgagggcagtaagcggccctcaggggtttctaatcgcttctctggctccaagtctggcaacac
```

```
V2-23_01         ggcctccctgacaatctctgggctccaggctgaggacgaggctgattattactgctgctcatatgcag---------------
V2-23_02         ggcctccctgacaatctctgggctccaggctgaggacgaggctgattattactgctgctcatatgcag---------------
V2-23_03         ggcctccctgacaatctctgggctccaggctgaggacgaggctgattattactgctgctcatatgcag---------------
V2-23_04         ggcctccctgacaatctctgggctccaggctgaggacgaggctgattattactgctgctcatatgcag---------------
V2-23_05         ggcctccctgacaatctctgggctccaggctgaggacgaggctgattattactgctgctcatatgcag---------------
V2-23_06         ggcctccctgacaatctctgggctccaggctgaggacgaggctgattattactgctgctcatatgcag---------------
V2-23_07         ggcctccctgacaatctctgggctccaggctgaggacgaggctgattattactgctgctcatatgcag---------------
V2-23_08         ggcctccctgacaatctctggactccaggctgaggacgaggctgattattactgctgctcatatgcag---------------
IGLV2-23*01      ggcctccctgacaatctctgggctccaggctgaggacgaggctgattattactgctgctcatatgcaggtagtagcactttac
IGLV2-23*03      ggcctccctgacaatctctgggctccaggctgaggacgaggctgattattactgctgctcatatgcaggtagtagcactttc-
IGLV2-23*02      ggcctccctgacaatctctgggctccaggctgaggacgaggctgattattactgctgctcatatgcaggtagtagcactttc-
IP_V2-23*p04     ggcctccctgacaatctctgggctccaggctgaggacgaggctgattattactgctgctcatatgcaggtagtagcactttc-
IP_V2-23*p04     ggcctccctgacaatctctgggctccaggctgaggacgaggctgattattactgctgctcatatgcaggtagtagcactttc-
V_V2-23*01_*03   ggcctccctgacaatctctgggctccaggctgaggacgaggctgattattactgctgctcatatgcaggtagtagc-------
```

IGLV2-33

```
V2-33_01      atggcctgggctctgctcctcctcaccctcctcactcagggcacagggtcctgggcccaatctgccctgactcagcctccttttgtgtccg
V2-33_02      atggcctgggctctgctcctcctcaccctcctcactcagggcacagggtcctgggcccaatctgccctgactcagcctccttttgtgtctg
V2-33_03      gtggcctgggctctgctcctcctcaccctcctcactcagggcacagggtcctgggcccaatctgccctgactcagcctccttttgtgtccg
IGLV2-33*02   atggcctgggctctgctgctcctcactctcctcactcaggacacagggtcctgggcccagtctgccctgactcagcctccttttgtgtccg
IGLV2-33*03   atggcctgggctctgctgctcctcaccctcctcactcagggcacagggtcctgggcccaatctgccctgactcagcctccttttgtgtccg
IGLV2-33*01   ---------------------------------------------------------caatctgccctgactcagcctccttttgtgtccg
V_V2-33*01    ---------------------------------------------------------caatctgccctgactcagcctccttttgtgtccg

V2-33_01      gggctcctggacagtcggtcaccatctcctgcactggaaccagcagtgacgttggggattatgatcatgtcttctggtaccaaaagcgt
V2-33_02      gggctcctggacagtcggtcaccatctcctgcactggaaccagcagtgacgttggggattatgatcatgtcttctggtaccaaaagcgt
V2-33_03      gggctcctggacagtcggtcaccatctcctgcactggaaccagcagtgacgttggggattatgatcatgtcttctggtaccaaaagcgt
IGLV2-33*02   gggctcctggacagtcggtcaccatctcctgcactggaaccagcagtgacgttggggattatgatcatgtcttctggtaccaaaagcgt
IGLV2-33*03   gggctcctggacagtcggtcaccatctcctgcactggaaccagcagtgacgttggggactatgatcatgtcttctggtaccaaaagcgt
IGLV2-33*01   gggctcctggacagtcggtcaccatctcctgcactggaaccagcagtgacgttggggattatgatcatgtcttctggtaccaaaagcgt
V_V2-33*01    gggctcctggacagtcggtcaccatctcctgcactggaaccagcagtgacgttggggattatgatcatgtcttctggtaccaaaagcgt

V2-33_01      ctcagcactacctccagactcctgatttacaatgtcaatactcggccttcagggatctctgacctcttctcaggctccaagtctggcaacatg
V2-33_02      ctcagcactacctccagactcctgatttacaatgtcaatactcggccttcagggatctctgacctcttctcaggctccaagtctggcaacatg
V2-33_03      ctcagcactacctccagactcctgatttacaatgtcaatactcggccttcagggatctctgacctcttctcaggctccaagtctggcaacatg
IGLV2-33*02   ctcagcactacctccagactcctgatttacaatgtcaatactcggccttcagggatctctgacctcttctcaggctccaagtctggcaacatg
IGLV2-33*03   ctcagcactacctccagactcctgatttacaatgtcaatactcggccttcagggatctctgacctcttctcaggctccaagtctggcaacgtg
IGLV2-33*01   ctcagcactacctccagactcctgatttacaatgtcaatactcggccttcagggatctctgacctcttctcaggctccaagtctggcaacatg
V_V2-33*01    ctcagcactacctccagactcctgatttacaatgtcaatactcggccttcagggatctctgacctcttctcaggctccaagtctggcaacatg

V2-33_01      gcttccctgaccatctctgggctcaagtccgaggttgaggctaattatcactgcagcttatattcaa--------------
V2-33_02      gcttccctgaccatctctgggctcaagtccgaggttgaggctaattatcactgcagcttatattcaa--------------
V2-33_03      gcttccctgaccatctctgggctcaagtccgaggttgaggctaattatcactgcagcttatattcaa--------------
IGLV2-33*02   gcttccctgaccatctctgggctcaagtccgaggttgaggctaattatcactgcagcttatattcaagtagttacactttc
IGLV2-33*03   gcttccctgaccatctctgggctcaagtccgaggttgaggctaattatcactgcagcttatattcaagtagttacactttc
IGLV2-33*01   gcttccctgaccatctctgggctcaagtccgaggttgaggctaattatcactgcagcttatattcaagtagttacactttc
V_V2-33*01    gcttccctgaccatctctgggctcaagtccgaggttgaggctaattatcactgcagcttatattcaagtagttac------
```

IGLV3-1

```
V3-1_01        atggcatggatccctctcttcctcggcgtccttgcttactgcacaggatccgtggcctcctatgagctgactcagccaccctcagtgtccg
V3-1_02        atggcatggatccctctcttcctcggcgtccttgcttactgcacaggttccgtggcctcctatgagctgactcagccaccctcagtgtccg
IGLV3-1*01     atggcatggatccctctcttcctcggcgtccttgcttactgcacaggatccgtggcctcctatgagctgactcagccaccctcagtgtccg
IP_V3-1*p02    ---------------------------------------------------------tcctatgagctgactcagccaccctcagtgtccg
IP_V3-1*p03    ---------------------------------------------------------tcctatgagctgactcagccaccctcagtgtccg
IP_V3-1*p02    ---------------------------------------------------------tcctatgagctgactcagccaccctcagtgtccg
IP_V3-1*p03    ---------------------------------------------------------tcctatgagctgactcagccaccctcagtgtccg
V_V3-1*01      ---------------------------------------------------------tcctatgagctgactcagccaccctcagtgtccg

V3-1_01        tgtccccaggacagacagccagcatcacctgctctggagataaattggggggataaatatgcttgctggtatcagcagaagccaggccag
V3-1_02        tgtccccaggacagacagccagcatcacctgctctggagataaattggggggataaatatgcttgctggtatcagcagaagccaggccag
IGLV3-1*01     tgtccccaggacagacagccagcatcacctgctctggagataaattggggggataaatatgcttgctggtatcagcagaagccaggccag
IP_V3-1*p02    tgtccccaggacagacagccagcatcacctgctctggagataaattggggggataaatatgcttgctggtatcagcagaagccaggccag
IP_V3-1*p03    tgtccccaggacagacagccagcatcacctgctctggagataaattggggggataaatatgcttgctggtatcagcagaagccaggccag
IP_V3-1*p02    tgtccccaggacagacagccagcatcacctgctctggagataaattggggggataaatatgcttgctggtatcagcagaagccaggccag
IP_V3-1*p03    tgtccccaggacagacagccagcatcacctgctctggagataaattggggggataaatatgcttgctggtatcagcagaagccaggccag
V_V3-1*01      tgtccccaggacagacagccagcatcacctgctctggagataaattggggggataaatatgcttgctggtatcagcagaagccaggccag

V3-1_01        tcccctgtgctggtcatctatcaagatagcaagcggccctcagggatccctgagcgattctctggctccaactctgggaacacagccact
V3-1_02        tcccctgtgctggtcatctatcaagatagcaagcggccctcagggatccctgagcgattctctggctccaactctgggaacacagccact
IGLV3-1*01     tcccctgtgctggtcatctatcaagatagcaagcggccctcagggatccctgagcgattctctggctccaactctgggaacacagccact
IP_V3-1*p02    tcccctgtgctggtcatctatcaagatagcaagcggccctcagggatccctgagcgattctctggctccaactctgggaacacagccact
IP_V3-1*p03    tcccctgtgctggtcatctatcaagataacaagcggccctcagggatccctgagcgattctctggctccaactctgggaacacagccact
IP_V3-1*p02    tcccctgtgctggtcatctatcaagatagcaagcggccctcagggatccctgagcgattctctggctccaactctgggaacacagccact
IP_V3-1*p03    tcccctgtgctggtcatctatcaagataacaagcggccctcagggatccctgagcgattctctggctccaactctgggaacacagccact
V_V3-1*01      tcccctgtgctggtcatctatcaagatagcaagcggccctcagggatccctgagcgattctctggctccaactctgggaacacagccact

V3-1_01        ctgaccatcagcgggacccaggctatggatgaggctgactattactgtcaggcgtgggacagcagcaactgcacaca
V3-1_02        ctgaccatcagcgggacccaggctatggatgaggctgactattactgtcaggcgtgggacagcagcaactgcacaca
IGLV3-1*01     ctgaccatcagcgggacccaggctatggatgaggctgactattactgtcaggcgtgggacagcagcaactgca----
IP_V3-1*p02    ctgaccatcagcgggacccaggctatggatgaggctgactattactgtcaggcgtgggacagcagctctgca----
IP_V3-1*p03    ctgaccatcagcgggacccaggctatggatgaggctgactattactgtcaggcgtgggacagcagcaactgca----
IP_V3-1*p02    ctgaccatcagcgggacccaggctatggatgaggctgactattactgtcaggcgtgggacagcagctctgca----
IP_V3-1*p03    ctgaccatcagcgggacccaggctatggatgaggctgactattactgtcaggcgtgggacagcagcaactgca----
V_V3-1*01      ctgaccatcagcgggacccaggctatggatgaggctgactattactgtcaggcgtgggacagcagcaact-------
```

IGLV3-9

```
V3-9_01        atggcctggaccgctctccttctgagcctccttgctcactttacaggttctgtggcctcctatgagctgactcagccactctcagtgtca
V3-9_02        atggcctggaccgctctccttctgagcctccttgctcactttacaggttctgtggcctcctatgagctgactcagccactctcagtgtca
IGLV3-9*02     atggcctggaccgctctccttctgagcctccttgctcactttacaggttctgtggcctcctatgagctgactcagccactctcagtgtca
IGLV3-9*03     atggcctggaccgctctccttctgagcctccttgctcactttacaggttctgtggcctcctatgagctgactcagccactctcagtgtca
IGLV3-9*01     ---------------------------------------------------------tcctatgagctgactcagccactctcagtgtca
V_V3-9*02      ---------------------------------------------------------tcctatgagctgactcagccactctcagtgtca
V_V3-9*01      ---------------------------------------------------------tcctatgagctgactcagccactctcagtgtca

V3-9_01        gtggccctgggacagacggccaggattacctgtggggaaacaacattggaagtaaaaatgtgcactggtaccagcagaagccaggccag
V3-9_02        gtggccctgggacagacggccaggattacctgtggggaaacaacattggaagtaaaaatgtgcactggtaccagcagaagccaggccag
IGLV3-9*02     gtggccctgggacaggcggccaggattacctgtggggaaacaaccttggatataaaagtgtgcactggtaccagcagaagccaggccag
IGLV3-9*03     gtggccctgggacaggcggccaggattacctgtggggaaacaaccttggatataaaa-tgtgcactggtaccagcagaagccaggccag
IGLV3-9*01     gtggccctgggacagacggccaggattacctgtggggaaacaacattggaagtaaaaatgtgcactggtaccagcagaagccaggccag
V_V3-9*02      gtggccctgggacaggcggccaggattacctgtggggaaacaaccttggatataaaagtgtgcactggtaccagcagaagccaggccag
V_V3-9*01      gtggccctgggacagacggccaggattacctgtggggaaacaacattggaagtaaaaatgtgcactggtaccagcagaagccaggccag

V3-9_01        gcccctgtgctggtcatctatagggatagcaaccggccctctgggatccctgagcgattctctggctccaactcggggaacacggccaccct
V3-9_02        gcccctgtgctggtcatctatagggatagcaaccggccctctgggatccctgagcgattctctggctccaactcggggaacacggccaccct
IGLV3-9*02     gcccctgtgctggtcatctatagggataacaaccggccctctgggatccctgagcgattctctggctccaactcggggaacacggccaccct
IGLV3-9*03     gcccctgtgctggtcatctatagggataacaaccggccctctgggatccctgagcgattctctggctccaactcggggaacacggccaccct
IGLV3-9*01     gcccctgtgctggtcatctatagggatagcaaccggccctctgggatccctgagcgattctctggctccaactcggggaacacggccaccct
V_V3-9*02      gcccctgtgctggtcatctatagggataacaaccggccctctgggatccctgagcgattctctggctccaactcggggaacacggccaccct
V_V3-9*01      gcccctgtgctggtcatctatagggatagcaaccggccctctgggatccctgagcgattctctggctccaactcggggaacacggccaccct

V3-9_01        gaccatcagcagagcccaagccggggatgaggctgactattactgtcaggtgtgggacagcagcactgcacaca
V3-9_02        gaccatcagcagagcccaagccagggatgaggctgactattactgtcaggtgtgggacagcagcactgcacaca
IGLV3-9*02     gaccatcagcagagcccaagccggggatgaggctgactattactgtcaggtgtgggacagcagcactgcacacc
IGLV3-9*03     gaccatcagcagagcccaagccggggatgaggctgactattactgtcaggtgtgggacagcagcactgca----
IGLV3-9*01     gaccatcagcagagcccaagccggggatgaggctgactattactgtcaggtgtgggacagcagcactgca----
V_V3-9*02      gaccatcagcagagcccaagccggggatgaggctgactattactgtcaggtgtgggacagcagcact-------
V_V3-9*01      gaccatcagcagagcccaagccggggatgaggctgactattactgtcaggtgtgggacagcagcact-------
```

IGLV3-10

```
V3-10_01      atggcctggacccctctcctgctcccctcctcactttctgcacagtctctgaggcctcctatgagctgacacagccaccctcggtgtcag
V3-10_02      atggcctggacccctctcctgctcccctcctcactttctgcacagtctctgaggcctcctatgagctgacacagccaccctcggtgtcag
V3-10_03      atggcctggacccctctcctgctcccctcctcactttctgcacagtctctgaggcctcctatgagctgacacagccaccctcggtgtcag
V3-10_04      atggcctggacccctctcctgctcccctcctcactttctgcacagtctctgaggcctcctatgagctgacacagccaccctcagtgtcag
V3-10_05      atggcctggacccctctcctgctcccctcctcactttctgcacagtctctgaggcctcctatgagctgacacagccaccctcggtgtcag
IGLV3-10*02   atggcctggacccctctcctgctcccctcctcactttctgcacagtctctgaggcctcctatgagctgacacagccaccctcggtgtcag
IGLV3-10*01   ---------------------------------------------------------tcctatgagctgacacagccaccctcggtgtcag
V_V3-10*01    ---------------------------------------------------------tcctatgagctgacacagccaccctcggtgtcag

V3-10_01      tgtccccaggacaaacggccaggatcacctgctctggagatgcattgccaaaaaaatatgcttattggtaccagcagaagtcaggccag
V3-10_02      tgtccccaggacaaacggccaggatcacctgctctggagatgcattgccaaaaaaatatccttattggtaccagcagaagtcaggccag
V3-10_03      tgtccccaggacaaacggccaggatcacctgctctggagatgcattgccaaaaaaatatgcttattggtaccagcagaagtcaggccag
V3-10_04      tgtccccaggacaaacggccaggatcacatgctctggagatgcattgccaaaaaaatatgcttattggtaccagcagaagtcaggccag
V3-10_05      tgtccccaggacaaacggccaggatcacctgctctggagatgcattgccaaaaaaatatgcttattggtaccagcagaagtcaggccag
IGLV3-10*02   tgtccccaggacaaacggccaggatcacctgctctggagatgcattgccaaaaaaatatgcttattggtaccagcagaagtcaggccag
IGLV3-10*01   tgtccccaggacaaacggccaggatcacctgctctggagatgcattgccaaaaaaatatgcttattggtaccagcagaagtcaggccag
V_V3-10*01    tgtccccaggacaaacggccaggatcacctgctctggagatgcattgccaaaaaaatatgcttattggtaccagcagaagtcaggccag

V3-10_01      gcccctgtgctggtcatctatgaggacagcaaacgaccctccgggatccctgagagattctctggctccagctcagggacaatggccacct
V3-10_02      gcccctgtgctggtcatctatgaggacagcaaacgaccctccgggatccctgagagattctctggctccagctcagggacaatggccacct
V3-10_03      gcccctgtgctggtcatctatgaggacagcaaacgaccctccgggatccctgagagattctctggctccagctcagggacaatggccacct
V3-10_04      gcccctgtgctggtcatctatgaggacagcaaacgaccctccgggatccctgagagattctctggctccagctcagggacaatggccacct
V3-10_05      gcccctgtgctggtcatctatgaggacagcaaacaaccctccgggatccctgagagattctctggctccagctcagggacaatggccacct
IGLV3-10*02   gcccctgtgctggtcatctataaggacagcaaacgaccctcagggatccctgagagattctctggctccagctcagggacaatggccacct
IGLV3-10*01   gcccctgtgctggtcatctatgaggacagcaaacgaccctccgggatccctgagagattctctggctccagctcagggacaatggccacct
V_V3-10*01    gcccctgtgctggtcatctatgaggacagcaaacgaccctccgggatccctgagagattctctggctccagctcagggacaatggccacct

V3-10_01      tgactatcagtggggcccaggtggaggatgaagctgactactactgttactcaacagacagcagtggtaatcatagcaca
V3-10_02      tgactatcagtggggcccaggtggaggatgaagctgactactactgttactcaacagacagcagtggtaatcatagcaca
V3-10_03      tgactatcagtggggcccaggtggaggatgaagatgactactactgttactcaacagacagcagtggtaatcatagcaca
V3-10_04      tgactatcagtggggcccaggtggaggatgaagctgactactactgttactcaacagacagcagtggtaatcatagcaca
V3-10_05      tgactatcagtggggcccaggtggaggatgaagctgactactactgttactcaacagacagcagtggtaatcatagcaca
IGLV3-10*02   tgaccatcagtggggcccaggtggaggatgaagatgactactactgttactcagcagactacagtggtaat---------
IGLV3-10*01   tgactatcagtggggcccaggtggaggatgaagctgactactactgttactcaacagacagcagtggtaatcatag----
V_V3-10*01    tgactatcagtggggcccaggtggaggatgaagctgactactactgttactcaacagacagcagtggt------------
```

IGLV3-12

```
V3-12_01      acggcctggacccctctcctcctcggcctcctcgctcactgcacaggctctgcgacctcctatgagctgactcagccacactcagtgtcagtgg
V3-12_02      atggcctggacccctctcctcctcagcctcctcgctcactgcacaggctctgcgacctcctatgagctgactcagccacactcagtgtcagtgg
V3-12_03      acggcctggacccctctcctcctcggcctcctcgctcactgcacaggctctgcgacctcctatgagctgactcagccacactcagtgtcagtgg
V3-12_04      acggcctggacccctctcctcctcggcctcctcgctcactgcacaggctctgcgacctcctatgagctgactcagccacactcagtgtcagtgg
V3-12_05      acggcctggacccctctcctcctcggcctcctcgctcactgcacaggctctgcgacctcctatgagctgactcagccacactcagtgtcagtgg
IGLV3-12*02   atggcctggacccctctcctcctcagcctcctcgctcactgcacaggctctgcgacctcctatgagctgactcagccacactcagtgtcagtgg
IGLV3-12*01   ---------------------------------------------------------tcctatgagctgactcagccacactcagtgtcagtgg
V_V3-12*02    ---------------------------------------------------------tcctatgagctgactcagccacactcagtgtcagtgg

V3-12_01      ccacagcacagatggccaggatcacctgtggggggaaacaacattggaagtaaagctgtgcactggtaccagcaaaagccaggccag
V3-12_02      ccacagcacagatggccaggatcacctgtggggggaaacaacattggaagtaaagctgtgcactggtaccagcaaaagccaggccag
V3-12_03      ccacagcacagatggccaggatcacctgtggggggaaacaacattggaagtaaagctgtgcactggtaccagcaaaagccaggccag
V3-12_04      ccacagcacagatggccaggatcacctgtggggggaaacaacattggaagtaaagctgtgcactggtaccagcaaaagccaggccag
V3-12_05      ccacagcacagatggccaggatcacctgtggggggaaacaacattggaagtaaagctgtgcactggtaccagcaaaagccaggccag
IGLV3-12*02   ccacagcacagatggccaggatcacctgtggggggaaacaacattggaagtaaagctgtgcactggtaccagcaaaagccaggccag
IGLV3-12*01   ccacagcacagatggccaggatcacctgtggggggaaacaacattggaagtaaagctgtgcactggtaccagcaaaagccaggccag
V_V3-12*02    ccacagcacagatggccaggatcacctgtggggggaaacaacattggaagtaaagctgtgcactggtaccagcaaaagccaggccag

V3-12_01      gaccctgtgctggtcatctatagcgatagcaaccggccctcagggatccctgagcgattctctggctccaacccagggaacaccaccaccc
V3-12_02      gaccctgtgctggtcatctatagcgatagcaaccggccctcagggatccctgagcgattctctggctccaacccagggaacaccgccaccc
V3-12_03      gaccctgtgctggtcatctatagcgatagaaaccggccctcagggatccctgagcgattctctggctccaacccagggaacaccgccaccc
V3-12_04      gaccctgtgctggtcatctatagcgatagcaaccggccctcagggatccctgagcgattctctggctccaacccagggaacaccgccaccc
V3-12_05      gaccctgtgctggtcatctatagcgatagcaaccggccctcagggatccctgagcgattctctggctccaacccagggaacaccaccaccc
IGLV3-12*02   gaccctgtgctggtcatctatagcgatagcaaccggccctcagggatccctgagcgattctctggctccaacccagggaacaccgccaccc
IGLV3-12*01   gaccctgtgctggtcatctatagcgatagcaaccggccctcagggatccctgagcgattctctggctccaacccagggaacaccaccaccc
V_V3-12*02    gaccctgtgctggtcatctatagcgatagcaaccggccctcagggatccctgagcgattctctggctccaacccagggaacaccgccaccc

V3-12_01      taaccatcagcaggatcgaggctggggatgaggctgactattactgtcaggtgtgggacagtagtagtgatcatcccacg
V3-12_02      taaccatcagcaggatcgaggctggggatgaggctgactattactgtcaggtgtgggacagtagtagtgatcatcccacg
V3-12_03      tgaccatcagcaggatcgaggctggggatgaggctgactattactgtcaggtgtgggacagtagtagtgatcatcccacg
V3-12_04      taaccatcagcaggatcgaggctggggatgaggctgactattactgtcaggtgtgggacagtagtagtgatcatcccacg
V3-12_05      taactatcagcaggatcgaggctggggatgaggctgactattactgtcaggtgtgggacagtagtagtgatcatcccacg
IGLV3-12*02   taaccatcagcaggatcgaggctggggatgaggctgactattactgtcaggtgtgggacagtagtagtgatcatcc----
IGLV3-12*01   taaccatcagcaggatcgaggctggggatgaggctgactattactgtcaggtgtgggacagtagtagtgatcatcc----
V_V3-12*02    taaccatcagcaggatcgaggctggggatgaggctgactattactgtcaggtgtgggacagtagtagt------------
```

IGLV3-16

```
V3-16_01      atggcctggatccctctcctgctcccctcctca--ctctctgcacaggctctgaggcctcctatgagctgacacagccaccctcggtgtcag
V3-16_02      atggcctggatccctctcctgctcccctcctcactctctctgcacaggctctgaggcctcctatgagctgacacagccaccctcggtgtcag
V3-16_03      atggcctggatccctctcctgctcccctcctcactctctctgcacaggctctgaggcctcctatgagctgacacagccaccctcggtgtcag
V3-16_04      atggcctggatccctctcctgctcccctcctca--ctctctgcacaggctctgaggcctcctatgagctgacacagccaccctcggtgtcag
V3-16_05      atggcctggatccctctcctgctcccctcctcactctctctgcacaggctctgaggcctcctatgagctgacacagccaccctcggtgtcag
V3-16_06      atggcctggatccctctcctgctcccctcctcactctctctgcacaggctctgaggcctcctatgagctgacacagccaccctcggtgtcag
IGLV3-16*01   -------------------------------------------------------------tcctatgagctgacacagccaccctcggtgtcag
V_V3-16*01    -------------------------------------------------------------tcctatgagctgacacagccaccctcggtgtcag

V3-16_01      tgtccctaggacagatggccaggatcacctgctctggagaagcattgccaaaaaaatatgcttattggtaccagcagaagccaggcc
V3-16_02      tgtccctaggacagatggccaggatcacctgctctggagaagcattgccaaaaaaatatgcttattggtaccagcagaagccaggcc
V3-16_03      tgtccctaggacagatggccaggatcacctgctctggagaagcattgccaaaaaaatatgcttattggtaccagcagaagccaggcc
V3-16_04      tgtccctaggacagatggccaggatcacctgctctggagaagcattgccaaaaaaatatgcttattggtaccagcagaagccaggcc
V3-16_05      tgtccctaggacagatggccaggatcacctgctctggagaagcattgccaaaaaaatatgcttattggtaccagcagaagccaggcc
V3-16_06      tgtccctaggacagatggccaggatcacctgctctggagaagcattgccaaaaaaatatgcttattggtaccagcagaagccaggcc
IGLV3-16*01   tgtccctaggacagatggccaggatcacctgctctggagaagcattgccaaaaaaatatgcttattggtaccagcagaagccaggcc
V_V3-16*01    tgtccctaggacagatggccaggatcacctgctctggagaagcattgccaaaaaaatatgcttattggtaccagcagaagccaggcc

V3-16_01      agttccctgtgctggtgatatataaagacagcgagaggccctcagggatccctgagcgattctctggctccagctcagggacaatagtcacat
V3-16_02      agttccctgtgctggtgatatataaagacagcgagaggccctcagggatccctgagcgattctctggctccagctcagggacaatagtcacat
V3-16_03      aggcccctgtgctggtgatatataaagacagcgagaggccctcagggatccctgagcgattctctggctccagctcagggacaatagtcacat
V3-16_04      agttccctgtgctggtgatatataaagacagcgagaggccctcagggatccctgagcgattctctggctccagctcagggacaatagtcacat
V3-16_05      agttccctgtgctggtgatatataaagacagcgagaggccctcagggatccctgagcgattctctggctccagctcagggacaatagacacat
V3-16_06      agttccctgtgctggtgatatataaagacagcgagaggccctcagggatccctgagcgattctctggctccagctcagggacaatagtcacat
IGLV3-16*01   agttccctgtgctggtgatatataaagacagcgagaggccctcagggatccctgagcgattctctggctccagctcagggacaatagtcacat
V_V3-16*01    agttccctgtgctggtgatatataaagacagcgagaggccctcagggatccctgagcgattctctggctccagctcagggacaatagtcacat

V3-16_01      tgaccatcagtggagtccaggcagaagacgaggctgactattactgtctatcagcagacagcagtg
V3-16_02      tgaccatcagtggagtccaggcagaagacgaggctgactattactgtctatcagcagacagcagtg
V3-16_03      tgaccatcagtggagtccaggcagaagacgaggctgactattactgtctatcagcagacagcagtg
V3-16_04      tgaccatcagtggagtccaggcagaagacgaggctgactattactgtcaatcagcagacagcagtg
V3-16_05      tgaccatcagtggagtccaggcagaagacgaggctgactattactgtctatcagcagacagcagtg
V3-16_06      tgaccatcagtggagtccaggcagaagacgaggctgactattactgtcaatcagcagacagcagtg
IGLV3-16*01   tgaccatcagtggagtccaggcagaagacgaggctgactattactgtctatcagcagacagcagtg
V_V3-16*01    tgaccatcagtggagtccaggcagaagacgaggctgactattactgtctatcagcagacagcagtg
```

IGLV3-19

```
V3-19_01      atggcctggacccctctctggctcactctcctcactctttgcataggttctgtggtttcttctgagctgactcaggaccctgctgtgtctgtggc
V3-19_02      atggcctggacccctctctggctcactctcttcactctttgcataggttctgtggtttcttctgagctgactcaggaccctgctgtgtctgtggc
V3-19_03      atggcctggacccctctctggcttactctcctcactctttgcataggttctgtggtttcgtctgagctgactcaggaccctgctgtgtctgtggc
V3-19_04      atggcctggacccctctctggctcactctcctcactctttgcataggttctgtggtttcttctgagctgactcaggaccctgctgtgtctgtggc
IGLV3-19*01   atggcctggacccctctctggctcactctcctcactctttgcataggttctgtggtttcttctgagctgactcaggaccctgctgtgtctgtggc
V_V3-19*01    ----------------------------------------------------------tcttctgagctgactcaggaccctgctgtgtctgtggc

V3-19_01      cttgggacagacagtcaggatcacatgccaaggagacagcctcagaagctattatgcaagctggtaccagcagaagccaggacag
V3-19_02      cttgggacagacagtcaggatcacatgccaaggagacagcctcagaagctattatgcaagctggtaccagcagaagccaggacag
V3-19_03      cttgggacagacagtcaggatcacatgccaaggagacagcctcagaagctattatgcaagctggtaccagcagaagccaggacag
V3-19_04      cttgggacagacagtcaggatcacatgccaagaagacagcctcagaagctattatgcaagctggtaccagcagaagccaggacag
IGLV3-19*01   cttgggacagacagtcaggatcacatgccaaggagacagcctcagaagctattatgcaagctggtaccagcagaagccaggacag
V_V3-19*01    cttgggacagacagtcaggatcacatgccaaggagacagcctcagaagctattatgcaagctggtaccagcagaagccaggacag

V3-19_01      gcccctgtacttgtcatctatggtaaaaacaaccggccctcagggatcccagaccgattctctggctccagctcaggaaacacagcttccttga
V3-19_02      gcccctgtacttgtcatctatggtaaaaacaaccggccctcagggatcccagaccgattctctggctccagctcaggaaacacagcttccttga
V3-19_03      gcccctgtacgtgtcatctatggtaaaaacaaccggccctcagggatcccagaccgattctctggctccagctcaggaaacacagcttccttga
V3-19_04      gcccctgtacttgtcatctatggtaaaaacaaccggccctcagggatcccagaccgattctctggctccagctcaggaaacacagcttccttga
IGLV3-19*01   gcccctgtacttgtcatctatggtaaaaacaaccggccctcagggatcccagaccgattctctggctccagctcaggaaacacagcttccttga
V_V3-19*01    gcccctgtacttgtcatctatggtaaaaacaaccggccctcagggatcccagaccgattctctggctccagctcaggaaacacagcttccttga

V3-19_01      ccatcactggggctcaggcggaagatgaggctgactattactgtaactcccgggacagcagtg
V3-19_02      ccatcactggggctcaggcggaagatgaggctgactattactgtaactcccgggacagcagtg
V3-19_03      ccatcactggggctcaggcggaagatgaggctgactattactgtaactcctgggacagcagtg
V3-19_04      ccatcactggggctcaggcggaagatgaggctgactattactgtaactcccgggacagcagtg
IGLV3-19*01   ccatcactggggctcaggcggaagatgaggctgactattactgtaactcccgggacagcagtg
V_V3-19*01    ccatcactggggctcaggcggaagatgaggctgactattactgtaactcccgggacagcagtg
```

IGLV3-21

```
V3-21_01       atggcctggaccgttctcctcctcggcctcctctctcactgcacaggctctgtgacctcctatgtgctgactcagccaccctcggtgtca
V3-21_02       atggcctggacctttctcctcctcggcctcctctctcactgcacaggctctgtgacctcctatgtgctgactcagccaccctcggtgtca
V3-21_03       atggcctggaccgttctcctcctcagcctcctcgctcactgcacaggctctgtgacctcctatgtgctgactcagccaccctcggtgtca
V3-21_04       atggcctggaccgttctcctcctcagcctcctcgctcactgcacaggctctgtgacctcctatgtgctgactcagccaccctcggtgtca
V3-21_05       atggcctggacctttctcctcctcggcctcctctctcactgcacaggctctgtgacctcctatgtgctgactcagccaccctcggtgtca
V3-21_06       atggcctggactgttctcctcctcggcctcctctctcactgcacaggctctgtgacctcctatgtgctgactcagccaccctcggtgtca
IGLV3-21*01    atggcctggaccgttctcctcctcggcctcctctctcactgcacaggctctgtgacctcctatgtgctgactcagccaccctcagtgtca
IGLV3-21*02    atggcctggaccgttctcctcctcggcctcctctctcactgcacaggctctgtgacctcctatgtgctgactcagccaccctcggtgtca
IGLV3-21*03    atggcctggaccgttctcctcctcggcctcctctctcactgcacaggctctgtgacctcctatgtgctgactcagccaccctcggtgtca
IP_V3-21*p04   ---------------------------------------------------------tcctatgtgctgactcagccaccctcagtgtca
IP_V3-21*p04   ---------------------------------------------------------tcctatgtgctgactcagccaccctcagtgtca
V_V3-21*01     ---------------------------------------------------------tcctatgtgctgactcagccaccctcagtgtca
V_V3-21*02     ---------------------------------------------------------tcctatgtgctgactcagccaccctcggtgtca
V_V3-21*03     ---------------------------------------------------------tcctatgtgctgactcagccaccctcggtgtca

V3-21_01       gtggccccaggacagacggccaggattacctgtggggaaacaacattggaagtaaaagtgtgcactggtaccagcagaagccaggccag
V3-21_02       gtggccccaggacagacggccaggattacctgtggggaaacaacattggaagtaaaagtgtgcactggtaccagcagaagccaggccag
V3-21_03       gtggccccaggacagacggccaggattacctgtggggaaacaacattggaagtaaaagtgtgcactggtaccagcagaagccaggccag
V3-21_04       gtggccccaggacagacggccaggattacctgtggggaaacaacattggaagtaaaagtgtgcactggtaccagcagaagccaggccag
V3-21_05       gtggccccaggacagacggccaggattacctgtggggaaacaacattggaagtaaaagtgtgcactggtaccagcagaagccaggccag
V3-21_06       gtggccccaggacagacggccaggattacctgtggggaaacaacattggaagtaaaagtgtgcactggtaccagcagaagccaggccag
IGLV3-21*01    gtggccccaggaaagacggccaggattacctgtggggaaacaacattggaagtaaaagtgtgcactggtaccagcagaagccaggccag
IGLV3-21*02    gtggccccaggacagacggccaggattacctgtggggaaacaacattggaagtaaaagtgtgcactggtaccagcagaagccaggccag
IGLV3-21*03    gtggccccaggaaagacggccaggattacctgtggggaaacaacattggaagtaaaagtgtgcactggtaccagcagaagccaggccag
IP_V3-21*p04   gtggccccaggaaagacggccaggattacctgtggggaaacaacattggaagtaaaagtgtgcactggtaccagcagaagccaggccag
IP_V3-21*p04   gtggccccaggaaagacggccaggattacctgtggggaaacaacattggaagtaaaagtgtgcactggtaccagcagaagccaggccag
V_V3-21*01     gtggccccaggaaagacggccaggattacctgtggggaaacaacattggaagtaaaagtgtgcactggtaccagcagaagccaggccag
V_V3-21*02     gtggccccaggacagacggccaggattacctgtggggaaacaacattggaagtaaaagtgtgcactggtaccagcagaagccaggccag
V_V3-21*03     gtggccccaggaaagacggccaggattacctgtggggaaacaacattggaagtaaaagtgtgcactggtaccagcagaagccaggccag
```

```
V3-21_01        gcccctgtgctggtcgtctatgatgatagcgaccggccctcagggatccctgagcgattctctggctccaactctgggaacacggccac
V3-21_02        gcccctgtgctggtcgtctatgatgatagcgaccggccctcagggatccctgagcgattctctggctccaactctgggaacacggccac
V3-21_03        gcccctgtgctggtcgtctatgatgatagcgaccggccctcagggatccctgagcgattctctggctccaactctgggaacacggccac
V3-21_04        gcccctgtgctggtcgtctatgatgatagcgaccggccctcagggatccctgagcgattctctggctccaactctgggaacacggccac
V3-21_05        gcccctgtgctggtcgtctatgatgatagcgaccggccctcagggatccctgagcgattctctggctccaactctgggaacacggccac
V3-21_06        gcccctgtgctggtcgtctatgatgatagcgaccggccctcagggatccctgagcgattctctggctccaactctgggaacacggccac
IGLV3-21*01     gcccctgtgctggtcatctattatgatagcgaccggccctcagggatccctgagcgattctctggctccaactctgggaacacggccac
IGLV3-21*02     gcccctgtgctggtcgtctatgatgatagcgaccggccctcagggatccctgagcgattctctggctccaactctgggaacacggccac
IGLV3-21*03     gcccctgtgctggtcgtctatgatgatagcgaccggccctcagggatccctgagcgattctctggctccaactctgggaacacggccac
IP_V3-21*p04    gcccctgtgctggtcgtctatgatgatagcgaccggccctcagggatccctgagcgattctctggctccaactctgggaacacggccac
IP_V3-21*p04    gcccctgtgctggtcgtctatgatgatagcgaccggccctcagggatccctgagcgattctctggctccaactctgggaacacggccac
V_V3-21*01      gcccctgtgctggtcatctattatgatagcgaccggccctcagggatccctgagcgattctctggctccaactctgggaacacggccac
V_V3-21*02      gcccctgtgctggtcgtctatgatgatagcgaccggccctcagggatccctgagcgattctctggctccaactctgggaacacggccac
V_V3-21*03      gcccctgtgctggtcgtctatgatgatagcgaccggccctcagggatccctgagcgattctctggctccaactctgggaacacggccac

V3-21_01        cctgaccatcagcagggtcgaagccggggatgaggccgactattactgtcaggtgtgggatagtagtagtgatcatcccacg
V3-21_02        cctgaccatcagcagggtcgaagccggggatgaggccgactattactgtcaggtgtgggatagtagtagtgatcatcccacg
V3-21_03        cctgaccatcagcagggtcgaagccggggatgaggccgactattactgtcaggtgtgggatagtagtagtgatcatcccacg
V3-21_04        cctgaccatcagcagggtcgaagccggggatgaggccgactattactgtcaggtgtgggatagtagtagtgatcatcccacg
V3-21_05        cctgaccatcagcagggtcgaagccggggatgaggccgactattactgtcaggtgtgggatagtagtagtgatcatcccacg
V3-21_06        cctgaccatcagcagggtcgaagccggggatgaggccgactattactgtcaggtgtgggatagtagtagtgatcatcccacg
IGLV3-21*01     cctgaccatcagcagggtcgaagccggggatgaggccgactattactgtcaggtgtgggacagtagtagtgatcatcc----
IGLV3-21*02     cctgaccatcagcagggtcgaagccggggatgaggccgactattactgtcaggtgtgggatagtagtagtgatcatcc----
IGLV3-21*03     cctgaccatcagcagggtcgaagccggggatgaggccgactattactgtcaggtgtgggatagtagtagtgatcatcc----
IP_V3-21*p04    cctgaccatcagcagggtcgaagccggggatgaggccgactattactgtcaggtgtgggatagtagtagtgatcatcc----
IP_V3-21*p04    cctgaccatcagcagggtcgaagccggggatgaggccgactattactgtcaggtgtgggatagtagtagtgatcatcc----
V_V3-21*01      cctgaccatcagcagggtcgaagccggggatgaggccgactattactgtcaggtgtgggatagtagtagt------------
V_V3-21*02      cctgaccatcagcagggtcgaagccggggatgaggccgactattactgtcaggtgtgggatagtagtagt------------
V_V3-21*03      cctgaccatcagcagggtcgaagccggggatgaggccgactattactgtcaggtgtgggatagtagtagt------------
```

IGLV3-25

```
V3-25_01        atggcctggatccctctacttctccccctcttcactctctgcacaggctctgaggcctcctatgagctgacacagccaccctcggtgtcagt
V3-25_02        atggcctggatccctctacttctcccccctccactctctgcacaggctctgaggcctcctatgagctgacacagccaccctcggtgtcagt
V3-25_03        atggcctggatccctctacttctcccccctccactctctgcacaggctctgaggcctcctatgagctgacacagccaccctcggtgtcagt
V3-25_04        atggcctggatccctctacttctccccctcttcactctctgcacaggctctgaggcctcctatgagctgacacagccaccctcggtgtcagt
V3-25_05        atggcctggatccctctacttctccacctcctcactctctgcacaggctctgaggcctcctatgagctgacacagccaccctcggtgtcagt
IGLV3-25*02     atggcctggatccctctacttctccccctcttcactctctgcacaggctctgaggcctcctatgagctgacacagccaccctcggtgtcagt
IGLV3-25*03     atggcctggatccctctacttctcccccctccactctctgcacaggctctgaggcctcctatgagctgacacagccaccctcggtgtcagt
IGLV3-25*01     ---------------------------------------------------------tcctatgagctgatgcagccaccctcggtgtcagt
IP_V3-25*p04    ---------------------------------------------------------tcctatgagctgacacagccaccctcggtgtcagt
IP_V3-25*p04    ---------------------------------------------------------tcctatgagctgacacagccaccctcggtgtcagt
V_V3-25*02      ---------------------------------------------------------tcctatgagctgacacagccaccctcggtgtcagt
V_V3-25*03      ---------------------------------------------------------tcctatgagctgacacagccaccctcggtgtcagt

V3-25_01        gtccccaggacagacggccaggatcacctgctctggagatgcattgccaaagcaatatgcttattggtaccagcagaagccaggccag
V3-25_02        gtccccaggacagacggccaggatcacctgctctggagatgcattgccaaagcaatatgcttattggtaccagcagaagccaggccag
V3-25_03        gtccccaggacagacggccaggatcacctgctctgcagatgcattgccaaagcaatatgcttattggtaccagcagaagccaggccag
V3-25_04        gtccccaggacagacggccaggatcacctgctctggagatgcattgccaaagcaatatgcttattggtaccagcagaagccaggccag
V3-25_05        gtccccaggacagacggccaggatcacctgctctggagatgcattgccaaagcaatatgcttattggtaccagcagaagccaggccag
IGLV3-25*02     gtccccaggacagacggccaggatcacctgctctggagatgcattgccaaagcaatatgcttattggtaccagcagaagccaggccag
IGLV3-25*03     gtccccaggacagacggccaggatcacctgctctggagatgcattgccaaagcaatatgcttattggtaccagcagaagccaggccag
IGLV3-25*01     gtccccaggacagacggccaggatcacctgctctggagatgcattgccaaagcaatatgcttattggtaccagcagaagccaggccag
IP_V3-25*p04    gtccccaggacagacggccaggatcacctgctctggagatgcattgccaaagcaatatgcttattggtaccagcagaagccaggccag
IP_V3-25*p04    gtccccaggacagacggccaggatcacctgctctggagatgcattgccaaagcaatatgcttattggtaccagcagaagccaggccag
V_V3-25*02      gtccccaggacagacggccaggatcacctgctctggagatgcattgccaaagcaatatgcttattggtaccagcagaagccaggccag
V_V3-25*03      gtccccaggacagacggccaggatcacctgctctggagatgcattgccaaagcaatatgcttattggtaccagcagaagccaggccag

V3-25_01        gcccctgtgctggtgatatataaagacagtgagaggccctcagggatccctgagcgattctctggctccagctcagggacaacagtcac
V3-25_02        gcccctgtgctggtgatatataaagacagtgagaggccctcagggatccctgagcgattctctggctccagctcagggacaacagtcac
V3-25_03        gcccctgtgctggtgatatataaagacagtgagaggccctcagggatccctgagcgattctctggctccagctcagggacaacagtcac
V3-25_04        gcccctgtgctggtgatatataaagacagtgagaggccctcagggatccctgagcgattctctggctccagctcagggacaacagtcac
V3-25_05        gcccctgtgctggtgatatataaagacagtgagaggccctcagggatccctgagcgattctctggctccagctcagggacaacagtcac
IGLV3-25*02     gcccctgtgctggtgatatataaagacagtgagaggccctcagggatccctgagcgattctctggctccagctcagggacaacagtcac
IGLV3-25*03     gcccctgtgctggtgatatataaagacagtgagaggccctcagggatccctgagcgattctctggctccagctcagggacaacagtcac
IGLV3-25*01     gcccctgtgctggtgatatataaagacagtgagaggccctcagggatccctgagcgattctctggctccagctcagggacaacagtcac
IP_V3-25*p04    gcccctgtgctggtgatatataaagacagtgagaggccctcagggatccctgagcgattctctggctccagctcagggacaacagtcac
IP_V3-25*p04    gcccctgtgctggtgatatataaagacagtgagaggccctcagggatccctgagcgattctctggctccagctcagggacaacagtcac
V_V3-25*02      gcccctgtgctggtgatatataaagacagtgagaggccctcagggatccctgagcgattctctggctccagctcagggacaacagtcac
V_V3-25*03      gcccctgtgctggtgatatataaagacagtgagaggccctcagggatccctgagcgattctctggctccagctcagggacaacagtcac
```

```
V3-25_01        gttgaccatcagtggagtccaggcagaagatgaggctgactattactgtcaatcagcagacagcagtg
V3-25_02        gttgaccatcagtggagtccaggcagaagacgaggctgactattactgtcaatcagcagacagcagtg
V3-25_03        gttgaccatcagtggagtccaggcagaagacgaggctgactattactgtcaatcagcagacagcagtg
V3-25_04        gttgaccatcagtggagtccaggcagaagacgaggctgactattactgtcaatcagcagacagcagtg
V3-25_05        gttgaccatcagtggagtccaggcagaagacgaggctgactattactgtcaatcagcagacagcagtg
IGLV3-25*02     gttgaccatcagtggagtccaggcagaagatgaggctgactattactgtcaatcagcagacagcagtg
IGLV3-25*03     gttgaccatcagtggagtccaggcagaagacgaggctgactattactgtcaatcagcagacagcagtg
IGLV3-25*01     gttgaccatcagtggagtccaggcagaagatgaggctgactattactgtcaatcagcagacagcagtg
IP_V3-25*p04    gttgaccatcagtggagtccaggcagaagacgaggctgactattactgtcaatcagcagacagcagtg
IP_V3-25*p04    gttgaccatcagtggagtccaggcagaagacgaggctgactattactgtcaatcagcagacagcagtg
V_V3-25*02      gttgaccatcagtggagtccaggcagaagatgaggctgactattactgtcaatcagcagacagcagtg
V_V3-25*03      gttgaccatcagtggagtccaggcagaagacgaggctgactattactgtcaatcagcagacagcagtg
```

Figure S3: Per gene alignment of alleles from pmIG, IMGT, IgPdb and VBASE2 databases for IGHV, IGKV and IGLV locus. The name of pmIG alleles are marked with grey background. Yellow background is used for the 104 IMGT alleles mentioned to be erroneous/false positives by Wang et al, 2008 [30].

IGLV3-27

```
V3-27_01       atggcctggatccctctcctgctcccctcctcattctctgcacagtctctgtggcctcctatgagctgacacagccatcctcagtgtcag
V3-27_02       atggcctggatccctctcctgctcccctcctcattctctgcacagtctctgtggcctcctatgagctgacacagccatcctcagtgtcag
IGLV3-27*01    atggcctggatccctctcctgctcccctcctcattctctgcacagtctctgtggcctcctatgagctgacacagccatcctcagtgtcag
V_V3-27*01     ----------------------------------------------------------tcctatgagctgacacagccatcctcagtgtcag

V3-27_01       tgtctccgggacagacagccaggatcacctgctcaggagatgtactggcaaaaaaatatgctcggtggttccagcagaagccaggccag
V3-27_02       tgtctccgggacagacagccaggatcacctgctcaggagatgtactggcaaaaaaatatgctcggtggttccagcagaagccaggccag
IGLV3-27*01    tgtctccgggacagacagccaggatcacctgctcaggagatgtactggcaaaaaaatatgctcggtggttccagcagaagccaggccag
V_V3-27*01     tgtctccgggacagacagccaggatcacctgctcaggagatgtactggcaaaaaaatatgctcggtggttccagcagaagccaggccag

V3-27_01       gcccctgtgctggtgatttataaagacagtgagcggccctcaggggatccctgagcgattctccggctccagctcagggaccacagtcaccttga
V3-27_02       gcccctgtgctggtgatttataaagacagtgagcggccctca-gggatccctgagcgattctccggctccagctcagggaccacagtcaccttga
IGLV3-27*01    gcccctgtgctggtgatttataaagacagtgagcggccctca-gggatccctgagcgattctccggctccagctcagggaccacagtcaccttga
V_V3-27*01     gcccctgtgctggtgatttataaagacagtgagcggccctca-gggatccctgagcgattctccggctccagctcagggaccacagtcaccttga

V3-27_01       ccatcagcggggcccaggttgaggatgaggctgactattactgttactctgcggctgacaacaat
V3-27_02       ccatcagcggggcccaggttgaggatgaggctgactattactgttactctgcggctgacaacaat
IGLV3-27*01    ccatcagcggggcccaggttgaggatgaggctgactattactgttactctgcggctgacaacaat
V_V3-27*01     ccatcagcggggcccaggttgaggatgaggctgactattactgttactctgcggctgacaacaat
```

IGLV3-32

```
V3-32_1         gctgtggactcagaggcagagctctggggcatttccattatggcctggacccctcccctgctcgtcctcactctctgcacaggctccgttatttcc
V3-32_2         gctgtggactcagaggcagagctctggggcatttccattatggcctagacccctcccctgctcgtcctcactctctgcacaggctccgttatttcc
V3-32_3         gctgtggactcagaggcagagctctggggcatttccattatggcctggacccctcccctgctcgtcctcactctctgcacaggctccgttatttcc
V3-32*01        ----------------------------------------------------------------------------------------------tcc
V_IGLV3-32*01   ----------------------------------------------------------------------------------------------tcc

V3-32_1         tctgggccaactcaggtgcctgcagtgtctgtggccttgggacaaatggccaggatcacctgccagggagacagcatggaaggc
V3-32_2         tctgggccaactcaggtgcctgcagtgtctgtggccttgggacaaatggccaggatcacctgccagggagacagcatggaaggc
V3-32_3         tctgggccaactcaggtgcctgcagtgtctgtggccttgggacaaatggccaggatcacctgccagggagacagcatggaaggc
IGLV3-32*01     tctgggccaactcaggtgcctgcagtgtctgtggccttgggacaaatggccaggatcacctgccagggagacagcatggaaggc
V_IGLV3-32*01   tctgggccaactcaggtgcctgcagtgtctgtggccttgggacaaatggccaggatcacctgccagggagacagcatggaaggc

V3-32_1         tcttatgaacactggtaccagcagaagccaggccaggcccccgtgctggtcatctatgatagcagtgaccggccctcaaggatccctgagcg
V3-32_2         tcttatgaacactggtaccagcagaagccaggccaggcccccgtgctggtcatctatgatagcagtgaccggccctcaaggatccctgagcg
V3-32_3         tcttatgaacactggtaccagcagaagccaggccaggcccccgtgctggtcatctatgatagcagtgaccagccctcaaggatccctgagcg
IGLV3-32*01     tcttatgaacactggtaccagcagaagccaggccaggcccccgtgctggtcatctatgatagcagtgaccggccctcaaggatccctgagcg
V_IGLV3-32*01   tcttatgaacactggtaccagcagaagccaggccaggcccccgtgctggtcatctatgatagcagtgaccggccctcaaggatccctgagcg

V3-32_1         attctctggctccaaatcaggcaacacaaccaccctgaccatcactggggcccaggctgaggatgaggctgattattactatcagt
V3-32_2         attctctggctccaaatcaggcaacacaaccaccctgaccatcactggggcccaggctgaggatgaggctgattattactatcagt
V3-32_3         attctctggctccaaatcaggcaacacaaccaccctgaccatcactggggcccaggctgaggatgaggctgattattactatcagt
IGLV3-32*01     attctctggctccaaatcaggcaacacaaccaccctgaccatcactggggcccaggctgaggatgaggctgattattactatcagt
V_IGLV3-32*01   attctctggctccaaatcaggcaacacaaccaccctgaccatcactggggcccaggctgaggatgaggctgattattactatcagt
```

IGLV4-3

```
V4-3_01        atggcctgggtctccttctacctactgcccttcattttctccacaggtctctgtgctctgcctgtgctgactcagcccccgtctgcatct
V4-3_02        atggcctgggtctccttctacctactgcccttcattttctccacaggtctctgtgctctgcctgtgctgactcagcccccgtctgcatct
V4-3_03        atggactgggtctccttctacctactgcccttcattttctccacaggtctctgtgctctgcctgtgctgactcagcccccgtctgcatct
IGLV4-3*01     atggcctgggtctccttctacctactgcccttcattttctccacaggtctctgtgctctgcctgtgctgactcagcccccgtctgcatct
V_V4-3*01      ---------------------------------------------------------ctgcctgtgctgactcagcccccgtctgcatct

V4-3_01        gccttgctgggagcctcgatcaagctcacctgcaccctaagcagtgagcacagcacctacaccatcgaatggtatcaacagagaccaggg
V4-3_02        gccttgctgggagcctcgatcaagctcacctgcaccctaagcagtgagcacagcacctacaccatcgaatggtatcaacagagaccaggg
V4-3_03        gccttgctgggagcctcgatcaagctcacctgcaccctaagcagtgagcacagcacctacaccatcgaatggtatcaacagagaccaggg
IGLV4-3*01     gccttgctgggagcctcgatcaagctcacctgcaccctaagcagtgagcacagcacctacaccatcgaatggtatcaacagagaccaggg
V_V4-3*01      gccttgctgggagcctcgatcaagctcacctgcaccctaagcagtgagcacagcacctacaccatcgaatggtatcaacagagaccaggg

V4-3_01        aggtcccccccagtatataatgaaggttaagagtgatggcagccacagcaaggggacgggatccccgatcgcttcatgggctccagttctggg
V4-3_02        aggtcccccccagtatataatgaaggttaagagtgatggcagccacagcaaggggacgggatccccgatcgcttcatgggctccagttctggg
V4-3_03        aggtcccccccagtatataatgaaggttaagagtgatggcagccacagcaaggggacgggatccccgatcgcttcatgggctccagttctggg
IGLV4-3*01     aggtcccccccagtatataatgaaggttaagagtgatggcagccacagcaaggggacgggatccccgatcgcttcatgggctccagttctggg
V_V4-3*01      aggtcccccccagtatataatgaaggttaagagtgatggcagccacagcaaggggacgggatccccgatcgcttcatgggctccagttctggg

V4-3_01        gctgaccgctacctcaccttctccaacctccagtctgacgatgaggctgagtatcactgtggagagagccacacgattgatggccaagtcggttgagc
V4-3_02        gctgaccgctacctcaccttctccaacctccagtctgacgatgaggctgagtatcactgtggagagagccacaagattgatggccaagtcggttgagc
V4-3_03        gctgaccgctacctcaccttctccaacctccagtctgacgatgaggctgagtatcactgtggagagagccacacgattgatggccaagtcggttgagc
IGLV4-3*01     gctgaccgctacctcaccttctccaacctccagtctgacgatgaggctgagtatcactgtggagagagccacacgattgatggccaagtcggttgagc
V_V4-3*01      gctgaccgctacctcaccttctccaacctccagtctgacgatgaggctgagtatcactgtggagagagccacacgattgat-----------------
```

IGLV4-60

```
V4-60_01      atggcctggaccccactcctcctcctcttccctctcctcctccactgcacaggg--tctctctcccagcctgtgctgactcaatcatcctc
V4-60_02      atggcctggaccccactcctcctcctcttccctctcctcctccactgcacaggg--tctctctcccagcctgtgctgactcaatcatcctc
V4-60_03      atggcctggaccccactcctcctcctcttccctctcctcctccactgcacaggg--tctctctcccagcctgtgctgactcaatcatcctc
V4-60_04      atggcctggaccccactcctcctcctcttccctctcctcctccactgcacaggg--tctctctcccagcctgtgctgactcaatcatcctc
V4-60_05      atggcctggaccccactcctcctcctcttccctctcctcctccactgcacaggg--tctctctcccagcctgtgctgactcaatcatcctc
V4-60_06      atggcctggaccccactcctcctcctcttccctctcctcctccactgcacagggtctctctctcccagcctgtgctgactcaatcatcctc
V4-60_07      atggcctggaccccactcctcctcctcttccctctcctcctccactgcacaggg--tctctctcccagcctgtgctgactcaatcatcctc
V4-60_08      atggcctggaccccactcctcctcctcttccctctcctcctccactgcacaggg--tctctctcccagcctgtgctgactcaatcatcctc
IGLV4-60*02   atggcctggaccccactcctcctcctcttccctctcctcctccactgcacaggg--tctctctcccagcctgtgctgactcaatcatcctc
IGLV4-60*01   -----------------------------------------------------------------cagcctgtgctgactcaatcatcctc
IGLV4-60*03   -----------------------------------------------------------------cagcctgtgctgactcaatcatcctc
V_V4-60*02    -----------------------------------------------------------------cagcctgtgctgactcaatcatcctc

V4-60_01      tgcctctgcttccctgggatcctcggtcaagctcacctgcactctgagcagtgggcacagtagctacatcatcgcatggcatccagcag
V4-60_02      tgcctctgcttccctgggatcctcggtcaagctcacctgcactctgagcagtgggcacagtagctacatcatcgcatggcatccagcag
V4-60_03      tgcctctgcttccctgggatcctcggtcaagctcacctgcactctgagcagtgggcacagtagctacatcatcgcatggcat-cagcag
V4-60_04      tgcctctgcttccctgggatcctcggtcaagctcacctgcactctgagcagtgggcacagtagctacatcatcgcatggcatccagcag
V4-60_05      tgcctctgcttccctgggatcctcggtcaagctcacctgcactctgagcagtgggcacagtagctacatcatcgcatggcatccagcag
V4-60_06      tgcctctgcttccctgggatcctcggtcaagctcacctgcactctgagcagtgggcacagtagctacatcatcgcatggcatccagcag
V4-60_07      tgcctctgcttccctgggatccttggtcaagctcacctgcactctgagcagtgggcacagtagctacatcatcgcatggcatccagcag
V4-60_08      tgcctctgcttccctgggatcctcggtcaagctcacctgcactctgagcagtgggcacagtagctacatcatcgcatggcatccagcag
IGLV4-60*02   tgcctctgcttccctgggatcctcggtcaagctcacctgcactctgagcagtgggcacagtagctacatcatcgcatggcat-cagcag
IGLV4-60*01   tgcctctgcttccctgggatcctcggtcaagctcacctgcactctgagcagtgggcacagtagctacatcatcgcatggcat-cagcag
IGLV4-60*03   tgcctctgcttccctgggatcctcggtcaagctcacctgcactctgagcagtgggcacagtagctacatcatcgcatggcat-cagcag
V_V4-60*02    tgcctctgcttccctgggatcctcggtcaagctcacctgcactctgagcagtgggcacagtagctacatcatcgcatggcat-cagcag

V4-60_01      cagccagggaaggcccctcggtacttgatgaagcttgaaggtagtggaagctacaacaaggggagcggagttcctgatcgcttctcagg
V4-60_02      cagccagggaaggcccctcggtacttgatgaagcttgaaggtagtggaagctacaacaaggggagcggagttcctgatcgcttctcagg
V4-60_03      cagccagggaaggcccctcggtacttgatgaagcttgaaggtagtggaagctacaacaaggggagcggagttcctgatcgcttctcagg
V4-60_04      cagccagggaaggcccctcggtacttgatgaagcttgaaggtagtggaagctacaacaaggggagcggagttcctgatcgcttctcagg
V4-60_05      cagccagggaaggcccctcggtacttgatgaagcttgaaggtagtggaagctacaacaaggggagcggagttcctgatcgcttctcagg
V4-60_06      cagccagggaaggcccctcggtacttgatgaagcttgaaggtagtggaagctacaacaaggggagcggagttcctgatcgcttctcagg
V4-60_07      cagccagggaaggcccctcggtacttgatgaagcttgaaggtagtggaagctacaacaaggggagcggagttcctgatcgcttctcagg
V4-60_08      cagccagggaaggcccctcggtacttgatgaagcttgaaggtaatggaagctacaacaaggggagcggagttcctgatcgcttctcagg
IGLV4-60*02   cagccagggaaggcccctcggtacttgatgaagcttgaaggtagtggaagctacaacaaggggagcggagttcctgatcgcttctcagg
IGLV4-60*01   cagccagggaaggcccctcggtacttgatgaagcttgaaggtagtggaagctacaacaaggggagcggagttcctgatcgcttctcagg
IGLV4-60*03   cagccagggaaggcccctcggtacttgatgaagcttgaaggtagtggaagctacaacaaggggagcggagttcctgatcgcttctcagg
V_V4-60*02    cagccagggaaggcccctcggtacttgatgaagcttgaaggtagtggaagctacaacaaggggagcggagttcctgatcgcttctcagg
```

```
V4-60_01        ctccagctctggggctgaccgctacctcaccatctccaacctccagtttgaggatgaggctgattattactgtgagacctgggacagtaacactca
V4-60_02        ctccagctctggggctgaccgctacctcaccatctccaacctccagtctgaggatgaggctgattattactgtgagacctgggacagtaacactca
V4-60_03        ctccagctctggggctgactgctacctcaccatctccaacctccagtctgaggatgaggctgattattactgtgagacctgggacagtaacactca
V4-60_04        ctccagctctggggctgactgctacctcaccatctccaacctccagtctgaggatgaggctgattattactgtgagacctgggacagtaacactca
V4-60_05        ctccagctctgtggctgaccgctacctcaccatctccaacctccagtctgaggatgaggctgattattactgtgagacctgggacagtaacactca
V4-60_06        ctccagctctggggctgaccgctacctcaccatctccaacctccagtctgaggatgaggctgattattactgtgagacctgggacagtaacactca
V4-60_07        ctccagctctggggctgaccgctacctcaccatctccaacctccagtctgaggatgaggctgattattactgtgagacctgggacagtaacactca
V4-60_08        ctccagctctggggctgaccgctacctcaccatctccaacctccagtctgaggatgaggctgattattactgtgagacctgggacagtaacactca
IGLV4-60*02     ctccagctctggggctgaccgctacctcaccatctccaacctccagtttgaggatgaggctgattattactgtgagacctgggacagtaacactca
IGLV4-60*01     ctccagctctggggctgaccgctacctcaccatctccaacctccagttagaggatgaggctgattattactgtgagacctgggacagtaacact--
IGLV4-60*03     ctccagctctggggctgaccgctacctcaccatctccaacctccagtctgaggatgaggctgattattactgtgagacctgggacagtaacact--
V_V4-60*02      ctccagctctggggctgaccgctacctcaccatctccaacctccagtttgaggatgaggctgattattactgtgagacctgggacagtaacact--
```

IGLV4-69

```
IGLV4-69_1     agggtgggtaagaaatacctgcaactgtcagcctcagcagagctctggggagtctgcaccatggcttggaccccactcctcttcctcaccc
IGLV4-69_2     agggtgggtaagaaatacctgcaactgtcagcctcagcagagctctggggagtctgcaccatggcttggaccccactcctcttcctcaccc
IGLV4-69_3     agggtgggtaagaaatacctgcaactgtcagcctcagcagagctctggggagtctgcaccatggcttagaccccactcctcttcctcaccc
IGLV4-69_4     agggtgggtaagaaatacctgcaactgtcagcctcagcagagctctggggagtctgcaccatggcttggaccccactcctcttcctcaccc
IGLV4-69_5     agggtgggtaagaaatacctgcaactgtcagcctcagcagagctctggggagactgcaccatggcttggaccccactcctcttcctcaccc
IGLV4-69_6     agggtgggtaagaaatacctgcaactgtcagcctcagcagagctctggggagtctgcaccatggcttggaccccactcctcttcctcaccc
IGLV4-69*01    -------------------------------------------------------------------------------------------
IGLV4-69*02    --------------------------------------------------------------------------------cttcctcaccc
V_V4-69*01     -------------------------------------------------------------------------------------------

IGLV4-69_1     tcctcctccactgcacagggtctctctcccagcttgtgctgactcaatcgccctctgcctctgcctccctgggagcctcggtcaagctc
IGLV4-69_2     tcctcctccactgcacagggtctctctcccagcttgtgctgactcaatcgccctctgcctctgcctctctgggagcctcggtcaagctc
IGLV4-69_3     tcctcctccactgcacagggtctctctcccagcttgtgctgactcaatcgccctctgcctctgcctccctgggagcctcggtcaagctc
IGLV4-69_4     tcctcctccactgcacagggtccctctcccagcttgtgctgactcaatcgccctctgcctctgcctccctgggagcctcggtcaagctc
IGLV4-69_5     tcctcctccactgcacagggtctctctcccagcttgtgctgactcaatcgccctctgcctctgcctccctgggagcctcggtcaagctc
IGLV4-69_6     tcctcctccactgcacagggtctctctcccagcttgtgctgactcaatcgccctctgcctctgcctccctgggagcctcggtcaagctc
IGLV4-69*01    -----------------------------cagcttgtgctgactcaatcgccctctgcctctgcctccctgggagcctcggtcaagctc
IGLV4-69*02    tcctcctccactgcacagggtctctctcccagcttgtgctgactcaatcgccctctgcctctgcctccctgggagcctcggtcaagctc
V_V4-69*01     -----------------------------cagcttgtgctgactcaatcgccctctgcctctgcctccctgggagcctcggtcaagctc

IGLV4-69_1     acctgcactctgagcagtgggcacagcagctacgccatcgcatggcatcagcagcagccagagaagggccctcggtacttgatgaagcttaa
IGLV4-69_2     acctgcactctgagcagtgggcacagcagctacgccatcgcatggcatcagcagcagccagagaagggccctcggtacttgatgaagcttaa
IGLV4-69_3     acctgcactctgagcagtgggcacagcagctacgccatcgcatggcatcagcagcagccagagaagggccctcggtacttgatgaagcttaa
IGLV4-69_4     acctgcactctgagcagtgggcacagcagctacgccatcgcatggcatcagcagcagccagagaagggccctcggtacttgatgaagcttaa
IGLV4-69_5     acctgcactctgagcagtgggcacagcagctacgccatcgcatggcatcagcagcagccagagaagggccctcggtacttgatgaagcttaa
IGLV4-69_6     acctgcactctgagcagtgggcacagcagctacgccatcgcatggcatcagcagcagccagagaagggccctcggtacttgatgaagcttaa
IGLV4-69*01    acctgcactctgagcagtgggcacagcagctacgccatcgcatggcatcagcagcagccagagaagggccctcggtacttgatgaagcttaa
IGLV4-69*02    acctgcactctgagcagtgggcacagcagctacgccatcgcatggcatcagcagcagccagagaagggccctcggtacttgatgaagcttaa
V_V4-69*01     acctgcactctgagcagtgggcacagcagctacgccatcgcatggcatcagcagcagccagagaagggccctcggtacttgatgaagcttaa

IGLV4-69_1     cagtgatggcagccacagcaaggggggacgggatccctgatcgcttctcaggctccagctctggggctgagcgctacctcaccatctcc
IGLV4-69_2     cagtgatggcagccacagcaaggggggacgggatccctgatcgcttctcaggctccagctctggggctgagcgctacctcaccatctcc
IGLV4-69_3     cagtgatggcagccacagcaaggggggacgggatccctgatcgcttctcaggctccagctctggggctgagcgctacctcaccatctcc
IGLV4-69_4     cagtgatggcagccacagcaaggaggacgggatccctgatcgcttctcaggctccagctctggggctgagcgctacctcaccatctcc
IGLV4-69_5     cagtgatggcagccacagcaaggggggacgggatccctgatcgcttctcaggctccagctctggggctgagcgctacctcaccatctcc
IGLV4-69_6     cagtgatggcagccacagcaaggggggacgggatccctgatcgcttctcaggctccagctctggggctgagcgctacctcaccatctcc
IGLV4-69*01    cagtgatggcagccacagcaaggggggacgggatccctgatcgcttctcaggctccagctctggggctgagcgctacctcaccatctcc
IGLV4-69*02    cagtgatggcagccacagtaaggggggacgggatccctgatcgcttctcaggctccagctctggggctgagcgctacctcaccatctcc
V_V4-69*01     cagtgatggcagccacagcaaggggggacgggatccctgatcgcttctcaggctccagctctggggctgagcgctacctcaccatctcc
```

IGLV4-69_1	agcctccagtctgaggatgaggctgactattactgtcagacctggggcactggcattca
IGLV4-69_2	agcctccagtctgaggatgaggctgactattactgtcagacctggggcactggcattca
IGLV4-69_3	agcctccagtctgaggatgaggctgactattactgtcagacctggggcactggcattca
IGLV4-69_4	agcctccagtctgaggatgaggctgactattactgtcagacctggggcactggcattca
IGLV4-69_5	agcctccagtctgaggatgaggctgactattactgtcagacctggggcactggcattca
IGLV4-69_6	agcctccagtctgaggatgaggctgactattactgtcagacctggggtactggcattca
IGLV4-69*01	agcctccagtctgaggatgaggctgactattactgtcagacctggggcactggcattca
IGLV4-69*02	agcctccagtctgaggatgaggctgactattactgtcagacctggggcactggcattca
V_V4-69*01	agcctccagtctgaggatgaggctgactattactgtcagacctggggcactggcatt--

IGLV5-37

```
V5-37_01      atggcctggactcctcttcttctcttgctcctctctcactgcacaggttccctctcccagcctgtgctgactcagccaccttcctcctc
V5-37_02      atggcctggactcctcttcttctcttgctcctctctcactgcacaggttccctctcccagcctgtgctgactcagccaccttcctcctc
V5-37_03      atggcctggactcctcttcttctcttgctcctctctcactgcacaggttccctctcccagcctgtgctgactcagccaccttcctcctc
V5-37_04      atggcctggactcctcttcttctcttgctcctctctcactgcacaggttccctctcccagcctgtgctgactcagccaccttcctcctc
V5-37_05      atggcctggactcctcttcttctcttgctcctctctcactgcacaggttccctctcccagcctgtgctgactcagccaccttcctcctc
V5-37_06      atggcctggactcctcttcttctcttgctcctctctcactgcacaggttccctctcccagcctgtgctgactcagccaccttcctcctc
V5-37_07      atggcctggactcctcttcttctcttgctcctctctcactgcacaggttccctctcccagcctgtgctgactcagccaccttcctcctc
V5-37_08      atggcctggactcctcttcttctcttgctcctctctcactgcacaggttccctctcccagcctgtgctgactcagccaccttcctcctc
IGLV5-37*01   ---------------------------------------------------------cagcctgtgctgactcagccaccttcctcctc
V_V5-37*01    ---------------------------------------------------------cagcctgtgctgactcagccaccttcctcctc


V5-37_01      cgcatctcctggagaatccgccagactcacctgcaccttgcccagtgacatcaatgttggtagctacaacatatactggtaccagcagaag
V5-37_02      cgcatctcctggagaatccgccagactcacctgcaccttgcccagtgacatcaatgttagtagctacaacatatactggtaccagcagaag
V5-37_03      cgcatctcctggagaatccgccagactcacctgcaccttgcccagtgacatcaatgttggtagctacaacatatactggtaccagcagaag
V5-37_04      cgcatctcctggagaatccgccagactcacctgcaccttgcccagtgacatcaatgttggtagctacaacatatactggtaccagcagaag
V5-37_05      cgcatctcctggagaatccgccagactcacctgcaccttgcccagtgacatcaatgttggtagctacaacatatactggtaccagcagaag
V5-37_06      cgcatctcctggagaatccgccagactcacctgcaccttgcccagtgacatcaatgttagtagctacaacatatactggtaccagcagaag
V5-37_07      cgcatctcctggagaatccgccagactcacctgcaccttgcccagtgacatcaatgttggtagctacaacatatactggtaccagcagaag
V5-37_08      cgcatctcctggagaatccgccagactcacctgcaccttgcccagtgacatcaatgttggtagctacaacatatactggtaccagcagaag
IGLV5-37*01   cgcatctcctggagaatccgccagactcacctgcaccttgcccagtgacatcaatgttggtagctacaacatatactggtaccagcagaag
V_V5-37*01    cgcatctcctggagaatccgccagactcacctgcaccttgcccagtgacatcaatgttggtagctacaacatatactggtaccagcagaag


V5-37_01      ccagggagccctcccaggtatctcctgtactactactcagactcagataagggccagggctctggagtccccagccgcttctctggatccaa
V5-37_02      ccagggagccctcccaggtatctcctgtactactactcagactcagataagggccagggctctggagtccccagccgcttctctggatccaa
V5-37_03      ccagggagccctcccaggtatctcctgaactactactcagactcagataagggccagggctctggagtccccagccgcttctctggatccaa
V5-37_04      ccagggagccctcccaggtatctcctgtactactactcagactcagataagggccagggctctggagtccccagccgcttctctggatccaa
V5-37_05      ccagggagccctcccaggtatctcctgaactactactcagactcagataagggccagggctctggagtccccagctgcttctctggatccaa
V5-37_06      ccagggagccctcccaggtatctcctgaactactactcagactcagataagggccagggctctggagtccccagccgcttctctggatccaa
V5-37_07      ccagggagccctcccaggtatctcctgaactactactcagactcagataagggccagggctctggagtccccagccgcttctctggatccaa
V5-37_08      ccagggagccctcccaggtatctcctgtactactactcagactcagataagggccagggctctggagtccccagctgcttctctggatccaa
IGLV5-37*01   ccagggagccctcccaggtatctcctgtactactactcagactcagataagggccagggctctggagtccccagccgcttctctggatccaa
V_V5-37*01    ccagggagccctcccaggtatctcctgtactactactcagactcagataagggccagggctctggagtccccagccgcttctctggatccaa
```

```
V5-37_01       agatgcttcagccaatacagggattttactcatctccgggctccagtctgaggatgaggctgactattactgtatgatttggccaagcaatgcttct
V5-37_02       agatgcttcagccaatacagggattttactcatctccgggctccagtctgaggatgaggctgactattactgtatgatttggccaagcaatgcttct
V5-37_03       agatgcttcagccaatacagggattttactcatctccgggctccagtctgaggatgaggctgactattactgtatgatttggccaagcaatgcttct
V5-37_04       agatgcttcggccaatacagggattttactcatctccgggctccagtctgaggatgaggctgactattactgtatgatttggccaagcaatgcttct
V5-37_05       agatgcttcagccaatacagggattttactcatctccgggctccagtctgaggatgaggctgactattactgtatgatttggccaagcaatgcttct
V5-37_06       agatgcttcagccaatacagggattttactcatctccgggctccagtctgaggatgaggctgactattactgtatgatttggccaagcaatgcttct
V5-37_07       agatgcttcggccaatacagggattttactcatctccgggctccagtctgaggatgaggctgactattactgtatgatttggccaagcaatgcttct
V5-37_08       agatgcttcggccaatacagggattttactcatctccgggctccagtctgaggatgaggctgactattactgtatgatttggccaagcaatgcttct
IGLV5-37*01    agatgcttcagccaatacagggattttactcatctccgggctccagtctgaggatgaggctgactattactgtatgatttggccaagcaatgcttct
V_V5-37*01     agatgcttcagccaatacagggattttactcatctccgggctccagtctgaggatgaggctgactattactgtatgatttggccaagcaatgct---
```

IGLV5-45

```
V5-45_01        atggcctggactcctctcctcctcctgttcctctctcactgcacaggttccctctcgcaggctgtgctgactcagccggcttccctctc
V5-45_02        atggcctggactcctctcctcctcctgttcctctctcactgcacaggttccctctcgcaggctgtgctgactcagccgtcttccctctc
V5-45_03        atggcctggactcctctcctcctcctgttcctctctcactgcacaggttccctctcgcaggctgtgctgactcagccgtcttccctctc
V5-45_04        atggcctggactcctctcctcctcctgttcctctctcactgcacaggttccctctcgcaggctgtgctgactcagccgtcttccctctc
V5-45_05        atggcctggactcctctcctcctcctgttcctctctcactgcacaggttccctctcgcaggctgtgctgactcagccgtcttccctctc
V5-45_06        atggcctggactcctctcctcctcctgttcctctctcactgcacaggttccctctcgcaggctgtgctgactcagctggcttccctctc
V5-45_07        atggcctggactcctctcctcctcctgttcctctctcactgcacaggttccctctcacaggctgtgctgactcagccgtcttccctctc
IGLV5-45*03     atggcctggactcctctcctcctcctgttcctctctcactgcacaggttccctctcgcaggctgtgctgactcagccgtcttccctctc
IGLV5-45*04     atggcctggactcctctcctcctcctgttcctctctcactgcacaggttccctctcgcaggctgtgctgactcagccgtcttccctctc
IGLV5-45*01     ---------------------------------------------------------caggctgtgctgactcagccggcttccctctc
IGLV5-45*02     ---------------------------------------------------------caggctgtgctgactcagccgtcttccctctc
V_V5-45*02      ---------------------------------------------------------caggctgtgctgactcagccgtcttccctctc
V_V5-45*03      ---------------------------------------------------------caggctgtgctgactcagccgtcttccctctc
```

```
V5-45_01        tgcatctcctggagcatcagccagtctcacctgcaccttgcgcagtggcatcaatgttggtacctacaggatatactggtaccagcagaag
V5-45_02        tgcatctcctggagcatcagccagtctcacctgcaccttacgcagtggcatcaatgttggtacctacaggatatactggtaccagcagaag
V5-45_03        tgcatctcctggagcatcagccagtctcacctgcaccttgcgcagtggcatcaatgttggtacctacaggatatactggtaccagcagaag
V5-45_04        tgcatctcctggagcatcagccagtctcacctgcaccttgtgcagtggcatcaatgttggtacctacaggatatactggtaccagcagaag
V5-45_05        tgcatctcctggagcatcagccagtctcacctgcaccttgcgcagtggcatcaatgttggtacctacaggatatactggtaccagcagaag
V5-45_06        tgcatctcctggagcatcagccagtctcacctgcaccttgcgcagtggcatcaatgttggtacctacaggatatactggtaccagcagaag
V5-45_07        tgcatctcctggagcatcagccagtctcacctgcaccttgcgcagtggcatcaatgttggtacctacaggatatactggtaccagcagaag
IGLV5-45*03     tgcatctcctggagcatcagccagtctcacctgcaccttgcgcagtggcatcaatgttggtacctacaggatatactggtaccagcagaag
IGLV5-45*04     tgcatctcctggagcatcagccagtctcacctgcaccttgtgcagtggcatcaatgttggtacctacaggatatactggtaccagcagaag
IGLV5-45*01     tgcatctcctggagcatcagccagtctcacctgcaccttgcgcagtggcatcaatgttggtacctacaggatatactggtaccagcagaag
IGLV5-45*02     tgcatctcctggagcatcagccagtctcacctgcaccttacgcagtggcatcaatgttggtacctacaggatatactggtaccagcagaag
V_V5-45*02      tgcatctcctggagcatcagccagtctcacctgcaccttacgcagtggcatcaatgttggtacctacaggatatactggtaccagcagaag
V_V5-45*03      tgcatctcctggagcatcagccagtctcacctgcaccttgcgcagtggcatcaatgttggtacctacaggatatactggtaccagcagaag
```

```
V5-45_01        ccagggagtcctccccagtatctcctgaggtacaaatcagactcagataagcagcagggctctggagtccccagccgcttctctggatcc
V5-45_02        ccagggagtcctccccagtatctcctgaggtacaaatcagactcagataagcagcagggctctggagtccccagccgcttctctggatcc
V5-45_03        ccagggagtcctccccagtatctcctgaggtacaaatcagactcagataagcagcagggctctggagtccccagccgcttctctggatcc
V5-45_04        ccagggagtcctccccagtatctcctgaggtacaaatcagactcagataagcagcagggctctggagtccccagccgcttctctggatcc
V5-45_05        ccagggagtcctccccagtatctcctgaggtacaaatcagactcagataagcagcagggctctggagtccccagccgcttctctggatcc
V5-45_06        ccagggagtcctccccagtatctcctgaggtacaaatcagactcagataagcagcagggctctggagtccccagccgcttctctggatcc
V5-45_07        ccagggagtcctccccagtatctcctgaggtacaaatcagactcagataagcagcagggctctggagtccccagccgcttctctggatcc
IGLV5-45*03     ccagggagtcctccccagtatctcctgaggtacaaatcagactcagataagcagcagggctctggagtccccagccgcttctctggatcc
IGLV5-45*04     ccagggagtcctccccagtatctcctgaggtacaaatcagactcagataagcagcagggctctggagtccccagccgcttctctggatcc
IGLV5-45*01     ccagggagtcctccccagtatctcctgaggtacaaatcagactcagataagcagcagggctctggagtccccagccgcttctctggatcc
IGLV5-45*02     ccagggagtcctccccagtatctcctgaggtacaaatcagactcagataagcagcagggctctggagtccccagccgcttctctggatcc
V_V5-45*02      ccagggagtcctccccagtatctcctgaggtacaaatcagactcagataagcagcagggctctggagtccccagccgcttctctggatcc
V_V5-45*03      ccagggagtcctccccagtatctcctgaggtacaaatcagactcagataagcagcagggctctggagtccccagccgcttctctggatcc
```

```
V5-45_01        aaagatgcttcggccaatgcagggattttactcatctctgggctccagtctgaggatgaggctgactattactgtatgatttggcacagcagcgcttct
V5-45_02        aaagatgcttcggccaatgcagggattttactcatctctgggctccagtctgaggatgaggctgactattactgtatgatttggcacagcagcgcttct
V5-45_03        aaagatgcttcggccaatgcagggattttactcatctctgggctccagtctgaggatgaggctgactattactgtatgatttggcacagcagcgcttct
V5-45_04        aaagatgcttcggccaatgcagggattttactcatctctgggctccagtctgaggatgaggctgactattactgtatgatttggcacagcagcgcttct
V5-45_05        aaagatgcttcggccaatgcagggattttactcatctctgggctccagtctgaggatgaggctgactactactgtatgatttggcacagcagcgcttct
V5-45_06        aaagatgcttcggccaatgcagggattttactcatctctgggctccagtctgaggatgaggctgactattactgtatgatttggcacagcagcgcttct
V5-45_07        aaagatgcttcggccaatgcagggattttactcatctctgggctccagtctgaggatgaggctgactattactgtatgatttggcacagcagcgcttct
IGLV5-45*03     aaagatgcttcggccaatgcagggattttactcatctctgggctccagtctgaggatgaggctgactattactgtatgatttggcacagcagcgcttct
IGLV5-45*04     aaagatgcttcggccaatgcagggattttactcatctctgggctccagtctgaggatgaggctgactattactgtatgatttggcacagcagcgcttct
IGLV5-45*01     aaagatgcttcggccaatgcagggattttactcatctctgggctccagtctgaggatgaggctgactattactgtatgatttggcacagcagcgcttct
IGLV5-45*02     aaagatgcttcggccaatgcagggattttactcatctctgggctccagtctgaggatgaggctgactattactgtatgatttggcacagcagcgcttct
V_V5-45*02      aaagatgcttcggccaatgcagggattttactcatctctgggctccagtctgaggatgaggctgactattactgtatgatttggcacagcagcgct---
V_V5-45*03      aaagatgcttcggccaatgcagggattttactcatctctgggctccagtctgaggatgaggctgactattactgtatgatttggcacagcagcgct---
```

IGLV5-52

```
V5-52_01      atggcctggactcttctcctccttctcgtgctcctctctcactgcacaggttccctctcccagcctgtgctgactcagccatcttcccattct
V5-52_02      atggcctggactctt---ctccttctcgtgctcctctctcactgcacaggttccctctcccagcctgtgctgactcagccatcttcccattct
IGLV5-52*01   ------------------------------------------------------------cagcctgtgctgactcagccatcttcccattct
V_V5-52*01    ------------------------------------------------------------cagcctgtgctgactcagccatcttcccattct

V5-52_01      gcatcttctggagcatcagtcagactcacctgcatgctgagcagtggcttcagtgttggggacttctggataaggtggtaccaacaaaagcca
V5-52_02      gcatcttctggagcatcagtcagactcacctgcatgctgagcagtggcttcagtgttggggacttctggataaggtggtaccaacaaaagcca
IGLV5-52*01   gcatcttctggagcatcagtcagactcacctgcatgctgagcagtggcttcagtgttggggacttctggataaggtggtaccaacaaaagcca
V_V5-52*01    gcatcttctggagcatcagtcagactcacctgcatgctgagcagtggcttcagtgttggggacttctggataaggtggtaccaacaaaagcca

V5-52_01      gggaaccctccccggtatctcctgtactaccactcagactccaataagggccaaggctctggagttcccagccgcttctctggatccaacgat
V5-52_02      gggaaccctccccggtatctcctgtactaccactcagactccaataagggccaaggctctggagttcccagccgcttctctggatccaacgat
IGLV5-52*01   gggaaccctccccggtatctcctgtactaccactcagactccaataagggccaaggctctggagttcccagccgcttctctggatccaacgat
V_V5-52*01    gggaaccctccccggtatctcctgtactaccactcagactccaataagggccaaggctctggagttcccagccgcttctctggatccaacgat

V5-52_01      gcatcagccaatgcagggattctgcgtatctctgggctccagcctgaggatgaggctgactattactgtggtacatggcacagcaactctaagactca
V5-52_02      gcatcagccaatgcagggattctgcgtatctctgggctccagcctgaggatgaggctgactattactgtggtacatggcacagcaactctaagactca
IGLV5-52*01   gcatcagccaatgcagggattctgcgtatctctgggctccagcctgaggatgaggctgactattactgtggtacatggcacagcaactctaagactca
V_V5-52*01    gcatcagccaatgcagggattctgcgtatctctgggctccagcctgaggatgaggctgactattactgtggtacatggcacagcaactct--------
```

IGLV6-57

```
V6-57_01       atggcctgggctccactacttctcaccctcctcgctcactgcacaggttcttgggccaattttatgctgactcagccccactctgtgtc
V6-57_02       atggcctgggctccactacttctcaccctcctcgctcactgcacaggttcttgggccaattttatgctgactcagccccactctgtgtc
V6-57_03       atggcctgggctccactacttctcaccctcctcgctcactgcacaggttcttgggccaattttatgctgactcagccccactctgtgtc
V6-57_04       atggcctgggctccactacttctcaccctcctcgctcactgcacaggttcttgggccaattttatgctgactcagccgcactctgtgtc
V6-57_05       atggcctgggctccactacttctcaccctcctcgctcactgcacaggttcttgggccaattttatgctgactcagccccactctgtgtc
IGLV6-57*02    atggcctgggctccactacttctcaccctcctcgctcactgcacaggttcttgggccaattttatgctgactcagccccactctgtgtc
IGLV6-57*01    ---------------------------------------------------------aattttatgctgactcagccccactctgtgtc
V_V6-57*01     ---------------------------------------------------------aattttatgctgactcagccccactctgtgtc

V6-57_01       ggagtctccggggaagacggtaaccatctcctgcacccgcagcagtggcagcattgccagcaactatgtgcagtggtaccagcagcgcccg
V6-57_02       ggagtctccggggaagacggtaaccatctcctgcaccggcagcagtggcagcattgccagcaactatgtgcagtggtaccagcagcgcccg
V6-57_03       ggagtctccggggaagacggtaaccatctcctgcacccgcagcagtggcagcattgccagcaactatgtgcagtggtaccagcagcgcccg
V6-57_04       ggagtctccggggaagacggtaaccatctcctgcacccgcagcagtggcagcattgccagcaactatgtgcagtggtaccagcagcgcccg
V6-57_05       ggagtctccggggaagacggtaaccatctcctgcacctgcagcagtggcagcattgccagcaactatgtgcagtggtaccagcagcgcccg
IGLV6-57*02    ggagtctccggggaagacggtaaccatctcctgcaccggcagcagtggcagcattgccagcaactatgtgcagtggtaccagcagcgcccg
IGLV6-57*01    ggagtctccggggaagacggtaaccatctcctgcacccgcagcagtggcagcattgccagcaactatgtgcagtggtaccagcagcgcccg
V_V6-57*01     ggagtctccggggaagacggtaaccatctcctgcacccgcagcagtggcagcattgccagcaactatgtgcagtggtaccagcagcgcccg

V6-57_01       ggcagttccccaccactgtgatctatgaggataaccaaagaccctctggggtccctgatcggttctctggctccatcgacagctcctccaac
V6-57_02       ggcagtgccccaccactgtgatctatgaggataaccaaagaccctctggggtccctgatcggttctctggctccatcgacagctcctccaac
V6-57_03       ggcagtgccccaccactgtgatctatgaggataaccaaagaccctctggggtccctgatcggttctctggctccatcgacagctcctccaac
V6-57_04       ggcagtgccccaccactgtgatctatgaggataaccaaagaccctctggggtccctgatcggttctctggctccatcgacagctcctccaac
V6-57_05       ggcagttccccaccactgtgatctatgaggataaccaaagaccctctggggtccctgatcggttctctggctccatcgacagctcctccaac
IGLV6-57*02    ggcagtgccccaccactgtgatctatgaggataaccaaagaccctctggggtccctgatcggttctctggctccatcgacagctcctccaac
IGLV6-57*01    ggcagttccccaccactgtgatctatgaggataaccaaagaccctctggggtccctgatcggttctctggctccatcgacagctcctccaac
V_V6-57*01     ggcagttccccaccactgtgatctatgaggataaccaaagaccctctggggtccctgatcggttctctggctccatcgacagctcctccaac

V6-57_01       tctgcctccctcaccatctctggactgaagactgaggacgaggctgactactactgtcagtcttatgatagcagcaatca
V6-57_02       tctgcctccctcaccatctctggactgaagactgaggacgaggctgactactactgtcagtcttatgatagcagcaatca
V6-57_03       tctgcctccctcaccatctctggactgaagactgaggacgaggctgactactactgtcagtcttatgatagcagcaatca
V6-57_04       tctgcctccctcaccatctctggactgaagactgaggacgaggctgactactactgtcagtcttatgatagcagcaatca
V6-57_05       tctgcctccctcaccatctctggactgaagactgaggacgaggctgactactactgtcagtcttatgatagcagcaatca
IGLV6-57*02    tctgcctccctcaccatctctggactgaagactgaggacgaggctgactactactgtcagtcttatgatagcagcaatca
IGLV6-57*01    tctgcctccctcaccatctctggactgaagactgaggacgaggctgactactactgtcagtcttatgatagcagcaatca
V_V6-57*01     tctgcctccctcaccatctctggactgaagactgaggacgaggctgactactactgtcagtcttatgatagcagcaat--
```

IGLV7-43

```
V7-43_01      atggcctggactcctctctttctgttcctcctcacttgctgcccagggtccaattctcagactgtggtgactcaggagccctcactgac
V7-43_02      atggcctggactcctctctttctgttcctcctcacttgctgcccagggtccaattctcagactgtggtgactcaggagccctcactgac
V7-43_03      atggcctggactcctctctttctgttcctcctcacttgctgcccagggtccaattctcagactgtggtgactcaggagccctcactgac
V7-43_04      atggcctggactcctctctttctgttcctcctcacttgctgcccagggtccaattctcagactgtggtgactcaggagccctcactgac
IGLV7-43*01   atggcctggactcctctctttctgttcctcctcacttgctgcccagggtccaattctcagactgtggtgactcaggagccctcactgac
V_V7-43*01    -----------------------------------------------------------cagactgtggtgactcaggagccctcactgac

V7-43_01      tgtgtccccaggagggacagtcactctcacctgtgcttccagcactggagcagtcaccagtggttactatccaaactggttccagcagaaa
V7-43_02      tgtgtccccaggagggacagtcactctcacctgtgcttccagcactggagcagtcaccagtggttactatccaaactggttccagcagaaa
V7-43_03      tgtgtccccaggagggacagtcactctcatctgtgcttccagcactggagcagtcaccagtggttactatccaaactggttccagcagaaa
V7-43_04      tgtatccccaggagggacagtcactctcacctgtgcttccagcactggagcagtcaccagtggttactatccaaactggttccagcagaaa
IGLV7-43*01   tgtgtccccaggagggacagtcactctcacctgtgcttccagcactggagcagtcaccagtggttactatccaaactggttccagcagaaa
V_V7-43*01    tgtgtccccaggagggacagtcactctcacctgtgcttccagcactggagcagtcaccagtggttactatccaaactggttccagcagaaa

V7-43_01      cctggacaagcacccagggcactgatttatagtacaagcaacaaacactcctggacccctgcccggttctcaggctccctccttgggggc
V7-43_02      cctggacaagcacccagggtactgatttatagtacaagcaacaaacactcctggacccctgcccggttctcaggctccctccttgggggc
V7-43_03      cctggacaagcacccagggcactgatttatagtacaagcaacaaacactcctggacccctgcccggttctcaggctccctccttgggggc
V7-43_04      cctggacaagcacccagggcactgatttatagtacaagcaacaaacactcctggacccctgcccggttctcaggctccctccttgggggc
IGLV7-43*01   cctggacaagcacccagggcactgatttatagtacaagcaacaaacactcctggacccctgcccggttctcaggctccctccttgggggc
V_V7-43*01    cctggacaagcacccagggcactgatttatagtacaagcaacaaacactcctggacccctgcccggttctcaggctccctccttgggggc

V7-43_01      aaagctgccctgacactgtcaggtgtgcagcctgaggacgaggctgagtattactgcctgctctactatggtggtgctcag
V7-43_02      aaagctgccctgacactgtcaggtgtgcagcctgaggacgaggctgagtattactgcctgctctactatggtggtgctcag
V7-43_03      aaagctgccctgacactgtcaggtgtgcagcctgaggacgaggctgagtattactgcctgctctactatggtggtgctcag
V7-43_04      aaagctgccctgacactgtcaggtgtgcagcctgaggacgaggctgagtattactgcctgctctactatggtggtgctcag
IGLV7-43*01   aaagctgccctgacactgtcaggtgtgcagcctgaggacgaggctgagtattactgcctgctctactatggtggtgctcag
V_V7-43*01    aaagctgccctgacactgtcaggtgtgcagcctgaggacgaggctgagtattactgcctgctctactatggtggtgct---
```

IGLV7-46

```
V7-46_01      atggcctggactcctctctttctgttcctcctcacttgctgccccagggtccaattcccaggctgtggtgactcaggagccctcactgact
V7-46_02      atggcctggactcctctctttctgttcctcctcacttgctg-cccagggtccaattcccaggctgtggtgactcaggagccctcactgact
V7-46_03      atggcctggactcctctctttctgttcctcctcacttgctgccccagggtccaattcccaggctgtggtgactcaggagccctcactgact
V7-46_04      atggcctggactcctctctttctgttcctcctcacttgctgccccagggtccaattcccaggctgtggtgactcaggagccctcactgact
V7-46_05      atggcctggactcctctctttctgttcctcctcacttgctgccccagggtccaattcccaggctgtggtgactcaggagccctcactgact
V7-46_06      atggcctggactcctctctttctgttcctcctcacttgctgccccagggtccaattcccaggctgtggtgactcaggagccctcattgact
IGLV7-46*02   atggcctggactcctctctttctgttcctcctcacttgctg-cccagggtccaattcccaggctgtggtgactcaggagccctcactgact
IGLV7-46*01   ----------------------------------------------------------caggctgtggtgactcaggagccctcactgact
IGLV7-46*03   ----------------------------------------------------------caggctgtggtgactcaggagccctcactgact
V_V7-46*01    ----------------------------------------------------------caggctgtggtgactcaggagccctcactgact
V_V7-46*02    ----------------------------------------------------------caggctgtggtgactcaggagccctcactgact

V7-46_01      gtgtccccaggagggacagtcactctcacctgtggctccagcactggagctgtcaccagtggtcattatccctactggttccagcagaa
V7-46_02      gtgtccccaggagggacagtcactctcacctgtggctccagcactggagctgtcaccagtggtcattatccctactggttccagcagaa
V7-46_03      gtgtccccaggagggacagtcactctcacctgtggctccagcactggagctgtcaccagtggtcattatccctactggttccagcagaa
V7-46_04      gtgtccccaggagggacagtcactctcacctgtggctccagcactggagctgtcaccagtggtcattatccctactggttccagcagaa
V7-46_05      gtatccccaggagggacagtcactctcacctgtggctccagcactggagctgtcaccagtggtcattatccctactggttccagcagaa
V7-46_06      gtgtccccaggagggacagtcactctcacctgtggctccagcactggagctgtcaccagtggtcattatccctactggttccagcagaa
IGLV7-46*02   gtgtccccaggagggacagtcactctcacctgtggctccagcactggagctgtcaccagtggtcattatccctactggttccagcagaa
IGLV7-46*01   gtgtccccaggagggacagtcactctcacctgtggctccagcactggagctgtcaccagtggtcattatccctactggttccagcagaa
IGLV7-46*03   gtgtccccaggagggacagtcactctcacctgtggctccagcactggagctgtcaccagtggtcattatccctactggttccagcagaa
V_V7-46*01    gtgtccccaggagggacagtcactctcacctgtggctccagcactggagctgtcaccagtggtcattatccctactggttccagcagaa
V_V7-46*02    gtgtccccaggagggacagtcactctcacctgtggctccagcactggagctgtcaccagtggtcattatccctactggttccagcagaa

V7-46_01      gcctggccaagcccccaggacactgatttatgatacaagcaacaaacactcctggacaacctgcccggttctcaggctccctccttgggggcaa
V7-46_02      gcctggccaagcccccaggacactgatttatgatacaagcaacaaacactcctggacaacctgcccggttctcaggctccctccttgggggcaa
V7-46_03      gcctggccaagcccccaggacactgatttatgatacaagcaacaaacactcctggaccacctgcccggttctcaggctccctccttgggggcaa
V7-46_04      gcctggccaagcccccaggacactgatttatgatacaagcaacaaacactcctggacaacctgcccggttctcaggctccctccttgggggcaa
V7-46_05      gcctggccaagcccccaggacactgatttatgatacaagcaacaaacactcctggacaacctgcccggttctcaggctccctccttgggggcaa
V7-46_06      gcctggccaagcccccaggacactgatttatgatacaagcaacaaacactcctggacaacctgcccggttctcaggctccctccttgggggcaa
IGLV7-46*02   gcctggccaagcccccaggacactgatttatgatacaagcaacaaacactcctggacaacctgcccggttctcaggctccctccttgggggcaa
IGLV7-46*01   gcctggccaagcccccaggacactgatttatgatacaagcaacaaacactcctggacaacctgcccggttctcaggctccctccttgggggcaa
IGLV7-46*03   gcctggccaagcccccaggacactgatttatgatacaagcaacaaacactcctggacaacctgcccggttctcaggctccctccttgggggcaa
V_V7-46*01    gcctggccaagcccccaggacactgatttatgatacaagcaacaaacactcctggacaacctgcccggttctcaggctccctccttgggggcaa
V_V7-46*02    gcctggccaagcccccaggacactgatttatgatacaagcaacaaacactcctggacaacctgcccggttctcaggctccctccttgggggcaa
```

```
V7-46_01         agctgccctgaccctttcgggtgcgcagcctgaggatgaggctgagtattactgcttgctctcctatagtggtgctcg-
V7-46_02         agctgccctgacccttttgggtgcgcagcctgaggatgaggctgagtattactgcttgctctcctatagtggtgctcg-
V7-46_03         agctgccctgaccctttcgggtgcgcagcctgaggatgaggctgagtattactgcttgctctcctatagtggtgctcg-
V7-46_04         agctgccctgacccttttgggtgcgcagcctgaggatgaggctgagtattactgcttgctctcctatagtggtgctcg-
V7-46_05         agctgccctgaccctttcgggtgcgcagcctgaggatgaggctgagtattactgcttgctctcctatagtggtgctcg-
V7-46_06         agctgccctgaccctttcgggtgcgcagcctgaggatgaggctgagtattactgcttgctctcctatagtggtgctcg-
IGLV7-46*02      agctgccctgacccttttgggtgcgcagcctgaggatgaggctgagtattactgcttgctctcctatagtggtgctcgg
IGLV7-46*01      agctgccctgaccctttcgggtgcgcagcctgaggatgaggctgagtattactgcttgctctcctatagtggtgctcgg
IGLV7-46*03      agctgccctga-ccctttcgggtgcgcagcctgaggatgaggctgagtattactgcttgctctcctatagtggtgctcgg
V_V7-46*01       agctgccctgaccctttcgggtgcgcagcctgaggatgaggctgagtattactgcttgctctcctatagtggtgct---
V_V7-46*02       agctgccctgacccttttgggtgcgcagcctgaggatgaggctgagtattactgcttgctctcctatagtggtgct---
```

IGLV8-61

```
V8-61_01       atggcctggatgatgcttctcctcggactccttgcttatggatcaggagtggattctcagactgtggtgacccaggagccatcgttctca
V8-61_02       atggcctggatgatgcttctcctcggactccttgcttatggatcaggagtggattctcagactgtggtgacccaggagccatcgttctca
V8-61_03       atggcctggatgatgcttctcctcggactccttgcttatggatcaggagtggattctcagactgtggtgacccaggagccatcgttctca
V8-61_05       atggcctggatgatgcttctcctcggactccttgcttatggatcaggagtggattctcagactgtggtgacccaggagccatcgttctca
IGLV8-61*02    atggcctggatgatgcttctcctcggactccttgcttatggatcaggagtggattctcagactgtggtgacccaggagccatcgttctca
IGLV8-61*01    ---------------------------------------------------------cagactgtggtgacccaggagccatcgttctca
IGLV8-61*03    -----------------------------------------------------------------ggtgacccaggagccatcgttctca
V_V8-61*01     ---------------------------------------------------------cagactgtggtgacccaggagccatcgttctca
V_V8-61*02     ---------------------------------------------------------cagactgtggtgacccaggagccatcgttctca

V8-61_01       gtgtcccctggagggacagtcacactcacttgtggcttgagctctggctcagtctctactagttactaccccagctggtaccagcagacc
V8-61_02       gtgtcccctggagggacagtcacactcacttgtggcttgagctctggctcagtctctactagttactaccccagctggtaccagcagacc
V8-61_03       gtgtcccctggagggacagtcacactcacttgtggcttgagctctggctcagtctctactagttactaccccagctggtaccagcagacc
V8-61_05       gtgtcccctggagggacagtcacactcacttgtggcttgagctctggctcagtctctactagttactaccccagctggtaccagcagacc
IGLV8-61*02    gtgtcccctggagggacagtcacactcacttgtggcttgagctctggctcagtctctactagttactaccccagctggtaccagcagacc
IGLV8-61*01    gtgtcccctggagggacagtcacactcacttgtggcttgagctctggctcagtctctactagttactaccccagctggtaccagcagacc
IGLV8-61*03    gtgtcccctggagggacagtcacactcacttgtggcttgagctctggctcagtctctactagttactaccccagctggtaccagcagacc
V_V8-61*01     gtgtcccctggagggacagtcacactcacttgtggcttgagctctggctcagtctctactagttactaccccagctggtaccagcagacc
V_V8-61*02     gtgtcccctggagggacagtcacactcacttgtggcttgagctctggctcagtctctactagttactaccccagctggtaccagcagacc

V8-61_01       ccaggccaggctccacgcacgctcatctacagcacaaacactcgctcttctgggggtccctgatcgcttctctggctccatccttgggaacaa
V8-61_02       ccaggccaggctccacgcacgctcatctacagcacaaacactcgctcttctgggggtccctgattgcttctctggctccatccttgggaacaa
V8-61_03       ccaggccaggctccacgcacgctcatctacagcacaaacactcgctcttctgggggtccctgatagcttctctggctccatccttgggaacaa
V8-61_05       ccaggccaggctccacgcacgctcatctacagcacaaacactcgctcttctgggggtccctgatcgcttctctggctccatccttgggaacaa
IGLV8-61*02    ccaggccaggctccacgcacgctcatctacagcacaaacactcgctcttctgggggtccctgattgcttctctggctccatccttgggaacaa
IGLV8-61*01    ccaggccaggctccacgcacgctcatctacagcacaaacactcgctcttctgggggtccctgatcgcttctctggctccatccttgggaacaa
IGLV8-61*03    ccaggccaggctccacgcacgctcatctacagcacaaacactcgctcttctggcggtccctgatcgcttctctggctccatccttgggaacaa
V_V8-61*01     ccaggccaggctccacgcacgctcatctacagcacaaacactcgctcttctgggggtccctgatcgcttctctggctccatccttgggaacaa
V_V8-61*02     ccaggccaggctccacgcacgctcatctacagcacaaacactcgctcttctgggggtccctgattgcttctctggctccatccttgggaacaa

V8-61_01       agctgccctcaccatcacgggggcccaggcagatgatgaatctgattattactgtgtgct-----------gtatatgggtagtggcatttc
V8-61_02       agctgccctcaccatcacgggggcccaggcagatgatgaatctgattattactgtgtgct-----------gtatatgggtagtggcatttc
V8-61_03       agctgccctcaccatcacgggggcccaggcagatgatgaatctgattattactgtgtgct-----------gtatatgggtagtggcatttc
V8-61_05       agctgccctcaccatcacgggggcccaggcagatgatgaatctgattattactgtgtgctgtatatgggtagtatatgggtagtggcatttc
IGLV8-61*02    agctgccctcaccatcacgggggcccaggcagatgatgaatctgattattactgtgtgct-----------gtatatgggtagtggcatttc
IGLV8-61*01    agctgccctcaccatcacgggggcccaggcagatgatgaatctgattattactgtgtgct-----------gtatatgggtagtggcatttc
IGLV8-61*03    agctgccctcaccatcacgggggcccaggcagatgatgaa----------------------------------------------------
V_V8-61*01     agctgccctcaccatcacgggggcccaggcagatgatgaatctgattattactgtgtgct-----------gtatatgggtagtggc-----
V_V8-61*02     agctgccctcaccatcacgggggcccaggcagatgatgaatctgattattactgtgtgct-----------gtatatgggtagtggc-----
```

IGLV9-49

```
V9-49_01      atggcctgggctcctctgctcctcaccctcctcagtctcctcacagggtccctctcccagcctgtgctgactcagccaccttctgcatcagcctcc
V9-49_02      atggcctgggctcctctgctcctcaccctcctcagtctcctcacagggtccctctcccagcctgtgctggctcagccaccttctgcatcagcctcc
V9-49_03      atggcctgggctcctctgctcctcaccctcctcagtctcctcacagggtccctctcccagcctgtgctgactcagccaccttctgcatcagcctcc
V9-49_04      atggcctgggctcctctgctcctcaccctcctcagtctcctcacagggtccctctcccagcctgtgctgactcagccaccttctgcatcagcctcc
IGLV9-49*02   atggcctgggctcctctgctcctcaccctcctcagtctcctcacagggtccctctcccagcctgtgctgactcagccaccttctgcatcagcctcc
IGLV9-49*03   atggcctgggctcctctgctcctcaccctcctcagtctcctcacagggtccctctcccagcctgtgctgactcagccaccttctgcatcagcctcc
IGLV9-49*01   ---------------------------------------------------------cagcctgtgctgactcagccaccttctgcatcagcctcc
V_V9-49*01    ---------------------------------------------------------cagcctgtgctgactcagccaccttctgcatcagcctcc
V_V9-49*02    ---------------------------------------------------------cagcctgtgctgactcagccaccttctgcatcagcctcc

V9-49_01      ctgggagcctcggtcacactcacctgcaccctgagcagcggctacagtaattataaagtggactggtaccagcagagaccagggaagggcccccggtttg
V9-49_02      ctgggagcctcggtcacactcacctgcaccctgagcagcggctacagtaattataaagtggactggtaccagcagagaccagggaagggcccccggtttg
V9-49_03      ctgggagcctcggtcacactcacctgcaccctgagcagcggctacagtaattataaagtggactggtaccagcagagaccagggaagggcccccggtttg
V9-49_04      ctgggagcctcggtcacactcacctgcaccctgagcagcggctacagtaattataaagtggactggtaccagcagagaccagggaagggcccccggtttg
IGLV9-49*02   ctgggagcctcggtcacactcacctgcaccctgagcagcggctacagtaattataaagtggactggtaccagcagagaccagggaagggcccccggtttg
IGLV9-49*03   ctgggagcctcggtcacactcacctgcaccctgagcagcggctacagtaattataaagtggactggtaccagcagagaccagggaagggcccccgatttg
IGLV9-49*01   ctgggagcctcggtcacactcacctgcaccctgagcagcggctacagtaattataaagtggactggtaccagcagagaccagggaagggcccccggtttg
V_V9-49*01    ctgggagcctcggtcacactcacctgcaccctgagcagcggctacagtaattataaagtggactggtaccagcagagaccagggaagggcccccggtttg
V_V9-49*02    ctgggagcctcggtcacactcacctgcaccctgagcagcggctacagtaattataaagtggactggtaccagcagagaccagggaagggcccccggtttg

V9-49_01      tgatgcgagtgggcactggtgggattgtgggatccaaggggggatggcatccctgatcgcttctcagtcttgggctcaggcctgaatcggtacc
V9-49_02      tgatgcgagtgggcactggtgggattgtgggatccaaggggggatggcatccctgatcgcttctcagtcttgggctcaggcctgaatcggtacc
V9-49_03      tgatgcgagtgggcactggtgggattgtgggatccaaggggggatggcatccctgatcgcttctcagtcttgggctcaggcctgaatcggtact
V9-49_04      tgatgcgagtgggcactggtgggattgtgggatccaaggggggatggcatccctgatcgcttctcagtcttgggctcaggcctgaatcggtacc
IGLV9-49*02   tgatgcgagtgggcactggtgggattgtgggatccaaggggggatggcatccctgatcgcttctcagtcttgggctcaggcctgaatcggtacc
IGLV9-49*03   tgatgcgagtgggcactggtgggattgtgggatccaaggggggatggcatccctgatcgcttctcagtcttgggctcaggcctgaatcggtacc
IGLV9-49*01   tgatgcgagtgggcactggtgggattgtgggatccaaggggggatggcatccctgatcgcttctcagtcttgggctcaggcctgaatcggtacc
V_V9-49*01    tgatgcgagtgggcactggtgggattgtgggatccaaggggggatggcatccctgatcgcttctcagtcttgggctcaggcctgaatcggtacc
V_V9-49*02    tgatgcgagtgggcactggtgggattgtgggatccaaggggggatggcatccctgatcgcttctcagtcttgggctcaggcctgaatcggtacc

V9-49_01      tgaccatcaagaacatccaggaagaagatgagagtgactaccactgtggggcagaccatggcagtgggagcaacttcgtgtaa--
V9-49_02      tgaccatcaagaacatccaggaagaagatgagagtgactaccactgtggggcagaccatggcagtgggagcaacttcgtgtaa--
V9-49_03      tgaccatcaagaacatccaggaagaagatgagagtgactaccactgtggggcagaccatggcagtgggagcaacttcgtgtaa--
V9-49_04      tgaccatcaagaacatccaggaagaagataagagtgactaccactgtggggcagaccatggcagtgggagcaacttcgtgtaa--
IGLV9-49*02   tgaccatcaagaacatccaggaagaagatgagagtgactaccactgtggggcagaccatggcagtgggagcaacttcgtgtaacc
IGLV9-49*03   tgaccatcaagaacatccaggaagaggatgagagtgactaccactgtggggcagaccatggcagtgggagcaacttcgtgtaacc
IGLV9-49*01   tgaccatcaagaacatccaggaagaggatgagagtgactaccactgtggggcagaccatggcagtgggagcaacttcgtgtaacc
V_V9-49*01    tgaccatcaagaacatccaggaagaggatgagagtgactaccactgtggggcagaccatggcagtggg-----------------
V_V9-49*02    tgaccatcaagaacatccaggaagaagatgagagtgactaccactgtggggcagaccatggcagtggg-----------------
```

IGLV10-54

```
V10-54_01      atgccctgggctctgctcctcctgaccctcctcactcactctgcagtgtcagtggtccaggcagggctgactcagccaccctcggtgtccaa
V10-54_02      atgccctgggctctgctcctcctgaccctcctcactcactctgcagtgtcagtggtccaggcagggctgactcagccaccctcggtgtccaa
V10-54_03      atgccctgggctctgctcctcctgaccctcctcactcactctgcagtgtcagtggtccaggcagggctgactcagccaccctcggtgtccaa
V10-54_04      atgccctgggctctgctcctcctgaccctcctcactcactctgcagtgtcagtggtccaggcagggctgactcagccaccctcggtgtccaa
V10-54_05      atgccctgggctctgctcctcctgaccctcctcactcactctgcagtgtcagtggtccaggcagggctgactcagccaccctcggtgtccaa
V10-54_06      atgccctgggctctgctcctcctgaccctcctcactcactctgcagtgtcagtggtccaggcagggctgactcagccaccctcggtgtccaa
V10-54_07      atgccctgggctctgctcctcctgaccctcctcactcactctgcagtgtcagtggtccaggcagggctgactcagccaccctcagtgtccaa
V10-54_08      atgccctgggctctgctcctcctgaccctcctcactcactctgcagtgtcagtggtccaggcagggctgactcagccaccctcggtgtccaa
V10-54_09      atgccctgggctctgctcctcctgaccctcctcactcactctgcagtgtcagtggtccaggcagggctgactcagccaccctcggtgtccaa
V10-54_10      atgccctgggctctgctcctcctgaccctcctcactcactctgcagtgtcagtggtccaggcagggctgactcagccaccctcggtgtccaa
IGLV10-54*02   atgccctgggctctgctcctcctgaccctcctcactcactctgcagtgtcagtggtccaggcagggctgactcagccaccctcggtgtccaa
IGLV10-54*03   atgccctgggtcatgctcctcctga-aatcctcactcactctgcagtgtcagtggtccaggcagggctgactcagccaccctcggtctccaa
IGLV10-54*01   -----------------------------------------------------------caggcagggctgactcagccaccctcggtgtccaa
V_V10-54*01    -----------------------------------------------------------caggcagggctgactcagccaccctcggtgtccaa
V_V10-54*02    -----------------------------------------------------------caggcagggctgactcagccaccctcggtgtccaa

V10-54_01      gggcttgagacagaccgccacactcacctgcactgggaacagcaacaatgttggcaaccaaggagcagcttggctgcagcagcaccag
V10-54_02      gggcttgagacagaccgccacactcacctgcactgggaacagcaacattgttggcaaccaaggagcagcttggctgcagcagcaccag
V10-54_03      gggcttgagacagaccgccacactcacctgcactgggaacagcaacaatgttggcaaccaaggagcagcttggctgcagcagcaccag
V10-54_04      gggcttgagacagaccgccacactcacctgcactgggaacagcaacattgttggcaaccaaggagcagcttggctgcagcagcaccag
V10-54_05      gggcttgagacagaccgccacactcacctgcactgggaacagcaacaatgctggcaaccaaggagcagcttggctgcagcagcaccag
V10-54_06      gggcttgagacagaccgccacactcacctgcactgggaacagcaacaatgttggcaaccaaggagcagtttggctgcagcagcaccag
V10-54_07      gggcttgagacagaccgccacactcacctgcactgggaacagcaacaatgttggcaaccaaggagcagcttggctgcagcagcaccag
V10-54_08      gggcttgagacagaccgccacactcacctgcactgggaacagcaacaatgttggcaaccaaggagcagcttggctgcagcagcaccag
V10-54_09      gggcttgagacagaccgccacactcacctgcactgggaacagcaacaatgttggcaaccaaggagcagcttggctgcagcagcaccag
V10-54_10      gggcttgagacagaccgccacactcacctgcactgggaacagcaacattgttggcaaccaaggagcagcttggctgcagcagcaccag
IGLV10-54*02   gggcttgagacagaccgccacactcacctgcactgggaacagcaacattgttggcaaccaaggagcagcttggctgcagcagcaccag
IGLV10-54*03   gggcttgagacagaccgccacactcacctgcactgggaacagcaacaatgttggcaaccaaggagcagcttggcctgagcagcaccag
IGLV10-54*01   gggcttgagacagaccgccacactcacctgcactgggaacagcaacaatgttggcaaccaaggagcagcttggctgcagcagcaccag
V_V10-54*01    gggcttgagacagaccgccacactcacctgcactgggaacagcaacaatgttggcaaccaaggagcagcttggctgcagcagcaccag
V_V10-54*02    gggcttgagacagaccgccacactcacctgcactgggaacagcaacattgttggcaaccaaggagcagcttggctgcagcagcaccag
```

```
V10-54_01      ggccaccctcccaaactcctatcctacaggaataacaaccggccctcagggatctcagagagattatctgcatccaggtcaggaaacacagcct
V10-54_02      ggccaccctcccaaactcctatcctacaggaataacaaccggccctcagggatctcagagagattctctgcatccaggtcaggaaacacagcct
V10-54_03      ggccaccctcccaaactcctatcctacaggaataacaaccggccctcagggatctcagagagattctctgcatccaggtcaggaaacacagcct
V10-54_04      ggccaccctcccaaactcctatcctacaggaataacaaccggccctcagggatctcagagagattctctgcatccaggtcaggaaacacagcct
V10-54_05      ggccaccctcccaaactcctatcctacaggaataacaaccggccctcagggatctcagagagattctctgcatccaggtcaggaaacacagcct
V10-54_06      ggccaccctcccaaactcctatcctacaggaataacaaccggccctcagggatctcagagagattatctgcatccaggtcaggaaacacagcct
V10-54_07      ggccaccctcccaaactcctatcctacaggaataacaaccggccctcagggatctcagagagattctctgcatccaggtcaggaaacacagcct
V10-54_08      ggccaccctcccaaactcctatcctacaggaataacaaccggccctcagggatctcagagagattatctgcatccaggtcaggaaacacagcct
V10-54_09      ggccaccctcccaaactcctatcctacaggaataacaaccggccctcagggatctcagagagattatctgcatccaggtcaggaaacacagcct
V10-54_10      ggccaccctcccaaactcctatcctacaggaataacaaccggccctcagggatctcagagagattctctgtatccaggtcaggaaacacagcct
IGLV10-54*02   ggccaccctcccaaactcctatcctacaggaataacaaccggccctcagggatctcagagagattctctgcatccaggtcaggaaacacagcct
IGLV10-54*03   ggccaccctcccaaactcctatcctacaggaataacaaccggccctcagggatctcagagagattatctgcatccaggtcaggaaacacagcct
IGLV10-54*01   ggccaccctcccaaactcctatcctacaggaataacaaccggccctcagggatctcagagagattatctgcatccaggtcaggaaacacagcct
V_V10-54*01    ggccaccctcccaaactcctatcctacaggaataacaaccggccctcagggatctcagagagattatctgcatccaggtcaggaaacacagcct
V_V10-54*02    ggccaccctcccaaactcctatcctacaggaataacaaccggccctcagggatctcagagagattctctgcatccaggtcaggaaacacagcct

V10-54_01      ccctgaccattactggactccagcctgaggacgaggctgactattactgctcagcatgggacagcagcctcagtgctc
V10-54_02      ccctgaccattactggactccagcctgaggacgaggctgactattactgctcagcattggacagcagcctcagtgctc
V10-54_03      ccctgaccattactggactccagcctgaggacgaggctgactattactgctcagcatgggacagcagcctcagtgctc
V10-54_04      ccctgaccattactggactccagcctgaggacgaggctgactattactgctcagcatgggacagcagcctcagtgctc
V10-54_05      ccctgaccattactggactccagcctgaggacgaggctgactattactgctcagcatgggacagcagcctcagtgctc
V10-54_06      ccctgaccattactggactccagcctgaggacgaggctgactattactgctcagcatgggacagcagcctcagtgctc
V10-54_07      ccctgaccattactggactccagcctgaggacgagcctgactattactgctcagcatgggacagcagcctcagtgctc
V10-54_08      ccctgaccattactggactccagcctgaggacgaggctgactattactgctcagcatgggacagcaacctcagtgctc
V10-54_09      ccctgaccattactggactccagcctaaggacgaggctgactattactgctcagcatgggacagcagcctcagtgctc
V10-54_10      ccctgaccattactggactccagcctgaggacgaggctgactattactgctcagcatgggacagcagcctcagtgctc
IGLV10-54*02   ccctgaccattactggactccagcctgaggacgaggctgactattactgctcagcattggacagcagcctcagtgctc
IGLV10-54*03   ccctgaccattactggactccagcctgaggacgaggctgactattactgctcagcatgggacagcagcctcagtgctc
IGLV10-54*01   ccctgaccattactggactccagcctgaggacgaggctgactattactgctcagcatgggacagcagcctcagtgctc
V_V10-54*01    ccctgaccattactggactccagcctgaggacgaggctgactattactgctcagcatgggacagcagcctc-------
V_V10-54*02    ccctgaccattactggactccagcctgaggacgaggctgactattactgctcagcattggacagcagcctc-------
```

IGLV11-55

```
V11-55_01       atggccctgactcctctcctcctcctgctcctctctcactgcacaggttccctctcccggcccgtgctgactcagccgccctctctgtct
V11-55_02       atggccctgactcctctcctcctcctgctcctctctcactgcacaggttccctctcccggcccgtgctgactcagccgccctccctgtct
V11-55_03       atggccctgactcctctcctcctcctgctcctctctcactgcacacgttccctgtcccggcccgtgctgactcagccgccctccctgtct
V11-55_04       atggccctgactcctctcctcctcctgctcctctctcactgcacaggttccctctcccggcccgtgctgactcagccgccctccctgtct
V11-55_05       atggccctgactcctctcctcctcctgctcctctctcactgcacaggttccctctcccggcccgtgctgactcagccgccctccctgtct
IGLV11-55*01    atggccctgactcctctcctcctcctgctcctctctcactgcacaggttccctctcccggcccgtgctgactcagccgccctctctgtct
IGLV11-55*02    atggccctgactcctctcctcctcctgctcctctctcactgcacaggttccctctcccggcccgtgctgactcagccgccctccctgtct
V_V11-55*01     ---------------------------------------------------------cggcccgtgctgactcagccgccctctctgtct

V11-55_01       gcatccccgggagcaacagccagactcccctgcaccctgagcagtgacctcagtgttggtggtaaaaacatgttctggtaccagcagaag
V11-55_02       gcatccccgggagcaacagccagactcccctgcaccctgagcagtgacctcagtgttggtggtaaaaacatgttctggtaccagcagaag
V11-55_03       gcatccccgggagcaacagccagactcccctgcaccctgagcagtgacctcagtgttggtggtaaaaacatgttctggtaccagcagaag
V11-55_04       gcatccccgggagcaacagccagactcccctgcaccctgagcagtgacctcagtgttggtggtaaaaacatgttctggtaccagcagaag
V11-55_05       gcatccccgggagcaacagccagactcccctgcaccctgagcagtgacctcagtgttggtggtaaaaacatgttctggtaccagcagaag
IGLV11-55*01    gcatccccgggagcaacagccagactcccctgcaccctgagcagtgacctcagtgttggtggtaaaaacatgttctggtaccagcagaag
IGLV11-55*02    gcatccccgggagcaacagccagactcccctgcaccctgagcagtgacctcagtgttggtggtaaaaacatgttctggtaccagcagaag
V_V11-55*01     gcatccccgggagcaacagccagactcccctgcaccctgagcagtgacctcagtgttggtggtaaaaacatgttctggtaccagcagaag

V11-55_01       ccagggagctctcccaggttattcctgtatcactactcagactcagacaagcagctgggacctgggtccccagtcgagtctctggctcca
V11-55_02       ctagggagctctcccaggttattcctgtatcactactcagactcagacaagcagctgggacctgggtccccagtcgagtctctggctcca
V11-55_03       ctagggagctctcccaggttattcctgtatcactactcagactcagacaagcagctgggacctgggtccccagtcgagtctctggctcca
V11-55_04       ctagggagctctcccaggttattcctgtatcactactcagactcagacaagcagctgggacctgggtccccagtcgagtctctggctcca
V11-55_05       ctagggagctctcccaggttattcctgtatcactactcagactcagacaagcagctgggacctggtgtccccagtcgagtctctggctcca
IGLV11-55*01    ccagggagctctcccaggttattcctgtatcactactcagactcagacaagcagctgggacctgggtccccagtcgagtctctggctcca
IGLV11-55*02    ctagggagctctcccaggttattcctgtatcactactcagactcagacaagcagctgggacctgggtccccagtcgagtctctggctcca
V_V11-55*01     ccagggagctctcccaggttattcctgtatcactactcagactcagacaagcagctgggacctgggtccccagtcgagtctctggctcca

V11-55_01       aggagacctcaagtaacacagcgtttttgctcatctctgggctccagcctgaggacgaggccgattattactgccaggtgtacgaaagt
V11-55_02       aggagacctcaagtaacacagcgtttttgctcatctctgggctccagcctgaggacgaggccgattattactgccaggtgtacgaaagt
V11-55_03       aggagacctcaagtaacacagcgtttttgctcatctctgggctccagcctgaggacgaggccgattattactgccaggtgtacgaaagt
V11-55_04       aggagacctcaagtaacacagtgtttttgctcatctctgggctccagcctgaggacgaggccgattattactgccaggtgtacgaaagt
V11-55_05       aggagacctcaagtaacacagcgtttttgctcatctctgggctccagcctgaggacgaggccgattattactgccaggtgtacgaaagt
IGLV11-55*01    aggagacctcaagtaacacagcgtttttgctcatctctgggctccagcctgaggacgaggccgattattactgccaggtgtacgaaagt
IGLV11-55*02    aggagacctcaagtaacacagcgtttttgctcatctctgggctccagcctgaggacgaggccgattattactgccaggtgtacgaaagt
V_V11-55*01     aggagacctcaagtaacacagcgtttttgctcatctctgggctccagcctgaggacgaggccgattattactgccaggtgtacgaaagt
```

```
V11-55_01        agtgctaatcacagtgagacagatgaggaagtcggacaaaaaccaaggttttaa
V11-55_02        agtgctaatcacagtgagacagatgaggaagtcggacaaaaaccaaggttttaa
V11-55_03        agtgctaatcacagtgagacagatgaggaagtcggacaaaaaccaaggttttaa
V11-55_04        agtgctaatcacagtgagacagatgaggaagtcggacaaaaaccaaggttttaa
V11-55_05        agtgctaatcacagtgagacagatgaggaagtcggacaaaaaccaaggttttaa
IGLV11-55*01     agtgctaat---------------------------------------------
IGLV11-55*02     agtgctaat---------------------------------------------
V_V11-55*01      agtgct------------------------------------------------
                 ******
```

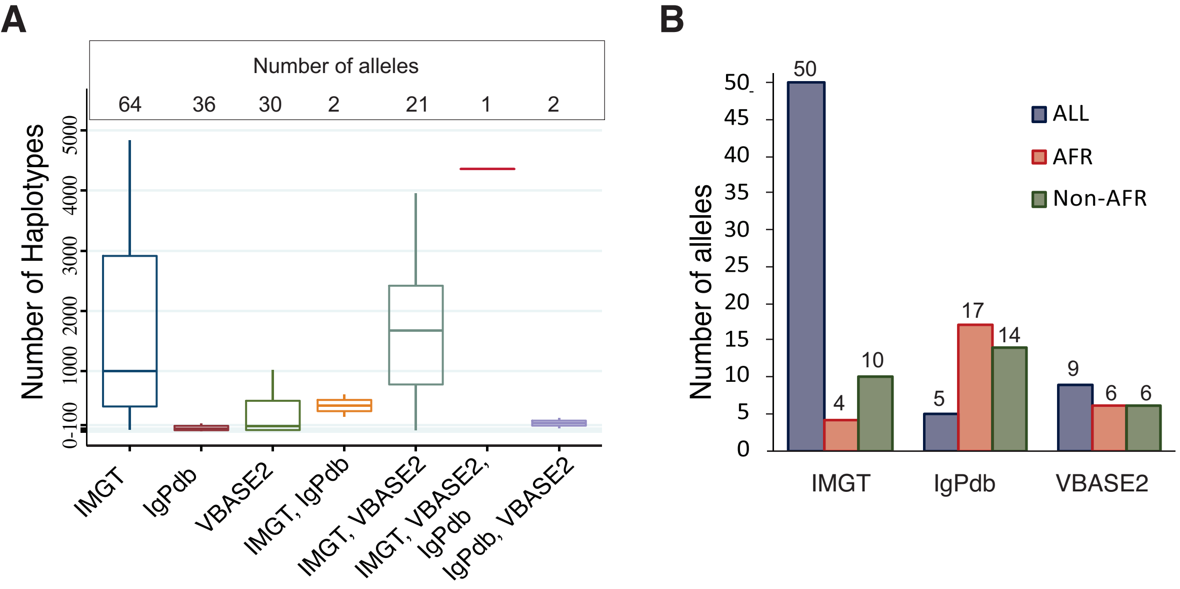

Figure S4: The known alleles are frequent and are present in all the ethnicities. A) Haplotypes support of the alleles mapped to the existing databases. Each dot is an allele. The alleles mapped to the alleles in multiple resources are also indicated. **B) The population distribution of the AS1 (known) alleles.** The alleles shared between IMGT and other resources are considered to be an IMGT alleles. Similarly, alleles are considered to be belonging to IgPdb if mapped to IgPdb and VBASE2 databases. IgPdb and VBASE2 harbor alleles unique to specific populations.

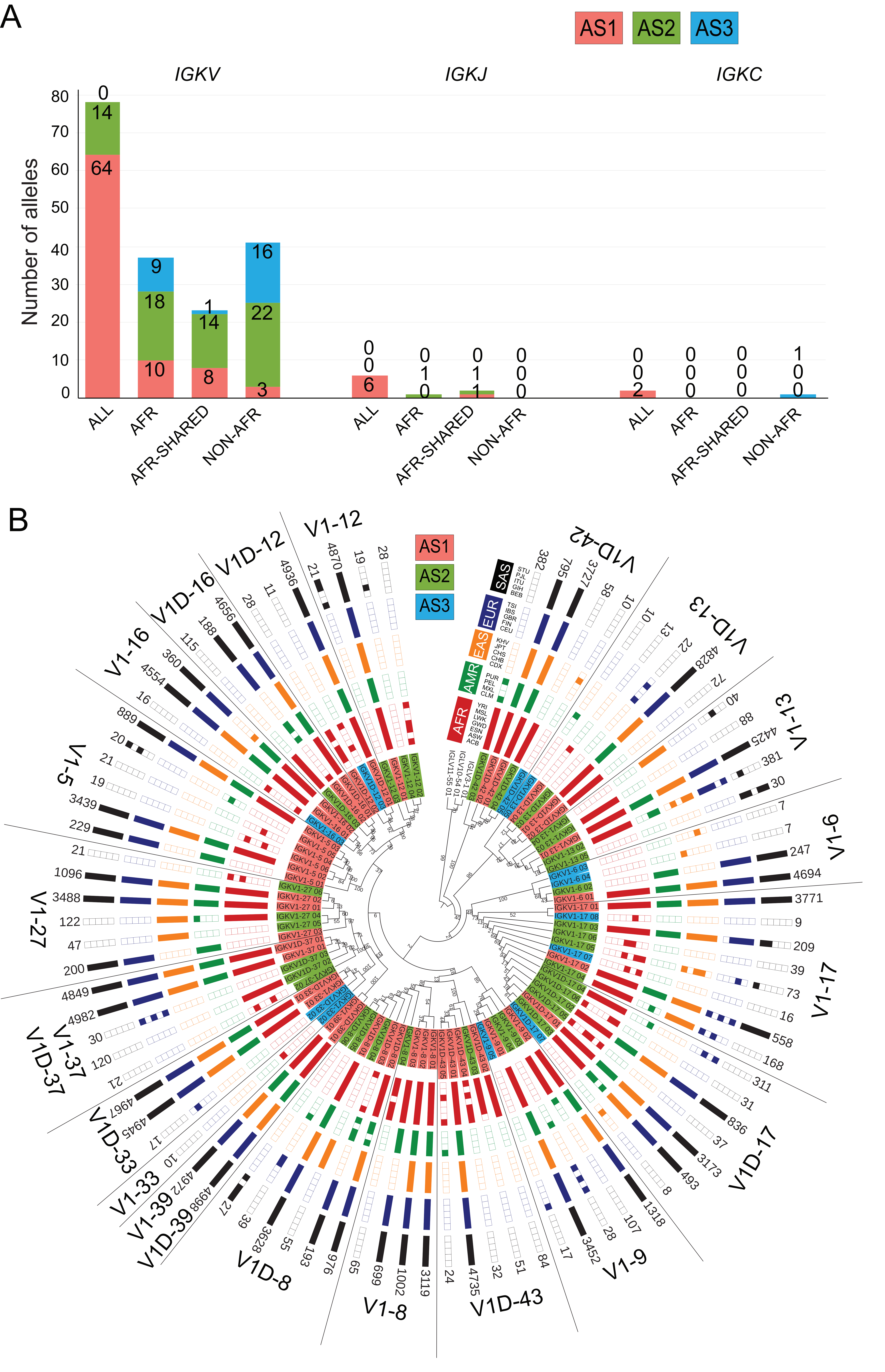

Figure S5: Population distribution of alleles in *IGK* light chain locus. A) The superpopulation distribution of *VJ* genes and *C* genes for *IGK* locus. The alleles are categorized based on the populations i.e. “ALL” represents alleles present in all the superpopulations, “AFR” category comprises of alleles present only in African populations, “AFR Shared” comprises of the alleles present in African populations and are shared with one of the other superpopulations and ‘Non-AFR where alleles are present in one of the populations other than Africans. **B) Maximum Likelihood tree of the population distribution of *IGKV1* family alleles.** For the population distribution of other *IGKV* families refer to Figure S7. A few *IGLV* alleles were used as outgroups.

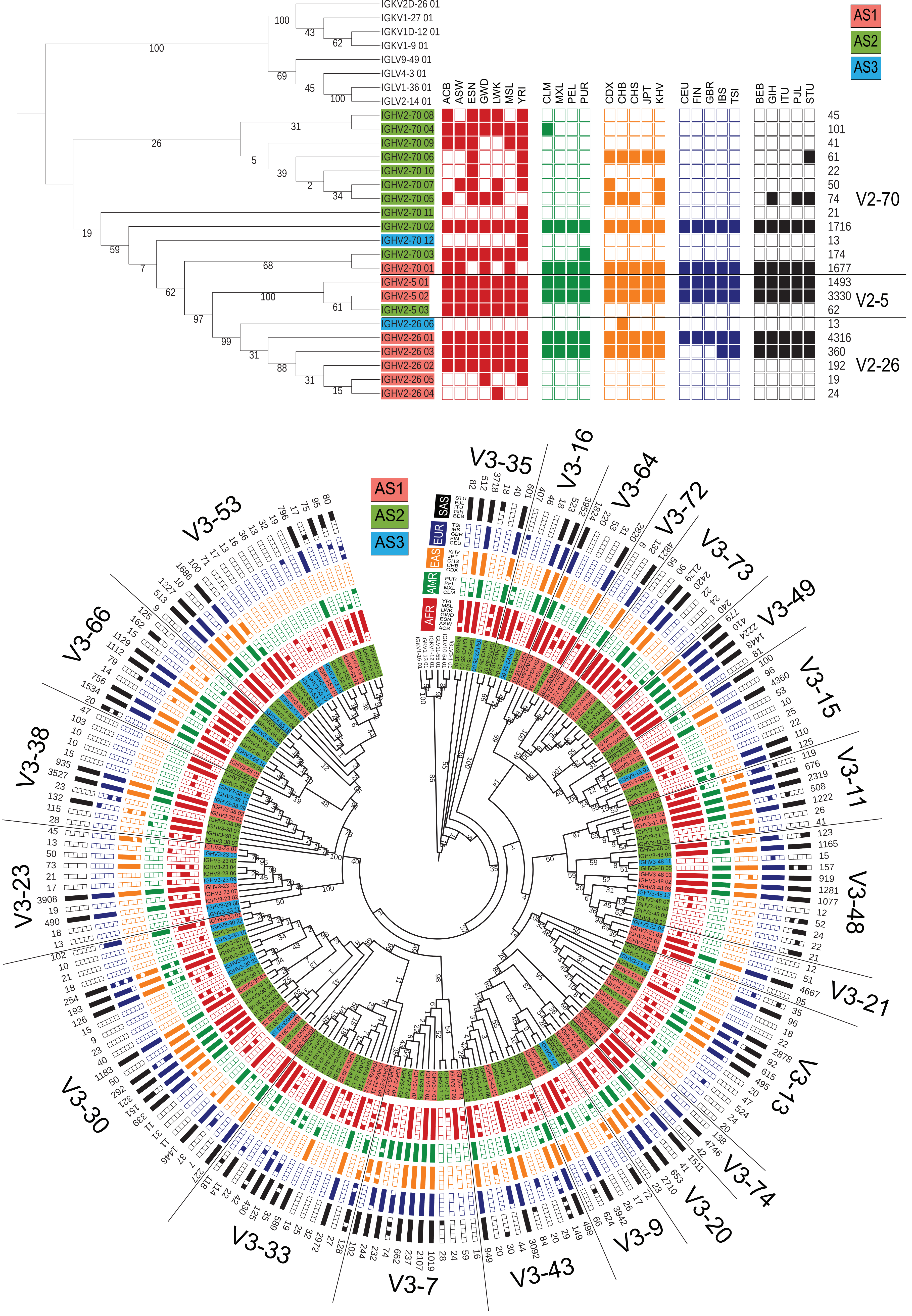

Figure S6: ML tree of the population distribution of IGHV2 and V3 family alleles. A few IGKV alleles were used as outgroups.

IGHA1 and IGHA2

IGHA1_IMGT	gcatccccgaccagccccaaggtcttcccgctgagcctctgcagcacccagccagatggg
IGHA1_GRCh37	gcatccccgaccagccccaaggtcttcccgctgagcctctgcagcacccagccagatggg
IGHA1_01	gcatccccgaccagccccaaggtcttcccgctgagcctctgcagcacccagccagatggg
IGHA1_02	gcatccccgaccagccccaaggtcttcccgctgagcctctgcagcacccagccagatggg
IGHA1_03	gcatccccgaccagccccaaggtcttcccgctgagcctctgcagcacccagccagatggg
IGHA1_04	gcatccccgaccagccccaaggtcttcccgctgagcctctgcagcacccagccagatggg
IGHA1_05	gcatccccgaccagccccaaggtcttcccgctgagcctctgcagcacccagccagatggg
IGHA1_06	gcatccccgaccagccccaaggtcttcccgctgagcctctgcagcacccagccagatggg
IGHA1_07	gcatccccgaccagccccaaggtcttcccgctgagcctctgcagcacccagccagatggg
IGHA1_08	gcatccccgaccagccccaaggtcttcccgctgagcctctgcagcacccagccagatggg
IGHA1_09	gcatccccgaccagccccaaggtcttcccgctgagcctctgcagcacccagccagatggg
IGHA1_10	gcatccccgaccagccccaaggtcttcccgctgagcctctgcagcacccagccagatggg
IGHA1_11	gcatccccgaccagccccaaggtcttcccgctgagcctctgcagcacccagccagatggg
IGHA1_12	gcatccccgaccagccccaaggtcttcccgctgagcctctgcagcacccagccagatggg
IGHA1_13	gcatccccgaccagccccaaggtcttcccgctgagcctctgcagcacccagccagatggg
IGHA1_14	gcatccccgaccagccccaaggtcttcccgctgagcctctgcagcacccagccagatggg
IGHA1_15	gcatccccgaccagccccgaggtcttcccgctgagcctctgcagcacccagccagatggg
IGHA1_16	gcatccccgaccagccccaaggtcttcccgctgagcctctgcagcacccagccagatggg
IGHA1_17	gcatccccgaccagccccaaggtcttcccgctgagcctctgcagcacccagccagatggg
IGHA1_18	gcatccccgaccagccccaaggtcttcccgctgagcctctgcagcacccagccagatggg
IGHA1_19	gcatccccgaccagccccgaggtcttcccgctgagcctctgcagcacccagccagatggg
IGHA1_20	gcatccccgaccagccccaaggtcttcccgctgagcctctgcagcacccagccagatggg
IGHA1_21	gcatccccgaccagccccgaggtcttcccgctgagcctctgcagcacccagccagatggg
IGHA1_22	gcatccccgaccagccccaaggtcttcccgctgagcctctgcagcacccagccagatggg
IGHA2_IMGT	gcatccccgaccagccccaaggtcttcccgctgagcctcgacagcaccccccaagatggg
IGHA2_GRCh37	gcatccccgaccagccccaaggtcttcccgctgagcctcgacagcaccccccaagatggg
IGHA2_01	gcatccccgaccagccccaaggtcttcccgctgagcctcgacagcaccccccaagatggg
IGHA2_02	gcatccccgaccagccccaaggtcttcccgctgagcctcgacagcaccccccaagatggg
IGHA2_03	gcatccccgaccagccccaaggtcttcccgctgagcctcgacagcaccccccaagatggg
IGHA2_04	gcatccccgaccagccccaaggtcttcccgctgagcctcgacagcaccccccaagatggg
IGHA2_05	gcatccccgaccagccccaaggtcttcccgctgagcctcgacagcaccccccaagatggg
IGHA2_06	gcatccccgaccagccccaaggtcttcccgctgagcctcgacagcaccccccaagatggg
IGHA2_07	gcatccccgaccagccccaaggtcttcccgctgagcctcgacagcaccccccaagatggg
IGHA2_08	gcatccccgaccagccccaaggtcttcccgctgagcctcgacagcaccccccaagatggg
IGHA2_09	gcatccccgaccagccccaaggtcttcccgctgagcctcgacagcaccccccaagatggg
IGHA2_10	gcatccccgaccagccccaaggtcttcccgctgagcctcgacagcaccccccaagatggg
IGHA2_11	gcatccccgaccagccccaaggtcttcccgctgagcctcgacagcaccccccaagatggg
IGHA2_12	gcatccccgaccagccccaaggtcttcccgctgagcctcgacagcaccccccaagatggg
IGHA2_13	gcatccccgaccagccccaaggtcttcccgctgagcctcgacagcaccccccaagatggg
IGHA2_14	gcatccccgaccagccccaaggtcttcccgctgagcctcgacagcaccccccaagatggg
IGHA2_15	gcatccccgaccagccccaaggtcttcccgctgagcctcgacagcaccccccaagatggg
IGHA2_16	gcatccccgaccagccccaaggtcttcccgctgagcctcgacagcaccccccaagatggg
IGHA2_17	gcatccccgaccagccccaaggtcttcccgctgagcctcgacagcaccccccaagatggg
IGHA2_18	gcatccccgaccagccccaaggtcttcccgctgagcctcgacagcaccccccaagatggg
IGHA2_19	gcatccccgaccagccccaaggtcttcccgctgagcctcgacagcaccccccaagatggg
IGHA2_20	gcatccccgaccagccccaaggtcttcccgctgagcctcgacagcaccccccaagatggg
IGHA2_21	gcatccccgaccagccccaaggtcttcccgctgagcctcgacagcaccccccaagatggg
IGHA2_22	gcatccccgaccagccccaaggtcttcccgctgagcctcgacagcaccccccaagatggg
IGHA2_23	gcatccccgaccagccccaaggtcttcccgctgagcctcgacagcaccccccaagatggg
IGHA2_24	gcatccccgaccagccccaaggtcttcccgctgagcctcgacagcaccccccaagatggg
IGHA2_25	gcatccccgaccagccccaaggtcttcccgctgagcctcgacagcaccccccaagatggg
IGHA2_26	gcatccccgaccagccccaaggtcttcccgctgagcctcgacagcaccccccaagatggg

```
IGHA1_IMGT      aacgtggtcatcgcctgcctggtccagggcttcttcccccaggagccactcagtgtgacc
IGHA1_GRCh37    aacgtggtcatcgcctgcctggtccagggcttcttcccccaggagccactcagtgtgacc
IGHA1_01        aacgtggtcatcgcctgcctggtccagggcttcttcccccaggagccactcagtgtgacc
IGHA1_02        aacgtggtcatcgcctgcctggtccagggcttcttcccccaggagccactcagtgtgacc
IGHA1_03        aacgtggtcatcgcctgcctggtccagggcttcttcccccaggagccactcagtgtgacc
IGHA1_04        aacgtggtcatcgcctgcctggtccagggcttcttcccccaggagccactcagtgtgacc
IGHA1_05        aacgtggtcatcgcctgcctggtccagggcttcttcccccaggagccactcagtgtgacc
IGHA1_06        aacgtggtcatcgcctgcctggtccagggcttcttcccccaggagccactcagtgtgacc
IGHA1_07        aacgtggtcatcgcctgcctggtccagggcttcttcccccaggagccactcagtgtgacc
IGHA1_08        aacgtggtcatcgcctgcctggtccagggcttcttcccccaggagccactcagtgtgacc
IGHA1_09        aacgtggtcatcgcctgcctggtccagggcttcttcccccaggagccactcagtgtgacc
IGHA1_10        aacgtggtcatcgcctgcctggtccagggcttcttcccccaggagccactcagtgtgacc
IGHA1_11        aacgtggtcatcgcctgcctggtccagggcttcttcccccaggagccactcagtgtgacc
IGHA1_12        aacgtggtcatcgcctgcctggtccagggcttcttcccccaggagccactcagtgtgacc
IGHA1_13        aacgtggtcatcgcctgcctggtccagggcttcttcccccaggagccactcagtgtgacc
IGHA1_14        aacgtggtcatcgcctgcctggtccagggcttcttcccccaggagccactcagtgtgacc
IGHA1_15        aacgtggtcattgcctgcctggtccagggcttcttcccccaggagccactcagtgtgacc
IGHA1_16        aacgtggtcatcgcctgcctggtccagggcttcttcccccaggagccactcagtgtgacc
IGHA1_17        aacgtggtcatcgcctgcctggtccagggcttcttcccccaggagccactcagtgtgacc
IGHA1_18        aacgtggtcatcgcctgcctggtccagggcttcttcccccaggagccactcagtgtgacc
IGHA1_19        aacgtggtcattgcctgcctggtccagggcttcttcccccaggagccactcagtgtgacc
IGHA1_20        aacgtggtcattgcctgcctggtccagggcttcttcccccaggagccactcagtgtgacc
IGHA1_21        aacgtggtcattgcctgcctggtccagggcttcttcccccaggagccactcagtgtgacc
IGHA1_22        aacgtggtcattgcctgcctggtccagggcttcttcccccaggagccactcagtgtgacc
IGHA2_IMGT      aacgtggtcgtcgcatgcctggtccagggcttcttcccccaggagccactcagtgtgacc
IGHA2_GRCh37    aacgtggtcgtcgcatgcctggtccagggcttcttcccccaggagccactcagtgtgacc
IGHA2_01        aacgtggtcgtcgcatgcctggtccagggcttcttcccccaggagccactcagtgtgacc
IGHA2_02        aacgtggtcgtcgcatgcctggtccagggcttcttcccccaggagccactcagtgtgacc
IGHA2_03        aacgtggtcgtcgcatgcctggtccagggcttcttcccccaggagccactcagtgtgacc
IGHA2_04        aacgtggtcgtcgcatgcctggtccagggcttcttcccccaggagccactcagtgtgacc
IGHA2_05        aacgtggtcgtcgcatgcctggtccagggcttcttcccccaggagccactcagtgtgacc
IGHA2_06        aacgtggtcgtcgcatgcctggtccagggcttcttcccccaggagccactcagtgtgacc
IGHA2_07        aacgtggtcgtcgcatgcctggtccagggcttcttcccccaggagccactcagtgtgacc
IGHA2_08        aacgtggtcgtcgcatgcctggtccagggcttcttcccccaggagccactcagtgtgacc
IGHA2_09        aacgtggtcgtcgcatgcctggtccagggcttcttcccccaggagccactcagtgtgacc
IGHA2_10        aacgtggtcgtcgcatgcctggtccagggcttcttcccccaggagccactcagtgtgacc
IGHA2_11        aacgtggtcgtcgcatgcctggtccagggcttcttcccccaggagccactcagtgtgacc
IGHA2_12        aacgtggtcgtcgcatgcctggtccagggcttcttcccccaggagccactcagtgtgacc
IGHA2_13        aacgtggtcgtcgcatgcctggtccagggcttcttcccccaggagccactcagtgtgacc
IGHA2_14        aacgtggtcgtcgcatgcctggtccagggcttcttcccccaggagccactcagtgtgacc
IGHA2_15        aacgtggtcgtcgcatgcctggtccagggcttcttcccccaggagccactcagtgtgacc
IGHA2_16        aacgtggtcgtcgcatgcctggtccagggcttcttcccccaggagccactcagtgtgacc
IGHA2_17        aacgtggtcgtcgcatgcctggtccagggcttcttcccccaggagccactcagtgtgacc
IGHA2_18        aacgtggtcgtcgcatgcctggtccagggcttcttcccccaggagccactcagtgtgacc
IGHA2_19        aacgtggtcgtcgcatgcctggtccagggcttcttcccccaggagccactcagtgtgacc
IGHA2_20        aacgtggtcgtcgcatgcctggtccagggcttcttcccccaggagccactcagtgtgacc
IGHA2_21        aacgtggtcgtcgcatgcctggtccagggcttcttcccccaggagccactcagtgtgacc
IGHA2_22        aacgtggtcgtcgcatgcctggtccagggcttcttcccccaggagccactcagtgtgacc
IGHA2_23        aacgtggtcgtcgcatgcctggtccagggcttcttcccccaggagccactcagtgtgacc
IGHA2_24        aacgtggtcgtcgcatgcctggtccagggcttcttcccccaggagccactcagtgtgacc
IGHA2_25        aacgtggtcgtcgcatgcctggtccagggcttcttcccccaggagccactcagtgtgacc
IGHA2_26        aacgtggtcgtcgcatgcctggtccagggcttcttcccccaggagccactcagtgtgacc
```

IGHA1_IMGT	tggagcgaaagcggacaggg cgtgaccgccagaaacttcccaccc agccaggatgcctcc
IGHA1_GRCh37	tggagcgaaagcggacaggg cgtgaccgccagaaacttcccaccc agccaggatgcctcc
IGHA1_01	tggagcgaaagcggacaggg cgtgaccgccagaaacttcccaccc agccaggatgcctcc
IGHA1_02	tggagcgaaagcggacaggg cgtgaccgccagaaacttcccaccc agccaggatgcctcc
IGHA1_03	tggagcgaaagcggacaggg cgtgaccgccagaaacttcccaccc agccaggatgcctcc
IGHA1_04	tggagcgaaagcggacaggg cgtgaccgccagaaacttcccaccc agccaggatgcctcc
IGHA1_05	tggagcgaaagcggacaggg cgtgaccgccagaaacttcccaccc agccaggatgcctcc
IGHA1_06	tggagcgaaagcggacaggg cgtgaccgccagaaacttcccaccc agccaggatgcctcc
IGHA1_07	tggagcgaaagcggacaggg cgtgaccgccagaaacttcccaccc agccaggatgcctcc
IGHA1_08	tggagcgaaagcggacaggg cgtgaccgccagaaacttcccaccc agccaggatgcctcc
IGHA1_09	tggagcgaaagcggacaggg cgtgaccgccagaaacttcccaccc agccaggatgcctcc
IGHA1_10	tggagcgaaagcggacaggg cgtgaccgccagaaacttcccaccc agccaggatgcctcc
IGHA1_11	tggagcgaaagcggacaggg cgtgaccgccagaaacttcccaccc agccaggatgcctcc
IGHA1_12	tggagcgaaagcggacaggg cgtgaccgccagaaacttcccaccc agccaggatgcctcc
IGHA1_13	tggagcgaaagcggacaggg cgtgaccgccagaaacttcccaccc agccaggatgcctcc
IGHA1_14	tggagcgaaagcggacaggg cgtgaccgccagaaacttcccaccc agccaggatgcctcc
IGHA1_15	tggagcgaaagcggacaggg cgtgaccgccagaaacttcccaccc agccaggatgcctcc
IGHA1_16	tggagcgaaagcggacaggg cgtgaccgccagaaacttcccaccc agccaggatgcctcc
IGHA1_17	tggagcgaaagcggacaggg cgtgaccgccagaaacttcccaccc agccaggatgcctcc
IGHA1_18	tggagcgaaagcggacaggg cgtgaccgccagaaacttcccaccc agccaggatgcctcc
IGHA1_19	tggagcgaaagcggacaggg cgtgaccgccagaaacttcccaccc agccaggatgcctcc
IGHA1_20	tggagcgaaagcggacaggg cgtgaccgccagaaacttcccaccc agccaggatgcctcc
IGHA1_21	tggagcgaaagcggacaggg cgtgaccgccagaaacttcccaccc agccaggatgcctcc
IGHA1_22	tggagcgaaagcggacaggg cgtgaccgccagaaacttcccaccc agccaggatgcctcc
IGHA2_IMGT	tggagcgaaagcggacagaa cgtgaccgccagaaacttcccacct agccaggatgcctcc
IGHA2_GRCh37	tggagcgaaagcggacagaa cgtgaccgccagaaacttcccacct agccaggatgcctcc
IGHA2_01	tggagcgaaagcggacagaa cgtgaccgccagaaacttcccacct agccaggatgcctcc
IGHA2_02	tggagcgaaagcggacagaa cgtgaccgccagaaacttcccacct agccaggatgcctcc
IGHA2_03	tggagcgaaagcggacagaa cgtgaccgccagaaacttcccaccc agccaggatgcctcc
IGHA2_04	tggagcgaaagcggacagaa cgtgaccgccagaaacttcccacct agccaggatgcctcc
IGHA2_05	tggagcgaaagcggacagaa cgtgaccgccagaaacttcccacct agccaggatgcctcc
IGHA2_06	tggagcgaaagcggacagaa cgtgaccgccagaaacttcccacct agccaggatgcctcc
IGHA2_07	tggagcgaaagcggacagaa cgtgaccgccagaaacttcccaccc agccaggatgcctcc
IGHA2_08	tggagcgaaagcggacagaa cgtgaccgccagaaacttcccacct agccaggatgcctcc
IGHA2_09	tggagcgaaagcggacagaa cgtgaccgccagaaacttcccacct agccaggatgcctcc
IGHA2_10	tggagcgaaagcggacagaa cgtgaccgccagaaacttcccacct agccaggatgcctcc
IGHA2_11	tggagcgaaagcggacagaa cgtgaccgccagaaacttcccacct agccaggatgcctcc
IGHA2_12	tggagcgaaagcggacagaa cgtgaccgccagaaacttcccacct agccaggatgcctcc
IGHA2_13	tggagcgaaagcggacagaa cgtgaccgccagaaacttcccacct agccaggatgcctcc
IGHA2_14	tggagcgaaagcggacagaa cgtgaccgccagaaacttcccacct agccaggatgcctcc
IGHA2_15	tggagcgaaagcggacagaa cgtgaccgccagaaacttcccacct agccaggatgcctcc
IGHA2_16	tggagcgaaagcggacagaa cgtgaccgccagaaacttcccacct agccaggatgcctcc
IGHA2_17	tggagcgaaagcggacagaa cgtgaccgccagaaacttcccacct agccaggatgcctcc
IGHA2_18	tggagcgaaagcggacagaa cgtgaccgccagaaacttcccacct agccaggatgcctcc
IGHA2_19	tggagcgaaagcggacagaa cgtgaccgccagaaacttcccaccc agccaggatgcctcc
IGHA2_20	tggagcgaaagcggacagaa cgtgaccgccagaaacttcccacct agccaggatgcctcc
IGHA2_21	tggagcgaaagcggacagaa cgtgaccgccagaaacttcccacct agccaggatgcctcc
IGHA2_22	tggagcgaaagcggacagaa cgtgaccgccagaaacttcccacct agccaggatgcctcc
IGHA2_23	tggagcgaaagcggacagaa cgtgaccgccagaaacttcccaccc agccaggatgcctcc
IGHA2_24	tggagcgaaagcggacagaa cgtgaccgccagaaacttcccacct agccaggatgcctcc
IGHA2_25	tggagcgaaagcggacagaa cgtgaccgccagaaacttcccacct agccaggatgcctcc
IGHA2_26	tggagcgaaagcggacagaa cgtgaccgccagaaacttcccacct agccaggatgcctcc

IGHA1_IMGT	ggggacctgtacaccacgagcagccagctgaccctgccggccacacagtgcctagccggc
IGHA1_GRCh37	ggggacctgtacaccacgagcagccagctgaccctgccggccacacagtgcctagccggc
IGHA1_01	ggggacctgtacaccacgagcagccagctgaccctgccggccacacagtgcctagccggc
IGHA1_02	ggggacctgtacaccacgagcagccagctgaccctgccggccacacagtgcctagccggc
IGHA1_03	ggggacctgtacaccacgagcagccagctgaccctgccggccacacagtgcctagccggc
IGHA1_04	ggggacctgtacaccacgagcagccagctgaccctgccggccacacagtgcctagccggc
IGHA1_05	ggggacctgtacaccacgagcagccagctgaccctgccggccacacagtgcctagccggc
IGHA1_06	ggggacctgtacaccacgagcagccagctgaccctgccggccacacagtgcctagccggc
IGHA1_07	ggggacctgtacaccacgagcagccagctgaccctgccggccacacagtgcctagccggc
IGHA1_08	ggggacctgtacaccacgagcagccaactgaccctgccggccacacagtgcctagccggc
IGHA1_09	ggggacctgtacaccacgagcagccaactgaccctgccggccacacagtgcctagccggc
IGHA1_10	ggggacctgtacaccacgagcagccagctgaccctgccggccacacagtgcctagccggc
IGHA1_11	ggggacctgtacaccacgagcagccaactgaccctgccggccacacagtgcctagccggc
IGHA1_12	ggggacctgtacaccacgagcagccagctgaccctgccggccacacagtgcctagccggc
IGHA1_13	ggggacctgtacaccacgagcagccagctgaccctgccggccacacagtgcctagccggc
IGHA1_14	ggggacctgtacaccacgagcagccagctgaccctgccggccacacagtgcctagccggc
IGHA1_15	ggggacctgtacaccacgagcagtcaactgaccctgccggccacacagtgcctagccggc
IGHA1_16	ggggacctgtacaccacgagcagccagctgaccctgccggccacacagtgcctagccggc
IGHA1_17	ggggacctgtacaccacgagcagccagctgaccctgccggccacacagtgcctagccggc
IGHA1_18	ggggacctgtacaccacgagcagccagctgaccctgccggccacacagtgcctagccggc
IGHA1_19	ggggacctgtacaccacgagcagtcaactgaccctgccggccacacagtgcctagccggc
IGHA1_20	ggggacctgtacaccacgagcagtcaactgaccctgccggccacacagtgcctagccggc
IGHA1_21	ggggacctgtacaccacgagcagtcaactgaccctgccggccacacagtgcctagccggc
IGHA1_22	ggggacctgtacaccacgagcagccaactgaccctgccggccacacagtgcctagccggc
IGHA2_IMGT	ggggacctgtacaccacgagcagccagctgaccctgccggccacacagtgcccagacggc
IGHA2_GRCh37	ggggacctgtacaccacgagcagccagctgaccctgccggccacacagtgcccagacggc
IGHA2_01	ggggacctgtacaccacgagcagccagctgaccctgccggccacacagtgcccagacggc
IGHA2_02	ggggacctgtacaccacgagcagccagctgaccctgccggccacacagtgcccagacggc
IGHA2_03	ggggacctgtacaccacgagcagccagctgaccctgccggccacacagtgcccagacggc
IGHA2_04	ggggacctgtacaccacgagcagccagctgaccctgccggccacacagtgcccagacggc
IGHA2_05	ggggacctgtacaccacgagcagccagctgaccctgccggccacacagtgcccagacggc
IGHA2_06	ggggacctgtacaccacgagcagccagctgaccctgccggccacacagtgcccagacggc
IGHA2_07	ggggacctgtacaccacgagcagccagctgaccctgccggccacacagtgcccagacggc
IGHA2_08	ggggacctgtacaccacgagcagccagctgaccctgccggccacacagtgcccagacggc
IGHA2_09	ggggacctgtacaccacgagcagccagctgaccctgccggccacacagtgcccagacggc
IGHA2_10	ggggacctgtacaccacgagcagccagctgaccctgccggccacacagtgcccagacggc
IGHA2_11	ggggacctgtacaccacgagcagccagctgaccctgccggccacacagtgcccagacggc
IGHA2_12	ggggacctgtacaccacgagcagccagctgaccctgccggccacacagtgcccagacggc
IGHA2_13	ggggacctgtacaccacgagcagccagctgaccctgccggccacacagtgcccagacggc
IGHA2_14	ggggacctgtacaccacgagcagccagctgaccctgccggccacacagtgcccagacggc
IGHA2_15	ggggacctgtacaccacgagcagccagctgaccctgccggccacacagtgcccagacggc
IGHA2_16	ggggacctgtacaccacgagcagccagctgaccctgccggccacacagtgcccagacggc
IGHA2_17	ggggacctgtacaccacgagcagccagctgaccctgccggccacacagtgcccagacggc
IGHA2_18	ggggacctgtacaccacgagcagccagctgaccctgccggccacacagtgcccagacggc
IGHA2_19	ggggacctgtacaccacgagcagccagctgaccctgccggccacacagtgcccagacggc
IGHA2_20	ggggacctgtacaccacgagcagccagctgaccctgccggccacacagtgcccagacggc
IGHA2_21	ggggacctgtacaccacgagcagccagctgaccctgccggccacacagtgcccagacggc
IGHA2_22	ggggacctgtacaccacgagcagccagctgaccctgccggccacacagtgcccagacggc
IGHA2_23	ggggacctgtacaccacgagcagccagctgaccctgccggccacacagtgcccagacggc
IGHA2_24	ggggacctgtacaccacgagcagccagctgaccctgccggccacacagtgcccagacggc
IGHA2_25	ggggacctgtacaccacgagcagccagctgaccctgccggccacacagtgcccagacggc
IGHA2_26	ggggacctgtacaccacgagcagccagctgaccctgccggccacacagtgcccagacggc

IGHA1_IMGT	aagtccgtgacatgccacgtgaagcactacacgaatcccagccaggatgtgactgtgccc
IGHA1_GRCh37	aagtccgtgacatgccacgtgaagcactacacgaatcccagccaggatgtgactgtgccc
IGHA1_01	aagtccgtgacatgccacgtgaagcactacacgaatcccagccaggatgtgactgtgccc
IGHA1_02	aagtccgtgacatgccacgtgaagcactacacgaatcccagccaggatgtgactgtgccc
IGHA1_03	aagtccgtgacatgccacgtgaagcactacacgaatcccagccaggatgtgactgtgccc
IGHA1_04	aagtccgtgacatgccacgtgaagcactacacgaatcccagccaggatgtgactgtgccc
IGHA1_05	aagtccgtgacatgccacgtgaagcactacacgaatcccagccaggatgtgactgtgccc
IGHA1_06	aagtccgtgacatgccacgtgaagcactacacgaatcccagccaggatgtgactgtgccc
IGHA1_07	aagtccgtgacatgccacgtgaagcactacacgaatcccagccaggatgtgactgtgccc
IGHA1_08	aagtccgtgacatgccacgtgaagcactacacgaatcccagccaggatgtgactgtgccc
IGHA1_09	aagtccgtgacatgccacgtgaagcactacacgaatcccagccaggatgtgactgtgccc
IGHA1_10	aagtccgtgacatgccacgtgaagcactacacgaatcccagccaggatgtgactgtgccc
IGHA1_11	aagtccgtgacatgccacgtgaagcactacacgaatcccagccaggatgtgactgtgccc
IGHA1_12	aagtccgtgacatgccacgtgaagcactacacgaatcccagccaggatgtgactgtgccc
IGHA1_13	aagtccgtgacatgccacgtgaagcactacacgaatcccagccaggatgtgactgtgccc
IGHA1_14	aagtccgtgacatgccacgtgaagcactacacgaatcccagccaggatgtgactgtgccc
IGHA1_15	aagtccgtgacatgccacgtgaagcactacacgaatcccagccaggatgtgactgtgccc
IGHA1_16	aagtccgtgacatgccacgtgaagcactacacgaatcccagccaggatgtgactgtgccc
IGHA1_17	aagtccgtgacatgccacgtgaagcactacacgaatcccagccaggatgtgactgtgccc
IGHA1_18	aagtccgtgacatgccacgtgaagcactacacgaatcccagccaggatgtgactgtgccc
IGHA1_19	aagtccgtgacatgccacgtgaagcactacacgaatcccagccaggatgtgactgtgccc
IGHA1_20	aagtccgtgacatgccacgtgaagcactacacgaatcccagccaggatgtgactgtgccc
IGHA1_21	aagtccgtgacatgccacgtgaagcactacacgaatcccagccaggatgtgactgtgccc
IGHA1_22	aagtccgtgacatgccacgtgaagcactacacgaatcccagccaggatgtgactgtgccc
IGHA2_IMGT	aagtccgtgacatgccacgtgaagcactacacgaatcccagccaggatgtgactgtgccc
IGHA2_GRCh37	aagtccgtgacatgccacgtgaagcactacacgaattccagccaggatgtgactgtgccc
IGHA2_01	aagtccgtgacatgccacgtgaagcactacacgaatcccagccaggatgtgactgtgccc
IGHA2_02	aagtccgtgacatgccacgtgaagcactacacgaatcccagccaggatgtgactgtgccc
IGHA2_03	aagtccgtgacatgccacgtgaagcactacacgaattccagccaggatgtgactgtgccc
IGHA2_04	aagtccgtgacatgccacgtgaagcactacacgaattccagccaggatgtgactgtgccc
IGHA2_05	aagtccgtgacatgccacgtgaagcactacacgaattccagccaggatgtgactgtgccc
IGHA2_06	aagtccgtgacatgccacgtgaagcactacacgaattccagccaggatgtgactgtgccc
IGHA2_07	aagtccgtgacatgccacgtgaagcactacacgaattccagccaggatgtgactgtgccc
IGHA2_08	aagtccgtgacatgccacgtgaagcactacacgaatcccagccaggatgtgactgtgccc
IGHA2_09	aagtccgtgacatgccacgtgaagcactacacgaattccagccaggatgtgactgtgccc
IGHA2_10	aagtccgtgacatgccacgtgaagcactacacgaatcccagccaggatgtgactgtgccc
IGHA2_11	aagtccgtgacatgccacgtgaagcactacacgaatcccagccaggatgtgactgtgccc
IGHA2_12	aagtccgtgacatgccacgtgaagcactacacgaatcccagccaggatgtgactgtgccc
IGHA2_13	aagtccgtgacatgccacgtgaagcactacacgaattccagccaggatgtgactgtgccc
IGHA2_14	aagtccgtgacatgccacgtgaagcactacacgaattccagccaggatgtgactgtgccc
IGHA2_15	aagtccgtgacatgccacgtgaagcactacacgaatcccagccaggatgtgactgtgccc
IGHA2_16	aagtccgtgacatgccacgtgaagcactacacgaatcccagccaggatgtgactgtgccc
IGHA2_17	aagtccgtgacatgccacgtgaagcactacacgaatcccagccaggatgtgactgtgccc
IGHA2_18	aagtccgtgacatgccacgtgaagcactacacgaatcccagccaggatgtgactgtgccc
IGHA2_19	aagtccgtgacatgccacgtgaagcactacacgaattccagccaggatgtgactgtgccc
IGHA2_20	aagtccgtgacatgccacgtgaagcactacacgaatcccagccaggatgtgactgtgccc
IGHA2_21	aagtccgtgacatgccacgtgaagcactacacgaatcccagccaggatgtgactgtgccc
IGHA2_22	aagtccgtgacatgccacgtgaagcactacacgaattccagccaggatgtgactgtgccc
IGHA2_23	aagtccgtgacatgccacgtgaagcactacacgaattccagccaggatgtgactgtgccc
IGHA2_24	aagtccgtgacatgccacgtgaagcactacacgaatcccagccaggatgtgactgtgccc
IGHA2_25	aagtccgtgacatgccacgtgaagcactacacgaatcccagccaggatgtgactgtgccc
IGHA2_26	aagtccgtgacatgccacgtgaagcactacacgaatcccagccaggatgtgactgtgccc

```
IGHA1_IMGT      tgcccaggtccctcaactccacctaccccatctccctcaactccacctaccccatctccc
IGHA1_GRCh37    tgcccagttccctcaactccacctaccccatctccctcaactccacctaccccatctccc
IGHA1_01        tgcccagttccctcaactccacctaccccatctccctcaactccacctaccccatctccc
IGHA1_02        tgcccagttccctcaactccacctaccccatctccctcaactccacctaccccatctccc
IGHA1_03        tgcccagttccctcaactccacctaccccatctccctcaactccacctaccccatctccc
IGHA1_04        tgcccagttccctcaactccacctaccccatctccctcaactccacctaccccatctccc
IGHA1_05        tgcccagttccctcaactccacctaccccatctccctcaactccacctaccccatctccc
IGHA1_06        tgcccagttccctcaactccacctaccccatctccctcaactccacctaccccatctccc
IGHA1_07        tgcccagttccctcaactccacctaccccatctccctcaactccacctaccccatctccc
IGHA1_08        tgcccagttccctcaactccacctaccccatctccctcaactccacctaccccatctccc
IGHA1_09        tgcccagttccctcaactccacctaccccatctccctcaactccacctaccccatctccc
IGHA1_10        tgcccagttccctcaactccacctaccccatctccctcaactccacctaccccatctccc
IGHA1_11        tgcccagttccctcaactccacctaccccatctccctcaactccacctaccccatctccc
IGHA1_12        tgcccagttccctcaactccacctaccccatctccctcaactccacctaccccatctccc
IGHA1_13        tgcccagttccctcaactccacctaccccatctccctcaactccacctaccccatctccc
IGHA1_14        tgcccagttccctcaactccacctaccccatctccctcaactccacctaccccatctccc
IGHA1_15        tgcccagttccctcaactccacctaccccatctccctcaactccacctaccccatctccc
IGHA1_16        tgcccagttccctcaactccacctaccccatctccctcaactccacctaccccatctccc
IGHA1_17        tgcccagttccctcaactccacctaccccatctccctcaactccacctaccccatctccc
IGHA1_18        tgcccagttccctcaactccacctaccccatctccctcaactccacctaccccatctccc
IGHA1_19        tgcccagttccctcaactccacctaccccatctccctcaactccacctaccccatctccc
IGHA1_20        tgcccagttccctcaactccacctaccccatctccctcaactccacctaccccatctccc
IGHA1_21        tgcccagttccctcaactccacctaccccatctccctcaactccacctaccccatctccc
IGHA1_22        tgcccagttccctcaactccacctaccccatctccctcaactccacctaccccatctccc
IGHA2_IMGT      tgcccaggtc---------------------------------------ccccacctccc
IGHA2_GRCh37    tgccgagttc---------------------------------------ccccacctccc
IGHA2_01        tgcccagttc---------------------------------------ccccacctccc
IGHA2_02        tgcccagttc---------------------------------------ccccacctccc
IGHA2_03        tgccgagttc---------------------------------------ccccacctccc
IGHA2_04        tgccgagttc---------------------------------------ccccacctccc
IGHA2_05        tgccgagttc---------------------------------------ccccacctccc
IGHA2_06        tgccgagttc---------------------------------------ccccacctccc
IGHA2_07        tgccgagttc---------------------------------------ccccacctccc
IGHA2_08        tgcccagttc---------------------------------------ccccacctccc
IGHA2_09        tgccgagttc---------------------------------------ccccacctccc
IGHA2_10        tgcccagttc---------------------------------------ccccacctccc
IGHA2_11        tgcccagttc---------------------------------------ccccacctccc
IGHA2_12        tgcccagttc---------------------------------------ccccacctccc
IGHA2_13        tgccgagttc---------------------------------------ccccacctccc
IGHA2_14        tgccgagttc---------------------------------------ccccacctccc
IGHA2_15        tgcccagttc---------------------------------------ccccacctccc
IGHA2_16        tgcccagttc---------------------------------------ccccacctccc
IGHA2_17        tgcccagttc---------------------------------------ccccacctccc
IGHA2_18        tgcccagttc---------------------------------------ccccacctccc
IGHA2_19        tgccgagttc---------------------------------------ccccacctccc
IGHA2_20        tgcccagttc---------------------------------------ccccacctccc
IGHA2_21        tgcccagttc---------------------------------------ccccacctccc
IGHA2_22        tgccgagttc---------------------------------------ccccacctccc
IGHA2_23        tgccgagttc---------------------------------------ccccacctccc
IGHA2_24        tgcccagttc---------------------------------------ccccacctccc
IGHA2_25        tgcccagttc---------------------------------------ccccacctccc
IGHA2_26        tgcccagttc---------------------------------------ccccacctccc
```

IGHA1_IMGT	tcatgctgccacccccgactgtcactgcaccgaccggccctcgaggacctgctcttaggt
IGHA1_GRCh37	tcatgctgccacccccgactgtcactgcaccgaccggccctcgaggacctgctcttaggt
IGHA1_01	tcatgctgccacccccgactgtcactgcaccgaccggccctcgaggacctgctcttaggt
IGHA1_02	tcatgctgccacccccgactgtcactgcaccgaccggccctcgaggacctgctcttaggt
IGHA1_03	tcatgctgccacccccgactgtcactgcaccgaccggccctcgaggacctgctcttaggt
IGHA1_04	tcatgctgccacccccgactgtcactgcaccgaccggccctcgaggacctgctcttaggt
IGHA1_05	tcatgctgccacccccgactgtcactgcaccgaccggccctcgaggacctgctcttaggt
IGHA1_06	tcatgctgccacccccgactgtcactgcaccgaccggccctcgaggacctgctcttaggt
IGHA1_07	tcatgctgccacccccgactgtcactgcaccgaccggccctcgaggacctgctcttaggt
IGHA1_08	tcatgctgccacccccgactgtcgctgcaccgaccggccctcgaggacctgctcttaggt
IGHA1_09	tcatgctgccacccccgactgtcgctgcaccgaccggccctcgaggacctgctcttaggt
IGHA1_10	tcatgctgccacccccgactgtcactgcaccgaccggccctcgaggacctgctcttaggt
IGHA1_11	tcatgctgccacccccgactgtcgctgcaccgaccggccctcgaggacctgctcttaggt
IGHA1_12	tcatgctgccacccccgactgtcactgcaccgaccggccctcgaggacctgctcttaggt
IGHA1_13	tcatgctgccacccccgactgtcactgcaccgaccggccctcgaggacctgctcttaggt
IGHA1_14	tcatgctgccacccccgactgtcactgcaccgaccggccctcgaggacctgctcttaggt
IGHA1_15	tcatgctgccacccccgactgtcactgcaccgaccggccctcgaggacctgctcttaggt
IGHA1_16	tcatgctgccacccccgactgtcactgcaccgaccggccctcgaggacctgctcttaggt
IGHA1_17	tcatgctgccacccccgactgtcactgcaccgaccggccctcgaggacctgctcttaggt
IGHA1_18	tcatgctgccacccccgactgtcactgcaccgaccggccctcgaggacctgctcttaggt
IGHA1_19	tcatgctgccacccccgactgtcactgcaccgaccggccctcgaggacctgctcttaggt
IGHA1_20	tcatgctgccacccccgactgtcactgcaccgaccggccctcgaggacctgctcttaggt
IGHA1_21	tcatgctgccacccccgactgtcactgcaccgaccggccctcgaggacctgctcttaggt
IGHA1_22	tcatgctgccacccccgactgtcactgcaccgaccggccctcgaggacctgctcttaggt
IGHA2_IMGT	ccatgctgccacccccgactgtcgctgcaccgaccggccctcgaggacctgctcttaggt
IGHA2_GRCh37	ccatgctgccacccccgactgtcgctgcaccgaccggccctcgaggacctgctcttaggt
IGHA2_01	ccatgctgccacccccgactgtcgctgcaccgaccggccctcgaggacctgctcttaggt
IGHA2_02	ccatgctgccacccccgactgtcgctgcaccgaccggccctcgaggacctgctcttaggt
IGHA2_03	ccatgctgccacccccgactgtcgctgcaccgaccggccctcgaggacctgctcttaggt
IGHA2_04	ccatgctgccacccccgactgtcgctgcaccgaccggccctcgaggacctgctcttaggt
IGHA2_05	ccatgctgccacccccgactgtcgctgcaccgaccggccctcgaggacctgctcttaggt
IGHA2_06	ccatgctgccacccccgactgtcgctgcaccgaccggccctcgaggacctgctcttaggt
IGHA2_07	ccatgctgccacccccgactgtcgctgcaccgaccggccctcgaggacctgctcttaggt
IGHA2_08	ccatgctgccacccccgactgtcgctgcaccgaccggccctcgaggacctgctcttaggt
IGHA2_09	ccatgctgccacccccgactgtcgctgcaccgaccggccctcgaggacctgctcttaggt
IGHA2_10	ccatgctgccacccccgactgtcgctgcaccgaccggccctcgaggacctgctcttaggt
IGHA2_11	ccatgctgccacccccgactgtcgctgcaccgaccggccctcgaggacctgctcttaggt
IGHA2_12	ccatgctgccacccccgactgtcgctgcaccgaccggccctcgaggacctgctcttaggt
IGHA2_13	ccatgctgccacccccgactgtcgctgcaccgaccggccctcgaggacctgctcttaggt
IGHA2_14	ccatgctgccacccccgactgtcgctgcaccgaccggccctcgaggacctgctcttaggt
IGHA2_15	ccatgctgccacccccgactgtcgctgcaccgaccggccctcgaggacctgctcttaggt
IGHA2_16	ccatgctgccacccccgactgtcgctgcaccgaccggccctcgaggacctgctcttaggt
IGHA2_17	ccatgctgccacccccgactgtcgctgcaccgaccggccctcgaggacctgctcttaggt
IGHA2_18	ccatgctgccacccccgactgtcgctgcaccgaccggccctcgaggacctgctcttaggt
IGHA2_19	ccatgctgccacccccgactgtcgctgcaccgaccggccctcgaggacctgctcttaggt
IGHA2_20	ccatgctgccacccccgactgtcgctgcaccgaccggccctcgaggacctgctcttaggt
IGHA2_21	ccatgctgccacccccgactgtcgctgcaccgaccggccctcgaggacctgctcttaggt
IGHA2_22	ccatgctgccacccccgactgtcgctgcaccgaccggccctcgaggacctgctcttaggt
IGHA2_23	ccatgctgccacccccgactgtcgctgcaccgaccggccctcgaggacctgctcttaggt
IGHA2_24	ccatgctgccacccccgactgtcgctgcaccgaccggccctcgaggacctgctcttaggt
IGHA2_25	ccatgctgccacccccgactgtcgctgcaccgaccggccctcgaggacctgctcttaggt
IGHA2_26	ccatgctgccacccccgactgtcgctgcaccgaccggccctcgaggacctgctcttaggt

IGHA1_IMGT	tcagaagcgaacctcacgtgcacactgaccggcctgagagatgcctcaggtgtcaccttc
IGHA1_GRCh37	tcagaagcgaacctcacgtgcacactgaccggcctgagagatgcctcaggtgtcaccttc
IGHA1_01	tcagaagcgaacctcacgtgcacactgaccggcctgagagatgcctcaggtgtcaccttc
IGHA1_02	tcagaagcgaacctcacgtgcacactgaccggcctgagagatgcctcaggtgtcaccttc
IGHA1_03	tcagaagcgaacctcacgtgcacactgaccggcctgagagatgcctcaggtgtcaccttc
IGHA1_04	tcagaagcgaacctcacgtgcacactgaccggcctgagagatgcctcaggtgtcaccttc
IGHA1_05	tcagaagcgaacctcacgtgcacactgaccggcctgagagatgcctcaggtgtcaccttc
IGHA1_06	tcagaagcgaacctcacgtgcacactgaccggcctgagagatgcctcaggtgtcaccttc
IGHA1_07	tcagaagcgaacctcacgtgcacactgaccggcctgagagatgcctcaggtgtcaccttc
IGHA1_08	tcagaagcgaacctcacgtgcacactgaccggcctgagagatgcctcaggtgtcaccttc
IGHA1_09	tcagaagcgaacctcacgtgcacactgaccggcctgagagatgcctcaggtgtcaccttc
IGHA1_10	tcagaagcgaacctcacgtgcacactgaccggcctgagagatgcctcaggtgtcaccttc
IGHA1_11	tcagaagcgaacctcacgtgcacactgaccggcctgagagatgcctcaggtgtcaccttc
IGHA1_12	tcagaagcgaacctcacgtgcacactgaccggcctgagagatgcctcaggtgtcaccttc
IGHA1_13	tcagaagcgaacctcacgtgcacactgaccggcctgagagatgcctcaggtgtcaccttc
IGHA1_14	tcagaagcgaacctcacgtgcacactgaccggcctgagagatgcctcaggtgtcaccttc
IGHA1_15	tcagaagcgaacctcacgtgcacactgaccggcctgagagatgcctcaggtgtcaccttc
IGHA1_16	tcagaagcgaacctcacgtgcacactgaccggcctgagagatgcctcaggtgtcaccttc
IGHA1_17	tcagaagcgaacctcacgtgcacactgaccggcctgagagatgcctcaggtgtcaccttc
IGHA1_18	tcagaagcgaacctcacgtgcacactgaccggcctgagagatgcctcaggtgtcaccttc
IGHA1_19	tcagaagcgaacctcacgtgcacactgaccggcctgagagatgcctcaggtgtcaccttc
IGHA1_20	tcagaagcgaacctcacgtgcacactgaccggcctgagagatgcctcaggtgtcaccttc
IGHA1_21	tcagaagcgaacctcacgtgcacactgaccggcctgagagatgcctcaggtgtcaccttc
IGHA1_22	tcagaagcgaacctcacgtgcacactgaccggcctgagagatgcctcaggtgtcaccttc
IGHA2_IMGT	tcagaagcgaacctcacgtgcacactgaccggcctgagagatgcctctggtgccaccttc
IGHA2_GRCh37	tcagaagcgaacctcacgtgcacactgaccggcctgagagatgcctctggtgccaccttc
IGHA2_01	tcagaagcgaacctcacgtgcacactgaccggcctgagagatgcctctggtgccaccttc
IGHA2_02	tcagaagcgaacctcacgtgcacactgaccggcctgagagatgcctctggtgccaccttc
IGHA2_03	tcagaagcgaacctcacgtgcacactgaccggcctgagagatgcctctggtgccaccttc
IGHA2_04	tcagaagcgaacctcacgtgcacactgaccggcctgagagatgcctctggtgccaccttc
IGHA2_05	tcagaagcgaacctcacgtgcacactgaccggcctgagagatgcctctggtgccaccttc
IGHA2_06	tcagaagcgaacctcacgtgcacactgaccggcctgagagatgcctctggtgccaccttc
IGHA2_07	tcagaagcgaacctcacgtgcacactgaccggcctgagagatgcctctggtgccaccttc
IGHA2_08	tcagaagcgaacctcacgtgcacactgactggcctgagagatgcctctggtgccaccttc
IGHA2_09	tcagaagcgaacctcacgtgcacactgaccggcctgagagatgcctctggtgccaccttc
IGHA2_10	tcagaagcgaacctcacgtgcacactgaccggcctgagagatgcctctggtgccaccttc
IGHA2_11	tcagaagcgaacctcacgtgcacactgaccggcctgagagatgcctctggtgccaccttc
IGHA2_12	tcagaagcgaacctcacgtgcacactgaccggcctgagagatgcctctggtgccaccttc
IGHA2_13	tcagaagcgaacctcacgtgcacactgaccggcctgagagatgcctctggtgccaccttc
IGHA2_14	tcagaagcgaacctcacgtgcacactgaccggcctgagagatgcctctggtgccaccttc
IGHA2_15	tcagaagcgaacctcacgtgcacactgaccggcctgagagatgcctctggtgccaccttc
IGHA2_16	tcagaagcgaacctcacgtgcacactgaccggcctgagagatgcctctggtgccaccttc
IGHA2_17	tcagaagcgaacctcacgtgcacactgaccggcctgagagatgcctctggtgccaccttc
IGHA2_18	tcagaagcgaacctcacgtgcacactgactggcctgagagatgcctctggtgccaccttc
IGHA2_19	tcagaagcgaacctcacgtgcacactgaccggcctgagagatgcctctggtgccaccttc
IGHA2_20	tcagaagcgaacctcacgtgcacactgaccggcctgagagatgcctctggtgccaccttc
IGHA2_21	tcagaagcgaacctcacgtgcacactgaccggcctgagagatgcctctggtgccaccttc
IGHA2_22	tcagaagcgaacctcacgtgcacactgaccggcctgagagatgcctctggtgccaccttc
IGHA2_23	tcagaagcgaacctcacgtgcacactgaccggcctgagagatgcctctggtgccaccttc
IGHA2_24	tcagaagcgaacctcacgtgcacactgaccggcctgagagatgcctctggtgccaccttc
IGHA2_25	tcagaagcgaacctcacgtgcacactgaccggcctgagagatgcctctggtgccaccttc
IGHA2_26	tcagaagcgaacctcacgtgcacactgaccggcctgagagatgcctctggtgccaccttc

IGHA1_IMGT	acctggacgccctcaagtgggaagagcgctgttcaaggaccacctgagcgtgacctctgt
IGHA1_GRCh37	acctggacgccctcaagtgggaagagcgctgttcaaggaccacctgagcgtgacctctgt
IGHA1_01	acctggacgccctcaagtgggaagagcgctgttcaaggaccacctgagcgtgacctctgt
IGHA1_02	acctggacgccctcaagtgggaagagcgctgttcaaggaccacctgaccgtgacctctgt
IGHA1_03	acctggacgccctcaagtgggaagagcgctgttcaaggaccacctgagcgtgacctctgt
IGHA1_04	acctggacgccctcaagtgggaagagcgctgttcaaggaccacctgagcgtgacctctgt
IGHA1_05	acctggacgccctcaagtgggaagagcgctgttcaaggaccacctgaccgtgacctctgt
IGHA1_06	acctggacgccctcaagtgggaagagcgctgttcaaggaccacctgagcgtgacctctgt
IGHA1_07	acctggacgccctcaagtgggaagagcgctgttcaaggaccacctgaccgtgacctctgt
IGHA1_08	acctggacgccctcaagtgggaagagcgctgttcaaggaccacctgagcgtgacctctgt
IGHA1_09	acctggacgccctcaagtgggaagagcgctgttcaaggaccacctgagcgtgacctctgt
IGHA1_10	acctggacgccctcaagtgggaagagcgctgttcaaggaccacctgagcgtgacctctgt
IGHA1_11	acctggacgccctcaagtgggaagagcgctgttcaaggaccacctgagcgtgacctctgt
IGHA1_12	acctggacgccctcaagtgggaagagcgctgttcaaggaccacctgagcgtgacctctgt
IGHA1_13	acctggacgccctcaagtgggaagagcgctgttcaaggaccacctgagcgtgacctctgt
IGHA1_14	acctggacgccctcaagtgggaagagcgctgttcaaggaccacctgagcgtgacctctgt
IGHA1_15	acctggacgccctcaagtgggaagagcgctgttcaaggaccacctgagcgtgacctctgt
IGHA1_16	acctggacgccctcaagtgggaagagcgctgttcaaggaccacctgagcgtgacctctgt
IGHA1_17	acctggacgccctcaagtgggaagagcgctgttcaaggaccacctgagcgtgacctctgt
IGHA1_18	acctggacgccctcaagtgggaagagcgctgttcaaggaccacctgagcgtgacctctgt
IGHA1_19	acctggacgccctcaagtgggaagagcgctgttcaaggaccacctgagcgtgacctctgt
IGHA1_20	acctggacgccctcaagtgggaagagcgctgttcaaggaccacctgagcgtgacctctgt
IGHA1_21	acctggacgccctcaagtgggaagagcgctgttcaaggaccacctgagcgtgacctctgt
IGHA1_22	acctggacgccctcaagtgggaagagcgctgttcaaggaccacctgagcgtgacctctgt
IGHA2_IMGT	acctggacgccctcaagtgggaagagcgctgttcaaggaccacctgagcgtgacctctgt
IGHA2_GRCh37	acctggacgccctcaagtgggaagagcgctgttcaaggaccacctgagcgtgacctctgt
IGHA2_01	acctggacgccctcaagtgggaagagcgctgttcaaggaccacctgagcgtgacctctgt
IGHA2_02	acctggacgccctcaagtgggaagagcgctgttcaaggaccacctgagcgtgacctctgt
IGHA2_03	acctggacgccctcaagtgggaagagcgctgttcaaggaccacctgagcgtgacctctgt
IGHA2_04	acctggacgccctcaagtgggaagagcgctgttcaaggaccacctgagcgtgacctctgt
IGHA2_05	acctggacgccctcaagtgggaagagcgctgttcaaggaccacctgagcgtgacctctgt
IGHA2_06	acctggacgccctcaagtgggaagagcgctgttcaaggaccacctgagcgtgacctctgt
IGHA2_07	acctggacgccctcaagtgggaagagcgctgttcaaggaccacctgagcgtgacctctgt
IGHA2_08	acctggacgccctcaagtgggaagagcgctgttcaaggaccacctgagcgtgacctctgt
IGHA2_09	acctggacgccctcaagtgggaagagcgctgttcaaggaccacctgagcgtgacctctgt
IGHA2_10	acctggacgccctcaagtgggaagagcgctgttcaaggaccacctgagcgtgacctctgt
IGHA2_11	acctggacgccctcaagtgggaagagcgctgttcaaggaccacctgagcgtgacctctgt
IGHA2_12	acctggacgccctcaagtgggaagagcgctgttcaaggaccacctgagcgtgacctctgt
IGHA2_13	acctggacgccctcaagtgggaagagcgctgttcaaggaccacctgagcgtgacctctgt
IGHA2_14	acctggacgccctcaagtgggaagagcgctgttcaaggaccacctgagcgtgacctctgt
IGHA2_15	acctggacgccctcaagtgggaagagcgctgttcaaggaccacctgagcgtgacctctgt
IGHA2_16	acctggacgccctcaagtgggaagagcgctgttcaaggaccacctgagcgtgacctctgt
IGHA2_17	acctggacgccctcaagtgggaagagcgctgttcaaggaccacctgagcgtgacctctgt
IGHA2_18	acctggacgccctcaagtgggaagagcgctgttcaaggaccacctgagcgtgacctctgt
IGHA2_19	acctggacgccctcaagtgggaagagcgctgttcaaggaccacctgagcgtgacctctgt
IGHA2_20	acctggacgccctcaagtgggaagagcgctgttcaaggaccacctgagcgtgacctctgt
IGHA2_21	acctggacgccctcaagtgggaagagcgctgttcaaggaccacctgagcgtgacctctgt
IGHA2_22	acctggacgccctcaagtgggaagagcgctgttcaaggaccacctgagcgtgacctctgt
IGHA2_23	acctggacgccctcaagtgggaagagcgctgttcaaggaccacctgagcgtgacctctgt
IGHA2_24	acctggacgccctcaagtgggaagagcgctgttcaaggaccacctgagcgtgacctctgt
IGHA2_25	acctggacgccctcaagtgggaagagcgctgttcaaggaccacctgagcgtgacctctgt
IGHA2_26	acctggacgccctcaagtgggaagagcgctgttcaaggaccacctgagcgtgacctctgt

IGHA1_IMGT	ggctgctacagcgtgtccagtgtcctgccgggctgtgccgagccatggaaccatgggaag
IGHA1_GRCh37	ggctgctacagcgtgtccagtgtcctgccgggctgtgccgagccatggaaccatgggaag
IGHA1_01	ggctgctacagcgtgtccagtgtcctgccgggctgtgccgagccatggaaccatgggaag
IGHA1_02	ggctgctacagcgtgtccagtgtcctgccgggctgtgccgagccatggaaccatgggaag
IGHA1_03	ggctgctacagcgtgtccagtgtcctgccgggctgtgccgagccatggaaccatgggaag
IGHA1_04	ggctgctacagcgtgtccagtgtcctgccgggctgtgccgagccatggaaccatgggaag
IGHA1_05	ggctgctacagcgtgtccagtgtcctgccgggctgtgccgagccatggaaccatgggaag
IGHA1_06	ggctgctacagcgtgtccagtgtcctgccgggctgtgccgagccatggaaccatgggaag
IGHA1_07	ggctgctacagcgtgtccagtgtcctgtcgggctgtgccgagccatggaaccatgggaag
IGHA1_08	ggctgctacagcgtgtccagtgtcctgccgggctgtgccgagccatggaaccatgggaag
IGHA1_09	ggctgctacagcgtgtccagtgtcctgccgggctgtgccgagccatggaaccatgggaag
IGHA1_10	ggctgctacagcgtgtccagtgtcctgccgggctgtgccgagccatggaaccatgggaag
IGHA1_11	ggctgctacagcgtgtccagtgtcctgccgggctgtgccgagccatggaaccatgggaag
IGHA1_12	ggctgctacagcgtgtccagtgtcctgtcgggctgtgccgagccatggaaccatgggaag
IGHA1_13	ggctgctacagcgtgtccagtgtcctgccgggctgtgccgagccatggaaccatgggaag
IGHA1_14	ggctgctacagcgtgtccagtgtcctgccgggctgtgccgagccatggaaccatgggaag
IGHA1_15	ggctgctacagcgtgtccagtgtcctgccgggctgtgccgagccatggaaccatgggaag
IGHA1_16	ggctgctacagcgtgtccagtgtcctgccgggctgtgccgagccatggaaccatgggaag
IGHA1_17	ggctgctacagcgtgtccagtgtcctgccgggctgtgccgagccatggaaccatgggaag
IGHA1_18	ggctgctacagcgtgtccagtgtcctgccgggctgtgccgagccatggaaccatgggaag
IGHA1_19	ggctgctacagcgtgtccagtgtcctgccgggctgtgccgagccatggaaccatgggaag
IGHA1_20	ggctgctacagcgtgtccagtgtcctgccgggctgtgccgagccatggaaccatgggaag
IGHA1_21	ggctgctacagcgtgtccagtgtcctgccgggctgtgccgagccatggaaccatgggaag
IGHA1_22	ggctgctacagcgtgtccagtgtcctgccgggctgtgccgagccatggaaccatgggaag
IGHA2_IMGT	ggctgctacagcgtgtccagtgtcctgcctggctgtgcccagccatggaaccatggggag
IGHA2_GRCh37	ggctgctacagcgtgtccagtgtcctgcctggctgtgcccagccatggaaccatggggag
IGHA2_01	ggctgctacagcgtgtccagtgtcctgcctggctgtgcccagccatggaaccatggggag
IGHA2_02	ggctgctacagcgtgtccagtgtcctgcctggctgtgcccagccatggaaccatggggag
IGHA2_03	ggctgctacagcgtgtccagtgtcctgcctggctgtgcccagtcatggaaccatggggag
IGHA2_04	ggctgctacagcgtgtccagtgtcctgcctggctgtgcccagccatggaaccatggggag
IGHA2_05	ggctgctacagcgtgtccagtgtcctgcctggctgtgcccagccatggaaccatggggag
IGHA2_06	ggctgctacagcgtgtccagtgtcctgcctggctgtgcccagccatggaaccatggggag
IGHA2_07	ggctgctacagcgtgtccagtgtcctgcctggctgtgcccagccatggaaccatggggag
IGHA2_08	ggctgctacagcgtgtccagtgtcctgcctggctgtgcccagccatggaaccatggggag
IGHA2_09	ggctgctacagcgtgtccagtgtcctgcctggctgtgcccagccatggaaccatggggag
IGHA2_10	ggctgctacagcgtgtccagtgtcctgcctggctgtgcccagccatggaaccatggggag
IGHA2_11	ggctgctacagcgtgtccagtgtcctgcctggctgtgcccagccatggaaccatggggag
IGHA2_12	ggctgctacagcgtgtccagtgtcctgcctggctgtgcccagccatggaaccatggggag
IGHA2_13	ggctgctacagcgtgtccagtgtcctgcctggctgtgcccagccatggaaccatggggag
IGHA2_14	ggctgctacagcgtgtccagtgtcctgcctggctgtgcccagccatggaaccatggggag
IGHA2_15	ggctgctacagcgtgtccagtgtcctgcctggctgtgcccagccatggaaccatggggag
IGHA2_16	ggctgctacagcgtgtccagtgtcctgcctggctgtgcccagtcatggaaccatggggag
IGHA2_17	ggctgctacagcgtgtccagtgtcctgcctggctgtgcccagccatggaaccatggggag
IGHA2_18	ggctgctacagcgtgtccagtgtcctgcctggctgtgcccagccatggaaccatggggag
IGHA2_19	ggctgctacagcgtgtccagtgtcctgcctggctgtgcccagccatggaaccatggggag
IGHA2_20	ggctgctacagcgtgtccagtgtcctgcctggctgtgcccagccatggaaccatggggag
IGHA2_21	ggctgctacagcgtgtccagtgtcctgcctggctgtgcccagccatggaaccatggggag
IGHA2_22	ggctgctacagcgtgtccagtgtcctgcctggctgtgcccagccatggaaccatggggag
IGHA2_23	ggctgctacagcgtgtccagtgtcctgcctggctgtgcccagtcatggaaccatggggag
IGHA2_24	ggctgctacagcgtgtccagtgtcctgcctggctgtgcccagccatggaaccatggggag
IGHA2_25	ggctgctacagcgtgtccagtgtcctgcctggctgtgcccagccatggaaccatggggag
IGHA2_26	ggctgctacagcgtgtccagtgtcctgcctggctgtgcccagccatggaaccatggggag

IGHA1_IMGT	accttcacttgcactgctgcctaccccgagtccaagaccccgctaaccgccaccctctca
IGHA1_GRCh37	accttcacttgcactgctgcctaccccgagtccaagaccccgctaaccgccaccctctca
IGHA1_01	accttcacttgcactgctgcctaccccgagtccaagaccccgctaaccgccaccctctca
IGHA1_02	accttcacttgcactgctgcctaccccgagtccaagaccccgctaaccgccaccctctca
IGHA1_03	accttcacttgcactgctgcctaccccgagtccaagaccccgctaaccgccaccctctca
IGHA1_04	accttcacttgcactgctgcctaccccgagtccaagaccccgctaaccgccaccctctca
IGHA1_05	accttcacttgcactgctgcctaccccgagtccaagaccccgctaaccgccaccctctca
IGHA1_06	accttcacttgcactgctgcctaccccgagtccaagaccccgctaaccgccaccctctca
IGHA1_07	accttcacttgcactgctgcctaccccgagtccaagaccccgctaaccgccaccctctca
IGHA1_08	accttcacttgcactgctgcctaccccgagtccaagaccccgctaaccgccaccctctca
IGHA1_09	accttcacttgcactgctgcctaccccgagtccaagaccccgctaaccgccaccctctca
IGHA1_10	accttcacttgcactgctgcctaccccgagtccaagaccccgctaaccgccaccctctca
IGHA1_11	accttcacttgcactgctgcctaccccgagtccaagaccccgctaaccgccaccctctca
IGHA1_12	accttcacttgcactgctgcctaccccgagtccaagaccccgctaaccgccaccctctca
IGHA1_13	gccttcacttgcactgctgcctaccccgagtccaagaccccgctaaccgccaccctctca
IGHA1_14	accttcacttgcactgctgcctaccccgagtccaagaccccgctaaccgccaccctctca
IGHA1_15	accttcacttgcactgctgcctaccccgagtccaagaccccgctaaccgccaccctctca
IGHA1_16	accttcacttgcactgctgcctaccccgagtccaagaccccgctaaccgccaccctctca
IGHA1_17	gccttcacttgcactgctgcctaccccgagtccaagaccccgctaaccgccaccctctca
IGHA1_18	accttcacttgcactgctgcctaccccgagtccaagaccccgctaaccgccaccctctca
IGHA1_19	accttcacttgcactgctgcctaccccgagtccaagaccccgctaaccgccaccctctca
IGHA1_20	accttcacttgcactgctgcctaccccgagtccaagaccccgctaaccgccaccctctca
IGHA1_21	accttcacttgcactgctgcctaccccgagtccaagaccccgctaaccgccaccctctca
IGHA1_22	accttcacttgcactgctgcctaccccgagtccaagaccccgctaaccgccaccctctca
IGHA2_IMGT	accttcacctgcactgctgcccaccccgagttgaagaccccactaaccgccaacatcaca
IGHA2_GRCh37	accttcacctgcactgctgcccaccccgagttgaagaccccactaaccgccaacatcaca
IGHA2_01	accttcacctgcactgctgcccaccccgagttgaagaccccactaaccgccaacatcaca
IGHA2_02	accttcacctgcactgctgcccaccccgagttgaagaccccactaaccgccaacatcaca
IGHA2_03	accttcacctgcactgctgcccaccccgagttgaagaccccactaaccgccaacatcaca
IGHA2_04	accttcacctgcactgctgcccaccccgagttgaagaccccactaaccgccaacatcaca
IGHA2_05	accttcacctgcactgctgcccaccccgagttgaagaccccactaaccgccaacatcaca
IGHA2_06	accttcacctgcactgctgcccaccccgagttgaagaccccactaaccgccaacatcaca
IGHA2_07	accttcacctgcactgctgcccaccccgagttgaagaccccactaaccgccaacatcaca
IGHA2_08	accttcacctgcactgctgcccaccccgagttgaagaccccactaaccgccaacatcaca
IGHA2_09	accttcacctgcactgctgcccaccccgagttgaagaccccactaaccgccaacatcaca
IGHA2_10	accttcacctgcactgctgcccaccccgagttgaagaccccactaaccgccaacatcaca
IGHA2_11	accttcacctgcactgctgcccaccccgagttgaagaccccactaaccgccaacatcaca
IGHA2_12	accttcacctgcactgctgcccaccccgagttgaagaccccactaaccgccaacatcaca
IGHA2_13	accttcacctgcactgctgcccaccccgagttgaagaccccactaaccgccaacatcaca
IGHA2_14	accttcacctgcactgctgcccaccccgagttgaagaccccactaaccgccaacatcaca
IGHA2_15	accttcacctgcactgctgcccaccccgagttgaagaccccactaaccgccaacatcaca
IGHA2_16	accttcacctgcactgctgcccaccccgagttgaagaccccactaaccgccaacatcaca
IGHA2_17	accttcacctgcactgctgcccaccccgagttgaagaccccactaaccgccaacatcaca
IGHA2_18	accttcacctgcactgctgcccaccccgagttgaagaccccactaaccgccaacatcaca
IGHA2_19	accttcacctgcactgctgcccaccccgagttgaagaccccactaaccgccaacatcaca
IGHA2_20	accttcacctgcactgctgcccaccccgagttgaagaccccactaaccgccaacatcaca
IGHA2_21	accttcacctgcactgctgcccaccccgagttgaagaccccactaaccgccaacatcaca
IGHA2_22	accttcacctgcactgctgcccaccccgagttgaagaccccactaaccgccaacatcaca
IGHA2_23	accttcacctgcactgctgcccaccccgagttgaagaccccactaaccgccaacatcaca
IGHA2_24	accttcacctgcactgctgcccaccccgagttgaagaccccactaaccgccaacatcaca
IGHA2_25	accttcacctgcactgctgcccaccccgagttgaagaccccactaaccgccaacatcaca
IGHA2_26	accttcacctgcactgctgcccaccccgagttgaagaccccactaaccgccaacatcaca

IGHA1_IMGT	aaatccggaaacacattccggcccgaggtccacctgctgccgccgccgtcggaggagctg
IGHA1_GRCh37	aaatccggaaacacattccggcccgaggtccacctgctgccgccgccgtcggaggagctg
IGHA1_01	aaatccggaaacacattccggcccgaggtccacctgctgccgccgccgtcggaggagctg
IGHA1_02	aaatccggaaacacattccggcccgaggtccacctgctgccgccgccgtcggaggagctg
IGHA1_03	aaatctggaaacacattccggcccgaggtccacctgctgccgccgccgtcggaggagctg
IGHA1_04	aaatccggaaacacattccggcccgaggtccacctgctgccgccgccgtcggaggagctg
IGHA1_05	aaatccggaaacacattccggcccgaggtccacctgctgccgccgccgtcggaggagctg
IGHA1_06	aaatccggaaacacattccggcccgaggtccacctgctgccgccgccgtcggaggagctg
IGHA1_07	aaatccggaaacacattccggcccgaggtccacctgctgccgccgccgtcggaggagctg
IGHA1_08	aaatccggaaacacattccggcccgaggtccacctgctgccgccgccgtcggaggagctg
IGHA1_09	aaatccggaaacacattccggcccgaggtccacctgctgccgccgccgtcggaggagctg
IGHA1_10	aaatccggaaacacattccggcccgaggtccacctgctgccgccgccgtcggaggagctg
IGHA1_11	aaatccggaaacacattccggcccgaggtccacctgctgccgccgccgtcggaggagctg
IGHA1_12	aaatccggaaacacattccggcccgaggtccacctgctgccgccgccgtcggaggagctg
IGHA1_13	aaatccggaaacacattccggcccgaggtccacctgctgccgccgccgtcggaggagctg
IGHA1_14	aaatccggaaacacattccggcccgaggtccacctgctgccgccgccgtcggaggagctg
IGHA1_15	aaatccggaaacacattccggcccgaggtccacctgctgccgccgccgtcggaggagctg
IGHA1_16	aaatccggaaacacattccggcccgaggtccacctgctgccgccgccgtcggaggagctg
IGHA1_17	aaatctggaaacacattccggcccgaggtccacctgctgccgccgccgtcggaggagctg
IGHA1_18	aaatccggaaacacattccggcccgaggtccacctgctgccgccgccgtcggaggagctg
IGHA1_19	aaatccggaaacacattccggcccgaggtccacctgctgccgccgccgtcggaggagctg
IGHA1_20	aaatccggaaacacattccggcccgaggtccacctgctgccgccgccgtcggaggagctg
IGHA1_21	aaatccggaaacacattccggcccgaggtccacctgctgccgccgccgtcggaggagctg
IGHA1_22	aaatccggaaacacattccggcccgaggtccacctgctgccgccgccgtcggaggagctg
IGHA2_IMGT	aaatccggaaacacattccggcccgaggtccacctgctgccgccgccgtcggaggagctg
IGHA2_GRCh37	aaatccggaaacacattccggcccgaggtccacctgctgccgccgccgtcggaggagctg
IGHA2_01	aaatccggaaacacattccggcccgaggtccacctgctgccgccgccgtcggaggagctg
IGHA2_02	aaatccggaaacacattccggcccgaggtccacctgctgccgccgccgtcggaggagctg
IGHA2_03	aaatccggaaacacattccggcccgaggtccacctgctgccgccgccgtcggaggagctg
IGHA2_04	aaatccggaaacacattccggcccgaggtccacctgctgccgccgccgtcggaggagctg
IGHA2_05	aaatccggaaacacattccggcccgaggtccacctgctgccgccgccgtcggaggagctg
IGHA2_06	aaatccggaaacacattccggcccgaggtccacctgctgccgccgccgtcggaggagctg
IGHA2_07	aaatccggaaacacattccggcccgaggtccacctgctgccgccgccgtcggaggagctg
IGHA2_08	aaatccggaaacacattccggcccgaggtccacctgctgccgccgccgtcggaggagctg
IGHA2_09	aaatccggaaacacattccggcccgaggtccacctgctgccgccgccgtcggaggagctg
IGHA2_10	aaatccggaaacacattccggcccgaggtccacctgctgccgccgccgtcggaggagctg
IGHA2_11	aaatccggaaacacattccggcccgaggtccacctgctgccgccgccgtcggaggagctg
IGHA2_12	aaatccggaaacacattccggcccgaggtccacctgctgccgccgccgtcggaggagctg
IGHA2_13	aaatccggaaacacattccggcccgaggtccacctgctgccgccgccgtcggaggagctg
IGHA2_14	aaatccggaaacacattccggcccgaggtccacctgctgccgccgccgtcggaggagctg
IGHA2_15	aaatccggaaacacattccggcccgaggtccacctgctgccgccgccgtcggaggagctg
IGHA2_16	aaatccggaaacacattccggcccgaggtccacctgctgccgccgccgtcggaggagctg
IGHA2_17	aaatccggaaacacattccggcccgaggtccacctgctgccgccgccgtcggaggagctg
IGHA2_18	aaatccggaaacacattccggcccgaggtccacctgctgccgccgccgtcggaggagctg
IGHA2_19	aaatccggaaacacattccggcccgaggtccacctgctgccgccgccgtcggaggagctg
IGHA2_20	aaatccggaaacacattccggcccgaggtccacctgctgccgccgccgtcggaggagctg
IGHA2_21	aaatccggaaacacattccggcccgaggtccacctgctgccgccgccgtcggaggagctg
IGHA2_22	aaatccggaaacacattccggcccgaggtccacctgctgccgccgccgtcggaggagctg
IGHA2_23	aaatccggaaacacattccggcccgaggtccacctgctgccgccgccgttggaggagctg
IGHA2_24	aaatccggaaacacattccggcccgaggtccacctgctgccgccgccgtcggaggagctg
IGHA2_25	aaatccggaaacacattccggcccgaggtccacctgctgccgccgccgtcggaggagctg
IGHA2_26	aaatccggaaacacattccggcccgaggtccacctgctgccgccgccgtcggaggagctg

IGHA1_IMGT	gccctgaacgagctggtgacgctgacgtgcctggcacgcggcttcagccccaaggacgtg
IGHA1_GRCh37	gccctgaacgagctggtgacgctgacgtgcctggcacgcggcttcagccccaaggatgtg
IGHA1_01	gccctgaacgagctggtgacgctgacgtgcctggcacgcggcttcagccccaaggatgtg
IGHA1_02	gccctgaacgagctggtgacgctgacgtgcctggcacgcggcttcagccccaaggatgtg
IGHA1_03	gccctgaacgagctggtgacgctgacgtgcctggcacgcggcttcagccccaaggatgtg
IGHA1_04	gccctgaacgagctggtgacgctgacgtgcctggcacgcggcttcagccccaaggatgtg
IGHA1_05	gccctgaacgagctggtgacgctgacgtgcctggcacgcggcttcagccccaaggatgtg
IGHA1_06	gccctgaacgagctggtgacgctgacgtgcctggcacgcggcttcagccccaaggatgtg
IGHA1_07	gccctgaacgagctggtgacgctgacgtgcctggcacgcggcttcagccccaaggatgtg
IGHA1_08	gccctgaacgagctggtgacgctgacgtgcctggcacgcggcttcagccccaaggatgtg
IGHA1_09	gccctgaacgagctggtgacgctgacgtgcctggcacgcggcttcagccccaaggatgtg
IGHA1_10	gccctgaacgagctggtgacgctgacgtgcctggcacgcggcttcagccccaaggatgtg
IGHA1_11	gccctgaacgagctggtgacgctgacgtgcctggcacgcggcttcagccccaaggatgtg
IGHA1_12	gccctgaacgagctggtgacgctgacgtgcctggcacgcggcttcagccccaaggatgtg
IGHA1_13	gccctgaacgagctggtgacgctgacgtgcctggcacgcggcttcagccccaaggatgtg
IGHA1_14	gccctgaacgagctggtgacgctgacgtgcctggcacgcggcttcagccccaaggatgtg
IGHA1_15	gccctgaacgagctggtgacgctgacgtgcctggcacgcggcttcagccccaaggatgtg
IGHA1_16	gccctgaacgagctggtgacgctgacgtgcctggcacgcggcttcagccccaaggatgtg
IGHA1_17	gccctgaacgagctggtgacgctgacgtgcctggcacgcggcttcagccccaaggatgtg
IGHA1_18	gccctgaacgagctggtgacgctgacgtgcctggcacgcggcttcagccccaaggatgtg
IGHA1_19	gccctgaacgagctggtgacgctgacgtgcctggcacgcggcttcagccccaaggatgtg
IGHA1_20	gccctgaacgagctggtgacgctgacgtgcctggcacgcggcttcagccccaaggatgtg
IGHA1_21	gccctgaacgagctggtgacgctgacgtgcctggcacgcggcttcagccccaaggatgtg
IGHA1_22	gccctgaacgagctggtgacgctgacgtgcctggcacgcggcttcagccccaaggatgtg
IGHA2_IMGT	gccctgaacgagctggtgacgctgacgtgcctggcacgtggcttcagccccaaggatgtg
IGHA2_GRCh37	gccctgaacgagctggtgacgctgacgtgcctggcacgtggcttcagccccaaggatgtg
IGHA2_01	gccctgaacgagctggtgacgctgacgtgcctggcacgtggcttcagccccaaggatgtg
IGHA2_02	gccctgaacgagctggtgacgctgacgtgcctggcacgtggcttcagccccaaggatgtg
IGHA2_03	gccctgaacgagctggtgacgctgacgtgcctggcacgtggcttcagccccaaggacgtg
IGHA2_04	gccctgaacgagctggtgacgctgacgtgcctggcacgtggcttcagccccaaggatgtg
IGHA2_05	gccctgaacgagctggtgacgctgacgtgcctggcacgtggcttcagccccaaggacgtg
IGHA2_06	gccctgaacgagctggtgacgctgacgtgcctggcacgtggcttcagccccaaggacgtg
IGHA2_07	gccctgaacgagctggtgacgctgacgtgcctggcacgtggcttcagccccaaggacgtg
IGHA2_08	gccctgaacgagctggtgacgctgacgtgcctggcacgtggcttcagccccaaggatgtg
IGHA2_09	gccctgaacgagctggtgacgctgacgtgcctggcacgtggcttcagccccaaggacgtg
IGHA2_10	gccctgaacgagctggtgacgctgacgtgcctggcacgtggcttcagccccaaggatgtg
IGHA2_11	gccctgaacgagctggtgacgctgacgtgcctggcacgtggcttcagccccaaggatgtg
IGHA2_12	gccctgaacgagctggtgacgctgacgtgcctggcacgtggcttcagccccaaggatgtg
IGHA2_13	gccctgaacgagctggtgacgctgacgtgcctggcacgtggcttcagccccaaggatgtg
IGHA2_14	gccctgaacgagctggtgacgctgacgtgcctggcacgtggcttcagccccaaggatgtg
IGHA2_15	gccctgaacgagctggtgacgctgacgtgcctggcacgtggcttcagccccaaggacgtg
IGHA2_16	gccctgaacgagctggtgacgctgacgtgcctggcacgtggcttcagccccaaggatgtg
IGHA2_17	gccctgaacgagctggtgacgctgacgtgcctggcacgtggcttcagccccaaggatgtg
IGHA2_18	gccctgaacgagctggtgacgctgacgtgcctggcacgtggcttcagccccaaggatgtg
IGHA2_19	gccctgaacgagctggtgacgctgacgtgcctggcacgtggcttcagccccaaggatgtg
IGHA2_20	gccctgaacgagctggtgacgctgacgtgcctggcacgtggcttcagccccaaggacgtg
IGHA2_21	gccctgaacgagctggtgacgctgacgtgcctggcacgtggcttcagccccaaggatgtg
IGHA2_22	gccctgaacgagctggtgacgctgacgtgcctggcacgtggcttcagccccaaggacgtg
IGHA2_23	gccctgaacgagctggtgacgctgacgtgcctggcacgtggcttcagccccaaggacgtg
IGHA2_24	gccctgaacgagctggtgacgctgacgtgcctggcacgtggcttcagccccaaggacgtg
IGHA2_25	gccctgaacgagctggtgacgctgacgtgcctggcacgtggcttcagccccaaggacgtg
IGHA2_26	gccctgaacgagctggtgacgctgacgtgcctggcacgtggcttcagccccaaggacgtg

IGHA1_IMGT	ctggttcgctggctgcaggggtcacaggagctgccccgcgagaagtacctgacttgggca
IGHA1_GRCh37	ctggttcgctggctgcaggggtcacaggagctgccccgcgagaagtacctgacttgggca
IGHA1_01	ctggttcgctggctgcaggggtcacaggagctgccccgcgagaagtacctgacttgggca
IGHA1_02	ctggttcgctggctgcaggggtcacaggagctgccccgcgagaagtacctgacttgggca
IGHA1_03	ctggttcgctggctgcaggggtcacaggagctgccccgcgagaagtacctgacttgggca
IGHA1_04	ctggttcgctggctgcaggggtcacaggagctgccccacgagaagtacctgacttgggca
IGHA1_05	ctggttcgctggctgcaggggtcacaggagctgccccgcgagaagtacctgacttgggca
IGHA1_06	ctggttcgctggctgcaggggtcacaggagctgccccgcgagaagtacctgacttgggca
IGHA1_07	ctggttcgctggctgcaggggtcacaggagctgccccgcgagaagtacctgacttgggca
IGHA1_08	ctggttcgctggctgcaggggtcacaggagctgccccgcgagaagtacctgacttgggca
IGHA1_09	ctggttcgctggctgcaggggtcacaggagctgccccgcgagaagtacctgacttgggca
IGHA1_10	ctggttcgctggctgcaggggtcacaggagctgccccgcgagaagtacctgacttgggca
IGHA1_11	ctggttcgctggctgcaggggtcacaggagctgccccgcgagaagtacctgacttgggca
IGHA1_12	ctggttcgctggctgcaggggtcacaggagctgccccgcgagaagtacctgacttgggca
IGHA1_13	ctggttcgctggctgcaggggtcacaggagctgccccgcgagaagtacctgacttgggca
IGHA1_14	ctggttcgctggctgcaggggtcacaggagctgccccgcgagaagtacctgacttgggca
IGHA1_15	ctggttcgctggctgcaggggtcacaggagctgccccgcgagaagtacctgacttgggca
IGHA1_16	ctggttcgctggctgcaggggtcacaggagctgccccgcgagaagtacctgacttgggca
IGHA1_17	ctggttcgctggctgcaggggtcacaggagctgccccgcgagaagtacctgacttgggca
IGHA1_18	ctggttcgctggctgcaggggtcacaggagctgccccacgagaagtacctgacttgggca
IGHA1_19	ctggttcgctggctgcaggggtcacaggagctgccccgcgagaagtacctgacttgggca
IGHA1_20	ctggttcgctggctgcaggggtcacaggagctgccccgcgagaagtacctgacttgggca
IGHA1_21	ctggttcgctggctgcaggggtcacaggagctgccccgcgagaagtacctgacttgggca
IGHA1_22	ctggttcgctggctgcaggggtcacaggagctgccccgcgagaagtacctgacttgggca
IGHA2_IMGT	ctggttcgctggctgcaggggtcacaggagctgccccgcgagaagtacctgacttgggca
IGHA2_GRCh37	ctggttcgctggctgcaggggtcacaggagctgccccgcgagaagtacctgacttgggca
IGHA2_01	ctggttcgctggctgcaggggtcacaggagctgccccgcgagaagtacctgacttgggca
IGHA2_02	ctggttcgctggctgcaggggtcacaggagctgccccgcgagaagtacctgacttgggca
IGHA2_03	ctggttcgctggctgcaggggtcacaggagctgccccgcgagaagtacctgacttgggca
IGHA2_04	ctggttcgctggctgcaggggtcacaggagctgccccgcgagaagtacctgacttgggca
IGHA2_05	ctggttcgctggctgcaggggtcacaggagctgccccgcgagaagtacctgacttgggca
IGHA2_06	ctggttcgctggctgcaggggtcacaggagctgccccgcgagaagtacctgacttgggca
IGHA2_07	ctggttcgctggctgcaggggtcacaggagctgccccgcgagaagtacctgacttgggca
IGHA2_08	ctggttcgctggctgcaggggtcacaggagctgccccgcgagaagtacctgacttgggca
IGHA2_09	ctggttcgctggctgcaggggtcacaggagctgccccgcgagaagtacctgacttgggca
IGHA2_10	ctggttcgctggctgcaggggtcacaggagctgccccgcgagaagtacctgacttgggca
IGHA2_11	ctggttcgctggctgcaggggtcacaggagctgccccgcgagaagtacctgacttgggca
IGHA2_12	ctggttcgctggctgcaggggtcacaggagctgccccgcgagaagtacctgacttgggca
IGHA2_13	ctggttcgctggctgcaggggtcacaggagctgccccgcgagaagtacctgacttgggca
IGHA2_14	ctggttcgctggctgcaggggtcacaggagctgccccgcgagaagtacctgacttgggca
IGHA2_15	ctggttcgctggctgcaggggtcacaggagctgccccgcgagaagtacctgacttgggca
IGHA2_16	ctggttcgctggctgcaggggtcacaggagctgccccgcgagaagtacctgacttgggca
IGHA2_17	ctggttcgctggctgcaggggtcacaggagctgccccgcgagaagtacctgacttgggca
IGHA2_18	ctggttcgctggctgcaggggtcacaggagctgccccgcgagaagtacctgacttgggca
IGHA2_19	ctggttcgctggctgcaggggtcacaggagctgccccgcgagaagtacctgacttgggca
IGHA2_20	ctggttcgctggctgcaggggtcacaggagctgccccgcgagaagtacctgacttgggca
IGHA2_21	ctggttcgctggctgcaggggtcacaggagctgccccgcgagaagtacctgacttgggca
IGHA2_22	ctggttcgctggctgcaggggtcacaggagctgccccgcgagaagtacctgacttgggca
IGHA2_23	ctggttcgctggctgcaggggtcacaggagctgccccgcgagaagtacctgacttgggca
IGHA2_24	ctggttcgctggctgcaggggtcacaggagctgccccgcgagaagtacctgacttgggca
IGHA2_25	ctggttcgctggctgcaggggtcacaggagctgccccgcgagaagtacctgacttgggca
IGHA2_26	ctggttcgctggctgcaggggtcacaggagctgccccgcgagaagtacctgacttgggca

```
IGHA1_IMGT      tcccggcaggagcccagccagggcaccaccaccttcgctgtgaccagcatactgcgcgtg
IGHA1_GRCh37    tcccggcaggagcccagccagggcaccaccaccttcgctgtgaccagcatactgcgcgtg
IGHA1_01        tcccggcaggagcccagccagggcaccaccaccttcgctgtgaccagcatactgcgcgtg
IGHA1_02        tcccggcaggagcccagccagggcaccaccaccttcgctgtgaccagcatactgcgcgtg
IGHA1_03        tcccggcaggagcccagccagggcaccaccaccttcgctgtgaccagcatactgcgcgtg
IGHA1_04        tcccggcaggagcccagccagggcaccaccaccttcgctgtgaccagcatactgcgcgtg
IGHA1_05        tcccggcaggagcccagccagggcaccaccaccttcgctgtgaccagcatactgcgcgtg
IGHA1_06        tcccggcaggagcccagccagggcaccaccaccttcgctgtgaccagcatactgcgcgtg
IGHA1_07        tcccggcaggagcccagccagggcaccaccaccttcgctgtgaccagcatactgcgcgtg
IGHA1_08        tcccggcaggagcccagccagggcaccaccaccttcgctgtgaccagcatactgcgcgtg
IGHA1_09        tcccggcaggagcccagccagggcaccaccaccttcgctgtgaccagcatactgcgcgtg
IGHA1_10        tcccggcaggagcccagccagggcaccaccaccttcgctgtgaccagcatactgcgcgtg
IGHA1_11        tcccggcaggagcccagccagggcaccaccaccttcgctgtgaccagcatactgcgcgtg
IGHA1_12        tcccggcaggagcccagccagggcaccaccaccttcgctgtgaccagcatactgcgcgtg
IGHA1_13        tcccggcaggagcccagccagggcaccaccaccttcgctgtgaccagcatactgcgcgtg
IGHA1_14        tcccggcaggagcccagccagggcaccaccaccttcgctgtgaccagcatactgcgcgtg
IGHA1_15        tcccggcaggagcccagccagggcaccaccaccttcgctgtgaccagcatactgcgcgtg
IGHA1_16        tcccggcaggagcccagccagggcaccaccaccttcgctgtgaccagcatactgcgcgtg
IGHA1_17        tcccggcaggagcccagccagggcaccaccaccttcgctgtgaccagcatactgcgcgtg
IGHA1_18        tcccggcaggagcccagccagggcaccaccaccttcgctgtgaccagcatactgcgcgtg
IGHA1_19        tcccggcaggagcccagccagggcaccaccaccttcgctgtgaccagcatactgcgcgtg
IGHA1_20        tcccggcaggagcccagccagggcaccaccaccttcgctgtgaccagcatactgcgcgtg
IGHA1_21        tcccggcaggagcccagccagggcaccaccaccttcgctgtgaccagcatactgcgcgtg
IGHA1_22        tcccggcaggagcccagccagggcaccaccaccttcgctgtgaccagcatactgcgcgtg
IGHA2_IMGT      tcccggcaggagcccagccagggcaccaccaccttcgctgtgaccagcatactgcgcgtg
IGHA2_GRCh37    tcccggcaggagcccagccagggcaccaccacctacgctgtaaccagcatactgcgcgtg
IGHA2_01        tcccggcaggagcccagccagggcaccaccacctacgctgtaaccagcatactgcgcgtg
IGHA2_02        tcccggcaggagcccagccagggcaccaccacctacgctgtaaccagcatactgcgcgtg
IGHA2_03        tcccagcaggagcccagccagggcaccaccacctacgctgtaaccagcatactgcgcgtg
IGHA2_04        tcccggcaggagcccagccagggcaccaccacctacgctgtaaccagcatactgcgcgtg
IGHA2_05        tcccggcaggagcccagccagggcaccaccacctacgctgtaaccagcatactgcgcgtg
IGHA2_06        tcccggcaggagcccagccagggcaccaccacctacgctgtaaccagcatactgcgcgtg
IGHA2_07        tcccggcaggagcccagccagggcaccaccacctacgctgtaaccagcatactgcgcgtg
IGHA2_08        tcccggcaggagcccagccagggcaccaccacctacgctgtaaccagcatactgcgcgtg
IGHA2_09        tcccggcaggagcccagccagggcaccaccacctacgctgtaaccagcatactgcgcgtg
IGHA2_10        tcccggcaggagcccagccagggcaccaccacctacgctgtaaccagcatactgcgcgtg
IGHA2_11        tcccggcaggagcccagccagggcaccaccacctacgctgtaaccagcatactgcgcgtg
IGHA2_12        tcccagcaggagcccagccagggcaccaccacctacgctgtaaccagcatactgcgcgtg
IGHA2_13        tcccggcaggagcccagccagggcaccaccacctacgctgtaaccagcatactgcgcgtg
IGHA2_14        tcccggcaggagcccagccagggcaccaccacctacgctgtaaccagcatactgcgcgtg
IGHA2_15        tcccagcaggagcccagccagggcaccaccacctacgctgtaaccagcatactgcgcgtg
IGHA2_16        tcccggcaggagcccagccagggcaccaccacctacgctgtaaccagcatactgcgcgtg
IGHA2_17        tcccggcaggagcccagccagggcaccaccacctacgctgtaaccagcatactgcgcgtg
IGHA2_18        tcccggcaggagcccagccagggcaccaccacctacgctgtaaccagcatactgcgcgtg
IGHA2_19        tcccggcaggagcccagccagggcaccaccacctacgctgtaaccagcatactgcgcgtg
IGHA2_20        tcccggcaggagcccagccagggcaccaccacctacgctgtaaccagcatactgcgcgtg
IGHA2_21        tcccggcaggagcccagccagggcaccaccacctacgctgtaaccagcatactgcgcgtg
IGHA2_22        tcccggcaggagcccagccagggcaccaccacctacgctgtaaccagcatactgcgcgtg
IGHA2_23        tcccagcaggagcccagccagggcaccaccacctacgctgtaaccagcatactgcgcgtg
IGHA2_24        tcccggcaggagcccagccagggcaccaccacctacgctgtaaccagcatactgcgcgtg
IGHA2_25        tcccggcaggagcccagccagggcaccaccacctacgctgtaaccagcatactgcgcgtg
IGHA2_26        tcccggcaggagcccagccaggccaccaccacctacgctgtaaccagcatactgcgcgtg
```

IGHA1_IMGT	gcagccgaggactggaagaagggggacaccttctcctgcatggtgggccacgaggccctg
IGHA1_GRCh37	gcagccgaggactggaagaagggggacaccttctcctgcatggtgggccacgaggccctg
IGHA1_01	gcagccgaggactggaagaagggggacaccttctcctgcatggtgggccacgaggccctg
IGHA1_02	gcagccgaggactggaagaagggggacaccttctcctgcatggtgggccacgaggccctg
IGHA1_03	gcagccgaggactggaagaagggggacaccttctcctgcatggtgggccacgaggccctg
IGHA1_04	gcagccgaggactggaagaagggggacaccttctcctgcatggtgggccacgaggccctg
IGHA1_05	gcagccgaggactggaagaagggggacaccttctcctgcatggtgggccacgaggccctg
IGHA1_06	gcagccgaggactggaagaagggggacaccttctcctgcatggtgggccacgaggccctg
IGHA1_07	gcagccgaggactggaagaagggggacaccttctcctgcatggtgggccacgaggccctg
IGHA1_08	gcagccgaggactggaagaagggggacaccttctcctgcatggtgggccacgaggccctg
IGHA1_09	gcagccgaggactggaagaagggggacaccttctcctgcatggtgggccacgaggccctg
IGHA1_10	gcagccgaggactggaagaagggggacaccttctcctgcatggtgggccacgaggccctg
IGHA1_11	gcagccgaggactggaagaagggggacaccttctcctgcatggtgggccacgaggccctg
IGHA1_12	gcagccgaggactggaagaagggggacaccttctcctgcatggtgggccacgaggccctg
IGHA1_13	gcagccgaggactggaagaagggggacaccttctcctgcatggtgggccacgaggccctg
IGHA1_14	gcagccgaggactggaagaagggggacaccttctcctgcatggtgggccacgaggccctg
IGHA1_15	gcagccgaggactggaagaagggggacaccttctcctgcatggtgggccacgaggccctg
IGHA1_16	gcagccgaggactggaagaagggggacaccttctcctgcatggtgggccacgaggccctg
IGHA1_17	gcagccgaggactggaagaagggggacaccttctcctgcatggtgggccacgaggccctg
IGHA1_18	gcagccgaggactggaagaagggggacaccttctcctgcatggtgggccacgaggccctg
IGHA1_19	gcagccgaggactggaagaagggggacaccttctcctgcatggtgggccacgaggccctg
IGHA1_20	gcagccgaggactggaagaagggggacaccttctcctgcatggtgggccacgaggccctg
IGHA1_21	gcagccgaggactggaagaagggggacaccttctcctgcatggtgggccacgaggccctg
IGHA1_22	gcagccgaggactggaagaagggggacaccttctcctgcatggtgggccacgaggccctg
IGHA2_IMGT	gcagccgaggactggaagaagggggacaccttctcctgcatggtgggccacgaggccctg
IGHA2_GRCh37	gcagctgaggactggaagaaggggggaccttctcctgcatggtgggccacgaggccctg
IGHA2_01	gcagccgaggactggaagaagggggacaccttctcctgcatggtgggccacgaggccctg
IGHA2_02	gcagctgaggactggaagaaggggggaccttctcctgcatggtgggccacgaggccctg
IGHA2_03	gcagctgaggactggaagaaggggggaccttctcctgcatggtgggccacgaggccctg
IGHA2_04	gcagctgaggactggaagaaggggggaccttctcctgcatggtgggccacgaggccctg
IGHA2_05	gcagctgaggactggaagaaggggggaccttctcctgcatggtgggccacgaggccctg
IGHA2_06	gcagctgaggactggaagaagggggacaccttctcctgcatggtgggccacgaggccctg
IGHA2_07	gcagctgaggactggaagaaggggggaccttctcctgcatggtgggccacgaggccctg
IGHA2_08	gcagccgaggactggaagaagggggacaccttctcctgcatggtgggccacgaggccctg
IGHA2_09	gcagccgaggactggaagaaggggggaccttctcctgcatggtgggccacgaggccctg
IGHA2_10	gcagctgaggactggaagaagggggacaccttctcctgcatggtgggccacgaggccctg
IGHA2_11	gcagccgaggactggaagaaggggggaccttctcctgcatggtgggccacgaggccctg
IGHA2_12	gcagctgaggactggaagaaggggggaccttctcctgcatggtgggccacgaggccctg
IGHA2_13	gcagccgaggactggaagaaggggggaccttctcctgcatggtgggccacgaggccctg
IGHA2_14	gcagctgaggactggaagaagggggacaccttctcctgcatggtgggccacgaggccctg
IGHA2_15	gcagctgaggactggaagaaggggggaccttctcctgcatggtgggccacgaggccctg
IGHA2_16	gcagccgaggactggaagaagggggacaccttctcctgcatggtgggccacgaggccctg
IGHA2_17	gcagctgaggactggaagaagggggacaccttctcctgcatggtgggccacgaggccctg
IGHA2_18	gcagctgaggactggaagaaggggggaccttctcctgcatggtgggccacgaggccctg
IGHA2_19	gcagctgaggactggaagaaggggggaccttctcctgcatggtgggccacgaggccctg
IGHA2_20	gcagctgaggactggaagaaggggggaccttctcctgcatggtgggccacgaggccctg
IGHA2_21	gcagctgaggactggaagaaggggggaccttctcctgcatggtgggccacgaggccctg
IGHA2_22	gcagctgaggactggaagaaggggggaccttctcctgcatggtgggccacgaggccctg
IGHA2_23	gcagctgaggactggaagaaggggggaccttctcctgcatggtgggccacgaggccctg
IGHA2_24	gcagccgaggactggaagaaggggggaccttctcctgcatggtgggccacgaggccctg
IGHA2_25	gcagccgaggactggaagaagggggacaccttctcctgcatggtgggccacgaggccctg
IGHA2_26	gcagctgaggactggaagaaggggggaccttctcctgcatggtgggccacgaggccctg

IGHA1_IMGT	ccgctggccttcacacagaagaccatcgaccgcttggcgggtaaacccacccatgtcaat
IGHA1_GRCh37	ccgctggccttcacacagaagaccatcgaccgcttggcgggtaaacccacccatgtcaat
IGHA1_01	ccgctggccttcacacagaagaccatcgaccgcttggcgggtaaacccacccatgtcaat
IGHA1_02	ccgctggccttcacacagaagaccatcgaccgcttggcgggtaaacccacccatgtcaat
IGHA1_03	ccgctggccttcacacagaagaccatcgaccgcttggcgggtaaacccacccatgtcaat
IGHA1_04	ccgctggccttcacacagaagaccatcgaccgcttggcgggtaaacccacccacgtcaat
IGHA1_05	ccgctggccttcacacagaagaccatcgaccgcttggcgggtaaacccacccatgtcaat
IGHA1_06	ccgctggccttcacacagaagaccatcgaccgcttggcgggtaaacccacccatgtcaat
IGHA1_07	ccgctggccttcacacagaagaccatcgaccgcttggcgggtaaacccacccatgtcaat
IGHA1_08	ccgctggccttcacacagaagaccatcgaccgcttggcgggtaaacccacccatgtcaat
IGHA1_09	ccgctggccttcacacagaagaccatcgaccgcttggcgggtaaacccacccacgtcaat
IGHA1_10	ccgctggccttcacacagaagaccatcgaccgcatggcgggtaaacccacccatgtcaat
IGHA1_11	ccgctggccttcacacagaagaccatcgaccgcatggcgggtaaacccacccatgtcaat
IGHA1_12	ccgctggccttcacacagaagaccatcgaccgcttggcgggtaaacccacccatgtcaat
IGHA1_13	ccgctggccttcacacagaagaccatcgaccgcttggcgggtaaacccacccatgtcaat
IGHA1_14	ccgctggccttcacacagaagaccatcgaccgcttggcgggtaaacccacccacgtcaat
IGHA1_15	ccgctggccttcacacagaagaccatcgaccgcatggcgggtaaacccacccatgtcaat
IGHA1_16	ccgctggccttcacacagaagaccatcgaccgcatggcgggtaaacccacccacgtcaat
IGHA1_17	ccgctggccttcacacagaagaccatcgaccgcttggcgggtaaacccacccatgtcaat
IGHA1_18	ccgctggccttcacacagaagaccatcgaccgcttggcgggtaaacccacccatgtcaat
IGHA1_19	ccgctggccttcacacagaagaccatcgaccgcttggcgggtaaacccacccatgtcaat
IGHA1_20	ccgctggccttcacacagaagaccatcgaccgcttggcgggtaaacccacccatgtcaat
IGHA1_21	ccgctggccttcacacagaagaccatcgaccgcatggcgggtaaacccacccatgtcaat
IGHA1_22	ccgctggccttcacacagaagaccatcgaccgcttggcgggtaaacccacccatgtcaat
IGHA2_IMGT	ccgctggccttcacacagaagaccatcgaccgcttggcgggtaaacccacccatgtcaat
IGHA2_GRCh37	ccgctggccttcacacagaagaccatcgaccgcatggcgggtaaacccacccacatcaat
IGHA2_01	ccgctggccttcacacagaagaccatcgaccgcatggcgggtaaacccacccatgtcaat
IGHA2_02	ccgctggccttcacacagaagaccatcgaccgcatggcgggtaaacccacccacatcaat
IGHA2_03	ccgctggccttcacacagaagaccatcgaccgcatggcgggtaaacccacccacatcaat
IGHA2_04	ccgctggccttcacacagaagaccatcgaccgcatggcgggtaaacccacccacatcaat
IGHA2_05	ccgctggccttcacacagaagaccatcgaccgcatggcgggtaaacccacccacatcaat
IGHA2_06	ccgctggccttcacacagaagaccatcgaccgcatggcgggtaaacccacccacatcaat
IGHA2_07	ccgctggccttcacacagaagaccatcgaccgcatggcgggtaaacccacccacatcaat
IGHA2_08	ccgctggccttcacacagaagaccatcgaccgcatggcgggtaaacccacccatgtcaat
IGHA2_09	ccgctggccttcacacagaagaccatcgaccgcatggcgggtaaacccacccacatcaat
IGHA2_10	ccgctggccttcacacagaagaccatcgaccgcatggcgggtaaacccacccacatcaat
IGHA2_11	ccgctggccttcacacagaagaccatcgaccgcatggcgggtaaacccacccacatcaat
IGHA2_12	ccgctggccttcacacagaagaccatcgaccgcatggcgggtaaacccacccacatcaat
IGHA2_13	ccgctggccttcacacagaagaccatcgaccgcatggcgggtaaacccacccacatcaat
IGHA2_14	ccgctggccttcacacagaagaccatcgaccgcatggcgggtaaacccacccacatcaat
IGHA2_15	ccgctggccttcacacagaagaccatcgaccgcatggcgggtaaacccacccacatcaat
IGHA2_16	ccgctggccttcacacagaagaccatcgaccgcatggcgggtaaacccacccatgtcaat
IGHA2_17	ccgctggccttcacacagaagaccatcgaccgcatggcgggtaaacccacccacatcaat
IGHA2_18	ccgctggccttcacacagaagaccatcgaccgcatggcgggtaaacccacccacatcaat
IGHA2_19	ccgctggccttcacacagaagaccatcgaccgcatggcgggtaaacccacccacatcaat
IGHA2_20	ccgctggccttcacacagaagaccatcgaccgcatggcgggtaaacccacccacatcaat
IGHA2_21	ccgctggccttcacacagaagaccatcgaccgcatggcgggtaaacccacccacatcaat
IGHA2_22	ccgctggccttcacacagaagaccatcgaccgcatggcgggtaaacccacccacatcaat
IGHA2_23	ccgctggccttcacacagaagaccatcgaccgcatggcgggtaaacccacccacatcaat
IGHA2_24	ccgctggccttcacacagaagaccatcgaccgcatggcgggtaaacccacccacatcaat
IGHA2_25	ccgctggccttcacacagaagaccatcgaccgcatggcgggtaaacccacccatgtcaat
IGHA2_26	ccgctggccttcacacagaagaccatcgaccgcatggcgggtaaacccacccacatcaat

IGHA1_IMGT	gtgtctgttgtcatggcggaggtggacggcacctgctactgagccgcccgcctgtcccca
IGHA1_GRCh37	gtgtctgttgtcatggcggaggtggacggcacctgctactgagccgcccgcctgtcccca
IGHA1_01	gtgtctgttgtcatggcggaggtggacggcacctgctactgagccgcccgcctgtcccca
IGHA1_02	gtgtctgttgtcatggcggaggtggacggcacctgctactgagccgcccgcctgtcccca
IGHA1_03	gtgtctgttgtcatggcggaggtggacggcacctgctactgagccgcccgcctgtcccca
IGHA1_04	gtgtctgttgtcatggcggaggtggacggcacctgctactgagccgcccgcctgtcccca
IGHA1_05	gtgtctgttgtcatggcggaggtggacggcacctgctactgagccacccgcctgtcccca
IGHA1_06	gtgtctgttgtcatggcggaggtggacggcacctgctactgagccacccgcctgtcccca
IGHA1_07	gtgtctgttgtcatggcggaggtggacggcacctgctactgagccgcccgcctgtcccca
IGHA1_08	gtgtctgttgtcatggcggaggtggacggcacctgctactgagccgcccgcctgtcccca
IGHA1_09	gtgtctgttgtcatggcggaggtggacggcacctgctactgagccgcccgcctgtcccca
IGHA1_10	gtgtctgttgtcatggcggaggtggacggcacctgctactgagccgcccgcctgtcccca
IGHA1_11	gtgtctgttgtcatggcggaggtggacggcacctgctactgagccgcccgcctgtcccca
IGHA1_12	gtgtctgttgtcatggcggaggtggacggcacctgctactgagccgcccgcctgtcccca
IGHA1_13	gtgtctgttgtcatggcggaggtggacggcacctgctactgagccgcccgcctgtcccca
IGHA1_14	gtgtctgttgtcatggcggaggtggacggcacctgctactgagccgcccgcctgtcccca
IGHA1_15	gtgtctgttgtcatggcggaggcggatggcacctgctactgagccgcccgcctgtcccca
IGHA1_16	gtgtctgttgtcatggcggaggtggacggcacctgctactgagccgcccgcctgtcccca
IGHA1_17	gtgtctgttgtcatggcggaggtggacggcacctgctactgagccgcccgcctgtcccca
IGHA1_18	gtgtctgttgtcatggcggaggtggacggcacctgctactgagccgcccgcctgtcccca
IGHA1_19	gtgtctgttgtcatggcggaggcggatggcacctgctactgagccgcccgcctgtcccca
IGHA1_20	gtgtctgttgtcatggcggaggtggacggcacctgctactgagccgcccgcctgtcccca
IGHA1_21	gtgtctgttgtcatggcggaggtggatggcacctgctactgagccgcccgcctgtcccca
IGHA1_22	gtgtctgttgtcatggcggaggtggacggcacctgctactgagccgcccgcctgtcccca
IGHA2_IMGT	gtgtctgttgtcatggcggaggtggacggcacctgctactgagccgcccgcctgtcccca
IGHA2_GRCh37	gtgtctgttgtcatggcggaggcggatggcacctgctactgagccgcccgcctgtcccca
IGHA2_01	gtgtctgttgtcatggcggaggtggacggcacctgctactgagccgcccgcctgtcccca
IGHA2_02	gtgtctgttgtcatggcggaggcggatggcacctgctactgagccgcccgcctgtcccca
IGHA2_03	gtgtctgttgtcatggcggaggcggatggcacctgctactgagccgcccgcctgtcccca
IGHA2_04	gtgtctgttgtcatggcggaggcggatggcacctgctactgagccgcccgcctgtcccca
IGHA2_05	gtgtctgttgtcatggcggaggcggatggcacctgctactgagccgcccgcctgtcccca
IGHA2_06	gtgtctgttgtcatggcggaggcggatggcacctgctactgagccgcccgcctgtcccca
IGHA2_07	gtgtctgttgtcatggcggaggcggatggcacctgctactgagccgcccgcctgtcccca
IGHA2_08	gtgtctgttgtcatggcggaggtggacggcacctgctactgagccgcccgcctgtcccca
IGHA2_09	gtgtctgttgtcatggcggaggcggatggcacctgctactgagccgcccgcctgtcccca
IGHA2_10	gtgtctgttgtcatggcggaggcggatggcacctgctactgagccgcccgcctgtcccca
IGHA2_11	gtgtctgttgtcatggcggaggcggatggcacctgctactgagccgcccgcctgtcccca
IGHA2_12	gtgtctgttgtcatggcggaggcggatggcacctgctactgagccgcccgcctgtcccca
IGHA2_13	gtgtctgttgtcatggcggaggcggatggcacctgctactgagccgcccgcctgtcccca
IGHA2_14	gtgtctgttgtcatggcggaggcggatggcacctgctactgagccgcccgcctgtcccca
IGHA2_15	gtgtctgttgtcatggcggaggcggatggcacctgctactgagccgcccgcctgtcccca
IGHA2_16	gtgtctgttgtcatggcggaggtggacggcacctgctactgagccgcccgcctgtcccca
IGHA2_17	gtgtctgttgtcatggcggaggtggacggcacctgctactgagccgcccgcctgtcccca
IGHA2_18	gtgtctgttgtcatggcggaggcggatggcacctgctactgagccgcccgcctgtcccca
IGHA2_19	gtgtctgttgtcatggcggaggcggatggcacctgctactgagccgcccgcctgtcccca
IGHA2_20	gtgtctgttgtcatggcggaggcggatggcacctgctactgagccgcccgcctgtcccca
IGHA2_21	gtgtctgttgtcatggcggaggtggacggcacctgctactgagccgcccgcctgtcccca
IGHA2_22	gtgtctgttgtcatggcggaggcggacggcacctgctactgagccgcccgcctgtcccca
IGHA2_23	gtgtctgttgtcatggcggaggcggatggcacctgctactgagccgcccgcctgtcccca
IGHA2_24	gtgtctgttgtcatggcggaggcggatggcacctgctactgagccgcccgcctgtcccca
IGHA2_25	gtgtctgttgtcatggcggaggtggacggcacctgctactgagccgcccgcctgtcccca
IGHA2_26	gtgtctgttgtcatggcggaggcggatggcacctgctactgagccgcccgcctgtcccca

IGHA1_IMGT	cccctgaataaactccatgctcccccaagc
IGHA1_GRCh37	cccctgaataaactccatgctcccccaagc
IGHA1_01	cccctgaataaactccatgctcccccaagc
IGHA1_02	cccctgaataaactccatgctcccccaagc
IGHA1_03	cccctgaataaactccatgctcccccaagc
IGHA1_04	cccctgaataaactccatgctcccccaagc
IGHA1_05	cccctgaataaactccatgctcccccaagc
IGHA1_06	cccctgaataaactccatgctcccccaagc
IGHA1_07	cccctgaataaactccatgctcccccaagc
IGHA1_08	cccctgaataaactccatgctcccccaagc
IGHA1_09	cccctgaataaactccatgctcccccaagc
IGHA1_10	cccctgaataaactccatgctcccccaagc
IGHA1_11	cccctgaataaactccatgctcccccaagc
IGHA1_12	cccctgaataaactccatgctcccccaagc
IGHA1_13	cccctgaataaactccatgctcccccaagc
IGHA1_14	cccctgaataaactccatgctcccccaagc
IGHA1_15	cccctgaataaactccatgctcccccaagc
IGHA1_16	cccctgaataaactccatgctcccccaagc
IGHA1_17	cccctgaataaactccatgctcccccaagc
IGHA1_18	cccctgaataaactccatgctcccccaagc
IGHA1_19	cccctgaataaactccatgctcccccaagc
IGHA1_20	cccctgaataaactccatgctcccccaagc
IGHA1_21	cccctgaataaactccatgctcccccaagc
IGHA1_22	cccctgaataaactccatgctcccccaagc
IGHA2_IMGT	cccctgaataaactccatgctcccccaagc
IGHA2_GRCh37	cccctgaataaactccatgctcccccaagc
IGHA2_01	cccctgaataaactccatgctcccccaagc
IGHA2_02	cccctgaataaactccatgctcccccaagc
IGHA2_03	cccctgaataaactccatgctcccccaagc
IGHA2_04	cccctgaataaactccatgctcccccaagc
IGHA2_05	cccctgaataaactccatgctcccccaagc
IGHA2_06	cccctgaataaactccatgctcccccaagc
IGHA2_07	cccctgaataaactccatgctcccccaagc
IGHA2_08	cccctgaataaactccatgctcccccaagc
IGHA2_09	cccctgaataaactccatgctcccccaagc
IGHA2_10	cccctgaataaactccatgctcccccaagc
IGHA2_11	cccctgaataaactccatgctcccccaagc
IGHA2_12	cccctgaataaactccatgctcccccaagc
IGHA2_13	cccctgaataaactccatgctcccccaagc
IGHA2_14	cccctgaataaactccatgctcccccaagc
IGHA2_15	cccctgaataaactccatgctcccccaagc
IGHA2_16	cccctgaataaactccatgctcccccaagc
IGHA2_17	cccctgaataaactccatgctcccccaagc
IGHA2_18	cccctgaataaactccatgctcccccaagc
IGHA2_19	cccctgaataaactccatgctcccccaagc
IGHA2_20	cccctgaataaactccatgctcccccaagc
IGHA2_21	cccctgaataaactccatgctcccccaagc
IGHA2_22	cccctgaataaactccatgctcccccaagc
IGHA2_23	cccctgaataaactccatgctcccccaagc
IGHA2_24	cccctgaataaactccatgctcccccaagc
IGHA2_25	cccctgaataaactccatgctcccccaagc
IGHA2_26	cccctgaataaactccatgctcccccaagc

IGHG1, IGHG2, IGHG3 and *IGHG4*

CH1

IGHG1*01_IMGT	gcctccaccaagggcccatcggtcttccccctggcaccctcctccaagagcacctctgggg
IGHG1_GRCh37	gcctccaccaagggcccatcggtcttccccctggcaccctcctccaagagcacctctgggg
IGHG1_01	gcctccaccaagggcccatcggtcttccccctggcaccctcctccaagagcacctctgggg
IGHG1_02	gcctccaccaagggcccatcggtcttccccctggcaccctcctccaagagcacctctgggg
IGHG1_05	gcctccaccaagggcccatcggtcttccccctggcaccctcctccaagagcacctctgggg
IGHG1_07	gcctccaccaagggcccatcggtcttccccctggcaccctcctccaagagcacctctgggg
IGHG1_10	gcctccaccaagggcccatcggtcttccccctggcaccctcctccaagagcacctctgggg
IGHG1_12	gcctccaccaagggcccatcggtcttccccctggcaccctcctccaagagcacctctgggg
IGHG1_14	gcctccaccaagggcccatcggtcttccccctggcaccctcctccaagagcacctctgggg
IGHG1_16	gcctccaccaagggcccatcggtcttccccctggcaccctcctccaagagcacctctgggg
IGHG1_18	gcctccaccaagggcccatcggtcttccccctggcaccctcctccaagagcacctctgggg
IGHG1_19	gcctccaccaagggcccatcggtcttccccctggcaccctcctccaagagcacctctgggg
IGHG2*01_IMGT	gcctccaccaagggcccatcggtcttccccctggcgccctgctccaggagcacctccgagag
IGHG2_GRCh37	gcctccaccaagggcccatcggtcttccccctggcgccctgctccaggagcacctccgagag
IGHG2_01	gcctccaccaagggcccatcggtcttccccctggcgccctgctccaggagcacctccgagag
IGHG2_02	gcctccaccaagggcccatcggtcttccccctggcgccctgctccaggagcacctccgagag
IGHG2_03	gcctccaccaagggcccatcggtcttccccctggcgccctgctccaggagcacctccgagag
IGHG2_04	gcctccaccaagggcccatcggtcttccccctggcgccctgctccaggagcacctccgagag
IGHG2_05	gcctccaccaagggcccatcggtcttccccctggcgccctgctccaggagcacctccgagag
IGHG2_06	gcctccaccaagggcccatcggtcttccccctggcgccctgctccaggagcacctccgagag
IGHG2_07	gcctccaccaagggcccatcggtcttccccctggcgccctgctccaggagcactctccgagag
IGHG2_08	gcctccaccaagggcccatcggtcttccccctggcgccctgctccaggagcacctccgagag
IGHG2_10	gcctccaccaagggcccatcggtcttccccctggcgccctgctccaggagcacctccgagag
IGHG2_13	gcctccaccaagggcccatcggtcttccccctggcgccctgctccaggagcacctccgagag
IGHG2_14	gcctccaccaagggtccatcggtcttccccctggcgccctgctccaggagcacctccgagag
IGHG2_16	gcctccaccaagggcccatcggtcttccccctggcgccctgctccaggagcacctccgagag
IGHG3*01_IMGT	gcttccaccaagggcccatcggtcttccccctggcgccctgctccaggagcacctctgggg
IGHG3_GRCh37	-cttccaccaagggcccatcggtcttccccctggcgccctgctccaggagcacctctgggg
IGHG3_01	-cttccaccaagggcccatcggtcttccccctggcgccctgctccaggagcacctctgggg
IGHG3_03	-cttccaccaagggcccatcggtcttccccctggcgccctgctccaggagcacctctgggg
IGHG3_05	-cttccaccaagggcccatcggtcttccccctggcgccctgctccaggagcacctctgggg
IGHG3_10	-cttccaccaagggcccatcggtcttccccctggcgccctgctccaggagcacctctgggg
IGHG3_11	-cttccaccaagggcccatcggtcttccccctggcgccctgctccaggagcacctctgggg
IGHG3_12	-cttccaccaagggcccatcggtcttccccctggcgccctgctccaggagcacctctgggg
IGHG3_16	-cttccaccaagggcccatcggtcttccccctggcgccctgctccaggagcacctctgggg
IGHG3_17	-cttccaccaagggcccatcggtcttccccctggcgccctgctccaggagcacctctgggg
IGHG3_23	-cttccaccaagggcccatcggtcttccccctggcgccctgctccaggagcacctctgggg
IGHG3_30	-cttccaccaagggcccatcggtcttccccctggcgccctgctccaggagcacctctgggg
IGHG3_31	-cttccaccaagggcccatcggtcttccccctggcgccctgctccaggagcacctctgggg
IGHG4*01_IMGT	gcttccaccaagggcccatccgtcttccccctggcgccctgctccaggagcacctccgagag
IGHG4_GRCh37	gcttccaccaagggcccatcggtcttccccctggcgccctgctccaggagcacctccgagag
IGHG4_01	gcttccaccaagggcccatccgtcttccccctggcgccctgctccaggagcacctccgagag
IGHG4_02	gcttccaccaagggcccatcggtcttccccctggcgccctgctccaggagcacctccgagag
IGHG4_04	gcttccaccaagggcccatcggtcttccccctggcgccctgctccaggagcacctctgagag
IGHG4_09	gcttccaccaagggcccatcggtcttccccctggcgccctgctccaggagcacctccgagag
IGHG4_14	gcttccaccaagggcccatcggtcttccccctggcgccctgctccaggagcacctctgagag
IGHG4_18	gcttccaccaagggcccatcggtcttccccctggcgccctgctccaggagcacctccgagag
IGHG4_19	gcttccaccaagggcccatccgtcttccccctggcgccctgctccaggagcacctccgagag
IGHG4_23	gcttccaccaagggcccatccgtcttccccctggcgccctgctccaggagcacctctgagag
IGHG4_27	gcttccaccaagggcccatcggtcttccccctggcgccctgctccaggagcacctctgagag
IGHG4_28	gcttccaccaagggcccatccgtcttccccctggcgccctgctccaggagcacctctgagag
IGHG4_30	gcttccaccaagggcccatcggtcttccccctggcgccctgctccaggagcacctctgagag
IGHG4_31	gcttccaccaagggcccatcggtcttccccctggcgccctgctccaggagcacctccgagag
IGHG4_32	gcttccaccaagggcccatcggtcttccccctggcgccctgctccaggagcacctctgagag
IGHG4_37	gcttccaccaagggcccatccgtcttccccctggcgccctgctccaggagcacctccgagag
IGHG4_40	gcttccaccaagggcccatcggtcttccccctggcgccctgctccaggagcacctccgagag
IGHG4_41	gcttccaccaagggcccatccgtcttccccctggcgccctgctccaggagcacctccgagag
IGHG4_42	gcttccaccaagggcccatccgtcttccccctggcgccctgctccaggagcacctccgagag

IGHG1*01_IMGT	cacagcggccctgggctgcctggtcaaggactacttccccgaaccggtgacggtgtcgtggaactca
IGHG1_GRCh37	cacagcagccctgggctgcctggtcaaggactacttccccgaaccggtgacggtgtcgtggaactca
IGHG1_01	cacagcggccctgggctgcctggtcaaggactacttccccgaaccggtgacggtgtcgtggaactca
IGHG1_02	cacagcagccctgggctgcctggtcaaggactacttccccgaaccggtgacggtgtcgtggaactca
IGHG1_05	cacagcggccctgggctgcctggtcaaggactacttccccgaaccggtgacggtgtcgtggaactca
IGHG1_07	cacagcggccctgggctgcctggtcaaggactacttccccgaaccggtgacggtgtcgtggaactca
IGHG1_10	cacagcggccctgggctgcctggtcaaggactacttccccgaaccggtgacggtgtcgtggaactca
IGHG1_12	cacagcagccctgggctgcctggtcaaggactacttccccgaaccggtgacggtgtcgtggaactca
IGHG1_14	cacagcggccctgggctgcctggtcaaggactacttccccgaaccggtgacggtgtcgtggaactca
IGHG1_16	cacagcggccctgggctgcctggtcaaggactacttccccgaaccggtgacggtgtcgtggaactca
IGHG1_18	cacagcagccctgggctgcctggtcaaggactacttccccgaaccggtgacggtgtcgtggaactca
IGHG1_19	cacagcggccctgggctgcctggtcaaggactacttccccgaaccggtgacggtgtcatggaactca
IGHG2*01_IMGT	cacagccgccctgggctgcctggtcaaggactacttccccgaaccggtgacggtgtcgtggaactca
IGHG2_GRCh37	cacagcggccctgggctgcctggtcaaggactacttccccgaaccggtgacggtgtcgtggaactca
IGHG2_01	cacagcggccctgggctgcctggtcaaggactacttccccgaaccggtgacggtgtcgtggaactca
IGHG2_02	cacagcggccctgggctgcctggtcaaggactacttccccgaaccggtgacggtgtcgtggaactca
IGHG2_03	cacagcggccctgggctgcctggtcaaggactacttccccgaaccggtgacggtgtcgtggaactca
IGHG2_04	cacagcggccctgggctgcctggtcaaggactacttccccgaaccggtgacggtgtcgtggaactca
IGHG2_05	cacagcggccctgggctgcctggtcaaggactacttccccgaaccggtgacggtgtcgtggaactca
IGHG2_06	cacagcggccctgggctgcctggtcaaggactacttccccgaaccggtgacggtgtcgtggaactca
IGHG2_07	cacagcggccctgggctgcctggtcaaggactacttccccgaaccggtgacggtgtcgtggaactca
IGHG2_08	cacagcggccctgggctgcctggtcaaggactacttccccgaaccggtgacggtgtcgtggaactca
IGHG2_10	cacagcggccctgggctgcctggtcaaggactacttccccgaaccggtgacggtgtcgtggaactca
IGHG2_13	cacagcggccctgggctgcctggtcaaggactacttccccgaaccggtgacggtgtcgtggaactca
IGHG2_14	cacagcggccctgggctgcctggtcaaggactacttccccgaaccggtgacggtgtcgtggaactca
IGHG2_16	cacagcggccctgggctgcctggtcaaggactacttccccgaaccggtgacggtgtcgtggaactca
IGHG3*01_IMGT	cacagcggccctgggctgcctggtcaaggactacttccccgaaccggtgacggtgtcgtggaactca
IGHG3_GRCh37	cacagcggccctgggctgcctggtcaaggactacttcccagaaccggtgacggtgtcgtggaactca
IGHG3_01	cacagcggccctgggctgcctggtcaaggactacttccccgaaccggtgacggtgtcgtggaactca
IGHG3_03	cacagcggccctgggctgcctggtcaaggactacttccccgaaccggtgacggtgtcgtggaactca
IGHG3_05	cacagcggccctgggctgcctggtcaaggactacttccccgaaccggtgacggtgtcgtggaactca
IGHG3_10	cacagcggccctgggctgcctggtcaaggactacttccccgaaccggtgacggtgtcatggaactca
IGHG3_11	cacagcggccctgggctgcctggtcaaggactacttcccagaaccggtgacggtgtcgtggaactca
IGHG3_12	cacagcggccctgggctgcctggtcaaggactacttccccgaaccggtgacggtgtcgtggaactca
IGHG3_16	cacagcggccctgggctgcctggtcaaggactacttccccgaaccggtgacggtgtcatggaactca
IGHG3_17	cacagcggccctgggctgcctggtcaaggactacttccccgaaccggtgacggtgtcatggaactca
IGHG3_23	cacagcggccctgggctgcctggtcaaggactacttccccgaaccggtgacggtgtcgtggaactca
IGHG3_30	cacagcggccctgggctgcctggtcaaggactacttccccgaaccggtgacggtgtcatggaactca
IGHG3_31	cacagcggccctgggctgcctggtcaaggactacttcccagaaccggtgacggtgtcgtggaactca
IGHG4*01_IMGT	cacagccgccctgggctgcctggtcaaggactacttccccgaaccggtgacggtgtcgtggaactca
IGHG4_GRCh37	cacagccgccctgggctgcctggtcaaggactacttccccgaaccggtgacggtgtcgtggaactca
IGHG4_01	cacagccgccctgggctgcctggtcaaggactacttccccgaaccggtgacggtgtcgtggaactca
IGHG4_02	cacagccgccctgggctgcctggtcaaggactacttccccgaaccggtgacggtgtcgtggaactca
IGHG4_04	cacagccgccctgggctgcctggtcaaggactacttccccgaaccggtgacggtgtcgtggaactca
IGHG4_09	cacagccgccctgggctgcctggtcaaggactacttccccgaaccggtgacggtgtcgtggaactca
IGHG4_14	cacagccgccctgggctgcctggtcaaggactacttccccgaaccggtgacggtgtcgtggaactca
IGHG4_18	cacagccgccctgggctgcctggtcaaggactacttccccgaaccggtgacggtgtcgtggaactca
IGHG4_19	cacagccgccctgggctgcctggtcaaggactacttccccgaaccggtgacggtgtcgtggaactca
IGHG4_23	cacagccgccctgggctgcctggtcaaggactacttccccgaaccggtgacggtgtcgtggaactca
IGHG4_27	cacagccgccctgggctgcctggtcaaggactacttccccgaaccggtgacggtgtcgtggaactca
IGHG4_28	cacagccgccctgggctgcctggtcaaggactacttccccgaaccggtgacggtgtcgtggaactca
IGHG4_30	cacagccgccctgggctgcctggtcaaggactacttccccgaaccggtgacggtgtcgtggaactca
IGHG4_31	cacagccgccctgggctgcctggtcaaggactacttccccgaaccggtgacggtgtcgtggaactca
IGHG4_32	cacagccgccctgggctgcctggtcaaggactacttccccgaaccggtgacggtgtcgtggaactca
IGHG4_37	cacagccgccctgggctgcctggtcaaggactacttccccgaaccggtgacggtgtcgtggaactca
IGHG4_40	cacagccgccctgggctgcctggtcaaggactacttccccgaaccggtgacggtgtcgtggaactca
IGHG4_41	cacagccgccctgggctgcctggtcaaggactacttccccgaaccggtgacggtgtcgtggaactca
IGHG4_42	cacagccgccctgggctgcctggtcaaggactacttccccgaaccggtgacggtgtcgtggaactca

IGHG1*01_IMGT	ggcgccctgaccagcggcgtgcacaccttcccggctgtcctacagtcctcaggactctactccctcag
IGHG1_GRCh37	ggcgccctgaccagcggcgtgcacaccttcccggctgtcctacagtcctcaggactctactccctcag
IGHG1_01	ggcgccctgaccagcggcgtgcacaccttcccggctgtcctacagtcctcaggactctactccctcag
IGHG1_02	ggcgccctgaccagcggcgtgcacaccttcccggctgtcctacagtcctcaggactctactccctcag
IGHG1_05	ggcgccctgaccagcggcgtgcacaccttcccggctgtcctacagtcctcaggactctactccctcag
IGHG1_07	ggcgccctgaccagcggcgtgcacaccttcccggctgtcctacagtcctcaggactctactccctcag
IGHG1_10	ggcgccctgaccagcggcgtgcacaccttcccggctgtcctacagtcctcaggactctactccctcag
IGHG1_12	ggcgccctgaccagcggcgtgcacaccttcccggctgtcctacagtcctcaggactctactccctcag
IGHG1_14	ggcgccctgaccagcggcgtgcacaccttcccggctgtcctacagtcctcaggactctactccctcag
IGHG1_16	ggcgccctgaccagcggcgtgcacaccttcccggctgtcctacagtcctcaggactctactccctcag
IGHG1_18	ggcgccctgaccagcggcgtgcacaccttcccggctgtcctacagtcctcaggactctactccctcag
IGHG1_19	ggcgccctgaccagcggcgtgcacaccttcccggctgtcctacagtcctcaggactctactccctcag
IGHG2*01_IMGT	ggcgctctgaccagcggcgtgcacaccttcccagctgtcctacagtcctcaggactctactccctcag
IGHG2_GRCh37	ggcgctctgaccagcggcgtgcacaccttcccggctgtcctacagtcctcaggactctactccctcag
IGHG2_01	ggcgctctgaccagcggcgtgcacaccttcccggctgtcctacagtcctcaggactctactccctcag
IGHG2_02	ggcgctctgaccagcggcgtgcacaccttcccagctgtcctacagtcctcaggactctactccctcag
IGHG2_03	ggcgctctgaccagcggcgtgcacaccttcccagctgtcctacagtcctcaggactctactccctcag
IGHG2_04	ggcgctctgaccagcggcgtgcacaccttcccggctgtcctacagtcctcaggactctactccctcag
IGHG2_05	ggcgctctgaccagcggcgtgcacaccttcccggctgtcctacagtcctcaggactctactccctcag
IGHG2_06	ggcgctctgaccagcggcgtgcacaccttcccagctgtcctacagtcctcaggactctactccctcag
IGHG2_07	ggcgctctgaccagcggcgtgcacaccttcccggctgtcctacagtcctcaggactctactccctcag
IGHG2_08	ggcgctctgaccagcggcgtgcacaccttcccggctgtcctacagtcctcaggactctactccctcag
IGHG2_10	ggcgctctgaccagcggcgtgcacaccttcccggctgtcctacagtcctcaggactctactccctcag
IGHG2_13	ggcgctctgaccagcggcgtgcacaccttcccggctgtcctacagtcctcaggactctattccctcag
IGHG2_14	ggcgctctgaccagcggcgtgcacaccttcccagctgtcctacagtcctcaggactctactccctcag
IGHG2_16	ggcgctctgaccagcggcgtgcacaccttcccggctgtcctacagtcctcaggactctactccctcag
IGHG3*01_IMGT	ggcgccctgaccagcggcgtgcacaccttcccggctgtcctacagtcctcaggactctactccctcag
IGHG3_GRCh37	ggcgccctgaccagcggcgtgcacaccttcccggctgtcctacagtcctcaggactctactccctcag
IGHG3_01	ggcgccctgaccagcggcgtgcacaccttcccggctgtcctacagtcctcaggactctactccctcag
IGHG3_03	ggcgccctgaccagcggcgtgcacaccttcccggctgtcctacagtcctcaggactctactccctcag
IGHG3_05	ggcgccctgaccagcggcgtgcacaccttcccggctgtcctacagtcctcaggactctactccctcag
IGHG3_10	ggcgccctgaccagcggcgtgcacaccttcccggctgtcctacagtcctcaggactctactccctcag
IGHG3_11	ggcgccctgaccagcggcgtgcacaccttcccggctgtcctacagtcctcaggactctactccctcag
IGHG3_12	ggcgccctgaccagcggcgtgcacaccttcccggctgtcctacagtcctcaggactctactccctcag
IGHG3_16	ggcgccctgaccagcggcgtgcacaccttcccggctgtcctacagtcctcaggactctactccctcag
IGHG3_17	ggcgccctgaccagcggcgtgcacaccttcccggctgtcctacagtcctcaggactctactccctcag
IGHG3_23	ggcgccctgaccagcggcgtgcacaccttcccggctgtcctacagtcctcaggactctactccctcag
IGHG3_30	ggcgccctgaccagcggcgtgcacaccttcccggctgtcctacagtcctcaggactctactccctcag
IGHG3_31	ggcgccctgaccagcggcgtgcacaccttcccggctgtcctacagtcctcaggactctactccctcag
IGHG4*01_IMGT	ggcgccctgaccagcggcgtgcacaccttcccggctgtcctacagtcctcaggactctactccctcag
IGHG4_GRCh37	ggcgccctgaccagcggcgtgcacaccttcccggctgtcctacagtcctcaggactctactccctcag
IGHG4_01	ggcgccctgaccagcggcgtgcacaccttcccggctgtcctacagtcctcaggactctactccctcag
IGHG4_02	ggcgccctgaccagcggcgtgcacaccttcccggctgtcctacagtcctcaggactctactccctcag
IGHG4_04	ggcgccctgaccagcggcgtgcacaccttcccggctgtcctacagtcctcaggactctattccctcag
IGHG4_09	ggcgccctgaccagcggcgtgcacaccttcccggctgtcctacagtcctcaggactctactccctcag
IGHG4_14	ggcgccctgaccagcggcgtgcacaccttcccggctgtcctacagtcctcaggactctattccctcag
IGHG4_18	ggcgccctgaccagcggcgtgcaaaccttcccggctgtcctacagtcctcaggactctactccctcag
IGHG4_19	ggcgccctgaccagcggcgtgcacaccttcccggctgtcctacagtcctcaggactctactccctcag
IGHG4_23	ggcgccctgaccagcggcgtgcacaccttcccggctgtcctacagtcctcaggactctattccctcag
IGHG4_27	ggcgccctgaccagcggcgtgcacaccttcccggctgtcctacagtcctcaggactctattccctcag
IGHG4_28	ggcgccctgaccagcggcgtgcacaccttcccggctgtcctacagtcctcaggactctactccctcag
IGHG4_30	ggcgccctgaccagcggcgtgcacaccttcccggctgtcctacagtcctcaggactctattccctcag
IGHG4_31	ggcgccctgaccagcggcgtgcacaccttcccggctgtcctacagtcctcaggactctactccctcag
IGHG4_32	ggcgccctgaccagcggcgtgcacaccttcccggctgtcctacagtcctcaggactctattccctcag
IGHG4_37	ggcgccctgaccagcggcgtgcacaccttcccggctgtcctacagtcctcaggactctactccctcag
IGHG4_40	ggcgccctgaccagcggcgtgcacaccttcccggctgtcctacagtcctcaggactctactccctcag
IGHG4_41	ggcgccctgaccagcggcgtgcacaccttcccggctgtcctacagtcctcaggactctactccctcag
IGHG4_42	ggcgccctgaccagcggcgtgcacaccttcccggctgtcctacagtcctcaggactctactccctcag

```
IGHG1*01_IMGT   cagcgtggtgaccgtgcccctccagcagcttgggcacccagacctacatctgcaacgtgaatc
IGHG1_GRCh37    cagcgtggtgaccgtgcccctccagcagcttgggcacccagacctacatctgcaacgtgaatc
IGHG1_01        cagcgtggtgaccgtgcccctccagcagcttgggcacccagacctacatctgcaacgtgaatc
IGHG1_02        cagcgtggtgaccgtgcccctccagcagcttgggcacccagacctacatctgcaacgtgaatc
IGHG1_05        cagcgtggtgaccgtgcccctccagcagcttgggcacccagacctacatctgcaacgtgaatc
IGHG1_07        cagcgtggtgaccgtgcccctccagcagcttgggcacccagacctacatctgcaacgtgaatc
IGHG1_10        cagcgtggtgaccgtgcccctccagcagcttgggcacccagacctacatctgcaacgtgaatc
IGHG1_12        cagcgtggtgaccgtgcccctccagcagcttgggcacccagacctacatctgcaacgtgaatc
IGHG1_14        cagcgtggtgaccgtgcccctccagcagcttgggcacccagacctacatctgcaacgtgaatc
IGHG1_16        cagcgtggtgaccgtgcccctccagcagcttgggcacccagacctacatctgcaacgtgaatc
IGHG1_18        cagcgtggtgaccgtgcccctccagcagcttgggcacccagacctacatctgcaacgtgaatc
IGHG1_19        cagcgtggtgaccgtgcccctccagcagcttgggcacccagacctacatctgcaacgtgaatc
IGHG2*01_IMGT   cagcgtggtgaccgtgcccctccagcaacttcggcacccagacctacacctgcaacgtagatc
IGHG2_GRCh37    cagcgtggtgaccgtgcccctccagcaacttcggcacccagacctacacctgcaacgtagatc
IGHG2_01        cagcgtggtgaccgtgacctccagcaacttcggcacccagacctacacctgcaacgtagatc
IGHG2_02        cagcgtggtgaccgtgcccctccagcaacttcggcacccagacctacacctgcaacgtagatc
IGHG2_03        cagcgtggtgaccgtgcccctccagcagcttgggcacccagacctacacctgcaacgtagatc
IGHG2_04        cagcgtggtgaccgtgcccctccagcaacttcggcacccagacctacacctgcaacgtagatc
IGHG2_05        cagcgtggtgaccgtgcccctccagcaacttcggcacccagacctacacctgcaacgtagatc
IGHG2_06        cagcgtggtgaccgtgacctccagcaacttcggcacccagacctacacctgcaacgtagatc
IGHG2_07        cagcgtggtgaccgtgcccctccagcaacttcggcacccagacctacacctgcaacgtagatc
IGHG2_08        cagcgtggtgaccgtgcccctccagcaacttcggcacccagacctacacctgcaacgtagatc
IGHG2_10        cagcgtggtgaccgtgcccctccagcaacttcggcacccagacctacacctgcaacgtagatc
IGHG2_13        cagcgtggtgaccgtgcccctccagcaacttcggcacccagacctacacctgcaacgtagatc
IGHG2_14        cagcgtggtgaccgtgcccctccagcaacttcggcacccagacctacacctgcaacgtagatc
IGHG2_16        cagcgtggtgaccgtgcccctccagcaacttcggcacccagacctacacctgcaacgtagatc
IGHG3*01_IMGT   cagcgtggtgaccgtgcccctccagcagcttgggcacccagacctacacctgcaacgtgaatc
IGHG3_GRCh37    cagcgtggtgaccgtgcccctccagcagcttgggcacccagacctacacctgcaacgtgaatc
IGHG3_01        cagcgtggtgaccgtgcccctccagcagcttgggcacccagacctacacctgcaacgtgaatc
IGHG3_03        cagcgtggtgaccgtgcccctccagcagcttgggcacccagacctacacctgcaacgtgaatc
IGHG3_05        cagcgtggtgaccgtgcccctccagcagcttgggcacccagacctacacctgcaacgtgaatc
IGHG3_10        cagcgtggtgaccgtgcccctccagcagcttgggcacccagacctacacctgcaacgtgaatc
IGHG3_11        cagcgtggtgaccgtgcccctccagcagcttgggcacccagacctacacctgcaacgtgaatc
IGHG3_12        cagcgtggtgaccgtgcccctccagcagcttgggcacccagacctacacctgcaacgtgaatc
IGHG3_16        cagcgtggtgaccgtgcccctccagcagcttgggcacccagacctacacctgcaacgtgaatc
IGHG3_17        cagcgtggtgaccgtgcccctccagcagcttgggcacccagacctacatctgcaacgtgaatc
IGHG3_23        cagcgtggtgaccgtgcccctccagcagcttgggcacccagacctacacctgcaacgtgaatc
IGHG3_30        cagcgtggtgaccgtgcccctccagcagcttgggcacccagacctacatctgcaacgtgaatc
IGHG3_31        cagcgtggtgaccgtgcccctccagcagcttgggcacccagacctacacctgcaacgtgaatc
IGHG4*01_IMGT   cagcgtggtgaccgtgcccctccagcagcttgggcacgaagacctacacctgcaacgtagatc
IGHG4_GRCh37    cagcgtggtgaccgtgcccctccagcagcttgggcacgaagacctacacctgcaacgtagatc
IGHG4_01        cagcgtggtgaccgtgcccctccagcagcttgggcacgaagacctacacctgcaacgtagatc
IGHG4_02        cagcgtggtgaccgtgcccctccagcagcttgggcacgaagacctacacctgcaacgtagatc
IGHG4_04        cagcgtggtgaccgtgcccctccagcagcttgggcacgaagacctacacctgcaacgtagatc
IGHG4_09        cagcgtggtgaccgtgcccctccagcagcttgggcacgaagacctacacctgcaacgtagatc
IGHG4_14        cagcgtggtgaccgtgcccctccagcagcttgggcacgaagacctacacctgcaacgtagatc
IGHG4_18        cagcgtggtgaccgtgcccctccagcagcttgggcacgaagacctacacctgcaacgtagatc
IGHG4_19        cagcgtggtgaccgtgcccctccagcagcttgggcacgaagacctacacctgcaacgtagatc
IGHG4_23        cagcgtggtgaccgtgcccctccagcagcttgggcacgaagacctacacctgcaacgtagatc
IGHG4_27        cagcgtggtgaccgtgcccctccagcagcttgggcacgaagacctacacctgcaacgtagatc
IGHG4_28        cagcgtggtgaccgtgcccctccagcagcttgggcacgaagacctacacctgcaacgtagatc
IGHG4_30        cagcgtggtgaccgtgcccctccagcagcttgggcacgaagacctacacctgcaacgtagatc
IGHG4_31        cagcgtggtgaccgtgcccctccagcagcttgggcacgaagacctacacctgcaacgtagatc
IGHG4_32        cagcgtggtgaccgtgcccctccagcagcttgggcacgaagacctacacctgcaacgtagatc
IGHG4_37        cagcgtggtgaccgtgcccctccagcagcttgggcacgaagacctacacctgcaacgtagatc
IGHG4_40        cagcgtggtgaccgtgcccctccagcagcttgggcacgaagacctacacctgcaacgtagatc
IGHG4_41        cagcgtggtgaccgtgcccctccagcagcttgggcacgaagacctacacctgcaacgtagatc
IGHG4_42        cagcgtggtgaccgtgcccctccagcagcttgggcacgaagacctacacctgcaacgtagatc
```

IGHG1*01_IMGT acaagcccagcaacaccaaggtggacaagaaagtt
IGHG1_GRCh37 acaagcccagcaacaccaaggtggacaagaaagtt
IGHG1_01 acaagcccagcaacaccaaggtggacaagaaagtt
IGHG1_02 acaagcccagcaacaccaaggtggacaagaaagtt
IGHG1_05 acaagcccagcaacaccaaggtggacaagaaagtt
IGHG1_07 acaagcccagcaacaccaaggtggacaagaaagtt
IGHG1_10 acaagcccagcaacaccaaggtggacaagaaagtt
IGHG1_12 acaagcccagcaacaccaaggtggacaagaaagtt
IGHG1_14 acaagcccagcaacaccaaggtggacaagaaagtt
IGHG1_16 acaagcccagcaacaccaaggtggacaagaaagtt
IGHG1_18 acaagcccagcaacaccaaggtggacaagaaagtt
IGHG1_19 acaagcccagcaacaccaaggtggacaagaaagtt
IGHG2*01_IMGT acaagcccagcaacaccaaggtggacaagacagtt
IGHG2_GRCh37 acaagcccagcaacaccaaggtggacaagacagtt
IGHG2_01 acaagcccagcaacaccaaggtggacaagacagtt
IGHG2_02 acaagcccagcaacaccaaggtggacaagacagtt
IGHG2_03 acaagcccagcaacaccaaggtggacaagacagtt
IGHG2_04 acaagcccagcaacaccaaggtggacaagacagtt
IGHG2_05 acaagcccagcaacaccaaggtggacaagacagtt
IGHG2_06 acaagcccagcaacaccaaggtggacaagacagtt
IGHG2_07 acaagcccagcaacaccaaggtggacaagacagtt
IGHG2_08 acaagcccagcaacaccaaggtggacaagacagtt
IGHG2_10 acaagcccagcaacaccaaggtggacaagacagtt
IGHG2_13 acaagcccagcaacaccaaggtggacaagacagtt
IGHG2_14 acaagcccagcaacaccaaggtggacaagacagtt
IGHG2_16 acaagcccagcaacaccaaggtggacaagacagtt
IGHG3*01_IMGT acaagcccagcaacaccaaggtggacaagagagtt
IGHG3_GRCh37 acaagcccagcaacaccaaggtggacaagagagtt
IGHG3_01 acaagcccagcaacaccaaggtggacaagagagtt
IGHG3_03 acaagcccagcaacaccaaggtggacaagagagtt
IGHG3_05 acaagcccagcaacaccaaggtggacaagagagtt
IGHG3_10 acaagcccagcaacaccaaggtggacaagagagtt
IGHG3_11 acaagcccagcaacaccaaggtggacaagagagtt
IGHG3_12 acaagcccagcaacaccaaggtggacaagagagtt
IGHG3_16 acaagcccagcaacaccaaggtggacaagagagtt
IGHG3_17 acaagcccagcaacaccaaggtggacaagagagtt
IGHG3_23 acaagcccagcaacaccaaggtggacaagagagtt
IGHG3_30 acaagcccagcaacaccaaggtggacaagagagtt
IGHG3_31 acaagcccagcaacaccaaggtggacaagagagtt
IGHG4*01_IMGT acaagcccagcaacaccaaggtggacaagagagtt
IGHG4_GRCh37 acaagcccagcaacaccaaggtggacaagagagtt
IGHG4_01 acaagcccagcaacaccaaggtggacaagagagtt
IGHG4_02 acaagcccagcaacaccaaggtggacaagagagtt
IGHG4_04 acaagcccagcaacaccaaggtggacaagagagtt
IGHG4_09 acaagcccagcaacaccaaggtggacaagagagtt
IGHG4_14 acaagcccagcaacaccaaggtggacaagagagtt
IGHG4_18 acaagcccagcaacaccaaggtggacaagagagtt
IGHG4_19 acaagcccagcaacaccaaggtggacaagagagtt
IGHG4_23 acaagcccagcaacaccaaggtggacaagagagtt
IGHG4_27 acaagcccagcaacaccaaggtggacaagagagtt
IGHG4_28 acaagcccagcaacaccaaggtggacaagagagtt
IGHG4_30 acaagcccagcaacaccaaggtggacaagagagtt
IGHG4_31 acaagcccagcaacaccaaggtggacaagagagtt
IGHG4_32 acaagcccagcaacaccaaggtggacaagagagtt
IGHG4_37 acaagcccagcaacaccaaggtggacaagagagtt
IGHG4_40 acaagcccagcaacaccaaggtggacaagagagtt
IGHG4_41 acaagcccagcaacaccaaggtggacaagagagtt
IGHG4_42 acaagcccagcaacaccaaggtggacaagagagtt

H gDNA

IGHG1*01_IMGT	gagcccaaatcttgtgacaaaactcacacatgcccaccgtgccca
IGHG1_GRCh37	gagcccaaatcttgtgacaaaactcacacatgcccaccgtgccca
IGHG1_01	gagcccaaatcttgtgacaaaactcacacatgcccaccgtgccca
IGHG1_02	gagcccaaatcttgtgacaaaactcacacatgcccaccgtgccca
IGHG1_05	gagcccaaatcttgtgacaaaactcacacatgcccaccgtgccca
IGHG1_07	gagcccaaatcttgtgacaaaactcacacatgcccaccgtgccca
IGHG1_10	gagcccaaatcttgtgacaaaactcacacatgcccaccgtgccca
IGHG1_12	gagcccaaatcttgtgacaaaactcacacatgcccaccgtgccca
IGHG1_14	gagcccaaatcttgtgacaaaactcacacatgcccaccgtgccca
IGHG1_16	gagcccaaatcttgtgacaaaactcacacatgcccaccgtgccca
IGHG1_18	gagcccaaatcttgtgacaaaactcacacatgcccaccgtgccca
IGHG1_19	gagcccaaatcttgtgacaaaactcacacatgcccaccgtgccca
IGHG2*01_IMGT	gagcgcaaatgttg---------tgtcgagtgcccaccgtgccca
IGHG2_GRCh37	gagcgcaaatgttg---------tgtcgagtgcccaccgtgccca
IGHG2_01	gagcgcaaatgttg---------tgtcgagtgcccaccgtgccca
IGHG2_02	gagcgcaaatgttg---------tgtcgagtgcccaccgtgccca
IGHG2_03	gagcgcaaatgttg---------tgtcgagtgcccaccgtgccca
IGHG2_04	gagcgcaaatgttg---------tgtcgagtgcccaccgtgccca
IGHG2_05	gagcgcaaatgttg---------tgtcgagtgcccaccgtgccca
IGHG2_06	gagcgcaaatgttg---------tgtcgagtgcccaccgtgccca
IGHG2_07	gagcgcaaatgttg---------tgtcgagtgcccaccgtgccca
IGHG2_08	gagcgcaaatgttg---------tgtcgagtgcccaccgtgccca
IGHG2_10	gagcgcaaatgttg---------tgtcgagtgcccaccgtgccca
IGHG2_13	gagcgcaaatgttg---------tgtcgagtgcccaccgtgccca
IGHG2_14	gagcgcaaatgttg---------tgtcgagtgcccaccgtgccca
IGHG2_16	gagcgcaaatgttg---------tgtcgagtgcccaccgtgccca
IGHG3*01_IMGT	gagcccaaatcttgtgacacacctcccccgtgcccaaggtgccca
IGHG3_GRCh37	gagcccaaatcttgtgacacacctcccccgtgcccaaggtgccca
IGHG3_01	gagcccaaatcttgtgacacacctcccccgtgcccaaggtgccca
IGHG3_03	gagcccaaatcttgtgacacacctcccccgtgcccaaggtgccca
IGHG3_05	gagcccaaatcttgtgacacacctcccccgtgcccaaggtgccca
IGHG3_10	gagcccaaatcttgtgacacacctcccccgtgcccaaggtgccca
IGHG3_11	gagcccaaatcttgtgacacacctcccccgtgcccaaggtgccca
IGHG3_12	gagcccaaatcttgtgacacacctcccccgtgcccaaggtgccca
IGHG3_16	gagcccaaatcttgtgacacacctcccccgtgcccaaggtgccca
IGHG3_17	gagcccaaatcttgtgacacacctcccccgtgcccaaggtgccca
IGHG3_23	gagcccaaatcttgtgacacacctcccccgtgcccaaggtgccca
IGHG3_30	gagcccaaatcttgtgacacacctcccccgtgcccaaggtgccca
IGHG3_31	gagcccaaatcttgtgacacacctcccccgtgcccaaggtgccca
IGHG4*01_IMGT	gagtccaaatatgg---------tcccccatgcccatcatgccca
IGHG4_GRCh37	gagtccaaatatgg---------tcccccatgcccatcatgccca
IGHG4_01	gagtccaaatatgg---------tcccccatgcccatcatgccca
IGHG4_02	gagtccaaatatgg---------tcccccatgcccatcatgccca
IGHG4_04	gagtccaaatatgg---------tcccccatgcccatcatgccca
IGHG4_09	gagtccaaatatgg---------tcccccatgcccatcatgccca
IGHG4_14	gagtccaaatatgg---------tcccccatgcccatcatgccca
IGHG4_18	gagtccaaatatgg---------tcccccatgcccatcatgccca
IGHG4_19	gagtccaaatatgg---------tcccccatgcccatcatgccca
IGHG4_23	gagtccaaatatgg---------tcccccatgcccatcatgccca
IGHG4_27	gagtccaaatatgg---------tcccccatgcccatcatgccca
IGHG4_28	gagtccaaatatgg---------tcccccatgcccatcatgccca
IGHG4_30	gagtccaaatatgg---------tcccccatgcccatcatgccca
IGHG4_31	gagtccaaatatgg---------tcccccatgcccatcatgccca
IGHG4_32	gagtccaaatatgg---------tcccccatgcccatcatgccca
IGHG4_37	gagtccaaatatgg---------tcccccatgcccatcatgccca
IGHG4_40	gagtccaaatatgg---------tcccccatgcccatcatgccca
IGHG4_41	gagtccaaatatgg---------tcccccatgcccatcatgccca
IGHG4_42	gagtccaaatatgg---------tcccccatgcccatcatgccca

CH2

IGHG1*01_IMGT	gcacctgaactcctggggggaccgtcagtcttcctcttccccccaaaacccaaggacaccctcatgatc
IGHG1_GRCh37	gcacctgaactcctggggggaccgtcagtcttcctcttccccccaaaacccaaggacaccctcatgatc
IGHG1_01	gcacctgaactcctggggggaccgtcagtcttcctcttccccccaaaacccaaggacaccctcatgatc
IGHG1_02	gcacctgaactcctggggggaccgtcagtcttcctcttccccccaaaacccaaggacaccctcatgatc
IGHG1_05	gcacctgaactcctggggggaccgtcagtcttcctcttccccccaaaacccaaggacaccctcatgatc
IGHG1_07	gcacctgaactcctggggggaccgtcagtcttcctcttccccccaaaacccaaggacaccctcatgatc
IGHG1_10	gcacctgaactcctggggggaccgtcagtcttcctcttccccccaaaacccaaggacaccctcatgatc
IGHG1_12	gcacctgaactcctggggggaccgtcagtcttcctcttccccccaaaacccaaggacaccctcatgatc
IGHG1_14	gcacctgaactcctggggggaccgtcagtcttcctcttccccccaaaacccaaggacaccctcatgatc
IGHG1_16	gcacctgaactcctggggggaccgtcagtcttcctcttccccccaaaacccaaggacaccctcatgatc
IGHG1_18	gcacctgaactcctggggggaccgtcagtcttcctcttccccccaaaacccaaggacaccctcatgatc
IGHG1_19	gcacctgaactcctggggggaccgtcagtcttcctcttccccccaaaacccaaggacaccctcatgatc
IGHG2*01_IMGT	gcacc---acctgtggcaggaccgtcagtcttcctcttccccccaaaacccaaggacaccctcatgatc
IGHG2_GRCh37	gcacc---acctgtggcaggaccgtcagtcttcctcttccccccaaaacccaaggacaccctcatgatc
IGHG2_01	gcacc---acctgtggcaggaccgtcagtcttcctcttccccccaaaacccaaggacaccctcatgatc
IGHG2_02	gcacc---acctgtggcaggaccgtcagtcttcctcttccccccaaaacccaaggacaccctcatgatc
IGHG2_03	gcacc---acctgtggcaggaccgtcagtcttcctcttccccccaaaacccaaggacaccctcatgatc
IGHG2_04	gcacc---acctgtggcaggaccgtcagtcttcctcttccccccaaaacccaaggacaccctcatgatc
IGHG2_05	gcacc---acctgtggcaggaccgtcagtcttcctcttccccccaaaacccaaggacaccctcatgatc
IGHG2_06	gcacc---acctgtggcaggaccgtcagtcttcctcttccccccaaaacccaaggacaccctcatgatc
IGHG2_07	gcacc---acctgtggcaggaccgtcagtcttcctcttccccccaaaacccaaggacaccctcatgatc
IGHG2_08	gcacc---acctgtggcaggaccgtcagtcttcctcttccccccaaaacccaaggacaccctcatgatc
IGHG2_10	gcacc---acctgtggcaggaccgtcagtcttcctcttccccccaaaacccaaggacaccctcatgatc
IGHG2_13	gcacc---acctgtggcaggaccgtcagtcttcctcttccccccaaaacccaaggacaccctcatgatc
IGHG2_14	gcacc---acctgtggcaggaccgtcagtcttcctcttccccccaaaacccaaggacaccctcatgatc
IGHG2_16	gcacc---acctgtggcaggaccgtcagtcttcctcttccccccaaaacccaaggacaccctcatgatc
IGHG3*01_IMGT	gcacctgaactcctgggaggaccgtcagtcttcctcttccccccaaaacccaaggatacccttatgatt
IGHG3_GRCh37	gcacctgaactcctgggaggaccgtcagtcttcctcttccccccaaaacccaaggatacccttatgatt
IGHG3_01	gcacctgaactcctgggaggaccgtcagtcttcctcttccccccaaaacccaaggatacccttatgatt
IGHG3_03	gcacctgaactcctgggaggaccgtcagtcttcctcttccccccaaaacccaaggatacccttatgatt
IGHG3_05	gcacctgaactcctgggaggaccgtcagtcttcctcttccccccaaaacccaaggatacccttatgatt
IGHG3_10	gcacctgaactcctgggaggaccgtcagtcttcctcttccccccaaaacccaaggatacccttatgatt
IGHG3_11	gcacctgaactcctgggaggaccgtcagtcttcctcttccccccaaaacccaaggatacccttatgatt
IGHG3_12	gcacctgaactcctgggaggaccgtcagtcttcctcttccccccaaaacccaaggatacccttatgatt
IGHG3_16	gcacctgaactcctgggaggaccgtcagtcttcctcttccccccaaaacccaaggatacccttatgatt
IGHG3_17	gcacctgaactcctgggaggaccgtcagtcttcctcttccccccaaaacccaaggatacccttatgatt
IGHG3_23	gcacctgaactcctgggaggaccgtcagtcttcctcttccccccaaaacccaaggatacccttatgatt
IGHG3_30	gcacctgaactcctgggaggaccgtcagtcttcctcttccccccaaaacccaaggatacccttatgatt
IGHG3_31	gcacctgaactcctgggaggaccgtcagtcttcctcttccccccaaaacccaaggatacccttatgatt
IGHG4*01_IMGT	gcacctgagttcctggggggaccatcagtcttcctgttccccccaaaacccaaggacactctcatgatc
IGHG4_GRCh37	gcacctgagttcctggggggaccatcagtcttcctgttccccccaaaacccaaggacactctcatgatc
IGHG4_01	gcacctgagttcctggggggaccatcagtcttcctgttccccccaaaacccaaggacactctcatgatc
IGHG4_02	gcacctgagttcctggggggaccatcagtcttcctgttccccccaaaacccaaggacactctcatgatc
IGHG4_04	gcacctgaattcctggggggaccatcagtcttcctgttccccccaaaacccaaggacactctcatgatc
IGHG4_09	gcacctgagttcctggggggaccatcagtcttcctgttccccccaaaacccaaggacactctcatgatc
IGHG4_14	gcacctgagttcctggggggaccatcagtcttcctgttccccccaaaacccaaggacactctcatgatc
IGHG4_18	gcacctgagttcctggggggaccatcagtcttcctgttccccccaaaacccaaggacactctcatgatc
IGHG4_19	gcacctgagttcctggggggaccatcagtcttcctgttccccccaaaacccaaggacactctcatgatc
IGHG4_23	gcacctgagttcctggggggaccatcagtcttcctgttccccccaaaacccaaggacactctcatgatc
IGHG4_27	gcacctgaattcctggggggaccatcagtcttcctgttccccccaaaacccaaggacactctcatgatc
IGHG4_28	gcacctgagttcctggggggaccatcagtcttcctgttccccccaaaacccaaggacactctcatgatc
IGHG4_30	gcacctgaattcctggggggaccatcagtcttcctgttccccccaaaacccaaggacactctcatgatc
IGHG4_31	gcacctgaattcctggggggaccatcagtcttcctgttccccccaaaacccaaggacactctcatgatc
IGHG4_32	gcacctgaattcctggggggaccgtcagtcttcctgttccccccaaaacccaaggacactctcatgatc
IGHG4_37	gcacctgagttcctggggggaccatcagtcttcctgttccccccaaaacccaaggacactctcatgatc
IGHG4_40	gcacctgagttcctggggggaccatcagtcttcctgttccccccaaaacccaaggacactctcatgatc
IGHG4_41	gcacctgagttcctggggggaccatcagtcttcctgttccccccaaaacccaaggacactctcatgatc
IGHG4_42	gcacctgagttcctggggggaccatcagtcttcctgttccccccaaaacccaaggacactctcatgatc

IGHG1*01_IMGT	tcccggacccctgaggtcacatgcgtggtggtggacgtgagccacgaagaccctgaggtcaagttca
IGHG1_GRCh37	tcccggacccctgaggtcacatgcgtggtggtggacgtgagccacgaagaccctgaggtcaagttca
IGHG1_01	tcccggacccctgaggtcacatgcgtggtggtggacgtgagccacgaagaccctgaggtcaagttca
IGHG1_02	tcccggacccctgaggtcacatgcgtggtggtggacgtgagccacgaagaccctgaggtcaagttca
IGHG1_05	tcccggacccctgaggtcacatgcgtggtggtggacgtgagccacgaagaccctgaggtcaagttca
IGHG1_07	tcccggacccctgaggtcacatgcgtggtggtggacgtgagccacgaagaccctgaggtcaagttca
IGHG1_10	tcccggacccctgaggtcacatgcgtggtggtggacgtgagccacgaagaccctgaggtcaagttca
IGHG1_12	tcccggacccctgaggtcacatgcgtggtggtggacgtgagccacgaagaccctgaggtcaagttca
IGHG1_14	tcccggacccctgaggtcacatgcgtggtggtggacgtgagccacgaagaccctgaggtcaagttca
IGHG1_16	tcccggacccctgaggtcacatgcgtggtggtggacgtgagccacgaagaccctgaggtcaagttca
IGHG1_18	tcccggacccctgaggtcacatgcgtggtggtggacgtgagccacgaagaccctgaggtcaagttca
IGHG1_19	tcccggacccctgaggtcacatgcgtggtggtggacgtgagccacgaagaccctgaggtcaagttca
IGHG2*01_IMGT	tcccggacccctgaggtcacgtgcgtggtggtggacgtgagccacgaagacccLgaggtccagttca
IGHG2_GRCh37	tcccggacccctgaggtcacgtgcgtggtggtggacgtgagccacgaagacccggaggtccagttca
IGHG2_01	tcccggacccctgaggtcacgtgcgtggtggtggacgtgagccacgaagacccggaggtccagttca
IGHG2_02	tcccggacccctgaggtcacgtgcgtggtggtggacgtgagccacgaagacccggaggtccagttca
IGHG2_03	tcccggacccctgaggtcacgtgcgtggtggtggacgtgagccacgaagacccggaggtccagttca
IGHG2_04	tcccggacccctgaggtcacgtgcgtggtggtggacgtgagccacgaagacccggaggtccagttca
IGHG2_05	tcccggacccctgaggtcacgtgcgtggtggtggacgtgagccacgaagacccggaggtccagttca
IGHG2_06	tcccggacccctgaggtcacgtgcgtggtggtggacgtgagccacgaagacccggaggtccagttca
IGHG2_07	tcccggacccctgaggtcacgtgcgtggtggtggacgtgagccacgaagacccggaggtccagttca
IGHG2_08	tcccggacccctgaggtcacgtgcgtggtggtggacgtgagccacgaagacccggaggtccagttca
IGHG2_10	tcccggacccctgaggtcacgtgcgtggtggtggacgtgagccacgaagacccggaggtccagttca
IGHG2_13	tcccggacccctgaggtcacgtgcgtggtggtggacgtgagccacgaagacccggaggtccagttca
IGHG2_14	tcccggacccctgaggtcacgtgcgtggtggtggacgtgagccacgaagacccggaggtccagttca
IGHG2_16	tcccggacccctgaggtcacgtgcgtggtggtggacgtgagccacgaagacccggaggtccagttca
IGHG3*01_IMGT	tcccggacccctgaggtcacgtgcgtggtggtggacgtgagccacgaagacccggaggtccagttca
IGHG3_GRCh37	tcccggacccctgaggtcacgtgcgtggtggtggacgtgagccacgaagacccggaggtccagttca
IGHG3_01	tcccggacccctgaggtcacgtgcgtggtggtggacgtgagccacgaagacccggaggtccagttca
IGHG3_03	tcccggacccctgaggtcacgtgcgtggtggtggacgtgagccacgaagacccggaggtccagttca
IGHG3_05	tcccggacccctgaggtcacgtgcgtggtggtggacgtgagccacgaagacccggaggtccagttca
IGHG3_10	tcccggacccctgaggtcacgtgcgtggtggtggacgtgagccacgaagacccggaggtccagttca
IGHG3_11	tcccggacccctgaggtcacgtgcgtggtggtggacgtgagccacgaagacccggaggtccagttca
IGHG3_12	tcccggacccctgaggtcacgtgcgtggtggtggacgtgagccacgaagacccggaggtccagttca
IGHG3_16	tcccggacccctgaggtcacgtgcgtggtggtggacgtgagccacgaagacccggaggtccagttca
IGHG3_17	tcccggacccctgaggtcacgtgcgtggtggtggacgtgagccacgaagacccggaggtccagttca
IGHG3_23	tcccggacccctgaggtcacgtgcgtggtggtggacgtgagccacgaagacccggaggtccagttca
IGHG3_30	tcccggacccctgaggtcacgtgcgtggtggtggacgtgagccacgaagacccggaggtccagttca
IGHG3_31	tcccggacccctgaggtcacgtgcgtggtggtggacgtgagccacgaagacccggaggtccagttca
IGHG4*01_IMGT	tcccggacccctgaggtcacgtgcgtggtggtggacgtgagccaggaagacccggaggtccagttca
IGHG4_GRCh37	tcccggacccctgaggtcacgtgcgtggtggtggacgtgagccaggaagacccggaggtccagttca
IGHG4_01	tcccggacccctgaggtcacgtgcgtggtggtggacgtgagccaggaagacccggaggtccagttca
IGHG4_02	tcccggacccctgaggtcacgtgcgtggtggtggacgtgagccaggaagacccggaggtccagttca
IGHG4_04	tcccggacccctgaggtcacgtgcgtggtggtggacgtgagccaggaagacccggaggtccagttca
IGHG4_09	tcccggacccctgaggtcacgtgcgtggtggtggacgtgagccaggaagacccggaggtccagttca
IGHG4_14	tcccggacccctgaggtcacgtgcgtggtggtggacgtgagccaggaagacccggaggtccagttca
IGHG4_18	tcccggacccctgaggtcacgtgcgtggtggtggacgtgagccaggaagacccggaggtccagttca
IGHG4_19	tcccggacccctgaggtcacgtgcgtggtggtggacgtgagccaggaagacccggaggtccagttca
IGHG4_23	tcccggacccctgaggtcacgtgcgtggtggtggacgtgagccaggaagacccggaggtccagttca
IGHG4_27	tcccggacccctgaggtcacgtgcgtggtggtggacgtgagccaggaagacccggaggtccagttca
IGHG4_28	tcccggacccctgaggtcacgtgcgtggtggtggacgtgagccaggaagacccggaggtccagttca
IGHG4_30	tcccggacccctgaggtcacgtgcgtggtggtggacgtgagccaggaagacccggaggtccagttca
IGHG4_31	tcccggacccctgaggtcacgtgcgtggtggtggacgtgagccaggaagacccggaggtccagttca
IGHG4_32	tcccggacccctgaggtcacgtgcgtggtggtggacgtgagccaggaagacccggaggtccagttca
IGHG4_37	tcccggacccctgaggtcacgtgcgtggtggtggacgtgagccaggaagacccggaggtccagttca
IGHG4_40	tcccggacccctgaggtcacgtgcgtggtggtggacgtgagccaggaagacccggaggtccagttca
IGHG4_41	tcccggacccctgaggtcacgtgcgtggtggtggacgtgagccaggaagacccggaggtccagttca
IGHG4_42	tcccggacccctgaggtcacgtgcgtggtggtggacgtgagccaggaagacccggaggtccagttca

IGHG1*01_IMGT	actggtacgtggacggcgtggaggtgcataatgccaagacaaagccgcgggaggagcagtacaacagcac
IGHG1_GRCh37	actggtacgtggacggcgtggaggtgcataatgccaagacaaagccgcgggaggagcagtacaacagcac
IGHG1_01	actggtacgtggacggcgtggaggtgcataatgccaagacaaagccgcgggaggagcagtacaacagcac
IGHG1_02	actggtacgtggacggcgtggaggtgcataatgccaagacaaagccgcgggaggagcagtacaacagcac
IGHG1_05	actggtacgtggacggcgtggaggtgcataatgccaagacaaagccgcgggaggagcagtacaacagcac
IGHG1_07	actggtacgtggacggcgtggaggtgcataatgccaagacaaagccgcgggaggagcagtacaacagcac
IGHG1_10	actggtacgtggacggcgtggaggtgcataatgccaagacaaagccgcgggaggagcagtacaacagcac
IGHG1_12	actggtacgtggacggcgtggaggtgcataatgccaagacaaagccgcgggaggagcagtacaacagcac
IGHG1_14	actggtacgtggacggcgtggaggtgcataatgccaagacaaagccgcgggaggagcagtacaacagcac
IGHG1_16	actggtacgtggacggcgtggaggtgcataatgccaagacaaagccgcgggaggagcagtacaacagcac
IGHG1_18	actggtacgtggaaggcgtggaggtgcataatgccaagacaaagccgcgggaggagcagtacaacagcac
IGHG1_19	actggtacgtggacggcgtggaggtgcataatgccaagacaaagccgcgggaggagcagtacaacagcac
IGHG2*01_IMGT	actggtacgtggacggcgtggaggtgcataatgccaagacaaagccacgggaggagcagttcaacagcac
IGHG2_GRCh37	actggtacgtggacggcgtggaggtgcataatgccaagacaaagccacgggaggagcagttcaacagcac
IGHG2_01	actggtacgtggacggcatggaggtgcataatgccaagacaaagccacgggaggagcagttcaacagcac
IGHG2_02	actggtacgtggacggcgtggaggtgcataatgccaagacaaagccacgggaggagcagttcaacagcac
IGHG2_03	actggtacgtggacggcgtggaggtgcataatgccaagacaaagccacgggaggagcagttcaacagcac
IGHG2_04	actggtacgtggacggcgtggaggtgcataatgccaagacaaagccacgggaggagcagttcaacagcac
IGHG2_05	actggtacgtggacggcatggaggtgcataatgccaagacaaagccacgggaggagcagttcaacagcac
IGHG2_06	actggtacgtggacggcgtggaggtgcataatgccaagacaaagccacgggaggagcagttcaacagcac
IGHG2_07	actggtacgtggacggcgtggaggtgcataatgccaagacaaagccacgggaggagcagttcaacagcac
IGHG2_08	actggtacgtggacggcgtggaggtgcataatgccaagacaaagccacgggaggagcagttcaacagcac
IGHG2_10	actggtacgtggacggcgtggaggtgcataatgccaagacaaagccacgggaggagcagttcaacagcac
IGHG2_13	actggtacgtggacggcgtggaggtgcataatgccaagacaaagccacgggaggagcagttcaacagcac
IGHG2_14	actggtacgtggacggcgtggaggtgcataatgccaagacaaagccacgggaggagcagttcaacagcac
IGHG2_16	actggtacgtggacggcgtggaggtgcataatgccaagacaaagccacgggaggagcagttcaacagcac
IGHG3*01_IMGT	agtggtacgtggacggcgtggaggtgcataatgccaagacaaagccgcgggaggagcagtacaacagcac
IGHG3_GRCh37	agtggtacgtggacggcgtggaggtgcataatgccaagacaaagccgcgggaggagcagtacaacagcac
IGHG3_01	agtggtacgtggacggcgtggaggtgcataatgccaagacaaagccgcgggaggagcagtacaacagcac
IGHG3_03	agtggtacgtggacggcgtggaggtgcataatgccaagacaaagccgcgggaggagcagtacaacagcac
IGHG3_05	agtggtacgtggacggcgtggaggtgcataatgccaagacaaagctgcgggaggagcagtacaacagcac
IGHG3_10	agtggtacgtggacggcgtggaggtgcataatgccaagacaaagccgcgggaggagcagtacaacagcac
IGHG3_11	agtggtacgtggacggcgtggaggtgcataatgccaagacaaagccgcgggaggagcagtacaacagcac
IGHG3_12	agtggtacgtggacggcgtggaggtgcataatgccaagacaaagctgcgggaggagcagtacaacagcac
IGHG3_16	agtggtacgtggacggcgtggaggtgcataatgccaagacaaagccgcgggaggagcagtacaacagcac
IGHG3_17	agtggtacgtggacggcgtggaggtgcataatgccaagacaaagccgcgggaggagcagtacaacagcac
IGHG3_23	agtggtacgtggacggcgtggaggtgcataatgccaagacaaagctgcgggaggagcagtacaacagcac
IGHG3_30	agtggtacgtggacggcgtggaggtgcataatgccaagacaaagccgcgggaggagcagtacaacagcac
IGHG3_31	agtggtacgtggacggcgtggaggtgcataatgccaagacaaagccgcgggaggagcagtacaacagcac
IGHG4*01_IMGT	actggtacgtggatggcgtggaggtgcataatgccaagacaaagccgcgggaggagcagttcaacagcac
IGHG4_GRCh37	actggtacgtggatggcgtggaggtgcataatgccaagacaaagccgcgggaggagcagttcaacagcac
IGHG4_01	actggtacgtggatggcgtggaggtgcataatgccaagacaaagccgcgggaggagcagttcaacagcac
IGHG4_02	actggtacgtggatggcgtggaggtgcataatgccaagacaaagccgcgggaggagcagttcaacagcac
IGHG4_04	actggtacgtggatggcgtggaggtgcataatgccaagacaaagccgcgggaggagcagttcaacagcac
IGHG4_09	actggtacgtggatggcgtggaggtgcataatgccaagacaaagccgcgggaggagcagttcaacagcac
IGHG4_14	actggtacgtggatggcgtggaggtgcataatgccaagacaaagccgcgggaggagcagttcaacagcac
IGHG4_18	actggtacgtggatggcgtggaggtgcataatgccaagacaaagccgcgggaggagcagttcaacagcac
IGHG4_19	actggtacgtggatggcgtggaggtgcataatgccaagacaaagccgcgggaggagcagttcaacagcac
IGHG4_23	actggtacgtggatggcgtggaggtgcataatgccaagacaaagccgcgggaggagcagttcaacagcac
IGHG4_27	actggtacgtggatggcgtggaggtgcataatgccaagacaaagccgcgggaggagcagttcaacagcac
IGHG4_28	actggtacgtggatggcgtggaggtgcataatgccaagacaaagccgcgggaggagcagttcaacagcac
IGHG4_30	actggtacgtggatggcgtggaggtgcataatgccaagacaaagccgcgggaggagcagttcaacagcac
IGHG4_31	actggtacgtggatggcgtggaggtgcataatgccaagacaaagccgcgggaggagcagttcaacagcac
IGHG4_32	actggtacgtggatggcgtggaggtgcataatgccaagacaaagccgcgggaggagcagttcaacagcac
IGHG4_37	actggtacgtggatggcgtggaggtgcataatgccaagacaaagccgcgggaggagcagttcaacagcac
IGHG4_40	actggtacgtggatggcgtggaggtgcataatgccaagacaaagccgcgggaggagcagttcaacagcac
IGHG4_41	actggtacgtggatggcgtggaggtgcataatgccaagacaaagccgcgggaggagcagttcaacagcac
IGHG4_42	actggtacgtggatggcgtggaggtgcataatgccaagacaaagccgcgggaggagcagttcaacagcac

IGHG1*01_IMGT	gtaccgggtggtcagcgtcctcaccgtcctgcaccaggactggctgaatggcaaggagtacaagt
IGHG1_GRCh37	gtaccgtgtggtcagcgtcctcaccgtcctgcaccaggactggctgaatggcaaggagtacaagt
IGHG1_01	gtaccgtgtggtcagcgtcctcaccgtcctgcaccaggactggctgaatggcaaggagtacaagt
IGHG1_02	gtaccgtgtggtcagcgtcctcaccgtcctgcaccaggactggctgaatggcaaggagtacaagt
IGHG1_05	gtaccgtgtggtcagcgtcctcaccgtcctgcaccaggactggctgaatggcaaggagtacaagt
IGHG1_07	gtaccgtgtggtcagcgtcctcaccgtcctgcaccaggactggctgaatggcaaggagtacaagt
IGHG1_10	gtaccgtgtggtcagcgtcctcaccgtcctgcaccaggactggctgaatggcaaggagtacaagt
IGHG1_12	gtaccgtgtggtcagcgtcctcaccgtcctgcaccaggactggctgaatggcaaggagtacaagt
IGHG1_14	gtaccgtgtggtcagcgtcctcaccgtcctgcaccaggactggctgaatggcaaggagtacaagt
IGHG1_16	gtaccgtgtggtcagcgtcctcaccgtcctgcaccaggactggctgaatggcaaggagtacaagt
IGHG1_18	gtaccgtgtggtcagcgtcctcaccgtcctgcaccaggactggctgaatggcaaggagtacaagt
IGHG1_19	gtaccgtgtggtcagcgtcctcaccgtcctgcaccaggactggctgaatggcaaggagtacaagt
IGHG2*01_IMGT	gttccgtgtggtcagcgtcctcaccgttgtgcaccaggactggctgaacggcaaggagtacaagt
IGHG2_GRCh37	gttccgtgtggtcagcgtcctcaccgtcgtgcaccaggactggctgaacggcaaggagtacaagt
IGHG2_01	gttccgtgtggtcagcgtcctcaccgtcgtgcaccaggactggctgaacggcaaggagtacaagt
IGHG2_02	gttccgtgtggtcagcgtcctcaccgttgtgcaccaggactggctgaacggcaaggagtacaagt
IGHG2_03	gttccgtgtggtcagcgtcctcaccgttgtgcaccaggactggctgaacggcaaggagtacaagt
IGHG2_04	gttccgtgtggtcagcgtcctcaccgtcgtgcaccaggactggctgaacggcaaggagtacaagt
IGHG2_05	gttccgtgtggtcagcgtcctcaccgtcgtgcaccaggactggctgaacggcaaggagtacaagt
IGHG2_06	gttccgtgtggtcagcgtcctcaccgttgtgcaccaggactggctgaacggcaaggagtacaagt
IGHG2_07	gttccgtgtggtcagcgtcctcaccgtcgtgcaccaggactggctgaacggcaaggagtacaagt
IGHG2_08	gttccgtgtggtcagcgtcctcaccgtcgtgcaccaggactggctgaacggcaaggagtacaagt
IGHG2_10	gttccgtgtggtcagcgtcctcaccgtcgtgcaccaggactggctgaacggcaaggagtacaagt
IGHG2_13	gttccgtgtggtcagcgtcctcaccgtcgtgcaccaggactggctgaacggcaaggagtacaagt
IGHG2_14	gttccgtgtggtcagcgtcctcaccgttgtgcaccaggactggctgaacggcaaggagtacaagt
IGHG2_16	gttccgtgtggtcagcgtcctcaccgtcctgcaccaggactggctgaacggcaaggagtacaagt
IGHG3*01_IMGT	gttccgtgtggtcagcgtcctcaccgtcctgcaccaggactggctgaacggcaaggagtacaagt
IGHG3_GRCh37	gttccgtgtggtcagcgtcctcaccgtcctgcaccaggactggctgaacggcaaggagtacaagt
IGHG3_01	gttccgtgtggtcagcgtcctcaccgtcctgcaccaggactggctgaacggcaaggagtacaagt
IGHG3_03	gttccgtgtggtcagcgtcctcaccgtcctgcaccaggactggctgaacggcaaggagtacaagt
IGHG3_05	gttccgtgtggtcagcgtcctcaccgtcctgcaccaggactggctgaacggcaaggagtacaagt
IGHG3_10	gttccgtgtggtcagcgtcctcaccgtcctgcaccaggactggctgaacggcaaggagtacaagt
IGHG3_11	gttccgtgtggtcagcgtcctcaccgtcctgcaccaggactggctgaacggcaaggagtacaagt
IGHG3_12	gttccgtgtggtcagcgtcctcaccgtcctgcaccaggactggctgaacggcaaggagtacaagt
IGHG3_16	gttccgtgtggtcagcgtcctcaccgtcctgcaccaggactggctgaacggcaaggagtacaagt
IGHG3_17	gttccgtgtggtcagcgtcctcaccgtcctgcaccaggactggctgaacggcaaggagtacaagt
IGHG3_23	gttccgtgtggtcagcgtcctcaccgtcctgcaccaggactggctgaacggcaaggagtacaagt
IGHG3_30	gttccgtgtggtcagcgtcctcaccgtcctgcaccaggactggctgaacggcaaggagtacaagt
IGHG3_31	gttccgtgtggtcagcgtcctcaccgtcctgcaccaggactggctgaacggcaaggagtacaagt
IGHG4*01_IMGT	gtaccgtgtggtcagcgtcctcaccgtcctgcaccaggactggctgaacggcaaggagtacaagt
IGHG4_GRCh37	gtaccgtgtggtcagcgtcctcaccgtcctgcaccaggactggctgaacggcaaggagtacaagt
IGHG4_01	gtaccgtgtggtcagcgtcctcaccgtcctgcaccaggactggctgaacggcaaggagtacaagt
IGHG4_02	gtaccgtgtggtcagcgtcctcaccgtcctgcaccaggactggctgaacggcaaggagtacaagt
IGHG4_04	gtaccgtgtggtcagcgtcctcaccgtcctgcaccaggactggctgaacggcaaggagtacaagt
IGHG4_09	gtaccgtgtggtcagcgtcctcaccgtcctgcaccaggactggctgaacggcaaggagtacaagt
IGHG4_14	gtaccgtgtggtcagcgtcctcaccgtcctgcaccaggactggctgaacggcaaggagtacaagt
IGHG4_18	gtaccgtgtggtcagcgtcctcaccgtcctgcaccaggactggctgaacggcaaggagtacaagt
IGHG4_19	gtaccgtgtggtcagcgtcctcaccgtcctgcaccaggactggctgaacggcaaggagtacaagt
IGHG4_23	gtaccgtgtggtcagcgtcctcaccgtcctgcaccaggactggctgaacggcaaggagtacaagt
IGHG4_27	gtaccgtgtggtcagcgtcctcaccgtcctgcaccaggactggctgaacggcaaggagtacaagt
IGHG4_28	gtaccgtgtggtcagcgtcctcaccgtcctgcaccaggactggctgaacggcaaggagtacaagt
IGHG4_30	gtaccgtgtggtcagcatcctcaccgtcctgcaccaggactggctgaacggcaaggagtacaagt
IGHG4_31	gtaccgtgtggtcagcgtcctcaccgtcctgcaccaggactggctgaacggcaaggagtacaagt
IGHG4_32	gtaccgtgtggtcagcgtcctcaccgtcctgcaccaggactggctgaacggcaaggagtacaagt
IGHG4_37	gtaccgtgtggtcagcgtcctcaccgtcctgcaccaggactggctgaacggcaaggagtacaagt
IGHG4_40	gtaccgtgtggtcagcgtcctcaccatcctgcaccaggactggctgaacggcaaggagtacaagt
IGHG4_41	gtaccgtgtggtcagcgtcctcaccgtcctgcaccaggactggctgaacggcaaggagtacaagt
IGHG4_42	gtaccgtgtggtcagcgtcctcaccgtcctgcaccaggactggctgaacggcaaggagtacaagt

```
IGHG1*01_IMGT   gcaaggtctccaacaaagccctcccagcccccatcgagaaaaccatctccaaagccaaa
IGHG1_GRCh37    gcaaggtctccaacaaagccctcccagcccccatcgagaaaaccatctccaaagccaaa
IGHG1_01        gcaaggtctccaacaaagccctcccagcccccatcgagaaaaccatctccaaagccaaa
IGHG1_02        gcaaggtctccaacaaagccctcccagcccccatcgagaaaaccatctccaaagccaaa
IGHG1_05        gcaaggtctccaacaaagccctcccagcccccatcgagaaaaccatctccaaagccaaa
IGHG1_07        gcaaggtctccaacaaagccctcccagcccccatcgagaaaaccatctccaaagccaaa
IGHG1_10        gcaaggtctccaacaaagccctcccagcccccatcgagaaaaccatctccaaagccaaa
IGHG1_12        gcaaggtctccaacaaagccctcccagcccccatcgagaaaaccatctccaaagccaaa
IGHG1_14        gcaaggtctccaacaaagccctcccagcccccatcgagaaaaccatctccaaagccaaa
IGHG1_16        gcaaggtctccaacaaagccctcccagcccccatcgagaaaaccatctccaaagccaaa
IGHG1_18        gcaaggtctccaacaaagccctcccagcccccatcgagaaaaccatctccaaagccaaa
IGHG1_19        gcaaggtctccaacaaagccctcccagcccccatcgagaaaaccatctccaaagccaaa
IGHG2*01_IMGT   gcaaggtctccaacaaaggcctcccagcccccatcgagaaaaccatctccaaaaccaaa
IGHG2_GRCh37    gcaaggtctccaacaaaggcctcccagcccccatcgagaaaaccatctccaaaaccaaa
IGHG2_01        gcaaggtctccaacaaaggcctcccagcccccatcgagaaaaccatctccaaaaccaaa
IGHG2_02        gcaaggtctccaacaaaggcctcccagcccccatcgagaaaaccatctccaaaaccaaa
IGHG2_03        gcaaggtctccaacaaaggcctcccagcccccatcgagaaaaccatctccaaaaccaaa
IGHG2_04        gcaaggtctccaacaaaggcctcccagcccccatcgagaaaaccatctccaaaaccaaa
IGHG2_05        gcaaggtctccaacaaaggcctcccagcccccatcgagaaaaccatctccaaaaccaaa
IGHG2_06        gcaaggtctccaacaaaggcctcccagcccccatcgagaaaaccatctccaaaaccaaa
IGHG2_07        gcaaggtctccaacaaaggcctcccagcccccatcgagaaaaccatctccaaaaccaaa
IGHG2_08        gcaaggtctccaacaaaggcctcccagcccccatcgagaaaaccatctccaaaaccaaa
IGHG2_10        gcaaggtctccaacaaaggcctcccagcccccatcgagaaaaccatctccaaaaccaaa
IGHG2_13        gcaaggtctccaacaaaggcctcccagcccccatcgagaaaaccatctccaaaaccaaa
IGHG2_14        gcaaggtctccaacaaaggcctcccagcccccatcgagaaaaccatctccaaaaccaaa
IGHG2_16        gcaaggtctccaacaaaggcctcccagcccccatcgagaaaaccatctccaaaaccaaa
IGHG3*01_IMGT   gcaaggtctccaacaaagccctcccagcccccatcgagaaaaccatctccaaaaccaaa
IGHG3_GRCh37    gcaaggtctccaacaaagccctcccagcccccatcgagaaaaccatctccaaaaccaaa
IGHG3_01        gcaaggtctccaacaaagccctcccagcccccatcgagaaaaccatctccaaaaccaaa
IGHG3_03        gcaaggtctccaacaaagccctcccagcccccatcgagaaaaccatctccaaaaccaaa
IGHG3_05        gcaaggtctccaacaaagccctcccagcccccatcgagaaaaccatctccaaaaccaaa
IGHG3_10        gcaaggtctccaacaaagccctcccagcccccatcgagaaaaccatctccaaaaccaaa
IGHG3_11        gcaaggtctccaacaaagccctcccagcccccatcgagaaaaccatctccaaaaccaaa
IGHG3_12        gcaaggtctccaacaaagccctcccagcccccatcgagaaaaccatctccaaaaccaaa
IGHG3_16        gcaaggtctccaacaaagccctcccagcccccatcgagaaaaccatctccaaaaccaaa
IGHG3_17        gcaaggtctccaacaaagccctcccagcccccatcgagaaaaccatctccaaaaccaaa
IGHG3_23        gcaaggtctccaacaaagccctcccagcccccatcgagaaaaccatctccaaagccaaa
IGHG3_30        gcaaggtctccaacaaagccctcccagcccccatcgagaaaaccatctccaaaaccaaa
IGHG3_31        gcaaggtctccaacaaagccctcccagcccccatcgagaaaaccatctccaaaaccaaa
IGHG4*01_IMGT   gcaaggtctccaacaaaggcctcccgtcctccatcgagaaaaccatctccaaagccaaa
IGHG4_GRCh37    gcaaggtctccaacaaaggcctcccgtcctccatcgagaaaaccatctccaaagccaaa
IGHG4_01        gcaaggtctccaacaaaggcctcccgtcctccatcgagaaaaccatctccaaagccaaa
IGHG4_02        gcaaggtctccaacaaaggcctcccgtcctccatcgagaaaaccatctccaaagccaaa
IGHG4_04        gcaaggtctccaacaaaggcctcccgtcctccatcgagaaaaccatctccaaagccaaa
IGHG4_09        gcaaggtctccaacaaaggcctcccgtcctccatcgagaaaaccatctccaaagccaaa
IGHG4_14        gcaaggtctccaacaaaggcctcccgtcctccatcgagaaaaccatctccaaagccaaa
IGHG4_18        gcaaggtctccaacaaaggcctcccgtcctccatcgagaaaaccatctccaaagccaaa
IGHG4_19        gcaaggtctccaacaaaggcctcccgtcctccatcgagaaaaccatctccaaagccaaa
IGHG4_23        gcaaggtctccaacaaaggcctcccgtcctccatcgagaaaaccatctccaaagccaaa
IGHG4_27        gcaaggtctccaacaaaggcctcccgtcctccatcgagaaaaccatctccaaagccaaa
IGHG4_28        gcaaggtctccaacaaaggcctcccgtcctccatcgagaaaaccatctccaaagccaaa
IGHG4_30        gcaaggtctccaacaaaggcctcccgtcctccatcgagaaaaccatctccaaagccaaa
IGHG4_31        gcaaggtctccaacaaaggcctcccgtcctccatcgagaaaaccatctccaaagccaaa
IGHG4_32        gcaaggtctccaacaaaggcctcccgtcctccatcgagaaaaccatctccaaagccaaa
IGHG4_37        gcaaggtctccaacaaaggcctcccgtcctccatcgagaaaaccatctccaaagccaaa
IGHG4_40        gcaaggtctcccacaaaggcctcccgtcctccatcgagaaaaccatctccaaagccaaa
IGHG4_41        gcaaggtctccaacaaaggcctcccgtcctccatcgagaaaaccatctccaaagccaaa
IGHG4_42        gcaaggtctccaacaaaggcctcccgtcctccatcgagaaaaccatctccaaaaccaaa
```

CH3-CHS

IGHG1*01_IMGT	gggcagccccgagaaccacaggtgtacaccctgcccccatcccgggatgagctgaccaagaaccaggtca
IGHG1_GRCh37	gggcagccccgagaaccacaggtgtacaccctgcccccatcccgggatgagctgaccaagaaccaggtca
IGHG1_01	gggcagccccgagaaccacaggtgtacaccctgcccccatcccgggatgagctgaccaagaaccaggtca
IGHG1_02	gggcagccccgagaaccacaggtgtacaccctgcccccatcccgggatgagctgaccaagaaccaggtca
IGHG1_05	gggcagccccgagaaccacaggtgtacaccctgcccccatcccgggatgagctgaccaagaaccaggtca
IGHG1_07	gggcagccccgagaaccacaggtgtacaccctgcccccatcccgggatgagctgaccaagaaccaggtca
IGHG1_10	gggcagccccgagaaccacaggtgtacaccctgcccccatcccgggatgagctgaccaagaaccaggtca
IGHG1_12	gggcagccccgagaaccacaggtgtacaccctgcccccatcccgggatgagctgaccaagaaccaggtca
IGHG1_14	gggcagccccgagaaccacaggtgtataccctgcccccatcccgggatgagctgaccaagaaccaggtca
IGHG1_16	gggcagccccgagaaccacaggtgtacaccctgcccccatcccgggatgagctgaccaagaaccaggtca
IGHG1_18	gggcagccccgagaaccacaggtgtacaccctgcccccatcccgggatgagctgaccaagaaccaggtca
IGHG1_19	gggcagccccgagaaccacaggtgtacaccctgcccccatcccgggatgagctgaccaagaaccaggtca
IGHG2*01_IMGT	gggcagccccgagaaccacaggtgtacaccctgcccccatcccgggaggagatgaccaagaaccaggtca
IGHG2_GRCh37	gggcagccccgagaaccacaggtgtacaccctgcccccatcccgggaggagatgaccaagaaccaggtca
IGHG2_01	gggcagccccgagaaccacaggtgtacaccctgcccccatcccgggaggagatgaccaagaaccaggtca
IGHG2_02	gggcagccccgagaaccacaggtgtacaccctgcccccatcccgggaggagatgaccaagaaccaggtca
IGHG2_03	gggcagccccgagaaccacaggtgtacaccctgcccccatcccgggaggagatgaccaagaaccaggtca
IGHG2_04	gggcagccccgagaaccacaggtgtacaccctgcccccatcccgggaggagatgaccaagaaccaggtca
IGHG2_05	gggcagccccgagaaccacaggtgtacaccctgcccccatcccgggaggagatgaccaagaaccaggtca
IGHG2_06	gggcagccccgagaaccacaggtgtacaccctgcccccatcccgggaggagatgaccaagaaccaggtca
IGHG2_07	gggcagccccgagaaccacaggtgtacaccctgcccccatcccgggaggagatgaccaagaaccaggtca
IGHG2_08	gggcagccccgagaaccacaggtgtacaccctgcccccatcccgggaggagatgaccaagaaccaggtca
IGHG2_10	gggcagccccgagaaccacaggtgtacaccctgcccccatcccgggaggagatgaccaagaaccaggtca
IGHG2_13	gggcagccccgagaaccacaggtgtacaccctgcccccatcccgggaggagatgaccaagaaccaggtca
IGHG2_14	gggcagccccgagaaccacaggtgtacaccctgcccccatcccgggaggagatgaccaagaaccaggtca
IGHG2_16	gggcagccccgagaaccacaggtgtacaccctgcccccatcccgggaggagatgaccaagaaccaggtca
IGHG3*01_IMGT	ggacagccccgagaaccacaggtgtacaccctgcccccatcccgggaggagatgaccaagaaccaggtca
IGHG3_GRCh37	ggacagccccgagaaccacaggtgtacaccctgcccccatcccgggaggagatgaccaagaaccaggtca
IGHG3_01	ggacagccccgagaaccacaggtgtacaccctgcccccatcccgggaggagatgaccaagaaccaggtca
IGHG3_03	ggacagccccgagaaccacaggtgtacaccctgcccccatcccgggaggagatgaccaagaaccaggtca
IGHG3_05	ggacagccccgagaaccacaggtgtacaccctgcccccatcccgggaggagatgaccaagaaccaggtca
IGHG3_10	ggacagccccgagaaccacaggtgtacaccctgcccccatcccgggaggagatgaccaagaaccaggtca
IGHG3_11	ggacagccccgagaaccacaggtgtacaccctgcccccatcccgggaggagatgaccaagaaccaggtca
IGHG3_12	ggacagccccgagaaccacaggtgtacaccctgcccccatcccgggaggagatgaccaagaaccaggtca
IGHG3_16	ggacagccccgagaaccacaggtgtacaccctgcccccatcccgggaggagatgaccaagaaccaggtca
IGHG3_17	ggacagccccgagaaccacaggtgtacaccctgcccccatcccgggaggagatgaccaagaaccaggtca
IGHG3_23	ggacagccccgagaaccacaggtgtacaccctgcccccatcccgggaggagatgaccaagaaccaggtca
IGHG3_30	ggacagccccgagaaccacaggtgtacaccctgcccccatcccgggaggagatgaccaagaaccaggtca
IGHG3_31	ggacagccccgagaaccacaggtgtacaccctgcccccatcccgggaggagatgaccaagaaccaggtca
IGHG4*01_IMGT	gggcagccccgagagccacaggtgtacaccctgcccccatcccaggaggagatgaccaagaaccaggtca
IGHG4_GRCh37	gggcagccccgagagccacaggtgtacaccctgcccccatcccaggaggagatgaccaagaaccaggtca
IGHG4_01	gggcagccccgagagccacaggtgtacaccctgcccccatcccaggaggagatgaccaagaaccaggtca
IGHG4_02	gggcagccccgagagccacaggtgtacaccctgcccccatcccaggaggagatgaccaagaaccaggtca
IGHG4_04	gggcagccccgagagccacaggtgtacaccctgcccccatcccaggaggagatgaccaagaaccaggtca
IGHG4_09	gggcagccccgagagccacaggtgtacaccctgcccccatcccaggaggagatgaccaagaaccaggtca
IGHG4_14	gggcagccccgagagccacaggtgtacaccctgcccccatcccaggaggagatgaccaagaaccaggtca
IGHG4_18	gggcagccccgagagccacaggtgtacaccctgcccccatcccaggaggagatgaccaagaaccaggtca
IGHG4_19	gggcagccccgagagccacaggtgtacaccctgcccccatccccggaggagatgaccaagaaccaggtca
IGHG4_23	gggcagccccgagagccacaggtgtacaccctgcccccatcccaggaggagatgaccaagaaccaggtca
IGHG4_27	gggcagccccgagagccacaggtgtacaccctccccccatcccaggaggagatgaccaagaaccaggtca
IGHG4_28	gggcagccccgagagccacaggtgtacaccctgcccccatcccaggaggagatgaccaagaaccaggtca
IGHG4_30	gggcagccccgagagccacaggtgtacaccctgcccccatcccaggaggagatgaccaagaaccaggtca
IGHG4_31	gggcagccccgagagccacaggtgtacaccctgcccccatcccaggaggagatgaccaagaaccaggtca
IGHG4_32	gggcagccccgagagccacaggtgtacaccctccccccatcccaggaggagatgaccaagaaccaggtca
IGHG4_37	gggcagccccgagagccacaggtgtacaccctgcccccatcccaggaggagatgaccaagaaccaggtca
IGHG4_40	gggcagccccgagagccacaggtgtacaccctgcccccatcccaggaggagatgaccaagaaccaggtca
IGHG4_41	gggcagccccgagagccacaggtgtacaccctgcccccatcccaggaggagatgaccaagaaccaggtca
IGHG4_42	gggcagccccgagagccacaggtgtacaccctgcccccatcccaggaggagatgaccaagaaccaggtca

IGHG1*01_IMGT	gcctgacctgcctggtcaaaggcttctatcccagcgacatcgccgtggagtgggagagcaatgggca
IGHG1_GRCh37	gcctgacctgcctggtcaaaggcttctatcccagcgacatcgccgtggagtgggagagcaatgggca
IGHG1_01	gcctgacctgcctggtcaaaggcttctatcccagcgacatcgccgtggagtgggagagcaatgggca
IGHG1_02	gcctgacctgcctggtcaaaggcttctatcccagcgacatcgccgtggagtgggagagcaatgggca
IGHG1_05	gcctgacctgcctggtcaaaggcttctatcccagcgacatcgccgtggagtgggagagcaatgggca
IGHG1_07	gcctgacctgcctggtcaaaggcttctatcccagcgacatcgccgtggagtgggagagcaatgggca
IGHG1_10	gcctgacctgcctggtcaaaggcttctatcccagcgacatcgccgtggagtgggagagcaatgggca
IGHG1_12	gcctgacctgcctggtcaaaggcttctatcccagcgacatcgccgtggagtgggagagcaatgggca
IGHG1_14	gcctgacctgcctggtcaaaggcttctatcccagcgacatcgccgtggagtgggagagcaatgggca
IGHG1_16	gcctgacctgcctggtcaaaggcttctatcccagcgacatcgccgtggagtgggagagcaatgggca
IGHG1_18	gcctgacctgcctggtcaaaggcttctatcccagcgacatcgccgtggagtgggagagcaatgggca
IGHG1_19	gcctgacctgcctggtcaaaggcttctatcccagcgacatcgccgtggagtgggagagcaatgggca
IGHG2*01_IMGT	gcctgacctgcctggtcaaaggcttctacccccagcgacatcgccgtggagtgggagagcaatgggca
IGHG2_GRCh37	gcctgacctgcctggtcaaaggcttctacccccagcgacatctccgtggagtgggagagcaatgggca
IGHG2_01	gcctgacctgcctggtcaaaggcttctacccccagcgacatcgccgtggagtgggagagcaatgggca
IGHG2_02	gcctgacctgcctggtcaaaggcttctacccccagcgacatcgccgtggagtgggagagcaatgggca
IGHG2_03	gcctgacctgcctggtcaaaggcttctacccccagcgacatcgccgtggagtgggagagcaatgggca
IGHG2_04	gcctgacctgcctggtcaaaggcttctacccccagcgacatctccgtggagtgggagagcaatgggca
IGHG2_05	gcctgacctgcctggtcaaaggcttctacccccagcgacatcgccgtggagtgggagagcaatgggca
IGHG2_06	gcctgacctgcctggtcaaaggcttctacccccagcgacatcgccgtggagtgggagagcaatgggca
IGHG2_07	gcctgacctgcctggtcaaaggcttctacccccagcgacatcgccgtggagtgggagagcaatgggca
IGHG2_08	gcctgacctgcctggtcaaaggcttctacccccagcgacatcgccgtggagtgggagagcaatgggca
IGHG2_10	gcctgacctgcctggtcaaaggcttctacccccagcgacatcgccgtggagtgggagagcaatgggca
IGHG2_13	gcctgacctgcctggtcaaaggcttctacccccagcgacatcgccgtggagtgggagagcaatgggca
IGHG2_14	gcctgacctgcctggtcaaaggcttctacccccagcgacatcgccgtggagtgggagagcaatgggca
IGHG2_16	gcctgacctgcctggtcaaaggcttctacccccagcgacatcgccgtggagtgggagagcaatgggca
IGHG3*01_IMGT	gcctgacctgcctggtcaaaggcttctacccccagcgacatcgccgtggagtgggagagcagcgggca
IGHG3_GRCh37	gcctgacctgcctggtcaaaggcttctacccccagcgacatcgccgtggagtgggagagcagcgggca
IGHG3_01	gcctgacctgcctggtcaaaggcttctacccccagcgacatcgccgtggagtgggagagcagcgggca
IGHG3_03	gcctgacctgcctggtcaaaggcttctacccccagcgacatcgccgtggagtgggagagcaatgggca
IGHG3_05	gcctgacctgcctggtcaaaggcttctacccccagcgacatcgccgtggagtgggagagcaatgggca
IGHG3_10	gcctgacctgcctggtcaaaggcttctacccccagcgacatcgccgtggagtgggagagcagcgggca
IGHG3_11	gcctgacctgcctggtcaaaggcttctacccccagcgacatcgccgtggagtgggagagcagcgggca
IGHG3_12	gcctgacctgcctggtcaaaggcttctacccccagcgacatcgccgtggagtgggagagcagcgggca
IGHG3_16	gcctgacctgcctggtcaaaggcttctacccccagcgacatcgccgtggagtgggagagcagcgggca
IGHG3_17	gcctgacctgcctggtcaaaggcttctacccccagcgacatcgccgtggagtgggagagcagcgggca
IGHG3_23	gcctgacctgcctggtcaaaggcttctacccccagcgacatcgccgtggagtgggagagcaatgggca
IGHG3_30	gcctgacctgcctggtcaaaggcttctacccccagcgacatcgccgtggagtgggagagcagcgggca
IGHG3_31	gcctgacctgcctggtcaaaggcttctacccccagcgacatcgccgtggagtgggagagcagcgggca
IGHG4*01_IMGT	gcctgacctgcctggtcaaaggcttctacccccagcgacatcgccgtggagtgggagagcaatgggca
IGHG4_GRCh37	gcctgacctgcctggtcaaaggcttctacccccagcgacatcgccgtggagtgggagagcaatgggca
IGHG4_01	gcctgacctgcctggtcaaaggcttctacccccagcgacatcgccgtggagtgggagagcaatgggca
IGHG4_02	gcctgacctgcctggtcaaaggcttctacccccagcgacatcgccgtggagtgggagagcaatgggca
IGHG4_04	gcctgacctgcctggtcaaaggcttctacccccagcgacatcgccgtggagtgggagagcaatgggca
IGHG4_09	gcctgacctgcctggtcaaaggcttctacccccagcgacatcgccgtggagtgggagagcaatgggca
IGHG4_14	gcctgacctgcctggtcaaaggcttctacccccagcgacatcgccgtggagtgggagagcaatgggca
IGHG4_18	gcctgacctgcctggtcaaaggcttctacccccagcgacatcgccgtggagtgggagagcaatgggca
IGHG4_19	gcctgacctgcctggtcaaaggcttctacccccagcgacatcgccgtggagtgggagagcaatgggca
IGHG4_23	gcctgacctgcctggtcaaaggcttctacccccagcgacatcgccgtggagtgggagagcaatgggca
IGHG4_27	gcctgacctgcctggtcaaaggcttctacccccagcgacatcgccgtggagtgggagagcaatgggca
IGHG4_28	gcctgacctgcctggtcaaaggcttctacccccagcgacatcgccgtggagtgggagagcaatgggca
IGHG4_30	gcctgacctgcctggtcaaaggcttctacccccagcgacatcgccgtggagtgggagagcaatgggca
IGHG4_31	gcctgacctgcctggtcaaaggcttctacccccagcgacatcgccgtggagtgggagagcaatgggca
IGHG4_32	gcctgacctgcctggtcaaaggcttctacccccagcgacatcgccgtggagtgggagagcaatgggca
IGHG4_37	gcctgacctgcctggtcaaaggcttctacccccagtgacatcgccgtggagtgggagagcaatgggca
IGHG4_40	gcctgacctgcctggtcaaaggcttctacccccagcgacatcgccgtggagtgggagagcaatgggca
IGHG4_41	gcctgacctgcctggtcaaaggcttctacccccagcgacatcgccgtggagtgggagagcaatgggca
IGHG4_42	gcctgacctgcctggtcaaaggcttctacccccagcgacatcgccgtggagtgggagagcaatgggca

IGHG1*01_IMGT	gccggagaacaactacaagaccacgcctcccgtgctggactccgacggctccttcttcctctacagc
IGHG1_GRCh37	gccggagaacaactacaagaccacgcctcccgtgctggactccgacggctccttcttcctctacagc
IGHG1_01	gccggagaacaactacaagaccacgcctcccgtgctggactccgacggctccttcttcctctacagc
IGHG1_02	gccggagaacaactacaagaccacgcctcccgtgctggactccgacggctccttcttcctctacagc
IGHG1_05	gccggagaacaactacaagaccacgcctcccgtgctggactccgacggctccttcttcctctacagc
IGHG1_07	gccggagaacaactacaagaccacgcctcccgtgctggactccgacggctccttcttcctctatagc
IGHG1_10	gccggagaacaactacaagaccacgcctcccgtgctggactccgacggctccttcttcctctatagc
IGHG1_12	gccggagaacaactacaagaccacgcctcccgtgctggactccgacggctccttcttcctctacagc
IGHG1_14	gccggagaacaactacaagaccacgcctcccgtgctggactccgacggctccttcttcctctacagc
IGHG1_16	gccggagaacaactacaagaccacgcctcccgtgctggactccgacggctccttcttcctctacagc
IGHG1_18	gccggagaacaactacaagaccacgcctcccgtgctggactccgacggctccttcttcctctacagc
IGHG1_19	gccggagaacaactacaagaccacgcctcccgtgctggactccgacggctccttcttcctctacagc
IGHG2*01_IMGT	gccggagaacaactacaagaccacacctcccatgctggactccgacggctccttcttcctctacagc
IGHG2_GRCh37	gccggagaacaactacaagaccacacctcccatgctggactccgacggctccttcttcctctacagc
IGHG2_01	gccggagaacaactacaagaccacacctcccatgctggactccgacggctccttcttcctctacagc
IGHG2_02	gccggagaacaactacaagaccacacctcccatgctggactccgacggctccttcttcctctacagc
IGHG2_03	gccggagaacaactacaagaccacacctcccatgctggactccgacggctccttcttcctctacagc
IGHG2_04	gccggagaacaactacaagaccacacctcccatgctggactccgacggctccttcttcctctacagc
IGHG2_05	gccggagaacaactacaagaccacacctcccatgctggactccgacggctccttcttcctctacagc
IGHG2_06	gccggagaacaactacaagaccacacctcccatgctggactccgacggctccttcttcctctacagc
IGHG2_07	gccggagaacaactacaagaccacacctcccatgctggactccgacggctccttcttcctctacagc
IGHG2_08	gccggagaacaactacaagaccacacctcccatgctggactccgacggctccttcttcctctacagc
IGHG2_10	gccggagaacaactacaagaccacacctcccatgctggactccgacggctccttcttcctctacagc
IGHG2_13	gccggagaacaactacaagaccacacctcccatgctggactccgacggctccttcttcctctacagc
IGHG2_14	gccggagaacaactacaagaccacacctcccatgctggactccgacggctccttcttcctctacagc
IGHG2_16	gccggagaacaactacaagaccacacctcccatgctggactccgacggctccttcttcctctacagc
IGHG3*01_IMGT	gccggagaacaactacaacaccacgcctcccatgctggactccgacggctccttcttcctctacagc
IGHG3_GRCh37	gccggagaacaactacaacaccacgcctcccatgctggactccgacggctccttcttcctctacagc
IGHG3_01	gccggagaacaactacaacaccacgcctcccatgctggactccgacggctccttcttcctctacagc
IGHG3_03	gccggagaacaactacaacaccacgcctcccatgctggactccgacggctccttcttcctctacagc
IGHG3_05	gccggagaacaactacaacaccacgcctcccatgctggactccgacggctccttcttcctctacagc
IGHG3_10	gccggagaacaactacaacaccacgcctcccatgctggactccgacggctccttcttcctctacagc
IGHG3_11	gccggagaacaactacaacaccacgcctcccatgctggactccgacggctccttcttcctctacagc
IGHG3_12	gccggagaacaactacaacaccacgcctcccatgctggactccgacggctccttcttcctctacagc
IGHG3_16	gccggagaacaactacaacaccacgcctcccatgctggactccgacggctccttcttcctctacagc
IGHG3_17	gccggagaacaactacaacaccacgcctcccatgctggactccgacggctccttcttcctctacagc
IGHG3_23	gccggagaacaactacaacaccacgcctcccatgctggactccgacggctccttcttcctctacagc
IGHG3_30	gccggagaacaactacaacaccacgcctcccatgctggactccgacggctccttcttcctctacagc
IGHG3_31	gccggagaacaactacaacaccacgcctcccatgctggactccgacggctccttcttcctctacagc
IGHG4*01_IMGT	gccggagaacaactacaagaccacgcctcccgtgctggactccgacggctccttcttcctctacagc
IGHG4_GRCh37	gccggagaacaactacaagaccacgcctcccgtgctggactccgacggctccttcttcctctacagc
IGHG4_01	gccggagaacaactacaagaccacgcctcccgtgctggactccgacggctccttcttcctctacagc
IGHG4_02	gccggagaacaactacaagaccacgcctcccgtgctggactccgacggctccttcttcctctacagc
IGHG4_04	gccggagaacaactacaagaccacgcctcccgtgctggactccgacggctccttcttcctctacagc
IGHG4_09	gccggagaacaactacaagaccacgcctcccgtgctggactccgacggctccttcttcctctacagc
IGHG4_14	gccggagaacaactacaagaccacgcctcccgtgctggactccgacggctccttcttcctctacagc
IGHG4_18	gccggagaacaactacaagaccacgcctcccgtgctggactccgacggctccttcttcctctacagc
IGHG4_19	gccggagaacaactacaagaccacgcctcccgtgctggactccgacggctccttcttcctctacagc
IGHG4_23	gccggagaacaactacaagaccacgcctcccgtgctggactccgacggctccttcttcctctacagc
IGHG4_27	gccggagaacaactacaagaccacgcctcccgtgctggactccgacggctccttcttcctctacagc
IGHG4_28	gccggagaacaactacaagaccacgcctcccgtgctggactccgacggctccttcttcctctacagc
IGHG4_30	gccggagaacaactacaagaccacgcctcccgtgctggactccgacggctccttcttcctctacagc
IGHG4_31	gccggagaacaactacaagaccacgcctcccgtgctggactccgacggctccttcttcctctacagc
IGHG4_32	gccggagaacaactacaagaccacgcctcccgtgctggactccgacggctccttcttcctctacagc
IGHG4_37	gccggagaacaactacaagaccacgcctcccgtgctggactccgacggctccttcttcctctacagc
IGHG4_40	gccggagaacaactacaagaccacgcctcccgtgctggactccgacggctccttcttcctctacagc
IGHG4_41	gccggagaacaactacaagaccacgcctcccgtgctggactccgacggctccttcttcctctacagc
IGHG4_42	gccggagaacaactacaagaccacgcctcccgtgctggactccgacggctccttcttcctctacagc

```
IGHG1*01_IMGT   aagctcaccgtggacaagagcaggtggcagcaggggaacgtcttctcatgctccgtgatgcatga
IGHG1_GRCh37    aagctcaccgtggacaagagcaggtggcagcaggggaacgtcttctcatgctccgtgatgcatga
IGHG1_01        aagctcaccgtggacaagagcaggtggcagcaggggaacgtcttctcatgctccgtgatgcatga
IGHG1_02        aagctcaccgtggacaagagcaggtggcagcaggggaacgtcttctcatgctccgtgatgcatga
IGHG1_05        aagctcaccgtggacaagagcaggtggcagcaggggaacgtcttctcatgctccgtgatgcatga
IGHG1_07        aagctcaccgtggacaagagcaggtggcagcaggggaacgtcttctcatgctccgtgatgcatga
IGHG1_10        aagctcaccgtggacaagagcaggtggcagcaggggaacgtcttctcatgctccgtgatgcatga
IGHG1_12        aagctcaccgtggacaagagcaggtggcagcaggggaacgtcttctcatgctccgtgatgcatga
IGHG1_14        aagctcaccgtggacaagagcaggtggcagcaggggaacgtcttctcatgctccgtgatgcatga
IGHG1_16        aagctcaccgtggacaagagcaggtggcagcaggggaacgtcttctcatgctccgtgatgcatga
IGHG1_18        aagctcaccgtggacaagagcaggtggcagcaggggaacgtcttctcatgctccgtgatgcatga
IGHG1_19        aagctcaccgtggacaagagcaggtggcagcaggggaacgtcttctcatgctccgtgatgcatga
IGHG2*01_IMGT   aagctcaccgtggacaagagcaggtggcagcaggggaacgtcttctcatgctccgtgatgcatga
IGHG2_GRCh37    aagctcaccgtggacaagagcaggtggcagcaggggaacgtcttctcatgctccgtgatgcatga
IGHG2_01        aagctcaccgtggacaagagcaggtggcagcaggggaacgtcttctcatgctccgtgatgcatga
IGHG2_02        aagctcaccgtggacaagagcaggtggcagcaggggaacgtcttctcatgctccgtgatgcatga
IGHG2_03        aagctcaccgtggacaagagcaggtggcagcaggggaacgtcttctcatgctccgtgatgcatga
IGHG2_04        aagctcaccgtggacaagagcaggtggcagcaggggaacgtcttctcatgctccgtgatgcatga
IGHG2_05        aagctcaccgtggacaagagcaggtggcagcaggggaacgtcttctcatgctccgtgatgcatga
IGHG2_06        aagctcaccgtggacaagagcaggtggcagcaggggaacgtcttctcatgctccgtgatgcatga
IGHG2_07        aagctcaccgtggacaagagcaggtggcagcaggggaacgtcttctcatgctccgtgatgcatga
IGHG2_08        aagctcaccgtggacaagagcaggtggcagcaggggaacgtcttctcatgctccgtgatgcatga
IGHG2_10        aagctcaccgtggacaagagcaggtggcagcaggggaacgtcttctcatgctccgtgatgcatga
IGHG2_13        aagctcaccgtggacaagagcaggtggcagcaggggaacgtcttctcatgctccgtgatgcatga
IGHG2_14        aagctcaccgtggacaagagcaggtggcagcaggggaacgtcttctcatgctccgtgatgcatga
IGHG2_16        aagctcaccgtggacaagagcaggtggcagcaggggaacgtcttctcatgctccgtgatgcatga
IGHG3*01_IMGT   aagctcaccgtggacaagagcaggtggcagcaggggaacatcttctcatgctccgtgatgcatga
IGHG3_GRCh37    aagctcaccgtggacaagagcaggtggcagcaggggaacatcttctcatgctccgtgatgcatga
IGHG3_01        aagctcaccgtggacaagagcaggtggcagcaggggaacatcttctcatgctccgtgatgcatga
IGHG3_03        aagctcaccgtggacaagagcaggtggcagcaggggaacatcttctcatgctccgtgatgcatga
IGHG3_05        aagctcaccgtggacaagagcaggtggcagcaggggaacatcttctcatgctccgtgatgcatga
IGHG3_10        aagctcaccgtggacaagagcaggtggcagcaggggaacatcttctcatgctccgtgatgcatga
IGHG3_11        aagctcaccgtggacaagagcaggtggcagcaggggaacatcttctcatgctccgtgatgcatga
IGHG3_12        aagctcaccgtggacaagagcaggtggcagcaggggaacatcttctcatgctccgtgatgcatga
IGHG3_16        aagctcaccgtggacaagagcaggtggcagcaggggaacatcttctcatgctccgtgatgcatga
IGHG3_17        aagctcaccgtggacaagagcaggtggcagcaggggaacatcttctcatgctccgtgatgcatga
IGHG3_23        aagctcaccgtggacaagagcaggtggcagcaggggaacatcttctcatgctccgtgatgcatga
IGHG3_30        aagctcaccgtggacaagagcaggtggcagcaggggaacatcttctcatgctccgtgatgcatga
IGHG3_31        aagctcaccgtggacaagagcaggtggcagcaggggaacatcttctcatgctccgtgatgcatga
IGHG4*01_IMGT   aggctaaccgtggacaagagcaggtggcaggaggggaatgtcttctcatgctccgtgatgcatga
IGHG4_GRCh37    aggctcaccgtggacaagagcaggtggcaggaggggaatgtcttctcatgctccgtgatgcatga
IGHG4_01        aggctaaccgtggacaagagcaggtggcaggaggggaatgtcttctcatgctccgtgatgcatga
IGHG4_02        aggctcaccgtggacaagagcaggtggcaggaggggaatgtcttctcatgctccgtgatgcatga
IGHG4_04        aggctaaccgtggacaagagcaggtggcaggaggggaatgtcttctcatgctccgtgatgcatga
IGHG4_09        aggctaaccgtggacaagagcaggtggcaggaggggaatgtcttctcatgctccgtgatgcatga
IGHG4_14        aggctaaccgtggacaagagcaggtggcaggaggggaatgtcttctcatgctccgtgatgcatga
IGHG4_18        aggctcaccgtggacaagagcaggtggcaggaggggaatgtcttctcatgctccgtgatgcatga
IGHG4_19        aggctaaccgtggacaagagcaggtggcaggaggggaatgtcttctcatgctccgtgatgcatga
IGHG4_23        aggctaaccgtggacaagagcaggtggcaggaggggaatgtcttctcatgctccgtgatgcatga
IGHG4_27        aggctcaccgtggacaagagcaggtggcaggaggggaatgtcttctcatgctccgtgatgcatga
IGHG4_28        aggctaaccgtggacaagagcaggtggcaggaggggaatgtcttctcatgctccgtgatgcatga
IGHG4_30        aggctaaccgtggacaagagcaggtggcaggaggggaatgtcttctcatgctccgtgatgcatga
IGHG4_31        aggctaaccgtggacaagagcaggtggcaggaggggaatgtcttctcatgctccgtgatgcatga
IGHG4_32        aggctcaccgtggacaagagcaggtggcaggaggggaatgtcttctcatgctccgtgatgcatga
IGHG4_37        aggctaaccgtggacaagagcaggtggcaggaggggaatgtcttctcatgctccgtgatgcatga
IGHG4_40        aggctcaccgtggacaagagcaggtggcaggaggggaatgtcttctcatgctccgtgatgcatga
IGHG4_41        aggctcaccgtggacaagagcaggtggcaggaggggaatgtcttctcatgctccgtgatgcatga
IGHG4_42        aggctaaccgtggacaagagcaggtggcaggaggggaatgtcttctcatgctccgtgatgcatga
```

```
IGHG1*01_IMGT   ggctctgcacaaccactacacgcagaagagcctctccctgtctccgggtaaa
IGHG1_GRCh37    ggctctgcacaaccactacacacagaagagcctctccctgtctccgggtaaa
IGHG1_01        ggctctgcacaaccactacacgcagaagagcctctccctgtctccgggtaaa
IGHG1_02        ggctctgcacaaccactacacacagaagagcctctccctgtctccgggtaaa
IGHG1_05        gggtctgcacaaccactacacgcagaagagcctctccctgtctccgggtaaa
IGHG1_07        ggctctgcacaaccactacacacagaagagcctctccctgtctccgggtaaa
IGHG1_10        ggctctgcacaaccactacacgcagaagagcctctccctgtctccgggtaaa
IGHG1_12        ggctctgcacaaccactacacgcagaagagcctctccctgtctccgggtaaa
IGHG1_14        ggctctgcacaaccactacacgcagaagagcctctccctgtctccgggtaaa
IGHG1_16        ggctctgcacaaccactacacacagaagagcctctccctgtctccgggtaaa
IGHG1_18        ggctctgcacaaccactacacacagaagagcctctccctgtctccgggtaaa
IGHG1_19        ggctctgcacaaccactacacgcagaagagcctctccctgtctccgggtaaa
IGHG2*01_IMGT   ggctctgcacaaccactacacgcagaagagcctctccctgtctccgggtaaa
IGHG2_GRCh37    ggctctgcacaaccactacacacagaagagcctctccctgtctccgggtaaa
IGHG2_01        ggctctgcacaaccactacacacagaagagcctctccctgtctccgggtaaa
IGHG2_02        ggctctgcacaaccactacacgcagaagagcctctccctgtctccgggtaaa
IGHG2_03        ggctctgcacaaccactacacgcagaagagcctctccctgtctccgggtaaa
IGHG2_04        ggctctgcacaaccactacacacagaagagcctctccctgtctccgggtaaa
IGHG2_05        ggctctgcacaaccactacacacagaagagcctctccctgtctccgggtaaa
IGHG2_06        ggctctgcacaaccactacacgcagaagagcctctccctgtctccgggtaaa
IGHG2_07        ggctctgcacaaccactacacgcagaagagcctctccctgtctccgggtaaa
IGHG2_08        ggctctgcacaaccactacacgcagaagagcctctccctgtctccgggtaaa
IGHG2_10        ggctctgcacaaccactacacacagaagagcctctccctgtctccgggtaaa
IGHG2_13        ggctctgcacaaccactacacgcagaagagcctctccctgtctccgggtaaa
IGHG2_14        ggctctgcacaaccactacacgcagaagagcctctccctgtctccgggtaaa
IGHG2_16        ggctctgcacaaccactacacgcagaagagcctctccctgtctccgggtaaa
IGHG3*01_IMGT   ggctctgcacaaccgcttcacgcagaagagcctctccctgtctccgggtaaa
IGHG3_GRCh37    ggctctgcacaaccgcttcacgcagaagagcctctccctgtctccgggtaaa
IGHG3_01        ggctctgcacaaccgcttcacgcagaagagcctctccctgtctccgggtaaa
IGHG3_03        ggctctgcacaaccgcttcacgcagaagagcctctccctgtctccgggtaaa
IGHG3_05        ggctctgcacaaccgctacacgcagaagagcctctccctgtctccgggtaaa
IGHG3_10        ggctctgcacaaccgcttcacgcagaagagcctctccctgtctccgggtaaa
IGHG3_11        ggctctgcacaaccgcttcacgcagaagagcctctccctgtctccgggtaaa
IGHG3_12        ggctctgcacaaccgctacacgcagaagagcctctccctgtctccgggtaaa
IGHG3_16        ggctctgcacaaccgctacacgcagaagagcctctccctgtctccgggtaaa
IGHG3_17        ggctctgcacaaccgcttcacgcagaagagcctctccctgtctccgggtaaa
IGHG3_23        ggctctgcacaaccgctacacgcagaagagcctctccctgtctccgggtaaa
IGHG3_30        ggctctgcacaaccgctacacgcagaagagcctctccctgtctccgggtaaa
IGHG3_31        ggctctgcacaaccgctacacgcagaagagcctctccctgtctccgggtaaa
IGHG4*01_IMGT   ggctctgcacaaccactacacacagaagagcctctccctgtctctgggtaaa
IGHG4_GRCh37    ggctctgcacaaccactacacacagaagagcctctccctgtctctgggtaaa
IGHG4_01        ggctctgcacaaccactacacacagaagagcctctccctgtctctgggtaaa
IGHG4_02        ggctctgcacaaccactacacacagaagagcctctccctgtctctgggtaaa
IGHG4_04        ggctctgcacaaccactacacacagaagagcctctccctgtctctgggtaaa
IGHG4_09        ggctctgcacaaccactacacacagaagagcctctccctgtctctgggtaaa
IGHG4_14        ggctctgcacaaccactacacacagaagagcctctccctgtctctgggtaaa
IGHG4_18        ggctctgcacaaccactacacacagaagagcctctccctgtctctgggtaaa
IGHG4_19        ggctctgcacaaccactacacacagaagagcctctccctgtctctgggtaaa
IGHG4_23        ggctctgcacaaccactacacacagaagagcctctccctgtctctgggtaaa
IGHG4_27        ggctctgcacaaccactacacacagaagagcctctccctgtctctgggtaaa
IGHG4_28        ggctctgcacaaccactacacacagaagagcctctccctgtctctgggtaaa
IGHG4_30        ggctctgcacaaccactacacacagaagagcctctccctgtctctgggtaaa
IGHG4_31        ggctctgcacaaccactacacacagaagagcctctccctgtctctgggtaaa
IGHG4_32        ggctctgcacaaccactacacacagaagagcctctccctgtctctgggtaaa
IGHG4_37        ggctctgcacaaccactacacacagaagagcctctccctgtctctgggtaaa
IGHG4_40        ggctctgcacaaccactacacacagaagagcctctccctgtctctgggtaaa
IGHG4_41        ggctctgcacaaccactacacacagaagagcctctccctgtctctgggtaaa
IGHG4_42        ggctctgcacaaccactacacacagaagagcctctccctgtctctgggtaaa
```

Figure S8: Alignment of CH1, CH2 and CH3 domain for *IGHA1*, *IGHA2* group and *IGHG1*, *IGHG2*, *IGHG3* and *IGHG4* group.

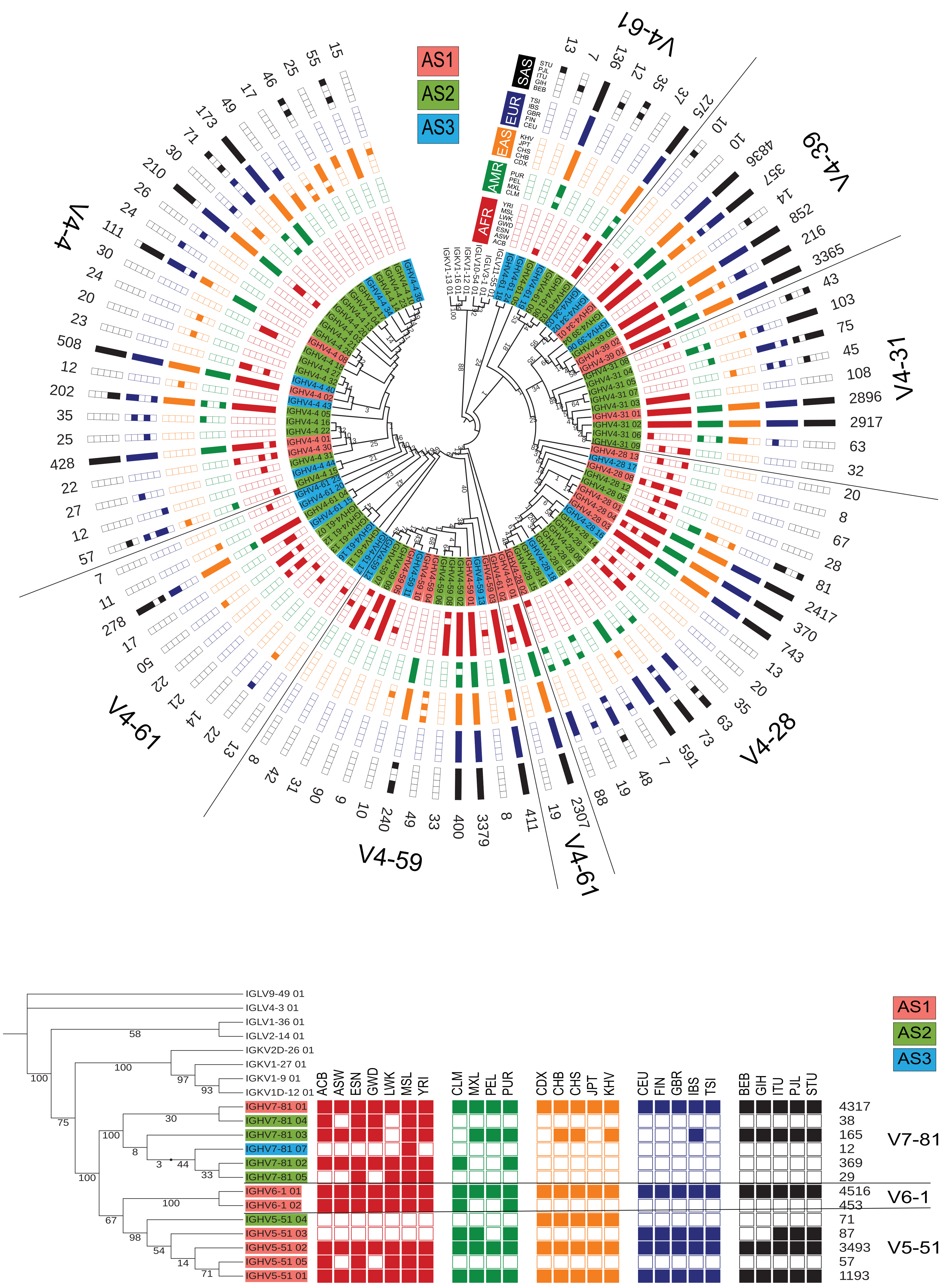

Figure S7: ML tree of the population distribution of *IGHV4*, *V5*, *V6* and *V7* family alleles. A few *IGKV* and *IGLV* alleles were used as outgroups.

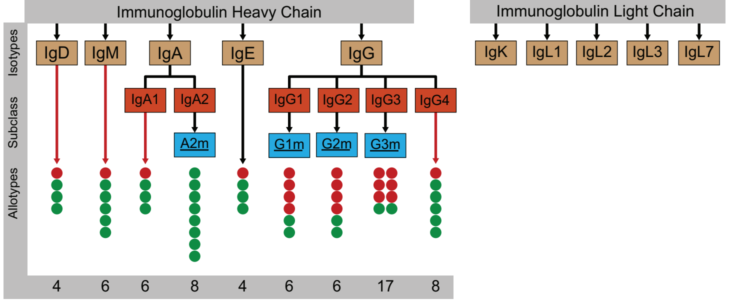

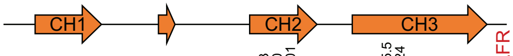

Allotype	CH2 (83 90 101)	CH3 (45.5 124)	AFR	AMR	EAS	EUR	SAS
IgA1_1	E P T	R L	578	306	652	695	485
IgA1_2	D P T	R L	166	330	325	250	450
IgA1_3	E P T	R M	30				
IgA1_4	E P A	R L	22				

Allotype	115 124	95	84 85.2 100 124 134 143	AFR	AMR	EAS	EUR	SAS
IgA2_1	P P	P	R Y D M I V	24	485	147	761	571
IgA2_2	S R	P	R Y E M I A	402	27	109	6	55
IgA2_3	P P	P	R Y E M I A	139	102	211	164	218
IgA2_4	S R	S	R Y E M I A	260				
IgA2_5	S R	P	R Y D M I A			416		34
IgA2_6	P P	P	Q Y E M I A			76		14
IgA2_7	P P	P	Q Y D M I A	54				
IgA2_8	P P	S	R Y D M I V	26				

Allotype	40	102	103	AFR	AMR	EAS	EUR	SAS
IgD_1	T	V	V	1141	620	962	983	926
IgD_2	S	V	V	119	2			
IgD_3	T	I	V	3	63	1		1
IgD_4	T	V	I	2		21		14

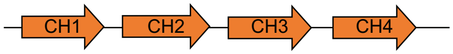

Allotype	8 12 125	AFR	AMR	EAS	EUR	SAS
IgE_1	A W P	1092	670	561	993	887
IgE_2	A G P	12	2	427		72
IgE_3	V G P	123	7		1	
IgE_4	A W A	21				

Allotype	95	35 98 126	1.1	AFR	AMR	EAS	EUR	SAS
IgM_1	D	R S V	T	598	593	613	919	819
IgM_2	D	R G V	T	297	65	211	73	96
IgM_3	D	R G V	I			177		3
IgM_4	D	R S G	T	143	6	1		
IgM_5	E	R G G	T	208	9		7	3
IgM_6	D	Q S V	T					22

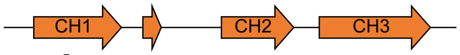

Allotype	120	12 14 101 110	AFR	AMR	EAS	EUR	SAS
IgG1_1	K	D L V A	949	286	278	176	300
IgG1_4	R	D L V A		16		30	18
IgG1_5	K	E M V A		10	377		76
IgG1_6	K	E M V G				22	11

Allotype	92 95 96	45.1	38	AFR	AMR	EAS	EUR	SAS
IgG2_1	P N F	V	A	327	413	406	447	574
IgG2_2	P N F	V	S	378	16			
IgG2_3	T N F	M	A	17	130	506	323	245
IgG2_4	P S L	V	A					7
IgG2_5	T N F	V	A		39	15	97	41
IgG2_6	P N F	M	A		41	17	96	51

Allotype	79	2	92 98	11 14 98 101 125	AFR	AMR	EAS	EUR	SAS
IgG4_1	H	S	L N	Q M E V L	485	469	310	596	507
IgG4_2	H	S	V N	Q M E V L	10	74	294	140	90
IgG4_3	H	S	V N	Q M E V P			15		
IgG4_5	H	P	L N	Q M Q I L	24				

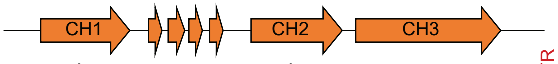

Allotype	CH1 (84.3 85.1 85 103)	CH2 (82 83 84.3 92)	CH3 (39 44 79 84 88 98 101 115 116)	AFR	AMR	EAS	EUR	SAS
IgG3_1	S S L T	P R Y T	V S N M K Q I R F	148	8			
IgG3_3	S S L T	P R F T	V S N M K Q I R F	14	135	8	368	187
IgG3_4	S S L T	L R Y T	V N N M K Q I H Y		12	18	13	59
IgG3_11	S N F T	P R Y T	V S K V K Q I H Y	24				
IgG3_12	S S L T	L R Y T	M S N V K Q I R Y		44	13	39	33

Figure S9: Isotypes, Subclasses and allotypes of *IGH* locus. The red circles in the nomenclature plot represents the number of allotypes (protein level translation of nucleotide germline alleles) matched with IMGT (similar to AS1) and green circles represents the new allotypes identified from G1K data. All the IGH constant genes are represented here. The three CH domains and one hinge region for IgA1, IgA2 and IgD is shown in 2-D diagram and the mutation existing in each domain is under the respective domain. The IgG3 protein has four hinges and three CH domains. IgM and IgE have four CH domains. The position of the amino-acid mutated is mentioned for the respective domain. The determinants marked in green represents no change in charge or structure whereas red determinant suggests the change in amino-acid properties.

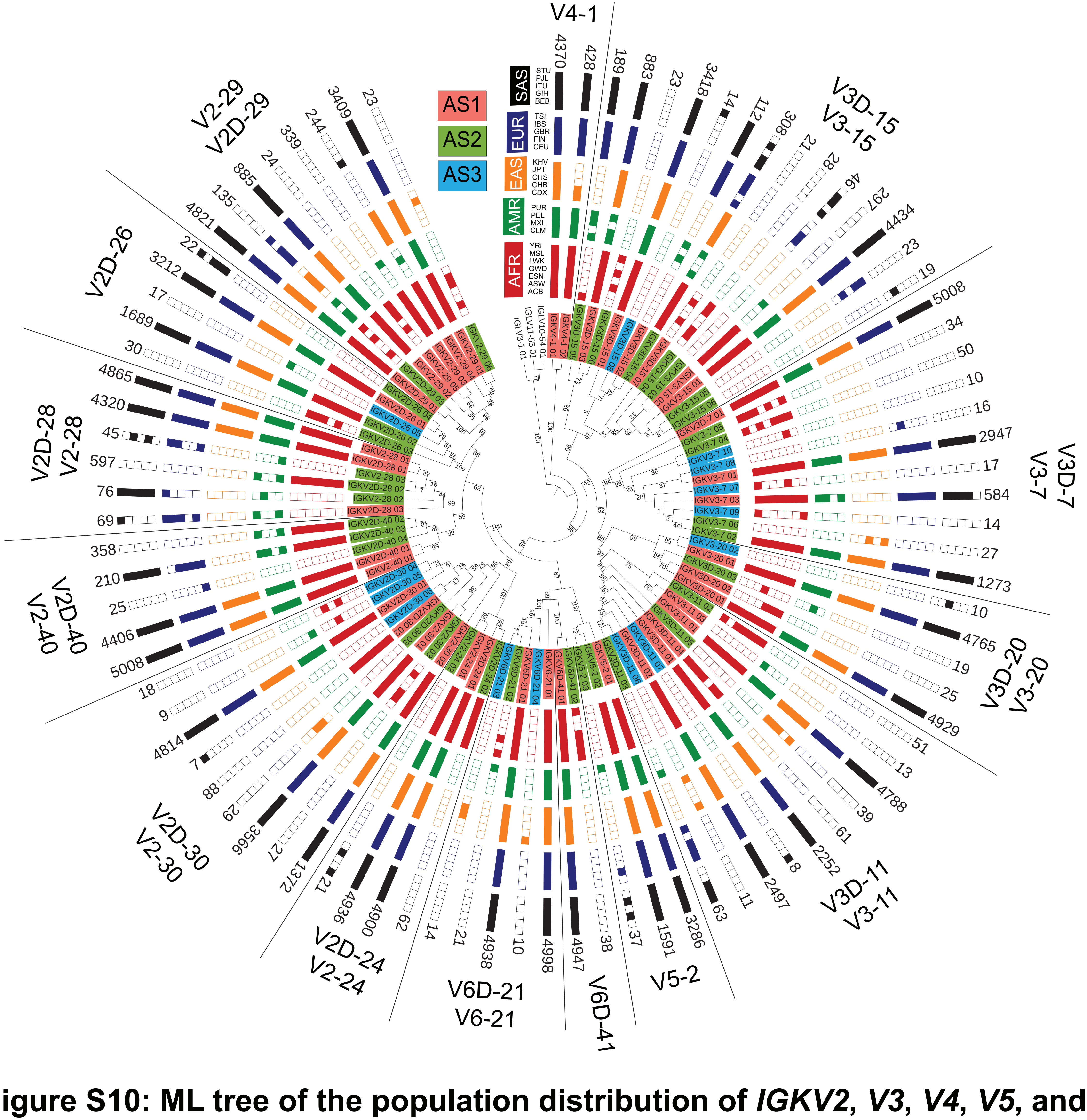

Figure S10: ML tree of the population distribution of *IGKV2*, *V3*, *V4*, *V5*, and *V6* family alleles. A few *IGLV* alleles were used as outgroups.

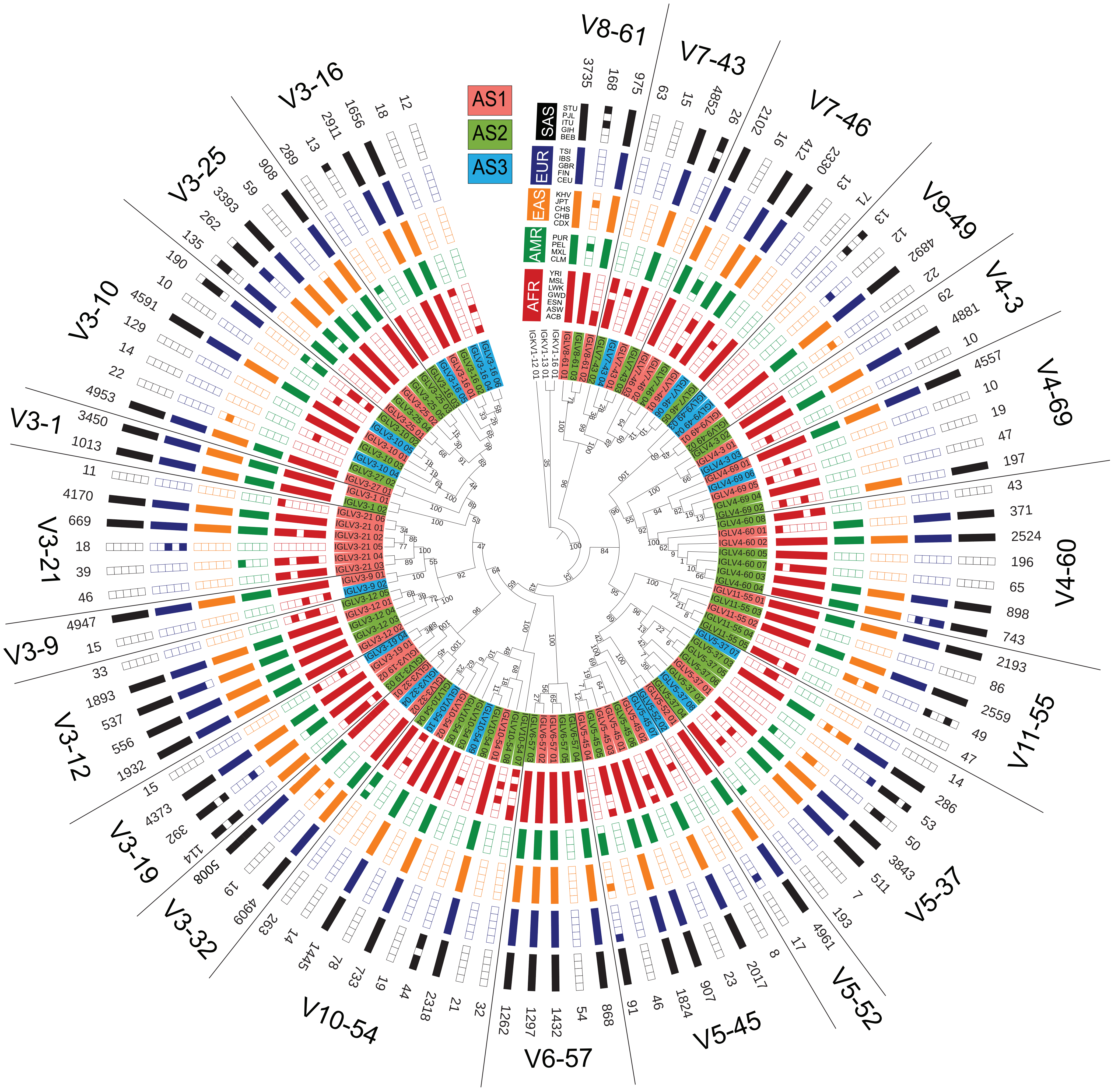

Figure S11: ML tree of the population distribution of *IGLV3*, *V4*, *V5*, *V6*, *V7*, *V8*, *V9*, *V10* and *V11* family alleles. A few *IGKV* alleles were used as outgroups.

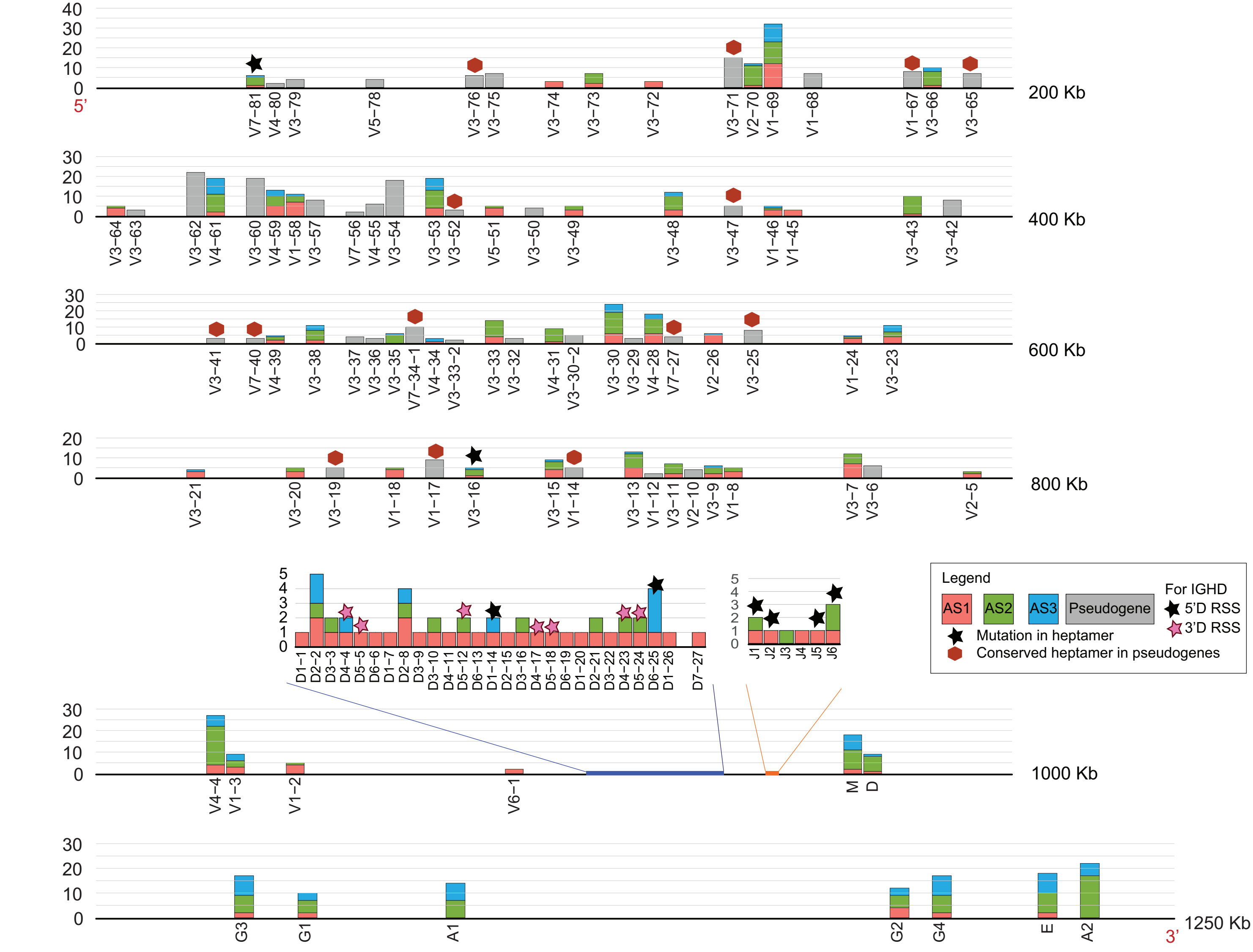

Figure S12: *IGH* alleles per gene with confidence levels in the locus representation. The alleles are marked on the locus with a confidence level AS1 (red), AS2 (green) and AS3 (blue), where the bar height represents the number of alleles. Pseudogenes are represented by grey colored bars. The Y-axis on the locus shows the absolute number of alleles. *IGHD* and *IGHJ* are projected outwards. A star indicates that the RSS sequence is mutated and it could have effect on the recombination frequency of these genes. *IGHD* have stars colored with black and blue which indicates the mutation in 5’ RSS and 3’ RSS, respectively. A red hexagon on the pseudogenes indicates the presence of conserved heptamers.

Supplementary Dataset legends

Table S1: 1000 Genomes population information for 2504 and 563 individuals. Individual Sample IDs with their source and EBV coverage is mentioned in the table below. The 563 genomes selected are marked with green shade.

Table S2: pmIG functional alleles for *IGH* Locus with confidence level information, database mapping information, full sequence and haplotype support in both population and superpopulation.

Table S3: pmIG functional alleles for *IGK* Locus with confidence level information, database mapping information, full sequence and haplotype support in both population and superpopulation.

Table S4: pmIG functional alleles for *IGL* Locus with confidence level information, database mapping information, full sequence and haplotype support in both population and superpopulation.

Table S4: Deleted pmIG functional alleles for *IGH, IGK and IGL* Locus with confidence level information, database mapping information, full sequence and haplotype support in both population and superpopulation.

Table S5: *IG* pseudogenes with conserved heptamer of RSS sequence.

Table S6: RSS sequences in pmIG functional alleles for *IGH* Locus for each allele.

Table S7: RSS sequences in pmIG functional alleles for *IGK* Locus for each allele.

Table S8: RSS sequences in pmIG functional alleles for *IGL* Locus for each allele.
